# Comparative Analysis of the Circular and Highly Asymmetrical *Marseilleviridae* Genomes

**DOI:** 10.3390/v12111270

**Published:** 2020-11-07

**Authors:** Léo Blanca, Eugène Christo-Foroux, Sofia Rigou, Matthieu Legendre

**Affiliations:** CNRS, IGS, Information Génomique & Structurale (UMR7256), Institut de Microbiologie de la Méditerranée (FR 3489), Aix Marseille Univ., 13288 Marseille, France; leoblanca18@gmail.com (L.B.); eugene.christo-foroux@igs.cnrs-mrs.fr (E.C.-F.); rigou@igs.cnrs-mrs.fr (S.R.)

**Keywords:** comparative genomics, large DNA viruses, marseillevirus, genome evolution

## Abstract

*Marseilleviridae* members are large dsDNA viruses with icosahedral particles 250 nm in diameter infecting *Acanthamoeba*. Their 340 to 390 kb genomes encode 450 to 550 protein-coding genes. Since the discovery of marseillevirus (the prototype of the family) in 2009, several strains were isolated from various locations, among which 13 are now fully sequenced. This allows the organization of their genomes to be deciphered through comparative genomics. Here, we first experimentally demonstrate that the *Marseilleviridae* genomes are circular. We then acknowledge a strong bias in sequence conservation, revealing two distinct genomic regions. One gathers most *Marseilleviridae* paralogs and has undergone genomic rearrangements, while the other, enriched in core genes, exhibits the opposite pattern. Most of the genes whose protein products compose the viral particles are located in the conserved region. They are also strongly biased toward a late gene expression pattern. We finally discuss the potential advantages of *Marseilleviridae* having a circular genome, and the possible link between the biased distribution of their genes and the transcription as well as DNA replication mechanisms that remain to be characterized.

## 1. Introduction

*Marseilleviridae* is an expanding family of large double-stranded DNA viruses infecting free-living amoeba of the *Acanthamoeba* genus. Their icosahedral capsids of 250 nm diameters enclose a 340 to 390 kb genome predicted to encode an average of 500 protein-coding genes [1,2,3,4,5,6,7,8,9,10,11,12]. Among these genes, some code for unexpected functions for a virus, the most surprising being homologues to cellular histones [1,2]. Viruses from this family belong to the NCLDVs (for nucleocytoplasmic large DNA viruses), i.e., the *Nucleocytoviricota* phylum, according to the latest International Committee on Taxonomy of Viruses (ICTV) classification [13,14]. Marseilleviruses’ replication cycles start with their phagocytosis by the *Acanthamoeba* host. Once in the cytoplasm, they form the so-called “viral factory” in the vicinity of the nucleus where virion assembly and DNA packaging occur simultaneously [1]. Mature particles are then released through cell lysis roughly 8 h post-infection (pi) [1]. However, the duration of the replication cycle is variable among *Marseilleviridae* with strains for which virions are released at 13–16 h up to 24 h pi. Since marseilleviruses encode a complete transcription apparatus and the host nucleus appears to remain intact during the entire cycle, it was initially assumed that marseilleviruses were *bona fide* cytoplasmic viruses, without a nuclear phase. However, it was subsequently shown that virally encoded RNA polymerase subunit proteins are not packaged within the virions, thus precluding the transcription of viral genes to start [10]. As a workaround, nuclear proteins are actively, albeit transiently, recruited by the viral factory to initiate the transcription of viral genes, thus placing marseilleviruses between viruses strictly replicating within the cytoplasm and those involving an intranuclear phase [10].

Marseillevirus T19 was the first *Marseilleviridae* to be isolated by co-culturing with *Acanthamoeba castellanii* [1]. Since then, several strains were isolated using the same approach, mainly from aquatic samples of different continents (Asia [9,12,15], Africa [4,5], South America [6,11], Europe [1,2,3,8] and Australia [7,10]). In addition, marseillevirus-like genomic sequences were identified in environmental metagenomics assembled data [16]. Among the isolated strains, thirteen were fully sequenced (Table S1), and their phylogeny shows that they belong to five distinct clades [10,15] (Figure S1). From the analysis of the genes encoded in these genomes, it was estimated that roughly 25% of them are of potential cellular origin, making horizontal gene transfers (HGT) a contributing factor shaping the *Marseilleviridae* genomes [1]. Surprisingly, only 23% of these exchanges involve the *Amebozoa* host, as opposed to 45% for bacteria and bacteriophages [1]. Even more remarkably, this large fraction of bacteria-related genes are subjected to strong purifying selection, and thus probably contribute to viral fitness [7]. One striking example is the *Marseilleviridae*-encoded restriction–modification (RM) system that involves restriction endonucleases and DNA methyltransferases of bacterial origin [17]. It is suspected that it serves as a weapon against amoeba intracellular parasites, thus giving to the virus a selective advantage.

Besides evolutionary questions, marseilleviruses’ physiology has been examined through several genome-wide surveys using various omics data. First, proteomic data of the viral particles of three *Marseilleviridae* members were produced, namely marseillevirus [1], noumeavirus and melbournevirus [10]. This not only revealed the proteins that build the structure of the *Marseilleviridae* virions, but also those packaged within it that could be essential for initiating the viral replication. In addition, the marseillevirus’ transcriptional activity during an infection cycle in *A. castellanii* was recently surveyed by RNA sequencing (RNA-seq), showing that the host translation apparatus is downregulated during the infection [18]. This now provides us with a sufficient body of data to conduct an in-depth comparative genomics study of the *Marseilleviridae* family.

In this study, we first experimentally confirm the circular structure of the marseilleviruses genomes. Using available genomic, proteomic and transcriptomic data, we then reveal a strong bias in the distribution of the marseilleviruses’ genes. We examine the genomic rearrangements as well as the genomic distribution of several gene categories along the genomes. More specifically, we unveil the uneven distribution of the core genes (i.e., genes conserved in all *Marseilleviridae*), the virion-associated genes and the paralogous genes (i.e., genes that were duplicated during the *Marseilleviridae* evolution). This work helps us to better understand the global organization of the *Marseilleviridae* genomes, as well as the evolution and physiology of this viral family.

## 2. Materials and Methods

### 2.1. Pulse-Field Gel Electrophoresis

A viral suspension of noumeavirus was prepared according to [10]. The viral suspension was calibrated at an OD_600_ of 0.24. Drops of 45 μL of the viral suspension were embedded in 1% low melting agarose, and the plugs were incubated in lysis buffer (50 mM Tris-HCl pH 8.0, 50 mM EDTA, 1% (*v*/*v*) laurylsarcosine, and 1 mg/mL proteinase K) for 24 h at 50 °C with light shaking (500 rpm). The lysis buffer was renewed every 8 h and 1 mM DTT was added 30 min before the second buffer change. After lysis, the plugs were washed once in sterile water and twice in TE buffer (10 mM Tris HCl pH 8.0 and 1 mM EDTA) with 1 mM PMSF, for 15 min at 50 °C. The plugs were then equilibrated in the appropriate restriction buffer and digested with 20 units of ApaI at 25 °C over night (o/n) and for 3 more hours in fresh reagent. Double digested plugs were then equilibrated in the appropriate restriction buffer and digested with 20 units of SwaI at 25 °C o/n and for 3 more hours in fresh reagent. All digested plugs were washed once in sterile water for 15 min, once in lysis buffer for 2 h at 50 °C and three times in TE buffer. Electrophoresis was carried out in 0.5× TBE using a 1% agarose gel for 20 h 18 min at 6 V/cm, 120° included angle and 14 °C constant temperature in a CHEF-MAPPER system (Bio-Rad) with pulsed times ramped from 0.47 s to 54.17 s.

### 2.2. Genome Analysis

We gathered the 13 publicly available *Marseilleviridae* complete genomes from the GenBank database (Table S1). Suspecting the tokyovirus assembly to be contaminated with *A. castellanii* mitochondrion sequences, we reassembled the original Illumina sequences [9] using the Spades assembler [19] version 3.13.0 with the “meta” parameter. This resulted in two highly covered assembled scaffolds—a 362,593 nt one, corresponding to the tokyovirus genome (Dataset S1), and a second of 41,646 nt, corresponding to the *A. castellanii* mitochondrion.

We performed a protein-coding gene re-annotation of all the sequences using the same gene-finding algorithm—GeneMarkS [20] version 4.32—with the “virus” parameter and kept the open reading frames (ORF) coding for proteins of at least 50 amino acids.

Global analysis of nucleotide sequence conservation along the genomes was performed using the mVista online tool [21] with the “Shuffle-LAGAN” alignment program and the “translated anchoring” option.

Genomic rearrangements were visualized using the ACT genome viewer [22]. We first generated pairwise genome-wide protein alignments using Promer from the Mummer package [23], converted the alignments to the “crunch” file format, and visualized the genome-wide alignments in ACT.

### 2.3. Homologous Proteins Clusering and Pangenome Analysis

Protein clustering was performed using OrthoFinder [24] version 2.4.0 with the following options: “-M msa -S blast”. For each cluster (referred to as “Orthogroup”), protein sequences were aligned using Clustal Omega [25], and phylogenetic trees were computed using IQtree [26]. The pangenome analysis was performed using the PanGP tool [27] and the Micropan R package [28]. The core genes were extracted from the orthogroups where at least one gene from each virus was present. The strain-specific genes correspond to orthogroups where genes belonged to a single virus (i.e., singletons). To avoid false positive singletons we only kept the genes that had no blastP match (E-value < 10^−5^) in the other viruses.

### 2.4. Nucleotide Biais Composition

Cumulated AT-skews and GC-skews were computed using an in-house script provided in supplements. Breakpoints of GC-skews and AT-skews in artificially rearranged chromosomes were performed using the “rearranged.oriloc” function (see [29] for a detailed explanation) from the SeqinR R package.

### 2.5. Transcriptomic Data Analyses

Raw RNA-seq data from the PRJEB34467 sequencing project [18] of the *A. castellanii* infection by marseillevirus T19 were obtained from the SRA database. The dataset covers the marseillevirus infection cycle through 9 time points: 0 h pi (ERR3528397), 1 h pi (ERR3528398), 2 h pi (ERR3528399), 4 h pi (ERR3528400), 5 h pi (ERR3528401), 6 h pi (ERR3528402), 8 h pi (ERR3528403), 10 h pi (ERR3528404) and 12 h pi (ERR3528405). Paired-end reads were mapped to the genomes of marseillevirus (GU071086), *A. castellanii* (GCA_000193105 assembly) and *A. castellanii* mitochondrion (U12386) using Hisat2 version 2.1.0 with the following options: “—rna-strandness FR—no-discordant –max-intronlen 1500”. This resulted in 95.3% of the reads being correctly aligned. Read counts and normalization in TPM values were performed using TPMCalculator [30]. Heatmap and gene-expression clustering was done on scaled log(TPM) values (centered by gene average expression and normalized by its standard deviation) using the “ComplexHeatmap” R package [31] with the following parameters: “clustering_distance_row = ‘spearman’” and “km = 3”.

### 2.6. Phylogeny and Selection Pressure Analysis

The *Marseilleviridae* phylogeny was computed using the concatenated multiple alignments of single-copy orthologous core genes and the IQtree software [26]. Bootstrap values were calculated using the ultrafast bootstrap approximation with 1000 replicates.

Selection pressure was measured based on the dN/dS (ω) ratios of marseilleviruses single copy orthologous genes using the Codeml algorithm [32] through the ete3 package [33]. For each orthogroup we computed a codon alignment based on nucleotide sequences and protein alignments. We then calculated ω values using two models: the M0 model (single ω for the whole tree) and the b_free model (distinct ω values for the gene of interest and for the rest of the tree). Each ω value was selected according to the LRT *p*-value between the models. To avoid saturation, ω values were only considered if dS ≤ 2, 0.01 ≤ dN ≤ 2 and ω ≤ 10.

## 3. Results

### 3.1. Marseilleviridae Genomes Are Circular

**Figure 1:**
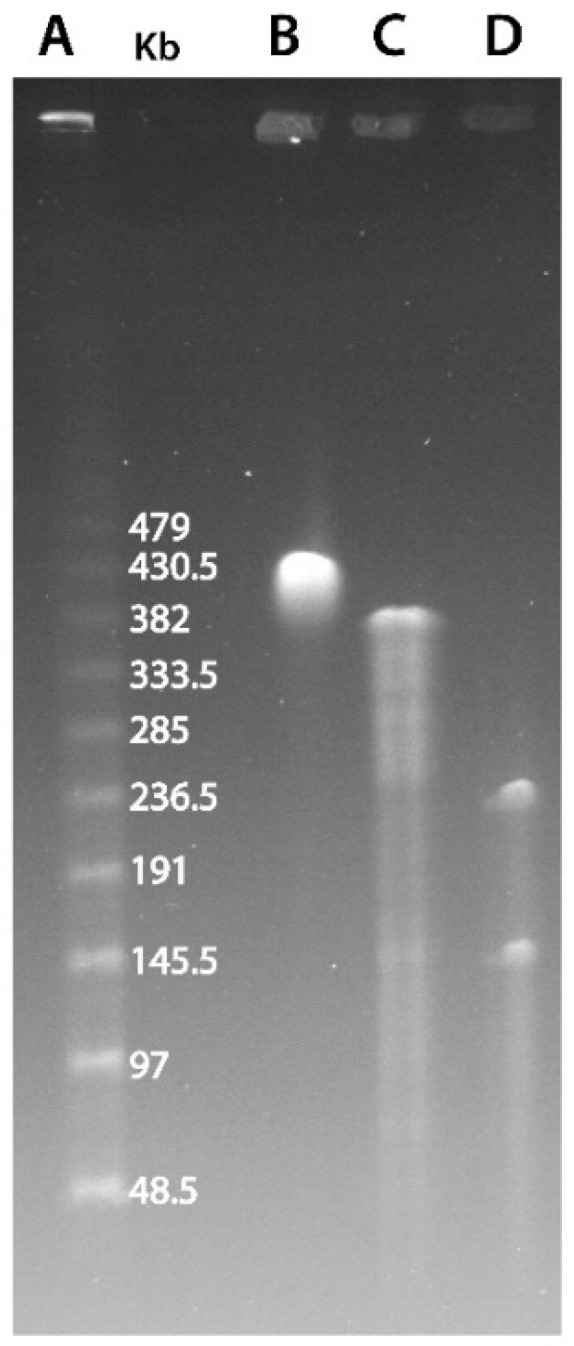
PFGE resolution of noumeavirus genomic DNA. (**A**) λ DNA ladder. (**B**) Undigested noumeavirus DNA. (**C**) Digested DNA using the ApaI restriction enzyme. (**D**) Double digestion using ApaI and SwaI restriction enzymes.

In its initial description, it was proposed that marseillevirus had a circular genome, albeit without experimental data supporting this predicted architecture [1]. The replication of a dsDNA genome involves different mechanisms depending on whether it is linear or circular. The analysis of genomic rearrangements also differs depending on the topology of the chromosome. Therefore, we first sought to experimentally confirm the circular structure of the *Marseilleviridae* genomes. We thus performed a Pulse-Field Gel Electrophoresis (PFGE) experiment combined with the restriction digestion of noumeavirus DNA, a *Marseilleviridae* belonging to the B clade (Figure S1). We used two restriction enzymes to cleave noumeavirus DNA: ApaI and SwaI. The first enzyme (ApaI) is predicted to cleave noumeavirus DNA at a single position. If the genome is linear, the digestion should result in two fragments, whereas a circular genome is expected to produce a single fragment of the size of the genome (376,207 nt). Figure 1C clearly shows a single band at the expected size of approximately 380 kb. The second enzyme (SwaI) is also predicted to cleave the DNA only once. The double digestion with both enzymes should thus produce two fragments in the case of a circular genome—one of 143 kb and a second one of 233 kb. Again, as expected, the migration of noumeavirus DNA subjected to double digestion resulted in two bands of the proper size (Figure 1D). It is well known that closed-circular supercoiled DNA moves very slowly in pulse-field gels [34]. Accordingly, the undigested noumeavirus DNA migrates slower than the single-cut one (Figure 1B,C). Altogether, these data confirm that noumeavirus DNA is circular, as are most likely all the *Marseilleviridae*.

### 3.2. Asymetry in Sequence Conservation along the Genomes

Among the isolated marseilleviruses, thirteen have a complete genome sequence (Table S1). They belong to the five currently established *Marseilleviridae* clades (Figure S1). Since the gene annotation tools and procedures used to annotate the available marseillevirus genomes are not standardized, we performed a re-annotation of the genomic sequences using the same protocol (see Materials and Methods). As shown in Table S1, gene density was consistent between strains, except for the insectomine virus that contained much more ORF, hinting at potential sequencing errors. Accordingly, the average predicted protein length was significantly smaller (Mann–Whitney *p*-value = 5.6 × 10^−79^) in this genome (89 aa) than in the other genomes (159 aa). We thus safely excluded it for the rest of the study, and only kept the twelve complete marseillevirus genomes that could be reliably compared.

We first sought to explore the large genomic rearrangements and insertions/deletions that occurred within these strains. Since *Marseilleviridae* genomes are circular, there is no reason for the assembler algorithms to start the assembly at the same position. Therefore, the genomes have to be aligned to a common starting point to be compared. We chose the strictly conserved Major Capsid Protein (MCP) gene which is encoded in a single copy in all marseilleviruses to define the starting position of the linearized genomes.

**Figure 2:**
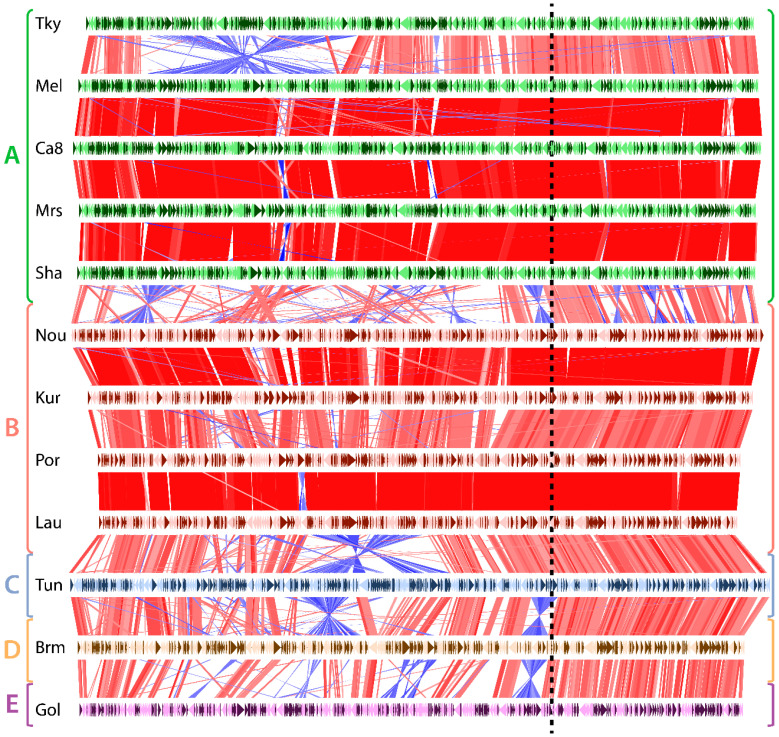
Genomic rearrangements in marseilleviruses. Each horizontal line represents a viral genome, namely: tokyovirus (Tky), melbournevirus (Mel), cannes 8 virus (Ca8), marseillevirus (Mrs), marseillevirus shanghai (Sha), noumeavirus (Nou), kurlavirus (Kur), port-miou virus (Por), lausannevirus (Lau), tunisvirus (Tun), brazilian marseillevirus (Brm) and golden marseillevirus (Gol), grouped and color-coded according to the clade it belongs to (shown on le left). Genes encoded on the forward strain are shown in dark colors and genes on the reverse strand in light colors. Vertical red and blue lines represent homologous genes between a pair of genomes. Red lines correspond to genes that are in the same direction, while blue lines represent inverted genes. The dashed vertical line separates the region prone to genomic rearrangements (on the left) from the one relatively depleted in rearrangements (on the right).

As shown in Figure 2, the pairwise comparison of *Marseilleviridae*, ordered according to their phylogeny, depicts a disparate frequency in genomic rearrangements. Unsurprisingly, rearrangement events were much more frequent between strains belonging to diverging clades than strains from the same clades. One exception, though, is tokyovirus. It contains a large inversion compared to the other marseilleviruses of the clade A. The *Marseilleviridae* phylogeny also shows that tokyovirus is the most divergent when viruses from the same clade are compared (Figure S1). All the other viruses belonging to the same clade (either clade A or B) exhibit almost perfectly collinear genomes. Oppositely, most of the inter-clade comparisons display a large amount of inversions and intrachromosomal translocations. Surprisingly, most of these rearrangements as well as insertions/deletions are not uniformly distributed along the genomes. They mostly occur in the leftmost two thirds of the genomic sequences (Figure 2). Conversely, the rightmost region of the *Marseilleviridae* genomes is virtually devoid of rearrangements. This region thus seems to be in a distinct evolutionary regime compared to the rest of the genome.

We next explored the sequence conservation of the marseilleviruses genomes at the nucleotide level. As expected, the pairwise comparison of the average nucleotide identity (ANI) computed using the OrthoANI tool [35] follows the *Marseilleviridae* phylogeny. The matrix in Figure S1 shows that pairwise ANI values range from 65.7% to 99.2%. Not surprisingly, the average pairwise ANI was significantly higher (Mann–Whitney *p*-value = 2.7 × 10^−9^) in the intra-clade than inter-clades comparisons (on average 88.75% and 69.56%, respectively).

**Figure 3:**
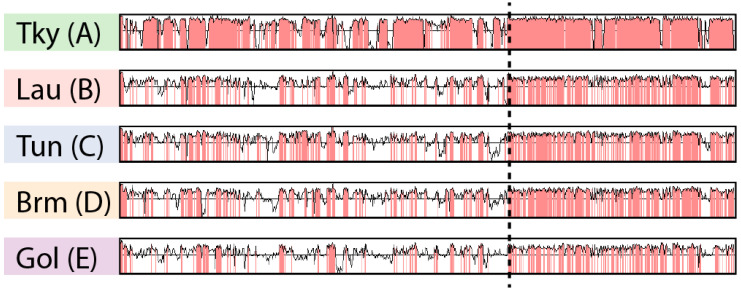
Nucleotide sequence conservation in marseilleviruses. Each row represents the nucleotide sequence identity from the glocal alignment of a *Marseilleviridae* against the marseillevirus reference. Regions with a sequence identity above 75% are highlighted in red. The following *Marseilleviridae* were used: tokyovirus (Tky), lausannevirus (Lau), Tunisvirus (Tun), Brazilian marseillevirus (Brm) and golden marseillevirus (Gol). The letters in parenthesis represent the clades the *Marseilleviridae* belong to. The dashed line separates the most conserved region from the most divergent one.

The ANI matrix (Figure S1) only gives an average estimate of sequence identity between pairs of genomes. To further explore marseilleviruses’ nucleotide sequence conservation, we analyzed its variations along the genomes. However, as mentioned earlier, several chromosomal rearrangements occurred during the *Marseilleviridae* evolution (Figure 2). In this context, a global genome alignment would not allow us to measure sequence conservation in a meaningful way. Instead, we used the Shuffle-LAGAN method [36] from the mVista tool [21]. This algorithm performs “glocal” genome alignment, which is a hybrid between local and global alignments. It first models the rearrangements between a pair of sequences and then aligns them. We compared a representative genome from each clade (tokyovirus, lausannevirus, tunisvirus, brazilian marseillevirus and golden marseillevirus) to the marseillevirus reference. Given that the viruses from clade A are highly conserved (Figure S1), we chose the most divergent one, namely tokyovirus, to compare to the marseillevirus reference and highlight potential divergent regions. It is clear from Figure 3 that sequence conservation is not uniform along the genome. Even when rearrangements are taken into account, the rightmost parts of the marseilleviruses genomes are more conserved at the nucleotide level. This mirrors our observations of the localized lower density of rearrangements in this region.

Owing to the apparent dichotomous distribution of sequence conservation within marseilleviruses genomes, we next examined potential variations in nucleotide composition. However, the overall GC-content was not found to be different between the most conserved regions (rightmost third of the genomes) and the rest of the genomes, with 44% and 43.4%, respectively. Beyond global nucleotide composition, though, asymmetries can occur over strands, with an excess of G over C (or A over T) and vice versa. Such asymmetries can be unveiled by computing the so-called cumulated GC-skew ((G − C)/(G + C)) and AT-skew ((A − T)/(A + T)) along the genomes. The Figure S2 shows the cumulated AT- and GC-skews in *Marseilleviridae* normalized by the length of each genome in order to compare all the viruses on the same scale. Although the curves are noisy, one can see a general trend for the AT-skews of all viruses with roughly constant values from the leftmost extremity of the linearized genomes to the middle, followed by a drop, a plateau and a subtle increase by the end of the genome. The amplitude of the variations is variable between the strains, but the minimal values are all roughly located from 70% to 90% of the genome lengths. A similar although more blurry trend is depicted by the cumulative GC-skew.

Nucleotide composition asymmetry is associated with several factors. The first one is the protein-coding gene orientation bias, which, due to the asymmetry of the transcription process, can lead to compositional asymmetries. Likewise, codon usage bias may cause nucleotide skews related to the asymmetry in encoded gene strands. Another main explanation is the mutation bias associated with DNA replication. In prokaryotes, the shifts in GC- and AT-skews are often correlated with the replication origin and termination sites. Analysis of nucleotide skews is thus frequently used to predict replication origins, but due to the multiple factors involved in composition asymmetry it is often a poor predictor. A workaround to uncouple the confounding factors is to artificially rearrange the genes to follow a perfect strand orientation, and analyze the GC- and AT-skews in this rearranged chromosome [29,37]. Deviations from the correlation between gene orientation skew and AT- or GC-cumulated skews are signs of replication-related asymmetries. We used this method on the *Marseilleviridae* genomes to identify breakpoints in skews and thus potential replication origin sites. As shown in Figure S2, there is a hot-spot of AT-skew breakpoints toward 80% of the marseilleviruses genomes, but there are also many AT and GC breakpoints outside this location that are distributed along the genome. Moreover, the breakpoints found using the forward and reverse strands should theoretically be co-localized, which is not the case here. Our interpretation is that a replication origin is probably present in the marseilleviruses conserved region, at roughly 20% of the rightmost extremity, but also that there are potentially multiple replication origins.

In agreement with this, we found that *Marseilleviridae* encode several copies of the predicted origin of replication binding proteins, containing the PFAM02399 protein domain. It is actually one of the *Marseilleviridae* protein families that contains the largest number of paralogs. For instance, noumeavirus encodes for as much as five different full-length copies of this protein with a recognizable protein domain. The PFAM02399 domains containing genes are evenly distributed along the genomes with no specific trend in their genomic distribution. In addition to this, there are several truncated proteins within this protein family that potentially correspond to pseudogenes, with up to six in golden marseillevirus. One can hypothesize that if the different encoded copies are functional, they may recognize different regions of the genome, in line with our suggestion of the potential multiple replication origins in *Marseilleviridae*.

### 3.3. Biaised Distribution of Core Genes

The analysis of DNA sequence conservation within *Marseilleviridae* highlighted large regions of sequence divergence (Figure 3). This prompted us to explore the pangenome of this viral family. To this end, we clustered the protein-coding genes into homologous gene families (orthogroups) using OrthoFinder [24]. Such a clustering delineates different categories of genes that are traditionally coined as “core” when they are present in all the studied strains, and “accessory” for genes not strictly conserved within strains. Among this last category, genes found in a single genome are referred as “strain-specific” genes. As shown in Table 1, the proportion of core genes is fairly constant among the marseilleviruses, with an average of 54%. Conversely, the proportion of strain-specific genes is much more variable, ranging from as low as 1% up to 14% for golden marseillevirus, with an average of 3%. These strain-specific genes can either correspond to genes only found in *Marseilleviridae*, the so-called “ORFans”, or to genes with homologs outside of the family. Here we find that 98% of the strain-specific genes are genuine ORFans, the others being HGT candidates. The very high proportion of strain-specific genes in golden marseillevirus points to an unexplored diversity of *Marseilleviridae*. To confirm this, we performed an analysis of the marseilleviruses pangenome and coregenome. Figure S3 shows the number of shared (i.e., core) genes as a function of incrementally incorporated genomes. The curve is clearly asymptotic, meaning that the pool of core genes identified from the strains under study (on average 271 gene per strain) will not evolve as new marseilleviruses are discovered. By contrast, a similar analysis of the pangenome (i.e., the total of marseilleviruses genes) displays an unsaturated curve. With an α Heap’s law parameter of 0.86 when fitting this data, the *Marseilleviridae* pangenome is considered open (α < 1) [38]. This confirms that the *Marseilleviridae*’s diversity is not fully explored yet.

**Figure 4:**
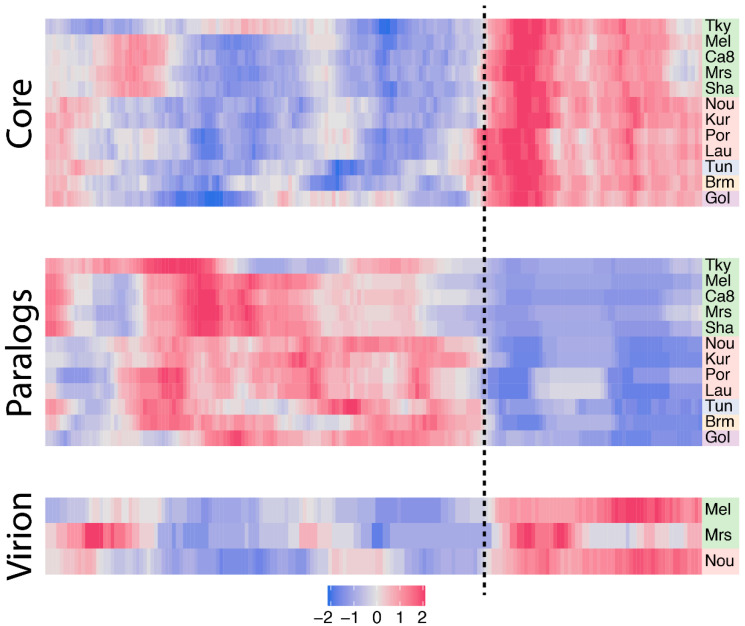
Density of core genes, paralogs and virion-associated protein-coding genes in marseilleviruses. Each row corresponds to a marseillevirus, namely: tokyovirus (Tky), melbournevirus (Mel), cannes 8 virus (Ca8), marseillevirus (Mrs), marseillevirus shanghai (Sha), noumeavirus (Nou), kurlavirus (Kur), port-miou virus (Por), lausannevirus (Lau), tunisvirus (Tun), brazilian marseillevirus (Brm) and golden marseillevirus (Gol). Strains are color-coded according to the clade they belong to, with A in green, B in red, C in blue, D in orange an E in purple. The z-score normalized density is color-coded from blue (low density) to pink (high density). The dash line separates the region of higher density in core genes, paralogs and virion-associated protein-coding genes.

Since core genes compose a large part of the marseilleviruses’ gene repertoires, we next wanted to study their distribution along the genomes, seeking for potential hot-spots. To this end, we first normalized each core gene genomic position by the length of its cognate genome. For each genome we next measured the density of core genes in a sliding window. The resulting smoothed density was next centered and scaled to a z-score according to the median value of all the windows to highlight variations in core gene densities. The heatmap presented in Figure 4 clearly reveals a strong asymmetry in marseilleviruses core gene densities. Again, the rightmost part of the genomes is strongly enriched in core genes compared to the rest of the genomes. This pattern is shared by all viruses, regardless of the clades they belong to. This region roughly corresponds to a third of the genomes.

Since the rightmost region of the *Marseilleviridae* genomes, now referred to as the “core region”, is enriched in core genes (Figure 4) and is more conserved (Figure 3), we reasoned that it could be subjected to a different selection pressure. To test this hypothesis, we computed the ratios (ω) of non-synonymous mutation rates (dN) over synonymous mutation rates (dS) of orthologous genes using the Codeml program [32] (see Materials and Methods). The distribution of ω values along normalized genomic positions is shown in Figure S4. Globally, all the genomic positions are subjected to strong negative selection (ω << 1) whatever the region, confirming the selection pressure previously measured on melbournevirus genes [7]. However, genes from the core region have more homogeneous ω values and seem to be under marginally, although statistically significant, stronger purifying selection compared to the rest of the genome, with an average ω of 0.097 and 0.156, respectively (Mann–Whitney *p*-value = 2.7 × 10^−94^).

Following the analysis of core genes, we next explored gene duplication events. More specifically, we analyzed the orthogroups previously defined (see Materials and Methods) and categorized genes into two bins: single copy genes and duplicated genes (paralogs). The vast majority of marseilleviruses genes are single copy genes with an average of 78% per virus (Table 1). Symmetrically, the proportion of duplicated genes is low (22%), and is even significantly lower in strain-specific genes, with only 2.8% in this category (Fisher’s exact test *p*-value = 6.5 × 10^−9^). We then again investigated the densities of duplicated genes along the genomes using the method previously described (Figure 4). Contrary to core genes, the paralogs are mostly present in the leftmost part of the genomes.

### 3.4. Biaised Distribution of Virion-Associated Proteins and Late-Expressed Transcripts

In viruses, genes coding for the proteins present in the virions, in particular the structural proteins that build the particles, are thought to be among the most conserved ones. There are now three *Marseilleviridae* members for which the viral particles’ proteome compositions have been analyzed by mass spectroscopy [1,10]. Two of them, marseillevirus and melbournevirus, belong to clade A, and the third one, noumeavirus, to clade B. To verify this assumption in *Marseilleviridae*, we analyzed the overlap between the core genes and genes coding for virion-associated proteins. The proportion of core genes in virion-associated proteins is remarkably constant among strains, with 83.5% in melbournevirus, 83.7% in marseillevirus and 83% in noumeavirus. These values are also significantly higher than the proportion of core genes in proteins not identified in virion proteomes, corresponding to 43.9%, 48.9% and 44.4%, respectively, with Fisher’s exact test *p*-values of 4.2 × 10^−14^, 9 × 10^−6^ and 1.9 × 10^−15^. Furthermore, we know from previous work that *Marseilleviridae* particle proteomes are well conserved, with a high correlation in their respective protein contents [10]. Altogether this means that *Marseilleviridae* particles are not strictly composed of core genes, they mostly contain these types of proteins, and the same core genes are used in different viruses’ particles. Knowing this, we expected the virion-associated protein-coding genes to be asymmetrically distributed along the marseilleviruses genomes, and indeed, the density of virion-associated genes is clearly biased to the core region previously identified (Figure 4).

The global transcriptome of *A. castellanii* infection by marseillevirus has been studied by RNA-seq through a replicative cycle [18]. We used this dataset to test whether the marseillevirus’ transcriptional activity was also regionalized along the genome. Starting from the raw sequence data, we mapped the reads to the *A. castellanii* and marseillevirus genomes, and computed a normalized expression value for each gene (see Materials and Methods). As previously observed [18], we found the marseillevirus’ genes expressions to be clustered into three main classes: early, intermediate and late (Figure S5). Early genes are expressed from the beginning of the cycle, with a peak of transcriptional activity between 1 h pi and 2 h pi, intermediate genes are mostly expressed between 1 h pi and 4 h pi, and late genes from 4 h pi until the end of the cycle. We next focused on the marseillevirus virion-associated protein-coding genes to check whether they were expressed in a time-dependent manner. Confirming the results previously obtained [18], we found an enrichment in virion-associated genes in the late expression class. In addition, we also found that marseillevirus genes orthologous to melbournevirus and noumeavirus virion-associated genes were enriched in that category (Figure S5). Finally, although the bias was less pronounced, we found that core genes were statistically enriched in the late expression class (61%) compared to the intermediate (41%) and early (43%) expression classes. Conversely, strain- and clade-specific genes are not specifically enriched in one of those classes (Chi-square *p*-value = 0.1).

We next wondered whether the genes encoded in the core region had a higher transcriptional activity than those in the rest of the genome. To this end we measured the global expression level of each marseillevirus gene by summing the expression value of all time points. As shown in Table 2, when comparing the genes from the core region from the rest, we found no statistical difference in global expressions (Student’s *t*-test *p*-value = 0.29). However, taking the summed time-point expressions as a proxy of global gene expression might introduce a bias, since late genes are only expressed at the end of the cycle, thus they might contribute to a lesser extent. To overcome this potential bias, we also analyzed the maximal time-point expression of each gene. Again, we did not find a higher expression for core region-encoded genes with this metric (Student’s *t*-test *p*-value = 0.43). Altogether, these data suggest that core region-containing genes had no particular behavior in terms of expression strength.

Finally, we analyzed the transcriptional activity of the core region regarding the expression timing. As shown in Table 2, the frequency of late genes (70%) was significantly higher (Chi-square *p*-value = 1.96 × 10^−7^) in the core region compared to the rest of the genome (44%). Thus the core region seems to be mostly expressed at the end of the replication cycle.

## 4. Discussion

Double-stranded DNA viruses have structurally diverse genomes that are either linear or circular. The first *Marseilleviridae* to be isolated (marseillevirus) was predicted to have a circular genome [1]. However, no formal proof was given in this initial work to validate this assumption, nor in the following studies describing new isolates from this family [1,2,3,4,5,6,7,8,9,10,11,12]. Assuming a genome structure without experimental evidence can impair our understanding of the physiology of the viral family, as the mechanism by which genetic material is replicated depends on that topology. A recent study of the faustoviruses, for instance, showed that the initially assumed circular viral chromosomes were actually linear [39]. It is thus essential to experimentally validate predicted genome structures. In this work, we confirmed and demonstrated that *Marseilleviridae* have circular genomes.

Surprisingly, circular genome topology is rather unusual among the numerous large and giant viruses infecting amoeba. From the eight viral families described so far, only two exhibit a circular genome: the Pithoviruses (with pithoviruses [40], cedratviruses [17,41] and orpheovirus [42]) and the *Marseilleviridae*. The six remaining families, namely the *Mimiviridae* [43], the pandoraviruses [44], the molliviruses [45], the faustoviruses [39], the pacmanviruses [46] and medusavirus [47], are all predicted, based on sequencing read mapping and genome assembly, to exhibit linear genomes. Then what would be the advantage, if any, of a virus encoding its genes in a circular chromosome? One possibility would be to escape exonuclease enzymatic activity. An example can be found in the *Escherichia coli* bacteria, which use the RecBCD exonuclease as a weapon against invading bacteriophages that contain free-ends DNA [48]. With the bacterial genome being circular, it is not subjected to exonuclease activity. Some bacteriophages counteract this attack by encoding inhibitors of RecBCD, such as the Gam protein encoded by the phage lambda [48,49]. An analogous escape mechanism could be at play here, whereby the circular structure of the *Marseilleviridae* genomes could lead them to escape exonucleases either encoded by the host, intracellular bacteria infecting the amoeba, or even other viruses in the case of coinfection. These kinds of virus–host and virus–pathogens interactions somehow relates to the ones driven by the *Marseilleviridae* encoded restriction–modification systems [17]. In that case, the viruses use endonucleases to digest competing pathogens’ DNA inside the amoeba while protecting themselves against degradation by methylating their own DNA. Here, the genome topology by itself would be sufficient to escape exonuclease activity.

*Marseilleviridae* are thought to be prone to frequent HGT, with roughly a quarter of their genes suspected to be acquired through this route [1]. Surprisingly, supposed gene exchanges with bacteria are even more frequent than the ones involving their amoebic host [1]. Indeed, with as much as 45% of all potential cell–virus gene exchanges, this represents an unexpected proportion. This might relate to the fact that *Acanthamoeba* are infested by a large variety of bacterial parasites or symbionts [50]. However, in other viruses, such as the giant pandoraviruses, which have been scrutinized for HGTs, it was shown that bacteria only account for 20% of the exchanges related to cellular organisms [51]. This proportion even drops to 13% when considering cell-to-virus transfers specifically. Yet these two viral families infect the exact same host, and thus face the same environment. So, there might be other determinants explaining the higher proportion of bacteria-related exchanges in *Marseilleviridae*. The genome structure could be one of them. Considering that *Marseilleviridae* adopt a bacterial-like circular genome, one could hypothesize that this topology somehow favors genetic exchanges, leading in certain cases to a selective advantage, as exemplified by the negative selection pressure acting on bacterial-like genes [7]. The *Marseilleviridae* RM systems are a striking example of such transfers [17]. It is noteworthy that the only giant virus family exhibiting circular genomes, the pithoviruses, also contains a high proportion (38%) of cell–virus potential gene exchanges related to bacteria, although it only accounts for 8% of the total gene set [40]. This again supports the hypothesis that a circular genome topology might facilitate gene transfers with this domain.

Following the discovery of the second *Marseilleviridae* strain (lausannevirus) [2], the authors noticed an asymmetry in the distribution of its genes along the genome. They unveiled an enrichment in annotated genes (i.e., with a predictable function based on sequence homology) on one side of the lausannevirus genome, and an opposite enrichment in “hypothetical protein” genes on the other side. They also noticed localized hot-spots of sequence rearrangements between lausannevirus and marseillevirus [2]. In this study, we expanded the comparative analysis to the twelve complete *Marseilleviridae* genomes that could be reliably compared. Our data clearly show a strong asymmetry in the *Marseilleviridae* genomes, with one region, namely the core region, corresponding to roughly a third of the genome that exhibits several peculiar properties. We first revealed that this region is virtually devoid of genomic rearrangements, while these frequently occurred in the course of the *Marseilleviridae* evolution. Accordingly, this region is also more conserved at the nucleotide level. Strikingly, the density of core genes is also much higher in this region. Such a regionalized distribution of family core genes has already been observed in other amoeba-infecting giant viruses, all of which having linear genomes. For instance, the viruses with the largest known genomes so far, the pandoraviruses, exhibit a regionalized enrichment of core genes in the first half of their genomes [51]. A similar dichotomous distribution was revealed in the distantly related molliviruses, where genes shared between molliviruses and pandoraviruses are also co-localized in half of their genomes [52]. Likewise, in faustoviruses, sequence conservation is not uniformly distributed, although it displays a different pattern with greater sequence divergence in the middle of the genome and at the extremities [39]. Somehow this relates to the strongly biased distribution of conserved gene order observed in the central part of the *Mimiviridae* genomes, as compared to the shuffled extremities [53]. Thus there are clearly different patterns of sequence conservation asymmetry in giant viruses infecting amoeba. Yet regardless of the genome structure, be it circular or linear, this asymmetry seems to be a common trait. Beyond amoeba-infecting giant viruses, the *Poxviridae*, also members of the *Nucleocytoviricota* phylum, retained most of the conserved genes in the central part of their genomes [54].

Besides the regionalized enrichment of core genes at a specific genomic location, we showed that *Marseilleviridae* genes coding for proteins detected in viral particles are also clustered together. Thus, whether they build the particles or are involved in the early phase of the infection, “important” viral genes are clustered in the core region. Then how could we explain such a regionalization? The globally late expression of the core region encoded genes might be a key to understanding this pattern. In mimivirus, the transcriptional time-dependent activity is clearly governed by the strict conservation of sequence motifs in gene promoters [55,56]. Thus genes do not need to be located in a specific genomic region to activate their expression in a time-dependent manner. On the contrary, the analysis of the marseillevirus’ transcriptome failed to unveil sequence motifs explaining gene expression patterns [18]. In that case, clustering the genes in a confined region of the genome might be a good strategy to activate gene expression at the right time. The transcriptional switch could then be done thanks to a particular topology of the DNA in that region. In that context, one has to keep in mind that *Marseilleviridae* have the astonishing ability to encode histones [1,2]. These could play a role in the transcriptional regulation of this specific genomic region. In other words, one can hypothesize that the core region’s transcriptional dynamics are controlled by DNA-dependent topological properties. It is also worth mentioning that *Marseilleviridae* probably use the host-encoded transcription apparatus in the early phases of the infection, and then switch to the viral encoded apparatus as soon as the viral RNA polymerase is available [10]. Thus genes from the core region might by controlled by the latter transcriptional system.

In circular bacterial genomes, genes tend to be less conserved with the increasing distance from the origin of replication [57,58]. Essential and highly expressed genes are usually located near the replication origin, and this is especially true for transcription- and translation-related genes [58]. One explanation for this correlation is the replication-associated gene dosage. As replication starts, genes located near the site of replication origin are in two copies, thus are more expressed than the ones located near the replication terminus, which remain in one copy [59]. Owing to the fact that *Marseilleviridae* genomes are (i) circular and (ii) highly asymmetrical, with core genes being clustered together, we explored the possibility of a relation with the distance to their origin of replication, akin to what was observed in bacterial chromosomes. Our work suggests that *Marseilleviridae* might share a replication origin located in the core region, thus resembling what is observed in the bacterial world. Next, we analyzed the proteins potentially involved in replication origin recognition. Our work revealed that *Marseilleviridae* surprisingly encode many copies of these types of proteins. If the different copies are functional, one can hypothesize that they recognize different sequence-specific sites. Our detection of several compositional strand biases argues for the presence of multiple dispersed replication origins, as seen in the circular archaea genomes [60]. However, sequence analysis is clearly limited to uncover such subtle genomic signals. A solution would be to use deep sequencing methods to uncover replication origins. The application of such a method to another member of the *Nucleocytoviricota*, the vaccinia *Poxviridae*, allowed the replication origins to be mapped at a single base pair resolution [61]. They happen to be located near the ends of this covalently closed linear genome, at the concatemer junctions. This mechanism could be shared with giant viruses with the same DNA topology. However, the question remains open for viruses with circular genomes, such as the *Marseilleviridae*, and deserves to be approached experimentally.

Our analysis of the *Marseilleviridae*’s gene content highlighted an open pangenome, meaning that the *Marseilleviridae*’s diversity has not been fully uncovered yet. This is mainly exemplified by the relatively large fraction of strain-specific genes in golden marseillevirus. Paradoxically, the recent works in the metagenomic data analysis of giant viruses through the assembly of huge datasets revealed very few metagenome-assembled genomes (MAG) related to the *Marseilleviridae* [62]. They seem to be nearly absent from the environmental microbial data. This might highlight the limits of such methods in revealing the true diversity of giant viruses in the wild, or indicate that environments containing *Marseilleviridae* have not been correctly sampled yet. We believe that future studies will be needed to isolate and characterize new *Marseilleviridae* members so as to fully comprehend this viral family.

## Figures and Tables

**Table 1:** Counts and frequencies of core, strain-specific, single-copy and paralogous genes in marseilleviruses.

	Core	Strain-Specific	Single Copy	Paralogs
**Tokyovirus**	269 (55%)	35 (7%)	396 (81%)	95 (19%)
**Melbournevirus**	265 (52%)	6 (1%)	409 (81%)	96 (19%)
**Cannes 8 virus**	268 (53%)	3 (1%)	407 (80%)	103 (20%)
**Marseillevirus**	264 (52%)	7 (1%)	402 (79%)	107 (21%)
**Marseillevirus shanghai**	266 (53%)	3 (1%)	404 (80%)	101 (20%)
**Noumeavirus**	277 (55%)	16 (3%)	394 (78%)	113 (22%)
**Kurlavirus**	272 (55%)	12 (2%)	381 (77%)	114 (23%)
**Port-miou virus**	269 (57%)	8 (2%)	383 (82%)	95 (18%)
**Lausannevirus**	266 (58%)	3 (1%)	375 (81%)	86 (19%)
**Tunisvirus**	281 (52%)	31 (6%)	385 (71%)	155 (29%)
**Brazilian marseillevirus**	272 (56%)	13 (3%)	373 (77%)	114 (23%)
**Golden marseillevirus**	282 (52%)	76 (14%)	373 (69%)	170 (31%)

**Table 2:** RNA-seq gene expression in marseillevirus.

	Early Expressed Genes Counts (%)	Intermediate Expressed Genes Counts (%)	Late Expressed Genes Counts (%)	* Maximal Expression (Mean ± SD)	* Total Expression (Mean ± SD)
**Core-region**	11 (7%)	39 (23%)	116 (70%)	7.4 ± 1.3	52 ± 9.8
**Other region**	50 (15%)	143 (42%)	150 (44%)	7.3 ± 1.3	51 ± 9.8

* RNA-seq expression is measured in log(TPM).

## Supplementary Materials

The following are available online at https://www.mdpi.com/1999-4915/12/11/1270/s1, Figure S1: Nucleotide-level genomic similarity between *Marseilleviridae*. Figure S2: Identification of potential origins of replication using cumulative AT-skew and GC-skew, Figure S3: Core-genome and pan-genome of the *Marseilleviridae*, Figure S4: Selection pressure along the *Marseilleviridae* genomes, Figure S5: RNA-seq marseillevirus gene expression, Table S1: Complete marseilleviruses sequenced genomes. Computer code: In-house python script used to compute cumulative AT-skew and GC-skew. Dataset S1: Reassembled genomic sequence of tokyovirus.
