# Supplementary material for: Comparative Analysis of the Circular and Highly Asymmetrical Marseilleviridae Genomes
Source: Viruses. 2020 Nov 7;12(11):1270. doi: 10.3390/v12111270 (PMC7695187; doi:10.3390/v12111270)
Supplement: Supplementary file 1 [file viruses-12-01270-s001.pdf]

Supplementary material

Comparative Analysis of the Circular and Highly Asymmetrical *Marseilleviridae* Genomes

Léo Blanca [1], Eugène Christo-Foroux [1], Sofia Rigou [1] and Matthieu Legendre [1,*]

[1] Aix Marseille Univ., CNRS, IGS, Information Génomique & Structurale (UMR7256), Institut de Microbiologie de la Méditerranée (FR 3489), 13288 Marseille, France

* Correspondence: legendre@igs.cnrs-mrs.fr

Table S1

Table S1. Complete marseilleviruses sequenced genomes

Strain	Clade	Genome length (nt)	# ORFs	Source	Reference	GenBank Accession
**Marseillevirus**	A	368454	509	Water of a cooling tower	(Boyer et al., 2009) http://dx.doi.org/10.1073/pnas.0911354106	GU071086
**Lausannevirus**	B	346754	461	Drinking water treatment plant	(Thomas et al., 2011) http://dx.doi.org/10.1111/j.1462-2920.2011.02446.x	HQ113105
**Cannes 8 virus**	A	374041	510	Water from a cooling tower	(Aherfi et al., 2013) http://dx.doi.org/10.1007/s11262-013-0965-4	KF261120
**Insectomime virus**	C	386631	731	Internal organs of Eristalis tenax larvae	(Boughalmi et al., 2013) http://dx.doi.org/10.1159/000354560	HG428764
**Tunisvirus**	C	380011	540	Water from a decorative fountain	(Aherfi et al., 2014) http://dx.doi.org/10.1007/s00705-014-2023-5	KF483846
**Brazilian marseillevirus**	D	362276	487	Water sample of an urban lake	(Dornas et al., 2016) http://dx.doi.org/10.3390/v8030076	KT752522
**Melbournevirus**	A	369360	505	Muddy fresh water	(Doutre et al., 2014) http://dx.doi.org/10.1128/JVI.02414-14	KM275475
**Port-miou virus**	B	349275	468	Brackish Submarine Spring	(Doutre et al., 2015, p.) http://dx.doi.org/10.1128/genomeA.01148-15	KT428292
**Tokyovirus**	A	362593	491	Water/soil sample from the Arakawa River	(Takemura, 2016) https://dx.doi.org/10.1264%2Fjsme2.ME16107	Reassembled (Dataset S1)
**Noumeavirus**	B	376207	507	Muddy sample of fresh water	(Fabre et al., 2017) http://dx.doi.org/10.1038/ncomms15087	KX066233
**Golden marseillevirus**	E	360610	543	Golden mussels	(Dos Santos et al., 2016) https://doi-org.insb.bib.cnrs.fr/10.1038/srep35237	KT835053
**Kurlavirus**	B	361368	495	Sewage water sample	(Chatterjee and Kondabagil, 2017) http://dx.doi.org/10.1007/s00705-017-3469-z	KY073338
**Marseillevirus shanghai**	A	368078	505	Unknown	Unpublished	MG827395

Figure S1

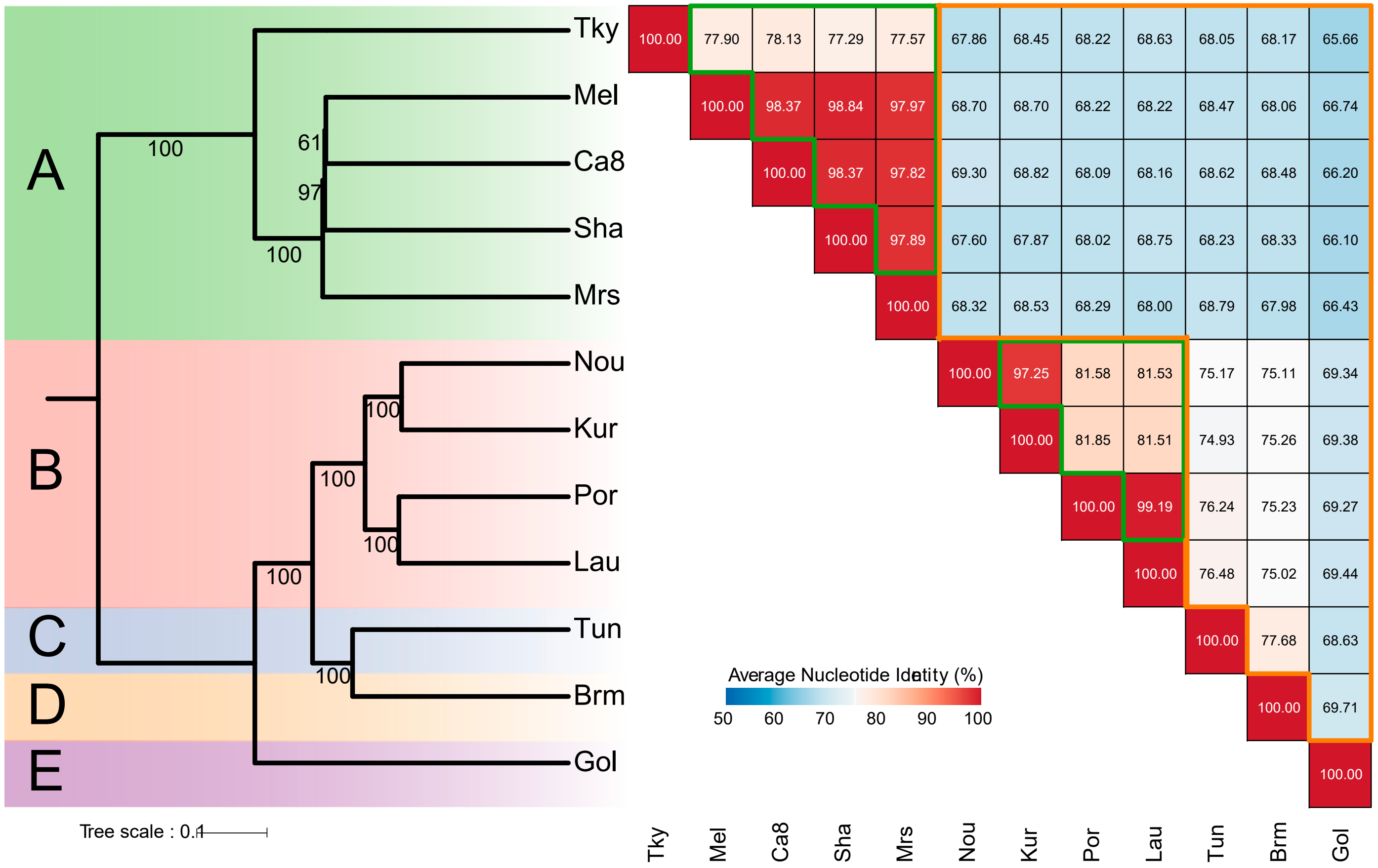

Figure S1. Nucleotide-level genomic similarity between *Marseilleviridae*. The matrix at the right shows the color-coded values of Average Nucleotide Identity (ANI) between pairs of marseilleviruses genomes. Values highlighted in green correspond to intra-clades comparisons while those highlighted in orange correspond to inter-clades comparisons. The phylogenetic tree on the left was computed from the concatenated alignment of single-copy orthologous core genes (see Materials and Methods). Each *Marseilleviridae* clade is labeled and color-coded with tokyovirus (Tky), melbournevirus (Mel), cannes 8 virus (Ca8), marseillevirus shangai (Sha) and marseillevirus (Mrs) in clade A (green); noumeavirus (Nou), kurlavirus (Kur), Port-miou virus (Por) and lausannevirus (Lau) in clade B (red); tunisvirus (Tun) in clade C (blue); Brazilian marseillevirus (Brm) in clade D (orange) and golden marseillevirus (Gol) in clade E (purple).

Figure S2

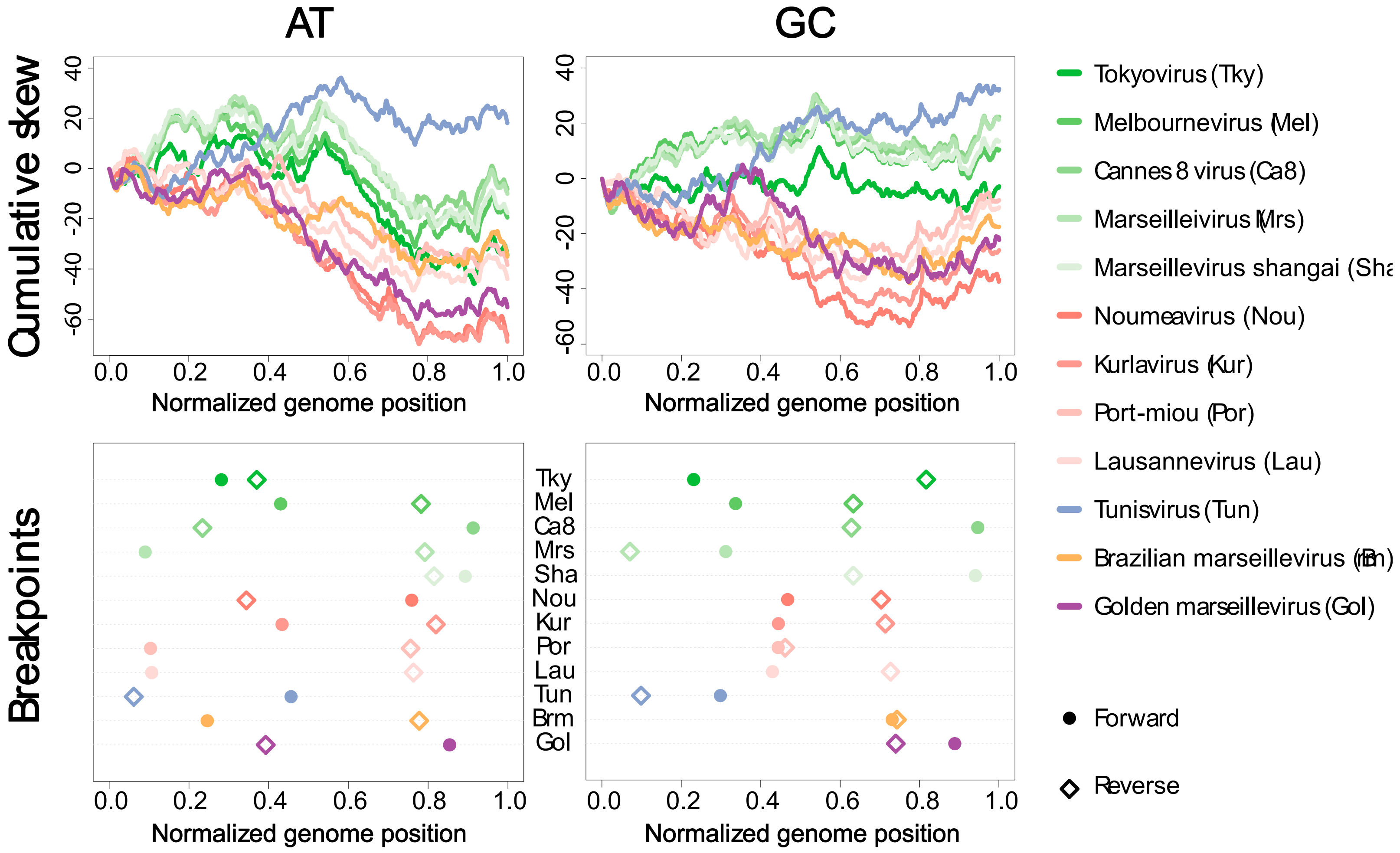

Figure S2. Identification of potential origins of replication using cumulative AT-skew and GC-skew. Each viral genome is color-coded using a similar color for viruses that belong to the same clade (A in green, B in red, C in blue, D in orange and E in purple). Breakpoints in the correlation of cumulative CDS skew and composition skews were calculated using the "rearranged.oriloc" function from the SeqinR R package (see Materials and Methods for details). Genomic positions were normalized by the length of each genome.

Figure S3

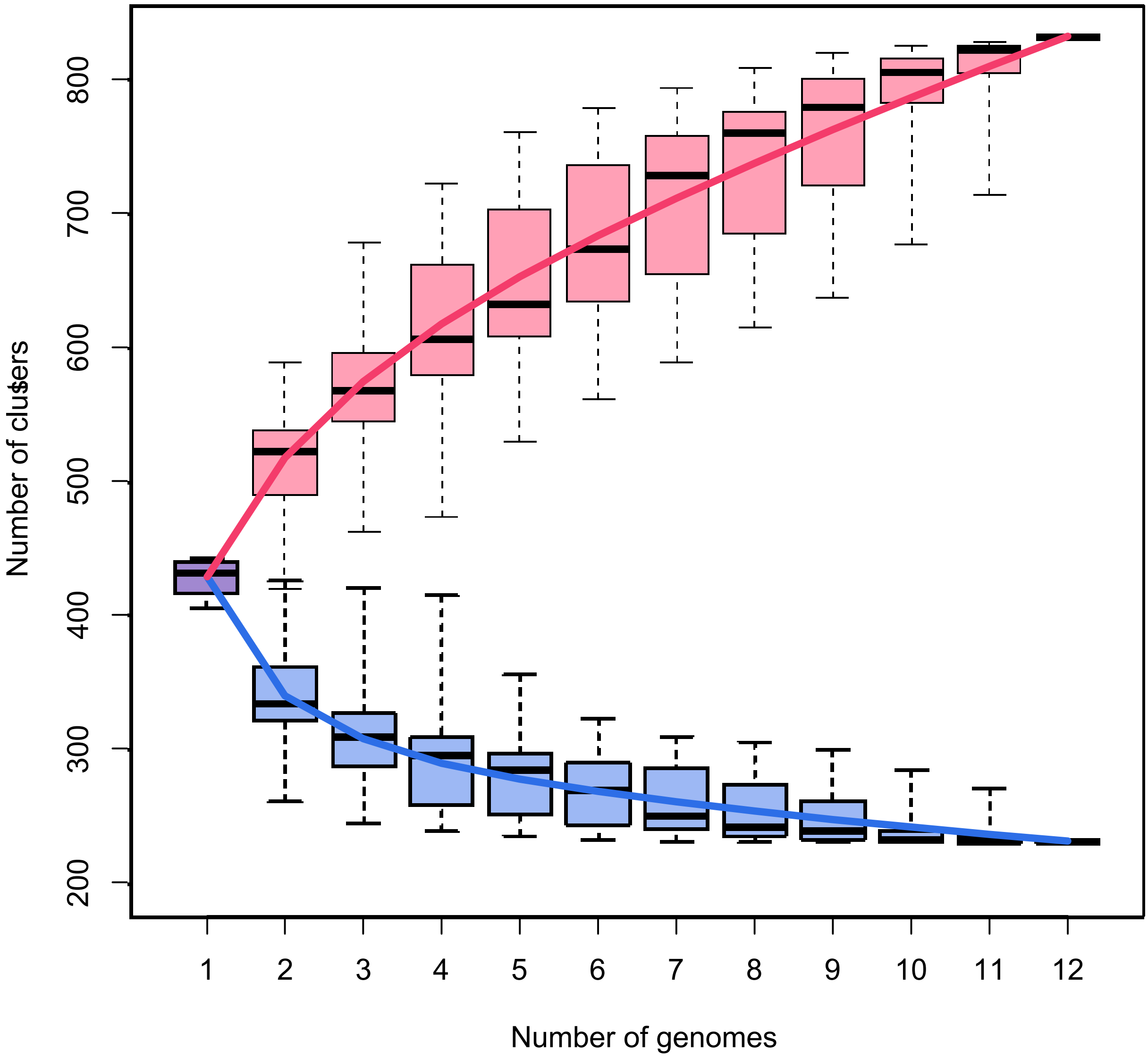

Figure S3. Core-genome and pan-genome of the *Marseilleviridae*. The box plots show the number of shared orthogroups (in blue) and the total number of orthogroups (in pink) as a function of the number of genomes being compared. Box plots show the median, the 25th and 75th percentiles. The whiskers correspond to the extreme data points. The curves correspond to average values.

Figure S4

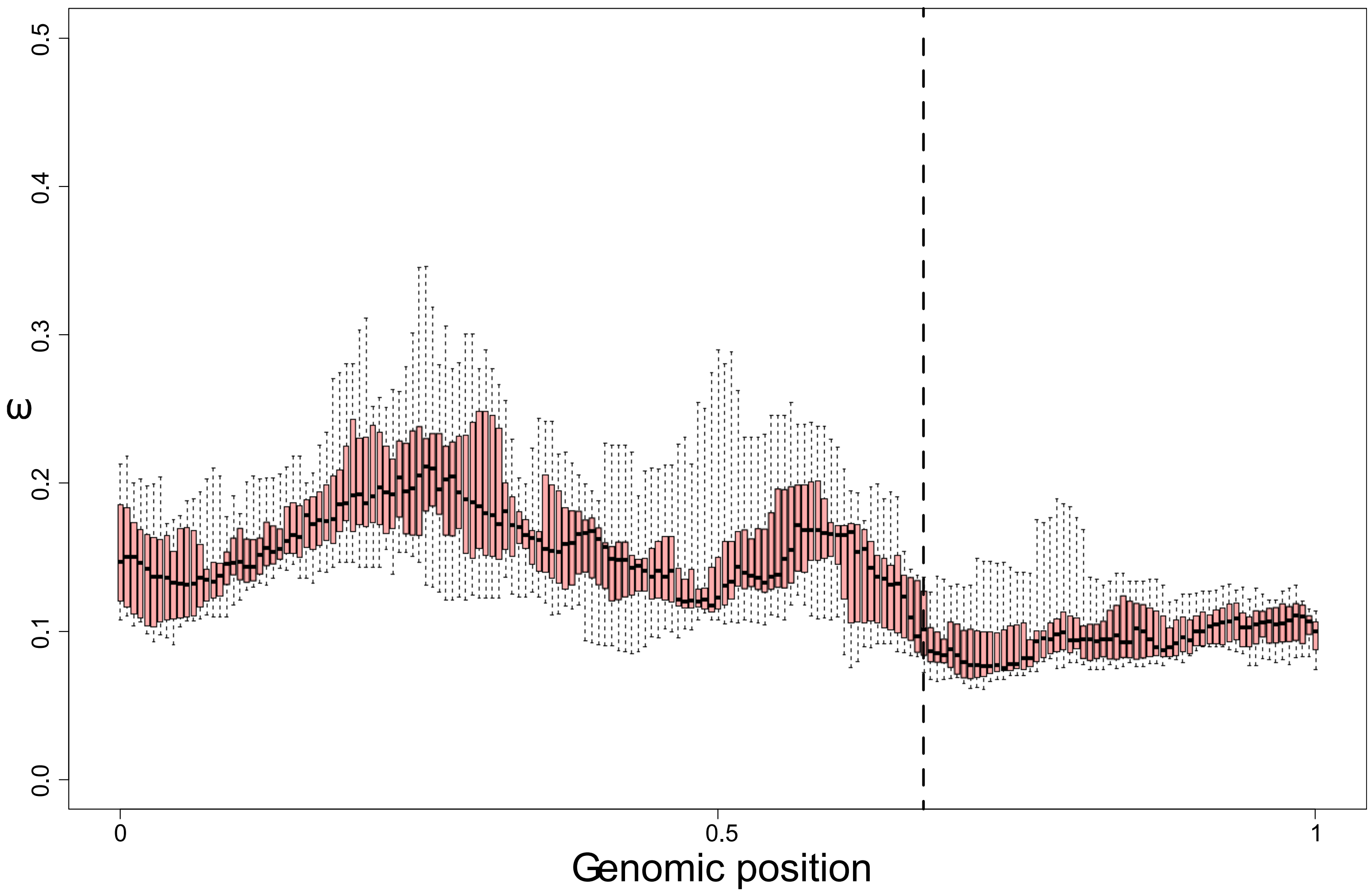

Figure S4. Selection pressure along the *Marseilleviridae* genomes. Omega values were calculated along the marseilleviruses genomes whose genomic positions were normalized by the length of each genome. Omega values were binned along the genome according to the relative genomic positions. Box plots show the median, the 25th and 75th percentiles. The whiskers correspond to the extreme data points. The dashed line separates the core region from the rest of the genome.

Figure S5

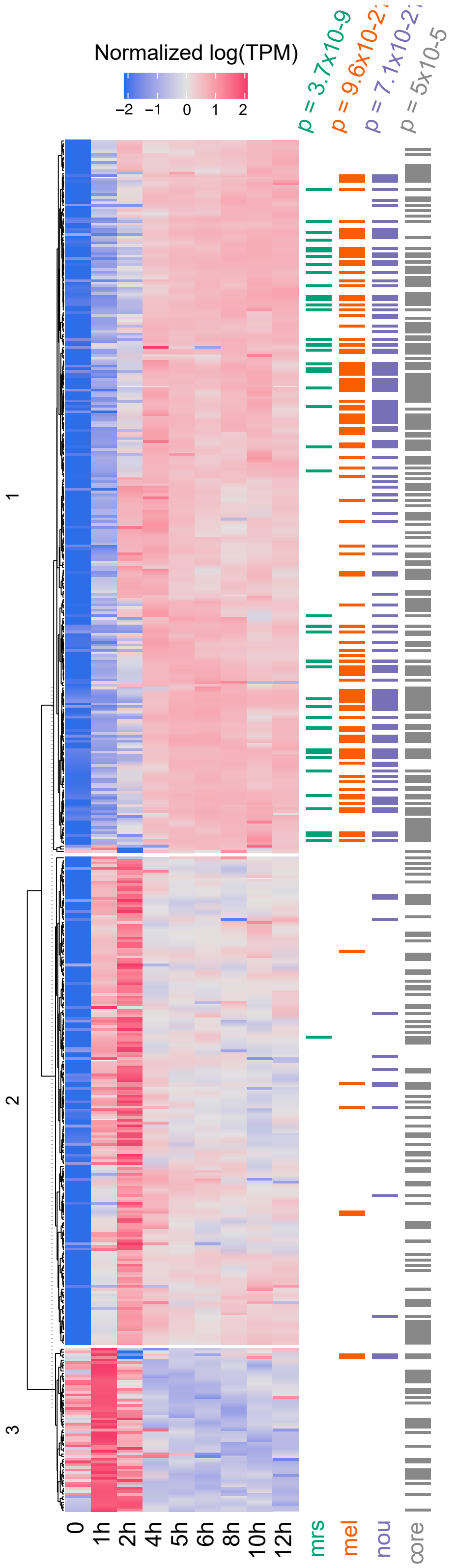

Figure S5. RNA-seq marseillevirus gene expression.
RNA-seq data was generated by [1]. The left part of the figure shows the heatmap of marseillevirus gene expression profiles along a replication cycle. Rows correspond to genes and columns to infection time points in hours (0 h corresponds to 30 min of infection due to virus adsorption period, see [1] for details). Expression data in TPM values were normalized and scaled (see Materials and Methods for details). The clustering was done using a spearman correlation distance and separated in 3 main clusters using kmeans. Genes whose protein products were identified in marseillevirus (mrs in green) [2], melbournevirus (mel in orange) [3] and noumeavirus (nou in blue) [3] are shown at the right of the figure. For the proteomic datasets of noumeavirus and melbournevirus virion particles we displayed the orthologous marseillevirus genes. The gray column represents the core genes. The p-values on the top were calculated using a chi-square test based on the counts among the three clusters.

1. Rodrigues, R.A.L.; Louazani, A.C.; Picorelli, A.; Oliveira, G.P.; Lobo, F.P.; Colson, P.; La Scola, B.; Abrahão, J.S. Analysis of a Marseillevirus Transcriptome Reveals Temporal Gene Expression Profile and Host Transcriptional Shift. *Front Microbiol* **2020**, *11*, 651, doi:10.3389/fmicb.2020.00651.
2. Boyer, M.; Yutin, N.; Pagnier, I.; Barrassi, L.; Fournous, G.; Espinosa, L.; Robert, C.; Azza, S.; Sun, S.; Rossmann, M.G.; et al. Giant Marseillevirus highlights the role of amoebae as a melting pot in emergence of chimeric microorganisms. *Proc. Natl. Acad. Sci. U.S.A.* **2009**, *106*, 21848–21853, doi:10.1073/pnas.0911354106.
3. Fabre, E.; Jeudy, S.; Santini, S.; Legendre, M.; Trauchessec, M.; Couté, Y.; Claverie, J.-M.; Abergel, C. Noumeavirus replication relies on a transient remote control of the host nucleus. *Nat Commun* **2017**, *8*, 15087, doi:10.1038/ncomms15087.

Computer code

Python code used for cumulative GC-skew and AT-skew calculations

```
#!/bin/python

import sys
import numpy as np
import argparse
import os
parser = argparse.ArgumentParser(formatter_class=argparse.ArgumentDefaultsHelpFormatter)

parser.add_argument('-f', action="store", dest="in_path", default= None, help="Genome file
(.fasta) [REQUIRED]")

args = parser.parse_args()

if args.in_path is None:
    print("Genome file (.fasta) [REQUIRED]")
    exit()

fullinputpath = os.path.abspath(args.in_path)
output_prefix = os.path.split(fullinputpath)[1]
output_prefix = os.path.splitext(output_prefix)[0]

'''Opening Fasta'''
fasta = open(fullinputpath, "r")
fasta.readline()                            #get rid of header
sequence = fasta.read().replace("\n","")    #removes \n for counting

'''init'''
compteur_A = 0
compteur_T = 0
compteur_G = 0
compteur_C = 0
boucle_A = 0
boucle_T = 0
boucle_G = 0
boucle_C = 0
GCskew = []
ATskew = []
AGskew = []
ACskew = []
TGskew = []
TCskew = []
calculGC = 0
calculAT = 0
calculAG = 0
calculAC = 0
calculTG = 0
calculTC = 0
pas = 100                                   #step size
window=1000                                 #windowsize of N nucleotides
taille = int((len(sequence)/pas)+1)
cumul=np.empty((taille, 4))


'''Boucle'''
for start in range(0,len(sequence),pas):    #browse the genome
    end=start+window
    if end > len(sequence):
        end = len(sequence)
    for i in range(start,end):              #count in groups of N nucleotides
        if sequence[i]=="A" :
            boucle_A+=1
        if sequence[i]=="T" :
            boucle_T+=1
        if sequence[i]=="G":
```

```
            boucle_G+=1
        if sequence[i]=="C":
            boucle_C+=1
    if boucle_A==0 :
        boucle_A=0.1
    if boucle_T==0 :
        boucle_T=0.1
    if boucle_C==0 :
        boucle_C=0.1
    if boucle_G==0 :
        boucle_G=0.1
    #cumulative GC and ATskews
    calculGC += ((float(boucle_G)-float(boucle_C))/(float(boucle_G)+float(boucle_C)))
    calculAT += ((float(boucle_A)-float(boucle_T))/(float(boucle_A)+float(boucle_T)))
    GCskew.append(calculGC)
    ATskew.append(calculAT)

    boucle_A = 0                           #set to 0
    boucle_T = 0
    boucle_G = 0
    boucle_C = 0


''' Write & Close - Files'''
fasta.close()
np.savetxt("{}_GCskewscore.csv".format(output_prefix), GCskew, delimiter=",",fmt='%f')
np.savetxt("{}_ATskewscore.csv".format(output_prefix), ATskew, delimiter=",",fmt='%f')
print("Done!\n2 files have been generated : XXskewscore.csv\nThanks for using this script")

'''Check the generated files on R'''
```

Dataset S1

Reassembled nucleotide genome sequence of tokyovirus (fasta file)

```
>tky
ATGACTTCTGTTATCTGTGGCACCCGCGTAACTGCTGCTTCCGGTTTTGTGGATTTGGCGACTTTCTCCGATTTGGAGGC
CTACCTCTATGGTGGTTGTTCTGCCGTCACTTATTTTGTGCGTGCCATCAAAAAGGCTAATTGGTTCTCTTTCCTCCCCG
TCGTTCTCCGCAACATCTCGGGTCTTCCCGGTTTCGGCTCAGAGTTTTCCGCCTCTGTGAATCGTTCTGGCGATTACGTC
CTCAACACTTGGCTGCGCGTGCGTCTGCCCCTCATCGCCATCCGCCCGACCAATGCTGGTGGCGCCATCAACGCTAACGC
CACTATCCGATGGACCAGGAACTTCATGCACAACCTTGTCGAGAAGGTCAACATCACTTTCAATGACCTCATCGTCCACG
AGTTTGACAGCTATTGGTTTGACTTCAACTCCCAGTTCAACATCGACGCTTCCAAGCGCGTCGGTTACAGGAACATGATT
GGAGATATTCCCGCCATGATTAATCCCGTGACGACTGGCAACCCCCTCGGAACCGGTGAGTTCTTCAATCTTCCCATTCC
TCTCTTCTACACCGAAGATTCCGGTCTCGCTCTTGCTGTGTCTGCTCTTCCTTTCAACGACATCAAGATCAACTACTGCC
TTCGCAGGTGGCAAGACCTGATTGTCCTCAACGTGGGTGTCGGTGCCAATCCTCCCACGTTTGACGACATTGTCCAGGTT
TCTTACGATGCCGCTTTCACTCTCATCTACAGCTCAAACGCTCCCGCCATCACCAACGTCGAGACTTGGTGCCACTACGC
CGTTGTGCACAATGACGAGCGTGTCAAGATGGGTAAGAATCCCCGTGATATGGTCATCAAGCAGGTGCAAAAGGTCAACG
AGACGACCATCAACCTTTCGCAGCTCAACGCTCTTGTTCCCATCGACATCCGCGTGTCGCATGCCGTTGTCGGTTACTTC
TACGCCATCAGGAACAGCTCCACCCCTGGCGAATGGTCCAATTACACCACCGAGCCCGCCTATGCTGGTCTTGACCCGCT
CGAGGCTGCCCAGCTTGTGTACGAGTCCACCGCCCGTGTCAGCAACGGCTCTGATTATTACAGCCTCATGGTGCCGTGGT
TCTGGCACAAATCCATCCCGGAAGAGACCGGCTACCACGCGTACTCGTACTCTCTCGACACGTTCGCTTCTGACCCCAAG
GGTTCGACCAATTATTCCAAGCTCACCAATGTGTCGAACCAGTATGTCCCTTCAGTCGCTGCTGTCAACGCCTCTGCTGG
TGTGACCAACACCGGCATTCCGATTCCTTCTGCTACCAACCCTGCCGTGCTTCAGCAGAACCAGACCTTCCAGCACATCT
TCCGAGTTTTGAATTTTAACGTATTGCGTTTGTCGGGAGGAAGCTTGGGTCTTCCAGTATTGTAGGATGTCAGTCCTTCA
GTCCCTCGCCCATGCACTTTATCCACAAAAAATATTTTTATGTTTTCACTCAAAACATAACTATTTGCTGAGAGACTCTG
CCCTCTTTTTGTAGGAACATTCCCTGCATCCTTTCCACGCTTTTGTTATCGCGTGCACTCTGACTTCTGCTTCTTTTCCA
CAAGAACAAACATATTTGTCGGAGTTTTGTTATTTTCGTAAGGCCCAATATATTCACAGCCTCTACTCATAAAAAGATC
CCTCACCTTGTCTTCTTTCGAAGAGTTTTGTCCTCTTTTTGTCCTCTTTTTGGGACATCCCCTGTAGTTCTTTTTGAGAA
GGTCCGCGAATAAAACCTTGTGTGTTTTTCCGCAATCACAAGAACAAAGACTTTTTGTCGAAGGGCGGAATGTTTCTTTC
GTTTCGAGAAGTTTCAACTCGTTGGCAGAAAAAGCTTTTTCTGCCAACGAGTATTCTTCCTCCAGAAAAGATATGGATTC
TTCCGCAGAAATGACATCAGACGTTCTGAGAATACAGCTCATGCATAACGGCTTCTTTTCGAATGTTTTTATTTTTGTCG
TTATTTCCCTCCCGCAAAATGCGCAACAGAAGGTTATTCTGTCCCCCTCAACATCAACGATAAGGTCGTCGAAACTCTGC
ATGACTATAATACTAGATTACTTTTATTGTTAAAAGGTAATTTTACTCTGGGAAAATTTTCTTCAGAAGCCTCCCATTTT
CTCCATAAATCCGAATTTCTGTGTCGTATCCCGCCTCTTTGCACGCTCTGATTTTAGCAAAGTTTCTTTCTTTCTGTATG
GTCATTGTCCACTCTGACTTAACCTCAATAATCCTATTTTCTTCCTCGATGTAAATATCAGGAAAGTATCTGCTGTTTGT
TCCTTTGTGCGTGTATGATACGACAGGAATTTTTGTCGGGTCTGTGGTAATTTGTTCTTCTGTATAACCTTCTTTTTCGA
GGAGGTCCCTCAGACAAAAAGGTTCGTACCCTTGACAAATCCAAACATCTCCGGATGGAGTCACAAATTCCTTTTTTCTG
TAGCAACTTTTCTGTATTTTTTTCTGTACCTCCGCGTTTTGTGACGGATTTCTGACTCCATACATCTTCATGCAAGTTTC
TTCTCTTCTGGAATCGGAACACTCGGAACATCTCTTTCCTTTGCAAAATTCTTTGTAGCAGGCGGAAAATATGTTGTTCT
CGAACTCACACGAACAAATTATCTTGAGACGAGAGTATCTGTCCCTGTATTCCGTTTCGCTTGTCGTGAGAACGCAACCT
TCTTGTTTTATAACTTCCTCCACTTTTTTCCACGGTACTCGTTCTCTCTTTTTTGAGCATTCTGCGCAACCCACCCAATT
TTCTCCCATATTCCCAAGACGAACAAATCCCTCTTCTCCGCATGCGCAAACATAGAGGATGTCTTGTTTGTTTCCCTTGT
ACTCACTTTGTTCCAACAAAATCTCACATCCTTTTTCTTGGAACTTGCTGACTATCTCATCCCAAGGTTTTCTTTTCTTC
TCCTTGAGACATTTCATGCAATTACCCTTTCCTTTTGGCGCCTCGGACAAAAAGTTGTTCCATTTGACCGTTGTTTCGTG
TCCTCCCTTGCATATCACTCTCAAAGGAGAAAAGACACCTTGGTATTCTGAAGAAGGACCCAGAAGCTCAAACCCATAAG
ACTCAAAAAATTCTCTTCTTTCCTGATGCTGAATTTCCCTCTCGGACAAATATTCTTCTTTCGTCCTAGGTTTTACCCTT
CTTTTTTGACTTGCGATGCGAGCAGTTGCCATCACGCAATTTCTACAAAATGGCAAAACAAGGAAGTTTCTTCCCTTCTT
TACCGTCTCTTTTTGGCACGTTTCGCAGGCAAACTTCCAAGGCTTCGAAACAAAGAATTCTTGCTGAGACATTTTATCCA
TAAAATATTTAGAGAAATATTTTTAGTTGTCTCATGGAAGTTTCACCTTTCCCATCGCAAGAATCTTCCGCTTCAAGACT
TTGTATGGATGGTTTTTACCATCCAAGTTATAGGGATAACCGTCTTCGTCTTGAATCCAACAGTATTCGACGCCAAATTT
TGGGATAGAACACCACACCTCTTCTCCGATGCCTTCTTCCGCTTCGTATGTCTCGATAAAAATGTCTTTGGGTGAAAGGT
CAAACTTTTTTGTGAGTTTCTCCAAAATGCCTTGAGTTGTGTTCATATTTTTTTGTGAATAAAATATGAAGTTTGTTACT
CTTGTTGCTCTTCGATAACCTCCCAGAGGTTCTTGACGATTGGAGTTTCATAATCCTTGACATGTTCGAGAAGAAGACTT
TTCCAATCTATTTTGTCTTTCCAAACGTCTTCCTGAAGGATACGGAGAACAGAATACCCATTTTTGATAGCTTGTTCTTC
CTTGTATCTATCTGATTTCTGCTGTTCTTCTGGCGACTTCCAATTGGAGACTTGTTCGTAATGCTGCCTTCCGTCGAGTT
CTATAATAGTTTTTGAGACGCAAAAGTCAAAAGGAAGAAAAGTGTTCTTTTCTGGATTCTTACACCACGAAACTTTGAAT
TGATGAATTGGGTCTTCGAAGTGTTCTTCGAAGAAGGGGAAGAGCTTCGTCTCGGTTTTATTTTTACAAACAGGGCACCA
ATAACCGTACGAAACATTCGAAAGTGCGGAAGAAAATTCGTGACGTTTTTCACAACGAAAGAAATATTTTTTGGCTGAGT
TTTTGAAGACTTCTCTTGGACTTTTTTTGTTTTTGGCGCACCAAAAAACTATTTTTTGGTGCGAAGCGAAGCTGTTTCCA
AAACAGCTCCCACAGTCATAAAAAGAACAGAGTTTTTGTTTTGAACAAAAAGGGCAAAATTTTCCTTTGGAGACATCGTA
AAGCCTCATCTCGAAACTATGTTTGCATTTTCGACACTCAAACCAAAACTTTTTATTTGAATTCTCGAACACCTCCCTCG
GACTCTGTTTATTCTTTTCGACGCTCCAAAATTTGGCCTTGTCGGAAGAAGCGAAACTCTTTTCAAAGCATGTTTTGCAT
TCATCCGAAGAGCAAAGTTTTTTACTAGAACAGAAGGGGCAAAAAGTCCCTTTCGAGACATGGCTTAAACTCGCTTCGAA
ACTATGTTTGCACTTTTCGCATTCGAACCAAAACTTTTTATTTGAATTTGCGCGCACTTCCCTCGCTCTTTTTTTTGTTCT
TGTCGACACACCAAAATGCGGAGTTTTTGTGGGACGCGAAGCTTTTTTTAAAACATATGTCGCACTCATCGGAGCAACAA
AGTTTCCAATTACTACAATATGGACAGAATTGGCCAGAGCAAGAAATATTGCCCAGGTTGCTTTCAAAAGAGTGCCCGCA
TTCCCCACAAATAAAAGAAAATTTTTTGTGACTATTCTTCGCAATCAACAAAGGATTCTCCTGCCCTTCAGCCAAATATT
TTGCTTTGTCGCACGAGGCAAAGCTTCGTTCAAAACAAGGCTTGCATTCCTCCCTTCCACAAAGTTTTCCCCTTGTCCGA
GTTTCGCAGTTCATGGTTTCTTCTCTAAATTTCAAGACCAAGTTCCTTCCATTTTCCAAAAATATAAAAAGTTTTTATAT
TTTATTCCGAACACAGCCCTCTCAAAAATTTCCGAAACTTCTCCGGGTTCGCTCCGTGTCTCATCAAAAGTTTTGCACAT
TCCTTGTCTTCGCGCATCGCTGCCGTGCAAAGAGCCGAAGTCAAATATTTTCCTTTTGGTATCTTTTCCAAAAGTTTTCC
```

CAGAGATTCAACATCCCCTTCTCGAACGACACGATGAGAAGATACCCCTTTGTCATAGTCAGGGTTTACAATCTCAATGT
ACTCTACAGCTTCCTCTCTGTTGCCATGAAGATATTCTGACACGAATTTTTCCTCTGGATAAGAATGACCGTCTAACCAC
GAAATGTAAGAAGACAAGAATTTTCTGGGCATTTTACGGAATAAATGCCCACGGTTCTTTCTTTTTCGATGTGCTAAAAA
TCGCACGCATTCGGATCTCTCCGAATGAAATTATAAACATGAATCCTGAAAACCTTCAGGTTACCTGCAAAAATTGTGGT
GAAACAAAGCACCATTCCGAATGTTCCATAACCTTCCAAACTTGCGGAGACTGTATGAGCAAAACAAAATGACCGGGATT
GAGCCTTGAAGAGAGGATGGAATTAAAAAGGATGGTGTCGAGGTTGAGAATACCTGAAGTTTTCCCTTCCCCCTTTGTTC
AGGGGCCACGTACCTCGTCAACAGCACATCTAAAAAATACCGCGTTTTTTCTTTCCACACAAAGAAAAAAGTTTTATAAA
ACTGAACAAAAATTATGGGACTTTCATCCTTTGAAAAATGTTCAACATGTCCGACAAGAAAAAGGCAAAGAAAGAGAGGC
GCAAGATGGCGAAAAAAATTGGCTTTGGTTTGACAGCTCTCGGAACTGCGGGTTTGGTTTTCTCTCTCCTCGCGAGAGAG
AAAAAGGTTGACAAATTATCTCTAGATGTCGCCAAAAGGGTGCTAAAGTCCCCCAAGGCAGATTCTATGTACCTTGTCCA
CGCCCTCGCGAGAGTTCCCGATCCGCCGTCGTACGTTGGAACTTTTGTTCTATTCCCTGTCGGAGGAGGACCAGATTATG
ACCAAAGGGAAGGAAATCTTGCGATACTCCAAACAGGGAAGACCTTCAGCACAAAGTATAAACCCCTCCTTCTTCAAACG
GACAATCCAAAATACGTGACTTGGAAGAACGAACACCCTCTCGATAAAATCCCTGATGAGCTCGAGAAGGAATTTCCAGG
ACGTAAGAGGTTCGAAGTCAAGAAGAACACCGAGCCAGTATACTTTTGCCCAAAGAACCTCCTCGCATCTGACAATCCCA
ACGTTCTCGCGAGACGCATTGCAAACAACGAAATGGCTTATGACAAGGTTGCGCTAGCGAGTTGCGCCTCTTCGGTCGGT
GTGGGATTGCTTCTCCTGTTTGCTTTCTAAACATTTTGCAATAAAATGTTTCATCTTATATTGAACGAGCAGAATCTCAT
GTTTCGACCGTAACAATAAAATATTTTACCTCACAGACAAAACCACATTCTTGTCGCTAAAATTCCTTCTTATTTTTACC
CACAAAATGTTCCATACAATCACCGTAAAGTGCGAAGGAAGTGAACACAAGTTCACAGGAAACGACGAGTTCGACACCCA
GTCCAAAGCAGAGGCTTTCCTCCTCAAAAAAGCAAAGGCTATGTCCCGTGACTTGGAAGAGTTTGGCGAGTACATCAAAC
TCGTCATTCTTTCCAAAGGCGGTGTCTCACCAGAAGTGAAATGCTATGAACACCTCGACGGAACAGACATCACTCCCAAA
CTTTTGAGGACTGGGAGTTTGTTCTCGTTCCAGATGACCTATCCATCTGGTTTGACCCTTGACAGTCAATATTCCTACAT
GGCGTTCGAAGAATTTGGAAGGGCTCTCGACGAAATTTACGGTTCTTCGGAAGAGTGCATGAACAGCTCAGAGGACCTTC
TCGCAGATTCTCGTCTCTTTGACAAGATTTTTCCTTCTGAGACCTTCCCAGAAAGCATAAGAGAAAAGATAAGGTGTCTC
CTCGAAAAGCTCTCGGACCTGTCCTTGGAACATCAGGACATTCACGCCGGAAACATTCTGATGGACGAACAAGAAACTCT
CAAACTCATCGACTTTGAGTACGTAGAGTTTTTGTGAAGTTGTTTTTATTCTTTGATAAAAACAAAAAAAGAGCTTTTGT
GGTTCTTTCATAGGAAAGACTTCATTTTAAGATAACGGACGAACAAAGAGCAAACGTCTTTTTATTTTGTTTCAAAATGA
CGAGCAAACTCCTCCCTCACATTTACGCTCTTCTTCGTGATGAGTTCCTTCTCGAAAAAGACGAAGTCGTTGTTTCTTTG
AAGGAGTATACAATGACCCTTAAAATACCGCCTCACGAAGGAAAGCCGACCATCAGCGTTTCCGTACGCTTTGCGGAAGA
ACGAATATGCAGGTGGTACGAACGGAAATCTCTCGGAAAAACATACTTTGGGAGCGGAGGGTTTTTTGAAGTACCTTCTG
TAGCAAAAGGAGTTGACTTTGTGAGATCCCAGTGCGCAGACAACATCGCCGTTCGCGAATCTCTGGTCCAAAAGCACAAG
AAATTCTTTGAAGAGAAGGAAGAACTTCTCGCAAAGATTAAACGTCTCAAGCAGAAGAATAGAGAGTTCAAGTACGTTCC
GGGAAACGCAGGCGCCCTCAAAGCGCAACAACACTTTGAATCACTGACAGAATAAATTATATATTTTTGGAATATATAAA
CCACAACTTCAAAAGATATCTTTTCGAGAAGACTCTTTTCTTGAGCGTCGCGTTTTTCTTTCAAGGGAAAGAGGCTTCTG
TATCGCAAGAGTTTTTCTTTGCGTCTAAATATTTCTAGAGAAATATTTTCTACCAAACAAATTCTTCTGCATTTGCCTCT
ACGTCACCGGCCTGACATTTTCCCCTCATGCAAAAAAGTTCCTTTACGTTCCCTTCTTCGTCGTACCTGGCAAACTTTCG
AATTTTCCCCGTTTGAAGTTCTTTCATGACAACTCTTCCACTCCTTTCGTAGACGACCTTGTTTTCTCTCGCTGTCACGA
CAATACCGTCTGGACGGTACTCCTTTGTCAGAACAATCTTTCCCTTCTTCCACCTCTGCTCGTGCCAGAGACGTCCATCG
ATTGTCCACGAAAATCCCTTTCTCTTCTCCCCGTGTCTATAGAAGAGTTCTTGTTTTCCTCTCTTGTTGGCGCTCTTTGA
GAGTTTGGTCAAAAAGAGATAGTTTTCGTGATGAGAAAACTTCCTTCCATCATCTTGCCTTTCGAGAGTTTCTTTTGTGG
TGTCCCTTCTCCATCGCTTCAGCAATCTCTTCCTGAAGCTTTTGGAAAAATAGGACGTCCTTTTCTTTTGGGTCTTAGGC
AAAGCAGCCATGTTTTTTGCCTTTCTCGTCAAAACCTTTGGACAGCATCTTTTCAAAGTAGAATATTTTAAACAAAATAT
TCTCTGTCGTTCGGGATTTCTTTTCCCAATCTCATCCACAACTCGAGGAGAGAAATAATCCTTCTCTCGTGCCGCATTGG
CAGTTTCTTCCGACACAGAAAAATCTCTGTTTTTCAAGAGCCAAACGGCAAGTCCTTTGGTCTTTGACCATGTGCACTCC
AAGAGGATTCGCTTGTCTTTTGGACATTTGCTCTCGTTCCATTCCAAAATTTCAGATTCTCCACCGGAAAAATCTCTTCT
TTGGCGGAACGTTTTTACATTTCTGACATCTCAATGGGCTCCATCATCAAAGTCGAAGCAGAGGGGAAGATTCGCATCTT
CCGCGGAAAGGACGATGTCGACGCGCAGTGCAAACTGGACGCGTGGGTGGAACGTAAAGAGTTTGCTCTGCGTCCCGAAG
AAAGGTGCGGAGAATATCTCAAAATTCGCAGAATGTACGGAGAAGAATGCGCTCCACTCGAGGTAGAATTTTACCAACAT
CTGGAAGGGACAGGCCTGACTCCGAAGCTCTTGAGATACGGAGATTTTTTCTCCTTTAGCGTCACATATGCGGAAACAGG
GTTGGTGTTGTCCAACAAGTTTTCATACATCGCGGTCGAAAGATTTGGAAAGTCTCTCGAAGAGATTTACGGTTCTTCTC
CCGAATGTATGAACTCGTCTGAAACGATGAGAAAAAACAAGCTTGTTTTCGACAGAACGTTCCCTTCAAAGAAATTTCCG
GAAAACGTGAGAGAAAGTGTCAGAAAACTCATCGAGCGCCTTTCGAAGGCTTGCCTGGACCATCAGGATTTTCACGCAGG
GAACGTTCTGACGAACGAGCGAGGACAACTAAAGTGTATCGACTTTGAGTGCGCTGCCATCACAACAACAGAATTTTGAA
GAGCGGGCCATTTTTCTCATCCCATATTTTTTGTGGATGAAAACAGCAAAAATATTCATAAAGAGAATATTTTATTCTTG
GTGTGTTCACGGTTTCCTCGAATGTGCAGTCCTCGGCATGTCCAAACAAAATGTTCTTCCGTTCTGTCTTGTCGTTCCAA
ACATCTTTCTGAAAAATCTCTCTTCCCTTGGAGCATTTCCGACTTTTTCTTTCACTCCAAAACATGCAAGCCACAAAGCA
GTTGTTACCCCTTTCCGAAGATGGCAGAGTCCGCCACCAAAAAGTGAAGAGATGGCAGGAATGCGACTTTGTGGGAAAAC
TCTGCCATCACAAAAAGATTGTAAGATATTGCGACGGAGCAAAGCACGGAAACACAGACACCTTCATCAACGGAAGGATA
AATGTTTCGACTCCATACGCTGACGGAAAGAGACATGGAAAACAAAGGATTTGGTTCAAGAACGGAATGCTTGCATCTTT
CACTCACTACGTCGAAGGAAGAGAACACGGCATCCACAAAACTTTTCACGGTGACGGAAGAACAGCCTGGGAGTTGAGGT
ATGTCTGCGGAAAAGTCTTTGACGAAGAGACTTTCTGGACCTTTGGAGACGGAAAGAAGAACAAGAAGCAAAGTGAGAGA
TAAATATTTTAAAACAAAATATTTTAAGAAAGATGCCTTTCTGTGTTTCTTGTGGAAGTTGGGTCCAGAATCCAAAAGTG
TCAAACGGACAGGTGTTTTGTGGGAAAGACTGCACCACAAAAGGAAATTTTTGCAGACACACAGGACTTCCGAAGCGCTC
CTACTTGGTGAGACAGCGTTCTCTCGACTAAATATTTTCAAAGATAAAAATATTTTACTAAATGTTTGCTGTTTGGCTTG
GCACTCCCACAAAGAACGGAATGCAATTCGAGAAGCCCAACATCCGCCAGGTGATGTCATGGTTTCGTTCTGGGGGAACT
CTTTCATTCAAAGGATTGGACAACTGTTTCGTCTCTTATCGTGGAGAAAACACTCAAGGAGAAATTCTCGATGTCTGCGA
ACGCGTTCCTCCAAATAAATTTGAAGAACTCAAGCTCGTTCCGACCGTGTCTGACATTCGAGGTTTTGCTGTTTTCTTCC
CAGAGAGTTATAAAATTCTTCTTTCATCATAAAAATATTTTTATATATTTCATTCGTCAAGGGGTCAAACACCTTATTCC
TTTGGCTGCACAGCGCGCGCGGAACATCCTTTCGTTTATCGAAGAAACTTTTGTCCTTCCGTTCTCTAAAATAAGCATGA
AACTCTGCCTGTTGTCAGATTGCCATTTGGAAATTTCCCCACAAAAGCTCCAAAAGTTTCTCAAAGGCAAACTTCTGGTT
CCGGACAAGAGCAAAGTCCTTTGTCTTTGCGGAGACATTGGAAATCCTTGGTCGAAAAACTATAGCAAGTTCCTTGCGTG
GTGTTCAGAGTCGTTTGAGTTTGTATTCGTGGTTGCAGGAAACCACGAATACTATGGAGAGCACACGATAGAAGAGACGG

ATGAAAGACTTTGGGAGCTGAGCAAAAGCCATTCGAACATCAAATTCCTCCAAAACTCTTCGTTCGACTACAAGGGCGTT
CTCTTTGTGGGAGCGACGCTTTGGTCAAACGTTCCGGACGACGCTGACGAACTTATGAACGACTATCTCGAAATTCCTGA
AATTTCCCCAGAGCTTATTCGGCAAAAGTGGAACAACTCCGCGTCTTTCATTCTCAGGGAAGTGGCAAAGGGGAAGAAGA
CCGTTGTTCTTACACATCATTGCCCTCTCGACGAAGACGGAGCCCTTGGAACTAAAAGCGAGTATCGAGAGTGCTATTGC
TCCAACCTCGGATTTTTGGCGAAAGAGAACGTCTCTGCTTGGTTCTGGGGGCACACACACGTTCCGTTTCACGAAAAAAG
AGGAAGTACAGTCTTTGCCAGCAACCCGAAAGGATACACGAGAGAGGAAGTGGGGTGGAAGCAAAATTTCTTTGTGGATA
TCTAAATATTTTTAGTCAAAAAATATGCAAAGCTTTTTGAGCAAGAGAGAAACCTTGATGTTCTACCTCTCTGGAGAGA
GAATCCCTGGAAACATTGAGGAGAAATTTCTCATAAAAAGGCAAGCGTTCACCTGCACACAACCTCCAGATTTTCGCATT
TGGTTTGTATTGCCTGATGGGAGACTTCATGGAGAAGAAAGAGATTCGTCTGGACTTGTTAGAATCTGGAACAAAGGGGT
CATTTCGGTCTTCTCCAGTTGACGACCCAGCTCTTTCGAGTCTTTTCGTATCTCTTTGCGAGCTCTGGATTTTCCTGAAT
CATCTTTTTCGTATCCACAGATTTCGTGGTTCTCTCTGACCTAGAAAGAAATATTTTTCCCTCGTGGGTCAGAGAATTCT
TCCCTTTTCTACCCATCTCAAGAAACAGTTCTTTCTTGAGGCTGTCGACCTCTGCCTTCTTTTTGCGAATCTCTTGTTCG
TATAGGGCGATAAAATCTTGGAGTTCGTGGATCTTTTTGACGCTTTCCATCTTATCGAAAAGAGAGGTTTGGCCGTATTT
TCTTTTGTGGGAAATGGGAATTCAAGACCTGAATAAACTTTTGAAGGAAGAGTCGCCTGCTTGTTTCAGCAGGCATCCGA
TGTCCACGTTTTCTGGAAAAAAGGTTGGCATCGATGCGTTCCAATGGATATTCCGTTTTTTGAGAGCTTCCGGAGATGAA
GGGGTCCTTCCTTCGTTTCTAAGTCACATTTGCGCTCTTCGCAAAAATGGTGTTCTCGTGTTTTGCGTTTTTGATGGAGC
AGAGTGCCCAGAAGAAAAGGAAGAGGAAAGAAAGGACAGGAAGAAGAAAAGAGACGAACTTGTCGAGAAGAAGAAAAATC
TTGAGAGGATTTTTGAAGAATTTGACGACGACTGCGATGTTGTCCCGAAAGACGTCCAGGAAGACGCCAGGAAATGGCTG
CACAAAAAGAAATACGCAGGTGCTGACCTTTCTGACCCTTCTGTTTTTTCGAGGACAGTTCTCGAGTCTTTGACAACGTA
CACAAAACAGTCTGTTCCCCTCACGAACGAAAGGGTGGAATCTGTGAAGCAACTCATGGACGTTCTTGGAATTCGCTATG
TTTGTGCAAAGGGAGAAGCCGATGGAGTTCTTTCGTCGTACGTCGCGAAAGGGAAGCTTTTCGCTGCTTGTTCGGCAGAC
ACCGATGTTCACGCTTACGGAGTGCCTTTTGCGATACCAGAGATCGACACCTCATCAGAGGCTCTTGTCCTTGTTCATAG
AGAGCAGTATCTCGAGGACCTCGGACTGTCAGAGGAACAGTTTTTGGACCTTTGCATCATGTGCGGCTGTGACTACAACG
ACAGAGCAAGTCAAGAAGCGAAAACTGCCGGGAAGAAGGCAAGAGGCATCGGTTGGAAAACCGCACTTTCTTTGATTCGG
AAGCACGGCTCTTTGGAAGAGATTGAGAAAGTTCCTGGAGTCAATGTGGAGCCGCTCAACTACAAAAGATGTAGAGAGCT
TTTGAAGACACAAGAGACAATGGATGAAATTCCCCCAGGAGGAAAGCCAAACAGAGAAGACTTGGAAAATTTCTTCCAAG
AGAACAACGTGCGCTTCGACGTCGACTATGTGATGGATTGTTGGTGCCCTATCGACATCTAAAAAATATTTTGTTTGAAC
AAAATATTTTGATGGAAGAGTTTTTGAACAAGAGAGAAGCTCTTGTTTTTGCTGTCGCGACATTGTGTTCTCCAAAGAAG
GAGAGGTTTCTCAAGTTCCATTCGAGCAAACATAGAGAGTTTTGGCTTCTTCCAAACAAGAAGAAGCACGGACCAGAAAA
GATTTTTTGTGGAGAAGTTGTCACATCAGAAAGGACTTGGAAGAACGGACTTCTGCATGGGGAAGAGACCCTATACACAG
AAAAAGGTAGAATGTTTGGCATGAGAACTTGGAGGGACGGAATCAGACATGGAAAAACTCTCCGCATCGAGAAGGCGTAC
ACATCCGAAAAATGGTGGGAAAACGGAGAGCTTGTGTATGACAAGTGCACATCTGATGATTTGCTCCACATTTTTTTGGC
TGGAACGACAACAACGTACAGAAAGGACGGAACAATCTCGAGAATCTATTCAAGAAGCTTTGGAGGCATCGCTTGGAATT
TTTCAGAGGAAGGGAGCGTCAGAAGGCGTCTTTCCAACCATCTGTGACAATATTTTTTATGGATAAAAAATATTCAAAGC
TTTGTGAGAAGGAGTGAGAAATTTGCCTTGTTGAGCTTGTTGTCCACCTTTTTCTTTGCCCATTTCGTCATCTCTTCGTA
CTTTTCGAGAGAAGTGTCTCCGTCTTTGTCTTTGAGCCAGATGCACGTCATCAGCAAGAACCAGAGCTGTTTTTTGTTGT
ACATCTCCATGTTCTGACACTGGAACATCAGAACCTCTTCCACCGTCTCGCGAATGTCTTCGATGTCTGTTGTCTCATCA
ATGAACTCTTGAATTTTTCTTTGTGAGAGTACATCGTCTTTTGGACCCTTCTTGCAAAGATAGCACACACGAAGGAACAA
AAGGATGTGTTCTTTTCGCTTGTTTGAACGAAGAGACTCGATGTCGTCTCCATAGATTGTTTGCAGTGTGTTTACACAAT
ATTTCACAATAGGGCTTTGATGAGCCAGAAGTTTTTCTGTGTACTTTAGCTGCATGCCGTCCTGAATTCTCTGGAACATT
TTCATCTCTGTGGTTTCTTCCAAGCCATAGAACGTTACAAACCTCATGGGCGACCTCGAGTTGAACCACCTTCTTTGCTC
TTCGGTGAGAACAAAAGAATTTCCTGTTTTCTTTGGACAAGAAGAGTACCAGATTTCTCCTTCCTTTCTTTTGTAGCTTA
TTTCGTTGGACATGAATCTTTGGACAGACAAAAGGCGTTGCTTGCCGTCCACACAAAAAAACACGTGGTCCCTGTACGAG
AACAAAAGCGCTGGGATGTACATTTCAGAAAAAATCGTGTCTATCAACATGCTCTGTTTTGCCGAAGACCACACCATGTC
TCTCTGGTAGCTGGCATCGAGACTGATTTCCCCATCAGAAAGCTTGGAAAAAACACTATTGAGGTCCGGGTCAGAACTCT
GCGTTTGGCGCTTATTTGTGCCGACATGAAAAAAATCTGAAGTCTTCATGTTTATTTTCTTTGGAAACTAAACTCTAAGT
CAAAGGTTCCAATCGAAGGTTCAAAAGTTCAAAAGTAACTTCTTTTCGTTATCGTTTGAACAAGTCAAAAACAATCTCAA
ACAATATGTCTTCTGTGAACGCTTCTTTCGTTGAGTTTCTTTCCAAGTTTTTCCTTGACTCTGATTTCTCGGAGAAGCTT
GTCGAGTATCTCGCTGAACAGCTCGACGCTGAAACCTCTGTTGTTGAGACCGCACTTTCTGGCTTTTTCGAAGAGTATGA
GATGGGAAAGTCGACCAAGAAGGTTGTCAGCAAGCCCGCGTCTCGTTCTTCTTCCTCCTCGTCCAAGGCCTCTTCGTCTT
CTTCCAAGCTCGACATGAAGCTGATTGAGAAGAATGTGAAGATTGCCAAGACCAAGACGAAGGAGGAAGACAAGAAGCAC
TACTTCAACGTCAGCACCAGTCGTCTCGGCACCAAGTCCACCAAGACTGCCAAGATGTACACGTTCGACGACAAGCTGTA
CATCAGCGGAAAGAAGGATTGCCCCAAGTTCTTGGCTGCGAAGAAGGCTCTTTCTGGTTCCAACGACGAAGAATCTGACG
CCGAGTCCGAGTCTGAAGAGGAAGTTGCCACTGACGAGGATGAGGAGCTTTCCAGCAAGAAGAAGCCGACCAAGAAGCCT
CTGTCTCGCACATCTTCCAAGACTGTTCTGAAGGAGGTTCTCAACACCTCCGACGAAGAGGATGAGGAAGAAGAAAAGCC
CAAGAAGAAGCCCGCTGCCAAGACAGAGAAGAAGCCGACCAAAAAGGCGGATGTGAAGAAGCATTCTGCCAAGAAGTCAA
AGGCTACTTCTGATGAGGAAGAGGAAGAAGAGACCAAGCCCGCCAAGAAGCCTCTCGTCAAAACCTCTTCTTCTCGCAAG
GTNNNNNNNNNNGATGAATCTTCCGAGTCGGAGACTGACAATTCTGAGGAAGAGACCAAGCCCGCCAAGAAGCCTCTCGT
CAAAACCTCTTCTTCTCGCAAGGTTGTCCCTGCCAAGAAGAACAAGAAGGATGAATCTTCCGAGTCGGAGACTGACAATT
CTGAGGAAGAGACCAAGCCCGCCAAGAAGCCTCTCGTCAAAACCTCTTCTTCTCGCAAGGTGAACGAAGAGACAAAGGGA
AAGGCCAAGGTGGAGAAGGTCGCCTCGAAGAAAAAGGCAGAGACTTCTGATGAGGAAGATGAGGAAGAGACCAAGCCCGC
CAAGGGAAAGGCCAAGGAAGAACCTGTTGTCGAAGAGAAGAAGGTGCCTGCCAAGGTTGAGAAGAAGCCTTCCACCAAGG
GAAAGGCCAAGGAAGAATCTGCTGATGAGGCAGAGGTAAAGAAGCCGACCGCCAAGAAGTCAAAGGTCGAAGAAACCAAG
ACTTCCAAGAAGGAGAAGGAAGTTGTCGAAGAAAAGGTAAAGGCGCTTGCCTTTGACCAGAAGAACGCCAGCGACCTTTC
TTACAACGAAGAGACTGGTCTTGTTCTCCATCCTGAGAATGAAGGAGCTGTCTTTGCGATGATGGATGGCGACGACATGG
TCGAATTGGACGATGCTGCCGCTGAAGATGCCAAAAAGCATGGTCTTATCGTCCACAAGGAGAAGAACTTTGCCAAGTAT
GTAGCGTCTCTCGCCAAGAAGGAAGCTTCCAAGGAAGAGGGAAGCGATGAGGAGGTCGAATTCTAAGTTTTTTATATTTC
TTTTCACCAAGAAATATCACACAGTTCCCATTTTTTTGTTCCAATCTTGTCGTTTACAAATCGCAGAGAGTTACAATAAC
TTTTGGTCAAGTGAAAGAACAATGGCTGACTTTAACAAACTCGTGAAACTCGCAAAGGATGAGTCGAACAAGGAACTCGT
AAAGTTTTCAGAGTTTTTGGTGGAGAAGTTGTCCCTTAACAAAGAAGATGTCGAAGCTGCGCTGAAAGACTATGGAAATG
GTGTTGTTCTGAGGAAGCCTGCAAGGACCAGAAAGCCTGCCGCTTCCAAAGAAGACGCGCCAAAGGTGCAATGTTCCATG

TTTATTCCGAAAACTGGAGCTCAGTGCAAGTACAAAAGTTCCGCCGAGGTCAATGGAAAGATGTATTGCACTTCTCATGC
AAAGAAGCTGGCAAGTTCTGACGCAAACACAGCTCTCGCCGTAAAGGGGGCAAAGAAGAAAAACGCGAGTCTCGATGAAG
AGACGACAGAGAAAAAGAACTCAAAGCTTGACGAGTACCTCAAGAAGATGTCGTTGAACAAGTTGCAAAAGAGCGAAAAG
ACGGGCAACTATGTCCACAAAGAGCACGGTTTCGCTTTCGACCCAGAGGACAAAAAGACTGTGATCGGCTTGGAGACGAA
AAACGGCAAACTTGTGGCTCTCACAGAGGCTCAGAAAGCGACTTGTTTGGAGATGGGCTGGGAAGTGAACGACGGAAACC
TTCCACTTCCAGAAGATATGGATGATGTTGACCAGGAATCCGAATCAGAGAGTGAAGAGGAAGAACCTCTCGATGCAGGA
GACGACCTCGACATTGGGGATGAAAGAACCTAGATATTTTTGGCTTTGCAAAAATATTCAAAGATTAATGAGCAGCTACT
CTGAATTTCGTCCTTTGACAGATACAAACATGCTGAACTGGATTCTTCTCTTGTTCATCGTGGGCATGATTGTTTGGCTT
GTGTTTTTCCGAAAACCTTCAGCTGTTCATATCGAACATAAGGAGGGATACGATGGGCAGGAAGAGTTTGTTTTGACAGA
ACTCAAAAAGGCGATGCTCCTTCCAGACAAGAGCCATCTGGAAATGACAGAGGCCGAATTCCAAGATGCGGCGAATAACA
AAAGAATTCAGATGGCAGCAGTGTCGGATGCTGGAGTTGAAAGTGTAGATGGAAAGAAAAGGGAAAAATATAGTTTTTAC
TTCCCTGTGGGAACTTCCGGATACCCCCTGAACCTTTATTCGAGTTTATACGATTATACCCCAAGAAGTTTGTCGTTCGC
AGGATGGAGATTCCTCAAAACCGACTCTCCCGTTTGGTTGCCCGGAAGGTGGAGGCTCTTCAACGGAAAGTGGTGGTTCG
TTATGCCCTGAGAAAACAAAAGAATATATTTGGTGGATAAAATATATTAGAAATTGTCTTTTCAGAATCCGGAGAGAATC
TAAAGGAAAAGTCGCCGAATAAACTATTATTTTCTGGATAAAATAATGAACAGACTCCTCATCTTTGCCTTTCTTGTTAT
TTTTGCTCTCTTGGTCGTTTGGCTTCTCTTCTTCCGCACTCCAACAAAGCATGTCCCTCCAGTCAAAGAGAAAGAGTCTC
TGATGCTTGATATAGTCTCCTGCGGTTGTCCCGATCTTGCCACTTCGCACCCAGGAAGGATAAAACACAAAATGACATGC
TCTGTCTTTCGTTCTTTAAATCCAGGTCTCACCGTGAAGCAGTTTCTAGAACACGAAGGATTCCCAACAGACAAGGATTC
CGAATGGCCAAAGCTCCCATAAAAAAATCCCATTTCTTCCAACGAAAATATCTCAAAAATTTTACATAATGGCTTGTGTC
GCGGAGCCGCTTTCTCTCGCTCACCTCTCTGCTTTTGTCGTCGTCGAGGAGAATGTCGACTGGACGGGTAGAGTCGACGA
ACTATCGAGAGAAACCATCGAAAAGGTCAAAACGAAGATTTCCGTTCATGGAATACACTACAGAAGAGGCAAGGTCGGGC
CGTACATTGCTCAGCTGGCAGAAATCATCGACAAGTTTCATAGGGTGTACCCTCTCGTCCAACATAGAGGACGCTTCGGA
GCGTGTTTCAGCTCCAAATTTCTCAAAGCTCTTCCAACACCAGACACTGTCCAGAGAATGACAGAAACCACCAAAGACCA
ACTTTTTGAGATTCTCCTCTTCTGGGAGTTTTCCACGCCAGAGCTCATTCCGGACGAATTCTTTTCTGCTCTGTGCGAAT
AAAATATTTTGAACTGAAAAAATATTTCTTTGTTGTTCCTTCAAAACAAATGGACAGATACACTGAAGTTCTCGCGGAGA
AGGGATGCGTTCTTTTGGGAGAGTTTAAGGGAGAGGAAGAAAGATTTGCCTTTCGTTGCAAGTGCGGAAAGGAGCCGTGC
TTTGCCTATCCAAAGCACGTTGTGAGAAAATCCTGGGTGGGTTGTTCCACTTGTATCAAGAAAGCGCCAAAACAGAGAAT
TGTTGACTTTTATCAGGCGCAGAGGAGAGCTCGAGAGCAACAACAAAACATTCACGAACATGCGCAAGAATAAACCTCTT
GTCAAATTCATCTCCGATGTTCTGTCCATCATCGACACATGAAACGTCAAATAAAGACTCTCTTTTTCTGGCTGATTGTG
TCCATAAAAGAGTTTCCAAAGAATTTGTTTGACGTTCTCTGTTCGTCAAACAGAGAACCGACTAAAAATATGTTTTGTTT
TAAAACATAAAAATGTTGGAACAAGAGTCCACAAAGTTCACAGACGACGTCACTCGAGGTCTCGGCGTCGAATGGCAGTT
CACGAGGCGAATCGCGGAAAAATACATCAACATCTCACATCTTCCCAGAGAAGAGAATTTTTGGGTGAGGATATCACTCG
AAGAAGATGGGACTGTTTTTTTCAGCGGAAAACTCGCCAACTTGACAACATTTTCTCCAACAGAAAGGGAACTATGCGTC
AAGCTTGTCAGGAGAGAGATTGTTCTGTCTGAAACCTACCAGCTGATGAAGGTTCGAGAGCAGCTGGAAGAGATGAACAA
AAACTTTTCGAGACTTTTAAATCTCCTTTCCAACGGCATCGAATATTCTCCCAACAATGAGAAAAAGATGGAAGAACTCG
AAGAACACTTTGTTGGACTATCGAGAGAACAATAATAAAATATATTTTGCAAAATATATTGTCATGTCCCTCTGCGTTGC
AGAGCCTTTCAGTCTTACTTTGCTTGCTGCTTTTGCGGTCGTCGAACATTCTTTGGATCGAGAAGATGTGATTCTTGATG
AGCTGTCAAAGGAAGCGATAAAAATTGCAAGGTTCAACTTTTCGTCCCCTCACGTACTGTACCGTTTGGGGAAGAGCAGA
GAATACATCACAAAGTACGAGCTCTTTGTGGTGGACATTGAAAAATTGTACCAAGACATAAGAGGTCACAAATACGAAGA
AGAACTTTCTCTTGGTGTTCTGAACGGACTCCCCACGTTGGACGTGGTATCGAAAAGTTCGCAAGAAACAAAGGATGCTC
TCTTTGGACTTTTTCTTTTCTGGGATAAACTTCCCATTGAAAAAGTTTCGGAATCTCTTTTTGATGTTTTGTGCGAATAA
ATATCTTTTGAAAGGAAAAGATATGGAGTTTCTGGCGTCTCCAGAAAAAGAGATGCTCGCCATCGCCGATCCGGAGGTGA
TAGGTAGACTTCCAAAATGCTGGGACGAACTTCCCAGCATTTTGAGGAAAAAAGTCTTTGATGGTTGTCCAGAAAGGGAC
TGGTCTCTGCCGTATTTCTCTTGCAGTTCGTGCAACATAAAAACCAATGTTTCGCTAAAGGGCGAAAAAGTAGAATATTA
CATGTTAGTGATGTATTTCGACATGTTCATATCAGCAGAGACCAAAACTCTCTGGTCTCTCGAAACTTTCACCAAAGAGC
TCTCCAAAATTCGAGAGTACGTCGGAGATTACGAAGAGGAGCTGACAAAGTTTGACGGGTGGTACCAAAAAATATTTTAG
CCATATTTTTTAAGAAAAGCCTGGACAATGTCAGGCTCTTGGTCGAGAGGAAGTTCTCTCGGTTGTTCAAGAGTTCCGAG
AAGTCTCTCCACGAAATTTTGAAGATCGTGAAGTTCTCTTTTTTTGTCTTGCAACTTTCTCTTCAGATTTTTGTTCTCTT
TCTCAAGGATGTCGACTCTCTGCCTTCCAAGAAAAGACTTTCGTTCCATCCCTTATGAGATGAAAATTTTTGGGAATTTC
TCCCTTGAAAAACGGATTTGTCTCAGAACCATCCTTCTCTCTTTGCCTTTGCGTAAGCACAAGCTCTCACTCTTTCTGGG
TCCATGTCGTTCCTCGCATAGGCAAGGGCGGCTCTCGCTCTTTTTTCTGTGTTCACGGGAAAAGAACCTGGAACTGTCCC
ACAAAAGTCTGAATCTCTCAGATGTTCCTTTCTGTATTTTTTAACGTTTGACGCTCCTGGAAGGGTTCCTTTCTTTGTCA
GTTCGAGTGGCTTTTCTTCAGGGAGAATTCCCCTTTGAACGAGAGAAATGTGGGTGGGACCTCCTCGAAGAATCTTTTTT
CCTGTTTTTGGGTTGATGAGAGCTTTGTGACTCATCTTACCTTGTGTCAAAAAAACATATCCAAGATTCTTCTTCTGGAG
ATATTCCATAAATTCTTACTTTTCCTTTGTGAAGAAAAGCCCTGACAACACACCAGAAAGTGTTTAGAATTATTTCTCCC
TTTTGTTGTGCCATTTCGAGGCACTCGTCTTCCGAAATTTTCTCTCCTTGAACAGAGAAACAACTGGTTTCCAGATCGGG
GTCGTCGGCGAGCACAGCAGATTCGTCCATATTTCTCTTGTTCTTTGCAAGTATTTCTGAGAGTCTTTGAATGGTGCGAT
AGACCATGAGTTTGCAATGAGTTTCATTGTTCACACAGAGACAGACATCGTTCGCAAACAGAAATCCCGGAGTGGCAGAA
AGAAGAACGCCGAGCGTATCCTGGCCCTTTCTGGGGGATGGAAAGCTTGAAAATTCTCGAGGAACGTTTTTGTGTTTGGA
CAGCCACAACAACCACATGGATTTTAAAGTTTCCAAACTATATATTTTTTGGATGCAAAGGTCCATGAGTTTCTTTGGTC
TTGGCACGCATTGATACGCAGAGAGCCAGTGCTCTGAACCCTTTCCCCTTTCTTCTGGAGCGTAAGCGCTCAAAAGTCTC
AAAAGACCTGTTTTCCTTGCGAGAGTTCTTTCTTGTCTCTTCATCCGCGACAATCTCTTCTCTGAAAAGAGCTGGAACGA
AAAAGCTCTGTCGTAAAGCCAGAGCTTCAGCCTTATTTTCGCAATCTTCATCTTACATTTTCTTTTCAAAACAAAAGAAA
GTTTTTAAAGGTCGAAAGAAGCCAGAAAGTTTTTGGCGATGATGTATTCGATGCTGCCTTTTTCCAAAGAAAAGCTCGTC
CTGCCCCCATCGTCCCAAAAGACGATAGAGTTCTCTTCCAGACGAATTTTCTTGTCTTTGTAAAAATTTGTTGTTCCTCC
CTCGAGCCAGGTGATGGTTATTTTGCCCTTCTGGCGACACTCCATGTTTTTGTACGAAGCGTTCCAATCCCTCGACGGTC
CAAAAGACTCTGTTTTCACCAGCCTTCCGTTTTTGTAGGTGGTTTTGTCCACCCATCTATAACCTCGTGCAATTTCTATC
CCGTGTTTCTCTCCGTTTGGAAGTTCGTGATGGTATCCTCGATCCGTCATTGTCTTTTTGAGAAACCTCGCCTTTTCTAC
TTTCGTCTGTCCTGTGCAAAGAGACCAAGAAACAACTTCTCTTTTTTCCAGAAATTTCTCCATCTACACTCCGGGGAGGT
GATAGTCCTTCATCCACCAAAGGCCGGAAACTCCTCGATTGATGGAGATGCTGTTGATCGGATATTTGATGACGTGCGCT
GGAGCGTTGAGAACTCTTCCGTTTTCTGTGTCGTACAACCAGATAAATTGCGGTGGATAGCCGTATGGATTGACTCCGAG

GTCCGCCTTTGTTCCTGGCTGAAGACGAAAAATAATGGGAGGAGGCTGGGATTGGGAGAGACCTATGCCGACATCCACAG
CATGGTCTCTCGAATTGTTCTCGAGAGCGAGATATCTCTCATCGATGGATTTGACATTCTTTTTCGAGAGAGGATAGGAC
CCCTGACTCACTCTGTAATCCTTGACTTTCGAAACGACAGAACAGGGCGCGTACGGCTGCATGCAGTTTGCAACTCTCGA
GTTCATCGGTGGCAGCGTGTCAAAAGGCCCCCATTGCTTCCACCAGTCACCAACTTCGAGAGGTCTTCCTTGTGTGTCCA
AGAGTTCCTCCCAAGCCTCGCAATGACCTCTCCCGCGACAGCTTCGAGATTTGCAAGCAGGGCAGAAAAGACCTGGATAT
TGAGGAACTCCTTCCGGAGAGCTGACAGCAGAAACCCAGTCGCGAGCGGTCGCCGCTCCAGAGACGTAGAGGTCTTTTGT
GTTGTGAGGAAGGGAACGAGGGTCCACATTTCCAGGAGAAACTCGGGGACGTTTCTGTCCGACGTTCATATCATAAATTC
CTGACCAAATAACATCTCTCGAAGTCAGAATTCTTTCTGGGTCATCTGGCCTTTCTTCAGAAAGCAAACAATACATCTTA
CTAATCAACAAACTCTGATCGCAAAAGCTTTCACCTTTTTGCATCAATGGATTTCCAAATAATTCGAGCAAAGAGGACTG
TCGTTCGGATGATGTCTGACAGAGGGTACATGCCCATCAAAAGGTTGCCAGACCCCAGAGAATCTTCAGCAAAAGAGCCG
ACAGTTTGGGAGTTTGAGAACGAACAACAAAAGAAGGCGAACGTGTTTTGGGACCTCGACACCGAGTCAAAGATGCCATT
CATTCAGTATGTTTTTGAGGTGGCGACGATGGACAAACTCGAACAGGTTGTTATTGTGGTTCTAAAGTCCCTTGTTTCGA
AAGCAAAGAACACTGTCGTCTCGATGTCTGCAAACGTCGAAACAACGGTGTTTGAGGTTTCTGACCTCCAGGTCATCACT
CCAGACCATGTTCTCGTCCCTCCTCACAAGAAGCTCACAGAAGAAAAAAAGAGGGAAACACTGGAAAAGTACAAGGTAAA
ACTTGAAAATTGTCCAAAGATTCTCAAGGATGACCCCGTCTCCAAATGGTACGGTTGGGTTTGCGGCGACCTCATCAAGA
TACTTCAACCGAAAGAGGGGAGCTTTGGGTCGAGCGTCAACTACCGTTTTGTTTGCCTAGATTAGAAATTTTTTCATAAA
AATTTCTTTAGTAATGCACAGCTCATGGCATGACTTTATTCAGGCGCACTCTGGCAAGGGACACTCGATGGCTGAACTCG
CTGCTCTTTACAAAAAGAAGCACGGAGCGAAGCGTGGTGGAGCGATGAGGGAAGAGGATGTGATTTGTTATAAGCGAACT
CGAAGCGCTGCTGGCAGGAAAAGGGCTGCTCCCAAGAAGAAGTCTCCCGCCCAGAAGAGGGCTTCTCCTGGAGAGCGCTG
TCGCGACGAGAGGGGCCGGTTCGAGAGTTGTTAGTTTCTCCATTTTGAGAAGGCGTAGCCTGTTCCTGCAAGGGCAGTGT
AGACCACCATAAACAAAAGAACAGAGAACAAAAGCTCGAGAGAATTCGGCTTTTCATCGGGAATGTGGCTCATAAGAAGA
GCGAATAGGGAGCTCAAGAAAATGCCAAAGGCAAACAAGGAGACCGCGAGTCTCGACATTTAGTTTTCTAAAAGTTGAAA
ACTAAACGCTGTAAACACGCAAAAGAATTCTGCGATATGAAGCTTGAGCTTTCCCTCGAATCTTTGCCTCTCGAAATATG
GTGCCACATCTTTGGCTTTTTGGGTGATGGAAACGACATGCTGGCGATGAGAACGTTTTGCAGAGAGCTTCGCGACATTG
TGGATGAAACCATCAAGAAAAAGATAAAACATGTGGTCGAGAGGTTTGGCGAGAGATGTGGCGGAAAGCACCCGAAAAGG
TCAAAAAAGGAGGGATGCGCTCGCTTGATGTGCCTTGGTGACTTTCGGAAGCAAAAGCCGATAATGAGCAGATACCCTCA
TCCGCCTCCTCCAGTTCCCGTTGAAGCTCCTCTTCCAAAGTGCAAGGACTGCGGTCTGGAAATCAGACAAAAATCCCCTC
TCATTTTTCACATTCCTTTTGTGGAGGAAGAACTGCCTTTTGAAACTTTTCTCAGAATCATGGACGTGTTCACCGGAAAA
ATATGCGTTCCAGGAAGGTACGAGAAACCCTTCGACCTGAACGAGTTCAAGAAAAGGGTGGAAAAAGTTTTGGTGCTCAA
GTTTGGCTTGAGATGGGCAGAAAGAAACAAAAAGCGCTTGTTGGATGGATGCGACTGGGCAAACAAAAACAAGCAATTGA
TGGCTGTTTTGAGGAAGTAGAAGAAATATATTTTGAAAGAAATATATTTTGAAAGAAATATATGCAGAGCTTTTTCAAGA
ACGGAGAGTATATTTCCTTTTGTTTGTCAGAAGGTGTTCCCCCTCTCAAAGAACAGAGAGACAGGTTTTTGAAAAGGGAA
GAATGTATGGCTCCATCAAGGATAGGGAATGAACTTTTGAAGCTCGTCGTTTCTGAGCTTAACGAAACAGAGAAAAAGGC
TCTTCTCGGAAATGCGTGCGAAACAGAAGACATCGAGAGGTTCGGTACGCTGACGTCCTGGAAAGGAAATACAAAATACA
TCGCAGAGAACGACGATGAACTTTTCAGTCCTTCCGCGGATTTTTACTGCGACATGTTTGAGAGGGACATGCTCAGCACT
GTGTCGCTCGTTAAGTTTGTTGAGAACGACAAAGACAAGCTGGCAAACATCGCGTACCTCTTGTCTTCGATGCTTCAAAC
GATGAAGGAGAGGAGGAGAGGATAATCCAAGAAATTTTCTAATCAACATATAGAAGAGAACATATATTTTGAGAAAAATA
TATGCAGAGCTTTTTCAAGAACGGAGAGTATATTTCCTTTTGTTTGTCAGAAGGTGTTCCTCCTCTCAAAGAACAGAGAG
ACAGGTTTTTGAAAAGAGGAAAACACATGGATGTATCAAAGATAGAGAACGACCTTTTGTCTCTTGTGGTTGGAGACATC
GACCATTTTGAGAGGGAAAATCTTTTGGAAGACGCTTGTGAAACGGAAGACATTGAGAGATTCGGAATTCTGACGTTTTG
GAAGGGAGACACGAAATACATTGCAGAGAACGACATGGAACGGTTCAACCTTCCTATAGATTTTTATTGCGATATGCTCG
AGAGGGGCATATTCGACACTTTTGTCTTTATGAAGAGCGTGGAGAAATCTGCCAGAATGTCTGCTGCTCATATTTTGTGT
GCGATGCTTGGGATGATGGACGAAAGGAAGAAAAAGGAGTTGGAAGAGCTTCGGTCAAAGTTGGAACGTTTTTCCAGAGA
CGAGATTTGAAGACTTTTTTGTTTCTGGTGTTGAAGAACCATATCTTTCAAAAGTAAAGATATTTTTTAGCTTCTGTCTG
GTAGGACGAGGAGACCTGTTTCGTGAGCGTGGCGGATGTTTCTGGAGGGAGTGACCCATTCGAGATTCGAGACTTTGTTG
TTGGACGCGAGAGCGAGGAAATTTCCATCTTAGTTTCTGGGATGGGGCACCATATTTCTTCCATATCTTTATTCTGAAAA
TATATTTTTAACTTCTGTCTGGGAGGGAGATGAGACCTGTCTTGTGCGCATGACGGTCGTTTCTATACTCTTCTCTTGTT
GTTCGCCCATTTATATTTTGGGCTATTGTTGTCATTGTTGCCCAAGAAAGGTTGGAGACTCTGTTGTCGTCTTTTCGACC
ATTTATGTGCATCACAGTTTTTTGTTCTTTCTTGGGTTCCTCTAAAAATGCCTTAGCCACGAGCCTATGAACCTTCATAG
TTTTGTGGTACACATGGCCATTGTCACACAAACTTCCACAGGTTTTTTTCCTTGCTTTTTCTAGAACTCTTCCAAAAGAA
GAAACCGTCACTGTTTTGTTTCTGTAAAGAATTGTCCTCCATTCTTCTCCGTCAAGGTCAAAAGAGGACTTGTAACACCA
TAGAAATCCCATATACTTTTTTCCAGAAGATAAGGCTCTGCCCAATCCCGAAAAACTTACCCCCTGCGTACTCGAAATAG
CTTCTTCTTTCGTTGCAAACTCACGGAGAATTTCGCCCATTTCATTCATCTGGACAATGATGTCTGTGGACAGTTCCGTT
TTTTTCCTTCCAATCTCTTCTCCTCTGCGGCAAATTTGCAAGTTTTCGACTTGGAGATTGTAGGGATTTCCATCGATACA
ATGAATCACGTTTTCTGTCGTTCTCTCTCCAATGAAAGCGTCCGCGACCAACTTTTTCACAGAAATAATTTTCCCTTTGA
TTGTCACAACCACATTTCCGTAATTTTCTCTCTGCCTGAGTAAAAAATTTTCGGGGGACGACACTCTCCCTCTGCTAGAG
ACTTTGAAGTTTTCCATGTTTGGTATATCTTTCCAGATTTCTCCCTCGAGATCGACAACAACCATAGACTCTTTTTCCTT
CTCGCTAAGGAAACTATGCCTCGTGTTCCAAGAATGCGTACATATCTCTAAATTCTCCACTCTGTTATCGGAAGGCTGAG
AATTGCGGTGGTTTACCACAAGATCGTCTCTGAGTTCTCCACAAAAAGCTTCGGCAACCAACCTATGAACAAAAAGATTG
GAATATTTCATGTATCCCCTGGTGTTCTTCTTGCCAAATGTTTTTATGTTCCCATTGAGCAAAAGTCTCCCTTTGTTCGA
AACTTTGAGAATGTTTCTATTTTTCCCCGAATAATTTTTCCAAACTTCTCCCTCTAGGTCTGGATCGTCTCCCCACTTCC
ACACAAACCCGGCAAAAGTTTTGAATTTCCCGTTACAACAGTCTCTGACCTGTTGAACCTTCGAACTAAAGACTCTCGCT
ATTTCTTGGGGGCTCTCCCACAATCTGACAAAAGACCCATCCTTTGTCCATTGTTCGACTGGTTTTAGTCTAAGAAATTT
TGTTGACTTTCGAATCCCGCGAGGAGCCCAAAATAAATTGGAGACATGGTTGTTTTCGCAATCCTCGTCGTGTCTGTTTA
CGCATTTCATACCCTCTGGGTTAGGTAAAAAGGCCGCCGCCACGATTCTTGCCACTCTCACTTCTTTTTTTGGTAGTCTG
ACATGAGAAGCTCCTCTCACAGTTTTACACGGGACCAATTGTCCATTTTCATTCTTTATTCTCCCGCAAGAGGACACTTG
ATACTCATATCCTTCGATGGGTTTCCAAATCTCTTCTTCTCCGCAATCGCACATTTTTGAAAGAACGAGGAATTTTGAGT
TTCTTTCGCGTTTTTTCAAAAGAAATATTTTGTTGACAAATTTATAAGTAATATGTCTTGCGTTCCAGGAATGTGCGATT
CTAGTAGAATGTCTAATGGAAATTTTGGTGGATGCGGTGCTCCTACAACTCCCCCAGGATTTACCCTCCTTGGCCAGCTT
CCGTATGGGGGGTATTATTCGAATTTTTTAATCCATGCGTACAGACCCTCTGCGGCTGCGGCGAACATGCAATCCTACGA
CTGCGCCACGAGGGTACATCAGCCCTATGCTGCTTATCCACCCACTATACCTCGCACCCAAATTGAGAGTCTCTACCAGC

CGAGAGCGAGCCGTGGTTGGGTCGGTTGTCGCCAATAGACGTATCCCTTCTCAAAATATCATTTCGAAGAAAATATTTGA
CATTTCAAAATATTTTAACAAATGAAACGCCAACGCACCTCCCTTCCCCTCTCTCCTCTCCGTCCTTCTCCTTTCGTTCG
TTTCCGAGACATTCCCCTCTGTGTTCCTTCACAGACGAACCATTCCAAACAGACTCCTTCTGAACAACTCCCTCCCTCTT
TTGAGAGACTCGAGAAGAGACATTGGAAAACACTGAAATTCTGATGGATAAGAAAGGAGCAATTTGTCTCAACAAAAATC
TTAGAAATATGGAAACTCTTTCGTACGATGAAGAGCGAAAGTTTCCCACTTCTCTTTGGGGGACGAACCCCAAAACTTTC
GCCAAGAGAATGAATCATTTTGACGACAGCTCTCTCCGTTGCGAAAAAGATATGAAGAGACAATTCAAATCTCTGGATGA
AGTTGACCAGAAAAACTAGAGTTTCATCAGATAAAATTTAAAGAAATTTTATCATCATGTCTTGTGAGTTTTCCGAACAG
AACGCTTTTTCTCTCCTTCTTCAGCTGCCGACGGAACTCCAAGAAGTGAAGGAGCTTTGTTCTTCAGAACGGGACAATCA
TCCACAGAAAGAACAAGCTCCCTTTTGCTCTCCGACTCTGGACAAACTCGACCAAACAATCCTCTCGTGTTTCTACAACC
TTGCTTTGGAGTCGTACACTGTCGACAGACAGGCGTTTCCAGAGAAAATCAGACTTGAAAAGATTCTTTCTTTGGACGAA
GAAGAACCACAAAATGTTCCTCTGAACATCAAAGACGAAGGAACTTCTCCTTCCAAAGAACTTTTGTGGGAGAAAAAGTT
TGGAGAGACGCAAGACAACAAATTCTGGGAGAAAGTTTCGGATTTGTGGAACAAGGTCGACTAGAAAACCGATGCGCCTT
TGGTGACTTTGCTCTCAAAATTTCACAACATGTCTTTGGATACGGAGTTTTTCCACGTCGCTTCCGAAGTCGAAGAAAAT
CCTGAAATCTCTTGGGAAGAACCTTCCAAACCCAAGAGGACGATTTGGGACATTTCGTTCTCGTCCGATGACGAACCTTG
CGAAGAAGATGCAGAACAGTTTTACAGATGGGTATATTGCCAGAAGAAACAGAAAGTTTGTGAATAAAATTTGAAGAAAT
TTTATAGTCATGTCTCTCCACGAGAATAGTGAAAAACTTTGGCCAGAATTCAAAGACCTTTCTGACACTTTCTCCCTCCA
ACAAAGCATCTCGAATTGGAACTTTGGAGAAGAGAGTCTTTGGAAAGACGAACCAAAGGATTACCGAGATTCGTCTGTGT
TGTTTCGAACTCTTGGCGAGCAGAAAACGGGAGGACTACATGCAAAGAGCATCTCTCGAGAATGAGATGGGAAGAACGAC
CATTAAAATATTTTGTTTGTAAAAATATTTCAAAATGTTCCTTTGTCTCTCTTTGACCCTCCTGTTTTTCTTTTGAAGCA
CCTCAGAGTAAAAACTCGCATTGTGGGGATTCGCAAGGTTCTCTTTATCTTTTAGAAATTCTCTCCCTTTCTGGGCAAGT
TTCGTACAGCCAAAAGACTGAATTTCTTCCGTTTTGTGAAAGACGTTGATTCGAGCCTCTGTTTTGCCAAAACATGAAAG
GCGTCTTCAGATGCACGACGAAAAGTATCATCCTTGTCTAAAAATATTTTTTAACCCTTCCTTCTACGGTCAACAAAGAA
ATTTCTCGGTGTGCGGCGAAAGCAAAAAAATGCAACTTTTTCAAGAATTTTAGTTGGGTTCGAAATAAAATATTTTATC
TGACAAAATATTTTGCGGGAAGGGAGAAATTATATTTGTGGAAACGCTCCTTGTCGTTTTGGAGGAAAGGTGCTCTTTT
TGGGATTTTTGAAAATTTCCCAGCCCAAAAAATTTTTCAATCCCAAAAACCTCGTCCTGTGACAGGGTTTTTTGAGACCT
TTTTATGGTATAAAACCAGCCGCAATATATTTGCAATGATTGCAAATATATTTATGGAGAATCTTTCTTGTCTTTATTGT
AAAAAGAAATTTTCTAGCACACAAGCCCTCAAACTTCACACAAAGACAGCGAAAAAATGTCTTCTTTCTCGGGGAAACTC
CGAATTACTCGACTGTGACTACTGTGAGAAGGAATTTCCTGGAGAAGAAAGTCTTCAAAGACACCTTTTGACTTGCAAGT
CCAAAGAAAAAATGTCTCATCTCTTCGAAGAGAACAGAATCCTCAAAGAGGAACTTTCGACAGCGAGAAAGGAACTGGAG
AAGTTCCGAGCTTTTCGCGAAAAAGAACTCACATCGAGCAAAAATACTTTACTCGATGTCGAAGATTTTCAAAAAGAATT
TTTTGATGCGTTCGAAAAAGTTCCTACAGACACTGTCATAAAAGGGTAAAAGAAACCATCAAAGCGATAAAAGAACTTTC
TTCATCTCAACGCAAGACCCTTTGGATTTCAAACAAAAAGAAAAAATCTGTCACGTTCGTGGAGAAAGGAGTCGCAAAAA
CGGAAACAGAAGATTGCATGGATACATTCTCCCTCGTTGCTGACACGTTCTTCTCAAGTTGCGTTCAGAAAATAAATTCC
CTGATCTGCGACATATCTTCTACAAGAGAGACGTATAATGTATCCTTTATGCAAAAAATTCTGAATGCACAGGATAGCTA
CTCCGAGAACAACCGCAAACGTTCCAAAGAATTTTTTACGCAGTTCATGGACAAACTGTCCATGTGTAAAAAAGAGAAAG
TTCTGGATGGGGCTTTACCTCTTTTGTTCGAGCTCAAGAAAAAAATGAGGGCCTTTGGTCTTTCGAAGGGAGGGGGCATC
GTTTTCTTAAACGGAGGAAAAAGTCAGGGAAGTTTCAGAAGACAAAGGAGAATTTTTTAGGTGTTTTATCGAATTGGTGG
AAAGATGTAAGGACGTCTGTGTTTTTGAAGCTCATAGCATGCCGTGCAGCAGCCACCTAAAAAAGACAGTGAGGGCTTCC
GATGAGTATTATTCTCTCGAATATGGAGCATTTTTGGAATATATGAAAAAAGATGAATTTTTTTCGTGATGTATTCCCTC
CCGCCAAAGCATCTTTTGTTTTAGAAACAAAAGATAAAGAACCTCGAATGAACGAGTGCTAACAAAGACTCTGAAAATGT
TCCTCCGCCTCCAAAGCGCCTTTTTTTCCTGGAGAATATTTGAGCTCCCTCTTTTTATGACGAAGTTCCTCGACCCTTTC
CCCGAGGAAAGAAACGCGTTTCCAAAGGTTTTGAATTTGCTCTACCTTTTGAGAGGCGAATTCAGGGCATGAGAAGATAA
CTTTTTCAACGTCTTCTTTCAGGAACCCGAGAGCATAATCTTTGGGTATCTTGTGGATTTTTTCCTCGTTGAAAAGAAGC
AGAGCTTTATCCGTTTCTCTCCACCTGTTTCTTTCGAGTGAAAAGCCCTCGATGTCGTACACAACTTCAAAAAGATTTGG
AGAAAGGGCTGTGATGCCCCATAAAATATCTTCTTTTGGAATGTTCAAAGACTTTGTAAGAAAAAGGTGAATTTCGTCTT
TTAGAAAACTTTGCATTGTTTGATATTTTATTGGATAAAATATCCTTAGATTTTTTGAAATCTTCCACATCAGCTCTTTT
CTTTGTGTCTTCTTGTTGAGTCGCAAGAACGAAAACGTCCAGAGAGGAGTACCACAGCATATTATTTTACCAGCGTAAAA
TAAATCAGAGAACTGGAGACAGTCCGCCCCCATGACAACCGCATCTTAAGAAAGTTCCTTCTTCGTCGGCGTATTTCTCG
ACATTTTTTCCCGTATTTGGACAAACACCACTCCCGCAAAGGACGAAAATCTCGTCTTTGTTTTGACGAGACCCTTTTAG
CTCCCGCGAGACAAGAACTCCGTTGCTCCATTCCTCTGTTTGCACGTAGGAAGAGTATTTTCCGAAAACCAACATGAGCG
TTCCCATTGCCGTTTTCGTTGTGGTGCCGTGCAGAACACCTTTATGAAACATTCTCTTGTTTTTGACGATGAGGCGGCAC
TTGAATTCGCTGCTTGTTTGTGGTCCGTGAAACTCTCCATTCAGGAAAGAGTATGTGTTCCCAGAAACCCTTGTCTCTCC
CTGGAGCTTTCCAAGAGGGTCGATGTGGTAATGACCCTTTCTTTTTCCCCAGAGTTCGAGTTTCTCAGAGAGGCGAAGAG
GAAGTCTGTCAAAGAAATTCACCTTTTTGTGGAGGAACCTGCTGTTCCTTTTGACGAAAGCGTTCCAGGTTTTCGCAGTT
TCCCCAAAAGACACGAGACCCTTGATGTCTACAAACTCGAGGATTGCCAACGAAATCTCTTCGGGAAACAGGGAAAACAT
GTTGAAAGAAGTATCCATCAGATAAAAACTTTTTCTCCAAAAGTTTTTGAAAGGCATCCATTTCACAAGAAATTATCTCC
TCTTCTTTTTCTCAGACAGTCTCATCATCTCCTGTTTCTTCCTTTCTCTCCCAAAAATCTCTTGTGTTTCCGATGGAACG
AGAGAAGGAAATATATGGGCCAAAGGTTCATGGTGGGAAGATTTCCGGATCTCTCTCGCCACTTTTTGTTCTATCCTAGC
TTCATTCCAATAAACATTGTGGGGTATGATCTCACATCTGTTCATGGTCAACGGGCGAGGAGAGGAAATTTTGTCAACGG
GAACCTTTGGCCCATATATTTCCTTCATCATCGGTGACTCCTCGGGAAGAATTCTTCCCAAGGCTTCGGAGAAACAAACA
AAATTCTCTTGCATCTTTGAATATATTTACTGAAGAAATGTGTTAGTCTGTTTTTGCTTTGACCCAGCGATAGGCAGTGT
ATGGAAAGACGGCAGATTGTATCCACGTTTTAGCAAGAAGAAGAGAGAACACCTTGACGTTCATCTCTCCTTTGTGAGCA
AGGCCGTTCGAGAAGCCGATGGGAACAGAAAAGATAATCACTCCCCACGAATAGGCGTTCACGAAACTTTTTGCTAGCTG
CATTTTACCGTTCCGGAACATTATGAAAGAATTTGCGATTATCCCTTTCATGCGTCTGCTGACAAAAATTTTGAGACGCA
CAAACGACGCGCAGATTCAGAGACAGGAAAATATATTTTGAGAAAATATATTTAAAAGGCTGCAAGACGATACAAGGCCT
CTTTGTATTCTGCGTTGTTGGAGAATATATCCATAAATTTCGAAACGTCAAGGTTCTTTATAGTTTCGATGAGTTCCTTG
TCTTGTTCTTTCTCTGGGGAGCTTCGCAAGAGAAACTCAAGGAAAAAGACCATCGCCGCTCTTTCTCTGCGCGATGTGAG
AGGTGACATCAAAACGTTTGTCGTTCTGCACAGCTCGTAATAGTCGAAAAACAGTTCACTGATAAACTCTTGAGGAAGGA
GAGAGAAGAACTCTGCTGATGACATCCTTTACAGAAATTTTAATACTTGGTTGTTCGAGGACGTTACGTTTCATTTTATA
CTATAAAATATCCAAGGAAAAGATGTATCTTTCCAGACCCATCGAAAACTTTGAAGCAGAAAAGTTCAAGAAAAAGGTAA
ACAAAGAACTCGAAAAGGTGGCGTACAAGCTCCACAAAACTCGCAAGCTTTGCAGGTCAAATCTCGAGAGCGAGGAGAAC

GACAAACAATACAGAAGGAAAAAATACCTTAAGCGATATAGGTCGTGGCTGAACGAATGTCTTCAGGACCTCGACGACAT
CCCACAAAGGAGTTCCTTGGAAGTACGATTATAAACCAACCGGAGGGTGGAGAGGAGTTTCCCAACATTGGTACGAATGG
GTAGATCCAGAAAAGGAAGAGTTAAAAACACCAGAAAAGAGAGTTTCATATTCCACCGTTGACAAAGGACCAATTCATCC
GGCCTTTGGGAAAAACGTGCCAGACGACGGTTCGTATTTTCCTCGCCTTGCGATGACGAAAGCGGACGCTCTTGCGGCGC
TCGGGTACGACGAAAGTTTTATCGCTGCGCTCTGTCTTCCATAGAATATACTTTCGTGACACAAAAATATTTACTTCCTT
CTCTTTCTTTCCTCGCGGAGAGACCTCTTTTTCTGTTGTCGGACCTTCTCTTCTGTCATCTGCTGTTTCTTCCAATTCTC
AAACTCCTCGAGAGGAGCGATTGCCTCATGAGAGGGTTTTCTCAGAAAATTAGAAAGGATGAGACCTTCGATGTGAACTG
GTTCGCGTTCGTCACAGTCTCCGTCCCAGTCTCCATAGTACGCGCCGATTTTTGACCTTTCGATCGCGATCGAAGGACGT
TCATTCACGAAAGGGAACCAACGCTCCAGAGTTCCTTCTTTCTTTTTGTAAAGTTCCGCTTCGTCGTCGTCAAGAGAGAA
TTTCACAACAATGTCGAACTCTGCAAAAGTCTTTTGCCCCTTCTTAACGAACGAAACGTTCACAGTCTGCATTTTTATGG
ATGAAAATAAAGAAGAATTTTCTCCAACAAATTTTTAATATTTCAAAGGAACGAGTTTATCGCTGTGTAGACGTTCGTTC
CGCTCTCCGCTTGCAAGTTTCTACTTCTCGTATAATGTGCTCTTCTGCGGGACAAGTCAAGATTTTGGAGACTCTTTGTT
CTTGATGGATGGGGAGAAAAGAGAGTTTCCACTCGACAAACAAAGTTTTTGGGATGCATTTGTGGATATATTTTTATCTT
GTAAAAAATATTTAGTATTTCCATCCAGAACTTTTTGTAAAATTTCTTCCTTGTACGTTTGAAGTGAGTCCTTCACGGCT
GCCTTTCGAGAAAAAGTTTCAGAACGAACTCATCCTTTGGGGGGAAGCATATCAAAAGGAATTTGACGTCGCAGTAGAGG
CTGTGTCGGTACGAGTCCGTTCTATGAGAGGATTTGAGAAATGTCTGGACATTGAACAGAGACAAACACTTGTGATGCAC
TCCAATTCTTCTCTTCCTTTTCAGAAAAATCTAGAAGATAAAGCTTTTTGAACCGCTTGTCGAACGTCTCAAGGCAGACA
CTCTTCATCTTCAGAGAATATTTTTATGCTGTAAAAATATTTGGAATGAAACAAAGGATAATTCACCCAGGGAAAGAGAA
ATTTCCCTAATCAAGAGGAAGCACTCGCCATTGACCTCTTGAGTCGTCAAAGGTTTTGATGAAGAGGGAGCTTTTTGATG
TGTTCGCGAGAGACGAATGGTCGGAAGAATAAATATTTTTTTACATTCAAAAAATATTATTTGTTCGAAAAGAACAGAAT
GTTTGAAAGAACGACGGGAAGCGACAAACGCGCAAAAAAACAGTTACCTATGTATTTTCGTTGTCAACAAACGCGCAAAA
AAATAGTCACCTATGCATTTTCGCTTCCGACAAATACGCAAAAATATCAGTGACCCCTGTGTTTAATCAGACGAGGGGTC
ATCATTTTTTTGCAAAAATTCCATGAAGTTTGAATTTGTGGAATTCCCAAGGCCAGAAATTTTTTTTGAGAAGATTTCAC
TTGTTCGCAAAGAGTGAAGATGTATATATTTGCGCAAATTATCACATTTTTTATTGAGAAATAAAAATCCGAGACTTAAT
GGAGCAGTACGAGTGCGAACTGTGCAACAAAAATTTCTTTACCCTTTCCAACTACCGGCAGCACCTAAGAACTAAAAAGC
ACCAAAAAGGTCAAATAAAACAAGAAAAATTCTTTTGCGGTGCCTGCCAGTATGAAACTACTGTGAAACGCGACTATCGT
TCCCATCTTCTCTCGATGAGGCACGAAAGGAATTTTAGAAACAAAAGAGTGGTCCGAGAAGGGTTTTGTGAATGCTGCGA
TTTCGATGCTGGGTGTCACAGCAAACTACAAAGGCATCAACAAACGAGACAACATGTGCTTTCTTTTGAGAAATTTTGTC
GTCTTTCTTCAGAAAAACTCAAAAAACTCGAAGAAGACATCCTTGAGTGTCTCCTAAACGAGGATAAATCTCCCTTCACA
GAACAAAAAAAGGCGTTTTCTGTTGACCTTTGCCCTGTGGGGCTTTGTAAAAACCCGAAAAACGTGCACTCTTTCTTTTT
GTGGATAAAAACAACCATAAAAACCATTGAATATTTCGTAAAAGATGACGAACAAGGAGTTTGCGTCATTTCTTGGGTAA
ATCACAAGCATAAAATGTCTAGAGGAGATTTCATGTATCTGATGTTGTCTTTGGCCGACAGAGTCAGAGAAAACAGGGAA
AAGTTTACAAAAAGAGAGGTGGAAAGGAGAGTTTCAGAGGGTGAACGGGCGACATTCTGTTTTGACGGACAAAATGGGCC
AGAATTCGAGAGTGTGGTGATGAAATATAAAAATCTTTGTTTTGAACAAACTCACCATGCTCTTCGTCAGTGTGAAATGT
TTTGGAGGTGGTGGAACGAAGAAAAAGAGTTCCACCACCTTCTCCGACAATGTTCCTTGTTTTTGCGAGTAGAAGAAGAA
AGTAGACCTTCTTACGGTTTAACGAGGACTTTGCTTGAAGAAGATAAATTTTTGGATGTTCTTTGTAGGTACTCAGCGAG
GACACGAGAAGAATATAAAGAAGAAATTTTGGCTCTTTCCTTTCTTTCTCGCATCGAAAACTTCCTCTCTCCTTCCTCAA
AACCTGAAAACTGTCCAAAGTTCTATTTGGCATTTTCTGCAAGGAGAGAAAGGGTGACGAGGGACATTACTGCTTCTTAT
TTTAATATGGGGAAACCGGGACCTTTTTCGGCTAGGGTAGTTTCCATCTTGTGCGAGCGAACTCAAAAAGCCGAATAACA
GTTTAGATAAACTGTCGTGTAGTAAAATGGGGAGTAAGAAAAACACCCAAGATGTTCGTCTCGAGTGCGAAACATGCGGC
TTTTCATCCACAAATGATCACGAATTTGGAATTCATGTAAGAGAACACTACGAAAAAATAGGCCTTTCATGTTCAATGAA
ATGCCATTCTTGTTCGTTCGAAACAGACTCAGCCACACTTTTGTGGATGCACGTACAAAAAAAGAGACACATGGCGTCTA
AACTATATAAGCAAGCTCTCGAACATTGCAAAGGAAAAAGGAGCGACGCATATGCTCTAACAGCCCAAACGTATGGCGAA
AAAACAGCAACGAAAGTGTCAAACGACCTCGCGGACAGATTCGTGCCGGTGAATGGCGAATGGATCATTTCTGTCTTGCC
ATAGAATATTTTTTAAAGAAAAAAAATATAGTTCATCACCGAACCCCCAGAAACGTTCATTTCTTGGAATGGAAAGATTC
CCATACGAGCAATTCTCATTCAGGGCGCGCAGTTCGGAATATACCAGTGCGGTGTCCACCTATTTTCGAATTTTTTCCCG
CAGTCTGGAGGACATCCGTACGAGCCTGTGACGACGCCAGCGCTTTTTTCTCCGATTCGGTTGTTCCAAGTGTTCCAGCC
GAATCGCGAAGAGACGTTGGGGTCGGGTCCATGCTTTAGTTCGTACGTATTCCCGTCTTTCTCAGAGATGTATCTGGTGA
CTAAAAAGTCAAAATTCCAGTTTCCTCTGTCCGCGATGTCTCTGGCAAAATATGGACGCTCTCTCACCCAATCTTTTTCA
TAAATATTGTATGGGAAGTTTGTACCTGAACCTGGCATGAATCTGGGAGCACACCTTCTCGGGTCTTCTCTCGTGTCTTC
GGTCGTCTGTGACAAAATCACGCGAGTGGGAATCAGATTTGCGATGGCCAATTCCCTTTCATAGTTTGTCTGAAAAGCGC
CAGACATCTTTACAATATTTTTACTTCCATAAAAATATCTTCAGTAGAAAAAGTGCTTCAAACGTTTCGGGTACGCTCCA
ACAGCCAAATATTTTTCTTCAATGTATAGACTACACAAATGCATGGGAGGATGAAGCTCTCCCATTTGAGGGTAAGTAAA
ACAATGCGTTCCCCTTCTTGCTCTATAGACGTGTTCGCATTCCTGTTGCGTTTTACATCTCCAACCATTCGCCACAAATT
TCACAAAAGGGGATGTGTAAATTTTTCTTTCTTAAAAATTTCGGATCAGAAAAGACCTCGTTCATTACACGTGAGAATTA
TGTCCTTTAGTTGCTCACGTTTATCACAGCATCGTGGTTTGTCCACAAGGGTATGTCTTTCTGTTCTTCTGAAAGCTCCT
CGTCCGACGCAAGAGTGTTTTCCAAATTTACCTGGTTGCCGCAATCTACAACATCCCACTTAACGCTTCTGCCGTATTTT
CCTTCCCATTCTTCATCATAGCCGAGGGCATGGAATGCTTGATCTTCTGTCAAAAATGGTCGAATAGCCACTGCTTTTCC
CTTTCTGTCCGCTTGTATCATCACCCTGTAAAAAACTGCAAATTCCAAAGGGAGAGGTGTATTTGTTTCCCTCATTTTAT
CCACAATCTTTAAAAATTTTCCTTGAAGCAACTTGTGTTCGTCGCTTGGCGACGGGAGATTCTCTTCCATTTTATGTAAC
AAAGTTTTGTTTGAAATTTTATGCGTTGTGAAAGAGTTTACGACTTTTTTTGTCGCCAAAGGCAAAGGACGCGCCGAATT
TGTTCCTGGAGGTAAAAACGCGAGAGACAATTCCAAACGACGACGTCCTTCCAAGTATATTTTTAAAAATTGTGGAATAT
TCTTTCGTCGAAAGAATATTTTGAAAAACATGGAGGGAGTTATCCGTTTTGAGACTCTTGATGGAAAGACGATGAATGTG
GATGCAAAGGCCATACGTGAAAAGTCGCCTTATTTCGAAGCGCTTCTGAGCGAAAAATGGAGGGGAGAACAAGAAAAATT
GTTCGGCATTGGCCTTTGCCTGAAAGAAGCAAAGAGAATTTTCTCTTTCGCTCTCGATGGTTCTGGTGCTCTCAAAGAGA
AGGACAAGGAATCTTTTTCATATCTCTTTCCTGGCGTTCCGATGTTGAATGCGAAAGAGGTTCTTCCCTTTACGGAAGAC
ACGTGCAAGCTCCTATTTCCTGGCTCCCTTGTTGTGGACAGCGCATCTCCCGCACAAAAGTTTGGATATTACGAGTTTGG
AATGACTGGAGGGTACATCGTCTCCAAGCCCCATGAGGGGGAAGTCGACGTCAAAGACTTTGTTCTTGGGCTCGGAGACT
TTTCTGCAACAAAGGTGTGCTATGGGTACGCAGGAAAGTACAAATGTTCCGCTTCTGGATTTTTATTGTGGTGTCATGTA
GTCATTTCGAAGCAAGAAGAAATTGTGAGAGAGATGTACAAACAAACGGGTCTCGTTGTGGTCCCTGCACACCAGTTTTT
CCTACGTCTCTTCTGCGAAGAGTGTTCTTTGCAAAAAGGAGGAGAACTCGAGTTTTTCTCAGAGAGGAAAATGTTTTTTG

TTTGCTGCGACGACGTTCGTGTAGTACCGCAACACCTCGAGTTCTCAAAATACGACAACACAAAAAAGAAAGATTTACCC
TGGGAACACTCCAAAAAAGTCGAGACTTTGCGAGCCGGTGTTTCTTTCGACAAGGGATTTCTCCTAGGAGAAAATTTCCC
ACAAGAAGCCGAGGCTGCCTCCATAAGGTTTGAAAAAGAGTAAACTTTTGCTTCGAAACGAAGAGACTATAAAATTTCTG
AATAAATTTTATCATGTCTGTCTCATTCTTTTGGTCCGACGATAACCTCGCGTCGCTCATCGCGAAATATCTTCCACCAA
GAGACGTGCTTTCTCTTTCCCATACAAACAAACAGAACAAGAGAACATTTTCAAATGCCTTTTCTGGGTTACTGAAACAG
AGTTGGTGGACAAAAGGAGCCACAGAATTTTGGAAGATATTTGGTGGTCATGTTTCGCAAGAACTTCTCGAATATGTTTT
GGAAGGGGAAAGCGCGTTCTACATCACAAACATTCTTATCCAGAGGGTACTTTACCGTACAAACAGAGAATATTTCGACT
CTCTCGTCAAAAGGAAAGTAGTTGCCATCGTCAGAGACCGCATCAGAACAAAAGACAAAGACGAACTGTATCTTTGTGGG
CATTTGTTCCAAAACGGAGGAGAAGAAATTATGGGTGAGCTTATTCGTAGAGGGGTTCGCATAGAAAGATTTTGCGGAGA
TTGCCCCAGATAGAATTTTGTTTTGAACTCAAAACAAAATGCATTCCCCTCTGCTCCTCTCCCTTTCCGAGAGCCTGTCG
ATGATGTCGTCCATTTCCCTTGCGTTTTGTCTCTGAATGTCCATACAAATGCGTACAACCTCCTCGTCAGAAACTCCCCA
ACTCTCTATGAGCTCGATGTCCACCTTTCTGCCGTCTCTAAAACATTCTATCATGTAGTTTTTTGCCAAAATCTTTGGGT
CAGAGCCCTGCATTATTCAGGGGTGAAGGTTGCACTTTTTCTTTCTGAACAGACAATCAGAGCAATGAACCTCTGCGCAA
CAACACTCGCAAATCTCGTCCTTCAGTTTGACCGTATTGCACACCCAGCACTCGAAAACGATGTCTGGATATTTTTCTAG
CGCTTCCTTCAGTGCTCGTTGGAGTTTCTCCTCTGTTTCGACGATCTTGACTCGATAATTTCGTATTTTCCACTCGCAAT
CTTCTGGAGTGGACATGGTTATCTTTTTTTATTTCAAAAGATAAATTCTTTTAGTTCACGCTTTTCTCTGGCACTTTGAA
GCAAACAAAAGTTTCTGCCAAATGGATGCTGTCGTTTCCGATGGGGAAAGGAGAGTCTTTGTCTCGGTAAATTCCTCTCG
TCATGATGTAGAGTTTTTCATTGTTGTCAAAGAGAGCCTTTGGGAGAAACATGGCTACCTCTGCCAGAGAAGGTTTGAAA
AGGTACGCGTATTTGTGCATCGTGATGTATTTCCTGACTTTGAATCTCTCTGTCCTTGTACCTTCAGCAGAGCCGTCGTC
CACGAAAATGCCGCACTCGGAGAATTCTCCGAGGTAGTTTATGGGTGTTTTATCCCATAGAAACGTTTGGTTGAAAACGT
CTCCTTTTTCCGCCTCGAAACACATCCTGTTCTCGTCCACAAAACAAGGAAACATTTTGTTTGTCCTTTCGCGAAGTTCC
TCGACGTCCATCTGCGGAAGTTCTTGGTAATATTCTCCCATTCTTTTTTGTGTTTTGAAAAAGCTTTCCCTTTCGCAACA
GATAAATTTTCAAAATTTATCAATCGATTGAAGAAAGTTCCAATACATTTCTGTTTTTCGTATCACCAAACATGAAGATC
CGCGCAGTCCAATTCTCTGTTTCCTCTCCCGAAGAGATTATTTCCAACTCTTGTTGTGAAGTCACAAAGGCAGAGACGAA
ACTCGAGAAACCCGACAAGCTGAACAAGACGAAAGGAGCTCTGGGAAACCCCCAGGATGGAAGTCTCTACGACGAACGAC
TCGGAACTTTGGATAGACTTCGCGCTTGCAAAACTTGCGACCAGAACAACGTCCACTGTCCAGGTCATTTTGGCCACATA
GTTCTGAAGAAACCCATCATCCACCCTCATCATTCGAAGATTCTCAAGAACATCCTCGAGTGCATGTGTCTCTCTTGCTC
TCGTCTTCTCATCACGGAAGAAGCGTCAGAGACCCTCGGCATTCTCAGGCACAAGGGAAGCGACAGGCTAAAGGCTCTCA
ACGAGTATTGCAGCAGTGTTTCCGCTTGTTCGAACTGTGGACAGCAAACTCCCCACTTCTTCCTCGAAGGATATCATCTG
GAGGCTTGCTATACCGATGAAGGTTCAAAGAAGGGAAAGCCAGACAAGACAAAGTCAACAAGAATCCCGGCAGAGTCTCT
CATCACCAAGCTCAAAAAGCTGACGAACGAGGACTGTGTGATGCTCGGCTTGAACAACTTTCTCATCGACGACGTTGCGT
ACAAGAACAAGGAACTGTTCCCTGTCGACATGGGGCATCGCCATGCAACGCGTCCAGAGTGGTTCATCCTCACTGTTTTG
CCTGTTCTTCCTCCTGTGGACAGACCTCCTGTCATCATCAAAGGAAAGCAAAGAGAGGACGACATCACAGACGGGTACGT
TTCCATCATCAAAGCAAACATCGCTCTCACCAAGCACCTGAACGGCACGACGAAGAAGCGAATGAAGGAAACTACCAAAG
TCGCAAAGGTGAAAAAGGACCCATACGACGAACTCTGCGAAAAGGTGAGAATTCTCTTCGACAACAAGGAAAACAAGAAA
ACTGTGAATGGACAAGTCCCTCATGGGTTTTCTCAAGTTCTCAAAGGGAAGGACGGAAGGTTCCGAAACACAATCCTGGG
AAAGAGGTCAGACCACACCGCAAGAAGTGTCATCACACCAGAGCCTTCCTACCCCATCGATGTTATCGGTGTCCCCCGCT
CTATGGAGTCTGTGCTCACAGCCAGAAAGATGGTTTGTCGAAGCACTTTGAACGAATGCAATGACCTCATCGCTTCAGGA
AAGTGCAAATCCATCATCCGTAGGGGGAGACCTCTCGACGTTGCAAAGATTGTGGAGAGACTCGGTTCTTGCAAGCTGGA
ACCAGGAGACACTCTAATCATCCCTTTGCCAGAGAGGTATCCTGTCATGTTCAACAGACAACCGACACTCCGCGTTGAGG
GAATCATGTCTTTGAAAGTTCAGTATCACGACGATAAGACGTTCAAGTTCAATCCAAGCCTTTGCACTCCTTATGGAGGG
GACTACGACGGAGATAAACATCATTAATGTCTCCAACAGGCGGCTGCCCCATAAGTTGCGAAACTGCTTATGGGGGAAAA
CAGTGTAAAGTTTCGCGAGAGTTGCGCGGTGGACTCTCATATAACCGTCTAGTCTCTTTCTTCCACGAGCCAAAAAGAAG
GAAGAGGCAACATCCTCAAATTGCGGGAAACTCCTAAAGGTATTCGGTACAACTTTCAGGTAGAAATATTTGAAAGAAAC
TTCGCGTAATGGCGTTGGAGCTCCCTTTTCTTCCAGTACCAAGAGACAACATTTTGGAAACTTCGGAAGGAGTAGTCGTT
GAAATATATCGCATCACTTGCAAAACAACAGAAAAATTGTACATCGGCCAAACGGTCTCCCATGTTCTCAACCACAAAAA
ATGGAGAAGGTTTGGCTCACACAAGAGGTTTCTCTCTCATGTTTCCGAGGCTGTGAAAAACAACAAGCCTAAACAATGTC
CTTACCTTAACAACGCAATTAGGAAGTATGGTCAAGAAGACTTTGTTGTTGAGGTGATTGGGGTGTGTGAAAAGAAAGAC
GCTGACAGATACGAGAAAGCTGCGATTCAAAGTTACGGCTCGTTGTACCCAAACGGTTACAACATCAAGCTTGGAGGTCA
AAACTTTGAACACACAGAACAAAGCAGAGAAAAAGTATCATTGGGCGTCGAGGCGATGTATAAAATAAAAAGAGAAGAAA
AGTTCTTGAACGTTCACATCCTTTCACAATTCGACCCAGAAGACTTTCTCTATCCTCTTCGAAGAGATGGCAAAGTTTAT
GGGTGGAAGTTGATGATGGTGACACCTGAAAGAGAAGTCATAAAGACAGATTTTGGTGGTTCAAGAAGGCCAGAAACAGA
AGCAAGAGAAATGGCGATACAATTCATACGACAACTTCAAGAAAAGGAGGGCGATGTAAAAATCCGAATATATGCGGTCC
CAAGAAGGACCAAAATGGACAATCCGCAGCCAAGCACCCACACCTCGATCTGAGACGGGTGAAGGTTCAGAGACTAAAAG
GGGATGCGTCTTTTCAAAAAGGCGTAAGATATAGTCCACTCCAACCAGAGATGGTTCTTTGAGCATATGTCCTCCAAAAA
CATGGAGGGAGACACTCAAGGGCACGAAGACGCGAGAAGGAAATGTCTCGTCGAGTGTGGTAGGAAGGGAAATGAACATA
CATGTCCCCCAAGGGGAAGCGGAAAAGGCAGAAATGGAAGAGGTGCTGTTGACTCCGCACCACATCGTTTCAGGTCAGAA
CAATGCTCCCATCATCGGTCTCATTCAGGACGCTTTGATCGGAAGTTTTCTGATGACGAAGAAGGAAACGATGGTGGATT
GGGACACGTTCTGCGATTGTTGTATGTCGGCAGATGTCGGCAGCGTAGACATCTCTTTTGTCTGGGACACTTTGAAAAGA
GCGGAAAAACACTATCCAGAGTTTATCAAGGATGGAATGCCCATCGAAAGAAAAACATACCAACCTGTCTGCGAGAGCAA
GAAGTGGTTCAACTCGAAAGAAGAAGTTGTTCATCACAACAGGTGCAAAAACAAAAAGAGATGTCGCGTGGACAAGCAAG
TCACCAAGAGTTTGCCAGGAAGGGTTTTGTGGTCTGTTCTTTTGCCAGAGAGGTTTTGTTACGACAAGGAAGATGTTCAC
ATCGAAGATGGCGTCATCCTCCCAGAATCTGCGCCTTTGACAAGGGCTGACCTTGGGCCAAAGGCAGGAAGTTCCATCGT
ACATCTGCTTTGGTTGGAGAGGAGTGAGCAAGAGTGCGCGGATTTCATCCACAAAACCCAACAACTCACTCATCGCTGGT
ACACGCAGAGAAACTTGAGCATCGGAACAGAGGACTGCCAACTCACAAAGCAGGGAAAGAAAGAGATTGAAAAGGAGATG
GCGAGCGTCCGTGTGCGTTGCAAGGTTGAACTCGAGTCTGGAAAGACTGGAGATGACCTCGAGTCTTCCATCACGTCCAT
CCTCAACTCTGTGGTAAACTTTGGCCAGAAGTTGACAAAAGAGCACATTGCAGGCGGAGAGAGCAATGGATTTGCAGTCG
CCATCATTTCTGGTGCAAAGGGAGGGTTCATCAACTTGTCCCAGGCCCTTGCGATGGTCGGACAACAGAACGTTGAGGGA
GGTCGCATCAAAATGTACATCTCTGGTGAGAGAAGGTGCTTGCCTCATTTCGAGTACGAAGAGAATGGCCCAGAAGAGAG
AGGTTTCATCTCTTGTTCATACTATGAGGGAATGACTGCAATCCATGCCTTCTTTGCTGCCATGGGAGGAAGAGAAGGCA
TCATCGACACAGCAGTCGGCACAGCAAACTCTGGATATCTTCAGAGAAAGATTGGCCACAAAGTCCAGTCAAACACTGTC

AATCAGCTTCTCTCTGTGACTATGTGCGACGGCAAGGTCATCCAGTTCATCTATGGAGGAGACGGAATGAACGCCTCTCG
GTTGATGAACGTGAATGGCGGTCTCTCGTTTGTGGACCCCAGACGCTTTGTTCGAGAACTCTCTCTTCGCGAAGAGAAGC
TCGAAAAGCTTGGAGAGGAAGACATTGACTGGATTCTGGAACCCATCAAGCACAATGCCACCCCTGCGATGAAGAGGGTC
GCAAAGAACGTGAAGACTCTCCTCAAAACGCAACTGATGGGCCTTTTGTTCCCTTGCTTGGAGGAAAAGAAGAAACAGTT
GAGGGACAGACTCGAGAAAATGTTTGTCAAGGCGATCGCACCTCCTGGACACTCTGCCGGTTATGAGGCGACTTGCAGCA
TCGGTGAGGTTCAAACTCAGTTGACTCTCAACAGTTTCCGCCTGTCTGGCATCGGTGAGAAAGCAGTGCTGACTGGTGTT
CCGAAATTCAGAGAGTTGATGCTTGCTTCCAAGTCTCAAAAGCACAGCTCAGCCACTGTTTGCATCGAAGCTCTGGACTT
TGAGGTCGGCACAGACGAAGAAAAGAGAAAGGCACTCCACATTGTCGAAGAGCAGAGGAAGGTGTTTGAGCATCGAAGAG
TGTCTGATTTTGTGGAAAAGCACGAACTTCGGTACTTTTTCGGAAGGGAAGACGAGTTCGCTATTGGAGAGCACGCTTCA
GAGTTCACCGACCAGGAACTTCTCAAATATCAGCCGCAGTGGTGGGTGGATTTCTACCTTGGAATGACCAAACAGACACT
CGTTGGTTATGAAGAAAAGGGAGAAGATTGCGTTTGGGTGGTGGAACTCAAGGTCAAAAAGGAGATGCTCTACAAGTACC
GAATGACTCTGAAAGAACTCGCCATCGCTGTCTCTGATGGAGGGGATGTGGCATGTGTCCCATCGCCGACTTGTGAGTTG
ACGCTTCTTGTCTACCCCGACTATCAAAGGGACGTTTTCGGAGAACTCAGCAAGATCCGAGCTGACGAAAAAGTCAGTCC
CATCTTCAACGACCAAAACGTCAACTTTTTCTTTGCGAGAGATGTCGTGACTCCGCACATCTTGCAAAAGAGAGCGTGCG
GCGTAGAGGGAGTCGGCCGTATCTTCCCTCTTCATCTCAAGGGAGAAAAGAGGATGGTGATAGACACAGAGGGTTCAAAC
TTCAAACAAATTCTCAACACTCCTGGAGTCGTCCCAGAACAGACCACATCCGATGACCTTCATCAAGTTCTCGGTGTTTT
GGGTATCGAAGCAGCGCGATCTGTTCTTCTCAAAGAGTTCCAGAAAGTGATGAGCGGCGGCTCGTATGTGAACTCACGAC
ACATCCGCATCCTCGTCGACGCGATAACACAGAGCGGGAAATTCACACCCGCTTCTCGAGATGGCATCGAAAGGAGCGTC
GGGCCTTTGGAGATTGGAAGTTTCGAGAAAATGGTGGACAACTTCTTCACATCGGCAGCGTTTGGAGAAAAGGACGATAT
GAAGAGTGTCGCGGCAGGTTGCTTCATGGGAACGCCAGTCTTGAACGGAACAGCGACTGTCACTGCGCTGAGAAAAGATG
TGAAGCCCCCAGAGATTGCAAGCAGCGGCGCTTCTTTGCTCGAAAATCTGGTAGAGAACGTCGCGTCCGAAATCAAGAAG
CTTTTTGTTGTGCCTTGTCAGTGAGAGAATAAATATATTTTGTTGGATAAAAATATATACAAAGTATCAAAAACTTTTTT
GAGAAAGTGGATGGGAGAACGCAAGCATCGTTTTGAAAAAGTCAAAATGTTTGGCGAAAGAGAAAAGACATTCAACAACA
AAACACAAAGAATGGCGCGTTCTCCCAGAAAATGTTTGGAACAACGAGATAGTTTGGAAGAAATTTTGCTTGAACGCATC
AAAAACCATGAAACGCCTCCACCGTAGAGAGTCTATGAGAATTTTCGTTCCCTCTCAAAAAATATATTTTACTTCAAAAT
ATAAAAAGGAAAAACATCCAGAACTTTTGAGGAAATATTTTCATCACTCAAAATGACAGTCGCTTATCTTGTCCGCAGCG
GAAAAAATTGGGGACGCACCACTTATCAATTCGGAGTCACTTCAGATTTGGAAGAAACAAAGAAAAGTTGGGAGGAGGAT
GGAAACGTTCCAGAAATTCTCGCTGAGGCTTTCGTTGGCAACTACAAGGACGCCATTGAAAAGCTAGAAAGGACGTTCTC
TGAACATTTTGGCGAGTGCACCTACGGGTTTTTCTACCCCAACTCTGAAGATGTCTGGGAGATAGTCTCCCTTTTTGAAT
TTGCTCTCGGCGTCACCGTCCCAAAGAAACATCTGTATGACGGAACAGGAAGTTCTCTACGCCCCGAAACGTCGTGGTCA
GCACTCTGTGTACGAGATGCGTGGTATCGTGAGCGGCCCTGTGCAGGTGCGCGCCATCCGCGAAGCTCGAGTATAAAAAC
ATATTTTTGTTCAACAAAAATATACGACCCGCGGAATATTTGCTCTTGCCCTTTTGAACGACCTTTGCACATTGAAGCGA
ACCGCTCTGCCGAGAAACCAGACTTGGGTTGAAAAAGGACGCTGTTTCCCGACCACAAACTTGTCTGTTTTGGAGACAGG
AGGAATATGGATGCGTTATACCTTTGAAATATCTTTTTACAAATCTGGGCACGTTGGAGCTACAAAAGATATGTTGTTCA
GCGCTTCCTCGCCTTTGCGAAGCCGAGCCTTAGTTGGTCATCACAAACTATTCTCGTTTCAAAGTAAAGCTCGTCCTTCC
CTTTGAGCGAGACACAAAGAATGTTGTCTAACAGGTCGAGAGAATATGTGTTGTTTGTGGTTTCGTCTTCCCATGAAAAA
CTCGAATTCTCTTCGTTCACGATATTTTGGCAAAAGTTGTAATACTCTTCCTGTTCTTTGTCGTCGAAAAGAAAGGGAAG
GCAGATCGTCGAGCCGTTCAGGTTAAAGTGAAGCACGTGCTTTGGCATTTTTCTTTTGGATATTTTTATCGTTAAAAAAT
ATCATGTACAAAAAGCGCACTTCCTGGTTCTTGTACACTGTATTCTGAGGTGTTCCGCATATTTTTCCGTATACTCACAA
TCGGAACATTTTGGAAGATTGCACCAGCAAAGAGGGTAAATGAGGTCTTTGCTTAAGAGCTGAAAGCGAAAGCTCACTAT
CGTTCCGTCGTCTCTGCGTTCTATGAGGGGGTTCACGTCGAGATTTTTCCAATGTTCCGCCCAATAAGCGAGAAGGTGCT
CTTTTTGTCTTTCGTTCATTTTATATTTTGTGGTAAAATATAACCTTTAGAACAAAGAATCTGGCATGTCTTTTTGGTGC
TCTGGGCAACAGTGAGCAAATAGCTCGCAGTCGACGCTCCCTCCAGAGTCGCTGTCTTCCACCAGGTAATAGACAAAAAG
AGAGTCTCTGTTGTGGCAGTGAAAAACATCGCACACAAGTTTTCCGCCTCTCTCGCTGGTGTACTTTTTTGAAGTGACGA
AAGACCTCATGTTCATCACTTTTTTAAAGTGGTTGTTTGGAACGACTATGAAACTCTCGAAGAGTGCTTTGAGCACAACA
GTTCACGATGGAGAGAGGAAAAATGGACACCAGAAAACGAGAGGCAATTTAGAGAGTGCACGCGCGCCATCAAGGACTCA
GAGTACCTGTGGAATGAGGTGAAAAAATATCAAGAGACCTGTTGCACCAAAGAGAGCCATCCAGACGACATCAAGTATTG
TCGGAGGCTCACAAACATTTTGAAGAACGAGGCAGAAAACAGAGAATGGAGAGAGGGGAAGAGGAGAATGATGAGCTAGT
CGAGGATTTTATCTTTTTATGAAAAGATAATTTATTGTGGAGGGAATCAACCGTTTGAACTTGCAATCTGTTTTTGTTGT
CAGACTGAGAGACAATAAGCTTGTTTTTTACATACGATGAACATCAAACATCAAGTTTTTGGAAACACTTCTCCTTCTCG
AAGAACTCTCTACTTTGTTGTGAAAAAGACGGAAGGGAACATTGCCAAAATCTGCCTCTTCCCCGCCCATGTCAAGAAAT
TTTCTGGAGACACTTGGTCTTGTGGGCCGGATTGGGAACGTTTTGACGAGGCACTTCAAAGCAACAAGTACATCAAAGCA
AACATTGGCTGGGAAGACGACATTCCTTTTCTCGAAGTGAAAGGACATCTTCTCTTGTTGTGCGGAAGAGAAGACGAACG
AGACCTGTTCAATCCACAATAAAATATTTTCTCCAAAATATTTTTAAGAGAAGAGATGTTTTCTTTTCTCCCACAAGTAA
CATGACAACAAATTTCGAAACTCGGAGGGACCTCACCGTTTCGAAAAATTTCGTCGTCCCTGTTGTGCCTTCTCTCGCGG
CTGCAAACATCGCAACACCCGGTTCCATCGTTTTTGACAAGTCAACGGGGAATTTGAACGTCTCTTCAGGTTCTGGTTGG
AGTCCTTCAAATCTCCCCATCGCGACTCCGACGGTGAGAGGTATCGTCTTTGGTTCGACAAACACAGGATCAGGAAGCAC
AACCTCCCTCGGATACCTCTCTGGAACAGCGGCAAGCAGCTCTGTGTTTGTTGGGTATTCGGCAGGTTCTTCTGTTGCGG
CTCTGATCACGGATTGTACGGCTGTCGGCTTCTCGGCCATGTCGACACCCGGAGTTCTTGATAGGTCAACAGCCGTCGGC
GCAAGCTCTGGAAACATCGCAGGGTTGGAAAACACTTCTGTCGGAGCCAATGCCGCCAGCGGTTCAACAGGAAATTCAAA
CGTCTGTTTTGGCTACAATTCAGGCATAAACATGACAGGTTCTGAGAACGTGATATTCGGAACGCAGGCGGCCGACGGAG
GAATCTCTGGGCCTTTCAGCGACAATGTGGTCGTTGGCTTCCAAGCGGGAAGAGCCGCTACCACTGCCTCTGGAAACGTC
GTTGTTGGACAAGCTGCTTGTCCAAATCTCGGGACCTGTACAAACTGCATCGTCGTCGGAAGGGCAGCGACAGCAGGAAC
TTCAGGAACAAACAGAATAGTTCTGGGAGCTTCTGCCGTCGGAGCTACGGATAATGAGCTGACCATCGCTCCCACAATCA
CACAATGGAGAAGTCTCGGTCTTTCATCAGCGGCAGCGGCGAACACGCTTCAGATTAATCCCGCGACAGGCATCATCACA
CAGGCGGCTTCATCCAGAAGATTCAAAGAAAACATTCGCGATCTCGAAGTCGACACAAGCAAACTTTACGAGCTCGCTCT
TAAAACGTACGAATACAAGACGGACGGGAGGGAAGATTACGGTCTTATCGCTGAAGACACCCACGAAATCCTTCCAGAGA
TTGTCACTCTCGACGCAGAGGGAAATCCTCACGGCATCAAACATTTGACCCTCGTGATGCTCCTCGTTGCCGAACTTCAA
AACCTCAAGAAAAGAGTAGAAGCACTCGAATAAATATTTTTGTTCTAAAAAATATTTACATGTCATCGAACGCGCTCTCT
CCGCACTCGCAAAAACTTTTGGGAAGGCCGTGCCCTTCCAGCCATGGAAGTATCAGGTCCCTTGTCAAGAAAAGGCAATC
ACAAAACATCGTCATAAAAATTTCTTGGACCTCTTTGTTTTGCGAGAGGACGTCTCTGTGCCTGCAAACGATAACCCTTC

CAGCAACTTCCGACACAAAATCCGAAAGGGAACCTTGCTTTGTCTTTTTAAAGTAGAGTTTGTACCATTCCGTTTTGTCG
CTCAACGACAGGACTTCTTTTCTCGTAAAGTTCAAAAAACGCTCCTCATCTTCCATTGTTGCTTTTCCGAGAAAGGGTGT
TTATTCCAAATTTAACGACATGAATATTTGTGGAAGTTTCAAACCATTATTTCCTTCTGAGACGAAACGACTTTTTATGC
GCGAAATTTGTGGAATATTTTATCCTTTGAAATATGTCTCTCGCTTCCAACATCATTCAGCACAACGGAGAATTCGACCT
GGTGTCCTTCACCTATAGAATGGAAGGGGCAAGCATCGAGTTTCTTTGCGACTTTGGAGACAAGCAGACGTCAAACAGGA
AACTCTCCAAACTTTTGGCAAAGGTCCTGAGCGGCACAACGTATACAATCTCTTCAAACGGCAACGATGGTGAAGTAGAG
ATTGAACACGACGGAGGAAGGATAAACTTCTCTGTCAAAAGCTACGGAAGCGTCAGAGGTTGTCTCAGCGTTTCCCTTGC
GGCAGAAGCTTGCATCTTGGCCCTTGAGAATGCCATTAGCGAGACATCATAAATATTTTTGAAATAAAAATATTTTTAAA
AAGTCTCTTCACAAAATATTTCGTATGGATGCTCTTCGAACGCACACGGCGAGTTCTGAACTTCCCACAGTCCACAGAGA
AAACATCGGGCTGTTCTTTAAAATTCAAGGATAAAAGCACCCTTTCAAACCGGACTTTTTCTTTCATAAAATTTGTTCTT
GCTCGTTCCCCCTAATTTTCATGGAAAAAAAGAGCACATTGGATGAAACAACAAGGTCTTGAGATAAAATATTCTTTGTA
ATATTTTTTTGTAATGAGCAGCACATCAAACTCTACCATAAAAAAGGAGTTTGTAGTTTCTCGAAACCTTGTCATTCCCG
TCTACAAAAATCTCGCGGCAGCTCCAGAGCCCGTTGTTGGGTCTATCGCGTACAACGCAGCGACGCAAGGCGTCATCTCG
TCAAACGGCCTTTCTTGGTCTTCTCCTTCCGCAAGTGCAGCAACGCCGACAGCGAGAGGCATCGTCTTTGGTTTGACTTC
CGTTTCTCCAACGACCACAACAAGCTACGGAAACGGAAGCGGAACCGCTCCCAGCGGCAACATCTTCGTAGGATGGAGGT
CAGGATTTAGCAACAACGGTGCGCAGACAGGCATCACAGCCGTCGGGCATCTCTCGTGTTCTCAGGCACAGACAGTGACG
AACAAAACGATGGTCGGAAGGTCTGCCGGAAATCAGGGAGCTGCACAGCAAAATTCCACAGGAGTCGGAAGCACAGTGTT
TACGTCGGGCGCATCTGGTTCAAACAACTGCGCTGCTGGCCAAAGCAGCCAGAGCGTCAACGTCGGCTCAGGAAACTCGT
CGATGGGAAACGCCTCTCTTGGAGGGACTGGGGCGCCCACGTACAACAACAGTGTAGGGATAGGTTATCTCTGTTTGTCA
GGAGGAACTGCCTCTTCTGGCATCGTCAACATCGGAGCGGCTCCGGGAGGGGCTTTTTGGAATCCAGGCTCTTCCACAGA
CGTTGTTTTCATCGGCAACGGGACGAGTCTCTCTGCAAATATCACAAACGTGGTTGCTTTGGGGAGAGGAAACTTTTCTG
GCGTCACTGCAAACAACACGCTCGCGATCGCAGACAACGTCACGCAATGGAGGTCTCTCGGTCTCGCAGCTTCCGCTTCC
GCCAACGTTTTGCAATTCGACCCTGTCACCGGTTTGGTAACACAGGCTGCTTCGTCCCAAAAATTCAAACAAAATATTCA
AACTCCTTTGGAAGGTGAAAGTTCCGACATTTTGGATATGAAAGTTTGTACATACGAAATAGATGGTCAAACTGACCACG
GAGTAATCTCAGAGGAAATTCCGGAAAAGTACGCCACCTTTGACGTCAAAGGGCAAAGGAACGGCGTCAAGATGTTGAGG
GTCATCATGTCCCTTCTTTCAGAGATCCAGGTGTTGAACGAACAACTTTCTCTCGTTGAGGAGAACGCATAAAAGAAGAG
CATTTTCTTTTCTCTCAAAAGAAAATTCAGAAGTAATATGACCACAAACTTTGAAACTCGCAGGGAGCTTGTCGTCTCGA
AAAAGTTTGTCATCCCTGTCGTTCCTTCTTTGGCCGCTGCATCTGACGCGACAGCAGGTTCCATCGTTTTCGACTCTTCT
TCCGGGAACATGAACGTTTCCACAGGAGAGGCATGGTCGGCTTCTGGCCTTCCAGCCGCGACTGGCACTGTTCGAGGTCT
CGTTTTCGGTGTGACAGATTCCAACATCGGAGAGACGACTTCTCTTGGATACTTGTCAAACAGCGCTGGTTTCGGTTCGA
CCTTTGTTGGCTACGCGTCTGGAGACGGTGCTGGTGGCGTCAGCGATTGCACTGCTGTGGGTCATTCATCCATGTCAATC
GCTGGAATCGGCCCTCAGTGTGTGGCTGTGGGTGTCAACGCTGGAAGGGCGGTAGGTTCACAAAACATCTCTGTCGGTGT
CAATGCGCTCAACGGAGCGACAGGAAGCAACAACATCTGCTTTGGCTTGGGTGCGGGCCAGGGCATGGCAGGCTCTGGAA
ATATTATTTATGGAGCGAATGCAGGAGACGGAATTGTGGGAAGCGTCACGAACAACATCGTCGTCGGCTTCCAAGCAGGA
AGGGCAGGAACGACGGCGTCTGGAAACATCGCCATCGGACCAAACGCTTCTCCGGATCTCGGAACGACAACAAACTGTAT
CAGCATCGGCTCTGCCGCGACTTGCGGAACTTCAGGAACTGGCCGCATCGCTCTCGGAACTTCTGCCGCTTGTGGTGTCG
ACAATGAAATGACCATCGCTCCCACAATCACACAATGGAGAAGTCTTGGTCTCGCTGCTGCCGCTGCCGCAAACACTTTG
CAAATTAATCCCGCGACGGGTATCATCACACAGGCGGCTTCATCCAGAAGATTCAAAGAAAACATTCGCGATCTCGAAGT
CGACACAGAGAAACTCCACGAGCTTTCTCTCAAAACGTACAACTACAAATCAGATGGGAAAAAGGACCATGGTCTCATCG
CGGAAGAGACGTTTGACATCATTCCGGATATCGTGACGCTCGACGCAGAGGACAAACCGCACGGCATCAAACATTTGACT
CTCACGATGCTGTTGCTCGCCGAATTACAAAGTCTCAAAGCTCAGGTTCAAGAGCTCCAATAAAATATTTTCTCTGATAA
ATATTTTTAATGAGTAGCACATCAAACTCTGTCATCAAAAAGGAGTTTGTCGTTTCAAAGAGATTGGTGGTTCCGGTTCT
TGCAAACGCAGCAGCAGCCACCGTTGCGACGCCAGGTTCCATTGCGTTCGACAACACAACACAGTCTCTCGTCGTTTCTG
GCGGTTCAAGCTGGGTCACGCCGACTGCCGCAGCCGCAACACCGACGGTAAGAGGAACTGTTTTCGGCACCACACAAAGT
GTCTCTAATGCGTCTGTCGGTCTCGGGAGCAACATCGGCGTCGGCACTGGAACAAACACTTTTATCGGAAACAGAGCGGG
AGTGGGAAATGCCATCGGCCAAACTTCATTGACGGGTGTGGGAGAACAAGCGTTTTTTTCGACTCAGAACGTGTTGAACA
AAACTGTCGTCGGAAAGAGCGGTGGAATCTCGGGGTCGACTGGAGCAAACTCGACGGGAATAGGAAATTCCGTCTTCCGT
ATCATGACTTCAGGTTCGAACAACAACTGCGCTGTTGGCACGGAAGCTCAGTCGACCACAACGGGTTCGGGAAATTGTTC
TATTGGAGACCTTACGATGAGCCCCGCTTCGACGTCGAATTCGACAAACAGTGTCGCCATCGGCCACTCACGTCTTGCCT
CTGTGTTGACCACGACCGCATCTTCCGTCATCAACATCGGCTCTGGAACAGTTTGGTCTCCAGGAACGACAGGCTCAATA
GGTGCCACGTTCATCGGAAACGGAGGAACTCTAACAAACAACACAACGGGAACTTTCGCCCTTGGAAGTGGCGGTTTTGC
AGGCACAACTCCATCACAAACGTTTGCAGTTTCAGACAACATCACACAATGGAGGTCTTTGGGTCTTGCAGTTTCCGCCT
CTGCAAACGTTCTCCAATTCGACCCAGCCACAGGTCTCATCACACAGGCAGCTTCGTCCCAGAGATTCAAAGAGAACATC
GGAGACGCTGAAGATTTTTCGAGCGATATCCTGGAACTCAAAGTTTGTTCTTATGAAATAGATGGTCAAACTGACCACGG
AGTCATTGCGGAAGAAGTTCCTGAATTTTTTGCCACCTTTGACGCAGAAGGGCAAAGGAACGGCGTGAAGCATCTCCTCT
TCACGATGGCCATTCTCTCAGAGATTCAGAAACTTCGAGAGCAAGTAAAGTTTTTGAGAGAACAAAGAGGCATCTAAAAT
ATTTTTATAAAAATATTTGGTATACACGAGACATCAGAAAGTTCATACATTTCCTGTCTTTCTCTGCGAGCGCTTCTTTT
GGGGGCAGTGACTCCATCGGCGCTTCAAAAACTTGAAATTTCTTTTTGTCCGTGAAGAGAACAACAAGTAGCCAAAGAAA
ATTCACGGGTCATCCCAACAACAAAGAGCACAAAAGTTCAAAAGATCGCCGAGACTCAAGAACGAAAAAACGTGCTGGGT
GATTTCGTTGGGGAGGTACTGCATTTTGAAGCGAAAGAAGAATTTTCAAAGCCCTTTGGAAAGTTCGATGTTTTAAATAT
TTTTAAGAAATTCTCTGAGGTCTGTCTATAGATACGCCGATGATGACGATGGTGAAAACAGAACACAGAAACCAAAGAAG
GGCGCCAGCCAAGAACAAAATAGCAAGTTCAAAATCTTGCATTTTGTAATGTCTAGAAATAGTCAGATACTGCAAGGGCT
TCAGATCCAAAAATCCTTGATTCTTCCTTTCACGCCCGTACCGAGGAACGCCTCAACAGGAAACATTTTCGTCCAACAAA
ACACAGGAGACCTTTACGTTCGAGATTCGAACGCGCAAACTCTTGTTGTCGGCGGCGGAAGTGTGCCATTCGACCCTTCG
TATTACATCTCCACAACAGGTTCTGACTTGAACAACGGCTCCCTCGCCTCTCCCTTTGCGACTTTTCGAAAGGCATTTGA
AACGGCCACGAGAAGAGGTTGGATGAACACATGTTCCATTCGTTTCCTTCCCGGAACGTTTCAGATCGGGGATGGAGCGA
CCCTGAGCTATGCGGTTTCAGGGGGTACAAAAACCGGTGCTTTGACCATCTATGGAAGTTCGACACAGTTGGTCGCTCCA
GTCGCGGGAGCGGGAGCGGTTTTCAATCCGGCGCTCATTCAAACGGACATGACATACACTCCTGGCGGGTTGACCCCTGG
AGCGCTCACAGGAAAGTTTTTGCATTTCCTTCCCACATCCTCAGCATATTTTGCAGACACATTTCACCCCATCGTCGACA
ACACAGCGGGAATCATCAGCGTCTCTTCTCTTTTCTCCTTGCCCGTGACGGCAGCAGATACTTTCGAAGTCGTCGAAAGC
ACAACACAGCTGATAACCGACCAAGCGCTCCTTCTTGTGACGGTAGACAGACCTATCATTTTCAGGATGCTCAAGATAGA

CTCTGGTTCTGCGAGCTTCAACTCCACCATAAGTTTGATAAATTTCCAGGCATGCTATCTTTCAAGCACAAATCCAGCGA
CAGAAGGCTTCAACTTTAGCACATCGAGGGTGGTGTTCACAGACATCGTTCGTCTTGTTCTTGATCCGTCCAATCAAATG
TTTGCGAACGGTTCGGTGCTTGATGGGGCGATAGTAAACCACCAGTTTTCGCAGCTCTCGATACATTGCGGGAATTTTGT
GATAAATTCTGTAATATCTTTCGAAGCTTCGAAGGTTGTGATCCTTGTGTTGCATGGTCAAAATTCGGAAGTTTTGACTG
CAAGTTCTACCGGAATCATCACACTGGCAAGTTTCATCGGGCAACGACCGGGTCAGCCTCAACTTTTCATCAGCGGTTCG
GCGGCGGTTAACATCTCACAGGTCGCTTTTTCGAGCACTCCTGGCATTGGTCTTCTCGGCCAAAATGCGAGAATAAACGT
CGAGAGTTGCAGTTTCGACACGTGTGGCTTCGGAATCTTTGGTTCTCAGTCGGACCTGTTCGTCACAAACAGCAGTTTCA
ATGCTTGTACGTCACCCATGTCTTTTGAAAGGGCATGCAGGGTCTCGTTGGATAATATTTCTGGAACGAACGCGGGCTCA
ACAGTGTTAACACTTGCAAGCGGAAGCAATGGAGTGGCAACAAGTTCTGTGACTATTTCGGGACTAAACGACTTCAAAAT
TGGCTCCAACGCTGCCGTTGCTTCCCCAGGTTGGCCGTCTTTGGTCGGCGGAGGGTCCGCGCTTCTGTCTGACTATGGCG
CTTTGAGCCCACAATTTTGCACATTGCAAGCCGTCTAAAAATCTTTTTGTTCCATGGCTCCAGAATATTTCTTTCGCAAA
AGAAATATTATTTCACTGGCCCTTCTCCACCAAAAATCCATCCGGATTTTCAAAATACAGCAGGTCGTCTGGCATGTCTC
CTTGATGTTCTTTGCAACAATGGTAGAAGCGGTTCTCGACTTTGTGAAATCCCATTACGCTCGGAAGGTGCAGAACGTTT
GATCTGTGTTCTTACAATAAAACCCTTGACGAGACGTCTCTTCCTCGTAGATGGCGAGAATTTTTCTACCCTTTCCCGTA
AGAGTAGATTGTCTCTGAGCCGTTTTCCTGTGTGTGGACATGGTAGTTTCTGTCTCCGATATATCGCACTCTGCGAGAAT
GGCCGTTCCAATCCTTTCGAAAACCTTTGCTGTCGTGCTTCCAGATTCCGCGACACGTTCCCCGTTTTTGTAAGTCCCGG
ATTCGACGAATCCGTTCTCGGAAACTGTCATATGAAAACCGGAAAGAACTCCGTGTGAATAGGTGAAAGCTGTGGCTTTT
TCTCCAAAGAAAGTAAGGCAAAAGCCGTGAAGCACTCCAGCAGGAGAGATATGAAAACACCCTTCTTCGATAGACAGAGG
AAGAGTTCCATACTTTTTGCGGACGAAGCGCATGTCTTGTTCGCGAATGAGCTGATGATAGGTCGAACACGTTTGCGCAA
AAGCGTTGATGTGCTTGACCAAAGGGAGGAACGAAACGATGTGAGAACCAATCTCGAGAGGAAGATGTTGAAACTCCATA
TTTTTTGCGAGTGAAAGAAAAATTGTTCTCCCGAAGGGTCATTTTCTCTTGCAAAATATATTTGAGATGAACGAGCCACA
AAAAGAGACTGTTGTTCGGATGTTGGCAAAGGCTTGGTCCATCCACAATATTCCTCCTTTTGTCGAAAGGGGAGAAGACG
GGAAAATCTTAAGAATCCGTTTCTTGAGGAACGACAAGGATTTGATACATCCTCTTTGTTGGTGCTTTCAAGAAGGGTGC
GAGAACTGCGAAAAGACGAGGGAACGAGAAGAGAACGAAATGTTCAGATGCAGAATGACGTGCTCTGAAATAAAAAAGTG
CTATCTTTGTGCCTGATAAAATATTTTTAGATATTTTATAGTTGAATGCCGTGCTGCTGAATGATATCGCGAAGAAATTC
GAGTTGTTGCCTTTGTTTTTCAAAGTCTTCTTTTTCTTTTTGAACCTCTTCCTGTCTCCTTTTGCGGTACTCTGTCTGCT
TTTTTAGGATGTCATCTTTGTGTCGTTTATAAGAATCTCTGGCAGTTTGTCTCACTTTTTCTCTGTTCTTTTCTCTGTAC
CTTTTTGCGGCTTCGAGATGGGCCTTTTTGGCCTTCTCCTCTTTTTCAGAGGACATGTTCATCATTGTATTACTTCAGGG
ATGTAATACAATTTTTTATCATCAAAGAAACTTTGATGATGATATTTAAAGGAAAAGAAACGAGCCCGTAATGGAACGTC
CCGGACAAGAGATTATTCCATCACAGACTAACGATACCTTCTTATCCCTTTATACTTTTTGCGAGCCCAAAACTTTCTCT
GATAGAACATGCCTTATTTGCCAAGAGAAGTTTTCCTCAGACGAACAAGTCAAGAAACATTTTCGTTCAAAAGAACACAT
CCAGAATTTCTCCTTGCAAAACAAACTCCCTCATCAAACGGCGGAATGGTGGGATTTGTTCGAAGGAGAGAACGAAATTC
AACATTTTTTGAGGATGGATATCGCAACGACGATCGGTCCTCTCCCTTCGTATATCAAGTGCATCTTTGCAAAAGGAATA
TTATTCGGGAATCTCTGGGAAGTCATTCCCAGAGATTTTTGACAATGGGAATTCCGTAATCTTTGATATTCTCAAGCAAA
AGGTTTTTCCAATTTATCCTGTCGTTCCATACATCTTCCTGCAAAATACGAAGAACAGAATATCCGTTTTTGATAGCACA
TTCTTCCTTATATCTGTCTTTCTTTTGTTGTTGTTCCGGGGTCTTCCAATTCGATACTTGCTTGTAGTGCTGAATACCAT
CCAATTCAATAATTGTTTTTGAAACACAAAAGTCAAAAGGAAGAAAAGTGTTCTTTTCTGGGTTCTTGCACCACGAAGTT
CTGAACTGATGAACCGGTTCAACAAATTGTTCTTCGAGAAAAGAGAAGAGCTTCGCTTCGGTCTTGTTTTTGCACTTGGG
ACACCAACTACCGGAAGAGATGTGGTGGAGAGAAGAGCAGAATTTGTGTTTCTTTTCGCATTCGAACCAAAACTTTTTAC
GTGAACTTGCAAACACCTCCCTTGGACTTTTCTCGTTCCTTTCGGTGCTCCAAAACCTTGCCTTGTCGGAGGAAGCGAAG
CTCTTTTCAAAGCACATATCGCAATCACTTGAAGAGCATAGTTTTTGGTTCGAACAAAAGGGGCAAAACGTCCCGTTCGA
GACGCTGTCCAATCTTGTTTCGAAGCCGTGTTCGCACGTCCCGCATCGAAACCAAAACTTTTTGTTAGAACTTGCAAACA
CCTCCCTTGGACTTTTTTTGTTCTTTTCGGTGCTCCAAAACCTTGCCTTGTCGGAGGAAGCGAAGCTCTTTTCAAAGCAT
GTTCCGCATTCATTTGAAGGACAGAGTCTTCTGTTCGAACAAAAGGGGCAAAACCGCCCATCAAGAATGTTATTTAATGT
CGCTTCAAAACTGTGTTTGCATTCGCACCTAAACCAAAACTTTTTATTTGAGCCCAGGAACACTTCTCTGGGACTTTTCT
CATTCCTTTCGACACTCCAAAATTTTGCCTTGCTGGAAGAAGCAAAGCTTTTTTCAAAACACGTTTCACATTCGTCCAAA
GAGCAGAGTTGTTTGTTCGAGCAAAAGGGGCAAAAACTCCCATTCGAGACGTTGTTCAAAATAGCTTCAAAACTATGTTT
GCATGTTCCGCATTCGAACCAAAACTTTTTATTGCTGGTCCTCGGTATCAAGAAAGGGCTTCCATACTCTTTTTTCAGAT
ACTTTGACTTTTCATGCGAAGCGAAACTTCGTTTAAAACAAAACTCGCATTCCTCTTTTCCACAGAGTCTGCTCCTTCCT
TTTTTTGTTCCACAATTCATCTCGTTTCTTCTCTTCGTATTTTCCAACTCAAAAACATTGTTCTTCTTTGGTTTTCGAAA
ACACAACAAATATTTCTTTTGCGAAAGAAATATATGGAAAGCACGCTCCAGGATGATACGTCATGTAGATACCGGATTTG
CAACTCTGTTTGGTTCTTGAGTTGGTCTCCTTCGAAAGAAGAGTCGACGAAGGCAAGAACGTCGAGAGGAGAACAGAAGC
TCAAAGGACCACAATATTACGCTCATCTCTTTGCGAGAGGAAGGTCTGAATTCTTTGCATGTTGCCAACTTCCTTGGTTT
GGAGGAGCGAGAGGTTTTGGAAGTTTAATCTCAAGGAAGAAAAAGACCATTACCACAGAAATATATTTCTGTGGGTATCT
TTACCGTTAAGTTTCGGGTGAAGGAAGACCCACCAAAATGTGTTTTTGACACTTTTCTATATATACGGGTCTTCCTTCAC
CCTCGGGAATGTGGATGGACTATCCATAAAATATCAAAAATATTTTATTGGTTCATCTCGACCAAAGGAATAAAAATTTC
TCCATCGCTTTGTATTTCTTCGCCAAATCTCCAGGTGGCACAAAATTTGACAGAAAGAGAGAAATTTTTCTTTCTGCTCG
TCTTTTTGATGGTGAACCCGAATAAATATCTGAGCTGTCCGTTCAGCCATCTCAGGAAAAATTCTGGCTTTGCTGGTATC
GTCCCAAAGAGGCTGACGAATATCTCTTTGTCTTGGTTCATCCTTCCAAGCGCACACTCTTTTTGATATATTTTTAACAG
GAAAACACACAAAGCGCGAAAATTGTCGGTCCGTAAAACATATGGATGATTGACCCACAAGTTCCGAATATCTGACAAAA
AGTAACGAAAATTTGCCTATTCGTAAAACATATGGATGATTGACCCACAAGTTCTGAATATCTGATGTTCTCGTATTTTT
TGACATATTTTTTCTTCCACCCTCTTTCAAATTTTTTTCGAGTGAAATTTGAAGTTTTGTCAGATATTCGGAACACATGG
ATGAGTGAACCACAAGTTCTCTGTTCTCATGTTTTTCATTGTCTTTTTTGTCAGATATTCGGAACTTGTGGTTCAATCAT
CCACATGTTTCCACATCAACACAAGTTTCCCATTTTTCCTTTGCCTCTTCTTTTCGAAGATTTTGCCAGCCGTTTTTCCA
AACACGTTCGCATTTGGAATGTTCTTGTACCCGTTAGCCTGTGCCCAAGTCACAAAATCTTGATATACTTCCGAAGCAGA
GAACCATCCATCTTCATCTTTGTCGTATTCCTCTGCTGAGAAGTCTTCCAAAAAGAGCTGAGAAACGTTCTTTGAGTTGT
TTCGACATTCCTTTTTCAAAGAGGTCTCTGGAATGTTCTTGATGTCCACTCCCTTCCAGTCAACAAGGAATTTGATAAAA
TGTCTCGCTCTTTTGTCTGAACAACGGAACATCGCATATCTCCTGTCCGACTGTTCGATCTTGACTGAAAAGGTGTTATT
TGTGGTCAAAAGAAAGTTGACGAAACTCTCCTCTTCGGTTGTTTCAACTCCCTTTCGCTCAATCTGGACGGTCTTTTCCG
TGATAAGATGTTTTATTACGTCAAAGGACTTGTGATATGCTCCAGCGTCAACCTGGTTCATTTCATCCAGAACGACCAAA
AGTTTCTTGTCGAGGACAGAGTTGAAACGATGAACCACCCTCTCGATGTCTCCGATGCATTTGCCAAGTTTTCTCCCAAA

GACTTTGTCCAATAAAAAGTCTGTAATTATTCCCTTTCCTGCGCCTTGGGCTTCACTGAGAATGACAAGGGCGACACCAG
TTTTTTCTCTTGGATTTTTGATGATATGCGACAGCCATGAAAGGATATATTCGTAATTTTCTTTTGAACTGTCCGCCCAA
ACTCCGAATAGATGGTCGAGAAGAGGTTGTATTTTCTCTCTGTCGACCCCTTCGTCGACAGGTTGAGCTTTGAAACCCTC
GAATGTGTTGATGTACTCGACAGGCAGACGGGAGTCATCTTCTTCTGGAGAAAAAGGGAAGAAACAGAGTCCGTGAACGG
TGAAGCTGAGATGATTCTCTTGGAAGAAACTAGCCAGTGAAATGGGGAATAGTTCTTTGGCGCCTTCCACTTCAAGAAAG
AATTTCATCGCTGCGCTTTTGACGAATGAACTTTTGTGAATTTTGAAACATTCATCCTTGTTTGCTTTGACGACAACGTT
TCCGCTCCCAGGCACATAAGCAAAAACACGCCTCATATTCGGGATGATATCGTCCCTCATCTCCTCTTTGTTCTTGTAGA
CTTTCCCAGAGAATTCCATCGAAAAATTTGCCCAATGCCAACGGTCGCTCGGGTCGATGAGACAAGGTAAAAACCCATTT
CTCGCTTTTTTCTTCCTTCCAACAAGAAGGATTCTTCTTTGTTCTCCGTTGTTGTTTGATGAACGACATTTGAGCCAAGT
GTTTCTTGGTCCCATCGAAATATAGGGAGAGTTGAGGTTCGAACTGTTATCGTGCTCTCTGTCGCAAATGGGACAATGCC
AAGGACAAACCATTTGAAAGTTGAGGAAAAAGTTGCTGTTTGTCTGTGTCATTCCCTTGTACTCGAGGCTTTCTTCGGCT
CCGGGAAGTTTGTAGAGTGTTCGCAAAGCTTTCGCCTCCCGTTCATCGGAAGGAGGAGACACGTCCTGTGTCATGCATAT
CCTGGGAATGGAAGAATCTGCCAAAGAAGGGAGTCTTTCACACCCCTCGACATCACAAACAAACAGTTTTGCATCTTTGG
TGAGTTTTCTCTTGATGGATTCCATACCCTTCCCTTTCTTCATGCATCCTGCGAGTCTCAGGCATTGCCATGAACTGTAG
ACTCGTGTGTCCAGAAATTCGTCATATCCGTCTCCTCTTTCTTTCAGGGAACAACAAACCCTGCGAGTGAACTCTTTCGC
ATCCTCCTTGTTTGAGAAACAATAGGTGTTGATGTACACATGGAAGCTGTCCTTTATGTTTGTGGATGAAGTAAAGGTTG
AAAACTCATCGTCCGATAACTTCTTTTGAAAACACTCTACAAAGATATACTTTATTTCTCGAATCACAGCAGCCAGAATT
TTTTCTCCCCTTCCTCCTTGTTCGTCACCTTTCCTTGGAGCGTCGATGTCGAAATACGGACATCTCAAGACAGAGCCCGG
AATCACCTCATAGAAATTCCTCTCTAAATGTCCCAGCTTTTTGACAAAACAGAGTGCCTCTTTTCTTGTGGAGAAAGCAC
AGCACAGTTTTCCTCCCCCATCATTGATGTCCTTTGCGACGATGCAGCAACCCCTTTTCGGTCTGAGTGACCTTGTTTCA
AAACATTCTGGGTAAAAACAGTAGAGATAAGGTTTGTTCACAAACGACTTTCCGAATACGGACACTTGAGTTAGAGTTTC
AGTATGATGAGAGTCGGCTTTTGACGGGATGGCGCTCATTGTATTGGGATTCTCAAGCCTCTTAATACATGTCATAAAAT
ATTTTATGACATAAAAATCAGCAAAAATTATCACAAGTAACACATCAAACAGAAGAAATGTCCACAAAGACACCCATGAG
TGGCAAAGAGAGGTCCAGAAAACACAGGGAGAAGAACAGGCAAAGTACAAGGGACAGTGCCAAAGCTTGGTATTGGAGAA
ACAGGGATGCGGTCATAGCAAAGAGACTTGAAAAACAGGAGCAACAAAAAAAGATGGAGGAAGAATTCCAGGCGCAAAAA
GAGGAACTGGAGAGGCTTCGCTCTCTCGTTGGTCAAAATTCGTAAAATGTTTTTCGAGCGCAAAAAAGATATTTGACGTC
CTCCTTGAAAGGTACACGCAAAACTGGAGTGTTTTACACCGCCGCCAACTTTCTAAAAATATTTTATTACAAATATTTTT
ATGTTCTCGCGTCATCTCCGCGTCTCAAAAGAGGAAAAGCTTCGAATAAGTCCAAAGCTCGAACTCTCAGATGGTCTTTT
TGAACTGAAAGAACCTTGAAAACGCACTTTCCTCCAAAGTCGATAAAAACATCGTTCTCGGGAAGCTTGCCGATTCTTCT
CAAGAATCGGCTGCCACAGAATCTTAGCACGGAACAGTGAGTGTATTCCTTCTCCAGTTTTACTGTTTCTTCCAGAACCA
CAAAAGTCTGCCCAGGGTGAATGTCAGGTGAATATGTCCAAAGATTGTGGTGAGGAAGAACAAACGTGTACTTGTCAAAC
GGAAGGCGAAAATCTTCTCCTTGTATCCCCCCCCCCACCATTCTTCGAGGTCGTCTGTGTTCTTTGGAAGGTGTTCTCTC
GTTCTCCAGTTCGCGATGCTGTCGTCGAACGACAGTTCCATATCTCTCTTTTCTCCGACAAAGAATTCCTTTCCGTTAAC
GCACTCAAACAGAACAGAGAAACGCCTGATAAGATGGAGTTCCGTAGCTTTCGAATCCGCGACTTTCTCGAAACGGACAA
AATCTGCGGATTTTCCATATGCCTTTTTTCTCGAAGAAGGACTGGATGTTCTTCCCACTTTGAAAACGTTCTTCGCGTAA
TGCGGACATGTGACGAGATACACAAATTCTCCTGACATTTTGGGTGTGAAAAAAAGTCAAAGGAGTCGCATTATGTTCCA
TTTTGTGGTCACAAAATATTTTTAAATTGAACCTTTCTCCCCCACCCAAGGACTACATTTAAAAATATGAAAACTTCCAT
CATTCCAAACATCATAAAATTCCTCGAAGAAGTTTACTTTCTTCCTCGACAAAACTACGTTATCAATGAGCGCGCAGCTA
TTCTTTCTATCGACGGGGAGACGTTCATGGGTTCTGAGGTAAACATTTTCCGGGCAGAGAACATAACACACCAATACATT
TGCAAGTGGGGAGAGCAGTTTGACGCGAAGGAAGGTTTTGTTTTTTATCAAGGGAGCGGCCTCATCCCCGAAGACGAGGC
GATTAAAATAATCATCGAGAGTATTTCGTCGAGTTCATGGATAAAATGCCATTTGGAAAGCCCTCTTTTTCTCGAAAAGA
AAAATAACAAAGAACTCAGGGAAGAGAACGACCTTCTCCTAAAGAAGATTGAGCGTCTTCGACAAAAGAACAGAGAGCTC
AAATACGCCCCAGGAAACAAAGGGGCTTTGAACGCTCAGCAACACTTTGAAACCCTTCAGGAGAAATAACGGAATTTGAC
CACGCTCCAGTCAACATAAAATATTTTATGAGTCATAAAATATCATCCATAGAATTTTTCCGCAATGTCGAGTTGTTTCT
CGAGATTGTCAACGAATTCGATGCTTTCTTGTTCTTCGAGGTGGAAGTCAAGGCCCAAAAAGTCGTTGCATTTATGGATA
CCCAACACTCGGTTGCAGAGGCTCAGTTCTGAATTCTCTTTGTCCAAAGTCACCGGTCCGAAATCCTCGCCCTTGTGAAA
GCATTCGACAAAATCTCTGAGTTTACCAATGAACTCCTTTCGAATCGCAAGATTTATTTGCACGCTAAAACGACCTTCTC
CGAATTTTTGATAGATATACCAAAAACTTGTGCAGCTCTCCCTATCCACAACAGCAGAATATCCTTGCATATTTTGTCAG
AGAAATAATACTTTTTATTTCTCCACCCGTTCTGTTTTTCCTTTCGGTTCTCGTCGCAAAATACTGCGAACAGAGAATGA
AAGGTTCTCTCCAATGGAACGCATTCCCCCGGCACGTTTGGAAAATTCGACTGCCTCTCCGCCATGAAGAAACAGGAAGA
GCGCCACTCGAAGCTTTTGGAGGAGATGAGAACCATTTTGGAGACCAAACAATCGTCCTTTGTGAAAATCAAACAACTGT
CCCACAAGCTAACCACGAATAAAAATGTCTCTCACTTCTTCGTTCGAAATGGACGGTGATGATTTCAACGGTGTCGTCTT
CACCTATTCCGATGGGGTTGTAAAGACCGAATTCTCTGGCCATCTATGGAACATTGAAGAGACGAAAGAGCAGTTCAGAG
CTTTCTTGGGAAAGGCAAAAGATGGAAAATTTGCTCTCGTCGAGTTTGAAAACTCGAACGGCGACTCCTCAATCACCCCA
CAATAGAGGAAAGGTCTACTTCAGCGTCTCCAAGTACGGAGGGAACGGAAGCGGAGACCTTTCTGTGACTCTTCCTTTCG
AATTGTGTGTCCCTGCCCTTGAAAACGTCGCTTCATAGAATATATTTTTGCTGGATAAAATATATCTGAGATTGGAAAAA
GCTAAAATCTTCCTTTGACTAAAGGCCGAAACATTGCGCGTTTTAAAAAAAGAAACGTTCGCATATTTTACTCACATAAA
ATATGGAACAAAGCAACCAAGTTTATCTCAATGCGACTCACCACTGCGGCATGTGGTATTTTCAAGTCTCCTCGAAAGAG
GAGGATCGCAGATGGGATTAAGTGCGGCTCAAGAGCTCTCCTTCGAACAAATCCCACCATTCCGCCGTTTGATGAGGAAG
TTTGTTCTGTAAGGAGAAATTCTGGATATGTTCTTTTGAACGGAAATGTTTTTTGACTTGTTCATCCGAAGAACACTTCT
CTTGGCAAATAAGGCATGTTCTATCAGAGAAAATTTTGGGTTCACAAAAAGTATAAAGGGACCTGAAAAGAAAATTCCAA
AATTCTTGAAGTTTCGCAACTTTTTTGTGTCAAAATGTTGGAGAAACTTCCGCTCGACAACCTGTTGCAAATTCTTGGTT
ACCTCAACATCGAGGACCTTGCGGCAGTTGACAGAAGTCACAGAGCTCTTCAGTTTGCATGCGCCCAAGTCTATCTGAGG
GATAAAAAGCACCAGAGACTTTCCCTTTTTCTCAAGTCTGTCAATCCAACAATGAGATGGGGCGAAAGGTGGTCGTCCCC
AACCCATATCAGCGCGATGAAAAGAGCCGGAAATACAAACAGAGTTCGGAGGAACCCCTTTGAGGTACCAAAGAACGGGA
GAGTCGAAGTTGAGAAACGCTCGAAGAAGAGACAAGCAATAAGACTGTTGGAGGAGACCCTCTCCAAGAGAAGGAAAGAA
AAGAACAAGCTTTTGTACGACCTTTGGCACTGCAATCAAAACGACACTTTTGGGCGAGACAAGGCAATCCTTGAGGTCTA
TAGACTTTTGAAGGAAACTGTTGATGATGACCTCATCACGACAGAGATTACTCTTTTGCTTCAGGACAACGGTTTCTACG
ACTCTGATGAAGAAGCGAATTTACGTTTGATGATGTGCTTCGACGACAGCGTATCGACATTGGATGGAAAGTAGACTCTT
GCGGAAGAAGGTACATCGACTGTGGCGTGAAACCTCTTCAGAAATGCGAGTTGATGAAGGGTGGTTCTTACGGAAAGGTA
GACCCTGTGCCAATCTCACAGAAGGCGGCTCCCAAGGCGAGCGTTCTCCCTCACAAGAATCTGTTCCCCAAACGATGGAA

TGATCGGATTTCACTGAAGAGCACGCTTGCGTGCTCTTCCATTCTCAGAAGAGCGTTTAGGGAGGAACCTTTGTTTTTCA
CGAGAAGGATTGGCCAGAAATAAATATATTTTCACGCAAATATATAAAAAGTTTGTCTCCTTGGCACCATCGACAAGTTC
TAAAAAAGAACGTATAGAGCGAAGAGGTACGCGATGTCAAACATTGAACTTTCCTAAAATATTTCTTGAAATATTTTTAT
TATGGAGTCTCTCGTCTATTTTTGTCTTGGAACGTCTGCTTCTTTTGCTGTCACAGGAGCAGCCGTTTTCTCTTGGAAAA
GGTGCGTCCCAAAGCTTTTCGACAAAAGAGTGTTGAAGGTCTCTGGTGCTCGTCTTTTCGAGATTGACCCTCGACAAACG
AGAGCGACATTTGCCCTCAAGCCCCGCGGAGAAGTTTCGAAAGTGAAGTCTCTCGGATTTAAAGGAAGAACACACAAGAA
GAACCTTTTGTCTGTGGGAGCGATTCTGGGGAAAAAGGTGTCTGATTCTCTTCCTGTCTTTCCAAATGTGGTGGAAGTCA
AAGAGAGCGACGGAGACGTGTACATTGAAACTGTCGGAGACGAAGATGTAGCGAGCAGCGACGCTGAGGCATTGCAACGT
TACATCTCTCACTATGAAAGGAACCACAAAGAAGATGGAAAGGATTTTTCTGATACCGTGATGACAACTTGTTATTTGGG
AGCAAGTTGTGGCTTGTATGCTGGGGCTGGAATCGCAGTCATCGACATGCTGTGCTCATAGAAAATATTTTGTTCAAACA
AAATATTTTATCAAGTCTCTTTGGTGTGGGAGACTTTAAGGCTCCAAACATCCCAATCTCCGCTGTTTTCACATGGTAGG
TCAATGTCCCCATAATCTCTAAAATACGTCTCTAGTTCTTTGATGGCTTTTTCTTCAGAGAGGTGGAGGGACCCGCTGAG
AGGGACGAGTTCGGGATGTGACGCTGTGTAGTCGTCTTTGACTATCGCAAGGAAAAAGACTTGTGACATTTTATGGTAAT
ATTTTGTTGCATAAAATATTTCTATTGTTCTTTTGTCTCCTTTTTATTGGAAGAACTGAGCTCATGGAGGTTTTGATTTT
TTGTGGGTCCTTACCAAAACAAAGACCTCCGCTGAGGTTCACTTAATCTAATCCGGATTTATGAAAAGTTTGGCGCCCTC
AATCATCCAAAGCGTTTGGCAGATGCTTGGTTTATCCCAAAGTTCTTCGTCAAATATTTTCAAGCAAAATATTTTATTCA
GAAGCACAAAAGGTGCAGCGCTTTTTTGCGGTTTTTTCTTCTTCGCATAAGGGGTCTCTCCAATTTGGGAATTCTTTGTC
CAGTATTTTTTCCCTCTCGATGGTCAGTTGTTTTTCTCTTTTTGGGTCTTCAAGTTTGGCTTGCGCCCTTTGGTTCTGAA
GGAAAGGGTCATTTCTCTTTGGGGGAAGGTGTCCGTGAAGCTGGTAATATTCCCGAGTTTTTCTGACATTTTCTTCCCAT
TTTCTGTTGTTTTGGTTTTCGTCCCAGCCAGGGAACATAGAGTCCATAATTTTTCTTCTTTCACATGGGTAACGTGTAGA
GTTTTTTCTTTGTTCTCCGAGCCATGCTCCCAAAACAGTTCGACTTGAAGGAAATTTTTTGTCGTTGTTATCCATCCATT
CTCTTAGAGCCTCCACCTTTTCGAGAAATCCTTCGTCTCTGGGATTGTACAAAAAATCTTGGCCATGGGACAGAAATATT
CCTCTTATATCGTTGTATTCTGAATCCTTTTCATTTGGAGATAGTCTGTACGCCTTTTTCCAAGTGTTGAGCCTTTCGTA
AAGGTTTTTGTTTTTTCCGGCTGTGAGCTCACAACGGTTTTTTTCGAGCCATTTGTCAAGGTCAATACACAAAATTTCAA
ATTTAGTTTCTCGAAGGTCCCATTTCCATCCTGGAATAATTTCAGCCGCTCTGATTATTCTTTTGTCGACGGGAACCGTC
CTCCTGTTTGCATATTTCATCCTAAGCCTCACACAAAACCAGTACAATTTTTTGTCGGTGGTCTTTTCCGGGAACACGCC
CTTTTTGTCCACAAATTTTTTGAGAAGCTCCAAATTCGCGAGGATTTTCTTGGTTGCAAGTTCGCGGTTTCTTTTATGTT
CTGTCAATGTTCTGTACGCCTCCCTTCCAGAGAGCAGGGATTCGAACTCTTTAGAGTACGCCGTCACATCAAATTCTTCG
GAAAGAATATGTTTCTTGTTGAGGTTCCTTATTTCGTCCAGGCACTTGAGAAAATCTGCTGTAATTTCTAGAGCTTGAAG
CGGAACCTTGAACATCTCTGTTTTTCCCAATCTCGAATCAGAGAACATGTAGTGCATATTTCTCTCGAACAAGTCGCAAT
TGCCGGTTTCGTATGTTCTTTCGAATTCCTGTTTTTCCGAGGAGCCCGTGCAGTATGCGCTCAGTCTTTGTGACAAATTT
GTAGCTTTCCCCACCTTGTATATTCCAGGATTTTTCGAAATATCTTTGAGGATGTACACAAAACCGTTCGGAGAGGTTTC
GACAAGTTCTTTTTCGTCCTTCAGCGTTTCGAAAACGAGTATCTCGGCGAATTTCGCGAACAAACCGCTCATCGTTTTGT
ATTTGTCGCTCGCGTCTTCGAGAGAGATTTTTGGTTCTTTGTCTACTTTGACAGTGCAATGTTTTTCGATGACAGAAATT
TTGACTTTTTGTGAAGAATGAAGGAAGAAGAATTTTCTTTTGCAGGAGCCAAAGAAAATCTTGTTCAGACGTATAAGAAA
TGCAAGTTTTTTGTCTTTGTTTGTCGAAGTCTTCGAGAGAATTTTCTTGAGAGACTCAAACTCAAAATATTTTCTTTCTC
TTGAGTTGACAAGGAACTTTTTAAAATCTCTCGCGCCGTTCTCAAGGTGAGCGGACCCGAAATACTTCCCAATATCCAAA
CAACGAATATGGAGCGCTTTATCCGAGTCAAAAATAAAGTTGAACGTCGCGTCATCGCAAACAACGAAACCTTCCAAAAA
ACGCTCCATTGACCAACGCTCAGTATTTGCTTAAGTTCACTTAATCTAACCCGGATTAGATTAGATTTTCTCAAGCACAA
AAAATCAGAAACAGAGCTCTGTTTCTCTCTTCCCTCCATCCATTCTTTTTTATTCAAATATAAAAAAGGTTCTACTCTAC
CAGAATTCGAGGAGTGACACCCATCGCCATCAGCTCTTGAAATCCGAGTTTTGTGGAGTAAGGTATCGACATCTTGACAA
CGTTGCTCTTGCCGTGACAGGCTCGGCAGTATTTCTTTTCCTTTGTTTCGACCGCGAGAAGACCACAAGCTTTGCAAACC
CACACTTCGAACTTGTCGCAGCTGAGGAAAAATCTGTCCCTCAGACAGAAAGTTGCTCCGGACCCTACAAGGCTGTCTCT
TTCCATTATCCCCGTTTTTAACCCCCCATTTCGGCTTCTGCCTTCTGTCGGTTGACGAGTGAGACTCTGGATTTTACCAG
TCGATCTGGCGTGCATCTTGTCGTCCACCATGTGCTTCAGGCGATTGTAGGCGACAGAACCCACAAACAGAGCGACTTCC
ATTTTCTTGCCTGTGCGACCGTCGATGAAGAACTCTTTTCCACCTGGAGAATACCCCCTCTTCTTCAACTCATCCTCTAC
AATCTGGTAGTTGCTCGTGAAGGGCGTGCAATCGGTGTAGCTTCCAGAGTATCCGTTCGTCCTTGGATTTCTCCTTTCGA
GTTCTTTCAACGCATCGTCCGCTCCAGGAAGGTCCTTTCGTATCCTCGCGGCCAGAACGCGATAAAGTTCTTCGTATTCG
AAACGATGAGAGTCAGGAAGTTCTCTGTGTGCACAGGCTGTCATCTTTCCAGTGTTGCTCTCCTTTGGCTGTCCGATGGT
CATACGAGACGGAAACGCAAGAGGATTGATGAGAAAGTCTGGATGAGTCGCAGAGAACGCATCGGAAGTGAATGGCATAT
TCTCTTCGCTTTCAAAGAGAGAGAACACTCCCTTTTGCGAGTGGAAGCTTGTCGCCTTGTCTCCCGTTTCCGGGATTCTT
GTGGTTGCAACAGCCACACGAATGCAAACGTGTCCGCTCGTGTCCTGAAAATACGAGACGCTGGTGACGCATCCCTTCTT
GTCCTTTCCAAAGTAGACAGATTCATCCTTTCTCGTCGCTCCAGAAACATCAGCGACGGCGATGAGAATGTCTCCGTCCT
CGACCACCTCTCCAACTTCGACAACTCCGTTCGAATCGAGATGGTCGAGACGCGGGCGCTCCTCCACATCTTCGCAAAGA
CATTCCCATTTTTTCTTCCCCAAAAACTTCTTTTGTTTGCTGTCCTTTTCCTTTGTTCCTGGAGGGTCTTGGTTCAAAGG
ATTTCTGTTCCAGAACACAGTCTGCCTGTCGATGAAAGCTCTTCTTTCGTCTTCTTTCTTTCGGCAGACGATGCCCTTGT
GACCAGCAAAAGAAGGAACATATTCAGACTCGCACTCGGGACACAGCTTCACCTTTTTGCCGCAGTTCTTGCACCACAAC
CTTTGACAAGACTCTGAAAACTTGACATCTTGGCAGTGGGAAGGGGAGAGCTGCTCCTTCCCACTGTCGATCTTTGCCAC
AGAGTTTTGGATGTGGTCAGAGACCATTCCTCCCCTCTGCGTAAAGGCCTTGCACGCAACGATGGCATCCTCTTGTCCAT
ACCCCTCGTAGCTACACACTGCTACAACGAGATTCTGACAAATGGGGCTCTCGTTCATTCCAAGGAGACGAGCTGCCTTT
GTGCTTGAAAGCGGCTTCTGAGGGTAGCGAAGAACATGCTGGTCGTAAGGAGCGATACCAAAGTCGAGACGAGGAACTGC
AACAGAAGACTTTGCCATGCTCGCAAAGTACGAGAGACGAGGAGACGGATTGTTTGCAGAATATGGGATGATGGAAGCAC
AAACTCCATAGACCAAAAGAGGATGAATTTCGCAATGCGTCCATTTTGTCTTGCTCTGGGAGAGCTGCTCCGCGGAAGGA
CAGATGAGGCTGTGTTCCTGCTGCTCCGCATCGAGAATTTCAACGATGCCAAGACCGACAAACTCTTGCCATGTGATGTC
ACCCCTCTTCAACCTCTCGAAATGCTCCTGGCCAAACTCGAGCTTCCCATCTTTCACCACCATCAAAGGACGAACGAGTC
TCCCTGCATCGCAGTTTACTCTCACCTCGTTGTGCTTCTCATCAAAGACGAAGCAACAGTCCCAGAAAAAGTTGCCAGCT
CTCTTGAGGACAGAGAGCGCTTCGACGAGTGCCTTGGGAGAGTCGGTCGAACCCATAATGACTCCATTCAAAAACACAAA
CGTCTCCAGTCCAAAATCTTGAGAAAAGTCTTCGAGAGCAGAGAGGATTTCAAAACACTCTTCGATGGTGCATCCGAGGC
TCACAGTCGCAGAGAGAGCAAGAACTTTCGACAGACCAGTTTTGTCCTTTGACTCTGGTGTGTCTGGAGGGCAAATAAAT
CCATAAGAAGATTCGTGGACCCTTCTCGGCTTTGTCATGTTTCCTTCCGTTCCGATGGCTGCATGAACCTTTCGAAGATT
GGACGCAGAAGCGACTCCGTTCATCCTTTCGTAGAGCTGGGAAGTTCCTTGCTTCTTTGTCTTTGTGTTGAAGCTGCTCC

AGTTTCCTGTGCTCATCGCTGTGCGGATTTTCTTTGTGATGTTTTTTGAGTGGATGGGCTTGAGGGGGTCCGCTGTTCTT
TCTCTCCCTTTGCAAGAGTCCTTGATGAACTTGGTCGTCTGGTTCCACATGCTGTAGAATAGATTGTTCAACAGAGAGTC
GACACAATCTATCCTCTTGTTCGTGTAGTGATCTCTGTCTTCGGGTTTTTTATCTCCCGTCTTGACCTTGAGAGCTCTCG
CGAGCATGTAGCACATGAACCAAACTTTCTTGTCTGTCTCTTTGTAGCAAGGGAACAACTTGTCGCAAAGTTCCTTTGAC
AGGGTCGCGACCCAAACGGCGAGCTTGATTTCCGCTTCATCAGCAGGAAGGTCATCGATGCCCTTTGGGTTTTTGTACCC
CATGCAAACAAGAGCTTTCGCCATCGTCTGGACTCTTTGCTTTCTCGAGTGGGGAAACATCGCCCTGGCATGTTCCAAGC
TGGGAATGAGCAAACTTCTCTGCTCTTTTGTGAGGTTCTTTGCCTTGATGAACTTGAGAAGGTCCAGATACGAGATGCCC
ATCGCTTCCAGCACAACGCACAAAGGGATTCCCTTCTCTGAGAGATGCTGTGCATACAACATGGCCACGCCATTTTCAAG
ACCCACATAGACCGTGGTCGTTCTCGTGTTGCTTGTCGCAGAGATGCGTATCTCTGAATGCACCTCGTACTTTGGCATCT
TTTTGTTCGCCTGTTTGTCTGCATAGGTATAGACCCTCCCAAACGCTCCCCTCTCAGAACAGACGACGATAATTTCTCGT
TCGACGATGAAATATCCGCCTCTGTCCGTGGGGTCCTCCCCACGCTGACGACATTCGTCGCCCTGGAGATTGTACCTCGT
CAGGTTGCAGAGACAACTTCTCACCATCACAGGAATCTCTGCGATCGTCTGTTGCTCGTAAACTGCGGTCTCTCCGTCCG
CAGTCGTCACAATGATGTCGCAATAGAATGGAGCAGAGTACGGGATTCCTCTCCTTCTGGCGTCCGAAGGGAAGATGTCC
TTTATCCTATCGTCAACTTCGCGATAAGTCACCCTTCCAAACGTCGGATTGGTGAATTCGACGACCTGAAACCAAGACTT
TTTCTTCTTGGCGGCGGGAGCAGCGCGTTCCTCATCTTCGTCTTCGACGACCTCTTCCTCCCTGTCAATCTCGACCCTTC
CCTTCTCTCGGATGATGTTGGGGAGAAGCTCCTGAACAAACTCATTGTACGCCTGAATTTGATGGTGCGCAGGTCCAGTT
TGAGAAAACCAAACCTTGAAAAGTTTCCACAGTTTGTCGTTGAGAGCGTCCATGTTTTTTGATGTACAAAAAAACTGAGA
TAAAATTCTGGGAGACTCTCGTTTTTCGAAAATGGGACTTTCTCTTTCTCGTCTTTTTTCGTGGGACTTTGAAGAAGAAA
TAAAAAAAGACATCGAACTCGGAATTTCTTCAGAGGAAGAAGAAATTTTCAGGGAGGAAGAAGAAAAAATATTTTGAAGAC
AAAAAATATTTATCGAAGCTCCAGAGGCGCATTTCATTCCTTTTCTTTCACAATGAAGTTCTCAAAGAATCCGAGTTCTT
ACTTTGCTCTTTTGGAAAACGTCAAGGACGAAAAGTTTGCAAACTCTCTGTTCCTTTCAAGAAATGTCAGAGACTCGAAA
AGGGCTGCTCTCCTTTGTTCTCTCGTCGAGGCGATCCTCTTGTTTGACGACGTTGCGACTTTGAAGTACCTCTGTGAGAA
GAGGTGGCTGCTGAAAATTCACAGCAGCAACGAAGGTCTGCTCACCTTTGGCTTTCGCCATTGGAGGCGCGAAACGTTTG
ACATCAAGATCAAGGTCCTCGATGCCCTCATCAAAACAAACTGTTTCAAGGTGCATTCGTGGCTCACACAAGAGCTCGGA
ATATGGCTTTGGATTGGCCAGGAAAACACTTTGGAAAGTCTGGAAATATACGGCAAGTTTCTCTCGCAAAATATTTACAA
CTAAATATTTTTAATGAAGGTATCCCTTCCCGAAGCAAAACAACTCGCAAAAAAACTTGGTATTGACCTTGAACAGGTTC
CGATCCATATCTGGAGATACGGATTGCAGATCGAGACGGAACATTTGGAAAGCGTTTCATGCAGCATGCATCAGGTTGGA
AAAGTTACGCGCGACCATCTTTTAGAATATGGGCCTTTGTACTATGTTTTGCTCGAACGGATGGAGCAGCGACTCGATGA
GCATTTCAAGGGAAAGAAAAAAACCGAGAGTTCTCCTTCCTGGGACAAAACTTCCATCCAAGTGCAAATAAAATATCACCC
AAATAATTTTCTTTTAAAGAAAATTATTTTTTATTTGAATATGGACCATTCGCTACGTAAAAATCTGAAAGATGAGCAAG
GTTCTATACGGAGCTGTCTCTGCATACGGAGCAGAGAGAATTTACGAAAGATTCGCAAAGGAAAGGAAGACATTTCATGA
ACAAGAACTTGTCGAATGGGTGACGAGGCCTTCCGGGAAGGCCTCGGAATACCAAACAGCAAAGGAGAGACTCTCGCTTT
TCTTTTGACGGAACTTTGCTTCCCTTTCGGAGGGAAAAAGGTTGTCTTTGCGGACGGGCTACACTACGAAAAACAGACGG
ATAAAAAGATGGATGAAGGGAAGGAGATTTATATCGCAACACAGAATATCGTCGAATGTCTCGAAAAAATGGTGGAAAGA
CAATTCTTCTGAACAAGGAATATTTTTATATTCAAAAAATATTGAAACCTTCCCAACGAACCATATATTTTTTTCTGTT
TGAAAATGAACGACCTTCCGCCCGAGATGCTTCTCGCAATCCTCGAGTTTTTTGACATCGAAACCCTTGTGTCGTTCACA
GAAACTTCCCATTTTTGGCATATTTTCATCCACGAAAACGCAAAGTTGCTTGTCCAGAAAATCTCGTGCGAATATGAAGA
TGCACCTCTCTTCTTCAAGAAAAAGTTTGATGGAAAACATTCCACCTCTCCCTGCACCTTTTCCGTCGACCCGCTCGGAA
GATTGCAGGGAGAGGTAAAAATCTGTGATGACTCTTTCTCGTTCAAAGATGGACAGCTCCACGGAAAATGCAAAGAGACT
CGGTACTTTGGTCAGGACGAGTGGACACGCGAAACGTCAAACTACAAGGAAGGAAAGCTTCATGGCGTTCTCATCAAGGA
GCAGTCGGATTTGTTCGATCCCCCAAAACTGAAACAAGCGGAAATATGGGAAGAAGGCAGGCTTATTTTGCGAGACATAG
AATTCGAGGAAAAAGAAGGCATCACAACAACATTTTCTCTCTGTGGGGAGAGAGTGTGCCAGGAAGAAACTTGGGATTGC
TACAAAAAGATAACGAAAGGGCAAGGGGCCACAACGACCATAGAATACATTGATGGCGAACGCTTCGTCACCGTAAACGG
AGAACTGCTGGATGGACCGAGAGAAAAAGGAGAAGCGTGGTCTCGCTGCTGTGAAAAGCATCAAAGGGAAATGCCCTGCC
ACATCTAGTGAATTTATTTCTTTTGCAAAAGAAATAAAATATGTTGCTACAAGATTTTTCCTCAAGTTCAAACCCCATTT
TGTCCATGTTTCCGAAAAGTTCATTCAGCGCCTCACCAGAGAGCTCCATTCTCTCCTTGATGTGGTAAGGAGAAATTTTA
GGGAACAAAAAATCAGAGGAGTTTCCTGTATGTTTCGGAGAGACTCGCTTGCGGCGAAAGAGCGTATCCAAACAATTTTG
TTGGTCAAATTACCTCTTTTTCGGAATCCACAAAATACGGCGACTGTTAGGCAAGCTCTCCTCCCGCGACTGTGAAACTC
CCGTCTCGCGCATTCAGAGTGGCGTCTCCCATCTTGGATATCTTTTGGAAGAAAAAGATATTTAGAAATTTTTAATGAGC
ACCTCTACGCTTTTCGCAGAGGGATTTTTCGAGTTGACGCTTCTTCTGCAAGAGATGTGGACAATCTCAAAATCTGAAAA
CTCGTCGAGCACAAGAGGGACGCAAGAGTTGCTCAAAAGAAATTTGAACTCCCCTTCCTTGCAAAGGGTAAAGAGTTTTG
CGTGCTGTTCCGCGCTGAATCCGTCGCATGTATATCCAACGAAAGAATTTGCTTTTTCTGGGACATACGGAGGGTCAAGA
TAAACAAAATCTCGAGTTCCGACATTCTCCAACGCCTCAGAGAAACAAGAGACAGAAAACTTCACGGGAGCAAGAAGCTT
AGAGATTTCGAGGATGTGCTCTTTCTCAAACACTTGAGGGTTTTTGTAGTGACCAAACGGCACATTGTACCCATTAGGCC
CCTCTCTATAAACTCCGCGAAAGCATGTTTTGTTAAGGAACAAGAACATCGCCGACGTTTCTGGAGCTTTTCTTTTTTTC
TCTGTGTTGAAGTTTTTCCTTGTCCAGTAAAAAAACGCTTCCTGGGAAAGAAGAGCCTCTCCTTCCGTCTTTGCGTCTCT
TTTTCCTTTCATCTCTTTGCAAGAGCGAAATTTCTCCATGATGTCCTCCAAAGATTCGATGAGTTTTTCTGGTCTTTTCT
GGATGTTCTTGTAGAAAAATATCAGGTTCTCGTTCTTGTCCGATACATAGATTTTTCCCTTGACTCGAATTTCTCCATCC
CTCACCTTTCTTAAAAGACCCAAAAGGACAGACCCTCCACCGACAAAGGGTTCATAGTAATTTTTTATGGTTTTGGGGAA
AAGCTCAAGGACGTTTTCGAGAATTTGCGACTTTCCTCCGACCCATTTCAAAGGGGGTTTTGACATTTTGCTTACGCCTC
TCAACTTTGTTCTTTTTCTCGTTTTATCTATTCTGTGGATAAATAATTCTGGACAATCTCGGAAAAAGCCACAAACTCGA
CTCCCATCTCATTGTAAAACGCGAGAATTTTCTTCTTTTTCTCGCATGCTTTGTCGCAAAAAACGCCATAACTTTCCCTG
CAAAGTCTTTCCGCTCCTCCGATGCAAAGTATTTTGAGAGGCTTCCCGTAAAGTTCTGGAACTTCGCAGTACAAAAATGG
GACGGATAAAATTTTCTGAGACGCTGTCCCTGTTGTCATGTAAGTTTGCGTTTTCACCTCGAATATCTCTTCTTCTGTTT
CGACATCGGGGGCTTTTCCTTCTTTCTTTTGTGGGCGCTTTGGCCTCTTCCCAAGAAGAACGCACATCTCTTCGCAAAGA
GCTTCTCCGAAAGGTCCGGACCATTGTCCTTTCAGCTTTAGGTCTGGTCTCTTTTTCGCCAGAATTTTCCTTCCCCAGCT
GTCCTCGTATTTTTTAAGTTTTTGCGTATACTGCTTTATCGTCTTCTTCTCCACAGGGGGTAAGAAACTGAGGTCTCCAA
AAATCCATTGAAGAACTTTCTTTTCCCGAAGAAGAACAATCCTTTTGTCTGTATTGTCCTCGAGAGAGTCTAAAAAATTT
GAAACTGTCGCTTCCATATTTTTCTTTACGGTACATTCCAAGCCATGTTTCGCTTCTATCAAAACACAAAGAAAAGATGA
AGGTTCAGATTGTCAACAAGTCGAGCAACGACATTCCTCGGTACGCAACTCCAGGTTCTTCCGGTCTCGACTTGAGGGCT
AACATCCCGGAAGACGTCGTTCTTGCTCCCCTAGACAGGGCACTTGTTCCGACAGGGCTTTTCCTTTCTATTCCTTCTGG

AATGGAAGGACAAATAAGACCAAAGAGCGGCGTTGCATACAATAGAGGACTTACTGTTTTGAACAGCCCCGGAACGGTCG
ACAGCGACTACAGAGGGGAAATCTGTGTGTTGATAGTGAACCTTTCCAACGAAAGCGCAAACATCGCCAAGGGAGAAAGA
ATTGCGCAACTCGTCTTTTCCAAAGTTGCGAGAGCAAAATGGGAGGAAGTTTCCGAACTCTCAGAAACGACGAGGGGAAA
AGGAGGTTTCGGTAGTACAGGAACACAATAAAAATATTTTTACAAAAGAGAAAAATATGGAAAGGAGCGCAGCTCCCACA
TGTTCAAACTCCTTTGGGAGGAGTACCTTTTTAGAGATTCTGATGTTTCCATCCAGGAAACTGTGGAAAACGAACAAGAT
TTTTTTGTGGTGAACGTGAAGCTCTTTATCTCTCAAGAAGAAATTTGCGGTTGGTCTGAATTTCACACTGGAGGCGAAAC
GCGCCATAACTGGTTTTCATGGGGAGATTTCACCATAGACAAGTTTCTCTCCGATGCCTGCAAACTCTTTGATGAAAGCC
CTCGCCTTGTGAAGCTGTCGAGAGACAGACACAAAGAGGCGATCCGAGAGAGGTAATTATAATATTTTTGCTCAAAAATA
TTTTATTCGCATAGAGGACACTCGCCGCACTCAAAAAGCCTTTCATCATCGCTCTCTGCGTTGTTTTTTCCAGCGTAAGC
CCTCGCACACTCTGGGCAGTAGACAGCAGAACATTCGCACATTGCACAGGGAAAGTCCGACGGTGTGTCTGTAAATCTGT
AGCGCAGACAAGCGGCGCACTGCTCCCTGTCGTCTCTCAAAATTCCCTGATTCTTCCTTTTTTGTATCCATCTCTTGTGC
TGTCGAAGAGTCGAAAGAGTCGGAAGTTTTTCCACCCTCGAGACGACGTCGATGGGTTTTGTGACTCCTCTACAAGACAG
AGGAGTCCTACCCCTTTCTGAACAGCAGACAAAACCCGGAACAAGGCCGAGATTTTCCCTGCAAAAAACACAGAAATAGT
GCCTTCCGTCTTCGCACGCGGTTACTTCCTCCCCCCAGATTTCGTCTTCGTTGTCGACAAGCTCTTCCTCATCCACAACG
TCCAAACAGACACAACAGAGATTGACTTGGACGTCTCCATAACCGAGATCTCCATTCTCGAGAAATTTCTGGTTTTCTGC
CAGCTTCTCGAGCGCTTTCTCTCCCATCAGCTTTTGAATCTTTGCGTATCCGCGCGACATCTCTTGTAAAATATTTCTGA
TAAAATATTTTATTCACTTTTTCAAGGAAAAGATAGAATGTTTTTCCAAAAATAACAATCATCGTTTCTCCTTTGACCGC
ATCTGATACACGCCTTTTTTCACTGCACAAGCGCCCCACGAGATGTCCATCATCGTACGTGTTTTTCCTGAGATATCCTT
CACATCGACCGAATCAGGGGGTCATTGTTTTTCTGATGTTTTGCACACTGCACACCCGAGACTCTCAAGTGCCACTTGTT
CGCTCCAAGGAACGACGTTTCTCCATTTTTTAGCTTTTTATTCAAAGGCTAAATTCAAAAAACACAGGCAAACTCGAGGT
CGGTCTCCATATCGAGAAGCTCTTGAAGCTCCCTTTTGACATCTTGGATCTGTTGTTGGACGCACATCAGTTCGAGGGAA
ATGTAGTCTTCCCACAAAAGCCCTTTGTTCGCTTGAAATTCTTCGTCTCCGTTGTACGTCGTCTCAAGGTCAACATTAGA
AAGAATTTCAACAGTCGACATTTTTTGTGTGAAAAATGTTTGGCGTTCCACTCACTGTTTTTCAGTTGTCCAAAGGAAGT
TTCTACTTCTCGAAATTTTTCCCTTTCGGAGCTTTGTCTTCTCAAGATCTCTTTCCAACCCGACGATGGTGGACAACAGA
GAAATGACGGAAGAAGAGAGCTCTCTTCTTCCTTTTCTGCCTCGACAAACTCTTTTACGACAGAAACAAAGTTCCTTTCC
TCAGAATGTCGATGGCGGACCAAGGCACAAAATTTTGAATTCTCTTTCTCAAAATATTTTATTGTGTTTTCGAGCACTCG
AAAAGGGAGCCAGCGACGATGTCTTGTTCGGCATTCGAAAGGTTGTTGGTGTTCCTCACATAACTCACTTGTCTAAACGC
TTCCTTTAGCCGTTCTGTCGCTTCCGCATCGCTTGTAATTTTTGCGTTTGCAAATTTGGCGGCGACAGCTCTGATGACGT
TCGAAGAAAGTTCTGAAGTCGCCTTTGAACGGAGGCAACGAGACAATTCATTGAACGAGCCGAACTCGACAGACATTTTT
TAAAAACCTCTTCTTGTGAGAAGCATTAATTTCTTCTTACCGCAAAGTAGAAGAGAACATGCAAAGCAAGATTCTTCCTC
TAGTGTTGGAACATCTGAAAGACGAACATCTCATTTTAGGTTCAGAGGTGCTTGCTGCGAGCATGTTCTCTCAAAAGGGA
AACATGAGGTTCTTTGAATGTCGTATGTTCCTGAAAGAGTTTGAAGAAACCAGTTTTTGTGGATGGACAGAGCCTGTTGC
GAGAGAAGTTGGGGAAACTCTTGAGGAGACTTTGTTGGACAAAGAAGAAACACAAAAAATCATGGACAAAGTCAAGCAAG
AAATTTCAGATTCTGTCTTGATGCGTCTCGTCAGTTGTCACAAGTACAAGGCAGGGATGGAGAAAAATTTGGAAGCCATT
CAAAAGCTTCGTGAAGAGATTCTTGAACTGGAATACTCTCCAGGAGGAAGAGGAGCACTTCAAGCACAGAAGCATTTTGA
GGGGCTCGTTTCCGGCTTGTAACTCTCATGGAAGAAAAAATTCTTAGAAAACTCGAGAAGGAGTTCTTTGTTGATGTGTT
TTCCCTTTCTGTCAGCATCGACGAGGAAGGGTACTGCAACCTGAAAGACGAAAAGACAGGAAACTCCATATGCAGCTGGG
AGATAAAAAAATACGGAAGAAGGACCAACTTTCGACAAGAAAGCTTTCTTGCAAGCCGTTCTCTTAAAGAAAAGAGGGAAC
ATCATCGGAGAAAAACACAAGGAACTTTTTTCAAAGTTTTTCGAAACAGAGAGGGAGATTAAACGCCTCACGGCAGACAG
GGATTCGCTTCTATAAATATTTTATTGCTGGGAGGAAAGAGCCTCAAAATCTTTAGAGAGTTTTTCTACGTTTGAGGTGT
TGTTCGGAGACAGTTCAAACCCGTCGCTGAAAATCTTCTTCAGCTCGGACAACTCCTTCCTCATAAGAGTCATTTCCTGG
TTCAAAAGCATCAAGTGATATCACGGATTGAGTCTGATGGTACAACACAAAGATTCCATCACCACATCCTTGGCAAACAC
TTCGCATGTACCCACCCCAAGAGAGTAAAGTTCGTATTGGAAGCTTCCGTCTTCCTTTTGTGACAACTTTGCACAAGAAC
TTCCATGAGTCTCACTAACCTGTAGCGAACGACTCCCATCTTTTTTTGCTCTTATTCCGATGTACCACTTCACTCCAACT
TCCTTTTGAATGAGAGAGACGAAATCCGCGACTTCTTCATCCATTGTTCTTTCTTGAGCTTTTTGAACAACGACACTTTT
CTATTTTTCCAACTCGAAATATTTTCAAGAAAATATTTCTTTCAACAAACTATGCAAGACTCTACTGGTCTCATCTGCCT
AACGCACAACGACAAACTTTGGAGAGAGAATGTCGTCCGTCTCGAAAAGTCGAGCAAAATTTTTATTGCAGATGCCCCCG
TTCTCTTGACGAAGAGAACGGAAAATCTTGAGGAAGACTGGAAGAACGTTATCCAAGAGTTTGACAAAAAATTCACCAAG
GTCCCTGGCCATGAATGGCTAAATTATTACTTTGCAGACATAAAATCCGCCATTCTCACTCTGCACGAAGTCATCGATGA
GAGAGAAAGAAGAGAGTATGCGAGACTTTGGGTGGACAACTGGGGAAAGTCCCACCAAAAGTTGAGAAAACTCACCAAAG
AGTACGAAGAGGCGTTCCTTGCGTTGTGGACGCAAGAGCCTTTTCTCTCTTCTTTGATGGAGAAAAAATTCGCAAAGTAT
AAAAAGAAATCCATTGGGTTGGATTGGCAGCAGCACTATCTCAAAAAACTCAAACGTCTTGTTCGCTTGACAGAGGAGTG
GATAAAACTCTCTCAAGAAAACAGCCCTTTCGGCGAACAAGAAATCTCTTTATTGAGAGACATCAAGAATCTTTGGGAAG
AAGATTCCACCTATGTGCAACAAGGTGAGAAAACTTGGCTTTTGCGTCTTGGAGAGATCGCCAAAATCAGCCCTTCGTGT
GCTCACGTTCTGTCGCAAAAGTACATAAAATACAAGAGAACCATTTTCCGACCGACCTCCAACGAAAAAAGAAGGGACTT
TATTCGTGGGTGGAAGCTCAACAAGATGAATTCGGCAATCTCTGACGACATGTCGTTGCTTCGAGCGACAAAGAAGAGAG
TGGAGATCGAGAAAAAGTACGCTCCTGGGGGAGAAGAAGCATCGAAAGCCAAAGAACACTTTGAATCTCTTGTGTCGACG
CAATAAAATATTTTTGGATTCAAAAATATAAACGAGAAAAATGGTACTTTCCATCCAATAGTTTCAAAAACATGCAACAA
GACGTTCGGGAGCTTCTCGTGAGGAAGGGAGTCGCAGAGAAAGACATTGAAATCTCTGTGGCTCCTTGGAAGAGCGGAGC
TTTGGGAGGCGAATTTTCTTGCGTTGTCCAAAACATCGGCGTTAAAATTTCATGGAAGGAATGGGGAGAAGGAGATAAAA
CAGAGTTTGGGTTTCCTGGTGGTTTCGTCTCGTTGGGAAACAAAGAGATCGCCCTTTCAGTCTTCAAACGGTCACTCAAG
AACTCCTCTGGGTACGAGAGCCTGAGAAAAACAGAAAAGAAAAAAGCGAGGAAACAACTTTTTGAGGATGTGCGTCTCCT
CAAGGAAAAAGTCTCTTTCCTTGAGGAAAAGGTCCTCGAACTCGAATATGCCCCCGGAGGGAACAAGGCGAAACAAGCAG
AGGAGCATTTCGAAAGCCTTTCGAGGGAACAATAGAAAATATTTGTTATTGCAAAATATTTTTACGAAATGGGGAACAAG
TCCAGCAAAATCCGAGTCGCTTGCTCCGGAATCTCTTTCAACAGCTTCAGAAAAGGAAGAAGAGAGTTTGATGCGTTTGA
ATTTGAAAAGTGGGCACATCTCCCCATGCCAGAAGGTCTCGGAATTGGAGAGAATAAAACTGGAAAACCTCTTGCAAGAA
AGGCGACAGAACTTTGTTGTGGGATGACAAACCAGAAAATTCTCTTTGTTTCGGGGAGATACGTTCTTGCTCAAACAGAG
TAGGAAACCGACGATATTTTATGCCATAAAATATCCAGAAAAACCATGTCTTCTCCCTTTCCTGAAATCCATAAAATTTT
ATTGGAAGGTCTCGGCATACGCCGAGAAGAGTTTCTCGCTTCCGAAGAAAATCTCAGAGGAGAGCGTTTGGTGCATATTT
CGATCCCAAAGTACGGAGCTTCTGTTTCTTGGTACGAGCCCTTGAGTGGAACAGACACCATCGTGGAAGTTGCGCAATGT
CTAAAAAAGAGGGTGCTCGAATCCACAAAGTTCAAAGAAGAGCAACATCTCAACACGTCGAGAATTTTTCTCTTGGAGGA

AGAAGTCAAAGAGCTGAAAGAGGCTGTGAAAGCTCTCTCGGAAAAAATACTCGAGTTCGAATACGCTCCTGGAGGGATAA
AGGCAGAAGAGGCAAAGAAAAATTTCGAGAGACTTTCTCGGAAACAAACATAACAAAAGGACACCTTCTTTGGTTCCTTG
CACACGTCGCCTGAAAGGAAGAGATGTCATCTGCAAGAGAACTGAGCGCTCAATAGAAATATTTTCTTGCAAGAAAATAT
TTCTTCTTGACGATGGAGAGTGAAATTTTGCCTATCATCGAGGAATTTCTTTCTGAAGAACATCTTCTCGAGAAGAAAGA
CTATGAAGTGTCCATAAATCAGAAGGAAAAATACGACACAGTCTCTGGCATTTACGTCCAGATTTTCCTAAAGAAAGTGA
GAGAAGAAATTTGTTCCTGGTCAGAGTTTTCCGTTGACAACGAGACGCTGTTCTACATCGGCAACGGCATCTTTCGAAAG
AACAAGGTATGCCTTGACATTCTCAAGCTCGTCATCCGAGAAAACAAACATCTCTACACGCTTCAAATGGAGAAACTCGG
GAATGCGATGAAACAAAACTGCTTGCTTGAGCAAAAGAACAAAGAGCTTCTGGAGCACAATGAGAAACTAAGAGCAAAGA
ACAGAGAGTTTCGGTACTCTCCAGGAGAAAGGGGATTCCTCAAAGCAGAGAAACATTTTATCGGTCTCATGTGATGGAGA
AAAATAAATACTTTTGTTCAAAAGTATTTTATTGCGAATACATGGAGGAGTCGCTCATCAAAGACGTTCTCTCGTTCCTT
TCCGAAGAGCATCAAGTACAGGAGGAAGAACTTTTCGTCCAGAGGAAGACATTCCCTTCTCCTTTGGGGTACAAAGGGAA
CGAAGAGATTTATGTCGTCGTTCTTTCGTCGGATCAAGAAAAACTTGTCTGTAGTTGGTCGGAAGTGGTAAAAAAGAATG
GCAAAATTGTGTGGCTGGTTTATGGCATTAAAAACACTAGAACAAACGTTCTTGACTTTCTCAAGAAACAAATAACATGT
TCTGAATTTTTGCTTGAAATGTACCGCAAAAGGTCCAAAGAAGTTGAAACGCTGAGAAAGGAGAATTCGAGACTTGTTCT
CGAGAACGAGAGGTTAACAATAGAAAATTTGGAACTCTCATTCGCTCCAGGCGGAAAGGGTGCTCTAGAAGCGCAAGAGC
ACTTTGAACGACTTGTCAAGCAATGAGAGAAATTCCTTTTGTCAAAGAACATTTTATATTTTTTCTCACATCGTCATGAG
TAGACTCTTGAACATCGTTCACTCTTTCCTCGAGATGTACAATCCCTCTGGTGTTTCTGTGGAACTTTTCTTCCAAGAGA
ACTGCACCATCGTTTCTTTCAACATTGAGAACCCAAAAGTCTCTTTCTATTGGGTCGAAGCAGAGTGCGGCACAGAGTGG
TGGTACGCATTCCCCGGAGAGAACGGGATAACTGACTCGCAAATTCTTCTTGACTTTATCCAGAAAAGGATAGAAAACTG
GGAAAGACAAAATTTTGCCGTTGGAACTGACTAGTATATTTTTATCCACAAAAAATATAAAAAGTTTTAGAGAAGAAAGA
ATCCTTTCGAGAACATGTTGAAGAGAGTTTTGGCGCTTCTTCGGGATGAGTACATGTTGGAAGAAAAGGACCTAAAAATC
AAGCAGATTCTGTACCCGGATGTGGAGCTCGAAGACGACATCATCGACGAACTGCACATAAAAATCCGTCTGGAAAGGAC
AGGTTCTACCATTTGCGAATGGAAAGAAGCAGAGAAACAAGAAGAGACCGTCTACTTTGAAGGAGATGGCGAAGAGGTCA
TGAGCAGTCGCGATATCGTCTGTTGCATAAAAGGGGAACTGGACGAATGCCCCTCCCTCCAAAAGTTTTATAGGGAGAGA
TGTCGAAAGCTGGCAGCAAAAAACAAAAAGCTCAAAGAAAGGAACAAACGTCTCGAGAAAAAATATTCGGAACTAGAACG
CTCTTTAGGAGGAAAGAAATCTCTCAAGACGGAAAAAAGATTCGAAAAGCTCTGTTGAATGGAGAGCACATTCGAAATCT
CTGGACCTTTTGCACAACAAGGAGAAAATTCTTGCGCTCCTTTGAGGAGTGCGAAAAGACAAGAAGGAATTTTGGGACTC
TTGTTCAGTAATATTTTATCCAGAATAAAATATCTAAAATTCGTACTCTACCAACCTCAAACAAAGTTCATCTCCGGTCT
CGAGAATCAGACGGACGTACCTCAACCCATCCCTTTCAAAGACGGAGACATCGTCTAGCTGTTTCACTGTCGGCATTTAC
AAAAGGAAATTCTTGGGTTTTTAGGACATTTTCTAAAAACTGAGAGTCCAGCCGTAAAATATCCAGTAATATTTTACATT
TGAAAACATGGACAGCGTTCTCCTACCGAAAGTCTTGGACATCTTGGAAGAGTTTCTTGTGCAGAGGAACGAGGTGAGTA
TCGAAAAACAGGAAGACTTTCATGTTTTCACAGAGTTTTGTGGCTCCCTTTGTTACTGGCAAGAGAACGATGACGGGACA
TTTCGAGAGGATTCTGAATCTCTGTCAGTCTCTGAAGAAGAGATGCTCGAGTATCTCCGTTCCCGCTTGGAAACACATGG
GCGTCTGAGGACGTTGCATGAAAGAAGAAAAAGAAACGTTCCGGGCTTCCATCTCGAGAGCGTTCTTTTGCCCTTGGTGC
TTCCTCGCATCATCGAGGAGTTTTGTCTCGAAGAGAGCGACATCGGCGTCAGCTACGCACTTAACCCCGGAGAAGAACCC
AGTCTCGAAGATCTTGACAACAATTCTACATGGGTGACCATCCATCCGAAAGATTCCAAACAGGAAATCTGTCGTTGGTT
AGAAGAAGATCTTGGAGACAGAGTTCGCTTCAGCGGAGAAGATGAGGTTCTCTACTGGCCTTCAGCCAAAGAAGCAGTCG
AAGACATATTGCTTCGCGCCACAGATTCTCCAGCCACTTTTGCCCTTGCTCTAAGAAATCTTAAAAAGACGAAACAAGAG
ATCGCAGAGCTGAAGCGAAAGAACGAGAAACTGCGAGACAGAGTTTTAAAGTACAAGTTTGCTCCAGGGAGCGAAAAGGT
CCAAGAGTTGCAGAGGCACTTTTCACAACTCGCCGAATAAAATATCTTTTCAAGAAAAGATATGAAAGGGAAGAGCCACA
TAAAATATTTTTCTCAAGATTTGTAATGACGACAAATTTTGAAACTCGGAGAGAACTTGTCGTCTCCAGAAAGTTTATCG
TTCCTGTTGTTCCGAATCTCGCCTCTGCCATCCTCGCAACTCCGGGAGCGATAGTTTTCGACACAAGCACAGGCAAGCTG
AATGTGGCGACTGGAAGTTCATGGAGTCCTGGAGGCGTTCCCATCGCGACGCCGACATCGAGAGGTGTCGTTTATGGTCA
GACGAGCACCGGAGCCATTGGCGTCGTGTCTCTCGGATATCTGACGACAACAACGACATCTTTCGGAACTTACGTAGGCT
ATGGCGCGGGAGCAAGTTTGGGCAACTTGTCTGTGGATTGCACAGCGATCGGGTACAGAGCAATGGACGTTCCTGCATCC
ATCGCTCGCTGTGTTGCGATTGGAGCGAACGCAGGAAACATCACAGGAAACCAAAACATCTCCATCGGCGCATCTTCATC
CTCTGGCGCGACTGGTGATGACAACGTGGTGTTTGGATTCGCCTCCGCTTCGGGAATGACAGGGTCGAGAAACATTTTGA
TGGGAACGCAAGTCTCTGATGGAGGAATTTCTGGACCTTTCAACGACAATGTCGTTGTCGGCTACAGAGCTGCAAGAGCT
GCAACAACAGCATCTGGAAACATCGTCCTCGGAAGCGGCGCGTCTCCAAACTTGGGAACTTGTGCAAACTGCATCGTTCT
CGGAACTTCAGCGACAGCAGGAACTTCAGGAACGAACAGAATAGTTCTGGGAGCTTCCGCAATCGGAACGACAGACAATG
AGTTGACCATCGCTCCCACAATCACGCAATGGAGGAGCATCGGTCTCTCTTCGGCTGCTGCCGCAAACACGCTTCAAATA
AATCCCGCGACAGGCATTATCACACAGGCTGCTTCATCCAGAAGATTCAAAGAAAACATTCGTGACCTCTCTGTTGACAC
TGGGAAACTTCATGACCTCGCTCTCAAGACGTACAAGTACAAAACAGACGGAAAGGAAGATTATGGTCTCATTGCTGAAG
ACACTTTCGAAATCCTTCCAGAGATTGTCACTCTCGACGCAGAGGGAAATCCTCACGGCATCAAACACCTAACTTTGGCC
ATGTTGCTTCTTGCAGAGGTCCAGAACCTGAGAAAAGAACTCAAGGAGCTTAGATAAAAATATTCTCAAATATTTTTTTG
TAATGAACAACATCTTGAAAAAAGAATTCAGCACATCAAAGGGACTTTTCATCCCAGTGTATCAAAACATTCCATCTGCT
CCAGAGCCAGCCATCGGAGGGATGGCTTACGACAATTCAACACAGGCGCTTCTTCTTTCTGACGGCATCACATGGTATTC
TCCCTCAGACCCTTCCACTCCCACGACTTTGGGAACTGTTTTCGGGACAACTTCAGAAGCTGACCCTTCCGCTACAGGTC
TTGGTTACCAATGCGGTTCTGCTGTCGGCGAGAACGTGTTTGTTGGGAGAGGAGCAGGTCAAAACGCAACAGCCTCTCAA
ACTGGGCTGACCTTTGTCGGCATTTTTGCCGGAAACGCCTTCCAAGCCACAAGCAACAAAACTCTCATCGGAAGAACTGC
TGGAGCGTCAGTTCCTGCTGGACTTGCCCTACAAAACGGAACGGGTGTCGGAAGGGCCGTTTTCAACAACGCAACAGGGT
CTGATGGAATCGCGATAGGGGACAGCTCACAGCTCGCCAACCTCGGCTCAAGAAGTTGTGGAATCGGAACAAACACTTTC
GGCATCACCCTCGGTCCAATTTATGATGACAGCGTGGCGATAGGTTATGATAGGTTGAATGTGCCACAGATTTGCTCGGG
CATCATCAACGTCGGTTCGTCAATGACCGTTTGGAATCCAGGAACTTCCACAAATGTCGTGTACGTCGGTTCTGGTTCTA
CCCTTTCGCCAAACATCTCAAACGTTGTCGCTCTCGGTTCTGGAACTTTTGCTGGGGCCGTTGTCCCAAACAACACATTC
GCTGTCGCCGACGACGTCACACAATGGAGGAGTGTCGGTCTCTCTGTGGCCGCTTCAGCCAACGTCCTGCAATTCGACCC
TGTTACCGGTTTGATGACACAGGCCGCTTCATCCAGAAGATTCAAAGAAGACATTGAGGATGCGGACGAGAGAGTTCCTT
CTTTGGCAGAGGCGAAAGTTTGCACTTACGAGATAGACGGAAGTACAGAACATGGCGTCATCGCCGAAGACATTCCCGAA
TTTTACGCTTGTTCCGACAAAGAGGGAAAGAACGGCGTGATGATGACTCGTGTGATCATGGCACTTCTTTGTGAGGTGCA
AAAGCTCAAGAAAGAGATCGCAGAAGAAAAAGAGAGGAGGAGATAAATTTTATATTTATGGGAAGGAAAACCCTCAAAAA
TGCAAGAGAAAGTTCTCGAGCTTTTCGAGAGCAAAGGAATTCCAAGAGAACTCGTCGAGTTTCGAGAGGACAAAGAGAGC

AGTCGTTGGTATTCTTCTGTTGACTGCTTTGTTGGTGGGACAAACACCCAAATATCTTGGAAACAAGCGGAACAGGAGGG
AGGAACAGTTTTTGCTTTGGCTGGAGACACTTATACAGGCGACAGCGATTACACGTTCCGTCGCATCGAACAAAAGACGG
AAACTCCCTCATTCAAGTCCCTGAAAAAGAGAGTCAAAAGAGAGTTGTTTCTGGGTCTGCTGAAGCGCGTCAAAGAACTC
GAAGAGAAGATTGTAGAGCTCGAATACGCTCCAGGAGGGAACATTGCGAGGAGCGCCCAAGAACACTTTGAAAGTCTTTC
GATGAAACAGTAGAAAATATTTTTATCTCTAAAAAATATTGAGAACGAGAGTTTCACAATCTGCGGTTCCTGGATACGAG
AAGTTCAACCCCTGTTTGAGAAGAGGGAATGTTTCTCAAACTTGAGACACTTTCCAGGTAAAAATACAAAAGAAAAATAT
CATGTTTTCTTTTATGAGCCAGAGAGAAAAATTGTCCCTTTCGTTCTCTGACTGGGGAGCGAAAAGGGATTTAGAGGACA
TTTTTGAGGCACGCATCCGCGCATCAAACAAAATACGAACTTTTTGGAAAAGATACGGGAGAAAGATGCATCTCGGAGAG
GGAGCGAAAAGGGAAGCGTTTATCGATGACGTGAAAGGAACGCCAGAAAATTTTCTCTATTCTATGGGAAACATGAGGAA
CGGTTGGATGAAATGCAAAAAAACGTCTGATTTTTCTTTCAGATTGAACCGCACAGTCGACACTGTTTGCTTGTTGAGAA
TAACAGGGAAAAATATCTGGAAGATTGTTGTGGCCGCCAACGGCTCTCCTTTCCCTGTCTATATCAAGAAAAGGAACAAA
AAGACCACGTTTTGTGTGGAGCTTCCCTTTTCTTTGCCTCTGTTTGCGATGCCCTTCTCTGAAATTATCATTGATGTGTA
TGGAGAAGAGGTTGAAAAGATGGAAATGAAAGGCGCGCAAGCCCTGGGCAATGTCCGCAGCACTACCAAAGCACAAACCC
ACATTCTTTTCTACAGGAGAGCGATAATCAAAGGTGGAGAGTGCAAGTTTGCACCCTTTGGTTTGCGGCCAAAAGGCGAA
GTTGTCAAAAGGAACGTTTCTGCTCTTTTCGAATGAATATATTTTGTGGATAAAAATATATTGAACTTTCTATGAAACAT
TTGCAACTTTTTCTTTCACAATGGATTCCCTTGCCAACGAAACTCTTGTTCACATTTTCGGATTCTTGGACGCGAAGGAA
GCGTTGATGTTCTCAATGACTTCATCCCTTCACAGGGAGTTGGTCTTGGCGAATCCAAAGGTTGTTCGCGACAGAATCTC
TCATGAGAACAGTGAGCACAAAGAAGAGAAATGGGTCACCAACATCCGCGGCGTCAGCACTGTTCTGAGAGTCAGAAAGG
AACAGAGATTCTCAAGGGAGAAATTCGAAACTGGTGCTTTCGTTGATGCTATCCTCTCAAAGGAAGAGGGATGTTTGCTC
AAAGGGAAGAAGAACGGAACCTGGAAAACGGAAAAGCATGAAGATGACTCTGTGTTTTCCAGCGAAACAAGGTCTGCTTG
GACAGATGGGAAATTAGACTATGTCCACGTGAAGGACATCTACGGAGAGATGTTCTTGCTTCCTCGAGAATGCAAGAAGG
GAGAGATGAGCGTTTCGTTCGAAGGGCAAGTTCTTGCCTGTGCTTTTGTCAAGGCCCCACTCTTGACCAAGAAATGTGAA
GGGGTTGAACATGGCATATTCAAGTGCGCAAAGAGGAGGACATACGTCCACTGCTGCGAGAAACACCAAGGAGAGATGCC
GAAATCTTTGTTTTAGTCGAAATATTTTTAAGAATAAAAATATGGATATTTTATTGTCATGGTTAAAAGAAGGTAGGGAA
GAATGGGTAAAAAGGAGAAGGAAGGAAATAAAAGAAGAGGCAACAATTCAGACCCTCATGAAACAGAACGGACAAATTGT
CCCAGAAAACAGAATGCAAAAACTTGAGATTGCGAGAGAAGAACTAAACATAATAACGGGTGTCCACGAAAAGAATAAAT
TGGAATGAGACTATATTTCTGGATGAAATATAGAATGGATTGTCCTTATTGTCAAAAAGAGATTGGAGACGAAATGGAAG
AGGGTTGGTTCTGCGAAGAATGCGGACATGTCTATTCTTTCTGTTGGCGTTGCGACAATGGAAAATGCAAGGAATTTATT
TCTGCCTCTGTTCCGGAGGAAGATGTATCCGGATACGGAGAGATGATGAAATGTCAAGACTCAAAGATTGAAGAGCTGAC
AAAGAGGGAAGCTCGGTCTTTTGGCGAAGAACTACCAGAAGGGAATGTCGTAATTGTGAATAAGGTATCTGGACTCTGTT
ATGCAACCGTTGGGAAGGGAAATGGGTTGTCTGAACCTGCATGCTATATTTGGAAATGCAACGACTGCGGGCAATACAGC
GACACTTGTTCGGATTAGAACGAACATCTTTTATCAAAAGATATTGAACTTGACGGGATAGGTGATATTTACAGCGAAAC
TCTCTCGTTCATATCCTTGGATTTTTGGATGGAAAATATTCACAAGTATCTGTTCCGATTACGGGAATTATTCGGTATAT
TTTTTGAACCAAAAATATCTAGAAAACCGAGCGCACTCGGTCGAATGCATTTTGGAGAAGTTCAGTAACCTTGACGCTCA
CCTCTGATGAATATTTGTTCTCTCCTTCCACAGTTATGGTGAGGTAGTTTCTGGCGCAATAGAGCCTATATTTCGTGTTG
CCGTCTTCCCAATCAAAATGTGCCGAGCGCTGATACATCACAGCTTCACATAGTTTTTTGAAATTTGAGATGTCTTCGTT
TGACCTCACGACGACGGGAAACACAAAGGAAGAATCTCCAACGCCGAAGCCGAAAAGCGTGCGTTGTTCCATTTTATTCC
ATATTCTGGAAATTTTTATGGGGATTACAAAAAAGAACACGCATCTTTTCCGTTGGAACGAAGAAGGACAGAAAAATATC
TTTTCAAAAGATATTTTTACGAAGCGAAAAAGAGGACGTATGGGCTCTTTTCACAGATTTTCTTTGCCTCTTGTGGGCAA
TCTCTACCAAGGACATCCAAACATTTTCTCGACTCTTTTTTCTCACAAGCAGCAAAGGCTCTCGAATTTTGGAGAATGTC
GCAACGTTCTTTGCAGATTTCTTCATAACCTTCCTCCTTTATTTTTTCTTCCAAAGCTTTACTCGTACCATAAATGAGCT
CTTTGTAGTCTTTGCGTTCCTTCAAAGAAAACCATACGCACTTGGACACGCATTTCTCAAACTTTGAGGTGTGGACTTGA
TAATCGTGCCCTAAACCGTCATACCCTAAACCGTCGCTTTCGTTCATGGAGTGTTTTTTGTATGGATGGTTTGATGGGTA
AAAAATCAGTTCACCAAAAGAGAAAACATGTGCCTCTCTTTGTGCGCAGATAGGATTCAATAAATTTGTTGGGATACTCC
AAGGACAAAACAAATTGGTCTTACGACATTGGGAAACGCAAAAGGAAGCGTTGTGATTAAACTTTGGTAAAAGAGTTTCT
TCCACGATGGAAAAGAAACTCGAGGACATTTTCAAAGGGTCTGTCTTTGCGTACGGAAGCGCGCTGTACACGACTTTTGC
AAAGGGAAGAAAATCCTTTGAAAAGGAAGAGTTTGAAAGATGGGCAAAGGCCGAAAGCGAACTGGGACTCAATGACGAGT
CGACAGAGAAGGAGATTGCAGAGGATGTCTCTGTGCTGTGCCTTGGTTTGTCGGACAAAAGGATAGTTTACAGAAAAGGA
CGATATTTGGAAGTGCCTGTGAAGAAGAGGGAATAAATATTCTGAATAAAATATTTATCTGACGAACACATAAACTTTCT
AAAAAGATGGATACACTTCCCAACGAACTTCTGGTTTGGATTCTCCAGTTTTTGGACGTGAAAGGCGCGTTGATGTTCTC
GGCAACTTGCTCAGAATATGGACGACTTGTCTCACAAAACAGTAAAGTCGTTCGGGATTGTGTAACAACAAAGAACGACG
GAACGACAAAGACGGTACAGATCACAAGCATCGATGGAAGAGCAAAAGTTTCAAAGACGAGAGAGATTGCCCATGTTGGG
AAAAAGGGAAGAAAATCCATAGTCAGTGACACATGGTGTCACTGCGGCTCTCTTTTCCGTGGGAAGAGAGTGGGAATATG
GATAGAAGAAAAATTTAAGAAAGGAGCTAAACTCTTCGAAACGAGGTCTTTTTGGGAAGACGGAAAGCTCCAGTACATCC
ATACGCGCTCCAACAAGTGGCAAATTATCGCCTTGCCGAGAGAGCCAAAGGAAAAAGAAGAACTTGTCCGTTTCGAAAAG
AATGTGCTCTCTGTCGCGTTTACAATGAAACTGGCGCTCCATGGATGCGGTGGAAAGAGCTATCGTATTTTTAAGGATGT
CGAAGGGAGAGAATACAGCGCTTGCTGTGATGAGCATCGAGGGGAACTTCCTTTTCTTCTCTTTTGAGACTATCTTGGAC
AACTAAACTTCTCACGGTAAAACCTTTCGCGGACAGTAAATTATTTATAAAATATTTTAACCACTTTCATCATCCGATGA
ATATAATTTTTAACCCTTCTTGTTGACCACAATTACTGAATATTCAGTAATTGGTAATTCTTATATGGGGCAACAAACCA
CAATTCCCAATTATTACGAGGTTATTTTTAAATTGGTAATTGTGGTTTGTTGCCCCATGAAGTTGCAAACTTTCGGAAAA
CAAACAAGGGTGTTGAAAAGAATTGTTTTGGATATATTTTGTGAAATATATCGAAGAGATGGAGAAAGTTATCAAGAATG
TTTTTATCGAACCCACAATTAGCCGTTGGTAGATTACATAAAAATATTGCATAAATAAAATGGGACACGTATACCTGATA
TCCAGACCTTCGTTTGCGGAAGGAGTGGTAAAGATTGGAAAATCAGAGAGCATTAAATCAAGGTTGTCTGCTTATGGGTC
ACAAGCAGTTTGGTATCGAGTGTGCTCTGTTGGAAACTGCAACGAGGTGGAAAGAAAACTCATCGAGTGCTTTACCGAAA
AATTTGCCCTTGTTGAAGGAAGAGAATTTTTCTCCGTTTCATCCATCAAAGATGCGATGAAAATTTTTGACGAAGTTGTC
GCTGATACAGACTTTACCCAATCGATGGGCGACGATTCTTTGGTGAAAACACCTCAAAACTCCTCGGTAAAAACATTTAT
CGACGGGTTTATGGGTGAGTTTTCAACGGAAAACTATACCGCATTTGAAAATGGCTGGATTATGGCGTCAGATATCTACG
AGAACTTTGTCGAGTGGAGCAAACAAAAAGGGAATAGTCATCATGCACACAACAGCGTATTTGGCAAAATGACAAACTCT
TTGTTTGACAAACAGAGGATGACACTAAATGGAAAAAAGCATATATGTTGGAAACTTCCCTCCAACAAAAACGCAGAACA
AGTTCAAAAAGCCCGTTCCAATTGTTCATGGAAGAATTTGACCCATCTAAATATGAGGCAAATAAAGATGGGTGGATTCC
ATCAGCAGACATCTATTCTGATTTTGTTAGTTGGTCTGTCAAAAATGGCTACAAAAACACTCCAAATGCAAATGTGTTCG

GGAGAAGCACCAACTCTCTCTTTGAAAAGAAAAGAGAAACATCGAAAGGAAAGAAAGTATCGTTTTGGAAAATCCGAGAG
CGCGATTCCCAAAAGGAAGACATAGTAAAAGAAGAAAAAGTGGAATAGTTTTTTGGGGCGACCAAAAAAGATATTTTATT
CAGAATAAAATATTCTGTGGGAGAAACCAAACATAAACCGGAAACAAAAGTATATATTCGTTCAAAGAAAAACGCGGAAG
GGATTGCCTCAAAATTCTTCAATCCATAAAGGCAATCCCTTCCGCGTTTTTCTTTGAACGAATATATACTTTTGTTTCCG
GTTCGTGCTTCAACAGACCTTTGGAATCTCGAAACTCTCCATTTTTTGTTTTTGATGCACTGTCGTTGTTTCTCCGTATA
ACCAAGGTGACGAAAACTCGAGCACCCATGAAAACCTTTTGCTTTTGTTAGTTCTCTTCAATCCAAAGTCGAATGTTCTC
CTAAGTATTGAGTTCACCCAAAACATCATCCCCTGTTCTGGTGGAAGTTTCCCAAATAACTCTTGGAAATATCTGCTTTT
CGCAACTTTTTGCCTCACTTCTTGAATTTTCTTCTTCATCTCTGAGCTTCCAATCTTTTTCGCATCCAAAACGCTCTCAT
ATCCAAGCCACAAAAAGAGCCTTCTTGCATATATGACCTTCTCGAGATTCACAGACAAAGCCCAACTCTCTTCCCTTTTC
ATTTCAAGTTTCGCTTCGTTCTTCTCCTTCAACATGACCTTCAATCTCAGAGTTTGTTCTTCTCTTGTCCCTGAAAATGC
GAGCTTCTGATTTTTGAACTGCTTTTCCTTCCCTTGGTAGACTTCCACAAATTTTGGGATTATGGCGCTTTGTTGTACTT
CAAAGCCGTGAGCAAGCATGAACTTTTTACAACTAAATATCTCTTCTTCTGTCTTTTCTTTCCTTGAGCAAAGAAAAGAG
AATTTCTCCCTCGCCAATTCTGGAGCAGAACATATTTTGTGGATGTTTTCACCCTTTATCTCTTCTTGCAAAAGTTTGTT
CATCTTCCCGACTTCTTTGAGTTCTTCTCCTTTCAAAATTCTCTCTGCGTACTCTACGGACGCTCCTTGCTCTTCCAAGA
GTTTTAGCAAAACACCCGCGATGTTCACCTTTGAAATATTTCTTCGTACCATCGTTTCAACGTACGCTTTCGAACGCGGA
GTTTCATGATATTTTCTTGTTGCTGGGTCCAGTTTTATTCCTTGAAGGGAAAAAGAGCAGCTCGTTCTGTTCTCAAGACT
CTTGATTACTCCCTTTCGCGTTGTGGGACAGTTGGACGTTCTGTTGTCGAAGGCGAGTGCTATCTCTTTCATTCCAATGT
TTCGAACGCGGAAGAGCATCTGTTCTGCTTCTTCGGCGCAACAGGATCTCGAACTAAAATACCCGTACACCTTGTCAAAG
TGAGTTTCCTCATAGCTCACTCCGGCTGAAATTTTTGGAGTGTAAGCGACAAGGTCGTAGTCCTTCCAGCTCTCCAACCA
AACGTTCTCGCTTTGGCCTTCTCCCGTATACCAAAGAACTTTATAGTCTAGGAATTCTGCCTCTTTGCAAATATTTTGAA
GTTTCTTTTTCGAGCTGCTTGCAAAGCATATTTTGTTCCCTTTCGCGAGGTCTCTCAGAAGAGCTTCTTTGAACTCGAGG
AACCCATCCAGTACAAGCACCTTTTTATCAGAATGAGCTTTGTGTTCGTTGCGAACAACGTGACATGAAACACCGTTTTG
TTCAAAAAGGTCGATGGTTGTTTGGTCGAGGTTCTTATCCATTGCAAGGACCCTCTTTGTGTTTTTGAGATAATACTTGA
ACGTTCTCCAACAGCTCTCCTTTTCTTTGATGTCGGAGAATAGACGAGCGAGAGTATATGTCACCTCATCACATACAAAA
AGGTCATATTTTCCAGACACCCGATGGAGTGAATCTATCTGAACGACGAGCCTCGACGAAAAGAGCCATCCTTTCCCTTC
ATCTTCGTAATTGGAGAATCCTGGAAGATTGTTTGACATCTCCCTCGCGAGAGTTCGCCTATATGTGACGGCGAGAACTC
TGAGGAAGGGTCGCGTTTTCAGATAGTTTGCAAGAGCTTTTGTTTTTCCTGTGCCCATCCCTCCTTGGATTGCGATGCAC
GCCTTTCCCTCTTGAAAAATTATTGGACGGGAGGTCGAGGAGGAATAGCAAAAATCTGCCTTGAGCTCTGGGAGGTCTGT
TTTCTCGAGTTTTCTGGGCTTTTTGGATTTTTGCGTGTTCTTTGTCGGGGCTGTGATGGGGAAATATCTCCCTGCACGGA
AGCAGTAAATCCAAAGTCTGTCGTTGTAAACGCTTGCGAGGTAGTTATCCGCATCGTGTTCTCTATCGCAAAAGGGGCAA
TTATTGGATTGCCCCTTTTCTCGTTGAATGCGCCATTTGGTTTCTCCTTGTCTCTGGATTTCAAAAACTCCTTCGAGGTC
GTTTTCCACAAACTCGTTCAAAACATCCTCAAATTCACCAGGATTTTCAAGGCAACGAAATTTTACTTCTTCGGGTTCCG
GTTCTGTATATTCTCCCATCCAAAGATATCCTTTCGGAGTTCCAAAGAACTCGATGGGGTCTTTATCTGTGTGTTCTGCG
ATTGGGAGCAACCTTCGGGAACTTTCGGATTTCACTGACCAGGGAGAGCGTATCGTTCTGTTTGTTGTGTATACCTGGAT
ATCCAGCATCTTTGCGAGAAGTTCTCCGTCCCTCCTCTTTTCAAGAAATTTGATAAACTCTTCGCATAACCCCTTCATCG
AAACAAGGTCGTAATGCGTCTTTGTTGTGACGACATGAAATGAAGTTTTGTATTTTCCAGATTCTTCGCCGCACGCCTCT
GTAATAAAAAAGTCAAATTTCTCTTCTGGGGAAAACTCCCTTCTCAACTTTTGGAAAAGTTTGGGAAAGTCCATATCAAG
GATTTCTTCCTTTGAAAAATCAGAGCTATCGAGGTCAAAGATTTCTCTGGTTGGATGTCCCTTGAGAAACTGTTCATGAA
ATCTTTTCTCGTTGTCAGGAACAACGACGTAGTTCTTCCAATACTCTTGGAATGTTGGGTAGCTTCCATAAGACCTCGAA
TATCTAGTTGTTTCGTACGCAAAGAAAAAATCGTGACCACAACTTTCTCCAAAAATATGGGCAAGTTTTTGTGGAGAAGG
CAAGCCGACTTCCCCTCTTTTTTCTCTTTCATTCTTTGACAGTCGATGCTTTGACCTGAAAGAGTTCGAGAAAAACCAAG
CCCCGAACCTCTCCTGAACACAGCAATATCTCGGAAGTTTCCCCAAGAAAAACGCCAAACTTACCCTTGTCGAATATTCT
TGTTCTCCTTCTCCCTCCCCTCTCTCATAGAATTTCGAAGTCGCTTCGGGCGGTTGCATTGTTTCAGAATACCTCTTCCG
ATGCTCTTCACTCTGATAATGTCTCTTCAAATCTCCCCCATCCTCTCCCTCGAAATCGCAGAGAGGGCATCCGAATTTTC
CATCCCAAGTTTCAGGTTCAACAAAATCGGGTTTAAGGATAAGATTGTAATTGGTAGTGTTTTCGCTCTGTGATAGCCGG
TCTGCCATTACAGGGCGTTTTTATTTTCCTTAAGTGCGTTATCTGTAAAATTTTCCTCATAACGAAAAATGTCCACCGGT
AAGATGGATGATAAGAAAAAGATCGCAAAGACGAACGCAGAAAGACAGCGCGAATGGGCAAAAAGAAATCCAGAAATTGC
CAGAGAAAGGAACAGGGAGTGGTATGAAAGAAACAAGAAAACTCTTTTGGCAAAAAAAGCAAAGCAGTACGCCGAAATCA
CGGAAGAGGAAAAAGAGCTCAAGAGGAAGCTAAAGAAGGAGCGAAAGGAAAAAGAAGAGAAGTTTGCGCGTTTGGGAGAA
ATTGTCGATGGATTTCTCCCAAATATTTCCTTCGAAGAGCTCCGTTCCCTCCTCCAAACTCTCGCCCCCAAAGACAAATA
AAAATATTTTCTCCCATAAAATATTTTATCTCAAATCAATGCTTTCAGTCTCTCATAAAATAATCTCAAACATGGAAGAA
AGAACTTCCAGACTCTATCCTCATATCGAGGAACATCTCAAGGAAAGGTATCTTTTTTTGCCGGGAGACTGCAATGTCGT
TCTAACTTCTCGCAAATTCCTTACACAAAACGGCTCTGAGGAGGGAATTCAGGTAGGGGTGTACAGTCGGTTCTGTGCGA
AAAGCCCTTTTTTTCTTTGTGGATGGGGAGAACTTCCAGATTCTCGTGTTTTTATTTCTGACTTTGGTGTTTTGAACGAA
GTGGAAGCGCTCAAAAACATAAAAGAGTACATTTCGTCCCACAGAAAAATACAAAAATACTCTGAAGGTTCTCTTTATCT
CGAAAGACTGAGGAACAAAGGGCTGCACACGGAAATTGGCATCTTGCAACAAGAGATTGAACGTCTCAAGCAGAAGAAGA
GAGAACTCAAGTACAGACCTGGAGGATATGGAGCTTTGAAGGCACGACAACACTTTGAAAATATTTTGAAATAAAATATT
TTAAATCTCACTCAGAATCCTCCAAACCAAAGGCAGAACGTTCCCCTTGTCACCCTTCATCTCTTCCTTCAAAGTTTTTC
GAACTCGCTTCTTTATGTTTTCTTTTGTGGTGTTTTTGTACAACAGAAACTTTTTCTCTGTCAGAAGCTCCATCAAAACA
CACAGAAACCTCATCCTATCTTTCGACCCAAACGTCTCGCCAATTTCGTACTTGTCCAAACGAGCCATCAGCACGCCATC
AAAAGTCACAACAAAATCTTCCATCGTCAGCTCTCTATCAAAGCAAATGTTTTCCCAACTGCAATGTTCATAGAACCTCT
CCATCCAAAGACCGAGAGTTTGTGGTGACTCAAACCATTGGAGTGGTTTGCTTTCTGGATACTCTGCAAAGACACAGAAA
ACGTGCCCCTTCCCATACCGAACGAACCCTTTTCCAAAGATTTGAGGAGTGAACCTGCCGTCGTTCCCCAGAGCTTTGAG
ATTCGTGGAGTTTCTTGCAAGCGTCGAAACCTCTTCTCTCGTGCTTTCGGTGAGAGGAACAACAAACATGAGATAGAGCT
TTTTCTCCCTTGCCGGACTCTCGATAATCCCTTTTGTGTTCCAGACTACATTTGTGGTCGCAAGCCAGAGACGAGGCCCC
TTCCCATTACACAGGAAGGAGACGCAATTCGGGGACTGAGCAGAGCCGTTCCACTCTCCGATAATCTCTCCAAAGAGGTC
GTTGCAAAGCGCGGTCGCTTCTCCGCAGAGGTCCAGGCAAGTTCCGTCGTTGACGTCCAAAATGTCGTTGTGCACGCTGT
TGAGAAATTGCATTTTATTGTTGGGAAACTCTATCAAGAAAAAGAGTTATGAAAAAGTCAACAGTCTGCGGTCTCTTGTA
CAGCGTCACGTCCATCTGTCTTCCGACCTATTTTGTTCCTTTGAACTACAACGAATTCAAGGCAAAGAACAACAGACTTC
CGACGAAAGAGGAACAAAGTTCCATCGTTTGGAAAAGCACGATGCACTCGTTTCTGTTTCCGTACTTTTTCCCGATGAGT
CTCATCCAGAAGAGAAAGATTTAACTTTTGGTCAAAAGTTAATAAGATGTCGTTCACAGCAAGGGAAAAATTGGCGGAAG

CTACCTCATCGGAGCTGCTGGATTTGCTCCTCTCATCGTGAAAGACATGGAAAAGAGAACCAGAAAAGGACGTTCTGGG
AATTTATGCCAATCTTCCTTTGGAGAACAACGTTCTTCCCATACTATCAAACTGTTTTTCGAAGGATAAAATATAACCAC
AACTAAAACATGAGCAGAAGACTTTCAAACTTTGCTTCGAAGCGCGAGGACGCTTTCGCGATGTTTGCGATCTCTTATTG
CTACTGTGCGACGGGGGCAGCACTCATCATCGGCGCAGAAGAAGGCAGAAAATCCGTCGACAATGGCACAAAGTGGTATC
AGGACCTTCCAAAGGTTCTTTTCAGAAGTTATTTCTTCCCTTTCTACATTCGGGAATATCTTTAAAATCTTTTTTCAAAG
CTGGATTCATCCACAAATAAAAGAAGATGTCAAAGGCAAAAACATTGGCTTGCACTTATGCGGTGACTGCCATTCCGCCG
TATCTTTTGTTTTGTGTTGAGGAGGGAGTGAAGTTGGGATGGTCCGAACAGAACGCGACCTTTGGAAGCGTGACTTCTTC
GTTTGGGTTTGCAATGTTCAAAGGGACATTTTATCCATACTATTTTTACAAGTACGCTAAGGGAGACATATCTTTTTGAA
TAGAAAAAGGATATGGATAGTGAAATTTTTGAGTTGGCGCTTGGCATAATCCATCAAGTGGAAGTCAACGACCCTGAGAC
GTTTGACGTGCTGCTTCCAGAACACAGAGAACAAACAAAGAGTGAAATCTTGGAAGATGTCCTAAAATACATCCAAGAAC
ACGCCGATGTGGACAAGGGAGATATCATTGTCGAAATTTCGGCCGGTCGTTGTAGGACGGAATGTCAATGTAAGAGAGAA
TTTTATTTTGAGAGAGTTTGCAGGGAGTGCGTCCACGGCCTTCCTTTTTTGGAAGATGTCCATGAAATCTCTGCGAGTGT
TTCCATCAAAGGGTGGGAATCTCGCAGAGACGTTGTTTGTTATTGGAGAGAAGTCAAGGATAAAAACAAAGGGACCTTCT
CTGTCTATCCCTTTAGGTTTCATGAACCTTGCAACGCCAAAGAAGCTCTACCCAAAATAATCTCTGTTGTTGGGAAGCAC
AAATTCAAAAACTTTCGGAGGAAGAAATTCCTGAAAGTTTTCAAGGAGACGTGCGATCTCACCGTTGCGCTCCAAGAAAC
AAAAGAGAAGAACAGAGCTCTTCGAGAGGAAGTTCATACGCTCGACAAAAAGAACAAAGAGCTTCAAAATTTGCTCGCAG
AGGCTTACGCTCCAGGCGGAATTTTAGCAAAAGAAGCGCAAAAGCACTTTGAAAGCCTCTCATAATATTTTGTGAAAAAT
ATTTATTAGGATCTTATCTTGTCGCAAAACAAGAAGGCTTATTTTGGCGCAACCAAAGGCTTTTCGTCTCTCTGATAAAA
TTTCTCGGGTAGGTTATAGTCTTCCTCTCCAGCGTACAGTTTCGAAGATTCAGACATCTCTTGGAGTTTCTTTGCTCTTT
CAGGGTTCCTCAAAAGAGACACGTGGTAGCGAAATACGGCACGAGCGAATGTTTCGTCCATTACGACTGGTTTATCAACA
AACGCTCAATACAGAAAAATTTCGACGATGTGCATAAGTGCACCAGAGGTGAGAGAGATGGAGGTTTTGAAATGGTTCTG
CGCCTCCACAGAAGAACATAAATATTTTATCAAGAGTTGTCGTTTGAAAGACACAGATACACCATCGAAGGGTTCATGTA
CTTTTTCTGAAGGTACATGTTTGCCAGAACATGTTCGCATTGCACATTGTCAACTTTTTCTGGGATGATGTCGTAGTTGA
AGCCAGACTTTACGAGACTCGAGATTCTGTAGATCGCACAGCCTCCGAACGCGCTATCCACTTGTTCGAGGTCGCCTCCT
GGAGTCGAACCGAGATGGTCCAATTTCCTTCCAACGGCATGGGAAATCCATCTGTTGGGATGGTATCTGCCTGCGCCTCC
CAGTTCAGTTGTCGCAAGACCATCGTAAATGACCTGAATTCCACCAGGGAATCTCAAAATTCCAAAGGACGCGATCATGT
CGATGTCTTTATCCTTTGCTAGCCAACCAAAGCTGTTGAAAAACCCGTCTTGATACAGACGACCGATGAGGTCAACGTCC
TGAACAACAGCGTAGTCCCAATCCTTGAAATCCTGCTTTATTTTATCCAAATAAACTTGTCGAAGCATCGACATCTTGTT
TATTCTTCCCTTTGTCACTTCGTGCCCTTCTGTCTTTGGCAAGTTCATGCTACAAGATGGCGCGTTCACACCACAACCCA
GAACCATCACTCTCTTGTTCCTCCTTGCCCACTCAAGAAGAAGTTTTCTCGTATTGTCTTTGGAGTCGTTCTCGACTATA
AGAAGGCGCCAGTCCTTGAATCTGCCGTTGAACCTTTCAAAATCCTTGATGATATCTGGAATTCTCTCTTCTCCGTCTCT
TATCATTGCACAGAAAACCACTCGAGATTTCCTTGCATGTTCGAGGCCAAGTTGAACCATCCTGTTGTACCTCTCTGGCC
ATTCCTTTCCAAAATCTTCAGGTGTTTTCTCTCCAAGGATTTGAGAGTTGAAGATGATCTGAACCTTCCAAAAGAATTTC
CAGAGGAGGAAGAGGAAGATGCCGACGCAGAGATAAAAGGCAAGCTCAAACGTCTCCATCCTGTGTTACTTGTCGTGAAG
AATTAAAAAGAAAAGCCTCTTTGTTCCAAACAAAGAAAAATGTTCCAAACGAAAAAGAGGAGCAAATGCGTAGACGACTG
CGTCTACAGGGGTTATTGCGAAACGAGCAGTCCGGAAGTGATGAAACAACTCAACGAGGAAGGACATCTTCGTAGCTTTT
GCAAACAAAAGTGTAGAACCATAGAGAACAGCAGAACATTTGCAAAGTGTGAAAAGAGAAGGAGAAAGGACTGCGAGTAC
CCTCTGGCCGACGATTGTAAAGAAGAAGTCGAAGAGGCTTGCAAGAGCGACTTGCGCGTTCTCTTTGTCAAAAATATTTT
ATACTAAATATTTTAACGGATGTTTCTTCTCGTACTTGTCGTTCTGGGTTTTCTGGTGCTCCTTCTTGATATGAAGAGGA
GGAAACTCACTCCTTTTGAAGTCAACATGGTCAAGGTAAAATCTCGAGACCCTCTCCTTCCGATAAAGTACGCTTGGTGG
TTCAAAGACCAAAAAGCAGGGAGGCCTCCAGTTTTCATGTTTTTCAGGGACCCGAAAGGAGGAATTCTCCACAGTTTCAC
GCTCGACGTTCCAGACGCTGACAAGGCGAGTTTTCTTTCGAAGCGAAATGTCGAGTTTCTTGACCTTGAAGTGAGGGATA
AACGGGACGATCTGCTTTGGTCTCAAAGAGTAAGATTTTAAAAAATATTTTATACTAAGTATTTTTATACAAGGGTCATC
GTACCAAAACCAACGTTTATTCGAGAAACAATCTCCCTCGTCATCCCAGAGCACAAAGTTTCGTCTTGTTCTCCCTCTCC
TTCAGGAATTTCAAACTCTTCAATATCAGGCTGTTCAGAACCACAAAGTTTGCCAATCTCATCTGCCATTTTCAAGAGGC
AGGTGTCCCTTTCAATCTGAGAAAACATGTCAGAACCAGAAGGCTTTGTTGTGATGCCGTAAAGGAACTTTTGGCCAGAA
ATCTCTCCAAAAGCGACAGGTCGAAAGTTGTTCGACCTAAACTTTCCAAGAGAGATCGCAGAGCAGCGAGAGCGGATGAT
GAATGTCGTGTGAGTGTTCATTGTGAAAATAAAGAGACGAACGAACTTTCCTTCCTTCGATAATTTTTTGAAGAGAAAAA
ATTATTTAGTCGAGCATCTGTTCCAAAGTTTCTCTGTCGAAAAGTTGGAGAATTTCTGTCCACTTTTTGTTCCCCCGAAC
GAACAGCTCCATATTTTCACTTGTCGCAAACAAGCGGAAAAATTCAAAGAGTTGGTACGTCTTTGCTTCTTGTTCATCGA
GTTCTCTCTCTTCTTCTTCTGAGCCAGAGACGTAGTCCGATGGGTCGAATATGACAGCTTCCTCTTCCATCATCGTTGTG
CTCCCAAAGTCGATGACCTTGATGCGTTTCCCGTCTTTCGAAACGAGGATGTTTGCCAGATTGAGATCGGCGTGAAACAC
TCCGACAGTGTTTAGTGCTTTGGCTGCCCAGAAAAGCTTCTTTTCCACAACGCTCTTGTCTTCCAAGTTTTGCAGAGGTT
CACCGTCAAACCATTCCATGCCGATGACAGCGGTCTCCTCTTCTCTGTCAAACTCAAGAAAGAGAGGTCTTGGGAAAAGT
TCAGCGTCTGCTCTCCAGGCGAGACACATCACATCAAACTCTCGGAGGATTTCGTCTTCCTCTCCGTTTGGACGACATCT
CATCACCTTTTCGGCAAACACTCTTTTTCCCTTTGTGCATTTATAGACACCACCAGAGCATCCACGAGCCACAAATTCTC
TCGTCGCGTTTTCGTAGCTTCCATCCCTTCCCTTGGCGAAGAGGTCGCTTGCGATACAAGAACGGAAAGACATTGTGAAA
GGAAAAGACAGATTTCTCTCTAGTGTCTGCAATTTATATATTTTCTTGAAAAATATATTAGTGTCCGCGAGAAGTGAAGA
GCTGTTTCATCGAGACGTCCTTTGTGCTTCTTTTCGCTTCGCGAATCCAAGACCTTTTTTGGAACGAATAGTCCAACAAG
AAGTTGACAACGTCGGCGTATCCGTTTTTTGTGGCTCCGATGAGAGCTCTCTTGGTTGAATTTTCCCTACGATGGAAGAC
GAGGAATTTGACAGAATCCAAATGTCCATTCTCTGCTGCCAGGTCGAGAGCTTCCTTCCCACAACCGACGAGGCTGTTGC
AGTGAAGCACTCTCAGAACCTTGACGTGACCATTCGCCGCTGCTCCGTTCATCGCTTCTTTTGTGGCACCTCCTTTCTG
ACAATCACAAGAAACGAGACGACATGCGAATGACCGAAAGTTGCAGCTTTGTTCACAGCGTCGGAAGTGCAACCTTCCGA
CCTGTTTTTGTGGAGAAATTCAACAACAGGAAGATGTCCTTCCATTGCCGCCCAGTCCATCGCCTTTTTGCTGCATTTGG
TGCCGCAGCGATGGAAAGTTTGGACTGAATGGAGGTCTCCCCTCAAAGCTGCAAGGTCAAATTTGTGAGACGACATTTGA
AAAGTGAAAACAAAAGCTTCGAATGTTCCATCAAAACAACAATTTGAAAAGGATGTCGAAAACGAGACTTGTCCATAACG
TGCAATTTTCAGAGATTGGAGAGACAGGCCCTTCGGCCAGGAAAAACGCTCTCGCTTTTCTTGTGGAGACGGGGAATGAC
CGCATTTTGGTCAGCATGCCTGTGGAGCTCTCTGGGGGAAAAGTAAAATACCCTGTCTGCTCAAAGTGCGGAACTTGCGT
CGTTTGGTCTTATGGACACTGTCGCTGCAAGAGTTTGAAAGAGGAAAAAAGCGGACACTACTCTGTGGACGATGAATATT
TCTTTGAACATAAAACGTGGAATGCATGAGAGCCTCCAACCATCAAATCAACCAATATTTTCCTTTGCATCGACAAAGGA
AAAAAATGCAAAACGAAGACATTGAGTGTGCCATCGCTGATGCAGGAATCGTCGGAAAGATCGCGGTTTCTGGTGTTGGT

CTTTTCTGTGGTTTCAAGGCAGGGATTTGCGCTTCCGCTGCTGCTTTTGCTCTCGGACCTGCCGCGTTTGCGACTTTTTC
GAGCGTACCTGTGACGCTCGCATTTGTTCCGTGCGCCCATGGCATGGTTTCTGCCATTGCGAGTCTCTTTTTTACAAAGT
CGTCAATCGTCCCTTGGATTTGGCTGAGTTATTCTGCGGGTGCTCTCGTTGGCGCCTGTCTCTTGAACAAGCCAAAGTTT
CTTGTCCCTTTTGTGGTCATCACAACCGCATATTCCATCGCAACCAAATAAAATATTTTTTCGTATAAAATATTTAATCA
AAACCATCGCGTATACAAAACGTAGTTGTCTCCGGACAGTTCGAGACGCGGTCCCGCTTCCGACCATTTTATGGCTCCTT
TTGCCTCAAAGTCTTTCATATACTCGTCCAAATTTTCAAAATCTGGAACCTTCCGTTTCCCCATTTCCGACAATTTCTTT
AGTGAATATGGGACACCAGAAACCCAAACGAATTCGCCTTCGTGCTTGCATACAACAACAAAAAAGGTTGACCTGTCCTT
CTGAAGCCAAAAGATTTGACCAACAAACTGCATGGAGCTCTTTCTGTTCCTCGAACAACAAGAGAGAATCTCGTTTCTCT
GTTCTTGCGATGAAGCATGCGAATGGGGAAACTCCAAAAAGAACGACTCAAAACATCGCTTGTCCGTAGAGATGAAAACA
GAAACGCTCTCTAAAAATCCAAACGCTTCCTTCGTCGTCGACGCTCTCAGAAACAGAAGCGTCGAGACTGTTCGAGTCAC
TGTCGACTCTCTGGGAAGAATACAAGGGAAGCTTTCTTTCCGCGACAACGTCTTTTTTTGGAAGGACGGTGTTCTTCATG
GAGAGTACGTAACAAAAAAAGTCCAGGGAGAGAGCATGTACCTCCTTGTGGAGAGAGGGTCGTTCGACAACGGCGTGCGC
AAACCTTTTTACACGTCTGTCCAAGTGCGCGCTCCAACTTATAGTTTTTGAATAAAAAACTATAGCTTCATAACAAGAGA
AAATTTTCCACGATCTTTCCTCTTCCCGACAAGCTCTTCCGACTCTGGACTTTCCTCTCTGTTCAAAAAAACGACGTAGT
CGACTCCCTCCAAAGAAGTTGGGACAACCGGAGTGCAAAGGATTTCACAGCTTCCAATGGGACAAGGGAGACTTGGAAAT
TTTGAAATCTTCTCGTTCTTGAAACAAAAGCACGACTCGTAATACGGACTACCCATAATTTTATCGGATAACCTTTCCCA
TTTTGGAGAATGAAGAAACTCTGCGGTCTCTTCTCTTGAGCCGATGAGAATGCACTTTGCCATATATTTTATCCACAAAT
ATATTTTTATTCGAAAACATTGTCGTATTCAAACTTTCGTGTCCCCGACATCACAGCTTCCAGATCCTTCCCGTGCTTTT
CGCAACATCTTCCGATGACGCAGCAGTCATTTTTTCCAGAACCCTCTGAAACTGGTTCTCCATTCGCTTCAAACTTCCAG
TAAGACTCTCCGTGAAACCACGAGGAAGAATAGAAAGACAATGGAAGTTTGTTTTTCTTGCATTTCGAGATGAGCGTCCA
CGGATCGACAGAAACATCAGAACAGATGTATTTTGAGTATCTTTGCCTGCGATACTCTTCTGCTTGAGTGACACAAAATT
TTTCTCTTGTTTTCCTTGTTCGGAAGGAGCGCCACTCTTTGAGGTATGCTCTGTTTTTGTCCAGGATTCGTACTCTTTTT
TTTACTCCCCTGAACCATGTTGTCCATGTCCCATAATTTGGAGTGCAGTCTTCGAAAGAGCGAACGACACCATGAAGCTT
TCCCCTCCGGAAGTTTCCCTCTTCGATGAGAGCATGGTCGTACGCCGTTTCCATCACACAACGGAAAGGTCCATGGAGCG
CTCCCTTCTTCACTGTCCAAAAACAAGAAGAGCCGTACTCGCCCTCGAATTCGAGTTTTCCCTCTATGCGACCAAAGGGG
TCAACACAAAATTTTGCTGTCATGTCTTCCCTTTCCTTCTCAAGAGGTTCGTTGTTCGGGACATACAAACCTCCAAGTTT
TTCTGCCACAATATTCCCATCGCATTCTACTTTCCTCAAAAGAAAATTCTTTTGAGCGTCCGCGAGTTCTCTTGTCCACT
TGTTCAAAGCGCAGAAATTAACGACTTCTTTGGCATCAAGAAAGCTCAAAATATGAACATACATTTCTGGGGGAAGCTGT
TCCATGGTTTCACAAAATTATGTGCAAGTCTGTTTGCACGATGTCGATAAAAGAAAAGCTTGTGTTTCACAAGCTTTTGC
TGTCCGACGCAACAGTTTCCAGGGAAAAGTTTCGCTCTCATCTAGAGAGAAATAAAATATCGTCTTTGGGAAAACTCCAG
GGTTGACAAGTTTTACGCTGAGTCTCATCTCTCAAATCTTTCCTCAAAGAGAAAGCCTCTCCAAAACGTCTCGAAATTCA
GACACGCTCTTTTCTTGTGTGGCGCTTCCTATGGTAATTTTCACAATGTCCCCCTCTACAGAAATTTTCATCGGTTCGAG
AGACTCTGTCTCGACCTCTGTTGTGTATGGAATGCCAGTACGACAAGACAACAAAAGGAGTTGGAGAGCAACTGCGTTTT
CGCCAGTTCTTTGGAAGCTGTCCATCGCGTTGTGTGGGTTCCTGGCTGATTTTATAAAATATTTTGTTTTGCAAAATATT
TAGAGTTGTTCCTCGCATTGAGTCTGAAATCCATGAATTGTGGGTATGATTTCTTCTTGTTTCTTTATCGCAAAGGCAAC
GGATCGCCGCTTTTTACCGCTCTTTTTTAAAATTCTCGACTGTTTGCCGCCAAAGTCTCGAGTTCTTTGTGAACTGCTCT
CTCAAGAGGTCTTTGCGACCATTTTCGAAAAAGAGACCTCCGAGTGCCACCCGTTCGGCCACTCGATCGAGAAAAAGAAA
AATATTGTTCTGTAAATATTTTTATGAAAATATGGACATTCTCTTGCGCATCACAAGAGAGGATTTTGAGTCCGTCGGAA
CAGACAACGAAACGGACAAAGTCAAGGCTCTTTTGAAGCTTGTAAAAAGAGAATGTCCAAGCATTGGAGAATTTCCCCAC
AAATTCTGGTCTGGAATGGAGGCGAGAAAACAAAAAGTCGAAGGAGTGATGTCTGACGCCGACTATCCGTTCATCTCCTC
CCTTTTCCGAGAATGTGAAAAGTTTCGAGAAAAAAGAAGAGGAGAGCATGACGAGCAGATGACACTCGTTTCGTTCATGA
TATCCAGCATCTTTGCGAGGTTTTGTAAAGAGAAAGCATACGTTTTTCTGAGTTCCGACAAACCAACAGAGGAAGCAGGC
CTCACAATATCCACAAATTTCTACGAAGCGGAACTTCCTGTCCTTTTGCGGCTTGGTCTTCCCATTTTTGTCTCTCGTTC
CGTCGAAAGGATATGGACTCCATTCCAAGAAATTCGAGAGGACTCTTTCGACTGCCCGGTTTGGAAAAGATATTGGGACC
CTTCAGATGGTCCAGAAACAGAGAAAAGTTTCGTGAAGCAAAAAATGTCTCCAAACGAATGGGATGAATGGAGGCTCGCT
CCTCCGAGAAAGCCTCTGAGTTGGAAGGAGCAAAGAGAAAGGATGAACTGCATCGCAAAGGGTTGAATAAATATTTTTTG
TTTGACAAAAAATATTAACACCAAAAATTTCCAGCTTTCTCTGGACCTATCCAGAACACCAACTCCACCAAGAAGCGCCT
CTTCGCGGCGGAGTATTCTCTGATGAGGGCGAGAGCAAGAGTTTCTCTCATAGAGGCCAACAGTCTCCTTTCTGTTTCTG
TCGGTATAAACTTGACCAGAGACCTAAAGATGGAGCACTCCTCGCGAAGACGAGAGAACAGAGTCAAAAAGTCCCCGTCT
CGTCTGGTCATCGCTCCCATAATGTCTTTGGTGGTTTCCGCAAATTCGGTGTATGCGCCGAAATGGCATCCTCTTAGCAC
AATGTACCTCGCCAGCCGAGGTTTTTGGACCGCTTTTATCTCTTTTATCGTCTCTTTCACTTCGATGGGAAGGGTTCCCT
TTTTGAGACCGAAAACTTGCACTGCCATACAACAGAGAGAACGGAGACTCGGCACTCTGCAAAAATGTTCCATCTCCCTG
TAAAATTATACTTTGAATGAACTCGTAAAAATATGTTTTTTCTCCTTCTGATATTCGGGAGTGTAAATGTTTTTGCCGAC
AAAACATTTCTTTGAAACACCTTCTTCCTTCCAAGGGAAGTTCTTTCAGATTTTTTATATTCCGTATCCCCAATATTTTT
GGTTGTGTGCCTGTTGTGAAACACTCGGAAAGGGACATTCTGAAAAAGAGAACCGCCACCTGTTGACAGCTCCGGGACAA
TTTCTAACAGGTTCCATCGGACCAGCTACCATGCAATGTCTGGGGTCGGCGTTTGGAATCTCCCCATCGGTTACGGTCAA
AACGCGAACGTCTCCGGCCATTCTCGAACTGTCCACGACGCGTACTTGTCTCGCAGGGGGAGGGCAGACGTTGCACACAG
CTTCCCATCCTCCTTGCTTTTGAACAAAAACAAAGAGACTTCCGTTTGCACATCCTCCTGTCGTCAGTTGGGACTTTAGC
CTTTCGGGGACGAGATTCCCCTGTTTATCTCTCACATCGCGAAAATCTGGATGAATTCCATCAGGACAGAGCACGAAAAA
TTCCGCAGGAGCCCAACCCGTCTGCGAAGCACCAAAAGCAGACTTCCTTGCTGCTCCTCGATAAAAAATTCCGTTTGACT
GCATCTTCTTACAAAAGAAGATGGACGACTTTTTGGAGAAAAGAGAGTTGTTCTGTTTTTGCATTTGTTTGGGAAAAATA
TGCGACACAGAAAAATATCGGATAAAAGTCGCAAAAGGAAACTGTGTGTACCATCTTCTCCCAGATGGGACAAGGGATGG
GACTTTTCAAGAATTTTGGAAGGATTCCTTTTCTCTCAAAAAACAGTCCGAGTACAAATTCGGAAATCTTGACGGACCTC
TTTGCGAATGGGCAGAGAATGGGGCTCTTTTATTTTCTGGAAACTACAAAACGGGACGAGAACACGGTCTTTTCACAAAA
GGGACGGCTTGTGTGTCTCCTGTGATTATGAGCAATTTTGTGGATGGCAAACTCCATGGAGAGTACAAAGAATATCACGA
CAACGGAGAGAAAAGGACGCTTTGCTGGTACGACAAAGGAAAGCAAACCGGAGAAGCAACACATTGGAACAAAGAAGGAA
AAGTCATCGGGAACGGGAGCATCGACCCTTTCGGAAACTACACGGGAGACAAAATATTGTGGTGGGAGAATGGCGTCATG
CAAGCCCATCACAAATATTCGAACGGAGAACTTCGCGGGTTGCAAATCTGCTATTCAGAGGCAGGAAGATACACAAGCAT
CCAACAATTTCACAGAGGAAAAAGAATCTCTGAATACAACTACAAAGGGTAAGATGTCAGCCCTACAGGCAAGGATTCGA
GAATATCAGAGACTTTTTCCATCGGTCAAACAGTTGAGAATAACACCTTCCAGCCGACCACAAAAAAGACTCAAGGCAAC
ATTTGTGCTCGGCGGAGAAGAGAAAGTTGTGCACTTTGGACAAAGAGGTGCGTTCACGTATTTCGATGGAGCGCCAGAAA

CAAAGAGGAACGCCTTTCGTGCAAGACATTCGAAAATTCTCAACAAAGGGAGAACTGCCTATAAAGTTCCGGGAAGCGCA
AGTTCATTCGCGTGGGTGATCCTGTGGTAAAATATTTTTTAAGAAAAATATTGAAAGAGTTCGAGTGTTTGTGAGACAAG
TTGTTTCGTATGTCTCTCATCAAGGAGTTTGAAGTCGGTGCTGGGGGAGTCGTCTCGCTTTTTAAAGGAGACGACTTTTT
TGTCTACAAACGTCCGCATCGTCGCTTCGACATCTCCCAGCTCGTCGAATATGAGACGCAACTGGCCCTTTGGAAGAGGT
ACAACGACGGCTTTGTGAAACCTCTCTTCTCTGAGATGAAGGATGAAAGACTCTGTTTTGCGATGGAGTGGTTTCAAGGG
AGAAGATTTTGTGTGGAGAGGGTGAAGGATGTGGACAAAGTTGCGAAAACTTTCTGTCGTTGTTTTCAGGCGATGGAAGA
TGAAAGAGTTTATCACCAGGATCTTGAATGCCGCAACATTCTTTGGAACGAAAAAACAGATGACGTAAAAATCATCGACT
TTGGGATGGTAGTCAGGTACGAGGACGACACACAACGAAAAAGGTTCGGTCCATCCTTCTCTTTCCCTCCCAAAGAGCTT
GAACTGCTCGATGGAATGTCGTGTCTGATGACGAGGGAAGAAGCATCAAAAGCACAAATTTGGGCTCTTGGAGATTTGTT
GCTTCAACTTCTCACAGCAAAGGATGAAGTCGAGAGGGTGAGATGGTGTGACGGAAGAGAGGCGTTCGAAGAGGATGTCC
GCAAACTCTGCGATGGAAAACAGGGATTCACAGAAAGGGCCATTCTTGCAGCTCTCCAAAGAGAGGCAAAAGACAGAGAA
ATTTTATTCGAATAAAATTTCTGAATGGGAAAGAGAAAATGTCAGAAGAGAACACGTTTGCGGTTTCGTCCGACTTCACG
AATGCCGAAATAGCAACTTACGCTCCGACCACTGTCGTTGGATTTCCCAACGAAGAGCTGTATGCAAAAGCGGAAAGCAG
AGCGAACGATGTCATTGAAAAAATTCAAAAGAGAATTCCAGTGCCTTATCCCTTGAACACCCAAAGAAATTTTCGTGGAG
TTTCTGTCAGCTCCCCTTCTATGAGAGCGTTTCATACGGACGGAGAAAAACTCTACGTTTGGGACGACTTTCGTTCGGAA
CTTTCCATCGACGAAGCGGAACTTGTCGAGTCTCTGTCGCAAAATATTCCGCTTTCGGCAGAATACCAGCCATATCTCAC
CAACAAACTTTTGAAAGACCTCAACGAGAATTTTACAACTCTCTTTTCATGGCTCAGAGAAAGCGTCGAACTCGTCCCTG
GTTCGGAGCGAGTGCAAAAAATTCAAGAAGATTTCGAGAGCAGGCAATAAAATATTTTAGATAAATTCAGATAGAATCCA
ACCACAATAACACGGAATGCAAACGTCTTCCTCTCCGACAGGCATCAACTTGAAAGGTTTGCATCGACACTTTTGGCATT
CTTTTTTGTCAGACTCTTTGTTGCACTTGGTACAGAGCCAAGCAAAGGACGGTTTCGGATGGAACTCTTCTCTGCATTTC
CAACATTTCGGAATCTCTTTACAATTCGACATTTTTTTTCTTTCTTCTGACAAAGGAAAAACTTATGGAAGAGTTTCTTG
ACGATTCAGAGCTCGCTTCTTGTTGTGTGAACGATGAGGGGCTTATCGAGTCTTTGAAGAAAAGAAAGGAAAACACATTG
ACAGTGTACGATATCAACAGTTGGAGAAAAAGGCGTTCTGATGAGTCAAAGATGCCAGGAATGAACGTCCTTTTGAATGG
CGTTCATTCGAACCTTTACACCAACCAGAAATTCCCTTCTTGCAAAAGGTTGTTTGCCAAAAAGTGCGAAAAGAATTTCT
TCTATCACCAAGTCAGCAAAAGGAACTTCCCAAATGTGGAGGAGCTGTGGATAGGCTCTCACCCTTGCGACGGCAAGGTG
TTTTATGAGGGGTTTCCAAAAATTTTCATAACACCGGAATACTCTCACTACGCAAAGAGGTGGGCTCCTAAAAATTGCAC
GTACGTCTCCATCACACAACAAGAGTTTGACAGCGCCGTTTCGTCTTTCGACAAACAAGAGCTCCAATAAACTTTCAGGC
AGAATAAATATTTCAAAAGAAATATTTGAAAGAGATGCAAGGTTACTACATTGTGTTTGCCTGTGACCAAAGAGGGGGTA
TCGGAAACAAGGGAAAAATTCCTTGGAGTTTGCCTGAAGACCTCGCGACGTTCCAAAGGATGACATTTGGAAGGACCATA
ATTTATGGGAGAAAAACACTCGAATCTTTTCCTGACTCAAAACCCCTAGCGAAAAGAAGGAACATCATCGTGTCGAGGAA
CAAATCTCTTCGAGTGGAGGGAGCAGAGGTTGCGCACAGCATCGAAGAAGCCTTCTCTTTAGCGCAAGGTTACTGTGCTG
TCGTTGGTGGCTCTGAAATTTACGCGCAATGTCTGGAGCAGTTTCCCGAGCTCTGCCTTGGCGTTTCGAGAACACTGGTT
TTGAGCAGCCACGAAGTGGACGCTTGGTTTAGCGTCAAGGAGACGAAATACAGTTATCTGATAAAATCCTCCCATAACTT
TGAGACTGCAATGCTGTTCCGGAAGAACGAAGGGGAGATGAGCTACCTTTCTCTTTTGTCTGAAGTTCTTCACTTTGGCG
AAAGGAGAGACGACAGAACGGGGACAGGAACGAGGAGTCTCTTCGGCAGACAACTTTCGTTTGAAAACATTTCAGAAAAT
TTCCCCCTCATCACAGTCAAGAAGATGGCGTGGGGGTGCATTCTCTCTGAACTTTTGTGGTTCCTTTCTGGGTCAACAGA
TTCAAAGATGTTGGAACTTCGAAACAACAACATCTGGAAGAAAAACAGTTCGAGAAAGTTCTTGGATGGAAGAGGTCTAA
GTTATAGAGAAGGAGACTGCGGTCCTATCTACGGGTTCCAATGGAGACATTGGGGGGCAAAATACATCGACTGCGACACA
GACTATCGAGGCAAAGGAACGGACCAAATTCTTTCCATCCTTTCGCAGATAAGGGACGAACCGACTTCGAGGAGGATGGT
GCTAAGTGCTTGGAACGTCTCTGACCTCGACAAAATGTGCCTTCCACCGTGCCATTCTTTTTCACAGTTTTATGTGAGAG
GACAGTTTCTGGACTGTCACTTGTATCAAAGATCTGCTGACCTCGCTCTCGGCGTTCCTTTCAACATCGCGAGCTATGCT
TGTCTTTTGAGCATTCTCGCGAGCGCTTCAGGAAAAACAGCGGGGAACCTTACCATGTCGTTTGGAGACGTTCACGTGTA
CGAGAACCACGTCGACAATGCGCACAAAATGACAGAGAGACTTCCCCATAAAAGTCCCAAGTTGCGCGTCCAAATTTCAG
ACGAGACTCTCTTTGGTCTTTCAGAGAAAGATTTCACGATGGAAAGGTATTCCTCTTGTGGCGCTCTCAAGTTTGACATG
GCAGAATAAAATATTTTCTAGAAAATATTTTGAAAGAATCTGTCTTTTCTTCTCCTGGAAATTTCCAAGAGCTTGCATAC
CTCGACGTCGAACTCTTCTTTGATGCTCGAAGAAGAAACTCTCTTTCACCTCTCGACTTCAAGCATTTCCTTCTGAAAGG
ATGAGCAAAAAAGTGAGAGTTCTTTGTTTGTGGCGCAACCCGGTGTCCTTTGTCTTCTTGGCCCATCCTCTTCGCGAGTT
ATTGTCTTCTGTGCGTTCAAATTCAGAAACAAGCACCTTCTGTATTTTATAAATGTCAAGAAACAAAAGGTCAAGGAGCC
GATATAAATTCAAACCCTTTGTCTTTCCGTCTGGTAAAACTGTATGGATTCAAGGGTTTGAGGACATCTATCTTGTGTCC
CTACTCGAGGAATTCAAAGAACACGAAATCCTTGTCTGTGAGCAAACTCCGTATTTTGTTCCGTACAAATGGTATGGAAA
GACCAGAAAATACCTTCCGGATTTTTTCGTCCCTTCCCAGAACCTTTTTATAGAGGTGAAGAGCGACTTCACCTTCTTCA
AACGAAGAGGTCAGGAAAGAAAGACGAGAGCGAAACTTGACGCTTGCAACGCCCTCGGTTACGACACGAGACTCGTTGTG
TACAAGTCAAGAAGTTCAAGAAAAGTTTCTTTGTTTGAGGAAAAAGAGGGAATCTTTCCCCAAGATTAAGGATGGCATTT
TCTCGTTCAAGACAAGACAGAAGACCTTTGGTCGTCGAGACAAAACTCCAAACCAAAGAAGACCGGACTTTGGCGTTTCA
CGACGCAGTGGTGTCTGCTTGTTTTGCTGGAGGAAAAAATATAGAAAGGAAGAAGAATGGCGTGGTTATCACAGGTGGTG
ATAAAAAGCCAGAGTATGTTCTTCATCTTTGGATGTCAGAGATTTTGAAAGATTCGTCAACGTTTTTTTACGAACCTCTT
ACTGGGTTTGTTCTGGAGGACTACGCGACAATGAAACCTTACGACTTTTCTCAGGCTCTTAGGGTCGCTAAACACATGTG
CAAAGCAGTCGCTTTCATCCACGAACTCGGCATCTGCCATCGAAATTTGACTCCAGACGCGTTTGTTGTCGTTCTTCAGA
ACGAGTACAAAAAGACAAAGACAACAACAAAACTCTGGAAATTCGGAAACTCGTGCGCTGTGAAGAAGTGGGGTCTTGGC
GAGTGCATCTCTCCGCCAAAGTCTCCATATTCCGCTCCAGAGACGCTGACCAACGAGAAGAGCATGAGCAGACTCGACTG
GATTTACGCAGACAGATACAGCCTGGGAGCATGCCTCGAATATCTTTTGACGTTTGGAGGAGAAGAGAAGTTGCGAGTCA
AAGAGATACACAGTTTCATAACCTTGATGAAACAACAAAGAGCGAGGAGAAGGCCTTCTCCTCTCAAAGCCATAGATTTT
TTCGAACAACTCTAAAATATTTTGAATAAAATATTTTTTCTCTGACTTGGCATTGTTGGGTCTAGAAATGTAAGAGGATG
GCGTTTTCTGTGTTTGTGAACGCGGACGAAAGTTGTGTGCTGTTTGGACTCGAGAAAGGTTTCAAAGTCTACGACACGGA
AACGCAAAAGCTTTTGCTTTGGAGGGATGTCGGACCTGTGACGATGGTGAGAGTTCTTTCCTCTTCAAACATTTTTGCGT
TTGTTGGGTTTGACAAGAAAAAGCTGACGTTTTGGAACGACGAGACAAAGAAGAGAAGCGCAGAGATTGCTTTTCCCAGA
GTCATCACGCAGTTCTTGTTTGGCAGAAGCAAGATGGTTGTTTCCACAGACGAAAAGACGTACCTGTACGACCTTGAAAC
TTTGAAACTTTTGGGAGGATACGAGACGACACAAAATCCCCACGGAAGCCTTGCAGTCAACAGCGACAGAGCGCAACACG
TCTTTGCTTTTCCTGGAATGAAACAAGGATATGTTCACATCATTCGAAACGGCATATCTTTGTTTGTTAAAGCTCACGAG
GGAGTTTTGAGAGTTTTGTCCCTCAATAGGGAAGGAAATCTGTTGGCCACGACTTCAGAAAAGGGAACGGCCATCCGCGT
GTTCGACACGACCTCTGGAGAAAGAGTAGCAAATTTCCGAAGAGGAAAAACAGAGACAAAGATTAACCACATTTCATGGT

CAAAAGATTCAAAATTTCTTTGTGTGAGTTCAGAGAGAGGGACGAGTCACGTTTTTCGAATGGGACAAAATTTCCAAAAC
TCTGCTCTTTCTGGATTTCTCCCCGACCCCTTGAGTGACTATGCGAGTTCTGAATCCTCTTGGTCTCTTGCGAGATACCT
TCATCCAAAGGGGATAACTCTCGTATCTGAAGGTTCCTTTCTGAGACATTTCAGCGTGGACGAGTACGTTTCTGAATGCA
TCGTTCCAGAAAACGGAGGGGAAGCGCTGTTTTTGGAACAGAGAAAGATGTGATATTTTTATCCATAAAAATATCTAAAA
TTTGAGAACTGCCTTGAGGTCCCACATTGTGGAGATGAGATTGACATGTTCCTTTGCCTTTTGGGGCGTGAGGTCAGATT
CATCCATTTTTATCTGGTAGAGGAGCTTCTTTTCCGCTTCTTTGAACTCTTCTGGAGTTGGGAGGATTTGAAAGTAGCTC
TTCTGTTGCGACATATTACTTTTGGGAAATTTTGCGAAGTTTCGGGTCTTCAGAAAGAAAGCCACATATTTTCTGTCTGT
TTTGAACTTTTTCGAGAGTTGCTTCTTCGTCTCCTGCGAGGAACACGTGGTGGGCGTGTTCGCAAAACTCGAGCACGCAG
CCGAGAGCCCACCAGTCGATGTTCTCGTCCACTTCTTCGTTTCGAAGAGATTCTGGTGAGCGATATGCGAGTGTGGTGTT
TGGAAGTTTCAGAGGAGAGCGCTGAACGCTATCAAAATCTCCAAGGCAGATTTTACCCTTTGAAGAGACGAAAATGTTTT
CTGGTTTGATGTCGCAATGAGTCAATCCTTTTTTGTGGAGGTATTCTATGGCTGAAAATATGCAAGTCTCGACTCTTTTT
ATAAACTGTCTGTTGCAAGGGCCAAAAGTCGTCACAAACCTGTAAAGGTCAAAGGGGTGCCATTCCAAAACAAGGAATCT
GTTCTGGAGGCCAAAGCAGGTGAGGATGTTCTCATGATGGGGAAAACAAGCGTAAAAAGCTTTTTCTTCCTTTTCTGCGT
CCTTGAGAACTCTCTTTGTTTTGTGTAGAACGTCAAACACTTCTTGGACGAGAGGAGAAGTTTGTAGAATGTCAAGGAAT
AAAAAATTTTGCATCTTATATATTTTTTATATTGTCTCAAGCCTTTGTTCGAGATTTTCGAGAGCGACCATTTCAAAGAA
ACACCTTGCGTCTCTGGTCTGTTCTCTCCAAAAATTTAAAAGCTCTGGAGAGCTTCCAAAACTTCCAAACAGAATGTCAC
ATCGAGAAACGAGCTCTCTTGGGTTTTGTACTGTCCCTATGTCCCCAGAAATGTCCAAAACGAGGATGACGCCATTTTTC
GTCTCGATGAGATGCTCTTCATAGTTTTTTGTTGGGACATGAGAACCACAAAATGCGCCGTTTATTTTCCACAACTCTCT
TGTTTTTCCGATGCCGCTGTCTCCCACAAGACCGACAGTTTTCTTTTGTTGTTGTTTTCGTCCACAAAATAATTCGGAAA
ATTTTAAGGAAAACATCACCCTTAAAAGATAAAACAATGTCCTTTCGAAAATTTCTGCACTGAGAGGACCTTGTCGATGA
CCTCTCCCACAGAATTTGGATCTCTGCAACTACAAAAAAAGTCTGGCGACACCGCGGCTTCAAAATCCGTTTCTGGAAGG
TCTGTTTTGTTGAGGATGACAAAAACGGTCTTCCCCTTTCTTAAAAGTTCTTCGCGCTTCGATAGAGCGTAAGTGGCAGA
TTCTTTCAAATCCCTGGACATAAAAACCAGAAAAACATCAGATTCTTTCAGGCAGACTTCTTTTATCTTTTCGTTGGATA
TGGCACAAAAGTCCCAGATAGAAAGTGACTTTTGCTCTCCTTTCACCGTTTTCGAGAAGCTAAAACAGTCTGCGCAACAG
GGCTTGTAACTATCCACAAATTCCTCTCCAGAGGCCACACGGCAAAACCACGTCTTTCCCACGAGCGGTTGTCCCAAAAC
AGATACCATCAACGACATTAAAATTTGTTGAACAACAAATTTTTAACGAGCCGCTTCCATCGCGACGCAGAGAGCAAAGA
CAAGGCACAGCCACGAAACGCACACAGTCCCAAAAATCATGGCCATCCAAAACTCTTCGAGATTTTCGTCGTCTCCGCCA
AATTTGAGAGTCATCCAACTGCAAAAAATTATGGTTGAAAAGAACCACAACAGAAAAGAGAGCAAAGGTTTGAGGCAAGG
CATAAATAATATTTTCAAAAACCAAAGACAAAAGTAATGGCTACCAAGACAATTCTCGCTGATAACCTTTGCGTTGTTGA
TTCCGAGGACACCTTTCTCCTCACCATCGATGGAAGTGCAGTCGCTCGCATCTCTGGAAAACAGGACGCGGAGAAGGCTG
TCATCGTAATCGTCGAAAAGATTGAAGGAGAACTCAAAAACTCTTCCACCAGCGTGTATCGAGAAAAGATTGATGGCGGG
TTTGCCATTTTCACTCGCAGCCTCGGCTATCTGGTGAACGGTTCTCCTGTTCAGAAGCACGTCGTCAAGTATCTTTCCCT
TCCCAGTCTCGTATTCGAGGAGAAGGAAGAAGAAACTCCCAAGATGGAAGTTTCTGAAATTCCGACTCCTCCTCCCGCTC
CAGAGGTCATCTCAAAGCAGCTCCCCGAAATTCCGAGCGTCGAGAAAGTTGCTGAAGAGACCAGCGTCTCTCGAAACACT
TATAGCGTGGAAGGGCTGATTCCGCACAACAGCACCGGCCTCTACGAGCCCTATTCTTCTGCCTACTAAAAGATTTTTTC
CAACTAAAAAATCTTGTCGATGTCGCCCTCTGACATGGCCGAAGGCCAGACGTACTCGAGATCCGGATTCTGGACTTGAA
ACTTTTTCTGGTAGAAATCTGGCTCTTTTCGGAGAAGTGACGCTCTGTGACTTTCGTGAAACTTTTGATATCCCAACCAC
CAAGGAAGAGGAACAGTTCTCTGTACGCGGATTTTCTTTTTTGGTAGAGAATGCTTCGTACCTGCCTTTGTGCTTCTCGT
TGAGAGCTCAGATATGCACGCATTGATGTACTGCCGGAGCGCCCTCTCATAACCAAACCACATTTTCGTGGCTGGGTGAC
AGCAAAAGCCAAGATTTATTTGCAGGCCCTTTTTCCCGGACGAGATGTCCTTCCCTCTCAAGAGAATTTCATAGGTTCTT
TCAAATTTTTCTTCTCTTGCGCGGTATTTTTTAGAGAATGTTTTTATCCATTCACATCTTGCGACAAAGGACTTTGCGAG
ACATTTGTGGTCGAGCAGAGGAGGAAGTTCGCCGAGTTCCACGGAAAGAAACCAAAGGTCTTCGAGAATTGTCAGGATCT
GGTACGCTTCGATAATCTGTTTGTTGAGCCTTCTGTTGTCCAGAGTTTTTGCTGTTTTTTCGAAACTCTCAAAGGGGAGA
AACGTGTTGACCATCGTTAGACAAAGAACAAGAAAACTTTTGCGTTCGCAGAGCATCAATTCGTAATGAAACTCGAGGGA
ATTCCCGTCAGAATTCTGGAAGAAAAAATATTTCTGATTCAAAGAGAAATATGGGAGCAACAAGTGTACGGAAACTGGCT
TTCGTTCCTTTTGGATGGGAACGAAAGGGAAATTCCAGAGCCTCCACAAGAGGAAGAAACGCGAGAAGTTTCTCTTTCTC
AGGTTCTCTCCTCTTTGGTAGAAGTTGAAAGTGAAGAAACTCTCGCTGATGTTCTGGATGCTCCTTTGGTGAAAATTCCA
CAGTTTGACCCCTTTGAAGTGATAAGCGAACTTTCAAAGATTCTTTCAGAATTTATTTCAGAGAATAACTTTTTGTCTCT
GTTGGTGTCTGAAATTCCAAAGCCCTCGTGGTACAAAACATTTGCAGAATATAAGAAGAGATGAAGCTCGCAAACGCGTC
CTGTTTGAGCATATTGAAAGAGTGCGAAAGAGGCGCTGAAACTCTCGAAATCCAAGAGATGGACGCAGAACAACTTGCTT
GGCTTGTCGTGTACGGCACAGGCGACACACACGTGTACGTGCAAAGCGTGTGTTGCAATCTCGATCCATCGAGATTCATG
AAGGGGGCTCCCAAAAGAGAGACGATACATCTCGTCTCTCCAAAAGACGATTTCGTCAGAGAAATATTTGCTGGACATCT
TGTCCTTGAATAAATTTTTATCTATAAAAATTTATGCTTGCTCGAGTGCGATGAAAGGAATTCCCGTCCAATACGCTCCG
AGCGCCAACAAAACGGTGAAAACAACAGCGACCAACGTCATCAAAGTCAGCGTTTTTACCCTTGTGTTGTAGAACCATTT
GTCGAGAGCTCTTCCTGCTATTTCCACCCACAAACTGGAGACGAACCACGCCGCAACAAAGAGCATGAATACGACAACAG
GGCGGAACGTTATCTTTTGTGTTTTCGGAGCTTCTTCGACTGTGATGTTAACGTTCTCTGGATTTATTTCTGTTATTTCT
GCTTGGGGGTCGCCTTCAAACTCAAAGTTTTCTCTCTGTCGCGTCATGCCATAGTGTCTCGAAACGCTGTCAGAGACGGG
AGTTTGCGCTCCGACGTCGTTCTCGACGAACGAATTCGCTTCGGGTTCGTAGAGATTTCCCATGACCGTTTCATCTATGG
GCTTTGAGTAACCATAAATGTCCGAAGAAGAAGTGGGGATGTACGTTTTCGTATAGTCTGTTTCGTACATGCTGGGCATT
GAGCCAGCATAGTTTCTCGAATAACTTGCCTTCTCGTCAAGATATGGAACTGGTTCTTGCGTCTCGCTCCCAAGGAGGGG
CAAATATGTTCCTGACATTACTAACAAAACCTTGACTAAAAAATAATGATAGGACTTTGTTGTCTACTTTTTATCGCTTC
ACTTCTAGTGCTCGTTCTGTTGTTTTTTCTCGTTGGTTTCTTCTTCTCGAAAACTCCGAGAGAGAATTTTGGAGAATTTG
ACAGAACAGGTCTTAAAACGGGAGATGTTCTTCTTCTTTCTGGGAAGACGTTCCCAGAAAGAATAATCTGTTTTTTGACA
AACTGCGAATTCAGCCATTGCGCCATGGTCGTCAAAAAGGAAGGGAAACTTTGGCTTTGGGAAGCTGACATTGGCCAGGG
AAAAAAGAAAGGTCCGAGACTCATTGAATTTGACCAGAAACTCAAAAGATACAAAGGATACCGCACGGCGGCGATAGTGA
GGATAGACAAAGAACTCGCCATTTCCGAATTTCTAAAGGGAGCAGAGAAGCACTTCAACAGGCCGATGGACGACGCGATG
TTCTCATACTTTTTTGGAAGGAACAAAAAAGACGACTCTGTGTTTTGTTCTGAGCTTGTTGCTTCGACACTAAAGGACTC
TGGAAAAATAAAGAGAGACAGAGAAACGTGCAAAGGCGTTTCCCCTTCACTTTTTCTGAAAGGTTTTGAGGGAATATACG
GTCGACCAGAATATTTTAGTTGGTAATGGATCAAGAGATCGGTCTTTGGGTGGAAAAGTTGCAGTACATTTGGCTGGAGA
AAACGCTGACACGCCTGTATCTTATGCCAAAGAACTACCCCGTCGTCGACAGCGAAAACAATGTGGTCAAACTGTCGAGA
TATGATGGAGACTGCTGGCTTTTTCCTACCACATTGAGCTGTCCATCAAAAAATCTTTTGAGAGTGGCGGACGCTTGGCC

AACAACGCCGAGAACTCTTTACAAAAAATATGGACCTTTCAGTCTCAGGATGGAAGGGAGAGAGCAAAAGATGGTCAAAA
TTCCTCGTGTTCCGGAGCTTTATTGGATGGGGAAGTTTCACAAAACTCCAATTCCTTCGCAGTTTGTGGGGCAAAAGCCA
AAAGACGTGAAATCACCTCTCGTCTCTGTGAAGAGCGGAGAGACGTACAAAAATTTATGGGAGTTTGAAGCCAAAACAAA
GGCGTCGAAAGACACGATCGCAAGAATCCATCAACAGTCTCTCGAAGGAAAGGGTCCGTACAGATATTTTTGGCAAACTT
TCAAGGACAAACAAGCACAAAAACTTGGCAGGCAAATCTGTCTCTCTTCCTTTGACTTTTGGTGCTTGTAGTGACCACGT
TCAGAAGAGATGCGAAACCGTTCCGTGAAGCGGTACTTTATCGAGTCAGAGATCCGTTCCGTAAAAAAGTCTCGTTCGTG
TCCTGATAAAATATTTTTTGATAAAATATTTAGTACGAAGGGCTCATGCCTTGTTCTTTCTCGTACTTTGCGAGTTCCTT
CTTGCTAAGTTTGGCGAGCTGACGTTCGCTCAAGCTCTTTTGAGATTTCTTGGTGGGCTTTTTAGCAGCGGAAGGCTTTT
TCTTGGGAGCCTTTTCTTCGGTGGTCTTTCGCGGCTTTCTCCTGGGGACGTGAACCTGGACGAGACTGACACCGGCACCA
GAGAACACGCCCTTTCCTACAACGTGGAAGAGAGCGGCATCGTCTTGAATGGCGCGCATGATGTGCTTGACAGAGATACG
AACTTTCTTTGAATCTCTGGCGGCGTTACCCGCAAGTTCAATAATCTCTGCCATAAAATATTCGAGAGCAGCGGCAAGAG
CGACAGAGGCACCTCCGGAAACACGGCTGCAAGCGCTGTGCTCACGAAGGACGCGCTCAGAACGAGCAACGGAAAGCTTC
AGCTTGGCCTTTGAACTCCTTGAACCTTCGGAGACCTTTGACGCTTTGTAGCTTGCGACAGCCTTTCTCGCTTCCGAAGC
CGCATGCCTGCCGAGGTCCTTGCCAAAGAGGTCCTCTGCCGAGAGAATCACAGCTTCAGCGCTGATGGTTTTGGTACCCT
CTGGCAAGAGGGTGGCAGCGCCATGTGAAATTTTCTTTCCCACGAAGACGGTGATGTTGCTGAGTTCAGCAAGAGCTTCG
GTTTGAACAGAAATGTCGGGATGGATCTGAGAGAGCAAGCTCCTCAGACCAAGACGGAAGTTCACGCTCTTGTCTCTCTT
GCGGGTGGTTTCCTTTTGTGTCGCCATTACAAAAGTAGTAAAAAAGTAAATATTTTGCAAAAAACTCACACAGAAGCTTT
TTTACTCGAAAAATTTTTCGTTCAAAGTTTTTCTCTGTCGCAAACTGTAATGTCAAAGGCAGCCAAAAAGTCAAAGTCTC
AACAGCACGGTCATCTCGCTTCTTATGTTCTCGGAGGGGAAACGCAAATCCCCAAGGCCACCACGCAGCACCTCTTGCGA
AAGGCCGGTTCTCTCTCAGCGACCGATGACACTGAAGTGCCCATCCGAGGTTTTGTTCGGATGAAGCTTCACAAACTTTT
GCAAAAATCCCTTCTTGCGATGCAGCTCGCCAAGAGGAAGACCATCCTCAAGGGAGACGTCAAGAAGGCGGCGGAGCTTA
TGCATCTGCCTGTTTTCGCAATTCCCACCAAGGAATCTGGTGCAAAGGGCTCTGTTTTCCTCTCTTGTCGTCAAAAGGGT
GCCGGTTCTGCCGGTTCCGGTTCAGACACCAATTCGCAAGAGGTCAGAAACCAGATGAAGTCTTCGTGCCTCATCATTCC
CAAGGAGAGGTTCAGGACGATGGCAAAGGAAATTTCAAAGAGGGAAGGTCACGATGTGCACATTGCCGAAGCCGCCCTCG
ACATGCTCCAGGTCATTGTCGAGAGCTGCACTGTCCGCCTTCTCGAAAAAGCACTTGTCATCACCTATGAAAAAGATAAG
AAGAGGGTCACCTCGAGGGATATCGAAACCGCATTCATGCTGGAACATGGTCCGCTATAAGCACAAATACAAATATAAAT
AAAATGTCGAGCATCAACTCCCTTCCTGACCTCATCGGCATGATGAAGAGTTCCATCGACAATGACGTCGCAGAGCTTCA
AAAGAAACTCGCTCAAGTTCGTCTGGAGAAGCTTCAGATCCAACAACTGCTTTTTATGATGCCTTTGACGACACAAGAAA
ATATTTTCGGATAAAATATTTTATTCTCTTCTCTTTCCTGTCAAAAACTTTCTTCTGGCTGAAGAAAACATCTGCGCTGC
GTCCGCATCGGGTTGAATCTGCGCCATCTCACAAGCCACAACAAACTGTGACAAGAGGTTCTGTTCTTCCAATTCTCTCT
GCTGTTTCTTCTTTTCTTCGAGTTGTTCCATCAACTGCTTCTCAACTTGGCCCAAATGGCAAAGTCTTTCCTGGAGTTGA
CGGCGAATTTCTCTCGTTTGTTCCTCAGACGCTGTCAGAAATTTGCCAAGTCGAATCAACGGCTTGGTCGCGGCTTTCTT
TGGGCTCTTCTCGTTCGTGGTGCTTGACTTTCTCTTTCCGAGAGAAACGGTTCCGTACTCGTAGTGGTCCATGATATCGC
ACTGCATCTCAAGTGTTGTATCGCACAAAAGAAAAAATCCCTTTGCGTTTTTTTGTGGTCAATTCTTCCAAATAATATTT
TGTATCAAACAAAATATTCTAGTTAATCTCAATGTCCTTCTCGTTCAACCACGAAAGCTTCTCGTAAATTTTGCGTTTCT
CCTCCATCGGAATATCGGGCTTGTCCAACTGAATTCGAAAATCTTTGGGAGTGAAACCGCGAACTGCTTCGGCATTCGCA
TGTCCCGCTGCTTGGAGAAGGTCCTCGTTTCCCAAAAACGTAGAGAGTTCATAGAGAAGGTTGAACCTGTCTGTCAGCCT
AAAGTTGTCCACGAGTTTGCAGTTCTCGCGTTGGAAGGAGAGAACCTCTTCCTTTGTCTTTTCCCTCTGTTCGTTCTCTT
CTTCAGTCACAATTTCCCCATAGTTTTTCAAAAGAATCTCTCGAAGATGGGGAGCAAGGTCCTTGTATTCTACTTCCTTG
TTCTGTTCCCTCCACAGCAAAAAGTCGACTGCCGTCTTGCAGTAGAGGTAGTGAAAGGGAAGGCGAGTGCACTCGCCATT
GTCGGGCTCTCCTTCCTTGTAGTTTTCTTCCTCGAGTTCAAAATCTTGGAGGGAAGAAAGAGCAGTACATCCCCCAAACA
AGGACTCGGAGACGTGAAACTTCCTTCCTTGAGGTTCAGAAGAGACAATCTCCGGCTTGGGACAGAGGATGGGAGTATAG
AATCTTCCAATGTCCTTTCGTTCTATGGTCAAAGAGTCCATTTCTGCCTTGTACTCTGCCTGTTCCTTTGCAAACTTCTC
CTTCTTGCCAGAGAGAAAAGCTTCCCAATCCTGGTCCTTACCAAAGGATTTGTAGAAATCAGACTGAGACATAACAGAGA
ACTCAGGGAGAGCGGGAGGGTCGACATATTCTCCTTCCTTGTTGATGTACTTCTCGGAAGAGACGAGAACAGAGTCGGTG
GACGAATACCAGATGCCAGCCATCGCTTTTGATGTTTGTTGTTGAGAGTTCGAATGTTGTTTTGTCGAAAGAAAAGAAGT
TGGGTTTTCGATAAGTAAGAAGATGTTTCAAGGAGGACCAAAGATCACAAATTTGGGTGCGTACGTTATTATGGATTGGG
GACCCGGAACTCCGAAACGCTATACTGGTCCCGCATATGTCGGAAGGTCTTTCGAAGCGCCGTCTTGTGCTCCGCCACAA
TATGTATACAGAGATAATATTACTCGTCCGTGGTTGCAACCATCAGTCATCGAGATCGGAAGAGGAAACATCGACAACAT
TTACGCCTGCAACTCAGCCTATGGACCGATAAATCCTTGGGACAACCTCGCGCCCGACCGCGCTCCATGCAAACGACCAG
AAAATGATGGCATAGGGTCGACGAATTGGCGAAAATGGTGTTAGATATGGGACTTTGTAACCATAAAATATCTTTTACTT
CAAAAAATATTTAGTGTGGACAAAAAGGCCATTTTTTAGGGTCAACACTCGATGGAAAGCGTTCCCTTTCGTTTCCGCAA
ACGTCTCTGACCACTTTGTTTGCAGTTTTTTCTCCGTATACGTACTCAAGAAGAGAATACGCATCGAGCTTTTTCTTTCC
TTCTGCTTGGGCTTTCATGTAGAAAATTATGGCCTTATGTCTCTTTTTTTGGATATGCCCCCACAAAAGAGGCGCATAAT
CAGACTCGAAACAACATGCGTGACACCTCATTATGCATTGCAGTCCCAGAGTTTCGTTGTGCTCTCTCATGTGCTCTCCG
AGTTCATGTTCTTTGGAAGTTGCGAACCCGCAAGTCTCGCATTCGAAAAGTTCGGGATTTTTATTGCTTCCCATTTTTAC
ATACGTAAAATCTTGGCTAAACTGTTATTTCGAGTTCGGAAATTTGGGAAATTCTCGTCTCCACCCTCTTGGCGAAAGGT
GTCGCTTTTCGCAGACCAAAATATGCTGATGTCAAAGAAGAAGAAACCTTGTTGCTTCTGTTTATGAAAGAAAGATAGAA
AATGGGAGAGTTGTCTTCCGATGCCCCAGGAGATACAAAGGGAAATATTTTCGTTAGAAACGCAAGGGTCAAAACATCGC
CACTGTATTCCTTCTTCGTCGTCACAGAAAAACTTGAGATAAAAGACATGAACTTTTCTTGTGAAAGAGTTTTTCTGTCA
GCAAATGGAGGCGTTTCACCATTTTTAGACAAAATAAGACCACGAGCACACTCAGTCACATCTTTTTTAAGTTTTTCTTC
CAAGAACCACCATTTCCAAAAAGACTCGCAATGGACGAGAGAATGATGGGTGTTCTCTGTCGACATATTTTTGTGTTTTA
TTGATGCTGTGTCGAACCCTACATAAAGTTTCTCATCGACGCAAAGCTCTGAGTTCCCGTTTTGTGCTTCTTTTTCGCTG
AGCTCCAACCTTTTGCTTCTGATGCAACAAGCCAACGTCAGAACAAAAGAAAAAAAGTCTTTTTTGGACATGATATACTT
GTGGTTCGTCCACGAAACCATGCATCTCCCTTCTTTTTCCTCCACAATGTCTTGAACGTGCTCAACGCAAGACTTCATCC
AAGAGAAAAAGACAACATCGTCGTTGGCGTCCCTGTAGTTTTTCTTTGGAGATATCCTATTCAAAAAACATCTTTTCTCT
GTTTCGAATGAAAACACTTCGTTACGCAAAAGATGTTGTAAAACAACCTTCTGGAGTTTTTCGAGTTTTTCTGGTGAAAG
GCGGCAGAATTTTTCGAACGCAAGCTTGTGTTCTCTTGTTTCTCTGTGTCTTTGCAACCTGCTGTGGCACCCAACATCGA
CCTTGCAGCATTCACAAAATCCTTCGGGAAAGGGATTTTTATTTTTGAAGTTTCTTTCGTGCCTTGCCGAGCGCATGTGA
ACATGATAGTTGCATTTTATGGCTGTCTCGTATCGACAAGCATCGCAAGAGAATTTTTCTGGCTCTGTTTCGCCCTTTTC
TGCTTTTTGGTGTTTCTTTGTTCCCAGGTGCTGCCGATAGTTGGAAAGAGTCGTAAAACTCTTATCGCACAACTTGCATT

CGTACTGCTCCATTACGTTTGGGCGCTGTATTTTTAGTAAAAAATTTATGGGAATTTGAGAATATTTTTACATCTTTGCC
TGTTGCAAACGAGTGAGAACACCATTATAAAAATTTTTCGGTCCTTGGACAAGTCCACAGATTCAAATTTTACGAAATTT
GAACAAAATATTTGCGCCTCCTTGCATGTTTAGAAGAAAGTGTGAGTGTAATTTTTATACGTCAGACTCTCTTGAAAAAG
ACGGGTGATGGAAAAATTATCTGTCAGACTCTCTTGAAAAAGACGGGTGATGGAAAAATTATCTGTCAGACTCTTCCCGT
CGTTCTTTCAAACATTTTCCGAAAAACATGCTTTTCTTCTCTCCAAAATATTTTGTTGAAAAAATATTTATGAGAAGACA
AGCCGAAACAAACCAAAGGATAATGTTTTGGAATTCTCCGTGTTCCTCTCCCAGAAGAAACGCCAGATGAGAAAGCGGAA
CTCTACGCCGATGGAGCTTGTGGCTGAGAAAAGAGATGGCAGAAAGAGCCATTGAAATGAGGAAAAGGTGTACGGATGCC
AAAGAGGTTTCTTCCAATGACTGAAATATTTTATGTCTCATAAAATATGGAACCTCTGTGTTTGTTCAAAGTAAAAAAGA
GATATTCTTGTTAGACTTTTCAACTAGAAAGATGTCTATCCGTTCGAAACTTTTAAAGGTGAAGTCTGAAGGAAAGTGGT
GCATTTTCAAATGCTACGACCTCGACAGAGAGGAAGATTTTCGAGAGAGTGGAAAAATATGGTATCTCGGTGACGCTCTG
AGGGAGAGCGGAGTCGATGTCAGCAGATGGGACTGCGGAGGCTTTGGAAACGTCATCGACCTGATGGTGGAACTCCAAAC
AGAGGTCATCTGGGAGAGAGCGGACGGATGGAATATGTTCCCTGTGCGCATCGGAGAAGGAAAAGAGAGACTCAAGAAAA
AACTTTTGAAGCTCTAAAATATTATTTGCAAATAATATTTTTTATGGTGGAGTTTGCCTTTCAAGAGAAATTTCTTCCCA
GAGATTCTTACTGATGGGAGTTTCGTAGTCCTTGATGTGCTCAAGCAAAAGTTTCTTCTTTGGCAAGGGTTGCTTCCGTC
GACTCCACAAAGTATGAAACTGGATGGAGCAAAATGAGCGGTCTGCAAATGCAGAATCTTTCGGCGAGTGCAAATATTTT
TATTGCTGGCTTTCGCGTTTCACTCGGAACGTTCCAAACTTTTTGTATTTCCACTTTTTGTCAAAATCTGTTTCTTTGGG
GCCAAAAGCATCGCGCATCTCTTGGGTGAGTTTGTACTCCTTGACTTCACAATACTCGACATTATAAAGTCTTTGATGCT
TGTTGCAGGCTTTGTCGCAAATGTTGTCCACTTGCTCTTCTGTAAGTTGCATTTTGCACTCATCGTCCCGACAAAGTGTG
AGTTTCGTACCGGAGCGATAGCGCGGAGCATCATCGGATACTCTCGACGGATAAGGATGGCGCTCACGTCCTTCCACAGC
GAGCCCAAATTTCGACGCCTCTTCGTAAACCCTGTTCTTGGTTCTCCACGATACAAACGTCGCATGACAACCACCAACAT
CGACCGATGTTCCCATACAGTAACGCTCGTCATATTCCGTTTCATAGTCCGCATAGTCCAGATCTTCGCAATACCACAGA
GAGAGCGTAAAATCGTGCCCCTCCAAACGAGGTGAACGCGGGTGGTACTTGTAGCAGTAACGAGTGGTCATTTCATTTAT
TTGGTGTGTTCTTCTTTTGGTCCGCGATTTGCATACAAAATATTTTATGTCCAATAAAATATGCCGCTGTCCCATCAAAT
AATATAGAGCGTTTTTCTGGATCGAGAAACGCCTGTGTAAGCCGCACGATACGCGTCATTTTTTGCCCAATTACTGAATG
TATCCCCAGCATCAAAAAAGACGTTGTCGTATGTGGACCCTTGAGACTTGTGAATTGTGCAACTGTAGCTGTAGCTGAGG
GGTGGCAGAAAAGAGTCTGCAATTCTGTAAAAATCTCTCCATTCTTTTTTCTTCCTGTCTTCCTCTTCCTCATCTTTTCC
GACGACCACCTGGTCCACGTTGAACCCCAACTGAGAAGCTTTGTAAACTCTCTTCATTTCGCGGCATCTGTCTTCGTACT
TCTTCCTGTCTTTCTTTTGGACGACAAAGACAGTGTCCAGTTTTCCAGAAGCTTTTGGGCTCTTGATGAGAAGTTTGTAG
GCATAAATTCCATTATCAAACCATGGATGGTCGAAACAGCCAGATTCCACAGAGAGAACTGTTGTCCTTTCACAAGTCGT
GTATTTCACTCCTTTTTTGTTCTTCTTCCCTTCGTGACCACCAGAGATAAAGTAGCCGTGGAACATCATCTTTTCTCCTT
CGAGGTAGGGGTTTTTTCTACTCCTTTCTCCGAAAAGTTCGAGACGGATGGCGCAGTTGTATTCAAAAACTACCTTGTTC
CTCCACGCGAGGATGACAGTGGACTCTTCCTCGTTCGAATCTTTTTGGACAAACTCAGAGATGAGGTACTCTGTGTTTGG
GACGATAAAAACAGAAGAATCCGGGTCGTTCGAATCTATTCCCCAAGATTCTGGATACTTTATCTCGTGTGTTTCAACGG
CATCTCGGAAGAGTTTATGGGCGAACGAGATGCTGTTGTCGCTTGCTCTCATGTTCTTTGTGAGACTCAGCCTATTGTTG
GGGGAAACCATGTCAAACACTGGAGACGAGGAACTTCCGACAGGAGGAAGCTGGTGTTTGTCCCCAAGGAAGAGAATTTT
TGCGCAGTGATGTCGAGACCAGTCTCCGATGTAATTTAGAAGCTTATCGTCAATCATGCTGCATTCATCCACAACTAACA
GTCGAACGTATCTCGTCTGTTCGATGGGGGACGTACGCTCTACCTTGCCGTTCCCTTCTTTGTCATACACGAACTGGAGT
CCAAGAAAACGGTGGATTGTGACCACGTCAAAGCCATCCATCCCCTTTCTTAGAACTTCCGCTGCTTTGTGTGTGGGACA
AGCGAGCGCAACTTGCTCTTTGTTTTCGAAATAGTTGAGACAGAAGCGTCTCACAGTCGTAGTCTTTCCGACTCCTCCAG
CACCAAACAAGAGAACACAGTTCTTCCGAAAATTCGAAGACGGCACACGAGCCATGAATTTCTCAAGACATCTCGTCACT
CTCTTTTGGTCGTCGTTCAATTCAAACTCTTCCTGCATCTTTCATAAAAAAATATTGTCCAAATTTTTTTCTTGGTTTGT
TTCTTCAGGTAATGTCTCCCTTGCAGATTCTTCTCGCCGCTTTGGTTCTTCTGTTTGTCATCTGGCTGCTTCAGGGCATC
TTCTCCGGTGGCATCCTTACTATCCTTGTCATCATCTCTGTCGCCATCTTTGTTTGGTGGTTGTTTATGGGACGTGCGTA
CTAAATTTCTTTGTGAAACAAAAGAAATTCTCTTTAGCCGAATGTGGACTCTTCGGAATATAGCAAGCATAGAATGTGCT
CATCTTCCGAGTGTTCTTTCGCGAGCTCTCCCATGTTCTTCGTCATAAGAAGAGGACTTTTTTTGAGAGAGCTCACAACG
TAGAGCGTTTTGCCTTCTGGACAATATTTTCGAACCTCTGCCATCGTATGAGCAAAGGACGTTGTCTTGTGGATGATGAA
CTTTTCTTTGGAGAGATAGGACTCTCCGCGAAGGGGGAAAAAGATGAGGGCTATTCTGTCTGGAAACTTCCTGGAAAGAA
AACTCCGAATTTTATTTGCATCGTTGGTCTGCAACATCTCTTACCCTTTGCTTATACTTTATCGCAAACCGTGTCGATAG
TTTGGACACTGATTTTCGTGTGAAGCTCCTTTGGCCCATTCCAGAAAAATCCCCTCTCTTGAACGTGAAGAGTCCATTCG
TCATCCCCTTTGGATGGAAAAGTGGCGTAATCCTCGATGTTGACTTCATAAAAAGCGTCCTTCATCTGCGCCTTGGCAAA
GTCCACAGCTTCCTCTTTGCTCCGAGACCAAAAAGTGCACTTGTCATTCACAACAACGACATGAATGGTTGAAAACAGGT
GTTCGTTATCCATCTTTTTCTTTATATTTTCGGAACGACGAAAGGGAGTTTATTTTATCCAAGAATAAAATAATGGCTAG
CGACAAAGAATTTGCTTCTCTGTCGTGCAACGGAAACTGTCGAGAGAGGCCTGTGCTTCTCTCTGATGGGACTGTATGGC
GTTCCTGAGAACGAGCATTTTCAGATATCTCTTCTTTGTCTTTCTTTTTGACTCGCATTTTTGGAGCTTTTCCCTAAGAA
CAGAGACTCGTTTCTCTTCTGTCTCTCTGATCACTGCTTGGCATTCCTTCAAAAAGAGAAGAGAGCACTCGTCGAGATGA
CCGATCTTTGCGGTTTTGAAATTTTCTGAGGAAGAAAAGCCCATCTCGTCGGCGTTTTCGCATCGAAAAGAAACGACACA
GTGCCTGCTCATCTTGCTTTCGCGCAAAACGTACAGTTCCCAACAGTCAAAGCACCCATACGCAACGAGCTGAACAAGTT
CCATAGTACGAAAGAATGCTCCAAAATATTCTTTCTCTCTTTTGATTGGAAATTTTATCCAGAACAAATTTTTGTTTCTT
TCATCCTCAAATTTCTCTCGCTGGGAAAGAACGTTCTTTTTTGTGAGATATACGAGCCACAATTCGAACTCTTCTCCTTT
CGCAAAACAGAATGTCCGACCCTTGCACAATCGGAAGAGAATCCGAGTACAGAACGAGACAACAAATATATTTTTACTGC
TTCTTTTCAAGAACAAAAGTTGATGGACTCGCTGCGTACTTTCGAAGTTGTTCCACATCTTCCTTTTTGACTGTGCTGCG
AACGGACTTCTTTGCCTCTTCGAAATGTTTGGAGTTTATGTTCCAGTTTGTTGATGAAATTTCCAGAGCTCTCCTTGTGG
ATGCGTTGTACTTTGAATATTGCACTTTCGAATCCGAGAGACATCTCTTCTGGCCTGTTGAAGCAGAAATTCCAAGGCCT
TCCAAAACCTTGTCGTCATCCCCCAAAGTTCCTGGAGTTTCAAACCATTGAGAGAGCTTCTCTTGATCCTTTTTCTTCAG
AGAGCTCCAAAAAGTTTCAACGTCTGCGCATGCTAAATTTCGTTCCATAAGTTTCGTTAAAACTATCGGAGTCGTCTCCA
CCAGAGAGAAAGCAGATTCTTTCTTCTTCCCAAACACAGACGCAGAAGAGGTGCCTGTCACTCCAACAGCCTCTGGTTCA
CTTCCCGCCAACCATTGGAAAACTTGTCCCATGTCGAGGCCAAAAACAGAATAGGGAATGTTGATGGAACGACGAAGCAA
AAATTCAAAGAGAATGGCCGGGTCCGCAACAGGATATTCGAATGCAGTTCCTTGAGCGCTTCCCCACGTTGTCAAAAGGC
TGCTCCTTCCGCTGGAGAGTAGCTTGACAAGGTTCGAATATTCCTCTCTTTTCTCTTGGGACGACTTTCTAGAATCGTAC
TCTCCTCCTGTCAACTTTGCCCAAGCAGCCTTTGAGTAGTCCAAGACTCTCCTCGTCGTTCCAGGTGTCAAATTTACTTC
TGTGGCAACATAAAACTCTTCTACGCGCTTCATTCCAGGAACGACCAAAAGAGTCAACATCTCCATCACAACTGGATCGT

CCACAGTTCTGACGAGCTCTTGCCAAGGGGTGTCTTGCAAAATGGTCATGTCGTACCCCTTTGACAAAAGGTGCTGTTCA
ATGAATTTGTAGAGAATTCTTTGTTGTTTCCGAAGAGAGGGTTTTGTTTGCCTTCCGGCGTACAGCTCTGGAGGAATTTC
AGGTAAAACGCTCTGGTCCTGTTGATGCACCTCTGAAATGAAAGAGGTGATTATCAACATCTTTTGTGGGAACGTCGCAC
CAGAGAAAAGCTCAAAGAAAATAGACGAGATGTTTGGGTCGTTCAAGTTCAGGATGGGGTAGACGGATTTGTTCAAGAAT
TCTTTTTCTGCAAATTTTATGGATAAAACTTGTGGTGTGTTCAAGAACCTGAAATCTGTCCTGTTCTTTTGGAACGACTT
CAAAAGGTCGTCCGGACCCAGAGAGAGGGTGCTCATAAAAGTCCCGTATTTTTGCCCCGTTTTCGTGGGGAAAGTGTACC
TCATAAAAACGTTGTTTGCAACGCCCTGTGTCGCTGCCTGTTGCACAACGCTTTTGAACAGTCTCGCAACGTCGCTCCCA
GAGTAGAGTTCCTTCTCGCAGATTTCGGCAAGAGCACGAAGTCTCGGTTCGTCAAGATTTCGAATAAACGTGTTGTATGG
CGGAAGCTTCCAGAACTCGGCTCTTTTCTCGGGCCTCTTGCAAGGAGCGCCGACGACCTTGGACGTTCCCGTCTTTCCCA
GAGACTCGCAAAATCTTGCGAGGGAGCGAGTGTCTGTCACCTTTTTTAGTCCATCGAAATGTGTGGTCAAAAGAGCCATC
ATTTGGTCATAGATGTCTTCCTTCTTTGGAAGGTCCACGAGAATGGAAGTGGTGAAACGGCGAAGAATGGCAGGGTCCAA
ATTCCATGGGTAGTTTGTGGCTGCAATCATCGCGACGTTTGGATATGAGACAATTCCGTCCATCACTTGCAAAAGAGAGT
TCACGGTCGTCGCGGCAAACTTGTCGTCGGAACGGTTCCCTGCAATGGAGTCGAACTCGTCCAGAAAGATGACAGAAAGA
ACGTCCACGGTTTCTTTGCTCGCGTCAGAGTTGAGCTGTACTCCGAGGTCGCAAGCTGCTTTTGAAGCGCAGCTGAACAT
GGAAGTTATCATCTTCTCTGATTCTCCAAAATATTTTCCTTTGAGTTGAGAAGCAGAGGGGGCGTAGAAGAGGATTCGAA
TATTTCCAGAACTTGAGAGTTCGTTAATGCTGGCCCTGACTATGTTGGTCTTACCTGTGCCTGGAGGGCCGTAGAGGAGG
ATTCCTTTCGTTGGCTCTCCGAACAACTTTGGATATCTTAGCGTGTTGATGAGACCGTCCTGAAGTTCCTTCTTTGGCGT
TTCGAGACCGATGACTGTGTCGAACCAATCTCTGCACTCTTTCCTTCCTTGTTCAAACACTATGGGATTGAAATTTTTGC
AAAGAGAGTCAGTGTCGTCTTCAGACGAATAGCGGTTTCGGACGTCGTCCTTGGCGTTTGCTATTCTCCTCTTGAGCTTC
TCTGTCTCTGACGAAAGATTGATGGTTCTTCCATCCACAGTTATCGCATCGACTCCAGAACTCGACAGTGCCTCTAAAAA
AGAACCGAGAGCGACGAGTTGCGAAAGCAGACCGACAGCATCTCCTGCTGACTCTGCTGATGAAATTCTTCCGTTAAGCC
TCGACCAAGTGCTCGAAATGTCCCGAAGGTCCATAGTTACTTCTTGTTTTTTATCCGCAAATATAAAAAACCTCACTCAA
CAAAAGTGTGAACTCTCACCCTTCCAGTCCTCCCCAAACGCTGGGCTCTTCCTATCATCTGTCTTCGAATGTGCTCTGCG
ACCGGATGCATGAACACAACGTCCGTGGTTATCTGAAGGTCGAGACCACTTGCGTTGTACGTTGCGTTCACCAAAAGAAC
TTGGATTTCTCCAGCCATGAAAGCGGCAACGATCCGTTCCTTGACAGATTTTTGCCCCTTGATCATCTTGTATTTTATCC
TTGCTCCGTTGAGGAGGCGTTCGAGACCTTTGAACGTGTCCTCATGGGAGCTAAAGATGATGATACTCAACCCATTCTTT
TCTGCGTTTTTGACAAGCTCTTCGACGTGCTCCATTTTGGACATCACCCTATCATCATGTTCCTCGAGTTTTTCCTTGCC
CTTGCCTACGTTTTTCTTTTCCTCTCCTTCCTTGACAAGAGGTATGAGAGAAGCTATTCCAATCTCAACACGGCACATTG
GGCAAGAGTCGCTCGCCTTTCCCTCTCTCGACAACCAAGCGCAAATGCAAGAACCGCAGAAAATGTGTTGACAACACGGA
GAGAGAACAGGTTCTGCAAGTTCGGAAGCACAGATCGGACAGTCCGTTTTGAGAGCTTCCTGGAGGCGAGCTTCCAATTC
TGCGATCTTTTCTTGCATGTCTTCCTCAACTCCCTCCCATTTTTCAAGAAGTGCTTTTTTGTTCTCTTTGTCGCTCGTTC
TGTACTTCGTGTAGGCTGCGATCTTTCCTCTTGCCTTCTCGAGACGTCTCTCGTATTTTTGTCTCGCAGCTTCGAAAACG
TTCTTTTCTTCTGCATCCCCTCCCAGAGAAGATATCGCTCCAGAGATGTTGCCTGCGCTCATCATCTCCAAAACTTCAAA
ACTCACAAACGCAGACAACGCTCCTCCGACAGCGTTTCCGAAACACAGGTGTTTCACCTCGAGAGGAGGAGGGAGACTCT
GAGATGCGAGAATGTACTCTCTCGGATTCGCGACTACAAGAGCGTCGATGACGGTCGGACCCAACGGGAGGAAGATTTGT
TTCAAAAAACTTTTTTTGCTTCTTGTCACAAAGTTGTAGTGGTCGTAAAACATGTGATATGTGGCTGTGACAAACCACGA
AAAGCAGGCGCTCCAAGCTTCCATGCTCGGAATTTTGACAGACGCCGGTTCGTCAAACACAAACCTCTTCCACACAAGGC
CAGAACAGATTCTGGCGAGAAAGTTGTAGAGAGTGTGTCCGCACAAGAAGACAATCTCTTTTTTCGTGTCCCTGTTTTTC
TCCAGAGTTTCTACAAAACTCTGGACGTCCTTTTTTGTGACGAGTTTGACGAGACGAATTCTGCCTTTTGGCATTTTGAG
TGTTTTCTCCCATTGCAACATGATGGACGAACTACAAACGACGAGAGTTGCGTCGATGAACCGATGTCTGATGTCTTCGT
CCATTCTCTTGTCCTTGACAAAGACAAAAGGGCCGAGATTGTAAGAGTTGTACGTGTGATAAGAGCGTTGGATGTCTCTG
TCTGTAGGTTTCATCTTTCCAAGAGCGATGAGAGTGCAAACGCTCAAAGTTTTTCCGTACCCCGGTTCATCGCCAAGAAT
GCCAAAGCGTGTTTTGAGTTCGTGTGTCCCCGCAAGCTCTGGTCCGGACACTTTTTTTGTTCTCTCCAGTTCCATCATAC
GATAGACGGTTGTCTTTTGGTGTTCGTACAGCGGAATGTCAAGAAATCTGGGCTGCTCAATAGTCGGATAGTCCTCCATG
TTTTGGTTCAAGAAACAAAGTTGTTTGTTTCTTTTGGATTTGGTTTTTGGGAACAAAAAATTCTTGAGTTTTACATTTGT
AATGTCATCGCGCTGCGCGAATAAACTCCAAAGCAAGGGGCTCACCGGAGCTCCAGAGGAACAAATAAGAAAATATTTTC
AGGCAGAGACGAACGTTTTCGAATACGCCAGAGTCTTGGCGTGTCTTTCTGGGAGAATTCAAGAAGAAACTTTTTCCGAA
TTCGAGAAGCTCTTGTCACGAAAGAGAGAAAGTTATTGCTCTTTGTTTGTCGCTGAGAAGTTGCGAGTCTCGGACACAAA
GAACAAAGTCGCTGTTCGTTCTGCGTTCGCAGAGTGGCACAGAGATAAAAAAGACCAAAGAGACACCGTGAAGCTGAAAC
AGTGCCTCGTGGACATCGGCATTTTCAAGACGCCGTACAAGTTGGAACAGTTTTTGAGACAGACGGGTTCAGACACTTGC
TCTCGGTATCTGGAAGAGACAGGAATTTTGGAAGCGGACATTCCTCCCTCAAAAGACTATGTTCTTCCGAGACTTTTCCT
GTGGCACACAAGGTTTTCAAACGACGAACGAGCAGTTTCCAGAGTCGCCACTTGTCTCGTTGTTCTGGGTATTTTCAAGG
ATAAAAAAGAAGTCCAAGAAAAACTCAAGTCGTTCTCGGAAGTTTATAGCACCGTAAGAAGCTGTTCGTCGTTCCTTGGA
ACTCTGGGTTTGTGGCCCGCTCCTGGGAGGGTTTATCTCGAAGAGAATGAGGCTTTGACGACCCGTTCAAAAGTGCTCGA
ATTCCTTCGTGCATCAAAGGATGAAATTCTGTCCAAAAAGGTCAGGGAGTGCGCTACGCTGACAGGAATTTTGGGAGAGG
AAGAGGACATTTCCATCTCTGGGAAGGAAGTTCTCGTTCTCAGCCCAAAGGTCGTTGATGCGATTAGAGAGCCCGCATCC
GACAAAAAGAGACAATGCCGAGATTTCTTCTCTGATATTGGCATTTTTCCGAAAGAGGCAGAGTTCATCCACCCATCCCA
GATTAGTCTCTATGGGCCGTCCCTGCAATACGCGTACAAAGAGGCAAACATCCTCCAAAAGTTGAGAATTCTCGAGTGTT
CTGAAGCTCTTGGTATTTTCTCTCGAGAGAAAATTTCAAAGAAGGAAGCTCAGCTCGAAAAATGCCTCGTTCGTCTCAGG
CAAAAGGGGTTTTCTTCTGTTTCGGCAGACGACATGATTTCCCCCGCAGAGCTTCGCGAGCTCGAGATGAAGGTGAGAAA
GGGAGTTTGGGACCAAGAGACTGCGGAATGCGTGGGGAAGGTTCTCGGCTCTGGAGAAAAACCAAAGGTCAAAGAAGGCG
CTCTCGTGGTAAGAAGGGAGAAGGAGAAATTTGAGAAAATTCTGGAGGAGCTCGACTCAAACATCGCGCGGTCAGACGTC
ATGGCTTATTGTCTGATGGTCCTCAAGGTCTTGGATATTGCTCCGAATTCTCTTCTCGTCCCGGAAGTTCTCGAATTTGT
TGATGACCTCTCATCGCGCGTCGACCTTTGGTTCGAAGAAACGCAGAGCCCAGTCCTGAAGCATGAAGTTCTTGTGGCTG
CTGGAAGCCTTGGAATTTTGAACAAACCAAAGTACGAAAGGCTCGCCGACCAACTTGTCCAAGATTCTCTTGACTTTTTA
GAAAGAAAGGGCATCAGCAAGAACAATCTCGACCAAGTTTTTTCAGGAGCAGGGACAAAGAGCTTTGGAATTCTCCGCAT
CGCCTCGATGGCCGGAATTTTGTCAGACGCAGAGATTCAAAGGTATCTTGTGGCTCTGAGACCCCAAAGACAAGTTTGTA
TTTCCGGTCTCAAAAAAGCTGCGTTCCTGCCGTTCGTTCCTGACAGCGACGTGGAACTTTTCATGCCTCTTGCTGCCCAC
AAGTTTATGGTTTGGGCAGAGAAGGAATTCGAAGCATCTGCTTTGGACGGAACAGAGTTTTTGAACATGGCATCTTGCAT
CACTCTCGTGAATTCAGAGGAACAAGGCTCCCTTGTGTTTGAAGTTCCTTCTTGGAGCACCATCAAGAAAGTTCAAAAGG
AGACGGTCGACAACATACCTTCTGGTCTTTTGACCCTCGAAAAAGAAGGCATCATTTCGCAGGACGTTGACACCACAGAC

ATCTCGAAAGTGGCAGAGTCCATCAACCAAAGGTTTGAACTAGAATTTGAGAAAAAGTCGCCAGAAAAGAAGCTCATGAT
CCTCAGCGCTGTCGCTTCCACCGGCATCGCAGAGTCTGCAAGTCTCCAAGACCTTCGAAAGGAAACGCTGCAAGAGCAAG
AACAAGAGAAACCTTTTATGGTGACGGCGTCTCCCCCCAAGAAACTTCCTTCTGGCGTTTCTTGCACGAAAGGAGCGAAC
GGAAGGTATTACTGGTTCCTGAACGGAAAGAGAACGAGAGCAAAGAAAGAGTGGGATAGAGAGCTCTGTCAAAAGAAAGC
TTTCATGAAGACCTCAAAGAGGCCAAAGAGATTGCCAAAAGATGTGATGTGCATCGAAGGAAAGGACGGAAAATTCTATT
GGCTCAAGAAGGGAAGACTCACATTCTCTGGAACGGATAGACCTTGTCCATAAAAATATTTTCTTACAAAATATTTACTC
AGACTCAACAACAACTTTATCCCTCATGAAGGACTCTATCAAAAACGCCCATCGCCACAGAGAGACGAGACTCTCCTCCC
GCTAAACAGCAGTTTTCAAAACTCTGCCAAACAAAGGCTTTGAAAATTTCTGCATAGACGCTTCCTTGCTCTTCCAAAAT
CTTTGGAAAGAGTGATGTATATTTTTATCCATAAAAATATATCGTATCAAAAAGACCAGTCGATCGGTTCCTTTCCAGCT
CCTTTGAGGTATTCGTTTGCTTGGACAAAGTGGTTGTTGTCCTTGAATCCCTCAACAGATAAAGGACTCGGATGAGCGCA
TTCGAGAACGAGATTTTTCTTTCCGTTGATTATACCTCGGAACTTTCTGGCATGACCTCCCCACAAAATATAGACAATCC
CCTTGCAGTTCTCGTCGAGATGGCGAACTGCATCTCCAGAAAAGTCTCCCCATACTTTGACGTGTGATTCCTTTTTCCCC
TCGAGTGTCGTCAGTGCTGTGTTGAGAAGGAGAACTCCCTGTTTTGCCCAACATGTGAGGTCTCCGGAACCAGAGAGACT
GAAACCTTGCTTTTTGACTTCCTTTGCGATGTTGGCGAGAGAAGCTGGAAGTCTGCCAGAACCGCTAAAAGAGAAGGCGA
GACCCATTGCATTTCCTGGGGTGGGGTATGGGTCCTGACCAACGATGACAACTCTCACAGAATCTCTCGGAGTTTCAACG
AACGCCTTCCAGACGCTTCCAATCTTTGGGACGATTTTGTTGTCCTTGTCTTCCCAAAGTTTGTCACTTATCTCTTTGAT
GTTCTCTTCTTGTTCGTCGAAAAACTCCTTCCACCCCAAAGGGAAGAACTTTGGATGTTTTGTCAGCTTCGAGAGGCCCA
CAGCGGTCCAATTCACCACGGTCGACGGAGCTTTTGCGCCAGAAGCGACTTTCTGGACAGGTTTTTTCTTTTGCTGTTCC
TCTTCTGGATGCACTGAAATTTTGACAAAAGGGTTATTGCACCTTATTCTGTCCACAATCTCGTCAAATTCTTCCGGAAG
CTGTTTCTCAGAAAAGGCGAGATGTTCGAGGTTTTTGATGGTTCCGATACGGCACAAAGTTTCCTTTCTTCCTTCCAGGG
AAGAGAAAATGGACAAAACCTTTCGCGGCTCCCTGGTGAGAACAAGAGCGTTCCCTTCCCTCCGTTCAGGAAAAACAAGA
CTTGGATGAACTTCCAACAGCTTTTGGTCGAACGAACTCTTTCCCTCACTGACGATGCGAACGACGAGTTGTGCGTTTGT
TTCCCATATCGGAACCTGACTTTTTGACGGTCCGCTCTTGTCTCTTGCAGAAACAACCCTTTCGACGATGTCCTCCCTCG
AGCAACGAAGGACTGAAAGACAAAAGTCAAGAAGTTCAGTGTCTGTGATGTTTTCCATATTTTTCTTTTTTGTGTATCAA
AAGAAAAAAGTTGGTTGATTATGAGCAGCTATAGCTTCCAGACAAAACGTACCCAGAGGCACAGTTTTCGCCAGAGGGGC
AAACTCTCTTTCCGCTCCAAACTCCAGAAGTCGGAATGTTTTGCAACGTGATGCTGTTTTTCCCGTCCGTTTGCTGTTTC
AACTCTGGTGTCACATCAAGCTCTTCACAAGAAGAACAGCCGGTTCCATCACCAAACGGAGAATAAGTTGCCCTTTCGAG
CGTCATCGTCTTTCCGACTGGACAGCTCAATGTGACGTCCTTCCCTCCGATCTGAGCGAAACCGCTATACAGCGGCCAGT
CACGAATTTTGCTGTATTCTCTTTCGGCGTACACGAAAAGATAGACGGAGACTCCAAAGACAAGAAGCAAAATAACTAAA
ATTCCGATACCGACCCAAGTAGATGCTTGCATATTACTATATTTTTTGAAGCCAAAAATATATCAACGGCAATCGGCAGC
TTTTGTCTTTGCTAGAACGTACTTTCGCAGTAACACACGCATTTTGGGGCGCACTTTCGCCTTTGTGGAGGATTTTGAGT
TGGGAAAACTAAACTCCTCCAGACGGTAACTCGCTGTTTCTGGGTCAAACACAAGCCCTTCAATCTCTTTTATTTTTCCT
TCGTCCATCAAGATGGAATCTGCCCGAATACATCCGAGGTCGATACAAGTCATCAGAAACCAATAGAGGCTCTGTCTCTC
TTCGTCTGAAAGAGATTTCTGCTTTTGTTCCTTTTCCGTGAACTGCAAAACAAACATTTCTCTTTTGGCCTTGTTTTTGA
CCTTTGACCAGGAAACGCTCTGTTGTTCGCCCGTGAAAGACTCGAGCTTTTTGTCGAATTTTTCCGTGTCAGATATGATG
CCCAATTCTGAGCGCAACGCGCCAATTATCATCATGCTCGTTTCCGCTCTCTCGTCTTGGTCTCCATCTTCGGAAAAACA
GAAAGTTTCAAATTCTTCGCTCCCAATCACGACGGAGTCGTCCTTCAGAGTTATTCCTGGATGAATGTTGCCGTTGGTAA
AGTTGTCCAAAATATCACACCAAAACGGATCGAGCGTGTACTCTTTGACGAGAGCGAAAATGGGATAGCTTCTCTGAGCT
TCGACCTTTTTTCTTCTTGACATATTGTCTTACATCACACAACAACTTTGTCTCATTTCTGCAATTTTTATCTGGCAAAC
AAAAGTAATGGGTCTTGCTATCTGGATTTTTGTAGTCATCGTCTTTCTGGCGGTTGTGGGAATGTTTGTTGGGTTTTGGA
TGTTCGACTCAAAGTTTGCTCAAGCAGCAGAGAACAACTTGCTGAGACCTTTCGCCGCACATCAAGGAGAACAAGTTTTG
AAGGCTGACGGCAAGCCGCAGATGAGCTGTCCGGCAGGGAAGAAGATGACTGTTATCGGCGCGGTCTATGAAGTTTACGA
CCCTTCCCTCGAATGCACAAGCAAACCCCTCACAGAGTCTGGAACGCCAAACTCTGTTTGTGGACCGATGTCAGCCACAA
GTTCAAACGACCAACTCTCTGGTGTTGGCGGAAGTGGACAATGTAGAATCCGAGATGTGACCGGACAAGTGGGAATGTTG
TGCAACGGAAAAGAGACTTGCGACTTTGTGGTCGATGCAAGCTCTTTGGGTTCTTACCCTTGCTCCGATGCCGCTCCAGA
CAGCGCAGAATATAACGACCTCCCCAAAGACTCTGCCGGTAAACAAGGATTTTGGGTACACGGCATCTATCGGTGCGAAT
AAAAATATTTTGAATAAAATATTTAGAACCAGATGTTGCTCTCGAATTTGGAAAATTCTGTAAAACTCTCGACAGACGGA
GAGGTATTTCCTCTCACCAGAACCAAAACATCCTTTTGTCCTTTGTATTTATCACAACACGGACAGAGAACGTTGAAAGC
TCCACCGTGGTCAAACCCGCCATACACTTCGTCTCTGATGCTGTCGAGAGTTTCTCCTTGCAAAGTCTCGTCTTCATCAG
TCTCTGTGAGAAGTTCCGGCATTTCATCATCTGCTTCTTCTTCAGCAAGGACGCTTTCGTCGACATAGAGAGGATTTCCT
TCGAAACGACGTACGAAAAACATGGTGTAAAAGAAAGGACAAATTCGCCTCGATGTCTTCCGATTCTTTTTTGTGGATGT
GTCTTGGAGAGGGGGAATGGTGGTGGACAAAACTTCAAACGACAAAACGCCAGTTTGAAAAAGTCAAAGAAGAGCTCGAA
TTCTGCCACAATTGGGTTTTTGACATTCTCTCAAAGGGAAATGCGCCAGAGAATTGCTACGCTTTCGCCGAGTGCAAAGA
AGAACTCTCGAAAGAAAGAAAAAGCAAAGGAGCGCGCATGTTTTACCACAACGAGGTTCCTTATCGCTTCAAAATAACCA
GAAAGGAGCCAAAAGAATTCAGGGACAACTCTCTCACAAAACAAGAAGCTTTTGAAGTCTTTACTCGCCCTTACGACACC
TATGATTTTTCTCTCTCGGTCTTTTGGAGATGCCTCCTGTCCTTGCGTCACTCCTCTCATAAAATATTTTGTGAACAAAA
TATTTATCTTTTTCTCCTTTGGTCTCTCAAAAACCTTTGCTTTCTCTGTTGTTCGAATTTTTGCTTTCTCGCCAGTTCGC
AAAGGGTCTCTTCTTTACAAACTTCTTCCTTTTTCTGGGAATGTGCGCCATGAGAGTTTTCGCGTTCTATCTCGTCGAGA
GGAATGACGAAACGTCTCGCTGTTTCCGCATATTCGTGGGGAGAGGCAGAACGCTCATCTGCCTTCGGAATTTGTTGATG
GAGGGCGCTTTCGTCAAAATATGGGCAGACGAGCCTTGCCAAAGCGCTCTCCATCACCACTTCAATGTAAGCTCTTATTT
TTGTTTCTGAAGCATTGGCGCCATTTCTCGAGGAAAGAAAAGACTCGAAAGGAACAACCTTCAATCTTCTGAGTATCGCG
TCGTCTTGCATCGAAAAATCTGAGACGGGCTATTGGAGAGTACGATTTTAATGGGCAAAGAGTAACCATAAAATTTCTAA
AAAATAGCGCGTTTCGAGACGAGACAATGTTCGCTTTTTACTGAGTGACGCTCCAGCGTCACAAGTAAGAAAATTTTGAA
AGAAAGAGTTCTTTCAAACATTCTCACAAAACACAACTTTTTTTCCTCCCATAAATATTTTCTCGAATAAAATATTTTAT
CCCAAGCTCAACAAAAAATCCTCTCAACCCTCGTAATCTCTCACTGCTACCGCACGAGGAAACCTCGGAACGCCTGTCTC
TTCATTCCTTCCCTGAAATTTTACAGTCACCATCTTCCCAATGAGCCCCTTTTTCTTTTTGTACATCTCTCTCCTCCATT
CGATGGTTCCTGTCGGTCGGCACGTGAAAGTGTCTCCGCTTTCCGTTTCCAGAACAAAGATTGCAGCTCCGTCTTCCGTT
CCTCCTTCGCTTTTCGAAACGCCAACAATTTCGTATTCTGCATCTTCAAAGTCCTTGTACTTGAGAAGCAGTCGCGACCT
TTTCTTGGGCGTGTACATCAACTCTGCATCGCGAAGGATGGTTCCTTCAAAACCTCGTTCGACCCATTCCTCGTGCAGTT
GCAGCATCTCCTCGTGACTATGGACTGGTTCCGTCAAAACTGGAACGATGCATTCGGGCAGGTCCCCAGAGAGAAGAGAG
TCGAGTTCTTTTTCCCTCTCGAGCCATGTCTTTTCTGTGTCTACAATGTCGAAAATCCAGTATTCCAACCTATCTTCTCT

TTCATGGGGTTCTTTTCTAACGCTTCTCGCTGCTCCAGTGATAAGTTCGAATCTTTCATTCTCTCCCATCTCCTCGCCAT
GAAAATAAAGTTCTCCATCCAGAACCATATTTTCCCTTCCCTTCAAAAGTTCTGCAATGCCTTCGCGGATGTGCTGCAAG
TGGACAATTTGCTTTCCCTTTCGCGAAAGGAGTTCGACGTTTCCTTCGACAATTCTGGCGATGCAACGAACTCCATCGAG
CTTCGGTTGCGCGACTGACCCCTCCCTGAATTGGAACTTTTTGGCTCGAGACTTCTTCTTTGGGTTCATGATATCGTTGT
ACTTTTCCGCAAGCATCGGAAGGAAAATTTCCTTTGTCTGGGACTTTTTGGCTGTATTCTTTTCTTCTTTCTTTGAAGAA
GCCATGACGTTGTGGTTGTTTCCGCCAGCATCCTCTTTCGCTTCCAAAATTTTGGAAGCGAAAGCGACAGCTTCTTCGTC
GTCTTTATCCACAGCGTATCCCTTGTCGAGCTGTTTAATCCAAGAGCAATCCCTCTCTTTGACAGCTTGCTCTTCTGAAG
TTGTGGAGTTTGACCTTCCAACGTTCTTCGGCTTCGCCTTGGTTTCGACGGTACGAAGCTTCCCTCCAACATAGCCAAAC
TCCTTGATGATATTTTCTCCTTCGCTCCAGACCCTCCACTGCATCGTCTTTCCGGTCTTTGTTTCGCAATAGAGAACAGG
CAACATTTTTCCTTTGAGAAGTCCAAAAGAAAAAACTTTCGATAAAATATTTTATGGGGCAATGCTGAGAATTCGTGTTA
TAACAACGTAGCCGGAATACACAGTGTTGACTGCGCTGTTGCTTGTGACGATGAGGCGATAATTGCTCAAGTTGATGTCT
GCTGTGACTGCGATGACCGCGGACCCGCTGTTGCTCGTTTGGTTGGTTGTTGTACCAAAGACAGAGATCAAAGAACCTAT
CCCGTTGCTCAGAACGGAAGATTGTCCCACCGCGTAGTAGACGTTTGCTGTTTCGTAGTGGGCGAGAATGTTCCATTGAA
AAAGATACACAGCGTTAAAAGACGGCGTGAGCGGAGTTCCAAAGGTGAATATCGTTGTTGGAGATGTTGAGGCGACGATC
ACTCTCGTTATCGGGGATGTGGACGTTCTGAGTTGTGCGCTGTCCGCTATAGTGAAACCTTGACTTTGTGCAACTATAAG
ATTGGTGATGTCGTTTGTGTTTGAGGTAATGTTTGTCGTGTTTGTTGCGACTTGTCCTTGCAACGTAGTGATATTGGAGG
TGTTTGTTGCGACTTGTCCTTGCAACGTAGTGATATTGGAGGTGTTTGTGGCGATGTTTGTTGTGTTTGTTGCGACTTGT
CCTTGCAATGTGGTGATATTCGAAGTATTGGTAGACGTCTGCCCTTGCAACGTAGTGATATTCGAGGTGTTTGTTGCGAC
TTGTCCTTGCAGAGTGGTAACGTCAGTCACGAGAGTGGAAACGTCCGACTGGAGAGTCAAAATATCAGCTGTGTTTTGCG
CCACCTGTGCCGCCAAGGGTCCGACGTCTCCTCCTGCCACAGACCATATTTGTCCGTTTGAAATGTACAGATTTTGGTCT
GTCGTTAAAAAAACAAGAGACCCCCTTGAAAGGACTGGAGGAAGTACAGAGTATGACCCAACGACGGGGATGTCCTTGAA
AAACCCGCGAACACGAGACATTACAAGAATAAAAAAATATCTTTTGAGATATTTTTATGAGAGAAAAGAACCACAATCTG
TGAATTACTTGCGGAAGACTTCCATCCTTTTCACGAATTCTTCGAGCCTCTTCCTATGGGGCAGCGCCATCTCTTCGGAC
GCATCAAAGTAGAAAAAGTCGCGAAGATGAAAGAGTTTTGCATAAAAATGTCCTATGAGGCTGCCGTCTTCATCCAAAAC
TCGTTCTCCATTTTCCTTCCACTCCCTGTGTGAAGGGGTGGAAGGAACGAACATCGGAGTTCCGCGGATGGCGGCGGTAC
CGAATGCTCTTAGCACACCCTCTTCGCAAACAGCGTCGAGTTTGTCAGCATCACGAACGATGCGAAGCTCTTCAAAAACT
GGCACATCTCCTTCCTTCTTTTGAACAGACCACGACGTTCGTTGGATTATCTGGCGAATCTTGCCCGTTTTTTGTTCATC
CAAACACAGAGAGGACAGAGCTTCTTCGAGGTCGACACGAGATGCGTACTTGTGGTCTGCAATATCGTGAAGTACGCAGC
CAATTTCCACAACTTCTCTGTCGAAAGTCAGAGAAGTTTCTTCCATAATTTTTCTCGCGTGTGCGAGAACTCTCAAAACA
TGCATCCAGTCGTGGGTCACATCCTGAGTGCTCATGATGGGTTTTGCGATGGCGACGGCTCTGTCCATTTTTTCCAAAAT
ATTTTTAAGAAAATATTTTATTCGATAACTCGAGGCATATCCCAACTCTCTGTTTTGTAATATGCTCCTTTGGATTGCTT
TGGTTCTCGTCGCTGTTCTCGTTCTTTGGCTTTTTATGAGACAGAAGAGGGAGACAGAAGCGTTCACGGAAAACAAAGGA
CAATTTGTCATCCCGAGATCGGGAACTTGGCTGGTCTCTTCTGACTTGGAAACAGAAATATTTGTGGTTGGTTTATCCTT
TGGGAAAAAGAAAACTCTGATTCGAAAGCTCGAGAAAGGACAAACTATCTCTTGGGAAAGAGGGACGCACATGAAATTTT
TAAGACTTGTTTAAGGTAAGTACCACCAGAAAAGAAAAGATGAACATTGACAGCACACAATACGTAACCTCGGGAGACGA
ATCAGAGTTTGACGTTCCTGTTGGTGAGACGACATGGTCGACGAGTCAACTCTATTGCATCGTCGATGCCTGTTTCGACC
TTGGAACAAGGGCAACTGAAGGGATTACAAATCCTGTGTATCATGTGTCGTTGAGCCATGGAGACTCTGAATTTATCCGT
TTCCGCATCCCCATCACAAAGGTGTACGACGGAATTCCGTCGTACACTGCCTTTTTCAAGCCGTCTCGTGACGTTCACAC
TTCTGCGTCCGACACCATCACTGTGCGAGTCGTAAATTCTTCTGGCGTGAGAATTCAAAATCCTGGTTCTTGGGCGCTTT
CATTCTTCATGTCTCCTTCTTCAGAATAAAATATTTTTTGAGAAAATATTTTTTACATGCCCTTGTTTGGCCAGTGCGGA
ATCACAAACTCGACCATCGCGTCACTTCCGATGGGCGTTCCGAATCCGCTGTTGGCACGAAGAACAAATCTCAGTCTTGG
CTTGAGAGGTTGAGGGAGTTCAAATTCTGTCGCTTCTCCAGGAAGTTCGAAAGTTTGCACGCCAGCCGGACACCCTCCCT
CATACTGCATTTTGACGGTGTAGCTGGAGATGTTCTGTGCGATGGGTTTCCAGAAAATCTTCCAACCGAATTGGGTACCC
GCGCCGTTCCCGTCGTTCCTCAATTCGAGGTTTGTGACAGGTTGAGTGTCAAGCGTGGAACAAGCAGGAAGACCTGTCCT
TTGATGGTACATCTGAAGCGCAGCTCCGAGTTTCTGCGGGTCCATGGATGAAAGGGAGACTTCCTCGCACTTGGAGTATG
CCGGTTGTTTCGTCTCCGTTTCGGTAAATCCTTCCTTTTTCGTCTCTTTCGAATGGAGAAGGAGAAGCACAAAAAGTATC
ACAAAAATTATCAAGAGTATGAACGACGGACTCATCTTAACAAATTTGTTTTATCTTTGGAGTTGCAAAAATAAATTCTG
CTTCCCACCTTTTTTCTGTTCGCCTTTCTTTTGGAAGGCTGGCGTTCGCCAAAAACAGACGCGACGATTTCCATCTCCTC
CTTGCTTTCGAGCAAACATTCATCGTCGCAAGAGGGGTCATGTTCCTTTCCTGAAGTACCGTCCATACACGAGCACCAAT
CCAGGACGCCGTTGAGCCTTGCGGAGCGATGTTGGGCAAAGTCTTCCATATCTTTTCTTTTCAAAAGATATCTTTTGTAA
GATGACGTCCTCCTACATGGTTTCACAGAGGAGAATCCCGACAAAGCCCACAAATTTCGACGCGAGGTTTGTGTCTTCTC
CCAATCAATGCATCGACGGAGAGATAACAGTCCCTCTCCTCGGAAAGAACGGAAGTTGTTCCGGAAATATGCTGTGCGTT
CCTTGGAAGTCAAGAGAAGCTCCGATTGTTCCACAAAAGTCGCAGATTGTTCGACACACAAGCAACCTGTATTCTATACC
TGAACCTTCCAGGCTCTGTTCTGGGATGAATTCGGGTCTTGCGTCTTCGACAGACTATGCATCGACATACCTTTGGGAAA
CAGACTGTCGAAGGAGTCGAGCTCCTCCAGATGCCAACTATGCGAGTCTCAACGATTACTACAACCTTCCAAACAGACAC
GACGGGACGGGTTTCAGAGACATTTCGAGCAGCCTTTCTCCGAGGTACTATCCGAGCATCGAGAAAAAATACGAAGTCTA
CCATATCATGCCAAAGGACACGCTTAGAGTTCAAGAGAGAGATTCTTGTTCTTTTCAAGTATAATTTTCCAAATGTAAGA
AACGCATGCTCGCAGGTTACATTTGCCCAGAAAAGGAACTCGAAGAAACACAAAACTCGCAAGTGCACATCTCGCTGGTC
AGAGCTTCAAAGAAGAATTTTGTAACGCATGTTTCTGGTCTTTCAGACCAAGAAAAAGCACTAAAATTTCTAAAAAGGCA
CCTTTGCTGTAACGGAAATTTGAAGGAAGGGAATATTTTGGAATTCCAGGGAGACCAAAGGCAAAAGTTGGCGCTTTTTC
TTGGGGAGAGATCCGGGTCACAAAACATAAAAATTCACGGAATATGAGTCTCGAGGATTTTTTGAAATTTGAAGAGAGGA
AAAAGTTTTGGGTAGTTTGCCCAGAAACAGCACAGCAAGAAATACAGAAATTCGAGAGAATTTTTGGAAAGAGTGTGTGG
GGCACAATACCAAAAGAAATAAAAAGGTTGTTCTTCCTTCGGAGAATTCCTCCAAGAGAAAGAACCCTGTCGCGGTTTTT
TGGAACTCCTCTCTCGTTGGACATACAAGTGAGCGTCGAAAGGAAGAATGGGGGCGTTGTTCTCGTCTGGAGATACCAAG
ATATACTCGAAGAAAGCGGAGAAAGAATATGTACGACGACAGAGTCCCTTTTTCGGGAACTCGCAATGTTGTTGGACGAA
TTCGACGGATACTGTGATATGACAGACGCAAAAATGTGGGTCGAGAAATTCAAAAGGTCACTTGAATAAAATATTTTTTG
AGTAAAATATTTTATGTTCTCTCTGCTTTCATTCTCTGTCTTCTTCTGTACCCTCCGCTGACGTGAGAGTTTGAGAGAAA
AACCCCTCTTTCCTTTCGGAATTCGCCAGTCTCTGTGCTATACCAAACTCTTTTTATACCGCAAGAGCCCAGAAGTTTGC
TGCAACAGTTGCAAGGTTTCGCCATCGCGAGACTGCCGTCGTTCAAGATGCGAACGACATAAAACTCAAGCTTCGGATAG
GACTTCTTGTGCATATGACCCTTTTAACAGTCGACGCAAAGCGCACATTTCTGCGTGCATCCGACAGTGGTTTCCAAGGA
CGAACCCGTCCATGTCTTTGTTGTATCCAAAAGAAACGGGCCTCCCTCCCATCACGATGACACAACCAATTTTCTGACTC

ATCTCGCTCTTTTCCGCTTGCTCTATCGCGAGAGAGAAAAGCTTCCTCTTTGTTTGGGACAGTTCTTCCATTCTCTTTTA
GAATAGATTGTAAAGTAAAAGAAAGAAACCAAAAAAGTTTTTATATGTTCGGTTCTTCCAACAAAAGAAGATGAGTAAAT
TTCCGGAAGTCGTTTCAAGAATCTCTCCCGTTGCTTTTCCTGTGGAGCTCATCATCCCTTTCGCAAAGAAGAGCTCGCGA
GGAGTTTTAGTGGTGGAGTATGGCTATGTTGGGAGAGCGTTGGTCGGAAAGGAAGGAGTTATTTCCCAAGGCGCCATATG
CGCTCACTCGTGGCATGAACCTCACAACGAGAAACAGAGAACAAAGTTTTCCATCGGAGCGAGGACAGAAAGAGCCATTT
TCGACATTGACCTTCCAGAACATGGAGTTTTTCGAGAGCTTTTGTTCCCAGGCGAGACAGAACGAGGAGAAGACTATATC
TCATATTCCGAGAAGGACTGGGAGATTCGTTAAAATATTTTAGACAAAATATTTTGTTAATGAAATGAGAGACTTTGTGC
CTTTGACTCAGTACAAAAGAGGATTTGTTTTTGCAGACTTTGACTTTATCTATCGTGAAGAGCCAGAATTTATCAGAGAC
ATTTCCGCTCTCGAAAAAGAAGGCGTCTCGTTCATAAGAAGAAAAAGGTTCAAATGTGGCAGAAACTTCCACGGAAAGAA
AAAATACGCCTATGACATCTCTTGGAAATATCGGGGAGACTCAAGAATTTTTCACCTTGATGTCGACCTGAAAATGGAGG
AAAAGCCAAAGAGTGCGGAATGGAACGTCGAGAATTTTGAGCTGAGAGTGTCGCAGGGGCTGACTCGTCTATTTGTCGGA
GACTCAAGAGACCTTCCAAACACGAGAGAGGGGAGAGTTGCGAGAATCTTGGCTTTTCTTCCCGAAGCGAAAATAAGCTC
CGACAAGTAATATTTTTTCTTGCGTAAAGAAAAAATGCAGAACCCAAACTCTGTGGAAATAAAAGCCTGGAAGGAAACCA
CAGAGGAGTTCTCTATTGTGTGGGACGATGTTCTCAGAGCGATGGGAAAATGGCGAGATTCTGAAAGCGCAGAAGTGAGG
GCAAAACTTCTCGCAGAGTTTTGCCAGGACAATTTCCCCAGAGATTCAGAATATTACAGACTTTCGGAAGCTCTGCGGAG
TTACGACTTTGACTGGTTCAAGTTTTGTTCTCTGAAAGACATCACTGTTGTCTCTGAACTCGGCAAGGGAACTTTCGGAC
ACGTCTATAAGGTGCTTTCTGGGGGGCGCCTCTTTGCTCTCAAGCTGTTCGACGTTGGAATCGTTGAGAGAGGAAAGAGA
AACCCCATGGGCATCGAGGGACTTTTGGACTTTCATCTCGGCAGGTACGAAGCAAGATGCATACTCGACTTGACTCTCCA
AAAAGAAGGAAGCATCCCTTTCCCAAAGTTTTATGACTTTGGAATCACCGCCGTTGGCGGCAAGGTCAGAACCTACATTC
TGATGGAGTACCTCGAAGGTGAGACGTTGTGGGAGCTCGTCAAAAGCGGCAAACGCCAAGAAAATGTGGAGGCTCTGACC
AAAGGAATCTTCTCTGCCGTCCGCCATCTCCACGTCAAGGGATATTCTCACGCTGACATCAGTCCCGCAAACATCTTCCT
CACAACAGCGGGAGAGGTTAAACTCATCGACCTTGGCTCTGCGTGCGACGTCAATGTGGATGTCTTTGAATTTCTTGGCT
CTGTGAATCCACCAGAAAACTTTGCAACACCCTCAAAGCGCATCGGCATGGTGTCACAGTTTCGGAATGATGTGTGGTGT
GCTGCGTACTGCGTCATTTACTCTTTTGGCAAAAGAGTCGGGAAGAGAAAGTTCCCCCAACCAAAGAGGGAGTCTTTTTC
AGAGTTTCTCGATGACCTTGCGCTGACGATGGACGAAGTCAATGAGAACATCGAGGAAATTCCTCCTGTCGTTTTGAGAG
CTCTCAGTCTCAACCCCGAAGAAAGACCTCTGTTTGGTCAACTTTTGTAGATATTTTATGAGAGAGTTTATCCATATTTT
CTTTGACACCAAAGCGCTCCAGACAAACTCTGTCGTTCCTTTGATAAATATTTTGTACACAAAATATTTATTGCGCAAGA
GATTCGAAGTGTCTCTGCGCTTTCAGCGCTCCTTCTCCTCCGGGAGCGTACAAGAGCTTCGCGTTCTCGTTCTCCAACTC
TTTTATTTTGGATTGTTGTTCTTTGACTGTGGTCAAAAGGCTTTTGTACTTCTCGACAAAAAGCCCAGAGAGATTCGAGT
AACAGATTTTTTTAATGCGCTTGAACGGCTCTCCTTGGTCTGTGTACGTCTCTCTTCCGATGCTGTAGACTGTCCAGTTC
GAATAGTCAGAGACCGTCTCTGACCACTCACAGACGACAACGTTCTCCCACACAATGACAAATTTCTTTCGAAGGTATCC
ATCACCAAAGTCTCTTTCTTCCCTAACAACAAGCTCCGCTTCCGGAATGGGATAACTCTCAGAGATGAAAGAGATGGTGG
TCTTCAGCATATTTTAGCAAGTAAAAAGTTATGCGCAAAAACGTCCCGTACACTTCGATGTTATTCAAACAGTTTATTCT
ATCCATATAATTTTTATTGTAAAAATTATGGTTCATTCGCATCTCTCAGAGTAGATATCCTCCCACCTCTTCTTTTTCTT
TGCTATCCTCGAGATGCACGAAAGGGCATAACTCCTGTAAGTTTCTGACGCAAAGACAGCGTTGTCGATGTCTGGATAAC
TGTCCGTTTCTCCGTGTTTGTCTTCCTCCCAATAAATTTCTATATCGAGCACCCAACACGAGACATTTTTTCCTCTTCCG
TTCTCTGCAAAGTCAAAGGAAATGTCCTTCTTTTTGATGTGGAATCTCTTGACGAGTTCCCCTTTGACTTGTTTGCTTGT
CCTCGAAGACATTTGAGAAAAATATCTATATTTTTCTCTTCTTTTGTTATTTTTTTTGAGAAAGAAATGTTATCCCTCCA
ACACAGAGGGAATTCCGTATCTTCTCAGGACGACTTCAGAAGATTGCAGAGCTCCGTTCACCCAACCCGGAGACATGGAT
ATCGCACTTCCAACAACAGAAACGTTCTTTCTGATTCTTGTCAGGTCGCGATATCCTCCCGCCTTCCAAAAGAAAGCGCC
GTGATTATGGTAGTAAAACAGCACAGTTTCAAGGTCTATCTTTTCTTCAGACACACCAAACATTTTCGAACAACACGCTC
GAAAATTCTCTGTCAGCTCTGGGTACAACTCGCAACTCTCAAACTTTCCGTTTTTCTCTTTGGGTATTTTGTTCCTCCAA
AAAAGGCCGCTGGCGTTGTCGCAATAGAGCATCAAAACATAAGGCTCTTCCGAATAGAACCATATCTGTCTCGCAACACC
AGAAGAAACATTTCTCCCTTTTCTTTGACCATTCAAAGACAAAAGCCTCCATGTTTCTCTTGGAACGATAAAGAAAGCTT
TGAACGCCGACCAATTCTCGACGTATGGAAAGTAATCTATCCCCAAAAGACAGAGTGCGTTTGGCGGAAGCGCCAGAATC
AGATTCTTTGTTTTCACCTTTCCAATGCCCAGAATTTTACATTCAAGATTTTTACTAAATTCTGTGAGAGTCGCTCCGAG
CACCACCTTTGTGTCTGTCAACCTCTTCGCGAGCTCAAAAACGACAGAGTCGTAGCCTCCCTCAATAAAGTGTTGTTGTG
ATGTGCCAGAGAGACTCGAATTTTCTCTGATGCCTGTCGACGTACCAATCGGCGCATCGAACGCAAAGTTGTATCCAGAA
AGGTCACGGTACGCTTCGATGGTCCCACAAGAAACCCCTTCTTTTATCAGAGACTCTCCATAAATTTCCCCGTTTAGTTT
TCCGCTTGAGAACATCCTGGTCCAATCCCCTAGAATTTCTGGAGCGATGCCGACTTCCGCATCAGAAACGCTTCCGTTCA
CAGACTTTGTCGTGCCTGTTCTGTAGAGCTTCTCAAGTTCTGCTTTTTGTTGACCTGTTGCGTCGCCAACAGAAACAAGG
AACCTTTTGCCTTTGAGTTCTGCAACATTTTGTGGCTCATTGTATGGGACAGGGAAGGATTTTTTCCCAATCATCTGGAG
AGTTTTTGTGAGTTCCTTGTCAATGTCTGGGAACGACCGCATGGCAAAAAGCTCTGCATACATGCCATTTCCGAGATTAT
ATGAAAGCAAACGACCTCCGAGACGTTCTTCTTTTTCTATGAGAAGGATTTTCTTGTTTGGGAAGAACGTGTTCATCCTC
CAAGCAAAAAAGACCCCGGAAACGCCTCCTCCAGCGATAATAAAGTCGTACATTATCTTCACAAATATTTTTCTTTGACA
AACAAAGAAAATGCAAGAAGAGACTTTTATCTTTGACGGGAAGAAGCACGTCTCTCTCGATGGGAGCATCACATACCTTG
GAGTGGAGAGGTGGTTTCTCGATGAACTTGCAAAGTATCTTTGGAACGAACACTGGGCAAATTTGAGGTGGAGGGAGGTG
GGAACAGAAAAATTCATCGCTTTCATCGACGAGAAGAAAGCACACATTGAGGGTGACATCGAAAGTCCTCTTTCTATTCG
AATTTTCTCAAATGGCGCACCTCTCCCAAAGAAATTTAAAATGGACGATGTCCAGAGTTTGGAAGAGACAGTTGGCGATG
AGCTGATTAGAATCACGTTTTCTTGATGAGATTGCCCATTAAAGCAACCACAATGAACTCTTCTCCTTTCATTCCTCAAA
TCGGACATTCGCCTTCTGCCGTCTGTGATGGCACGCTTTCTCACAAGGGAAACAATTTGTACGGATGTGAGTGCGGCGGA
GCTCTAATTTCTGCTTTTGATGTGCCTTTGTCGTCACAATCTGTCATTGCAGAAATTATCGCAATCAACAGGGCGATGAA
TAACAAGAGCAAGACTCTTGAACAGACCCATAATGAGGCCAGAAAAATCATGCTTGGGGGAGCGCGTTAACGATATTTTA
TTCAAAGATAAAATATGGAAGGAGAGACTTTCGAGTACGACGGAAAAAAACACGTGTCTCTTCGAGACGGAGGAGTCTAT
GTCGGTGTGGAAAATTGGGTCGTTCACGCGATCGCTGAGGCGCTTTGGGAAGACCATCTCAAAAATTTGAGATGGAGGGA
GTACGGTTCCGGGGAATTTTATGCTTTTGTCAAGGAAAACGATGACTACATCACAGGAAATTTGGACGACTACGAGACGG
TGTTTGTAAATATCCTTTCTGTAGAGAGGCTTTCCTCCCTTCCGAAAGTTCCTTTTTGGAGAAACAAAAAGATCGCGCGA
ATGGCAGCTGACAGATTGGCGATTGGGTCTACGAGAATTCAAGATTTCATCCATATTTTCTAGATAAAATATAAGAGAAG
AAATAAAAATGTACGTGTGTCTTGTTTCTTGCGCTGCGAGAAGGCTTTTTGTGGTCAAATTTAACGGACCTCTCGATGAG
CTGAAAAAGAGACTCGGAGAACGAGAAATTCTCCTTGCCGCGTCCAAACAAAAACATAAGGATGCGGACAAGGTCCTCTT
GAGAGTTTTCGAGGCTTTTTATGGAGCGAATTACCAGAGAATTTCAGAGTTTGAATTTCTGGAGAAAAGCGATGATGACG

TCGTCACAAGATTTCTCGATGCGACAAGCGGAACAAGAGAGGCATTTCACATCAAAGCCGCAATGGAAGCGATAGACAAA
GACATCCAAAGGCTCGAAGAAAAACAGAAGAACCTCCGTCAAAAGAAGAGGGCTCTCAAGGAAGAACTCGAGAGCCTTCC
ATGCCAGAAAAATTCTTTGTAAACATTTCATATTTAGATAAATATGAAGCGACAGGCAGAAGACTTTGCACAGCGAGTG
AGGGAAAAACTTTCGAATCATTGGGACATCTTTGTCAGAAAACTCTCAAAAGACGAATATTACGTCACAGACCTTTCCGG
TTGCGCGTTCGAACTTTTCTCTGAAGGAAAGGAGAATGGGAACATCGATGCTTTCGTCTCTGCTATCGAATCTCAGATTT
TCTACAAGACATTTTTAAGGCTGGAACAAAGGCTCGGAGTTCCAAAGGATAAAAGAGTTTCTTTGTCTGAACTCGCCGGT
TTTGTGGAGTAAGATGCAAGCGTTCCTCTCTCCGAGAGAATTTCTTAGTTTCTCGTTGAACGGAGAGAGTTGGGAAAAGT
TTGTCGACGTGTTCCTCGGTGTGGTAAACTACGAGAACGGAAGGGCTTCTTTCCTTCCAGACGGCACAATTCATGGCATA
TTTACACAGAAAACACAGAGAAACACTTTTGTGGAAACATCGTACTCTTTTGGCGCGTTTGTTTGGGAGACAGAGAGAAC
AGAGAAGGGGAATCTTTTGAGAGAGAAGAGAGAGATCGGAGGGAGGATTTCTGAAAAGTTTTGGAAGAGAGGGGACAATG
AAAGGGTTCTCGTTTCGTATAAAAAGATGGATGGAAACCTCCAAAAATCCAAAAGTTTCTGGAGGTCGGGAACTCCTCAA
ACTCTGACCAAATTCGTGAATGGCAAAGAGCACGGCAAAGCTTTCTCTTGGCACGAATCAGGAGCTCTTTCAAAAATCAC
AGAGTATTTTGAAGGACAGCCTCATGGAGAATCTCTTTCTTTTGATGAAGCGGGAAACGTTATCTCTTCTGGCTTCTACA
GCCTTGGAAAAAGAGAGGGTGTTTGGATAACCTCTTCGTCTGAAGGAAAGAAAAAGCAAGTGTGGAAGAGAGGAAAACTC
GTTTGTCCCACACGTGTCTGGGAATAAAATATTTTTCTTTGAATAAAATATTATGTGTCGTCTCTTTTTTCTCGTATGTA
ACGATGTCCAAACAACAACCTTCCACAAACGGTCAGGTTGTTGTCCCCACGTATTCGGAACTTCCGCGCGTGCAAACAAA
AGGGAGTTTTATTTCTGTTGGTGGAAATCTCTACATCTCCGATGGGCTTTCTTGGCAGGTGGCTTTCGCATCCGGGAGGA
AGCTTTTGGATTATGAAGACTGGACGACGTTCCAGCTTTCGGGACCTGCTGCCAAATGGACAGCTTTCGACCCGTCGAGG
ATGGACGCGACAGTCACAAACACAACAGGAAAACTAACACTGAACGAGACTCCTCAGTCTTTCACGCTGGCTCCCATTCC
GAGCACCCTCTCGCACATTACGTATCTCTGCTATCGCAACGGACCGGACCTCATTCGCTCAAGCGGAGCGGGAGCGAGCC
CAACTGCCAGAGGAGCGTATTCGGCTCCCGACGACGGAAGTGAGCTTGTCTTCGAAACGAGCATCGCCGTTTCTCAGTAT
CTCCCCATCGGTTCTACCTTTGTTCCCGCTGTGGCTGCTGAAATCACAGATTACAAGACAGACATCAGAACAGGCGCTGG
CTCGTTTGCTTGTTTGGACGAAGAGACGTACATTGTGTATGACGTCTTCTCTGTGGAAGGAGAATTCTTTGTTGTGGAGG
AACGTTTACCATTTGGGAGACCGAGTTTTGGCGGAAGCCAAGCGACAGAGTACGCAGCGTACACAACACTCCACCCCTTG
TGCAAACGAGGAGGAAACAGAGACCCTTCCAAACCTGTCGACATGCTCCAGCAGTTCGCAAAGGTTGCCATTTGCTACAA
CCGAAGCAGAGGATACATCCGCTTTCTTGTAGAAGGAGAGGAAAAACTTCGCCTGGACAAGATCGGAATTCCTCCCACAT
ACGGGAGAGTCCTCGATGAGGGAGGAGCGGCAGCCGTCGTCGCTCCCAGGCAATTTCGCCCTGGGTTTGGAATCTTTACT
TTGATGGATGCTTCAGCTCCTCTCGCAGCTCCCGGAAGCACGACGCGAGGAATTGTGCGTCTCAACCCAGCAACTCCACC
ATTCAACTACTACAACACCACCCAATACGACGCAAGGGGAGATTTTGTCTCCCAAACTTTCGCGTCCACGACATTGACCG
ATTGTTATGCGACCCCAGCCGCACAAGGAATTTCCATGTCCCTTTTGCCTTTGAGCATCTACACACAACAAGTGGTGACA
CAATAAAAAATATTTCAAGAAATATTTTTTATATCAACCGACTCTTTCAAGAGAAAAAAGTTATGTTTAGTGAGATGGAG
AGACCGAGACACGGAGAGATTTCTTGCGAAAAGGAAGGATGCAAAAACAAAGCTTATTGGGAGCTTCTGGAAGAGAATTC
TTCTTTGGCGTTTGTTTGCGGTGTCCACTCGAGGAAGAAAGAGAGAGTTGCTCTTGAAAAGGTCCCACAAAAAGAAAGGA
AAGAGAAGCTCGAGGAAACCCTTTCATCTCACAGAAAGACAGTGAGGAGGGCAGCAAGAAAAAACAAAGGTAGAGGAAAT
CTCGGCTTGTTCCGTATGAGAATGATGAAGGAAGTTCCTCTTCAGGAGGGGTGGCTCAACGTTTTCCCAAACTTTCGCCA
CCAGAATCGGAGAGACGGCCTTGGTTGCATGTCCCTTTCGCCGATGTCTCTTGGCCCTGTTGAGCATGTGCACCCAGAAC
TTCCCCCAGCAAAAAACATAGAGAATTTTTACCAGGGGTCGAAAGTCTTCTCGGAAGAAGTAGACAAAGAAAAGAACCCG
ACAGCTCTGTTTTACGAGAACAGAGAGAGGCTCTTTTCTGATGAGGTCCCGCATCGGCACAAGTACAAAGGAACAGGGAA
GAACAAGAACATTCCACTTTATTTCTTTGGACGAACGTCAATGGAGAAGAAGAGAGGCTCGGATACGTGGAATCAAGAC
AAACATATTGCAACTTTTTCGAGAGACTCGCAGCAGAAACAGAGGATTTCAAGGAACTCTGTCATTTGCAAGATAGTGGT
GTGAACTTGATGTTTTGTGGTTACGACGCCCACGACATCAAATCACAAACTCTCGAGGAAGCTTACCTCGATCCGAGTGT
TCCTTTCGGCCACGAAAGGGTCCTTTACACGATGCTTGCGCTCAGAGACACCCCGGAAGAATATCCTTGGAGGAAGCACA
AAACTCTCGAATTTTAACTTTTTCATGAAAAAGTTAAACAAGTGCTGGAGGAGCCCAGAACGCGAATCTTGTGTCCATGG
CTGTCGACGCCTGAGAGACAAGAATCTCTACGGCGACCTTTGAGAAAAGTTGAGCCTCGTTGTCTCCCTTTAAAATTTCG
GCGTTTGGGTAGTTCTTTTTTATCCATGTTCTTGCTGTCTTTTTTAGGCACTGAACGCCAGCGATCCCCAGTTTTTTGCG
GAAGGATGTGCTCAGACTCGAGGGATAGAGAAAACACGCTGCGAGTGCACAAGAGGCTATTGTGTTTTTCGGTGTGAACT
TTTGTTGGAGAATGTTCAGCGCCAAAAGAGCGACCTCCTTCCTTGTTGTGTCGTTGACGCCAGAATATCTGAGAAGTTTG
TCTGCGTGCTCTTTCGTCGCTTTCTCTTCCCTCCACTGACAAAACTCTCCGAGGGTCTGTAGCGCGGATTCTGAAATTTC
TGCGATATACTTTAGGGAACGAAATTTCCTTCTTGTTTCGTTGGAAATTTCTGCGGATGGCGGATTGCACCCATAAATAT
TGGAAACCACACGAGAAGCCGTCTTTCCGGAAACGTCGACTCCCCTGGCAAAGCCCTCATGAAGAGATAACAAAAACTCT
GAAGGACTTTTCTCGAAGACTTGAAAGTCTACTGCGTTCTGGAAAATGACGGGCGCCTCTTGAGACATTTAACACAAGGG
AGTCAACAAGAATATTTTTTCGATGAATGTAAAAAAATATTTTAGAAATATCCTCGAACGCTCGAGAACTGCTCAAAAGG
AACGAGCACTTCCGATGTGTCTTGGGTGCACCCCTTGCATTGGTCTTTGGTCGTGTTTACTGTACAACATTTCGTGATAC
AATACGCTTGAAAATGTCCAGAAATCCCAGATTGTCTGAACATGAAAGAAGCGGGGCACGTTCCGAAAAACACCTGTCCA
GAGACAGCGACAGAGCGGCCAACTGCGTTTCTTACATCTCCCTTTGTTTTACCCACAAAGATGGCCGGAGTGAAAGGACC
TGCTCCCTGAATGCTCGACCCAAGCATCGCTCCGAAATACTCTTGCGTGAAATCGGGAACGTCTTGGATGACCGTCTGCG
GGTAGTAGTAGATTGTCTGCCAAGGAACCCAAGTGTATGAAAATTGTGGTCTGTAGCCCCACGGTCTCCAACCTCCAGGT
CTCCAGCTGGGGCGAAACCCTCCTCTCGGACGAAAAGAACCTCCTCTTCTCATTACAAGTTTTTTGTCTTGAAAAAAACT
TTTTATATCATGGTCGAAAGACGGCACTCGAAACAACCTCCTTCCATGGAAGGCCTTATTCCGCAGCAGGGAGAAAAATT
TCCTTGAGCGCACGCAGGAGGTCCGAACCTCGACGCTCTACAGAAAGCGCGATATGTGCCGTCCGCCTGTTTCAGAAAAT
TATACAAAGGGGGAGAACAACTGAACGAACTGAACTGAGCAATGTTAACAGAGAGCCTTCTGCCCATCTCGTCTCTAATA
TCACAATTCGATGGACCTGCGAAAACAGTTGGTTTGTACCACAACACAGTCTCTGAACTTCCGAGCTTTGGTTTGTCCCT
ATAAGAGAGCGGTATCAGTCCCCGGTAGTAAACGTCAGTTCTTGGAGGGTCGTTGCTCGTCATTACAAAACTCTCGAAAA
GAAAATACAGAGTTTCAAAGCGTAAAGGAAAGGATGTCAAACAGAAAGGACGCCGCGAGAAAAGACAAAGAAAGGAGGAG
GATTGTGTACGACTTTGCCACAGGAAGGCTGTTCCTCGCGGAATCTGATGTCGAGATTTCTCCCTTAATACCGTCTTCTG
GACTTTCAAAACAGGCGAGCCCCAACGGATACACCATCGAGAACAATGTCCTTTCGCTGTGCGAGGCGTCATCAAACGGT
CCGGGAATCGTCTACGCTCTCCAAACAAACAAAAACCTCGGATTCGGAAAGGGGAGTTTATTTTCTGTGTTGGAAGGAAA
AGAAAACTCTTCTTTCGGGCACGGTGCAGGAAAAAGACTCGCAGAGGGAGATAAAAATTCCCTGTTTGGGTTTTCTGCCG
CTGACGAACTCGTGGAAGGAGAACTTAACGTCGCTGTTGGAGCATCATCCCTTTGCACTTTGAAAAAAGGGGATGCAAAC
GTTTGCATCGGCGTGTTTTCTGGAGATGGAATGATTGCAGGCTCGAGGAACGTTTTCCTCGGGTTTCATTCAAGAGCGAA
AAAGCAAGACATCTCTTGCAGTGTCGTCATCGGGTACAACGCCGTCGGAGAGAAGAGCAATGAACTTCTTGTGTCTGACC

AAATTCACTCCTTCCGTATGAGAGGTCTCGAGATGGAGCCTCGGTCGTGTAACGCAACGATGCTTTGTCATGACCAGACT
ACAGGCCTTCTCTCACCCATAAAGTATCCTTTGAGAATGAAAAATGCCAGTTATGTGGAACATGACACCATCAAAAAGTT
ACTTCAGACACCTGTCTGCTTTGACAGTTCTGGGTCGACGTTCATTGAGCTTGACAAAGCCAAGGACATTCCGGGTGTAA
CAACCAAAGATGCATCGGGACAAGTTTCTGGGATAAACCACCCAAATCTCATCATGACCATTCTTGCTGCTGCACAGGAA
CTAAGGGAAGACATCTCCACCCTCAAGAAAAAGGCAAAAGAAGTTGTTCCAGTTTGTCCCACTGAGAAGAGACTTTGCGA
CCTTGAGGCCAAACTTGGAGAAGACATCGACATCAAAAGAGAGCTCTCTGTTTTGACCGAAAAGATGGGAAATTTACCCA
CTGAAAACTCAGAGTCGATAGAGAGACTTCATCAAAAAATGGTGAAGCTGGAGAAGAAACTTTTGGAACTTGTGGCGCAA
ACCAGCCTTGAAAAAGAGGCTTATTCCAAACTCTCTGAAAAGGTCGAAACGCTCTCAGCGTTCGGGACGAGTCAAAACAC
AAAGACGCAGAGCTCTATTCTCCTTCTTTCGGAAGAAAACAGAGAGTTGTTCGAGAGACTCTGTTCGGCAGAAGAGCAGA
ACTTAAAACTTTCTTGTGCTCTCAAAGAACAAAAAGAAGAAACAGACAAGAAACTACAAGAACAAAAAGAAGAGACACTT
TCTGTCATGAAAAGGCAACACGAAGAACTGATGGCTCTGTTGAAGCATCAACAGAAAGAGAACAGACCCATCTCACCTCT
GCAAGTTTCAACAAGCGGCGACCTCAGAAAGCGCATCATCCCCATCGACAAGATTTTGGATGATTGGGAGAACATTTCAT
AATATTTTTTCGTATAAAAAATATTATCAAGAGAAAGAAAAAGATGAACGTCGACGCCAATGCAATCCTCAACCTCATCC
TGAATTCTGCTCTTCAGCAGATGAACCTCAACAAGGAGAACTCTCAGGCGCAGCCTCAAACGAACAATTCTTCGTCTTCT
CGAGAAGACGAGAGGCCAAACTTGATGGATCTTTTTGAACCCACCCCTTCACCTCCTGTCGTTTCGATGAAAAACAAGCG
TCAGCTTATGGCGCTTGGCGTTCTTTTGATTCACTTGCAGGAGAACGGAGAGCTCCCCTGCCGTGTGAACTCGGCACTTG
TGGTTCACTGGCCTGAAAACGAGCATGGAAAATTTGAAGAGTTTGGCGATACCATCCTCAACAATTCAGAGAACATTTTG
GGGGAAGGAACCACTTCCAGCGAGTACGTTGGAGATGTCCTTAACGTTCTCTCACGCGATTAGTAACTATTTCTTTTGAG
CAAAAGAAATATTCCAAAGAAATGGAGGACCCGATGGACGACAAGATTTCGAGACAGGACTTTCTTCTCTCTTACACTCA
AAGGAAGAAGGAAGGCGAAAATCCGCAAATTTCAAAGGTCAAAGACCTCAACAATCAGATGCTCTCTGATTTCGCAGACG
TTGTGTTCACTTCTGATGAAAAGGTGCCTCGTTCAGAGGTAGCACAAAAGTGGAGAATCTACGAAGACGGCATCGAAGTC
AAAAGGTACATGCAGAACAAGAAGAGAATCATTCTGCCTTCCGAAGTGGCACAACGAAAGGCTCGCGAGTTTTTTGACAG
GGACATCGAAGACATCTACGAGTCGGGTTATCCACTCACAGTGCAAAATTTTAGCATTCTCGACTGTGAGAAAACAAGGA
GAATTATGGGACAAAAACACAAGAGAGTTTTTAGTTCCGATGACCCAAACGTTTGGTTCGAAAAAGCGCGAGAGTTTCGC
GAGCCTGTCGACCAGGAAGACATTCGCCGAGATTCTAAATTCAAGGCTCTGAGCCTCGCAAAGCTCTACATCTAAAATAT
TTTTATAAAAATATTTACATGAAACACATCACAAACATATCATCTCTCCAGTACCTTTGGAACAGGACAAAATCCTCTTC
TTTGAGGTCGTCCCAATTTTTTATGGACACGTGAGAGACGAAAGAGCCCTTTTGTTCAGAGTGCACGTTGAGGAGATTTT
TGAGCATCTTTTCTGCCTCTTCTTTCGTCGTCTCTTTGAGAAAAACGATGGGGATGTATTGCCTTTGGTTGACGTCAACC
CAAAACATCTGCGCTCCTTCGAACGAAAGGCCGAATTGCGCCTCGCATAATTTCTCTTTGAGGAGAGGCGATGTTTTGTA
CTTGCGGTAGATTACAAAGTTTTCTGATGTGCAGACTTTGCTCTTTGAATTTCCCATCTCTCTAAAAATATTTTGTCTCG
TAAAAATATTTCTTTCAGAGCTCCATTATTTTGTGGAAGAGACCTCTGCTCGCTTCGTGACTTACCACGACCACAAGGCG
AGAAACGGCTATCTCGTCCCTGAGATACTCGAGTATCTCTGTGTTCACCTCTTTGTGAAGCTGCGCGAGCGCTTCATCCA
AAAACAGAAAGTTTGCTCCCACCACCTCGTTGATAGCAAGGATGAACGAGAGGCAAGCTTTTTGCCTCTCCCCCCCAGAG
AGCGAGAGGAAGCTGTCGTATTCGTGACCTTTGTAAAAGATGTTTGTGCACATCTGGGTCTTTTTCCCAGACTTTGTCTC
TTTTGTGGTTTTGAGAGTCACGGTTATGGGGTCCTCGACAAACAACCTGTTGAGATGATGCCTCGCTGCTGTGTTTATGG
CAGACAGTGTTTTCTCGACTGCAAGAAAAGTCGCCGACCTGCTTTTCTCCTTCAAAAGAAAAGCGTCGATTGATTTTTTC
TGGTTCTTTTCCGCCATACTTTGCACTTCATTTATTTTCTGGAGAACTTGTTCTTTTCTTTTTTTCTTTTCGTTGTAACC
CTCCCATTGCAAGGCAACAAAGCAGAGGGTTTTGCTTCTCTGCAATTTCTCTTCAGCAGAAGAGAGCTCCTTTTGAAGGT
CCGAAAGTCTCTGTCTTTGTTTGGAAAGTTTCTCCAAAAAATTCTCTTCTCTTCTTTCGTTCAAAAGACAAATGCGAGTT
TTAATGTCTTTGAGTTGTCTCTCGAGCTTCTTCTGACGAAGGAGGGAATCTTTTGCTCTGTCGAGTTCTCTCTGTGCTTC
TTCTGTGGGAGGAACATCCACAGTTTCAAGAAATGCGTTCTCTTCTTCTGTAAGTTTCTCACATTTTTTCTCCATAGCGA
GAAGCCAGGATGGGACCTTTTCATCAACAGGGACGTGACTCTCAAGTTTCTGGGTGATGTCATCCAGTTCTTTTTGAGAT
TCAAACTTTGGCGAACTTTCAGTCAAAACACATTCAGATAGTTTTTCAAGAAGAGACTTTGCCTTTGAGAATTGCGCTTT
AGCGTGTTTGAGTTTTCTGGTCTTTTCTGCGAGAAGTTTCTCTACCTCTTTTGCGTCGAGGCCTGTGCTCTTGTGTTCAG
ATTTTTCCAACGCACCAGAAACAAATTTGAGACTCTCTGAGCAGCAAGGGCACACCAAAACAGCAGCTTCGAGCTCTGCT
CTCGCTTTTTGTTCGATGAGCGCTTCGACCTTTGATGAAAGGGATTTCTCTTTTCCAGAGAAAGAGTCCATGTTTTTTCC
CAGGTGCTTGGAATAGGAAGAAACGGAAGGGCTGATTTTTGTTGGGAACTCTCTCTTGACTTCTGCGTAGATTGTTGACA
GTTTTTTCTCCACTGCTTTCCTCTTTGACAGAAACGCATCGTACGACGTCTTTTGTTTTTCGAGCAATTCCTTCCTTGTT
TTGAGGTCAAAAATCTCCTCTTTCGAGAGGCACATTTTCTTCTTCTCTTGGAGCTCTGCAAGGAACGAGGAGAGTTCCTT
TTCGTACTTTTCTCTGTCAGCCCTTCTGCTGACTAAAAGAGCAAACTTTCTCGCATCGTCTCTCTTCTTTGTTAGAAGAG
AGAGAATGTTTTTGTTCACGGTTTCTCCAAAAGAATCCTCGATGCACTTTTTTTCTCCCTCTAATTTCTCCACAGACAGA
GAGATGTCTGTGTTTTTCTCTAGTTCGGATATGGCTGTTTGACACTCGACGATGCTCTTTTTCAAAATTCCGACTTGCTC
CTCCAGAACAAGAATTTCCTCCTTGGAAACTTCCACAAAAGGTTCTGTCGGCTCCTTGAGACCCTCAAATTCAGAGAGCG
ACTGTGAAAAACATTTCTCCTTTGTTACAAGTTCAATCCTTTCCTCTTCCACGCTAGAGATATATTTTTTGAGGTTCTGG
CGTTCTCGTTCGATGGTCTCTTTGTCGAGGCTGAGATTCTCCACAAACGCCAACTGTTCTCCTGGAGTCAACGAAAGGAT
GGAATTGTCCAGATTCTGGACAACGTAACAAGACGCCATAAATTCTCTCGAGTTCAAGACCCTGCTGTTGATGGTTTCCT
GGGCAACTTCTCCCTCATATTCTGCGTCTTTGTTTTTGTGGTCAAGAACAACCAGCCTTCCGGGACCTGCTTGTCTTCTT
ATCGAAAGACCATATTTCTCTGGGAGCGAAACGGACACAGAACAACTTCTCGAGCCAAAAGCTTGTGGTTTTTTAAGTTC
GCCATAGAGAGCCCAGAAAAATGCGTTCAACACTGTGCTCTTTCCTGTTCCAGAGGGAGCAGAGAGTAAGACAAGTCCCC
TTTCCGGAAAATTCACGTCCTTTTCTTTGAAACAGCGAAAGTTTCTCAGCGAGACTTCCATCTTGTTTTTCTTTCTCCTC
TGCGTTTCTCAAACTTTTTTGATAAAAAGTTTTTTATATCACTGTGAAATCCTCAAGAAAAAGCGGAACGTCGTCCACAA
GAAATTCTGTGACTTCTTGCTCCTTTGGAAAAGCAGAAGGGAAGAAATCTCCCTTCAGGCTGCACAGTTTGCCCACGCAA
ATGTCGTCGAGAGTTTTTTTCGTTCCTTCCCAAGAACCGAGTCCGAGAGAGTATCTCAACTTGTCAGTGAGGGGCGGATG
CGTCAAACAAGTTGGACAGGCAGCTAAAACTCCACTCAAAGCCTGTTCCATTGTTTTTCTACTGTTCTTCAAGAGAACTT
CTGGTATCTGTCTTCCAGAAATCGCGCATTCTCTTGCAATGGAGAAGGGCTCTCCCTCTGTTTCTTGAAATTTCCAAAAC
ACCCCTCTTCGGTACAACGAGTGCTTTTCTGGGTTGTACTCGCAAACTGGAATCTTTTCTTGGGGAAAGATGCAAAGGTC
CACCTTTCTTTCATCTGAGAAAAGTTTCTCTCTGTTCGCTCCAGGAGAAGGTTTTTTGTAGACATGTATTTCCTGATTTT
CCGCCAACCATATTCCGCCAAGATTCAACGTCAGAGCTCTTTTTTTGTTCACCGAAAGCACCTTCGCGGGAGAAGAGAGC
GACGACAAGAGAAAATCTCCTTCTTTGATATTCTCCGCTTTCAGAACATCTCCTTCTGACGTCAGAAAACAAACAGCATT
CGACACACAAACAAGTCTCTCCATTTCTTGGAGATAAAAAATATCTTTGAATATTTTTTGATGTAAGATGAAAGTGTTGT
TTTTGTGGCTTGTTCTGCTTTTGGTTGTGGAGGTTGTTGTTCTCTACAGCATGAAAAGATATGCACACTCGACAGAGAAA

GAGCCTTTGTTTTTGATGTTGACATTAGTCGGTTATGTTGTGCTTGGGTATCTCATCATCAAAATTCTGGAACTGCAAGA
GAGCATCGGTCTCTTCTTTGTTTTTCGGAACATCGTTGTTGCTCTTCTCGCTCTCGGCATCGGTCTCTTTGTTTTTGGCG
AGAAAAAGTTGTCTTTGAAACAGGTTCTGGGAGTGGCGCTCGGAATTGCAGCTCTCTTTTTGATAGCGTAGACCATGTAA
TGAGACAAATATTTCACAACTTTGTTGTGGTGCTTCCCTGGCTTTTGGCGATAGTCGTTTTGGAGCAGCTCTCCTTTGTA
TGTGCAAAGAGCTACGCAGGTGGAAAGTCTTGGTGTTTTCTTGTGTTGGCGGGGGTCGTGTACTCTGCCGTTGGCATCGT
TTTTGCTCTTGCGCTGAAAGCGCATGATAACCTTGGAATGTTGAACTTGATGTGGAACATTTTATCCACAATCTTTGCAT
TTGCCCTCGGCATCATCATCTTCAAGGAAGCGAACCTCAGCACAAGAAAGATTGTCGCTCTGGTTCTCGGAGTTGTCGTT
CTTGTCCTCGCAGTGTAAAATATTTTATTCAAAATATTTAGACTTCCATCTCCTCACCGAAATATCTCGAAACCTCAGAC
ATCGAGGGTCTCTTGTTCGGGTCTTCCTCCCAACATTTTTTGAGCAACTTTTTGATGTCTTTGGGACAATCGTTCGGAAT
GGAGAGACGTTTCCCTTCTCTCGCTAGCTCCGCGACGCGAACTGGGCTTTGTCCTTCATAAGGTTTCTTTTTGGTGATGA
CTTCCCACACAAGAACGCCAAAGCTGTAAACGTCTGCCTTTTCATCGTATCTGGAGCCGCTCAAAACTTCTGGTGCCATG
TAAGCTGTGGAACCAACGGACGTCATCGTCATGTTTTCTTGCTTGAGCCTGGCAAAGCCAAAGTCTGATATTTTCGCTTC
CCAGACGTCGTTCACGAGAATGTTTGAACTTTTTATGTCTCTGTGAACAACACCTTTCGAGTGAAGGTAGCAGAGACCTT
CGCACACACCAGAGAGAATTTTTCTCTTTGTTTCCCATTCCATCTTTTCTCCCAGAGACAAAAGAGTGCCAAGGTCGCCA
GGAGTTTCCAATACGGTCACAAGAGAGATGTTTGGCTTCTCGAAACAACACCCAACAAAAGTTGCGATGTTTTTGTGGTC
AAAGCTCGCCAGAAGAGCGACCTCTTCTCTGAACCTGAGCTTCGAGTCTTCAGACATATTTTGGTTGATGACGCGCTTCA
CGGCGATATGAATTCCCTTCCAGGTCGCAGAGTGCACAACGCCAAACGAACCCATTCCCAGCTGCTTTCCGAGAGAGATT
TCACGAAAGTTCATCACAAAACGGCTGGTGTTGATAGACAAGAGGCCGCTGACTTTCTGGTCTTCCTTTGCTTCGATGAA
TCTCTCTGATTCTTGGAGGCGTTTCAGGTCCCTCGTGCTTGAGATTCTGTCGATGGAAATTTGTCCAGGAGACGCTCCTT
CACAAAGAGCCTTGAGTTTGACCACAACATCTCCAAACAAAAGTGGAGGATTGTCTCTTTTGACAAGCACATTTCCAGAC
GCGATCCCGAAACGGCACTTTCCAGGCCAGGAAATTTGGGAAAGTTTCTCGAGTGTCTCTTCACAACAGAACAGAGCTGC
CTTTTCTGAACCAAAGACAAACAGGAGACATCCCTCTCCCCTTCCCATTCCTTGGATGTGGTTTTCGTACGCTCCGTACT
TTTCCGCCACTTCGCGACAACAGGTGTTGAACTGTGTCCATATTTCTCTTGCGTTCTCTGGTTTCTCTTCCCAGAATTTG
AACGCGTCCACAATGTCCACCAACGCAAGGGTGACGTACTCTTCGGGTTTGGGAGTTTTGTACCACCCCTTTGTGGTTTG
TGTGCTGCTCGAAGCGCTCGAGTAGTTTGATGTTGACTTTTGCGTTGAACTTCCAGATGCAGCCAGACCGGAAGCCCTCG
AAAGGATTTCCAAAAAAGTTGGCCTTGAGTCTGGCTCTTGGCTCCAACAACTCGTCATAAGGTCGATGTAACCATCTCCA
AATTCTCCTGTGTTTTCTGGGACTTCTGGCCTTTCTTTGTCTCGAATGACCGCGACTGCCACAGCGGCGCTGTTCATTCC
TTCAAAGGGATTTTTCCTTGTCAACATCTCCCAGAGAATAATTCCAAAACTATACACATCGGATTTTTGAAGATCACATC
CCTGCTCTTCGTTGAGGATCTCTGGTGCAGTCCAAAGAACAGTTCCGATTCCAGCCTCTGCGTCTTTGAGGAACGCTGCC
ATGCCAAAGTCTGAAACTTTCACATCCCACTTTTCATTCACCAAAAGATTCAAAGACTTGAGGTCTCTGTGCGCGATGCC
CGAACTGTGCAGGAAGTGCATCCCCTTTGCAGCTTGTGTGGCAATCTGAACGCAAAGCGCGGGCGGCATGGAAGGCATGA
GTTCGTTGTGCAAAACTTCGTAAAGAGAGCCGAGACCCATGTACTCCATGACGATGCACATGTTTGGAGGCTTTGTGCAC
GCCGCCATAAAAAGAATGACGTTTGGATGGCGCAAGTTGGCCATCGTTTCCGCTTCTGAACGAAAGTCTTGGATGGATTT
CTTGTTTGTCGTCTGCGACTTTAGAATTTTTATGGCAACATTTTGTCCTTTCCAAGACCCAGAACAAACCTTTCCAGAAA
AGCCTTCTCCAATGTCTCCAGAAATTTCCAGTTCAGAGATGTCAATGTCCCAATCCTGTCTTTTCTCTCTTTTGGAAAGA
AGAACGACGAGCAACACAGCAACGAGAGTACAACATAGGACGACCACCACAAACGTTCCTCCGAGAACTCCGCCCACAAT
CGCACCAACGTTTTCCGAACTTGAGGACGAAGAACTTGTCCCGAACTTGGCGTTACAAAAGGGAACGCTGATGTTTCCAA
GGTCTTCTCGTCCAGCATTGGAGAGGAAGACAGTCCTCGATCCTTTGTTGCAACACTCGAGACCCGAACCGCAAGAAGTG
GAGAAGCCGCCGATAGAAAAGCCGCCAATATCCACAGAAGAGAGCGAAAGAACGTTGGATACAATTTTTGAACTTGTGTA
TTCAAGGGACGCAACCCTTTCGACAAATTTGAAGTTTACAAAACTCCAAAATTCTGCTTGGGTGGAACCTTCAAATTCTC
TTGTCAGAGAAATGTTTTGGATGTCGTGAAAGGGGATGGAACTTGAGAAAAACACCCTCTGAAGAGAAACTCCCTGAGCA
GAAGCGGCTTCTGTGACATTTTCTGGAAAAAGACAACCAAGAAAAAGAAACTTTGAATTTGGGTACTCTGCAAAGACCTG
CGCCTTTTGGGCAAAAGCGTCATCGACAACAACGACCTGAGCCCCGACTGTTTTCCCAAGTTCGGTGAGAGCATCTGAAA
AAACATCTGAAAAACTTCCAAAGGGATCGAGGAAGCTTTGGTCTCCCATCAAACGAAGAGCCCCCCAAAGTTCCTCTTTT
GTCGAGAGAGAAACAGAGAAAAAGTTGTCTCTAAACTCTGAAGGAAGAGGTGCGACGCCAAAAACGGGAACGTTCACATT
TTCTGCTCTGTCCAGAGGCAAAGATACAAAGAGAGGGACGTTGTCGAGAGTTTCGAGCTCCTGGATATTTGATGCCTGGA
AAGTCTCCAGATTTTTGTCATCGTACGACAAGAGGATGAAGCCAGGGTCGGCAGGAATTCCCAGTCGAAGAGCAGAGTGG
AAGCTGTTTGAAAAGTCCAAAGTTTGTCCCATTTTCGTGGGTACTTTGAAATCGTCCGTGGTCGGATTGCAGCTTTTCCA
AGATATGGGTTTATCTGTTGCGTACTCTCCATAACCGCTGGGATACTTGAACACATACATTTGCCTCGTCGCAGAGTTGC
AACATCCGATGACGCCCGAGCAGTCATCCCCAAGAGGTCCAAAGAAAACCTCGCCTATCTTGATGATGGGATTTGAAAAA
ACAGTGTCTAGAAAATTTTGTCTCGTCACGTTTCCTGTCATCTTTTCAAGAATGGAGATAATCCATCTTCCGTTGACGTA
CCCTTCGATGGCTTGTTCGGCAGCAGGCAACCAAGAAGGGAAGAACTCGTTCCTGTCTCTCCAAAATTCGTTCTGGAGCT
TCGAATTGTTGTCCGCCAAGAGAGGGAAAGGGGAAAGGGAGTAGACGTTGTCAGAATCTCCTGTGAGCCAAAGTTCGTTC
GGAGTGTCGTTCATCACCAGCGTTTCGATGATAAAAACAAGGGAAGGGATGACTTGTTTGCACCTCCGAATGAACTCGGC
AGATTGTGGACCAAAGAGAAGGACGACAAGAGCTTGAGGATTCGCTGATGTGATGACTTGAACTGCTTCGTCCATCATGG
GACCAGAGATGACCGTGTAGTCGGGAATGACATGACCTGCTACCACGCGAAGACCGAAAGGCTCGATGGAACGTGTGAAG
TTGAGACTCGTGCCATCAAGGCCAGCGCCTGCAATAAAAGCTATCCGAGACACTCTGATGTTTGACGTGAGAAACTGTGC
CATAACGTAGAATTCTGTGTCGAAGCTGGGGCGAAGGTTGAGATAATTTTTAGAGAACGGAGTGAACAACTTCACGTCTC
CAGAGAACGAACCAAACAGGGGAGGAGGATTTGGTTGGAACGGAAGAGACTGAACTATCGCCGTCTGCATTGGAGCCAAA
CAAGTCACAGAGAGAAGTTGTGACTCTGGTAGAGTGAACAAGTTCTCTAAATTTTGCACTGCCTGAAGATAGTCCTGTTG
GTCGTCCAACACAAGAAACTCAAGGTTCAAAGTTTTGAGGACTCCGTTTCTGTCGTTTGCTGTTTTGTTGTTTGTCGCGT
AGATCGCAGTTTGCAGCCCTTCGATGAGGCCTCCGATTCCGACTGCGGGACCAGAACGAGCAGCCGTCGTCGCGACGACA
ATTTGCGAATCCACAACCATAAAGTTCACGAGAAACAAGAAACAGAGCGCCAAATTTTTTCGAACCATCTTGATAGTTCT
TTCCTCTGCTTCGGAAAAGACTTTTTGGGAACGATTCTTTTTCAGACTTTGTAAAAATATTTTCTTCAAAATATTTTATT
TCAAGAGAGACGAAAATTTCTTTCTCATCCTTTCGGCAAAGTACGCCTCGCTCTGATGCTCCCATTCACAAAAATTGTTG
GGGGCTGCTGACGCTTTGTCTTCTTCCTCTTCCTCCTTTTCTGCGTATCTGTTCGCCAGAAACTTTAGGAGGCCTTCCGA
CTTTTCGACACCATCCAAGGGCAACTTTATGAAATCGTCCTTGTGCGGAAGAGGGTACCTATTCACCCTCTTCAAAAACT
CTCTTGCTTCTTTGTCAGAAGAATTTCTAGAAACGTACACTTGGATAGCCCAGAGAAATTCCCTCATCCGATACTTGTCC
ATCTCCATTTTAAAATCCTGCCGTTGGATAAATCTCTCTGTTCAATTCTTCCAGTTGGAAGCGAAATCCTGGGTTGGGGT
CCACGCACTTTCTCTTGCTTTTTACAAATGCCAAAGCAGTGTCGTACGTCATACCTCTTTTGAGCATTAAGAACGCGATG
ACGACAGAGCTCGATCTCGAAATTCCTGCTGCGCAATGAACAAGGACGTCCAACCCGGCTGCGAGAGATTCCTGGATAAA

ACTCGCAGCAGAACGAAACACTGGTTTGATGTTTGTTCTGCTGGAATCCATCGCTTTGATGATGAAACGAGGGAACGCAA
CTTTGCGATCGTACTCGCAAAGTTCTTCTTTGCTCAAAATGGTGACGACAGCCCATTTCTTTTGCTCTTCGACGGATGAA
GAAGACAAAAGCAAAAAAGAAGCGAGATTGCCGAGATAGAGTCTCTCTGTGATGTGATTGTACGTTGCTGTGGTTGGATA
GTTCATTATAAAGTTTTAAAAATAAACTTTATTCGAGCTGCTTTGCGTCGGCAGAGAACACGATTTTGTCCTTTGTTTCC
AAAGAGTAGACAGTGTACCCTTTGAACACTCCCTCTTCCGAATAGAGCTCAACTTCCGAAAGCCAGCCGGTGCGGTACCA
TTTTTTGTGTTCACCGACTTTTTTTCCTTCCTTCAGCCTCACCTCAGTTCTCTTTGTTTTTGGGCACCACCACGTCGTCT
CGAGACCATGCTTCTCTCCGTTCAAAAACCAGGTTCTCTTTTCCAGTCTCTCCCTGTCCCATATATCACAAGGTCCTTCC
AATAGATTGTTTTTGTTCAAAAAGAAGGTGTTTTCGCCGTTTGCGTAGAATTTATGCCCAAGAGCAATAACAGCAAATTC
TTGGTAGTCAGAGAACCCTTCAGGGACGTTTTGCGAGTCTCCAGACACAAAGAGGCGAACGCTCGAAACGTACTCGTCGA
GTTCTGCGATTCCAGAGCAAGTCGAAACTTTCAGCGCTGACAGAAGCTTTAGAGACGCCATAGTAAAGAAAAAGTTTGAA
AAGTTCTCAAAGAGTAACAGTTTCTCCAATGTTTCGCATTTCAGACCTTGACTTTTCTCGTGAAAAAAGTTGTGACTTTT
GTGGGAAAAGGTCTCTCTTTGTTTTGGAATATTCCAGAGAAGAAACAAAGGTGTTTTACAACGTCTGTGTCGGCTGTCTC
AAGAAGAAGGAGAGGGAGATTGGCAAAAAGATAGAATTCCGCATCGCATAAAATATTTTCTTCAAAATATTTTTCTCCCC
TTGGTCTCACACCGAAATGTTCTCGCGTCCGCGAAGTTTGCAAACGAACGGTTTGCGTTCGCCTTCAAAATAGAGAAGCC
ACTCTTGGAGTTTCCCGTCCTCGGAATAAACCTCTGTTTCTGTGGTGCGCCCAGCAGAAGTCCACCTTGTATGTTGACCC
ACTTTTTTGCCGTTTTTCCACCGCCCTTCGTATATTTTGCCCTTGATCACAGAAGACACCCACGTTATCGTCTCGTTGCC
ATTCTTTTCGCCGTTTTTGTACCACACCTCTGTTTTTATTCCGTGCTCGGTGGAAACGAACGGACCCTCTGCTTTCCCGC
TTCTGTTGACAAAGAAAGACGTCATTCCGCAAGAGAGAAATTTATAACCGAGCGCCCGCGTTGCAAAGTCAGCATAAGAC
GTGTATCCTAAAGGGGGAATGACACTGTCTCCAGAGACAAAGTTTTTGATGCTCTCAACATACTCGTCGAGTTCAGGAAT
CCCCGAAAACTCTTCGACCTTGAGAGAACACAAAAGCTTCAAAGACTCCATGTAAAAGAAAAATTTCGCATCGCTCTCTG
AATTCGGCGGTTTTTGGAATTTTCTTGAAAACCCATGGAACGGCGAACTCATAAAATATTTTCTCATAAAAATATTTTTA
TCTATCGAGCGCAAGAATTCCCAACAAGATGGGAACGCTGTAGAAGCACCACAGCGTGGGAATTTCATGGATGTTTCCAG
CCATCCTGTTCATCAAAACAACTTGCGCACACCAATAGAGCAACTTGAGCCAACCTCTCCTCTCGTAGATGGAAGGACCA
AACCACAAAAGAAGATATGCTCCCCAATTCATCTCCACGCCGTTGAAGCTAAACAGCGGAAAGGTCCAGAAGAGATGTGT
CGGTCCCATCCTCGTGCATGTATCAGTTCCGACCATAATTTCATGCTTTGAAAGTGCCTGATAAATCCTTCCTCCACTCG
GAAGACTGCTCCTCGGAACCAGACGGACAGAGAAGAACACAAGCCACAGAAAAGACAACCACATTGCGCACGAGAAAACT
GCCCTTTGGCGAACTGTGGGGGATTTGAGCCATCTGTAAAAATTCCAAGCGAGCGGCTGAGCAACAACAAGACCAAACGC
AGCGTACGAAAGAGCAAGGTTCATTGTCGCTCCACAGTCGTTCACAAAAAAGTGTTGTGCTGTTTGAAGCCATTCCATCA
AGCTGTAGAGCGCAAAGATTATCCACATCGGACTCCTCTTCCTTTTGGTGGCCGTTGCCATCGCTGTACCAGAGACAGCC
AAAACAAAAGAGAGAAGCGGAGTGTGACACATTTTATTTTTGTCTCAAAAAATAAAAAGAAAGTCTCGATATGCCTCAGA
TGACATACATCAGCAAAGGAAGAATTCCCGGAGAGAGCATAACCGGCATATCGGAAATCTTGAGAGGAATCTTCGCTCCA
AGAAGAGCATCCAACACAAGAAAAACAAACTTTGTGACGCCCCTCTCTTCGAAAAAAGCAGGAAAAACCAAAAAGAGGAA
AAAGCACCCCCAATTCAGTGTGAAACCGTCAAGCGGAAAATCCCAAACGACAGAAAGCTCTTGGTTCACCAATGTAATTT
CTTCCTTTTCTCTTGCCAGGATGTCGAAAAGTTGCCCCTTTTGCCTAAAGAGGGAAACGTAAGAAGAAAGCCAAAACACG
AGAGACATCCAAGTGACGACCTCGAACGCTTTATAATTTCCTGTCTGCCTCAAGCGGCAGAGATTCATGACAAAAGGCCA
AGAAAAGACCAGAAAATAGCCCAACAAAACAGACGTTTCCTCGCCAAAAAGCTGCGTTCCGTAATCTTTGTTGATGCTCA
GAAAAACTCGCAGAGCGAGGAGGGTAGACATTTTCCCTTTCTTTTTGTTTCCTATAAAACAAAAACACGGAATGCCAACA
CAGTAGCCACAAAAAACAGAAAGATGAACAGAATCTCGAGGTTTGCACTTGTGTGTTCGAGGTAAAACACTCTCTTGCTC
AGAATTTTCTGCTTCTCTTCCATCTTTAGGAGCTGTGCAAGTTCGGGAAGTTTCCTGTCTTCCGATGCGACAGAGTCAAA
GAGCGATGTCGCATGGACGATTGAGCCGCAACAGAAACTGTTCTCTCCAATCTCTGGAAACGTTTCAGAAAAGCTCTTGA
GAAGTTTCCGGAACGTTTCCCTGTCGTGGCTCTCGATAGCCCGATATATAAATTTTTCCTTTAGAACTTCCCTCTTCTGC
TCGAATTTATTCAGCTTGAAACAACCAACAGAAACGACGACTGTTTCATGGCTTTCTTTTGCCTTTTCTTCCGTTGTCAA
AAATATCAGCCTCATCTTTCCTATCGAAAAATAGAGAAAGATTCTCTGGTTCAGTTTCGCTAAAGAAGATGTATCAAATT
TATACCGACGGTTCTTGTTTGGGAAATCCAGGAAAGGGCGGCTACGGAGTCGTCATATGTAAAGATGAAGAAGTCGTTCT
GACATTGTCTGGGCACTTGGCGGAAACAACAAACAATAGAGCAGAAGCAGAGGCTTGCATTGTGGCTCTTTCATGGCTCT
CAGAACCAACGGAAGTTGAAATTTTCACTGATTCGCAGTATGTCGCGAAAGGAATGACGGAATGGCTTGATGGATGGAAG
AAAAACGGCTGGAGAACAGCGAACAAAAAACCTGTTTTGAACAAAGAAGTGTGGCTTGAACTCGACAGGCTCAACGCAAA
GCATAAAGTCTCATGGCGGTGGATTCCTCGCTCTTCCCACAAATTCAATAAACTTGCGGACAGCTTGGCAAACGGGGCAG
CAAAGGGGTAATAAAATATTTATTCAAAATATTTAGAAAAAATCTTCCTGTTCTCTGTCTTTGGAAATTCGACGCCGCAC
CTCAAAATTCCTCTGTGAGCATCTCAGATGCCCTTGAAATCTGTTCGCCGTGGGGAAACGCTAAAGTCAGAAGTTCCGTT
TCCTTTTGAGATACTCGACAGTTTGAACTTTTTTATCATCGTCCATTTCTCCAAAGAAAAAGAAATCCTTCCGGAGAATT
TTTGAGTTTCCTTTTATGATGGAGGCGCTTCCCAATGAAACCCTTTTTCACATTCTCTACTTTTGTGGTTCCCAAGAGCT
CAAAAATTTCGAAAGAACTTGTCGTTCAAACAGAGACGTGTTGAGATGTGGTTCTTTCTGGTCCCTTTATGCCAGAGAAA
GAAAGCATCCAAAGAAACCAGAAAACATCTCGCAAAAAGAGTGGTCTCTTCGGATGGAAACGCAAGTGTCTGTCAATGTC
AAAAATCTGATGGGAGAGCCACATTTTGGATATCCTTCATCGTCTACGCGCGTTCTGTGTCAGAGGGATTCTGCGAGATG
TCTCATCGATGCGATCTCGAGATTGACGCGATTTGCTCCCCACAACATCCTGCTGTACGCTGGAGGGAATCTTCTGGACA
AGAACATGCCAGTCAGAGGTAAAAGATTCATCAGAAAGTCGAGGCTTCGCGAAGACAGAAGAGTCAAACCCATCGGTATT
TCTTTCGTCCTCATCTGAAAGAACGAATATTTTATTAAAGATAAAATATTTTGAGACAAGATGCAAAAAATTATCGCGTG
CTATCACGGAGGTTGTTCCGACGGAACTTCTGCGGCATGGGTTCTTCAAAAGAGATATCCAAACGCAGAGTTTCACGGAA
TTCGCCCATCAGAGACGGATTTTTCTTGTATCGACTTTGAGGGAGCGATTGTTTTCTTTCTGGATGTTTCTCCCGCAAAT
TTTGACGAAATCCTCCCAAAGGCAGAGCACGTTTTTCTTTATGACCACCACGCAACGACCAAAAAACTTGTGGAATCTGT
TGGAACGAACGAGAAGTTGTCTGTTGTATTTGATACGAAAAGGTGCGGCTGTCTCATAACTTGGGATGAACTTTTTCCAG
GAGAGGAAAGACCTTGGTTTCTCGAATACGTCGACGATAGAGACAGATGGCAATGGAAGCTTCAAAACTCGAGAGAAATA
AACGAGGCCATCTACTCTCAGGGTTGGATGGAGAGGCTCGACGAACTTTGGAACACAGAGAAAGAGTTGCTCATCGCATC
TGGGAGAGAACTTCTGCAAATAAAAGAAAGACACATCAACGATGCCATCCGCGTTGCGGTTCCAGTGAAATTTTGCGGAT
ACGAAATATGGCTCTGCCAAGCCAAATGGCAGCTTCGTTCAGAAGTCGGAAACAGACTCTGTTCTGTGCTCTTGCCGAAT
GGCAAAATTCCGTCTTTTTCTGCCATTTGGAAGCACGATGAGGAAACTGGAGAAATATGGGCGAGCCTTCGAGGAGAAAA
AAACTCTCCTTGTCTTGCGACTCTTTGTGAAAAATTCGGGGGAGGGGGTCACCCGAAAGCGGCGGGTTTCACCATCGAGT
CCGCAGAGGAGTTCGAGAAAATATTCGAAATAATTTGGACAAATTATTTCACTGAAGAAACTTTGTGGATGTTTTTTATC
TTTGAAATGTCTCTTGAACAAGTTCTCGACGAAACGCTCGATATTTGGATGGACGCATTCGACGAAGAAATTTCTGATAA
AAGGAACGGCGCCAAAATCCTCGAAGGTTTGCGATACTCTCTGGCTCTCGTTCGCAGGGAAGACGTGGCAAGGAGAAAAA

GAAAGTATGAAAAAGCGCATGGAGTTTTTAGGACGACTCCCAAAAAGCTCGCAAAACTCTGCGAGAGCAGAGAAAGAGGA
TTTCTGACGTTCAACGAAACCACGCGCGCTTGTGTAGGTTACGACCCAGAGGAAAGAAAGACGAAAAAGAGGATGAAGAG
GTACAGAGAATTGTCTGAGAAAAAGAAGGAACTTTTGGAGACCTTTCTTTCTTACGCTCCCAGCAATCGTCCCGTCTTTT
GGGATTGTAAAGACAAACTCTGGTTCACATACGATAAAAACTGTTACGGGGATGGGAAAGGAAAATACATCACAGTGAAG
AGGGGGAAGCCGAGTCTGACCCACTCGAAAGGACCGCTGAAGGTGAAATTTTGGGACGGAGAGATTTCTCAATCTGGGGG
ACTCTGCGATTGCATCGACAAGATTTTGTAGATATTTTTGTTTGTAAAAAATATCAAACCAAGTCTCCTTGTACTTTTGC
TCTCGACGAAGACCATCGAAAACACTTCTGTTTTGATGTGCAGCGACACGTAAAAGCCATACACTATAATTTATTTCAAA
CAAAAATAAATTATCCTTGAAAGAAGCCTAAACACTATAAAATGCATCGCTTCCTCGGGAAAAGAGAACTCGTCTCTTTT
TGTGCTTGCGACGCCCCAGAGAACCTCCCAAGAAAGGAAGATTATGTCACAGTGAAAACAAGAATGAACGTGGACGTCAG
AACGAGCCACGTTCTTCCTGACGGCTCCATCCATTTTTTAGAAATAAAATATGGGACAACTGTGGTGAAATTTATAAAAA
ACATGGCAAGAAATTTCACTCTGAGGACAACACAAGAATTCAAGGCGGGAGTCCCCCATGGAAAATACAAAGTTTTCAGA
ACGGATAAAAACGGGAAGGAAGAACTTCAGGTCGAGGGAACGTTCCGAAAAGGGAAGGCTCACGGCAACTTTTTCATCCG
CGGAGTAGAAAACGCCATTGAGCACACATGCACTTTTGTCAAAGGGCGAGTTTTGGAATTTTCAGAGGCTGAAGGGAGGC
ATGCTATAATCTCCAGAAACAAAAAGAGCGGAACATTGCACTACTTTGAGAAAGATGGGGAACTTTGTCTCCCTTTGAAG
GTCTCTCGTTACCTCTTTTCTGAATTCAAAAGAGAATGGCCCTCCTACTCCCATCTTTTATTCGACACAATTTCACAAAA
ATTTAAAACGTACGAGTACCGCACACAGCTCGTCTTTCCCTCCTTTGTCGATACACAAGAAACCAAGTTCAGCAACATAC
CCGGACACGACAACTTCAAACCCAACGAGGTGCGCGCTACAAATTATTTTGTGTGGGTGGGATGTCCATACAAGGAAAGC
TTGCGTGAAGAAGAACATCCAACAAATCCCCCAACGATACTTTTTGTTTAAGTTTTGAAACAAAAACATGCACAAGTTTC
TGGAAAGGAGAGAGGTCACTTCTTTTTCTCTGAATGGGAGCTTTTGCCCAAGGAAACAAAACTTTGAAACCACAACGCAG
AACGAGACGGAAACTTTGACGGCGCTCCCCGATGGCTCTGTCGTCAGAAGCTATGCACAGACCCATTCGCTCCACGGGGA
AACCATCACAAAAGAGAGAAAATACAAGAAAGGCAGACTTCACGGAAAGTTTTCTCTTTTTGTGACTTTAGCAGAGCAAG
AGACGAAACGAGTGGAGGGCAAATTCCGTAACGGAAAGCTTCACGGGAGGTGGACACTTTACGATAAGGGGAGGGTCGAA
AGTTTTACGTTTGTGAAGGGAAAACTCATGGAATGGACCAGCCATCACGGAGAAACGCACAGCGTGATTTCCCACAACGA
AAAGAAGGGAGAGCTTCATGTTCTCGAAAAGAAAGGAGAGAAAACATTTCAGTGCCACTTTTTTGAAATGATGGGAAAAC
GCGAGATACATTCCAGCTTGGGTGTGGCCGTCCCAGCAAGGACCTACAAAAACTACAAATGCTTCACGAGGACATTTTCT
CCATCTCTAAAGAAACAATTGGTGCAAAATTTCAAAGAGAGAGGATTTTGGAAGGGTCGTCTCAAACTTGCCAGGCCTTG
TCTGTACATCGAATGTCCTCACGTCCGAACTGCCTACGAAGAGATGGGATATCCGATAATCGTCAGCGCTTTCAGACCCG
GAGAATAGATATTTTTTCTTTGACAAAGAAAAGAAAAAGATGCAAAAGTTTTTTGAGAAGAGAGAATTGGTGTGCATTGC
CCTCGTGGACACGAGCCTTCCCTCTCCGGAAAGGAAGGATTTCATGACCAAAAAAATCACAAAGAAAGGATTTCGAGAGG
TCAGAGGCGTTCTTCCAAACGGAGAAACGTTCTACTACATTGCCAAATTCAGTGGGGGAGTTTCCCTTTACGCAACATAC
ATCGACGGGAAGATGGAAGGGCTCTTTCGCGTTCTTCTCGACAAAGAACTTTTGGTGGAGGGACAATTCAAAGAAGGAAA
GCCGCATGGAGTGTTTTATGAATGGTGCGGGAACTCTGTGGCATCCGTTTCCACTTTTGTGGATGGAAAGATCTTGGAAT
GGGCTTGTGACGATGAGGTCTATGTCATTTCAAGGAACGAAAACAAGGGAACCTTGTTCGCTCTTGGAAAACAAGAATCG
GAGAACGGAGGTCTCTTTAGGTTTTCATGGCTCTTTTCTGGGAAAGAAGACGGAGAGCCAAACATAGAAAATATCGGCTC
TTCTCTCCACATCGCTCTACCGAGAGAAAAGTTTCGGCATTTGGAATGTTCAGAGATAGAGTTTTTTTCAGATGAAAGAG
AGGGACGAAAGAGTTCTCCGCAGTGGCACAGTTTGACCACAAATTTCCTTTGGGTAGGAAGACCCACAACTTACGAGGAG
AGATGTCCATAATTTTCTTTTGATAAAGAAAATTCACTAGTCGGAGTCTGTGTCGAGTTCTTCGTCTCTTGACGCCTTGA
GAGGCTTTCTCATTCCTCTGGGGCGCCGGATAATTGCAAGCTCTTCGTCAGGTTCGCGATCGTCATCTGAAACGCCACTG
AACACAAATCCATCCTCTTCTTCTCGATGTGTCTTCTCTTCGAATCTGTCCTTTATTTTGAACTCTTTGAGAAACCCTTC
CGCAATCGCGTTCTTCCTCAACGAAGAACAAAGAAGTTTCTCCTCATCAAGAATTCCGCGAAGACAAAGAGCATCGTCAC
AAAAACTTCCTTCTGTTTTTCGAACACACATCAACGCTTGCTCGAGAGCACGAAATCTCCGTCGAGAGAGTTCCGCTGCT
CCTTTCATCGCCTCTTCTGCTTTGGCAAAACAAGAGTCGCGAAAGATCTTGATCGCCAAACGGACTTTCTCAAATTGGAC
GCTTCCAGGAGTGGCTATGCCCTTGATGTACACAGGAGACGCAAAAGTGGCAGAACACACAATCGCATCTTGGTTTTTTT
GTTTCGCTGACGACGTCAAAGTACAAAACTCCCTTGTGGGCTGTTGCGAGAAGAAGGGCTGTTTCGGTTTGCATCATTTT
TGCGGAGTGGAAGAGATTCTTTTTTGTTCTCCCAAAGAGTCGGTTGAAACTTTCGAAATGCACAAGTTTTTCGAAAAGAG
GGAAATGGTCTCTTTTTCTGTCTCTGAAGGGAACCACAAAATCCCAGAAAAGAAAAACTATGAAAAACCCAAAGTGTCCC
GTAGACACTTTGAAAAAACTACAAAAACTGTCCTTCCAGATGGAAGCTCGGTCCATCTTTTGGTTGAGACGATTATTTTG
GATACAGACGGCACTTTCACCCGCATCAACACGAAAAGTCACCAACACAAGAACAACGAGAAGCACGGAAAATGTGTTCT
TTCTGATGTTTGGGCAAGCGAAAAGAAAAGAATTCGCGTAGAGGGCAACTACAGGAATGGCAAACCTCACGGAAAATTCA
CAAGATATGTTGGTGACATTGTCCAAGAAACTTGTGAATTTTCAAATGGTAGACTCGTTGAGTGTTCCGTATTTCTTCAA
CCCCAAGGCAGACTTTGTCAAACAATATTGTGTAGGGAAAAGAACGGCACAGAACACACCATCACAAAAATAGAGATGAG
GGATGGCTCTTTGTGCATCGAAGAACAAAAATGCCCTTTGGAAGGAGCTCACAGAGAAAGGTTGAGATTCTTTTCTCCTG
AAGGCAAAGTAGAAGAACTAGACGAAGAGACAGAATTTGACAGAATGCGGGAGAGGAACATCTCATCTCGCATGTTGGGT
ACAGAATAAAATATTTTATTGATAGAATAAAATGTACAACTTTTTCGGAAGGAGAGAACTTTTATCTTTCTGTGTCTCTG
AAAAAAACCATAAACTTTTAAAAAAGGAGGACTTTGTTTTGAAACAAAGGATTGTTTCCGATGATGCAGAGCGGGAGAGT
GAAAGACTCCCAAACGGAGAGCTTTTCCGTCTCAAAGGCTCCATTTCCGACAAAGGGACACACATAGACGACTCTACCAT
ATTCACAGAGTATGAGTACAAGAGAGGTGCTCTTCACGGAAAATATCGCGCAAAGAAAGAGATCAGAGACAGAACTTGTG
AAGTGGAAGGAGAGTTTCAAAAAGGGAAGCCTCACGGAACCTTTGTTTTCTACGAAGGGAAAAAGTTCTCAGAGAGTTGT
GTCTTTGTGGACGGAAGAGCTTTCGAGTCTTCTCTAGACACACTCTCAAAAGAAACAACGATATTTTGCAGAAACAAAAA
CAGAGAGGAAGAGTACCACATCACAAGGACAAAAGAATTTGGAATTTCTCCATACGTCATCCGCATCGAAGAAGAAAAAG
TAGTGGGTTTGGACGTCGTCGAAACGAGAAAAAGGGAATTCTCAGAGACTGAGGAAGTCGTCGACGAAACGAGGAAAGCA
AACGCAGACAGCAGGGGCTCTTTCGAAATTTTCTTTTTCTCTCGTGGATACCCAGAGCAATAAAAATTTATGAGAGCTTG
GGTGTGTATTCTCGTATGCACAAGTTTCTTGAGAAGAAGGAGTTGGTCTCTTTTTCCGTTGCTGAAGGGAACCATAAAAT
TCCGAAAAAGAAAGACTTTGAAAATCTTGTGTTTGGTCAAACAACGAAAAACGGAACGGAAAAGTGGACAAGATTACCCA
ACGGACAGCCTGTCTTTCTTTGTTTGGAAGGAGTGAGAAAGTTTCACGGGACTGCCGTTTCCTGGACTATAGAACAAGAG
TACAAAAAAGGACTTGCTCATGGCAAGTACAGAGTTTTTTGTTGCAGAGAAGGAAGTGAAAGGAAACTTGTGGTCACTGG
CGAGTTTGAGAGAGGAGAGCCGTCGGGGACGTTTTGCGAGTTTGAAAGCGTTGAGACTTGGAATAGAGTCGCCTACGTCA
GAGGGAAGGTGTTCGAACGTGACATCGTATTCCAAGGGTACGAAATGAGAGTCGTCAGGAACAAGAAAAAAGGAGAAGAG
ATTTTTGTGGAGACTAAACACCGTATGGAAGGGGTATTTGAGAAGCGCATCAAAGAGGCGTCTCTTTCAAAAAAGACGTG
CCGCATAAGGTTCTTTGGCGAGGAAAAAACAGCAGATGTCACGAGAGAAATGGGAGGTTGTGACATGTTGTCTGTCCGAG
ACGAAGTTTTCTTTCTTGATAATTTTTACGAAGTAAAATTATCCAAAATGTCTTTGAGTTCGTGGGCAAACTCTGTCACG

TCGTCGATGTTATCCCCCCATTCTTCCTTGCAGTTAAAAGTCGCTCCTTTGTATGAAATGGTAACCCCTTCGCATTTGCA
CGCTTCGATGGTCAAGTCTTTTGAAAGTTTCATATTCCCGGATTTTTTGTCCAGAAAAGTTTGAACTTGGTTGAGTGCGT
CGTATGTGTCCTTTATTTTTACTTTGCCAGAGAAAGAGGCGTTTTGAAAGTGCAGGGAAAATTCGACGACGAGGGTTTTT
TTGTGGCTGACTTGCATCATAATAATTTTTATGTAAAATTATTCTAAGCTTGTATCTGATGTTTTGCGAGAAGTTCCTTG
AGCCTTGAAAGCTCTTCGAGCTGCTTGCGTTGCTCTTTTTGTTTCTCTTGGCGAATCTCTGCCCTTCTTTTTAGGGCCTG
TTCTTTGTTCCGTCTGTACCATTCTCTCGCATGTTCTCTCGACTTTTCCCTGTTCCTTTCTCTAAATTCTCTGGAATAAT
CCTTCTGAGTCTTCTTTGCGGCAACAGAAGCCATTTCACTGTTACAATGGACGTTATGATTTAAAAATTCTATTCCAAAA
TAGTTTTGGAAGACATTTAACCAACGAGCCTTTGGAAGTAAATGAGCTCGACCAAAGTTCGCAAACTTCCAAAGACTATT
GTCTCTGATTTTTCTAGTTATACCTTTTTCTATCCAAAGACTTTTGAATTGGACTCTTTCGCTTGTCCTCTCTGCGTTTT
TTCTGGTTCTTCAAAGGGAGATTTAAAGAAACATTACCAGAGCGAAGAGCATCTCTCCGAATTTTTCAAGGCTGTAGAGA
AGCCTCCCGTAACTTCTAATTTTTGGGATTTGCAAGATGGAGAATCTGAGGAAGAACTCGCTCTTAGGACAAGTTTGATG
TATGCTCTTGGTGTACTTCCCCCATATTTGAGGGTGGAAACGAGATGGGTGAATACGGTCATGTTCTCTCATCGAGTCGC
TCCAGAGAGCGAGGAAAGTTTCGCCAAAAGAACATCGCGAGGGGAGACGTACCTTTCGAAACCCATCGAATTTGCCATAA
ATTATGGGAGACAGATGGGTCATAAACTCTTTTTTGTCTATGGGGAGAACAAGTATTCGAGAAAATATTTGAGTTTCCGG
GACTTTGAAACTTTTTGGAGCGTATACGAAACGCTTCCCGACGAAGAGAAGAGGTTCGACGAACTGTTCACAAGCGAATC
TCCGACCAAGGAAATCTTTGACCTTGAGACGACAAGGTACGCAAAAGGGAAGTTAAACGAGAACACCATATTCGAACTCT
TTGTCAACGCGAGAAAAGAGTTTTCCCCAGAAAAGAAGCTAGAATTCTGGGCTTTGAAGAGCTCTGGAAGGGAAGGAGAC
AAATACAAATATTCTCTCCATATTCTGAGCAGCGAGATGTATTTGAACCTTGAATCTATGGGTGGTTTTATCCAAGATTT
TGCAAAGTTTTTACAAACAAGAACAGAGTACGCCATTCTTTACGAAATGATGGACAAGCTCATCTACAACCGAAACAGAG
GAATTCGCTCTCCACTTTCGAGAAAGTGCGGAAGTGAAAGGAAGCTTGTACCTCTCTTTGATGGAGAAGACATTCGAGAA
TTTTTCGCAACAGCTCATCCTTCCAGGTATTTCGGTCCCTTTGGAGAGGAGAAGCAAGAGCAAGAAGAAAAGGAGAGAGA
GGTTCTCGATGAGTGCGACGACTTTGAAGAAGCTTTGATGGATTACGTTGAGAAGAAGCTCGACAACTGCTTTGATGTGT
TTCGTGAGGGAGAAATGTGGAGACTTCAAAGAGTCTCTGGTTGCTGGAACCATTGTCCCATCTGTGACAGAGAACACGAA
AAGGACAACATGTTTGCATTCGTCAAAGGTTCCGGACTCTACCTCGGATGCTATCGTGGCGAGAAAGGAGAGAGGTCGAT
ACTCCTGTGTAGGAAGGAAGGAGCAGAGAAAAACAAATGCGCAGAACGAACCAGCCAAAAACCTGACCTTCCTCCCCTTT
GTTGCGATGAGGTTTATCGTGAACCGCAAGTCCGAGAATTTGTCGAAAGGAAGAGATGTGCGACTTTTCTCGCCAGCGCC
ATGGGGACGGGAAAGACAAAAGCTCTGGCGAGGTACCTCTCTTCCCATCCAAACAAATCTGTTTTGGTCGTGACCTATCG
CAAGTCTTTGGCGAGAGAGCTTGCATTGAAATTTCCTGGTTTCAGCCACTACAAAGAATCTCCAGATTGGACAAACGCAG
AAAAGCTCATCGTCCAAATAGATTCACTTTGGAGAGTCGATACGTCGAGATACGACATCGTTGTATGCGATGAGGCGACA
TACACATTCTCTCGTTTGGCGAAAGGAGTCAAGAATGTTTCCGGTTGTTGGAAGACGATGAAACATCACCTTCAAAAGGC
AAAAGAAGTCATCTTTATGGACAAGAACATGTCTCAGTCGCTTGTGGACGTCATGAAACTTTTGGGGTTGAAATGTCACG
TCGTCAAGAACGAATTCAAAGCTCATACGCAAAGAACGTGCTTTGTTTGCCCAGATTTTGAAGAGTTTAAAAATTCTCTC
ATTCATGACCTTGAAAGAGGAATGAAGCTCTGTTTTGCGAGCAGTTCAAAGAAGAAACTTCAGATTATCTGCAAGGAGGC
AGAATTGCTCGACTACAAAGTTTTGTGGTATACGGGCGACGGAAATAGTGAAGAGGTTTGGCTCGAAAGTTGGAAAAACT
ATGACCTCGTTGCTTACAGTCCTACAATTTCAGCAGGAGTAAGCTATGAAGAAAAACACTTTGACAAGGTATACGGTTAC
TTCAGTAGTCGCTCTTGCCCAGCAGAAGAGTGCGAGCAGATGCTCTTCCGCACCAGAGACATTCGAGCAAACGAGATGGT
CGTTTGCTTTGACAACAGGAGTTCTCCCGCTCCTGTGACTCGAAAAACGGCAGCTTCTCTTCTCGAAAAGAGACTCAAGG
TCATGGACTCTATTCCTTGTATTGGATGGGATAGAAACACAAAAGGAAGACCTTTGAATATGAAGCATCCGTTCACAGCA
CTTTATGTGGATACGGTCGTCCAGGAAAACATCTCGAAACGCGACATTTCTGGAACACTCATCAATCTCTTGAAAGAGCA
GGGAGTCACGATATACTTCGCAGAGAGCAAACTCTCTTCTATAGAAAAGAAAGAGGCCAGACAAATAGCGAGAGAAATAG
CAGAAGAGGTGAAAGAAGAAGAATTTCAAGGCATATCCTCAGCGAGAGAAGTGGAAAGACCAGAGTTCTCGTATCTTTGC
GCTTTGGAAGACAGAACGAGGGAAGAGAATTTCAGCTGTAAAAAGTTTATGCAGGCTCATCTGTTTGGAGTGGAACAAAA
AGACATCACCGTCAAATTTCTAGAACAGTATCATGGTCAGCACAAGCAGTTCAAAAACCAGAGGATGGCGCTGTTCGGAA
CACGAGAAGAGCAATCTCGAAGGCTCAAGGTGTTGGCAAGCAAGAAAAAAGCAGAGCGAGAAGCGATGGACGGAATAGAG
CGTCTCCAAGAGAAGCAAAGCTTTGAAAAAGTCGTCTATGCGAGAAGGCTGCTTTTGAAGCTAGGGTTCTCGGATGTTCG
GGAGAAGAAAAAGATACATTGCGATGAGATGGCGAAAAGGGTCGTATTAGCAAAGGAGATGATACGGAAGAGCAAGAATT
TTAGAGTTTTGTTTGGAGAGATGCCAAAGAGGAAGGAGATGTTTTGGCTCAACGGAATCCTGCGAAGAACGTTTGGATGT
TCTATACAAAGGAATTCAAAAAAGAAGGCATTCAGCTGGGTGTTGTTGCTTGTTTCTCCTTGGAGCTACAACGGAGTTGT
CCCAAACACAAAGGTAAAACTCGACAAGGATGTGAAAATTCCCGAAGTGTCTGGCTTTGAGGTCTGATAATATTTTTAAC
AAAAAATATTCGAGAATTCCAGAAACAAAAGAGAAAAATATGAGGGGGTGGAGAGCGGTTGAATCTTGGAACCCGGTCGT
TTGGAATGAGCTCTCCACCCCCTCATATTTTTCTCTTTTGTTTCCCGTACAGTCGACAAGGGGTCAACTTCATTTCACTT
ATTTGTTACAAGTGGCCTTCCCATGTTTTTTGTTCTATCTTTAGATACTCTCCAAAAAAGAAGTTTCTCTTCTCTTTTGT
TTCTGTCGTCTCCTTGTCTTTGGAAAATTCTCTCTTCCACCCTTTTCCGCCCTTGAACGTGAACCACCAGATTTTGAAGT
GAAGGTTGCATTGCAGCCCTTCGCGTTTGTCTTTGTATCCGAACCATTCCACCTGTTCAACGGAGGGGGTTTCCGTTTCT
TTCCTCATGAGAAGATGGAGAGTTTCTTTCTTTTTGTTTCTGGATATGACACTGCTTTGCAAAGGGCCTAAAGAATATTC
CAAAATCTCTCCGTTCACAAACACGCAAGACGACAAAAGTCTTCCCCCAAAGTTGTACAGACGGAACGTTCCGTGTGCTT
TCCCATTTCGGAACATTCCCTCAACTTCGACTGCGACTCCCTTTCCACAAGAAAGGACAACTTTGTATTCTCCGTGCTTC
TTTTTTCCTTTGTATTCACAGGAAGTCACAACAGAACAACTGAAATCTTTTCCTTCGTCGAAAATTCTTGTACTTCCGAG
CCACCCTTTGCGAACAAAAGTCAAAGAGCCATCTGGAAGCTCGGTGCGCCACACAAGGAAGCCTCTTTTTTCCTTTTCCT
TCTCCACCTCAAAATCTCTCTTTGATACCGTTCTGTGGCTTCCCCCTGCTATCGAAAAATTTAAAACTTCCCTTTTGTCA
AGAAAGTGTTGCATCTTCTCATGGAAGAGTTTTTGGAAAAGAAAGAGCTCGTTTCTTTTTCTGTTTCTTCTTCATCTTTC
CCACAGAAAGAAAAGTTTCTGAGGCAAGTTATAGGGCTCAACAAAGAAAGGACGGAATTCTGGACTGTCCTTCCAGATGG
GACAACGGTCCATCTGGAAAGTGTTTCAGGAAACACTTTCCTTTGCCCATACAAAGACGGAAACCCTCACGGAGATTTCG
AATACAATTGGGAAGGTTTCAAGGAGAGAAGAACGGGTTCGTTCAAAAGAGGAAAGGCGCACGGAAGCTTCTTTGTTTGG
GATAACGAAAGAATCTCTTTGACTGCGACGTTTGTGGATGGAGAGATTTTGGAGATGGAGAGCCGCTTTCGCAAATTTCT
CTTTTCGAGAAACAAAAAGAGAAGAACCCTTCACATTCTTTTTTGGGAGAGAAATGGAGACAGGCTCGTCATCGAAAAGA
AGCTGATGATGAACGCATCCCAGTCAAACAGCGTTTTTTCTTGCCCGTTTGTCCCTTTTGGAAAAGAGCCGCTCTTTACG
TTTGCGAAGCAAACCCTTTTGGAAACAGAGTATTTCCCTGATAGACCCAGCGAAGAGAAAGTGTCAGAAAGTTGGGAGAA
CATGTTCTCCGTAGATTGAATGAAGCACAGACAGTAAATATTTTTACAAATAAAAATATGGAAGAATTTCTCCACAAACG
AGAGTTTGTCGCTTTCTATGTCGTTTCGAGACAAACTCCAAAGAAACGGAATTTCACTGTCTTTGACGGTGAATGGATGA
AACTCCCAAACTCTCTGCCGGTTTCAAAAAGACGACATGACGATTCTTTGTGAATACAAGGGAAAACTCCCCCACGGAAA

TTTTATTTGGAAGTGCGGGACGATGACAAAAACAGGTTCGTTCATGGGTTGTTTTTGAGTGCGCCAAAGTGTTTTCTTTT
GGAAATCTCGTCGAGAGACAAGGGAAATATTTGTGCTGTCAAGGAACAAGAAGAAAAAGGAAGAGTATTCACTCTCAAAG
TTTCAAAAAAACGGAACGCTTTCGGTCCATTCGCGGGTCACAAAGGGAATTTCTTGCGGTCCCTATAAACTCCCGGAATG
CATGTGCGTCGTTGGAAAGTCAAGGGTATGCGCACATTCCTTACGGAGAACGCACAAAAGGAGGAAGTAGACGGGGAAGA
ATTCACGAAACTGCGAGTTTCGAAGGTTTCGTTGTCGCGACGAATAGAAAAATATTTTATTTCAAATATTTTATGAAGAA
CACATGGAAAGATTTCTGGAAAAGAGAGAAACCGCGTCTTGGTTTCTGAACGAACCTGAACTCGAACCAGAAAAAGAGAG
ATTTGAACAAGAGCGGATTTTTCAGGAACAACTTTTACATCGCTCTCGAACTGCGGTTTGGAGAGTTCTTCAGAACGGTG
AAAGGACTCATCGCTCCCTCACGACAGAGGACTCCAATTTTATTATTGTCGAAAGTTGCAGATGCAAGGACGGAGTTCCT
CACGGAAAGATGGTGGAATACACCACAAACAAAAGAACTGGTAAAACCTGTCTTTCGAGAGAGGCCGAGTTTGTGGATGG
CAAGCTCCATGGAGAGTTGTGGGTATGGAGAAGCGTCGGTCACCTCCAAACTAGGGCAACATTCACAGAAGGGAAGCTCA
TCGAAAACAATGGTCACATCTTCACTTGTGTCTTTTCGAGAGGAAGAGGCAAGGCAAGTTTTTTATACAAGAAGCTGACG
CTGCATTTCTTGGACAGAAAAGGAGAGGAATATTCTTCTGCAAAAACTTTTGGAACGTATTCATCAGAGGATTTTTTGAC
TTGGGGAGGCGGAGATTGCACCCCCTTCTGTCCTTTAGAGGCAAAGTTTACGATGAGAGACGTCGAAATGGTCGTAGAAA
GACATGAGGGACAAGAGAGTGCGCGCTTCGACAGACGTTTCGAAGAGAGGACGGTCGTCAATAGATTGACGTTCTATTGA
AGTTTTTTCCATCCAAAGAAAAATGTCAGAATTTTACCTCGTGTCGAGTTGCGAAGAAAGGCCTTGGTCTCCTCCCAACC
TTTACGCTCTTCAGAGCACAAAGGAGCTTCTCAGCTTTTTGGAGGAATTTTTCGGAAGGGGCGAGGAAATCTTTGTCGTT
CGGATAACAAAGGACGGAAAGTGGAACAGCGTGAAAGAATACGACGACTTTGTCGATGCCGTGGAAGAAAAGGACCTCTT
TGGAGAAATATATGAAAAAGATGGGATACACAGCGTCAAAAAGGCGAGAAGTCTCTTTTGTGCCATGAAGAAGAAGCTCA
AAACTCTCCCTGACGTCAAAGGCATTGAAGAGGAGAAACTCTTTGCTTGTGATGAGTGCCAGGTCGTCACAGAAAAACTG
ACGTCTTGTGAAGACGATGAGTGCGTCTGTTTGCACTCTTGTGGATGCAACAAGCTGAGAGAAATCTCTTTTCTCTAAAT
ATATTTTTTGTTTCAAAAAATATACACAAGTATGGACACAAGAAGCTCACTGACGAACAACTCGCTGACGCTCTCCAAA
GGATTGTCCTCGCAAAGAGGAAAACAGAAAGGGATATTTCTGGAGAAAGAGGGGACAGAAGCAAACTTTACAGATTTCTT
GGGGAACTTGAAGGAGAAAGGGCTCTTATCCTCGCAGAGATGAGAAGGCGACAACCGCAGCAATACATGACCCCGAGGTA
AAAAGTTTATGGGAAAGCGAAGCAATAAAAATTCAAAAGAAGTTTCTTCTTTTGAAGATGGAAGTTCATATCTGCTCCTT
TCCTCAAGAGGTGCTTCTCGAGATTTTTGGCTTTTGCGGTGAGAGGACTTCTTTCGAACTCGGAAAAGTTTGTAAACTCT
TTCGTGGTCTTTCTGTGGACAAACAACTTGCGAAGCTTTTCAAGCATGAAAGGAACAGAAGGAATTTGGACGAAAGGCTC
TGGTTCTTTGCTATGATGAAAAGGGTCAAAGTGTACGGACAGTTCCAGAGATTCAGTAACACAGAAACCACAGAGTACCT
CTGCGCTGGAAATAAAATCTCTGACCTCAGAAGGAGGATGGCAGAGAAGCACATGTCTCCTCCGCACTATCTCGTCGTCC
AGTATTCTGAGGGAGAAAAGCGTGTGACTCCCCCAGATTGCGAACTTCTCGACCCAAAGAAAATATATTTTGGAAAATCC
ATAAAAGAACCTTGCTAAGTTTTTGTATTGAAAAACTTTCACAATGCAGTCTCTGATCCTCGAAGCGATGAGACATTCCC
TCCGCGTTTCCTCGAGACCGAGGAAGAACATTGGGAGACTTCGGAAATGTCTCGAATTAATGAGCGCGATAGAGGCAAAA
ATTCCAAAGGCAGAACAAAGGTACGCTTTCTGTCGTTACAACGAAACTCTCTATGAAGAGACTCATGACAATGTGGCGAC
AGAACTTAAAAGGATGGTCGGCTTTGCGCCCGACTGGAGCTTCACGAGTCAAATGGGAATTTTTCACGAAGGGTACGAAG
AAATAATAGGCGAGTTATCAGAAACAAACGCCATCTGGTGGGAAACGGAAGAGGCTTCTCACATCACGTTCCTCCCTCCT
CGTCCTCTCTTGGAAGGAGATTTGAAAGTCAGACTTGTTTGTGGCGAGCAAGGGTACACCATCGTTGACGCAGATGGTGA
TGAAACCCCTTGCGACAAGGAAGATGCCGTTCTTTGCCTTTCTGCTGCTTGTGAAAAAAGAAAGGGAGGATATCCTATTT
TGAGTGCCTGTCTCGAGAGGGTCGAATCGATGCTTTGAAAACTTATATTTGTTGTCCAAATATACGGAAAGATGCATCTC
GGAAAAATCTCTCTTGTCGCTGAGAAGACGCCAGACGGGAAAATGTGGGAGTTCACTGTTCTGTTCTTTGAATCGGACAG
GGAGGATGTCGTGTGTTTTGTGGATGTGGGAAAGGCGAAAATTTCAGAAATGTTTGTGACACCAGGAAGCGTCCCTGTCG
GATATTTGAGGATAGGCATCCTCGAATTTCAGGAAGAATGCAAGAGGCATCTGCGGATCTCAATCATCGAGAGCCTTGCA
AGGAGAAAAAAGATTATGAACACCCTCTTCCTCTGTGCTGTTGAAGACCGAGACGAGGAAAGAAAGTCTCTCTTCAAATC
TCTGGCAAACAAACAGAACAAAAAGGTGCAAACTCTCTTGTCCCGCACAGGAGACAAACTTTTCGTCTCCCACAAGCAAA
AGTTTGAGACGGACGATGGAGAACTTTTTTGGGAATCTTAGGACAAAAGAATAAAATATTTCGCATAAAATATTTTGATG
CAAGCTTCTCTTGATGTTCACAGGTTCTTCGTTAGCGGGATTTTTTAGAATGTCAACTGTTTGACGTAAAAGTACGACCC
TGTGCCGAAATTTTGCAAAGTGACGGTGTTTCCTGTCACTCGATGGCGGAGAGTCATCGTTTGGCCAGAAGTCGCCTTTA
GTATGCCGCTCCCTCCACACGTCACCCACACAGACGTAGAGAGTGCGTTTTCAAAATTGTACTTGACGACAGCGTTCGTC
GCATCATAAATCTCCATCGCCGACCCTGTGGCGGAGTTTGTGCTTCTTTGGAGGTAGTAGATGATCTCGAAATCTGCTGT
GGCGGGAAATGTGAACGTAGTCCCACCGCTGAGGGGAGCGGAACTATAACCTGCGTCTGTCGTCCATGACAAAACATTCG
TTCCGACGTTTTGCGTTCCAGAAGAAGCTGCAAGACTGAATTTCCAATCTGGAAGGGCTGGCATCTGAGTCGTCGAGAGT
TTTCCAGAAACCAGCCCAGCGTAACCGTTCGTTGCATTCTTATTCGAGAGACTCTCTTTCGACGCCATGTTCGTGTCGAT
GGTAGAGACGTTGTCGTTTGTTCCTACAGCCTGTCGCATGACAGAATCTCCTCCAATAACAAGAAGTTTGTCTGTACCGG
TACCTGTGACAGACAAGTACCCAACAATTCCAACATTTGTCTGTGTTGGTGTTGGACAAGCACTGAAAGAAAATTTGGAA
TTCGAGTCTATCCCAAGGTATCCGGAAGGTTGGTTTTTTTCCGAAAGATTCTGTTTGGTTGTTTGAAGAGATGCGATGTT
TGTTGTGTTTGTGGATACCTGACCCTGCAATGTAGTGATATTCGAAGTATTGGTAGACGTCTGCCCTTGCAACGTAGTGA
TGTTTGAAGTGTTTGTTGCGATGTTTGTCGTGTTTGTGGCGACTTGACTCTGTAGAGTTCCAATATCCAATGTGTTTGCG
GCAACTTCCCCTTGCAAAGTTGTGACGTTCGTTTGCAGCGTGGAAATGTTTGTTGTGTTTGTCGCTATGTTTGTTGTGTT
TGTGGCGACTTGACCCTGCAATGTGGTGACGTTAGTTTGCAGCGTTCCGATATCCAAAGTGTTTGTGGCGACTTGTCCCT
GCAATGTGGTGACGTTCGTTTGTAGCGCCCCAATGTCCAAGGTGTTTGTAGCAACATCCGCCTGCAAAGTTCCAATATCC
AAAGTGTTGGTCGCCACCTGAGTTTGAAGCGTGCCGATGTTTGTTGTGTTTGTGGCGACTTGTCCCTGCAATGTGGTGAC
GTTCGTTTGCAGGCCAGAAATATCCAAAGTATTTGTATTGACCTGAGTTTGTATTGTCGAGACTTGGAGTTGCAAAGCAG
AGATGTCTGTCGTGTTTGCGGCGACCTCTCCTTCCACAGTCGTAAGGTCGACATTGAGAGCTGAGATGTCGTTGGTGTTT
GTCGTCACCTGTGTCTGGAGTCCTGTGACTGTGGTGTTTAGAGTGGTTACTTGCCCTTGCAGATTCGTAACATCGGTACC
CAGAGAAGAGACCTGTGTATTGAGAGTTCCGATGCTCGCTGTGTTTGCTGCAACTCCGCTCTGAAGAGAAGAAACGTCTC
CTTGCAAGACGCCGATGTTGACGGTGAGCCCTGCCGTTTCGACTTCTAAAGCCGCAATATCGGCTGTGTTTTGGGCCACT
TGTATCGCAAGAGGTCCGACATTTCCTCCAGCGATGACCCACTGCTCTCCTGTCGACATATAGAGATTCGAGTCTGTTGT
GAGCAAAATAAGAGTTCCTCTAGGCAAAACTGCCGGAAGAACAGAAACGGATGCGATGAGGGTTATGTCCTTGAAAAAAT
TTTTCTCCCTCTGCATCACGTGTTACAATGAACAAAATATTTAGAACCAGAAAATATTTTAGCTTATTTCTCCTTGGAAC
ATGCCGTAAAGTAATGGATATCGCAAGCGTTGTTTTTGACAAGGACAAACTTAGAGAATTTTTAGGGACAGAGAGACTTT
GGATTGACAAAGAGGGTCGCATCCGAGACGACTCTGGGAACTGCTTTGACGTCTGTGTTTGCATTGTGAGACAAGACATT
TCGTTGGATTGTGTAAGGCTTCTCCTCAAGCACAAAATAAAATTTTTACATAGTTGCGAAGGGTGCTCTCTTTCACAAGA
AGGATAATCCCTGTCCTTCTCAAACTTTTGGATGAAAAGAAATGAACAGCCACAAAGAATTCTTTGTTATGGAGCGCCT

TCCAAACGAGATAGTGTTGCACATTTTATCTTTTTGCGAAGCAAAGGATGTTTGCAGCTTCTCAAGAACAACAAAAGAAT
GTTTGTCGTTGGCAGAGGACGAAAGACTGTGGAAACAGCTCTGTGAGAAGAAGAAATTTCCAAAGAGAAAAAATGCGATG
GCTGGAGAGAATGGTTCGTCAGAATGATAAAAAGGAAGCCCCTCATGCTATCGGTGTCTGCCAATTCACAAAAGACAGAG
AGGTGCATGAGACTCTTCTTTCGCACGGGCAAGTTCTCGAACTTTTCATTCAACGATAAAATATTTTTGCAAAAATATTT
AAAACTCTTCGTCTCTCTCTTTGTCGTCATCTTCCAAGTTTTCTTGTTCTTTGAAGCTGAGACCTCCGAATTTCTTCCCT
CCTGTGGGCTGGATTTTTATGGCGCGAGAGAACTGCTTTCTGAACTTGAGAATGGTCATCGTCTTTCTCACCGCGTAGCT
CGGGAAGTTTTCGTCGTGCCATTCCTTGTAGTGGTTGAAAATCTCGCTCACAGTCAGGAAAGATTTCTCTTCGTCTTCTT
CCTTCTTGTCGATGACGATGCATTCCTTGATGTACTTTCGGATCGTGTCGTTTCTCGCCCTCATCTTTGCGGTGGCGGCC
TCGACTTCTTCTGGTTCGCAGAGACCCTCTTCTTTGTACTTTCTGAAATCTTCCAAACACAGCCACAAAAGAGCGTGGGC
AAGACCCGGAATTTTCTCTTCAAAGTGTTCGTCCGCTTTGAACATCTTTGCTTGTCTGCGAAGTTCTGGGTCTTCTATCC
AGTTTTCGTCCTGTTCAGACAGGAATGTCGCCTCGAACGGAATGAGTCGAATTCTGTTCCATGTTGCTTGGTCAGAGCCA
GGAACTCCCGGAGGTTTATTGGCCATCATGAAGATCGTGAACATTGGCTTGATGTCCCTTCCCTTTTCAAAGAGGTTCCT
CACAAAGAACGAGTCGTTACCAGAGAGTTCTTTCAAAATTCCTGGGTTAAACTTCTCGTTCTTGTGAACTTCTTGGATCA
CACCATATCTGGTTCCCGGCGCTCTTGCGAGTTCGGGACGGGCAGAACTCGGGTTTCCAGCCCTTCCAGCAAGGCACATT
TCTCGAGGAAACTTTATCAGGTACTGTCCAAAGATATATTCGAGAAGAGAAAAGAACACAGACTTTCCGTTGTTTCCCTT
GCCTGTGCAGATGTAGATTCTTTTGTTCCTATTTCCTCCTTGCATGCAAGAAGACACCATTCGAAGAGCACATCTTCTGA
TGCGAGGATTCGGGAACAACTTTTTGAGAAATTCGCGACATTCGATGACTGACCTGTCGTCCTCTGACAGAGATTTATAG
CTGATTCCCGTACTCATCGAGATAAAGTCGTCAGGACTTCCATCTCGGAAGATTCCAAGTTTCAAGTCACAAACTCCGTC
TTCCATTCCCAAAAGATCCCTGTTTTCGTCCAACTTTGAGAGGAATTGTTCGTTCAAAAACAGCCTTTTGCACATCGTCA
TCACTCCTCGAGAAAATCCTTCCATCTGGAGTTTGGACTGGATGTCCACGCATTTTTTAATCGTCAGTTGTGCGTTCGGG
TCCTGAGACTGCCCCGACACCCCAGACAATCTGTTGATCTCGAGACGGAATTCTTCTGGGAGCTCAAAGCTGATAATTCG
CATCAACTCGTTTGCATCGTCCAGTTTGTACCAACGATGACCTCGGAATTCGTACCAAATGTTTGCCGTCGAATTGGCAC
AAATGAACCTATCCGAATACTTGACATGGATCAGACTTGCGATGTTTGCATGCGTTGGTTTCGATGCCAACGACGCCTCT
TGCAAAGCCTTTGAACTTTTTTCTTGCTTCCACTCGCCATATCTTTTCGGGTTGTCGTTCTTTGCCATCTGATAGAGACT
TCCGATGCTTTTACCCTTCATCTCCATCGTATCCCAAAGGTATTCGCATTTCTTCTCGTCGAAATTTGTCGCTCTCGAGC
TGAAATCTATCCAAAGTTCGAGGGCTTCTGGAAGACCGTTCCCGATATTAAACAAAGTCCAGCCTACATTCATCCAGTCG
TCATAGTTTTCCGCACGAGAGTCGTCGAGCATCTCCAAAACTCTCGAATTTCGAACTTCTGCGAGATCTTGCATGATCTG
TTCCACAGTTTTTGTGAGACGGGCTGGTCTCTTTCGACGCGTCATCTTCTTTTCCGTTGGGATGATACCTCGAACGATGG
GGGTTGGCTCTCTTCTTCTCTGGATGGACATTATGTACGGAAGGTCTTTGGGAATTTCAAGATCCATTTCATCCGCTTCC
TCTCCGAGCAAAGCGTCCACATCAATCTCTTCGAGCTCGTCACTGTACACTTGTGTGATGGTGTACGGCTCCAAAAAGTC
TCCGATATCTCCTGGCTTTGTTGAGCCATACATAAGCCATGCTTTTCCTTTGAGCGAGTCGATGCATTTTTCTTCAGGTT
CGAGCATTTCGAGGTCCTTGAAAGCTCCAGTTTCAACGAGTTCTTTCACCACCTTTTGTCTCATAAACACATTCTGGACG
TTTGTGGCCACAATAAAAAAAGGAAAGAAAAGATGGAATCCATCCTTCACTTGTTCCCCAGAAAGTCTCGGCCCTGACCT
CTCAAGGACGATGCATGACAACATTCTCTCTGTCTGGTCAAATTCAGGGACGATAACGCGGATGACGTTTTGATACGCAC
GGATCACAGCCATGACATGCTCTTTTGTGTATTTTCTCTTTGGCATCTTTTTGCCAGAGTCCTCGAAGCGAAAGTCAACG
TCCACGTAGAGAGGATGGTACTTGGATGGCATCTCTGTGACGCAAAGAGTGACTCCGTTCTCGACAGCGTTGCTGTAAAC
CTCAAAAAACGCCTTCAAATTCTTTACTCCTTCAATGGCATACCTTCCAGAAGGAGAAAAGGAAGTGTGTGTGCAAGGAA
TTCCCTTGGCAACACGAAAAGAGTCCAAAAACGCAAAAATATCCCTGGTCTGTCTCTTCGCCGCCATTTAATTTTGTCTC
GGAACAAAAACTCTCAAAAGCGGTTTGCGAAATCTCAACCTTTCGGGTTTTTTCTGAATATTTCTTTTCAGTTCAAAAAA
AGAAATGTGTAAAACTTTTTTGTTTGCTTCCCTTCTTCCCGCAGAAATTTTGTGCAACATCGTCGCGTTCTCTGACAACA
CAAACTGCCTGTCCGTCAACAAACTCTTTCATCACGAGTTCTCTAGAAAGATGAAGACGAGGAACGAAAAGAGAGGACTC
TCCCTCCTCGAAAGGGGGTCATTCTACGGAATCGTGCTTGCAAAAAATGCAGAGAAGGGCATCCTTTCCGCCCACGCTGC
GCACTCAGGAAACAAACGCCTCCTCAAAAAGGTGATGATGTACGACAAAAAGAATTGCTACGCCGGAAGCCAAGAGAACG
CACTCTACTGCGCCTCTTTTTCTGGGAACAGAAAGCTTGCACTCTGGGTGAAGAAGAGGTTCCGGGCAAACTGTGTGTCT
GGATTTCATGGCGCGCTTGAAGGAGGCCACACAAAAGATGCTCTCCGCTGGTGGAAAAAGATGGAGAAAAAAGTCGGAGA
AAAAGAGAAATCCGGATTTCTTGGGCATGCGATCGTTCGAGCTCTCAAGTCGGACAACAAAGAGGCAGTCCCTGTTCTCC
TCAAGATGGGAGGAGTCCCCACACCAGACTCTTTCGACATTTGTGCAAAGAGCAGAGACCTTCAAACTTTCAAACTTCTC
CTTGGAGCGGACAAGATGCAACGGAGAGAAAGAGCTCTCCTCGCAGCCATCAAAACCAAAGACCAAGAATTTGTCTTTGC
GTGCTTTCGCGCAGGCATTCAAACAGAAAACATTCACATAGAGTTTGCGCAAAAGACAGACAGAATGATGTCTCTCCTCA
TCTCATAATTTTTGTAAAAAATTATATCAGCGATAACGTTGGATGGCGTCGGCCACAGTCCCGAAAAAAACCGTCTCTTC
TTTTTCGAACCAGAAGAGCTCTTGCGATACCAAAGGAACATCTCGCGGTCCCACTTTGTTCTTTGAACCCCAGAAGCGCG
ATTCCGCTTCTGACGCAAAAGACTTGAGATAACACTCTTGACAAAACCAGTCACCACAAAGGCCTCTGACACCTTTTTGG
TTATCACAAGGTTCTGGTGTCATTCCAGAAGAACTCGAAATTCTGCAAACCCCTCCTTCACATTCCATCTCAAAACAAGC
TCCCTCCATTAAAATATTTTGTTTGAAATATTTTACTAAAATTCTTTGGTCATTGACACCATAAACTTCAAAAAGAGAAA
TCAGACCGTGTTCAATCTCTCTACGAAAATTTCCTTGAGATGAGCCAGTCAGAGAGTTCCTTGTAATTTTTAGAGACTTG
CGGTCTCATTTCTTTTTCGACGAGAGAGTTGATGTTTTGAGCGGTCACGTTGTACTTCATCAGAGTGAACACTGCCTCTT
TGACCTTGTGCTCAAGGAGAAATTTCCTTGTGACATTGAGAGCGATGCCGAGCTCAAACATTTTGTTACTCTCTTTTTGT
GGGATATCTTTCTCATAGTTTTCCCTCCTGTGGCGGAGAGCTTCTCGAATCTTTTGGACGTTTTCTGCCGCTTTCAAAAA
CTCTTCTGACATTTCAATGCTAAATATTATCCAGAAATATTTAGAAAATTTCGAATCTCTTCTCTCCAGTCCGTCCGCTC
CAACCAAACGTCCTCTTGCAACAAACGTAAGAACGTGTAGCCTCTGTCTTCTGCGAGTTGTTGCTTGAGTTTGTCCCTTT
TTTGTATCTCTTCTAGCAAAGTCCATCCTTTGACTTGGCGAAAATGTTGTGGTCCATCGAGCTCAACGATTTTCTTTCCA
ACGACAAAGTCGTAGCGAAAATGTTTGCCCGTCTCTGGATTTCTGCACCAGTCGAACCTCGCCTCTGGAACTATTTCCTC
AGTGAGCGAAAGGCTTTCGAGATGCTTTTTGAGTTTGCCTTGTGTTTTGAAGTGACAACCAGGGCACCAACTGTTTCTCC
AGAATATTGAACTAGGAGAACACCAGAACTCTCCGTGTTCCTCGCAGTCAAAGAGAAATTTGGAGCACGATGCTGTATAC
ACTTGACGAGGAGTTTTTCTGTTTCTCTCGGACCAAGCGTCCGCTCTTGGATGGGAGGCGAACGACCTTTCAAAACACCA
TTCGCACTCAGAGTTTTTGCACATCGGAGGAGAAGGGGAAGCGCATATGAGACAACAACCTCCCTTTTTTCTTTGTGTCA
TAGTACACAGTTTCGTTGTGAATTCGTGCTTGCAGACATTGCATTCGAAAAGATATTCTTTGTTGCTATGAGGTCTCACT
TCACGAGGCTTTTTCTCGTTCTTGAAGCTCCAAATTTCAGAAGCGGGGTTGGACGCAAAAGAAAGCTTTACGCACCAAGA
ACATTTCTTTTCTTCTTTGCAAAGCTGTCTTCCACCACAGAAAGAACACCATATCCCCCTTTGGATGATGTGCGGAGAGA
GCGAAAAAGTGTGCCCGCAGTCCCCACAGTCAAAGAGGAAATATTTATGGGTGGAATTGCGAACGTCTCTCGGAGACACC
TCGTTCTCTGAACTCCAAAATTCAGCCATTTTGTGGCTGGCGAAACTGGCTTTGTGGCAAAAATCACAGTCCTCTCTTTG

GCACATTTTTCTTCCCTTGCAGTATGGACACCAGTTTCCTCCTCTTTTGTCTGTCATCATCATCGGTGTCGTTTGAAAAC
TGTGCAAACATTTCTTACAGTCGAACCAAAACTTTCTTTCGCAACCTTTCCTTATCATTCTCGGGTCCTCTACATTTCTT
TTGCTCCAATACTTTGCCTTTTTGTGGGATGCGATGGAGTGCTCAAAGCAAGAAATGCATTCTCTGTCTTCACACAGCTT
GCTGCGACCCAACGAAGAACAATAAGGGCAAGACGCGTTTTTTGTCTTTGATAAATCCGAGAGTTTATGGGGGAACTCGT
GAGAGCACTTTTCACAAAAGAACAAATAGGACTTGCCCGTGGATTTCAAAACCTTTCTGGGACTTGTGTCGTTCTTTTCT
GACCACATCATCGCTTTGGGATGGGACGCAAAACTTTTGTTGAAACAAATTTCACACTCTTCATCACAGAGTTTTCCACC
TCTCAACGAACAAGTCATCCTTTTTGAATTTTTTGGAGCTCCAAAAAATTCGTCGATATCTTCCCTTTCAAAGAGACATT
CAGGGAAGAGAGTCTTCCTTTAAATGCCGCCAAGGAAAAAAGAAGATGGCCAAAAGACGCTCAACATCAGACCTTGGGAC
CTTTTGAATTCTTATCCGTCGGCCAGCTACATCGCCTGCGGCCCTCCGGGCAGCGGAAAGTCCAATTTTGTGCGTAGTTT
TTGTTATTACATGCGCCACAAATATCCCGTTGCCAAAATTGTCTCGGGTACGGAGACAGAGAATCAGACATTCCACGAAA
ATTTTCCTCCAGTCTACATCAGCACCAAATACGAGCAGCATGCGGAGCTCCAATACGTCGCAAGACAGAGAAAGGCAAAG
ACAGACAAAAAATGTAAAAACTCATCCGCTATTCACGTTGTGGACGATTGCGCTGATGACCCTAAAACTTTGCGTTCTCC
TCTCATGACCTCATTCTTCAAAAATGGCTCGAGGCATTGGGACGGAATTTTTATGCTTCTAACTCAAGCCGGGTTGGATG
CTCCTCCAGGGGTAAGAAAGTGCGCTTCTTATTACGTGCTTTTCTACGAGGCCTCTCTTGTCGAACGCGAAAAACTCTGG
CGAAACTTTGGCTCGAAACTCGGAACTTTCAAAGAGTTTTGCGACATCATGGACCAAGTGTGCGTCGACCACGCCTGTAT
GGTCATCGATAACAGAACGCAAAGCAAAAACATTGAGGACATGCTCTTCTGGTATCGCGCGCCTCATCCCATTCCAAAGT
TCAAGTTTGGTTGCAAACAGTACAGAAAGTGGAGCAAAGAAAGGTACAACAAAAATTATGAACCCCCTCTCATCTAAATA
AAATATTCAAGATAATATTTTTTATGGAGAGACTCGAGAAATTCCCAAAAAAGTGGAAAGGCGTCGTTCACTGCACAAAG
CATTTCGGTCACTCGTCTTACCACAAAACAGAGGACGTCCACTCTTGCTCTCCCTTTGTGAAAGAGAGTCAGTGGAGAGG
AGTTGTCTCTCTTGTTTCTTCGAAAAAAGAGTTTGCCCTCGTTATCCCCCACAAACTCTTTGAGTTCAGCTCTGAACTTT
TTGTGCAACTTCGAAAGAGAGGGAACTGGTTCGTCTTTTCGTACCCCTGGTTGTCCGGTGGTTGTTCGACGTGCGATGGA
GAAACAGAAGCTTCGTTCTGTATCAAAGAATATGGTGGAATTCATGACCTTTTGTGGAACCATTTCACAGACAGAGAAAG
GGAAGATGGGCTCGGTCTTTTCCTTTGGTTAGACGAGGAATGCAGATATCCAGATTCTTTGCAAGAGTTTCGAGAGAAAT
TCCCAAAGAAGAACATTTCGTGGATATTCGCATAGATAAAATATTTTATATAAAATATTTAGAATTTATCCTTTGAGCCG
CAGAAGCAACAGCAAAGATGACTCTCGCTCACCATGCGAATTTGTACTGAATCCTCGTCTTCCAATATTCTCCAAAAGTT
TTTCTGTGTGAGATATGGGGTTCCTGGAGCCTTTGACTTTCTATCGAGCAATTTGGCTTTCTTGGCAAAGTAGAGAACGT
CTTGCGGAAGGATGTGGTTGTTGTAAATTTGAGGGAATACAGTTTTCTCGAGATTCGCAAGGTTCGCGTCCTCTGTCAGA
ACGAGAGGAAGTTTCTTTATTCCGCCCAAGAGAATCCACACAAGCTTTCCCTCCGCAACGACACCAAACTGTGCCATCGA
AAGGGAATCTTTCAGAGAAGCGATGCTCTCTGAGCCATCTTCTTCCCACCTCCGGAAAATCTTGGAGTTCTTCTTCCAAA
CAAAATCCTTTCGCATTTTCTTGAATTCCTTTGACATACTTTTTCCCTCCAGTTCCCTTCTTCCAATCCTTTTTCGTTTC
GACGCCTCTGCGATTCACAGATTTTTCTGTGCCGTGTTTTTCTCCGTTCGACCAAGAAATTTTCTTGACAAGTCTTCCTT
TTCTGTCCCAGAAAAATTCAGGGCCGTGTTTTTGTCCTTGAAAGTACCAAAAACACGAACGCAAACTTCCGTCCCTTCTT
TTGACCAAAGAAGTTCCATGCATTTTGCCGTTCTTCCATTCTACAGAACAGCGAGGGGTGCCAGATTGTAAAAAATAGCA
TGTTTGTGGACCATCTTGTTCTCCAAATTTATACTCTGCCTCATAGTCGAGTTTTTTGTTCTTCCAAAGGCGATGTTTTC
CATGCCTTTCTCCTTCATAAAATTCGAGCTCATACCTTATGATGCCGCGGAAAGATTCGCCTGTTTCTTTCCCGTGTCTT
TTTCCGTTTGGGAGGACATACCAAACGGCGTCCGAGTGCCCTTCTTTGCGACAAAAAGAAAGTTTCAACTCTTCGTCTTG
GCAAAACGTTCCGCTCGCAAGCCAGAAGCTGCAAAGAAGTTTCGGATCTCTGAAAAAATATTCCATCGTTAACAATATTT
TTCTAAAACTTGAGTTATTGTGGGTCTATCTTTCGGATAGTATCTCAAAAGTTCGGAAAGACACTCTCCGAGTTTCTTGG
GCATCCCTTGTTGCTTATGCGGAATGTATTTGTGTCTGATGTGTTGGCAAATCAGAAGCGCTTTGTCAAAGCACCAAAGA
GGCTTCCCAGACCAAAGCTCTGACAGAACGCATCCGAGACTCCAAATGTCTATTCCTTTCCCGAAAGGTCCCAAAGGATA
CACAAGAAGCTCTGGTGCGCGATAGTGAAGCGTCACCACTTCATTGTCCACAAAAATCTCTTCCTCTCCGCAGCAGAGTG
AAAGACCAAAGTCGCACAATTTTGCTGTTTTCCCATCGTACAAAATGTTTGATGGCTTGAGATCGGCATGAATTATTCCT
CTTTTATGAAGAAATTCGAGACCTTTGCAAACATCCACAAAAACTTTCTGGATTTCTTCCGCGTCGAGATGCTTTCTCGA
AAGGTCAAACGGAAGATGTTCGAGGACGAGAGTCTGTGAAAGATTTTGCCTCTCGAACCTCAAAAGTTTACAGATGTTTT
CATGACCTTCTCCAAAGAGGTCGTAAATTTTCTTTTCTGGATGAGCTTCAGAGTCAAAGGATTTGCAAACGTATGTTTTG
TACGTCTCTCCTTTTTCTCTGTCTGCGGTTATTGTTGCTTGCAAAGGATGCGATGATTTCTGGATGAGTTCCATCTCAAA
ATATTTTATGGACCATAAAATATCTCTGAAAGAAATACATTTTCGCAAAATGAACGAACCGGCCGACAGTCAGAACAGCA
GAAAACAATGTCTGCTCCTAAAAAGCACTTTTCTTTTCTTTTGGAAACTTTCATGCACGTTTATACCATACCCCTTCCTG
GTGCAGGATTCATTATCGGATGGACCGAATCACAAGACAAGAAGAACCGTAACAAGAGGTGGTACGTCGCGCTTCCAAAA
GTGATGCTCAAGACCATCTTTTGGCCTTTCAACGTCTCCAAAAATATTTAGGGTTTTGCTGATAAAAAACTAAAGCAATC
TTGCGGGATGAACATGCAACAAAACTTTCTCAAAGAGATTCCTCCCTTTGTTTTGAAGGCTGTGACGTCTGGAAGACCGA
TGAGGAAAGATGTCGAAACAGCAACGAAAGGAAATTACTTCATGAAGCAGCACAAAGGGCTGGGATTGAACATCGAAGCA
TCATCGACAACGCGGAGCTTCACAAAAACCACAGCGAAATAATTGTGGTATCTGACAGCCGCTGTTGTTCTGATCGCGAC
CTCAAAGAGATTCGTCTCTCTTGGACTCCCCATATTCGGGTTGAGGTGAACAATGGAAGAGATTGGGAAAAAGGGGGCTC
TGACATCAAAGATAGAAGGCGTTCATTGACTCTCGCAGCACAAGGGAAAATTCTAAATATTTCAAAAGAAATATTTTCAA
ACAAATATCTTCTTCTCCTTCCAAAGCACAGAGTAGGACGGAAGTTTCGACCAAAGGGACTTTTCGTGGAAGCTGCAATC
GTACTTTGTGCGGACAAGTTCCTTGCCTTCTTTGTTGAAAATTCCTTCTATCTTCTTTCCTGGTCGCGAGTACCTATGAA
AGAGAAACACTTGCTCTCCCAAATCTCCAGGTTGGAACTGTTCGTTCTCTTTCCTTCCAAGCCAAAGATTGAGTCCGTTG
CCGAATGCGATGGGGCCTGTCGTGTAGAGGTCTCTTTCTCCGTACTCTTTGTTTTCTACCCTCGAGACGACGAGGTCAAT
AATTCTTTTTAGCGCTGGGTGATTGGGAGTAACAGCAAAGAAAGCTTGATAAATTCCTGAAGACACGCCTTCATCTTTAG
CAGAAACGAGAGATTTGTCTCTTGGAAGCCAATCTCGAAGAGGGGAAAGGCAAACCATAGCGGAGTCTGCGTAAACTCCT
CCGTTCACCCACATCACGCAATATCTAAAAAGGTCTGCCTTGTACGCTCCTGGAACGAGCGAATCGTACGCCTTTACTAC
TTTCTGGTCAAAGTGCTCCACAAGAAACTCTCTTTGCTTTTCTGTGGGAAAGTACATATGTTTGTACTCCGAATTTTTCT
GGACCCAAGAGTCCAGTGCTCCTTTCATTCCAGGGAGGACTTTCTCTTCATTCGTTTGGAAGATGAGAAACGGCACTTTT
TGTTTGTACGTTGGCGTTTCTTTTCTTTCTTGAAATTCTGGATATTTTTCCCATTCGTTCTTCCCAAAGACTCGGCACTT
TACGTCCATAGAAAACTCCTCTTGAATGTTCCCTCGGAAGTCATCGAACCAAAGAAAAAGGAATGTGAAAAGACACAAAG
AGAGGAGAAAGACCAAAAGCCACATTACAGTTGGTGCGACCTTTCATAACGATGAACTGCCAAAGCGCTGATGAAGAAAG
GAAGCGCGAGATCGAAGAGAGAGTGCTTCGCCTAATGTTTTTGACTTTGCTTTGGCATTGCGCGTATAGAGACCTTGAAA
ATATTTTATAAAAAATATTTTTTATTTCTTTGAAACAGCCACAACCTTTCCTATCGCAAGAGTTTTTGACCTGTCGCGGA
TCACGAACCTCCCGAGCTTTGGAAACTTTTCAAAGGGTTCGACGAGAATTTGATCTTTCGTTGTTATGACGACCTTTCCG
ACGTTTCCCTTTTTGACAAGAAGAGATTTCATAAACTTTTCTCCTTGTTTGACTCCCAGCACCTTTTCGACGCAACACTC

GTGCTTTCCGAAAAAGAGCTGCATAACGCACTCTGTTCCTGGACAAAAGAGAGGAGACGGTCCCAGAACGTGGACGAGGC
AGAGAAGTTTTGAGTTTTTTTGGATGATGGAACCAGAAGAACAGAGAAAGTCTCCCTGCCTTATTTCGTCAACCCCCGAA
ATGCAAAGAGTCGCGTTCTCTCCGACTTCCACAAAAGGAACTTCTTGACAAAAGTCTGTGTTTATGGACAAAATTTGAAC
CTCTCTTTCCCCAGGACAAAGAAAAAAGTTCGAACCTTTTCCGACAACTCCTCTTTCCACCCTTCCAAACACAGAGTATT
TTCCTCCCTCCTTTTGGACAGACATAACAGAGAATCTCGTCTCTGAGCTTTTCTTCCTTTCCAGAGACACGGAAGATAAA
ACATCAAGAAGACATTGTCCTTCCCACCACGAATTCTCAAATTTTGCAGAGACATTCTCTGAAGAGAAACCAGATCCTGG
AACGAACGCGACATTGATGGGGGTGAATCCCCTTTCGACGAGACGCTTCTTTGTTTCTCTGACAATCTCGTTGTACCTTT
TTTGGTCCCAGCCGACGCTGTCCATTTTGTTTATAAACACAACGAGACGAGGAACTCCAAACGCTTTTACCAGAGTGAGA
TGTTCCAAAGTTTGTCCTTCTTCCGAGAGGCCTGCCTCAAATTCTCCCTTTCTTGCAGAAATCACCAAGACGCAGATGTC
AGCTCCCACGATGCCTTCGATGGCATTGGGAACATAATTTCTGTGGCCGGGAGCATCCAGAATCGTCCACCTTTTGCCAG
ACCATTCAAACACTTCGCGACCCACTTCTATCGTCTTACCTCTCCTCCTCTCTTCTGGGTCTGTATCGAACAAGAACGCT
CTTCCCCAGCTTTTTCCCGCGCTTTCTTCTGCCTCTCTTTCCACTTGTGAAAGGGCTCTTGGGTCGACCCTTTTCGTGTC
CACGAGAATGGTTCCACAAAGACTGGATTTTCCAGCGTCAACATGACCGAGAAACACGATGCTCATGTGCTGTCCCTCCA
TTTTTATGTTGGACCTTTTGCTCGAAAATTAGCAAAAAGAACAACCTTCCCCATAAAATGTCTCAAATACAAGATACTGA
CCGTCCCTTGTGTCCTCCCACAAATTCTCTGGAATTTGTTCTCCGTCGAGACGAACAAAAGAAATTTTTCTGTTCTTTTT
ATTATATCTCTTCACTATTTTCGGAATTATCTCTTTGGAGAGATGCTCTTTCGTGATGTCTCTTGTTATCACGAGAGGAA
AGTCTTTTCCTGGAGCCACTCTCAACCACAAAAGTTTCCTGTCCAAAGGAATTTCGCCGAATTGCGAAAGGCAAAGTCTC
TTCTTCAACTCTGGAATCCCTTCTTCTCTTTGCGCTCTCCTCTCCCAATTTTCGTAGACGATGTCGTTCTCTTGCCAATA
AAAATAAGGAGTCGAAGCACAGCATCCCATTATTTGGTCATTGTTCTATCTGCAATATCTCTTTCTTTCTGAACTCTGTT
TTTTTGCACAGTGTCCTCCGAAGCTCTCCAATCTCGAATTTCAACATGTTTGCCTCCCTCGCTTTCAAAAGGTATCCTGC
CCCGAATGAGGCTATCGTTGCAACGAGAGTGGTTGTGGGCACTCCGAACCAAAACATCACAGCGACAACAACGTAACTTA
GGACCATTACTTTTTACATCCAAAAGTAATATGGACAGAGTCGGAAAATATGGGCTTTTCGTAAAAAGAATCTCCCCGAA
AGACGCAGACGTCACAAAGGAAGCGCTGGAAACAGTGAACAACATGCTCGTCTTTCTTGCAGAGAAACTCACCAAACAAG
CAACCATCATTGTGGGGGACAAAAAGACGATAAAACACGATGTCTTTTTGTGGCTTTTGACAGACATCCAGGGAGAACTC
GGGAAGCATTCACAAGACTTTGCAAATTCTGTTTTGTATGGGGGAAAAGAGCTTGTTTTCCCCACAAAAAGAACAGAGAA
CCTCATGAGAAAAAACACCTGTTTGAGAATCAGTCAAAACGCTGTTAGAGCGTTGACGGCCATTCTCGAATATTTTTGTG
GACAAATTATGGAAGCATCTTTCTCTCAGGCAAAGAGAAACAAAAGAAAGAGAATTCGACCTTTCGAAGTTGAGGCCGCA
GTAGCCAAAGACAAAGAACTCTACTCAATGTTTGGGAAGGGTGTCATCTCTGGCAGATAAAATATTTTATGAATGAAAAT
ATTTAGTAGACCTTTTCTTTTCCCTTGTCCTTTTTCTGGGGCGTTTGAGGCTCTTCCTCCAAAGACCAACCTGTCGAGTC
GACTGGAACGTATAAAATTTCTTCCTTGGGAGAAATTCCTTCAAGAGGATTCTCGACCGAACAAATCTCCCAAGCGTTGG
GGCGAGCAGACAACACCACAAAAAGTTTTTTGTCTCTGTTCCTGACAAGTTTGCCGATCCAGGAATGTTGTATCGTTCCT
TCTTCGCAACAAGCGAGACTCCACCCCGTCGAGTCATCAGGGATGTACCAAGGAATTCCTTCTATTGGGAAACCACTGCT
CGTGTACTCGATCTGGCAAACCTTCCAAGCACCGACTCCTGAGTGTCTTAGCATCACTCTATAGAGCTTTTGATGCTTGT
CTCTTACGAGCTTGCCTATCCAAGAAGGTTGAAGTTTTCCGTCTTTTGTCTCGCACGTGTCGCTTCCGAGAGAAATTTCG
GAACTCTCGTTGTTTTTCGCAGTTTTTCCGATCCAGAACGGACTCATTCTGACTCTGGGAGGCTCGAGTGTTGACTTGTG
CTGGGCTCTCGGGAAGAAAATCTCAGAGGGAGTGAGAGGTCGCCAAAGAGTTTCTCTGCTGAACGAAAGACGGCTTTCGT
CAACGCTCAACCAACGAACAGGGTCAAGCGAGAGCCTTTGCCTTTCGACACGAATGTAATCGTCCCCCACAAACATCAAA
CGGTACACATCTTCCTTCTCATTTTTGATGAGACTTCCCAACCACTCTTGCTTGACAGGTTGAAGTTCTTGTTCCTTGTC
TCTGGGGTCTTTCTGGTGAAAGTCTTCCCTCCTCCTTGGAATGTCCAACGAAACATTTTGCTCGATCGTCTCTTCGGCGA
CCATGAGTTCGCATGTCAGCCTGTCAAGTTCTTCCAAAAGTCTCTTTCTCTCTTGCTTCTTCTCGCAAAGGTCGTACTCG
AGACGAATGACATCGATGTGCTCACGGAGAGCTTCGAGTTTTTCGAGAGTGAAGGAATCCATTGTGGGAATTTTGAAGGA
ATTTCTTCTTGGATTGTTCAATATTTTTTATAAAAAATATTTAGAAATCTCTCTTGTACTTTTTCTCGAAAACGTCCAAC
TCGCCCTTCCACATCTCTTTCGGTGTGCGTTCAGTGAGCTCTGCATGTTTCTCCTTGAGCTTTCTCAAAATCTCTCCGAC
TTCTCGGAGTCGTCCCTTTGTGAGAGTTCGGATGGGCATGCTCACAAGGTAATCATAGCTGTCTTTCCACTTGTCATAGC
CCATTTCCTCCATATTTTGGAGAAGAATTTCCTCTTGCATCTTCATATCGAGAGCGCCAGAGAGAACGTCCTGCAAAAAC
CTCTGCTTGTTTGCTTGTTTGAGAATTTCTTGGGAGAGGTCAGCGAGTATTTTGTCCTTTCTCTTTTGGTAGAGTTCGAG
ACGAGCAGGGCAGTATTTGTGGAGGATGTCATCCACAGTCGCAAATTTGATGGGATTATCCTTTTCGTCGAGAGCTGTCA
TGTTCCGAAGAGAGAACGTAGATGAGAGCTTGAGGTTCTTGACTGTCGGAATAAAGTCCTTTGTGGTGTTGATGACAAAA
GACGCCTTTTCGTCCGTGTACTTGTCCTCGAAAGACTTGATGTGCTTTTGGCTCTCAAGATTTTCCAGATGTTTTTTGCA
ATCGTAGGTCCAAAGTCCTACAGGAGTTTCTACAATCTTCCACTTTGTTTTGGACATCTTTTCAAGAACACCAAATGTTT
TCACCTTGCCTTCTTCCACTTCCATCGTTCCGTTGAACCCCTTCCACCAAGGAGAAAGTTTTGGAGTCTCTTGCTCATCC
AACCAGCAACGAATCCATTGACAAACTTCAAGCGGATTGTGAGGAGGAATTTCTGTGCTGTGACCAGTTCCGATGCCGCA
TGCTCCGTTCACCACAATCAGAGGGATAATGGGGATGTAGTTGATGGGTTCGTTATACTCATTATCCTCGAAAACCCTTT
CGTAGAGGTCATCGTCTTCCGGGGGAAAGACGATTCTGGTGACCTTTTCGAGGTAGGAACTGAGATATCTCGAAGCAGCA
TGGTCCTTCCCTCCACGAAGACGTGTCCCAAAAGACCCATCAGGGAAAAGGAGACGCACGTTGTTGCCGCAACCCACAAA
GTCTTGCGCCATTCCGATGATGGTGTTTGTGAGAGACTCTTCTCCGTGGTGATATCCGGCAACCGAAGCGACCTCTCCCG
TCAATCTGTCCACATGTTTCTTTTTGTTCTTTCCGATGATGTTTGAACTGAAACAGGCATAGAGGACCTTTCTTTGGGAT
GGTTTGAGCCCATCATACACAGAAGGGATGGATCTTCTGTTGTTTGCTCTGTGGTACGTGGACAACCTCTTGTCGATGAA
TTCAGAGAGAGTGAGTTCCCCCTCGTACGTTTCGTCTGGAGTCAAAGGTCCAGCCAACCACGTTTTCCTCTCGTTCGACT
TGTTCTTTGAAAATGCGAGGTCAAACCATGTGTTTTCCGTTCCATCAGAGACGTAAGAGACAAGCTTTTGGTCAGAGAGG
TACTTCCTTCCGTCTTCTGGTCTGCTCGAACCAAGACCTTTGAAATACTGGACTTTGAGGTTTTTGTGTCCTGGGTTCTT
TACGAGCCATTCTTCGAATTTTGTGGTTGAAAAGAACCACTCTTCTTTCTTGCCCAGAAAAGCCTTGAGAATGGGAGTTC
TCAGAGACTGAACGAACCCGTTCTGCAAAAGTTCCGGATGGTGTTTGTGGAAAAAATTGAGCACAAGTCCCTCGATGTGA
CTTCCGTCCATGTCTGCGTCGCACATCAGATAGACCTTTCCATATCTCAGAGTTTTTTGGAGGTCCTTGGACAGACCGAG
AACATTCCTCAAAAGTTCGAGCTCTTTGTTCTTTCCAATCATCTTTTGCGTTGCGTTTGTCGTGTTGAGCACCTTTCCCT
TCAGGGCAAAGGCGCCGTACCTTTCCCTATCTTTGAGAGAAGTGAAGCCAGAGACAGCCAGAGTTTTTGCTGAATCTCCC
TCTGTGAGAATGAGAATGCACTCTGCGCTTCTCGCTGTTCCGGCGAGCTGAGCGTCGTCGACCTTTGTAAGGTCGAGTTG
CAGCTTCTTTGTTTTCTTCGTCTTCTTGGGATCGAGACGAGAGGACAATTCTTGAACAAAGCCCCACTTCATCAACTTTT
TTGTTTGTTCTGGAGAAATTTTCATCGTCTTTGGAACGGGCGACGCAAGTTCGTGCTTTGTCTGGCTTGTAAACTCTGGC
TTGTCCAAGTCACAAACAACGAAAAGATGAAAGAAGCACTCGAGCTGCTTCATGCTCGCCTTTGGCTTCCCATCTGCCTT
TTGCTTTGCGTTGAACGCCTTGAGAAGGGGCATTAGAACAGCACTCTTCCATGCATTGACATGCACCCCAGTGAACACTT

CAAGACCGTTCACGAAAGAAAGGCTTGTTTCCTGGCTCGAATCAGCAGAAACTTCTTGGAGATTCTTTTCGAGAAGAGCG
AATCTCGAGTCGGGAGACGAAAGGCAGAGGACGTTTGGATTTTCACCAGAGAAAAGAGCCACATAATCTGAGAGGGATGG
GAAGGAGAATTTCTCTCCGTTGAAAGAAGCAGAGAGTCCTGTCACCAGCGACGCATCTCTCAACGTCTTTTGGAATCTGG
AAAGAAGTGGCTCGGTCCATCCATCAAGTCCAAATCTCGCAAAGTCTGGGAGAAAACGAACCTCCGTCCACCCTTTTTTC
TTTGTGCACTTTTCAATGACTGGAGGACTCCTCCTTGTGAGATTGTCGAAAAACTCTTGCTCAAAGATTCTCTGGTTCTC
TGGGTCAAAACAGCGAACAATAAAGTGCTTGGAGAAAATGGACGTGAGCTTCGCTCCCAGTCCGTTCCTTCCAGAAGTCT
TTCGAACTTCCTCGTCGTTGTAGTTTGTCGATGCCTTTGAGTAGCCAAAGAAGAGCTCTGCGGGGTACAGAAGAGAAGAC
GAGGTGTTTCCGAGTTCGTCGGTGTAGCTGTACTCTTTCTTCTCGACAGGAATGTGCGCTCCATCGTTCCACACAGTGCA
GTAACCCGTCTCCCTGTCTATGGAAACGTTGACAGAGCGCATCGGAACGCCAGAAACGGAAGAGCGAAAATAGTTGTCCA
CAGCATTGCTCACAATCTCGTAAAAAATGCGCTCAAGACCTTCTGGAAACTCTAGGGTTCCGAATTGCATCAACTTTTTC
TCCTCTGACCAGCACCATCTCTCGTGGGGAAGAATGCGGATGGAACCGAGGAAAGTTTCCGGACGATGGAGGGCGAATTG
GAAGTCGTTGAGTCTGCGTCCCGAGTCCATGAAGATATTTTATTTGAGATGTTTGTCTTTGAAAATATTTTGAAGAAAAT
ATTTGGTTTGGTTCAGCCATCAATACGGGATAATCGGTCCAGAAACTTCCTGTTCGAGAATGACGAGTTCTCCCAAAGTC
TGGGAAAAGTAGACATCAGGGATGTTCTCATACTTTTCTGGTACAAGGACTGTTATCGTTTTGAGAAATTCTTCGCTTAT
CCACCATCCGTGAATATCAGGAAAAATTTGCCCAAGTTCGTTCCAGGACATCGAATAAACAGTTTTGGTTGTTCAAATAA
ACAATGGAATTCTTCCTCTCTCCAAGAGATTTCTGCTGCTTTTGTTTGGTGTCTCCCGTCCCTTTCGATACGAAAGAAGA
GGAGCAACAATTTCAAAAGAGAAGTTGTTCCGTGGATTTGGACGAAGTGGAACGACTCCTGTTGGAAGAGAACTTTTCTT
CTCCGACTTCTATCCCTTCTTTCCAAGAGGACGAGAGAGGAGTGTTTTCCCATTCCAAGGAAACGTCGAGCATCATCAAA
GGGACAATCATCAAACATGGACCTTTTCGGAAGGAGGTGCTCAACGTTGCGAGGTTGTCGAGACATCCATTCAAGGGAGA
CTTTGAAATAAGAGGAAAGACGAGGATCGTGGTGGAAGGGGTCTATTTCCGAGGAAAACTCCACGACAGAATTTCGACCA
AGGTGTCGATGCCGTTCCAAAGATGGGACAAGGAAGTTCTCTCGACGCAAGAGGTCGCTTTCTACGATGAAGGACACTTT
GTCTCGAGGGAAAGGAGAGTCTATTCGGTAGACAGCAGTGTGGGATATTTTGAGAGAATCTGTCCCTTGTTTTCATAAAT
ATTTTTTGTTGAATAAAAATATGTTTGCGTTCCTCGAGGAAAAAGAACGTGTCAGTCTCTGCTTTGTTTTTCCAGAGAAT
CTCTGCGTTTCAGATGAAACGACTGAAGACGCCTGCTCCACAGACACGAAAGGGAAAAACATCACAAGGAAGAGAAACTT
TTGCTTCTTTCTGGATGGTCCAAAGGCCAGTTACGGCCCGTTTTGCAGTGTGTTTGAAAAATGGGAGAATCTCGAAAGAG
CGACTTGTTACACCCGTGTGAAAAAGACAGGGATGCATATCGACGGAAAGAAACACGGTCGTGTAATTGTGGAAACTTTT
GGACCAGCAGAGGTTCAAAAAGGAGACTGTTTGGATGGAGGAATGAAGGTGTTTCAAGAGTACGAGCACGGGAAATTTTT
AAGAGCAGAGTGGGGATGGTAAATATTTTGAACAAAATATTTTATTCGACAAGTTTTCCTTGTTTGAAAGTCCACGACTT
TACAGTCTTTCCAGACGCATCCTTCATCGTCCGCACTCCATCCTCAAGTCCGTTTTTGTAGAAGGATGTGATTGTTGAGA
CGAGTTGCATCTCCTTGTTTCTCGAATGGAAGAGCCAGTTTCTCTGGGTAAACTCTCCCTCAAGTTTCCCATTTGCGTAT
GTTTTCTCAATGTCCTTCCGAAAGATTGTCTTCTTCTCAACAAGAGAGTTTTGAAGGGAAGACCAAACAGACGAGATTGT
TTCCGTCTCGTTGAACGAGAGAAAAGGTCCATGACGGAGCTCTGTCCCTTCCAAGACAGAAAATGTTTTCCTGAGAGTCT
TTGTCACTCTTTCGGTGAAATTCTTTCCCTTTGCTGGGCCAATGTGGACGGTTCTGGGAACCACACAAGTCGTCTGACCG
TTCCTTTCTATGAGAGTGAGCGACATCTTTCTGTACAAAAACTTTTCTCAAAAAGTTTTATCTTGTCTTTGATTCAAAAA
GAGAAATATTTCTCTTGTTCTTTGAGCTGAAAGAGCAGAACTCTTTTTTATGGATGGAAGGCGCTATTCTGATGTGAAGG
TCGCACACAGAGAGAACAGCCCAAAAAGGCATCTTTCGAGAGTGAGTTTTTCCAGAGCACAAAGTTTTTGAATTAAAAAA
ATGGGCCAAACATCCACTTTATTTCACAATGTCTTTTCGAACAGGTTTCACCAAAAAAAATCTTGTCGCTGTTTCTTTCC
GCGACATGATGAACACGGGTCTCATCGACCTGGAACCTCCATACCAAAGGGAGATAGTCTGGAACGAAGAACTAATGAGT
TTGCTCATCCAAAGTATGCTCTTGCGTATCAACATCGGAGAAATAACTTTGATGGAACTTGGCCAGGACGGATACCGTTC
TATCGACGGAAAACAAAGGCTTACTTCTATCGGAAAATTTATGAACAACGAAATTCCAACGTGGTACGAAAAGCAAGAAG
AACAGAAAATTTGGTACACAAAAGTCCCGAAAAATTCGAGGAATTCGCGAGTGCTGACTGAGTCAGAAAGAGGTCTCATT
GACTTTTATGAGCTCACGACATGCGTCTATCCCACCATCCCCTTGGAAAGACAAAAGGACATTTTTAAGAGACTGCAATA
TGGAAGAAGTTTGTCATCCGGTGAAAAGCTTTTTGCACAGTGCGGAGACACAGCAGAAACAATGATGGTCCATCTTGCGC
CACTCCTGCCCAAGTCCTGTATTTTGTCGCATCAAGTCAAAAGGAAGAGCTACATCATCGCGTTCGCTCGACTCGCGATG
CTTATTTCCCATTTCTCCGAGAGTTTTGGACCCCCTCCGAATACATACCTCGAATCTGCAAAAGTTCTAGAGTGGTCCAA
GCGGAAGGGGTACAACAAAAAACTCTTGAGGCACTCAAAGGAGGTTCTTTCTCTTTTTGCAGAGTTTTGTTCTAATGAAG
AGAAGTGGACTATGAACAACCCTCTTTTGGTGTATCTCGGGACTTATATTTTCCTCAACAGAAGAAGTCTGAGAAAACTG
GAAGATGAAGAAAAGAGTTTGAAGATCCTCGAGATGAAGAAGGAGATCTCAGAAATTCTGGAGAAGCACAAAAAAGCAGG
AGCCTATTTCAAGGGACGATGTATCAAGGAATTCACTGAATTTTGCAGCGTCTGAATATATTTTATCCAAATAAAATATT
GTACGACAGAGGACAAGGTTGTCATCGCGATAAGAGGGCACATTTTATGAAGGAGACTAACACTGAAAATATTTTCCAAA
TAAATCTCAAAAGAGAAGGTTCAAATTCAGAAATTTTTTACAATGTCTTTTCAGTGCTCTGTTGCCGATGAAGGGAACGG
TCTCGTCGGTCTCGTCGACCTCGTCTTCCAGTACAAAGAGGGAAGAGCTTCGTTCTGGATAAACTGCTGTTCTGTGGAGA
ACAAGGATTTGCAAGAGATTGTTTGCAAGTTGCTGGATGGAGTAAGCCAGGCAAAACACGAGGAGATTGTCGGTGTTGAG
CTCAAAGACAAAAGACTCAGTCTCGAATATTTTTGTGGAGACCTCACCTTCCAACACGAAAGCGGAAATGGGCAGTATAG
TGTGTGCGTTCCTTTCGACAAGTGCAAAGAGGGTCTGGAGAAACTCTGCGAAGAACTCGAAAGGCACTTGTTACACCTGA
CAGGAGAGCATTGCGTCGATGATTCAGGGAGCGATGAAGAATCGGAGGAGAGCGAAAGTGAAGAGTCAGAGGAAAAGGAT
GTCGAGGAGACTCCGTATTAGATATTTTATTCGAATAAAATATTCAGAATTTTGTTTGCTCGTCAAGAGGGACAATCTCG
ACAAACTATTTCGAGACTCTTTTCATCCACAAGTAAAATATTTTATCGAGAGGCAGAGAGTGCTTTTGAAATTCTTTCCT
TTCACGATGGATTGGAACGACTACACCATCTATGTCGACGCTTCTCGCAAGGGAGACGAGTGGAAATTCAAACTCTCGAC
GAGCAAAAGCGAGAGGAGCATGTGCTGGGTGAAGCTCAGCACAAAGATTTCTCTTGACGGGTTTGGCTTTCCCAAAAGGG
GGAATGTCGGAGTCGCGATGCTCACGATTAAAGAATTTTTGGAGACTTGTTACGATGTGCACATAAAAGCCGCGAATGAA
AGGTCAGACAAGCTCACTCAAGACATCCAAATTTTCGAGGCTGCCCTGACCCATTCCCCAAAGGAACAGCACAAACGTAT
CAACGAGTTGCTGAAAGAAGCTCGAACGGAGAGGAACCGTCTTTTTGGTTTTTTCATGAGACGCACAATGATGTCGTTTA
TAAGAGACGAGATAGTGACTCCAAGGAAAACCATTGAGTTTTCTGGCTCTGCTATACTGTGGTTTTTCGGTCGCGTCGAT
GGTCGCCTTTACATCGACAATCCGATTAGAAGTCTGTTTGGAAGGATGAGAGAGTCTGAGGAGGGAGAATTACCCAAAGA
GCCCGAAGAGGAAGAGTCTGACGATGAAAGTTCAGACAGTTCTGATGATGAGGTACGTTTTTGAGTTTTGGAGGGTGGAA
GAAGAATATTTTATTCAGAAAAATATTCAAAGTGTTTATGTGCTTGGTGTATTGGCTTGCTGGATGTTTTTCTTTGTTTG
GAAAGAAAAAATTATACTTGTTGTTTTGAAAGTTCAAGGAGTCTCCTGAGTTCTTTTACTTCTTCCTCTTTTTGCAAAAG
GAGAATATGTTCTTCCTCCCTCTTTTTTCTGTCTTCCGCATTTTTGGCGAGAACTTTCTCTCTATTTCTCCAATACCATG
ACTTTGCGTGCTCTCTAGTTTTTTCTCTGTTCTTCTCCCGAAAAGTGCGAGAGTAATCCTTTTGGGTCTTCTTCTCTGTT
GTCAGGGACATAATATTACAATATATTATATCGCAAATATATTCTTCCAACAATTTTTAGAAGCGTTTAAAGAAAAAGAA

TTGTCGGGTAATGAGCTCCACTAAAGTTCATCAAACGCCGACAACTAGTTATTCAACTTTTTCTAGTTATACCCGTTTCT
ATCCGAAAACTTTTTGTGAAGACCAGACATCGTGTCCTCTCTGTGAATTTTCTGGTTCTTCAAAGGGAGACTTGAGAAAA
CATTATGTCGGAAGCTTTCACAAGCTCAAGTTTGAACAAGAGACAAAGAAGAACGCGGTCGTTTCGTTGGAATGGGAGAG
GAAAGTGGGAGAATCAGAGAGACACTGGGAGGTTCGAACCAGCCTCCTCGAGCATATCGGCTTGCCTCCGGAATATCTCA
AAATAACTTTGGAAGAAAGGGTATCACATTTTCCAAACGGTCGGAATTTTAGTCTCTTCGGACAACTTTGCTTTTGTTTC
CGGAATTTTTCCGTTGTGATTCCAAGGAGCCGAGAAGAGAAGGTGCCATTTCGCTCTCTTAGACTTTCCGTTTCTCCCAA
CATAGCATCCAAACATTTTCCTCGACACATCGTTCGCCCACCTCACAAACAGACTTTCTTCCTTTGACACCTTCCCAAAC
AGAGCTTGGAAATTCTTGCTCTTCAGAACGACTTCTCTCGCATTCCCTATCCTCGAAGCCATCTCAGAGCTTTTTATTCT
CTTCCCTTCGCAGACGTCTTCAAACCCAAGAAGCTTCATCAACCTCTTTGCATAAACGACCTTTTCCAATGTTAGATCTC
CATGGAGCTTCTCTATCTCTGAATAGTTTTGCTTCTTGATATTTTTCTCATCTGCCATTGTTCTAAGCCTCTCATCTTGT
TGTTCCTTTGTTCCTTCGAACGCCAATCTCTGATTGAAGAACTGCTTCTCCAATCCCTTGTAAGTATCCACAAACTCGTA
CGTGACATCACCTTGTTCCACCTCGAAGCGATATGCCATCAGCCATTTCTTGATCTTGAAATTCTGCTCTTTTGTTCTTT
CACGAAGTGTGCAGAGGTGCTGAAACTCTTCGTTCGTTGTCTGTTCAGCGGAAGAAATTCCTCTCGTATCTTCTTCCGGT
TCTATTTTTTCATCCGATTCTATTTTTTCAGAAGGCTCACAAAGAACATTTTCGGAATTGTCTATAACTCCAAATGGCAA
AAGTTTCGCGTGGTGAATTCTGCACATTTCTTCTTTTGTTCGTTCTACTTCGTCTTTGACAGACAGAAAAAGTTTCAAAA
CTGCTTCTTCATCCTTCCACAAAAAATATTCATTCCCTTTTGCTGTGAAACACTTTCCACGAAAACTCTCAATGACAAGC
TTCTCGCTCTGAGAAATAAAATCTGTCTCAAACATCGCGAGGACTCTGACGTTCCCGTAAGAATTCTTCCTTGATTTCCA
ATTCGAGGACTTTCCGACCTTGTATACGCCCTTTTTCCAAATAGCGTCGTCTCTCAGCAGATAAATTATTCCCTTATCCA
TTACAAATGTCGCCTGTTCTCGAAAACAAAAGGCCGTTTTCTTTTGTTTTCTGGTTGTTTTAAGAAGGGTATTTGAACAT
ATATTTTTGAACCAGAACTTATGGGAGAGTGAAATGTATATAGATGGACTCTCTGGTATATAACTCTGAACATTTCGACA
TCACACAAATATTTTATCAACAAAATATTTATTCTCAGATATCCAAATTCTCTTCTTCAAAAGACGAAACAGGGAGGAAC
TTGTCTATCCATTCGGGCTGTGGAACGAATACTCCGTCTTCTGAAAATTCGTCTTCTTCACCCCTTGTCTTAAATCTCGA
TATCAAGCGCCAGCTACAACGTTTGTTCATGCTCGTCTTGCCAAAAGAAACCCCAAACACCTCTCTCAAAACCTTATTTA
TCCAAGCGATTGGAGACTTTGCCAAACATCCGAAAAAGTGAACGTACAACCTCTCTTGTTCTTTCACCCTTTCAATCTCC
TTCGCAACGGAACGCGCAAAGATTGCATTCTTCACTTTCTTCCCAATGTCAAAATCAGAGAATCCGAGACCGTCGAGCAA
CTCCCACGCAAGGTACACTTTTTCTATGTTTTTCTTCTCTGAACGAAGGATTCTCTCACCCAAAGACAAATATCTTCCTT
CTCGAACTTCTTCCCGAATGATATTCCAAAGTCTTTCTCTCACCTCGTTCCTCTCACCAGAGGCGAAGCATCTGTTTTTG
AACTGTTGAGAGAATTTGCGATAATTCCAGACAAAGTCAAAACAGAGTTTTTCTGGTGGGACGCGGAAGGTTTTGCACAG
AGAGTATCTTTGACAAGAGCGCTTTTCTTGAACGTTTGCGTATCCTCTCGAATTTATTTCTTTGTGCTTGTCTTCCGCTA
TAATTTCAGAGACGAGGATATTGTGGCATTCCACCTCGTCGTTGACGACAGAGATTTCCTTCATCTCCTCTGAGATGCAC
TCGAGTTCCCTTTGTTCACAGAAAATGCTTTCCTCTTCCAATGTCATTCCTTGGTGTTTCAGATATTCAAAGAGTTGCTC
TTTGAACCATCTTTTGGAGAGATTGTTTCGAAGGGCGTTTTGGAGGTACTGGGACGTGAATTCGTTCTTTTCAAGTTCAT
CGGTCAACACTCGAAGACGAACGCCGCGAGGGATGACAACTTTCTCTTCCTTTTTCTTGTTCTGTATTTTCAGGCACCTT
TTTCCAGAGCAAAGGTCCATCTCGATGTGTCGCAACAGAGACTCAAGAGAACACGGAACGTTTGCGCTTCTCGAGTTGAC
AACACCGACAAAGTTTTGAGATGAGATGTTCCGAACGCGGAATATCATCTGAACACTGTCGACGACGTTGCACGAGAGAC
TCGTGAAGTAGAAGAACACTCTATCAAAATGTGGGTCTTCGAAGCTCACACCAGCGGTGATACAACTCGTGTAAGCGATG
ACGCGGTAGTTTTTCCAAACGCTCGTGTCCACAGGTTCTCCATTCATCGTGTCTGCCGTGTACCTGAGAATTTTTCCTTC
CATTCCCGGTTCTTCTTCGAGCATGTCACAAAGGATATGAAGTTTCTTTTCGCTCGTCATCGCCAGAACAATGTTTTCTC
CATCTTTCAGGGACCTCTGAGCCGCCAAACAAAGCTCTTCCACGCTAGGAAGGATTGTACATGTTTTTTCTACGTGGGCT
TTGTGGTCGTTTACCACCACATGCACCTTCCTTCCAAAGTCTCTCAAAAGTTGCACAGAAGATTCAGTCAGAAACGCGTC
CAGCACAATCACAACGTCCGCGTTCTGGACAAGTTCTCTCAGAACTTCGAAACATTCCATGGGTTCGCGACACACGGACA
TCATCTGATTTATGGTCTCTGAGAACTCATCGATCACGACGACAGAATAATGGCCAGACATCCTCGCGATAGAGTCGATA
GAGCACACCAATTTTTTGCAACCACGGATTTTATAGTCTTTGAAATCTGAATAAAGCGAGAACCCAGCATTCTTCATCGT
TAGCTCTCTGTTGAGCGACTTTCTGTACGAAAGGATGACTTGTGTATGTGTATTCTCTTTGAGAAACTTTGAGACAAATT
TGGTCTTTCCTGTGCCGAGATTCGACTGGATAGCGAGCGCATCTTTTCCTTCTGGAATTTGACCATAAACAAACTCTTTT
GAAGTTACCGTTTCGTCCGCTTTTGGTAGAAACATCTCCCTTTCGAGAGTTTTAAATTCTGGCTTAATCCAGCCAATCTT
TTCGAGCTCCTCGTCGAGTTTTTGCAAGAGACTCGGATTCTCTCGGTTGTCCTCACGAAAGTTGTTCCTCATCGCTCTCA
GAGACACAGGGTGCTTGTACGACACAGCCAATGAAGCGTTTCCTTCTATCCATTTGTCAAATTCCGTTTCGTCATATCCC
TCAGTCTCCATCGAGAATTCTCTCAAGAGTTCTTTGCCAACTTCTTCATCTTTTGTGATACCAAAGATAAAAAAGGCAAC
GTCTCGCCATTCGTTATAATCTTTCCTTCTTTCTTCAGAGAGACCAGACAACAGGAATCGGAGAGGTTCCATATCGCCTT
GCACGACAAAGGTCTTTTCCACTTTTTGAGACTGTTTTCTGATGGTTGGTGGCTTTGGACCAATGTCTGCCCACTTGGAT
GAATGGCAGCTGCAAAAGACGTCTTTGAAAGATATTTCGTCCTCCCACCCATCAAAGAGTTCAAAGTATCTTGTCTCTCC
GTATTTCGCTGAGCCGAGGACCCTCATCTGCCTGTTCTTTGTATAGATGCTGAAGTCCAGCATTTTGAGAACAAGGTCGT
ACTCTGGTCTCTCCTCCAACCAAGAGCGAAACACATCGACGAACCCCTTCAGCGCTCCGACATCTTTGTGCATTCTATTC
ACAAAAATTCTGAGGCTGAGTTTATATTTTTCTCCCTTTTGACCAGAGCTCTTGAGAGGAACAATTTTAAGCTTCCCTTC
CGTTCCAAACTCTTTTCTCGCCTTGCAAAACAGCTTGTAGACCCTGTTTTCAGAGACTTGACCTTTCGAGAATTCCAAAG
TTTCCAGGTCGTAGATTTCCTTGAGAGGTTTGCCCTCCAACATCATCTCTCGAAACCATTTCTTCCTCCTTGGAACTGTC
TCGTAAAACTCCTCGAATTTTTGAATGCTCTCGAATGGCGCGTACATCGGCATCATCGTTTTCTCATGGGTCGTGACAAA
GCACATGTCAAAGCCCTTTCTCATCCCATAGCGAAGAGCGAACCACTGCGCTCCCTTCCCTTCGAGATATCTCTCTCCAG
CGGAAATTCTGTTCTTTCTGCTCTCTTGAAAGGTTCTTGGAAGCTCGTAGAAAAAGACGACATTCATCTCTCGATGCCAT
TCCACGCGAAGATATGGAGGGATTAGACCACAATATCTCAAAATCTCCTTCTTCTTTCGAAGAACGTCCTCGTCTCCACC
CTTTGAAACAAGCCAAAGAACGGACGGATCATGATCTTGCTGAGACTTTGTTACGAGTTTCTCCCATTTTTCACGATGCT
CTTGAGATGTCCAGTGTTGTTCTATGGAAAAACCAAGTTTCTCTCTGAAGTTGCACATCGGGCATTCAGAAGCTCCGTCT
CGAAACTTTTTCGTCTCTGGCTCATTGTCGAAAGAAAGGGTGTATTTTTTGGGATAACTAGAAAAAGCGTTTGTGTTAGT
TGCCGTGTGAGTTTGTCTCTTTCCAGGGAGGGAACTCATTACACGGCAACATATTTTTTCTTAAATTTTTCTAATGAATT
CATTAGAAATAAAATCACAAGAAGGGAACTCTTGTAAGGTAAGAAACAGTATGTCGGTGGTCGCGCAAAAGAAGACCCAG
AAGGATTATTCGAGAGAATATAGAGAAAAAAACAGGGAAAAGACCAGAGAACAAGCCAAAGCATGGTACTATCGCAACAG
AGAGAAAGCTCTCGCTCACAAGGTGGAAGTCAACAAGCGAAAAAAGGAAGAGAGAGAAAAAGAACTTCTCCTTATCCAAC
AGCAACAAGAAGAGCTCGAACGTCTCCGTTCGCTACTCGATGAAGCGAACAAGTGCATTTTCTCCAAATAAAATATTTTT
GTTTGTAAAAATATTTCAAAATCGTGGTCCTCTTTTCCCATTTATTCTCTTGATACATTTTGCCCATGCATAACTTTTTC
AAAAATAGAGAACTCGCCTCTTTCGCTCTCGTGGAAGACATAGACATCCAACCAGAAGAATTTGTGCGAACTTCAGGCAT

CACAAAGGAAGAGGGAAAGGATTGGAGTTACACCAAAAGTGGTCATCTCCCAAACGGAACAAGTCACGGCACTTATTCCA
AAGAGGGGAAAAAGAACGGCTGGGAAAAGGAAAAGAGACTAAACTACAAAATGGGAAAGCTTCACGGTTCCTTCTATGGT
TCTTTCCGTAACGCCTTTTTCTTTGAATGCTCTGGAAATTTCGAAGAAGGCGTCCCTGTGGGAAAGTTCCGTTTTTGTGG
AGAATTTATGAGGATCAAGAAAGAAGAAAGTTTCCTTTCTTTCGAGAACGGTATGCCGACGTTTTTTGAAGGCGTCAGAA
ACTTTCCCATAATTTGGGAAGGGAAAACCCTTTCTTTGGACGGAAAAGTATACAAGAACATCTCCTTCTCTGAACAAAAA
TTTTTGTTCTTTGAGTCTGAAACACCTCTCTCTGTCGGATTTTGGAAATGTCCGTCGCACATTCTCGAGAAGTATTCAAC
AAAAGTTTATGGGACAAACGAACAAGGAAAGATGAAGAGAATTCACATCCCTGTGTTTCCCCAGTAATATTTTTACGAGT
AAAAATATTACTACAAAGTCTCTTCTTTGGGAAGAGGCGCGAAGAAACTCTGTCTGCGTGTCGTTGTCCAAGACGCAACG
TTTACTGTATTTGGCGGCTCGATGGACCCATCCGCTCTTTTGTTGTACGCTCTTCCGGAAGAGCGAAAGGGACGAGCTCT
CTTTTTTCGAGGAATTTTTCAGTGTCCATGTCCCAGATGAAAACTCCAAAAATGTCTCTCTGTTTTCGCTTCGAAAGGAC
GAATGGAGCGACATTTTTCTATCAACATATTTTTATGGGTAAAAATATTCTGTCAAAGGTAGACTTTGGAAACTTTCTCT
TTCCTTGGGAAAGGCAACCTCACAACAGTCAAAATCCCTGTGATATCTTCACTTGAGCAAAAATGTCTCTCTCCTCCCCT
TTTTGTACACGCTCATCGTCTCAAGAGGACACATCGACCTTCCCTCTGTGTCAGAGACAAGATATTTCTTTTGCGAAAAT
TCCCCTTCAAGCTTCCCATTAAAAAATGTTCCAAAAGACTCGAGTTCCATGTGCTTTCGTTTCTCCACGATTTTGAATGG
ACCGTGTCTGGTGTTGGTACCCAGCAAGTACGAAAACGTCCCGGTTTCCTCCACAGCGACGCGTTTGCCATTCAAGGTCT
CTTCCCACCTTTCTGTTTCTTTTCTTTCTACAAGTCTCTGCTCAACAACCAAATAGAGCGCCGACCATTCCCTTGGTTCC
AAAAACGTTTCCATGTTTTCCCTTCTCGTTGCACTCGACAGTGTCAGTTTCTTTCGATATGAGAACTTCTTGTCCAAACA
TCGTCATCCAAGTTTCTCCCTCGAATGCGATTGTCTCTCCGTTGAAGGCAATTCCTTTCACTTCGACGCGGATGGGACCG
ACATCGCCATTTTTCTGTGTGATGATGGTCAAGGAAAACGGTCCATGAACAAGTTTGTCTCCCTTCCGAGAAGTGGACCA
TCCCTTGTAGTTGGAGACAATCTCTAGTTCCTGGTCGCTGGACCCCATTTTTTGTTTAGTCACAATCTCCTCTCCTTCTA
TGTTTGTTTGCATCTCAGTCGGACAGACGAGTTCCAATGAGAGCCACCCTCTTCTTTGGAGAAATTTGTCTCGTCTCAAA
GGTGAATATTTTTACTCATAAAAATATTTTGATGCGCTTCCCGTCCGCGTACACCACAGAGTTTTGAAGGTCTCCATTGG
TGTCTGTCAGCTTCCAAGGGCCGTCGAGAACACCTCTTTTGTAAGAGCTGAGAATTGTCGAATGCAGGTACAATGCGCCT
GCGCGGCCTGTCAGCCAGCTTTTCTGGCTCATTTCTCCCTCAAGTTTTCCATTGACAAAAGTTCCGCAAAGTTCTGTTTT
GTGCTCAGTCTCTTTCTTGACAAGTCTACCAGAAAAAAATACCCCAAACAAATGTCGTGGTTTTTGTGGCGACAACAGATT
CGAAAGGACCATGACGGATCTTTGTTCCCTTTCGAAAGGAGTAAGTTCCTTCCGTGACTCTTTCGACCTTCTCTGTGTAT
TTTGACCCCTTGCCGCTGCCTGCGGTCATGGTCTTTGGAGTTTCAACGACAACGTGTTTTTTCTTTGGTCTCTGTTCCAG
TTCGGAACAAGAGACGATTTCGTAACTGGCGCATTCTCTGGGTTCGAGGAAGCACTCCATCTTGTGATAAAACTTTTCTT
GAAAAGTTTTATGTGTCCAAACGATTGTTCCACAAAAATCACGTCTTTTGAGAAATGTCAGAGAGACACTTGGAAACGTT
CTTTCTTTGTTTTTTCGTCCTTTCTCCCGTATAAACGCAATACATTCTATGGACTCTTTCTTCACAAATCCTGATGTTGC
TGCCCCATGTCGTGAAATGGAATCCGTCTTCTTCGCCGTAACAACCTCCGTCAAACGTGAACCAAACAATTTCGTACACT
CTCCCGAACCAGGTCTCAGAAACGCCAAAGTAAAGAGTTTTTCCAAAAAGAAAAGGGTCCACCAAAACCCTTTCGATGAT
GTCCCTGAGTTCTGTCTTTAGCTCCGAAAAGGCTCTTTCATCTCCGTGAGCCACAATTCTGCCGTCACAGAAAGAGTATC
CACAAGTTCTTTTTTGCATTTACCCTGTTCTTTACCCAGCAAAACGTTTCGATAACATCTTTCAAAATTCGACCTGTTTG
CCGTTCCGATGCAGCCTCGTGATGACTTGACCTTCTGGGAATTTCACTGTTTGAATTCCAAGCTTTTCTCCTTGTTTGTA
TGTTGTAGTTGTCGCTCTACGGAAAATATTCTCTCCATTTTTTACAAAAAAGAACTCTTCCTTCTTTTCTTTGACTTTGA
GTTTGCCGTCGATGTACCTTCTACGAGTTCGTATCAGTCTGTTGTACACATCTGGGCCATTTCCTTTTCTTCTGCCGAGT
TCCAGCACAGAGAAGGAAGGTCCATGTCTGACATTCGTTCCCTTCCGGAAGGAAATGGCGCTCTTGCGTTTGAGGAGAAC
GAATGTGTTCACACCTTCTCTCGACCAGGAAGCTGCGATCTTGCTTAGCGTCTCCTTTTTTACTTCCTCTTCTCTGACTT
CAATGTCATCAGAGGCGACAACGAACGAAAGGATGTCATCGTCAAAGAGAAACTCTTCCATCTATGAAACTTTTTGAACA
AAAAGTTTCATGTTGTCTTTGATATTCTAAAATCCGAGGTACCCTCTATACGCAGTCGAATGGTTGGCGAGCACTGTGAG
CAAGTTGTCTGTGAACGTGTAGTTTGTGCCGACGTGTTCATAGAAAAGCGTCTTGTCCAAATCCCAAATCTCTGACGCGA
TCGAAGGGGGAAGTTCAGGAACGATATCTTCAGAGTTTGCGTACCTCAGAATTTCGAGATGCGAGATCGCTTGTGCTGCG
CTTGGGGAGAGACATCTGGGAGCAGCAAAAGTGCACGATTTCTTGAGATGAGAGTCCAAGGAAAAATCTAAAATGGCGAG
CTGGGCCACACCCCCACCCAAACTGTATCCTGTGACGTACAGGTCTCCAGGGTATTGACGTGTCAGGCTAATAAGCTGTT
GCTGGATGCTCGCATAGGCATCGTAAAATCCCTGATGGACAGAAAGGCCAGCGCTCGGCACGCGAAGTTTCTTTGGTTCC
ACCTGGTCATAAGTCACGTCTGTTATCCAATCTATCGCAGTTTCGCTTCCGCCAAAGGCGATGACAGAGACGTTCTTTGA
GCTGCTATGGAGAAAGAGGGCGTAAGTTTCAAGCTCTTGCGAAGTGCTGAAATTCGGCTTTATTTCCTGGACAACGTCAA
AATCTGGAGGGGGAGGGACTCTTTGTGTCGTGAGTTTGGCTGAAGTGGCGTTCCAAGAATTTGCAAACAAAAACCTCGAA
AGGTCGGGCTCTGTTGGGTTGTCCCAGTCCACGGAAGGGTAGGGTAAGAAAGTCTGGTTCTCTGCCCCGTAAAGTTTCCC
CTTTAGTTGAGGAGTTTTTCTCTCCTCTCTCCCATCAAAGAAGGCGAACCAAACCACAAATATCGCAAAGAGAATAAATC
CGAGAGTCACGAAAATATGAAGTTTCATCGTTACACTAAGTCTCGGAACAACTATTTGTTTTCTCTAAAACAAATATGGG
TAAGATGTCCCAAGAAAGGGAATGGCTCCCTCTTTTGCACTTTGAGTACGAAAATATCTGGATTTGTCTTTGCAAGAAAG
AAGGAGCTTGTCAAGAACTTGTTCGTATCTCAGAGACGACAATTCCCAGAACACAAGACGACATCTCAAGTTTTTTCCCT
TGGTTGGAAGAGGGAGAAGACAAAGAACTTTTTCTCGAGACCTTCCGTTCAGCATTCTCTCCAAGGACCGGTTATTGGCT
GCAAGTGTTTGGAGACTGCGTCTTCTTTGTGTCTGAAAAACAAAAGACAAAGAAAGTCTGCGAGATTGTCTTTTCCAAGC
CGATGGAATTTTCTCTCGGAAGGAGAAGAGATACGAAAAAATGGGAGTTTCTCTAGAAATATTTTTTGTGGATAAAAATA
TACCTTTTTCTTTGGAAGAAACTTTTCTGTTCAAGATCACAGGCCTTTTTTGGAGTTTTCTGGGAAAGCGCACCAGGAAA
AGGGAACGACAATCTCGAACAATTCTCCGCAAACCCCCTTCCCGTAAACTCTAGGAAGAGAAAATTTCTTCCCGACTTTT
CTCATCAAGTTGTTTGTGGAATCCCGTCTTTTCGTGAATATGCTCGTTTGCAAGCCGACGAGGAGCGGCGCGCTTCGTTC
CGTCTTTTCTGTGCGCACCTTTCTGTAGGGATATCCCACTTTCGCAGGAAAGAAACGTCGCTCTTCTTTGTCATAGACTC
GGAAGGAAGTCCCTTCCTCCTTTATTTCACATGTCGATGGAATTCCATCGACATGTGAAATGTGGAACGTCGGCTCTTCT
TTTGTTTTGAGCTCGAGCCATTCGAGTTTTCCTTTCTCAAAAGACCCAGAAATGTGAGAGAGTTTACCTCGTTTGAGTCT
CTTCAGCCAAAACTTTCCGTGGGGAATCCCGTCTCGATAAGAAGCGCTGAAACCAGTGACGACACCGTTCCTTTTTTTCT
TTTTGTATTGTTTTCCGTGCTTGACATCTCCAGGAAGAAACTTTTCTTTTACGAGATATTCTTTCCAACCTTGTTTCTCT
TGTTTTATTTTTGTGGTCAAATGCTGCCTTTTCAAAGGCAAACGCCCCTCTCCGTTGGCAAAGAAAGAGACGAGTTCCCT
CTTCTCAAAGAAGCTCTCCATTCCGAAAAAGAGGACAACATTTTGCTCTGTAGAATTCTTTTTGTCGTCTAGAAATATTT
TTATTCACAAAAATATTGTACAAAAGTTTTCTCTCTCCTTTACGCGCAGACGCCAAAAGCAGAAGTTGGCGCATCGAGAC
ATTTCTGCCACCAGCCGCAGTCAGAGTCTCTGGTGCATTGACCCGGCTTTCCGCAATACTGCGAGCCGGAAATCCAAAAG
ACTTTGTTTGGCGACTTGCATCGACAAGGCTCTTGGTACCCTCCAGAAAATCCCTCATTGCACAGACAGGGTTTGAAAGG
AGTGTACGGATTCGGAGGTTCAGAGTCTCGACATTTCACTTGGTTATAATCTTGGGCATAACAGTCGATGCGCTCCAAAC

ACTTTCCTTTCTCGATGCAGTAAGCCCTTCCTTGATACCAGCGCATCCGAGAGTCTTCTGGACATTGACAGGTTCCGGTG
AGAGGATTCCATTCGAAAGTGTCTTGACATTTACAGGTTTTGTTGGCACACTGAGAGAAACTTCCGCATTGGTAGTCGAA
ACTGCATTGCTTTTTCGCACAACTGCCACCGCTCCCGATGAATCCGTCATTGCACTTGCAGCTCCAAAGGGGAGTTTTAA
GGCTCGGGAAAGTTACGTCGCAATGGGCGTTGTTTGAAGCGCAAGAGCTGCAATTCGGGAGACACTTGTCCATGCAAGTC
ATCGAGTTCTTTCCCGTGTGCGGGCAATGGTACGCAGGGTCGATGTAAGAGTTGAAAAGGTGCAAACTACGACATCTGAA
GGTGTCTGAGCTGTAGGGGAATGGACAAGGAGAGTTTCTCGAGTCGATGTAATCCACGCACTCTTGGACAGATTCGTACT
GCTGAAGAGGTCCAGTGCACGTCGCCATAATTCCTTGACAGATGTCGAACGTGGTTCCTGCTTCTTGTTTTTCAAAGATT
TGTTCTGCGTCCTTTGAGTTGATGACATAGTCTGAAATGATGGTCAAACTGCAAGGTTTGAACCAGATGTACTCCTTGTT
TCTCGTTCCGCCGATGACAATGTCGTATTTTCCTGCCGTGTAGTTGTAGTTGACCTTTACGATGCCTCCTTGGTAAAACT
CTGCCATGTCTGGTTCCGTCCACTTCAGGGTCGTTTCATCCAGATAATTTTCGAGATACGTTGGAGGCACCATCCCAGGA
AAGAGAGGGAATAGAACGGCAAGATACTCACTCGCTATGAGCGCAGTGTTAAAGTCTCCGATGCCTCTTATCGTGTAGCT
TACGTTAGGGTCGTAATATTTCGACGCTTCCAACGCCATCGTCAGGTTCGGTGCCTGAGTGCGAGAGACGTAGTCAGAGA
TGACCTTTGTCCTCTGCTGTTTCAGAGTTTCGACAGGGACGTCGCAAGAGAGCGAGCAAGTGAATGTGCTGCACGCAAGA
CAAATCAGGAGCCAAGAAGTGAGATTCATCTTGCCTCTATTACTTGCTTCACTAAAAATATTTTATGGTCTTGAAATATT
TTCTTCTTGTTTGCATCCAAAGAAAAATAGAAAGTGAAGCTCCTTGCGGAAAGCGCATACGTCCAACGAACAGAACATTT
CATGACAGAACAAGTTCAAAATATTTTGTTCAAAATATTTTTATGACCAACCAAAGAAATCTGTCGCTCCGTCGAACGCG
TCGTACAAAGAACCGAGGAGAGAAGCAGAATCCCCTTCTTCCGAAGTTCCAAAAAATCCAGCTTGTTCCATGTCTTCCGC
CGCAGAACTAAAAGCGGAAGCAAGACTTTCAGAAACACCAGAGAAGAGACTCGAGAGCGCTTCTCCTGTTTTTTCTGCGA
CTTGTGCGGCAGCAGTTCCGACCTCTGAAAATCCAGGAACGAGCGACTTTGCAAAGTCCGTGACTCCCACCATCGTCGCA
AAACTTGCAGAAACTCTCGAAAGATAGATGGGGTCAAACAGCGCCAAAAACATCAAACCAGAGTCTGAAATTTTCTGTCT
GTCGAGAGCGTCTTCCGTCAAAAGATTGCGGTAGTACCAAGACTGTTCGTATCTTTTTTGCCACTCAGAGTACGCCATTC
TCAGCGTTGAAATTCCCTCAGCTCCAAACAGAGCGTCGTTCAAAACTCCTTTGAACCCGGCGGACAGAAGCGCATCGTAC
GCTTTGAAAGAAATCTCATCGTTTGTTTCTCCTGGAGAAAAGAGTCCTTTCAAAATTTCTGACAAGTTTTCATCCTTTGT
TTTTGTCAAAGTTTGGAGAATGTCCACAGTGCGATACTTTGAGTTTGTGGCTGCTTCTTGCCTTGCCTTTTTCTGAATGT
CTGCCGCTTTGCATGCAGTGGACCCAGGATTTGAAGCGCATTGTATCTCGATGAACAAAAGAATCGCTCTTGTGACATCG
TCGGTGCCGTCGTCAGCTTGTGCCACTCTAGCGTACGACAGGGGAAGTGTTTGCAAAACACCAGACACGAAAGAGAGAGG
AGCCTTCACCAAAAATCCGACCAAGGGATTGGCGGCAGCGACAGAGCCGGATATGAGATTCGGCGCCTTGTCCTGGACGA
ACTTGTAAACTTTCGGAAAGAGAGAGTCAGAGAAACAAGCGTCGTATTTCTGGTCCCTCTCTCCCGTTTGCATCGATATC
GCAAATCCTCTGGAAGCGTCCCAGTCGTACTCTGACGTGTTATAGCAAACGAGCGCAGCTTTGTCGAGTTGAAGTTTGTC
AAAAAGTTGTTCCGTCTCTTGTTTCTCTTGTTCAAACGCAGAAAGAGCGTCAGAAAGGAGAGTTGTCTCTGCTTCGCTGA
AAGCGATAGAGTCAATGTTTGTGGAATAAGTCAAGGACAAAAGGACGTCGAAAATTTGTCCAAACTTTGAAGAAGCGAGA
GCGTTCGAAACAAAAGCAGAGAGTTGGGTTTTTAGAACCTGCGCTTGTTGCGTTGAGAGTTTTGAAGAAAGAGCTTGTCG
TTGTTGAACCTGAAGTTCCGTCTTTGTTGCAAGAACTCTCTGTTGTTTCGCGAATACAGAGCTTCTCATCTCTTTGGTTG
CTTCTCCCAAAATTTGAGAACTTTCTGTCCTTGTTTGTTCCTGAAGAGTTTCGACCTTTTCGAACTGGATGACAGGTTTG
GCAAAGTCTGAAATTTTTGAGTCAAGAACTCCCTTTGATGTTCTATCAAGTTGTTCCAAATAGTTTTCCCACGCCCTGTC
CAGCTCAGAGAGAAGAGCTCTCTGTTTTTCGAGGATGCGAATCTGCGAGTCTCGAAGAGAAGACAAAAGTTTTGAATTTT
CTAATATTTTTTGGGAGAGTTGGTCTCTAACGAGTTTTGCGTCGAGTTCTGGATTTTGAGATGGAGCTGAAAGGACGGCG
TCCGCTCTTCCAAGAGACAGAAGTGCATCGATGTCTTCGAGCTTCTGCTTCATCGTTTCTTGTATTCTCTCTGCGTCGTT
CTTCCGAACCACGAATTCTCTGTACTGTTTGTTTTGAATACTTTTCTGGATGTCTTCTGCCTTTTTTCGTAAAACAAAAG
TGTCTTGCTCGAACATGGCGTCTGTATATCCCGCTTTTGACGCTCTCTCTAAAATTCCCTCTTTCTCTGCGCTGAATTTG
AGCGAAGCGTTCAGTGCCTCTGAGAGGTCTATCTTTTTTCCAGAGACGTCAAAGATGTCTCCAAGTTCCAGGATGTTTGT
GGTTTGAGTTTCTTTGGTTGGAGTTTCCACTGTGATGTCAAATCCATCCACAGATAAAAGGGTGAAGAGAGGACCCTGTT
TTTCGTCGTACACAACAGCGCCTGGGGCGAACCTCAAAAGACTGTTTTGAAACTGTGGGAAGAATTTATCCATTGAAATC
TTTTCCCAGAATTCGTTCGGACCTTGTTCATACAAAGTCTTTGCAGTGTCTTGCGCTGCTGCATAAAAAAGTCCAAAGGT
TGAAAAGTCGAGCTTTTCTATCAACGACGCCGTCGCTCTCCTGTCGAATGCGTTCTGAACGGCAGAAGACACAGAGTTGA
CCGCGACAGAGCCAAAGAACCATTCAGAGATTTGGTAGTATGAAAGGTCCCATTCCAGTTGCTGACCGAACCACTTCTCG
AGAGCTTGCTTCAAAAACTTGAACTGGAATTTCTTGGAGAATGTAGGATATTTTTTGAGAATGTAGGATATTTTTTGAGG
GACAGCGAGTTTTTTTATCCAAAGCCACGCGAACCAAAAGGGTAGCGCTCTCGTTTCCAGAGCGCTCAACCCGTACTGCG
CAACAAGCTCTGGAGTGAACCATTGTTTTGCGAAAGAGAGCACAGAGTCGAGAGAGGTCAGTCCAGTTTTTCGTATCGCA
GAGAAAAACAGAGAAACAGCAGAAAGGGAGCCGCTCGCTGTTGTGCTCAAAAGACTCGAGATTTTTTCCGGTGTCATGAA
TTGGAACATGTCGAAACCCACGAATTTGAGAACGTACTGGGAACAAGCCAAAACAGCCAAAAGACCGACCAATGAATATT
TGTACCTCAACGCAAGCTTTGCCGGAGCGGTGGCGATTCTGGACAGCTGCTTTGCGATCGCAGACATTATTTCCGTGTTG
TGTTTTTTCTGTTGCTTCAAAACACCACGAAAGTCCTTGTTTGTTTTGTAACTTTGCGTGAGCGTTTCAAGCTCCCTTTC
GTATTCGAGATTGTTCTTGAGGGTTTGGTACGCCATCTCCTGCACAAGGTCTCCAGTGTCTTGGTATTTTATCCAAAAAT
CTCTGGCATTTGTTGCGAGGTCGGTGACAGCAGAGACAGCAGTTCTCGAGACGATGGTGCCAAAAAGGTCCAAACTTCCC
TGTCCCACAGAAAGGGAAGTTTGCAAACTTCCTGTATACGAAAGGGCTCCATTCATCAGGTCGAGAGAAGCTGAAATTCC
GTCTTTCGTCACGTTCGAGACTTCTGACAAAAGAAAGAGTCTCACATCGTACCCTTGAATGACTTGGTTCAGCCCAGAAA
CAACGAGGCCCTGAGACGCGGCAGGAACAATAAAACTTAGAATTTTGAGAGCAGCCAATTCCGGAGATTGTTGGAGTCTT
TCGACCCATGTTTCTGGGAGGAATCTCCCAACAACTTTGAGACAAAAATCTGGGTCAGAGACGAACAGAGCGTATCCAAG
ACCTGTTGTGGCTGCTGCAAAGGTCAACAAAATTCCTTTCTTGACGAGCGCCCTTTTGAGAGCCGACTCTTTCTCTTGTT
GTTCTAACCAAAGAAAATACTGGTTGATGTATGCCTGAGAATACACTGGAACAATTCCAGAAACTTGCTGTACTTCTGGA
GCGGTAAACTCTCTCAAAATTTGCATCTTTGCCCTCGAAGAGCTCGAAGCACTTTGGCTGAACAACGTCTCAATGTTTTT
TGTTGCCATCATTCCTGCCGATGGAGACAACAGTCTCGACTCGTTCAGAACGTATTTTGAAAGTGTCAGCTTCTTCTGCT
GAGGGAACGACGCTTTCCTCGCTTCGAGATCTTTTCGGAGAGATGCGAGAGGAACTTGCTTGAACGCTCTGCTCGAGAAC
TTTGGAGCTTTTTTGCGAGTGCCTGCAAACTCCATTTACTTGTGAGATTCAAAACAAATGCTCTGAGGAATGTGTGTTCC
CTTGACGTTTGAAAGAAGAGGCGAGTTCTTCTTTGGAGGAGGAAGAGGTCGCTTTTCTGGGAGAGATTGGTATTGACCCA
TATTTCTTTCTGCAAATATTTTCATGAGAAAATATTTATGTTTGCTTCATAAATTCGTGCTCAAGGACATCTTTTATTGT
CGGTCTCTTTCGTGGCTTTCTTTGGAACAACCAAAAGAAAAAATCCCTTTCGCGACGAGGAATCTCAAGAAGGAACCTTT
TCGACTTTATCGTTTCGAGAAGTTCTGGAACATTTCTCCCAGAAAAGGGAAAGAAAGAAAAGAGCATGCAAAAAAAGACG
ACGCCCGCGCTCCAAACGTCACATGCGACTGCATCTCTCTCCTCTGGTTTCTCTCCGTAAACTTCTGGAGCACAGTATTC
GAAAGACCCTCTCGCGGTCGGCTTTTGTCCTTTTGCGGTTGAGAAGCCCCAGTCTATGAGTTTCAAACCTTTTTTGTGAA

CCATCACGTTTTCGCACTTGATATCGCAATGGGCAATTCCAAGCCCGTGGACAAACAAAAGAGCGTTGCAAATTTGGCGA
AACCACTCTTTCGCCTTTTCTTGTTCGACCGGCTTCCTTTGGACAATTTCGAATATGGAGATGCACCCGAGATATTCCAT
CACCGTCCAAGAGTGCCCGCCGAGAGAGATTTTTTCAATCACCTTGCAAATGTTTGGGTGTTCGGGAATTTCTGGATCGG
AAGTCCCATATTTCACAGCATATCTTTTTTCTTCTTTTTTGTAGAGCCAAACCTGGCTCTCTGAACCAGAGCCGACGATG
TGCTTCCTCTGATAATCACCAAGGACTTCTGGAAAAAAAGTCTTCATGTTTGTTTCTTGACGTGTAATGAGTAAAGTTAT
CAATCTTGAACCTGTTCCAAAGACAGAGGAAGGTTTCCCCTTTGCTTTTCAACATCCACAATACGCTGGAATGAAATGGT
TTTGTTCAAGAGACGAACAACACAAAATCACGAGTGTAACGCTTCTGCCGGACATGACGAAAATGGTGAATTTTTTGGAA
GACCTTGACCAGGCAAGAAAAATTCGCAGAGAGCTCAGAAGAGAGGGCTGGGTTGCGATTCAACCAAACTTTTCGATCAC
GTTGTCAGACGACTAAATATTTTATTCAAAATATTTTATGCTGAAAGGGCTTCGTTCATGTTCTTTGTCATCCGGAGAAG
ACGCTGAGGATTGGAGATTGCGATCTGTCTCGAAATTTCGTTGCGAGTGATTTCTGCGAGGTCGGAGAAACTGTACTGTG
AAAACCTTCCCTGGACCTGATTATAGACCTTGTCTCTCGTGAACTTGATGATGTTGTGCTCGTCTTTGGGAAGGATGGTG
CTCTCTTTTGAAATGTACCTCTGTCGGTAGAGGGAGTAGAGATAACTGTTAAGCCTCTCGATGTCTTCCTGAACTTCCGT
AATTTCCTTCTTTCTCTCTGGGAACATTTGAAGCAGCTCTTCGGAATCTCTCTTTGACTCGAGCTTCGAAAGGTTGAGGT
AGCGAAGGGAAAGGCTCGGCTCGTTTCCCCTAATGTTTCTCTTTCTTGTGTATTCGGCGCTCACAACCTTTGAGTACGTC
CCGTTTTGGAGAGAGAAAAGGATTCCGTTCTTTCCTTGTGGGGGATTTTGGGCAAGTTCTGCAAGATTTTCATCGAGATT
CACAGCTTCGGGATATGTGATGTTTGGATGTTCCAGGATATGTTCTGCCTCTTCGAGAGTTTCTCCGTTCGAACGGAGAG
TTTGGACGTGCAAAAGGCGAGGCGTTTCAAAGTTTGAAACAATTCTGTTTTCTGGGTGTTGAAGCAAGAAAACATAAACA
AAGTTCTTGTCGAGAACAGAAGAGAGTTCGCTCCTCGGGACACAGTCGTCAAACAGCTCACCAAAGTTTTTCTTGCTCGA
CCACCTTCTGTTTGTACAGTCGATGAGACGATGGGATGAAATGTACCATCTTTCGTTGTTCCAGAAAACTCGAAGAACGG
TTCCCTCTTCGGACCAATGAGCGCTTTTGATAAATTCGTTGCCTGTCGGAACCTCGGAAATTTCCGGAGTGAAAGGGAAG
CTTCTGCAAACCTCAATGTACTCTGGAGCGCCCCAGTCGGAAGTGCTATTCCTTTCCAAAACAAGGCCTCGACACCTTTG
AAGAGAGGGGTGCGGAGGTTCGTCGATGTAATGAACGAGAAGCATTCCATCGACTTCGTCAAAAACTTTTACCTGCGACA
CAAGAGAATCAGGGATCGAGAGAAACTCGAGAAGGGAGTCGTCAAGTTTCTCCTTTGTCGAAGCATGTTTTTTCCTTCTT
TCCGCGTTCTCCTTCTCATCGAGAGCTCGAAGTTGTTCAACGTTGAGAAGAGGGACTTGCTCGGATGTTTTTCCAGAAGA
CATTTTGTTCTGAGTGCTCACTCTGTTTAATCCACAAGAGTAAGGATGAAGGTTCGTGTCGAAAAAGTTTCTCCAGATGC
GGGAGTTTTTTGTGAAGGCGCTCTGGAACCATGTCAAAAAAGTTGGGCAACAAGAGGTTATTTCGCCCCAGATTCGAGAC
CGAGTCCAGAGCTCGGGTTTCCGCAATCCTGGCCTTTGACGAGAGTTTGGGAAGGTGTCGGTTTGGGAGGAGTCGGAGCA
AACATCTACAGTTGTAGTTTCGCAGAAGACGGAACTGCGAGATATCCCCTCAGCGGAAGAACAGTCCCTCTTTCTCCAAC
GCTCTACGCGAATCAAGGGTGCAATGCGTACAGTTACAAACCCTGGCCTTTCACAAACAGAGTCACAACGGAAAACAGGC
AGATTGTCGCTCCAGCGGAATATCCGGGAGCGCTCATGTGTTTGAGGCAATACTAAAAAATATTTCTTTCCAAATATTTT
GTTGAATGGAAAATTTGGAGAAACATTTGCTGTTTTTTCTTTCACATCAATATGTCCAAAATTTCACCTCTTTGCGCTGA
AGCAGTGTTTCCCGAGTTCGAATTTGAGCAAGAGCTCCAGCATCTTCCTGGACACTCTTGCGTCTCTCGTGGGAAGTTTG
GAGAAAAAGCTGTCATCGTAAAGTCTGGAAGCGATGCCACAGACAGCTATGACGAGTCTGTTGCGTACTCTGTCATCATG
AGCCACCCGCACGAAAATCTTCTCGAGCCTCTGGGTATGGGAAGCAACGGCAAATGGTTTCACATCGCCCTCCCAGACAT
GGGGATGGACCTCTCTGACTTTATTTGGAATCATGGGTCGTACACTGCAAGCCCAGAGGGTTGTAGAAACATTTTCACAA
AGATTGTCAAGGGCCTCCACCATCTTCACAAGCTCGGCATGGTCCACACAGACATCAAACCAGAGAATGTATTTGTCGAC
AGACAGGGAAATGTCAAGATCGGGGATTTCGGAAGCGCGCACTTTCTCAACGACGGCGTTGTGTACGCTGACTACGAGGT
TACTACGCTCTGGTATCGTCCTCTCGACATCATCTTGCGGAGAGGAAAGCCTTTCACCGAAGCGCTCGACATCTGGAGTC
TTGGGTGTTTGCTGGCTTGTATGTGGCTTGGGAAGCCATTGTTTCAGCAAAGCAACGAAGCTGGGATGGTCAAAGCCATA
ACTGGGACGCTCGGCGGTCTCAGCGACTATGAAAAAAGAGAGCTTCTCCCGAGCAAGTACGGCGCCTTTTGCAGAAAGGA
TAGAAGACCCAAGCTCACTCTTGGCGATGAAGGAGGTTTTTGGACTTGCCTTTGCTCCAACATGCTCTACTACACAGAGG
CTGATAGACCTACAACGACCGACATCCTGGAAATTCTTGACCAGTAAATATTTTTAAAAGTAAAATATTTTACCTCTCAT
GATGTGGAAGAACTCGAGATGTTCGTCTGGGAGAAGTTGTGCTTTGTTTCCGAAAAGAGGGGAAAATATATTAGCTTGGG
TATTCTTTCAGGTCGGACACATAATCTGAAAGAAACACTGTATTTGGTACAGCTGGAAAAGGACCACAAAATTTGAAAAG
GAGAGAAGGCGCTCAAAACATGCCGGAGAGCACCCAAAAAGAGAAAGGTTTTGTCCCTCTCGAATAAATATTTTTATAAA
AATATTTTATTACCAGTCGCCATCGGAAAACTGCGACCAAACGTCAAACTGCTCTTGGGAAACATATCCACCACCGACAA
ACTCTTTGTGAAGCTCTTGGCGAAGCTCTTCAAAATTTTCGTGGATGAATTTGATGTAGTTTGTTCTGTCCTCTGTCGTT
TCACTGTCACCCATGCCGCAGATGATGGAACTTTGGAGTTCGACGTCCTTGATGTTCCGAATTCGAGAGTTCATACGAGC
GCTGAAATTCGCTCTCACCTGTTCTCTGAAACCGATCCGGATGTTCGCTTCAAAGTAGCTCAGAGAGTTCACAAGCCTTG
AGCAGTGCCCTGAACTGCACGTGGCCGCCATGTCCACAAGTTCTTCCAAAAGTCTCTGGGCCATCTGTTTGGAAGAGATG
GCGTCTTTCTTTTCTATCCTCTTCCAGATGATGACAAACACATCAGAGAGTTGGAGATCATAGTCTGTGAACGTCGCAGT
GTCGACAGAGATTCTGTCGAGAGCGGCGAGGGCCTTCTTTTGGTCTTCGTCCTTTAGCGTCTCCGTTACAAGGCGAACGA
CCTTTGCGTGGACAGAGGGGAAGTCAGAACAATCTGGTGTTTCCTCTTCCATACATTTTTCCAAAAACTCCATGACAGAT
TCGTTCAGGGACGTAGTGTGGACGTTTTGTTTGTCTGTGTAGACCGTGGTCATTCCCATTTTCGACTGGGCGATTATGTT
GAGCACTGTCTGCGCTCTGGTGCGGTATTTCGCCGTTCCAAGCCTCAAGAGCACATCGCAAGCGTCTGCTTGGAAATTTT
CATTCCCGAGACCGAAATTCTCTGAAATCTGAGCCAACCATTCCATAATTTCTGCCTTTTTCTCTTCGTCAACGATGACC
AACTGGAGAAGGCACTGCGCAGACAGAACGTTGTCTGGGATGTCGTTGTGTTCCTTCACAGAGAAGAAAGATTCTTGGAT
GTCTTTGATGAATTCTGCTGGGGCAGTGGCCCACAATCTCTCAATGTTGCAAAGAGATTTTAGGCATCCAGTTTTGCTGA
ACTCTGCCGCGAACTTGTACCGATAGGGGCTGGAAACTTGGAGGTCTTTTGTGATGTCCATTATCGTGCTTCTGGCGGTT
TCGATGTGCTCTTCTTCCTCTGAAAAAACAAGAAATTTCAGAGCTTCGAGCCTGTGGGAGAGCAACATTTTGCAACATTT
GGAAAGTTTAGAGAAAAGTTCATAACATACCTCGAAATGTCCCAGATTATAGAGACAAACGACGTTCAGCAGCCTCTCGA
AAGAGTCGATAAAAACAGACTCTGAGCAACCACAAAGAAAGGAAACTTCTAAATTTTTCTTTTTTCCTTCCAAATATTTT
AGAGAATCTCTCGAAGCTGCGAGGCAATCCGCTCCCACGTCGCTTCTCTCGCTTTTGTAGATCGCTTCGAGAGCTTGAAT
TCTCGTTCTCTGCGGAAGCATGGAATTTCTGCAAATTTTTTGTATTGCCTGGGCTGCTTCCTGCCCTTGTTCAACATCAT
CTGAAGCCAGCCTAAAAATAAGGCTCGCGAGTTTCGCGTTTTTCTTGTTCATCCCTGTTAACTTGTCCTTTTACAAAGGT
CTTAAGGATGTTCTGGAAGTGGTCCTCTGTTTTGACAAACAAAGGAAACGTCGACATGAGAGACCTGGACAAAAGAATTC
ACAAGATTATTTTGGACGGAGGGAAAGAGGCAGAATTTTCAATGGTTCGAGAGTTTTGTTTCGAACCGTACTACGTGGTT
TTTGAAAACAGAGGGAAAGCCTTTTTTTCGTACGTCACAAGATTCACAAGATTTTTCTTGGAAGGAGGAAGGGAAGTTTT
CGCTCTCGAACTCAAAGAAGGAGATTTCATACAGGGAAAGGATGGACCGATACTGGTCAAAAAAAGAGAAAAACTTCAAA
GTGAAGTCGAGGTTTTCAAGGTTCTTCTCAAGGAAGATTTTGCATTCTTTGTTGACAGTGTGAGAGTGAAAAAGTCATGA
GCAAGTACGAGTCGCTTCCGACGTTTTGCGAGCCGCTCGAGTGGTTCAGAGAAAAGGAAAGAGCTTCCTTCTTCATTCTG

CCGAAGAAGAGCTACATTTTCAACGTCGAATTTGGGAAGAAAAAGATTGTAGGAAAGTTTGCTGATGGAACGAACAAGAA
GTTTTCCATCTCAAAGAAAACGGACGAAACGAGGCTGTACCTCGACTCTTGGTCTTTCATGGACGGCTCTTTAAAAATTC
TCATCGAGAAACGGGAAGATGGCACTTGGATGGGAAGAGTGAAGCTCGGAAAGGAAAAAGTCCATTGGTACGTTGTCGAA
TAAATATTTTTTGTATAAAATATTTTATACTGTTGACCACTCTGCGAGACGGAGACATATCTTCTCTTTCGTTCCGCTGC
TCGGCACTCCCACTTTCTTTGCGATATCTCTGAGCTCTACGAGAGTGTACTTCATACACTCTCTCGCATCGATGAGATTT
TCAGAACCTTTTTGTGGTGTTCCGGTGCCTGTTCCCAGCTTCTTCCTTGGAGCGCTTCTTCCCTTTTTGTAGCAACACTT
TACTCCAGACTTCATCTTTCTTATTTCCGAACCGTCAACGCACCCCTCCTTTCCACTCCTGTCGATGGGTCTCCTTGCTG
AAGGACATCCAGGATACGGATTCTTTCCTCTTGGTATTCTTTTTTCCTTGAGTTCTCCTCCTTCTCCGACTCTCGTCGGT
CTCTCTATGGAAGCGGTGGATGACGACATCTCTTGAAGCCGTACCGGCACCACAGAAAGACCGTTGCTTTTGAGCCATGT
TTCAACGGCTTTCTTTCTCTTTTTCCCTATCTCTTCCGTTTCGCTTGAGGTGTTCCAAGAAACGCGAACGCCCTCGTAGA
TTCCCTTTCTCAGTTCATCAATGAATTTTTTCCTTGTTTGTTCGCTGCCAGATATTTTGGTCTCTTGTTTTTTGAACAAG
AGACGAGGAAGAGTTTTCCTGCCTGCCTCTGCGGCAGTGGCTTCGAAAGGGACAATGTACCCTTCTTTCGGTTTGAAACA
TTCGACATCTTTGTCCCCAAACAACTTGCAATCTACTGCCGCTTCCTTCAGCGCAAGAAGGAATCCTTCGGCAACCTTTG
CTTTTGGTTGGACGATTTCGTCATAAATTTTATCATCCACTGTCTGCTTCCCTCCTGACCGCCTTGCGCGATACAGCCAA
ACCTGCACTTCTCTCTCTTTTTCTGGAAGGTCTTTGTGGGAGCAAAACCTCACAGCTCTCCCTATCGCCTGGCTGATTGT
GGCTTGGTTAAAGAACGGATCGATGATGTGGACATGTCTCACCCTTTTGAGAGTCACTCCCTCTGAGATGCTCTTGGAAC
CTATCATCACTTGGATGACGGAGCCATCCGCGTTTTTCTCTCTGTTGTACGCCTTCACAATCCTTTCTCTTTCTTCTTTT
GTTTCGTCGCCAGTAAAAAGGGCGTACTTTGGTTTTTTGTTCTTTGTTCCGTACTTGGCGAAACCGTTGGCTTCGAGCGC
CTTGGCGATGCTTGCGGCTCCTCCATAGTTTACAAAGTTTGAGTAAACAAACGCCGGTGTCGGATCTTTTTCTAGTTTTC
TTAAAAGTGTGGCCAACTTGCAGCTGTATTTTCCGAGTTTGGGCATCTTCCACATTTCCTTGGTTTCTGATTTCTGGCCA
TTGCTCCCACAAGAACCGTTCGGATACACAAGGTTCGCGGCTTGCCTCTCTGCTTTATAGATGCTGTTGTTGTACTCGAG
ATTCTTCTTCCTGAGCTCCTTTGGTGCGGTCTCGTACTTGTCGTAAGCTTTTTCCTGAAATTCTGACATGTTGCAGACAA
GAACTTTGGGTCCCTTGACTTTGGGGTACGCCTTTGGGTCAGCACCTCGAACGTAAGAGACGAGTCCCTTTATTCTCTCC
TTGAATTCGTCCATGTTTCCAGCGATGACATCTCTCGTTCCTGGCTCGTAAGCAAGATATCTCTTTTTAAAGCTCGAGAG
GATGAAGGGGTCGTCTCTGAACTTGAGGATGTTGAGAGTTTTGGCGATCTCAATCTCGTCGTCAAAGATTGGAGTGGCAG
ACAACAGGACAATTTTGAGCTTTTTCGAAGAGGTTTGGAGTTCCTTCATCAGCGCTTCGTACCAAAGCTTGCCGAGGATA
TTTTGCACTTCGTCGATGATAAGCAGTCCATAATCCTTCATGTCCAGCTTTCGCTTTTTCGAGTCAAGGTAAAACTTTTG
GTAAGACATCACATCGTATCTTTTGGATATTTTCTTCTTGGCTTCAGACACCACCCTTTTCGCTCTTGGAGACTCTGGTT
TCTCTCTGATTACACTTCTGTCCTTGTCAGAGACATAAAATTCCCCTCCGCACTCTGACATCAACTCTCCGATAAAATTT
GCTTCCAATGACGCGGACAAAAGAACAAGGGTCTTTTTCGTTGCCTTTTTTCCCATGTTGCTCTCTGCGATGCGAATGGC
TTCGCAAGTTTTCCCCGATCCCAGCCCATGAAAAAGAAGAATTTTGTTGTGTCCTGTCTTTGGCCCCATGTACTCTTCGA
TGAAAAGTTGTGGGGGTTGAAGGGTGAATTTCTTTGGACTGCAAAGTTCTTCCATCGTGAGGCTTCCTTCAGACCTCTTG
TATTTTTTCTTCAGAGCTTTCATGTCCGCCATTAACTTGAGATTACAAGAAAAAACTTTCAAAAGTAATATGAGCATCAC
GCACAACTCGGGTCTTTCTTCTTTTCGAGCGAAACCAGAGAACAACAGCGACTCCTTGAGGCAACTTTTGCGGTATCTCG
AAGAACAAACCAAGTCGTCTGCGGAGGTGCTCCGCAACATGACAAAGGCGTTTGAGAAACTTTCGGAAGAAGTGGTGTCT
CTGCAAGACGAAGTTCGAAATGTGAAGGAAGAGGTTATCAATGTCGAGAAAAAGACAAAGACACTTTTGAGTTCGTCGGT
CGGATTCAAAAACAGCATCCAATCTCTTGGCGAGAGAATCTCGAGGCTCGAAGAGCCATCAGATTTTGAAAAAACTCTTG
ACAAGGTGGAGACTGTTTTGGAGGGAATTGTGGAAGAAAAGTTCCCTGAATTTGAAAAGAGACTCGGAGAGATTGAAGAG
GCTCTTGTTCGTCCTGTGGAAGAAAAGCCAAAGCCGACTCGTTCAAGGAAGAAGGACTGAGTATAAATATTTTTGAACAA
AATATTTTTATGTACATGCTGCTCTGGATGAGCCACAATCCTTGCATCCCTCGCAAAAGGCATAGTCTTTGTTTGGATTT
TCCGAGTCGATAAAAATCCACCCGTTCGATGCAGGGCTGAAAGCTCCGACGATTTCACCCTCAATATCTGGATGGAAAAC
CTTTGTTTGTCTGTGAACGTTCGCAGAGAATTTTGAATTTGCAGAGGGAATGGACAGAATTGGCAAGAACGAATTGCACC
CATTGCAACCAAGTCCAGGGACAGAGCAGCTGGAAATTATCTGCTCTGACCAAGGACCAATTTTTCCATAGTTTCCGTTC
TGGTCGACATACCTCACAGAGTACCATGTCGGGATACACCAAAGAGGCTGGTTCTGTTCTGCCTTCCCAAACTGGAGAAG
GGATGGTTTTTCTGTTGGAGTCGGAATTGAGCTGTCAAGTTTGACCTCCTTGAAAGAGCCGTCCTTGTTCTCCCAAGGGG
GTATTTTGGGAGTCCAATCCCTTTCAAATCTGAGGATGCCAAACAGAGCGATGACTCCCACTATCAGCAGAACGACGACG
GCGACCAGGCCGTACCAAAAACCGGGACTCATTACTCTCTGATATTTTCAACAAATACGATCTGCATCCCAAACTCTTCG
TAGATGGCAAACATTATCGCAGGCCAAGGAATTCCGTCTGGTCTCTTTTTTATTTTTGACCAAATTTCTTTCGGCACATC
CTCATGTTTTATTTCTTGGCGACAGCAAGGACAAAAAAGAGTTTCTAATTTTTTCAAGCAGGTTTTGTGGAACTTGTGAC
CACAAGAAAGAATAAATCCGGTTCCTTTTGAAAATTTCTCGAGACACACCGCGCACTCTTCAGAGAAACTTGGAGTTTCA
TCCGTTTTGTGGACAAAACAAAGTTCTTCCGAATTCTTTGTCTTCTTTTTGCATCTCTCTCCGTTCTTGTTCGTTCCTTG
ACATTGAGACATAACTTACAAGTCGCTCGTTGAATTTTTCTTTCTTGAATGGTAATATGGACTATCTCGAGGAAGAAGAC
TTTGCGTACTATGGGGAACAAGAGAACGTCACAACAGAAGACATTTTTTCGCAGTACATGCGAGAGGTCGAAGAAGGGAG
AGAGCAAGAAGCTTATGAAGACATTGTCGGAATCCAGGAACAAGAGCAAGAATTTGCTTACAGCTTCCAACAGATGGTCT
CTTCAGGAAAGTCAGGGGGAAGTTCCAGCGCCGTTTTCGTTGGAAGGAGAAGAATCATCTCTGATGAGGAAAGGTTCGAA
AAGGAGGTGAAAGAAATTCTCGAAGAGGAAGAGTCGGTGACAAGGCCGGGACCCAGGGATAAAACAAAGATTGTCGAGAA
GTTGGAGGAACTTCTCCCAGAGTCGAGATTCTACAACGTGAGAATTTTCACAAGAGCCTATCTCTTTGTCCAAAAGAATG
GCAAAGATTTCACTCCTGAACAATTCAGAAGCTCCCTCAAGGCATACAAAAAAACAAAGAAGCAGATGATAGCAGAGGAT
TTTCTCAGGTATTGTCTCATCGTCGTGGACCATATGTAAATATTTTTTGAATAAAATATTTTATTGTCTCCCAGACTCTG
CTTTTATCATCTGCTCGAGGAAACCTCTCGGATCGGGAAGACCGACATCTTCCAACATCCGCAGACGAATTTCGTCTTTT
TCTTTCTTTTTGAGACGAGGGGCTTTCGGGCGCGGCTCGAGTTTTTTCTTCTTATACCCACAAAATTCTTCTATGGACCT
TTTGACCATGTAATCCAAAAGTTTCGCTTCGAGACCCTTCTTTCTTTCCCTGAGTTCTTTGCTTCTTTCGGAAAGTCGCT
TGAGTTCACCGGAGATGCTCTCTATCTGTTTTATGTAAGATGACATAGTGAGTTTCTTCTTTCTTTTTGGTTCTTTCAAA
GAAAAACTATATGGAACTTTTTGTGAAATTCTTTCTTTGTTTTCTGGGATGGATCGGAAGGACAACCCCTTTGTTCGTGA
AGGATTTTGTCTGCTGGTCTCTCCAGAAAAAAACAACAGCAAAAGTCACAAGAAAGCTCACAAAGGTAAAGTGTTGGATT
TCTGGACAAAAGTACCATATTTTGCTTAAACACAAAAGAGGTCCGTTCAACATGATCGGGAACATTTCTTGCGATGGGAA
AGACCGATCCTTGAGAATGGCAAAATACCTTGGACCAAACAACGACTTTTTTGGTCAAACGGTCACTCCAAACGACCTTG
GCTACGACGAGGTTTCCATTGAAGTTGTCTTCCCAAAACAGAAGATTCTGGTTTTCGAGAGGGATGAAGCCATTTCCCTT
TCATAAAATATTTTTGGACAAAAATATTTATTCAAACTTCTCTCGCCAGAGTCTGACCAGAACGTCTCCGTGACAATCTT
CGGTGTCCTTGCAGAAACAACCGAGAGTCTTCCCAGAAAGCTCTCCAAGTCCGTTCCACAATTTTTCTCGTGCGTGTCTT
TCGTACTTTTGGATACATTCTTCCCTTGGCATCTCTGATATTTTGTAAGGATTCGCCCACACACTTTGAGGTAAATTCCA

ACCTCCCATCGTGCAACGACGTCCGATCCAGATGTCACAGTCCTGGATGACCTCTCCATTCTTCCTTTGGATTTTGACCC
TCGAAGGCTCCCCGAGAGCGGGGCCGTACTTGACAATGACGCAAGGCACTCCCTTTTTCTCTGCGAAAGAAATTGTGGAC
TTTGTTCCTTTGGATGTGCCGTCCCAGAACGCGACCAAGAAAGAGCAGTCGTCAACAATCTTTTCGTTCCTCAGAATATA
GTCCCTCTTTTTTGTTGGGTCTTCTGGTCTATGAACAACAAGAGGAACTTTCTTGTGCCTTGCCCACATCTCTGCAAATT
TGTCTGCCCCTTTCGCTCCACCAGAGACGACTGCTGAAAATTCTTGTTGTTTCCATATTTTTCTCATCTCTCTCGCAAAA
AGCTCCTTATCGCTAAAGTCCCTTCCACCAACAATTCCGATGTTGCTCATATTTTTTATCTGATAGAATAAAAAATCTTA
CGAGTTCAATATCTCAAGACGAGCACATCAAACACTCCGCTTCTGCGGCGACACTCACAGCGACTCCGGGGGCGCTTGTT
CTGAGATAGTACATTCCTGTCTTTGCTCCGTTCTCCCATTGATAGAAATGGTAGGACGTCAACTTTTGGTACGTCGGATT
TCGCATGAAGCAGTTGTGAGAACTCGACTGACAAACGTACCGACTTCTGTCTATGCCGAGTTCGCAGAGCCTTTCCTGAA
AAAGTTCAAAGGCGTTGAGATATTTCTTCTTGATGTGTTCAAGGCGTTCCGTTTTCGTTGGGTCGCTTGTAGAAATTCCT
TGGACTGAACCTTGCCCTTTCAAACATTTGAGCATATTTTGTTTGTCCCAAGCTCCGAGGCCGATCATGTCGTCGACAAA
GTGTCTGTTCACAACGGCAAACGCACCACTCAAAACAGTTCTCTTTCCGATGATGCTCGTGAACGCTTCAAAGGATTCGT
TGTTTCTGAGAATTTGTGCTGTGCTTGCAGTGGGCATCAGAGCGATGAGGAGACTGTTGTAGACACCATCTCTCATAGTC
TCTATCCTCTCTTTGTCCCAGTCAACGCCGCAAAAAGATTCGGGGATTCCCTTTTTCTTTCTGTATTGTTTGACTTGCTC
AATGGTGATGACATCAAAGGGAATTCCTGTCTCTTTGTGGAGAGATTCAGCATCCCAAAGGTCATGAGAAAAGAAACCCT
TGGAAAGAGGAGAACCGGGAAAAGTTTCATAGTATGGTGTTTGGAAGCCCAGAGAAGCCCTTTCTTTCGAGATCTTTCTC
GACTCTGAGATTGCTGCGTGGTACATCGTTTCAAAGATTTGTCTATTCAGGATTTTTGCTTCTTCGTCCTGCCAACAAAT
ATCCATCATCGCAAAAGTGTCGGCAAGTCCCTGCACTCCAATGCCGAGAGGGCGATGTCTCAAGTTCGTCGTCTTGATGG
ATGGAAGTTCGGAAGGATAGTAGTTTCTGTCTATGACGTTGTTGAGAGACCGAACGATGAACCTGACAGAAGACGCAAGA
GAGCCAAAGTCATATGTCCTGGTGTCTTTGTCCACAAAACTGTTGAGACAAACAGAAGAGAGATTGCACGATGCAATCTC
CTCGTCCGAAGTGTTCTCTGTGATCTCTAAACATAAATTCAGAGAATGAGGGATTCCCGTGTGTTTCTGGTTGTTCTTTC
GACAGATGGCGTCTTTGTAGACGATGAAGGGCATGCCCACCTCCGCTTGAGCGACGAGAATGTGATGCCAAAGTTCTTGA
GCTTTCACTGTCGTTCTGGCCTTTCCTTCCCTCTCGTACTTTTCGTACAGTTCCTCAAAATCTTGTCCGTAGATTTCGTC
GAGACCGGGAGCTTCCTTTGGGCAAAAGAGACTCCAATCTCCATCACTTTCCACCCTCTTCATGAAGAGGTCAGATGTCC
AAATGGCGTAGAAAAGTTGTCTCGCTTTTTTGAGGTCATCTCCTCCTGGCTTCTTTGCATCCACAAACGTGAAGATATCA
ATGTGCCAGCAAGCAAGGAAGAAAGTTCCGCTTCCCTTTCGTCTGCCTCCTTGGTCCACATATCCCAGAACAGCGTCTGG
AACACGCAGAAGAGAGACGACCCCAGAAGATTCGCCAAGACCTCCACCAATGTCAGAGTGTCGAACGTTGGATGCAGAAT
ATCCAACGCCTCCGCTTCTCGACGAAATGATGGCCATCTGTTTCCACGTATCGATGATGCCGATGAGATTGTCTGGAATC
TTTCCCAGGAAACAGGAAGAGCCAGAAAATTTCTTGAGACCTGCGTTGAAAAAGATGGGACTCGCCACCGTATAAAGTCC
AAGAGAAAGTCCGTCATAAAACTGCTTGACCTTTTGAACGTCAGGCATCCAAAGCTGAGTGGCGACACGAAGGTACATGT
GCTGAGGTCTCTCAAAAACTCGAAGTTTTCCATCATAGCACTTCTTCTTCAGGAGATAGGATTTTTCAAGGGTGCTGATG
GCCATCCAATCAAACCGGTAGTCCCTTTCGTGGACAACAAACTCGTCCAGTTCTTCGGCATGCGAAGCGACAAAATCATA
GAACCTCTCGTCGAGCACGCCTCTCATCCCTTTCGCCATGTCAGAGAACTTTTCTGGTGTTTCTGACCAGAGCTTGCGAA
TTTCCGCTCTTCCTGCCAAAACTCCCCAGTCGGGATGGATGAAACTCTTGAAAACGCAGACTTCTGAGAAGCTCTTGAAA
AACTCAGAGTCTGTGCACGCATCGATATCTGGAAATCTCTGGGAGATGACGCCTTTATTCACCGAAAGACCCACGGAAAG
CTCGTCAAACAATTCGGAAGCGGAAAGAACGGACATCTTTTATATTTGAATTTCCTTTCGATGAAATATGTCTTTTGTCG
GTTCTCTTTGTCCGGCTTTTCTTCAAAAAAATATATTTGAAGAAACACTTTACCGAAAGCAAATGACTGAACATCTGTTT
CTCGCAGAGACTCCTCAAGGGCATTCCATCAAAGTTCTCATCGAAATTCTCAACAGCTGTAGCGCGAAAGATATTTGCGT
CAAAATTTGCAAAAAGGGAATTTTCCTCCACTACATCGACAACAAGGGAACGATTCTCATCGTCGTCTCTCTCGACAGAA
AGGATTTTAGGAATTTTGACTACAACTGCGATGTCGACACCAACATTGGTGTGAACATCCAGAAATTTCTTGTTCTTTTG
CGTTCCATCAAAAAGAAGGATGTTCTGACTTTGTACATTCCAAAGGACCTTCAGTCGCATCTTCGAGTACAGATTGCTCC
TGTCTCCCTTGGAAAAAAATCTTCGGCAAAGGTTGAAGATTACGGTCTCAAGATCCACAAGATTCCAGGAGAAGAAGTCG
AGGTTCCAGAGGAGGAAGGGTACCATTTTCCAAAGGTCATTCCGAGTTCAGAGTTCCAGAAAGTCTGTAAAAAGATGAAC
CTTCTCGGGAAGACCACCAAAATAAACATGGAGGAATCCAGCTATCTCAAGTTTTCGTCAGACTGCGCAGACGTCATGGA
TGGAGGAATCTCTTTCGGAGAACCATCGAGAAAGGGAGAAAGTTACGAGGCGGAATTCACCACATTTTCTCTCAACCAAC
TTGTAAAAATTCCTGGACTCTCTTCAGATGCCCAATTCTTTCAGCCAAAGCTCCCAAATTATCCTTTTAAAATTTCTCTG
TCTGTTGGAGGCATCGGAAAAGCAGAATTTTTTATCAAGGACAGACAAACCATTGAACACGAAGAGAGCAATTCACGGGT
CTAACCAGCTTAAAAATTTCTTCTTTAAAGAGAAGAAATCACAGGCTTTTTTTCCACTTAAGGCAGATGAACACTATGGA
CTTTCCAGAAATATCAGAGTTTGAAGAGACGACAGACGAATGTTCTCACCCTTCCATCGAAGAAAAGAAGGGAAAGAGAA
TCTGCTTGGAGTGTTTCCAAATTTTGGAAGGAGAAATTTCATACACTGAAAAGGATAGCGCGACGTTTGGCCCTAAAAGA
ACGTGCGCCGGAAGAGTCACAACAAGAGGAGAAGAAAAAAGGAACATTTTCAAAGACCTCGAAAGCGTTCAACTCCCGGA
AAACGTGATGGAAGCAGCCAATGCCATGTACCAAAAAATTATGGAAAAAGAAGGCGGAGAAGCATCCATCAAGAGAGGCA
AAAAAAGAAAGGGAGTCATTATCGTGTGCACGCAGAAGGCGTTCAAACAGTACGGAGAACATCGCTCCGTGTACGACGTC
GCCGAACAATTTGGAGAAACACAGAAGGGAGCCATTTCTTATGGTCTCAAAAAAATATTGCAAAGTCTTTCGCGAGAGCCT
TACAGACTATACGAAACCCGCGGATCTCGTGCGGCGGACAGTGACAAAGTGCGGCATTCCTTTTGAAAAGTACCAAGAAA
TTTACGAGATGTGCCTTTCCGTTCAAGGCAAAACAAGGATGTTGAAATCTGCTGTCCCCCAGTCTGTTGCTTCCGCTGTA
GTTTTTCTTTGGCAGACAATGAACAAAGAAAAGCCTTGGTCGTGCTACATGAACAAAAAAGACTTTGCAAAAACTGTGAG
TCTCAGCGAAGCGACCATCACCAAACACGCAAAGGAGGTTGCGAAAATTCTCGGTCTCGACGACGTCAAAGTATAAATAT
TTTCTTCAAAATATTTATTCGAGGATGGAAAAATTATCCATGCTCTCTAGTATCTTTTGTTCCATCTCGAGAACACGAG
CAACAAGTTCAAAGTTTTTTTCTTTTAGTTGAGAGACTTCATCCAGAGTTTTTTGATGAAGTCCTCTCATCTCAGATAGG
TCCCTCTCGAGTTCTCTTATCGTTTCAACTTTTTGGTCGTTCTCCGAAAGGAGTCTGATAATGACTTGGTCAGTTTCTTC
CATATTATTTTCTTTGTCTACAAAGAAAATTAGAGTATTTGGGAAATAAGTTCGTCGTATGGCTGCTCAAGGAAACTTTG
CGTTTCTACCAAATTCTTACAGAGTTTTCTCATGGCAGAAAATCCTTTCTTTTCATAAAGGTCTCCCTTCCACGCTTCCA
TCAAAAAATTCTGGTTGTTCTGGGGTTTCTCCCCAGACGTTAAAAAAACAAGGAACTCTCTGTGGGATTTGACGACTTTT
TCTGAATCTTCCACGAGCTCTTTGATGTTTTGCAAAAGATGTTCCTTTGCTTTCTGAGGATTTTCAAACGATGAGCAGAG
TTGGTCCGCTTCCTTTCTCGTTTTTTCGAGGATCTTCTTTCCTTTTGAGATGTTTTTGAGAGAGATGTCCTTCATTCCGG
AAAGGAGATTGGGGAGGGCTTCCGCTTCTTCCATGGTTTATTCGGCTGTCACGATATATTTAAAGACTGTGGCTTTTCCT
TGAGCTTTCGAATCGGCCATGTCAAAAACAACTTTTGAATTTTCAAGAATTTTTATTTCTTCCTTGTCCCCTTTTTCTTT
TGCCCATTCGAGAACCTGCGCAAGCACCCACTTTTTTCTGTCAAGTTTCTTTGGCATCTTTGGAGCACCAAGGAAAGCGG
TCTTTGAAGCTGTGGGTACAAACACCACACATTTTTCATCTCCATACTTCATCAGCAACCAAGACATCAAGCACTCTGAA
AGTTTGATGGCGTCCATGTTTGTTCCATCAGAATTGTCTCCGGATTTGCCTTTCTTCCCTTTTCCGAAAGAGGTGCAAAA

CTGTTGCTCAATCACAAAGATGCTGGAACTGTCCCACAACTCTTTTTTTTCTTCCAACAAGGAAAAAAGGTTGAGACGAA
CCTGAACATCCAACTTTTTTTCATCAGAGGATATGTCGTAGACGTCAATGTCGATGCGCTCTCCTCCCAAAAGGATGGAG
CGTTTGACCTCTTCGTACGTGGATGTTCCCTTTGCTCTTTCCTTTTTGTACTTTGTGCCGAAAAATTTCTCTCTCTGTTC
TTTCAAAAAAGATACGTCCCAGTTTTCCACAGACCAAGCAAACGCCTTCCATCCGACGTCGAAAGACGAAACTCTGATGC
TTTCCTTTCCATCGATAAACTCCTCGAAAGACGGGATGGTTTTTAAAGTTCTCGCCTTTGACATTTCTCTATAAAATATT
TTTGCTCAAACAAAAAAATATTATGCTGGCAATGTCCCACAAGCCTCAAACAACATCTCGAGCTTCTTCTCAAACTCCTG
GAGAGTTCCGTTGTTCTCTACAACAACATCCCAAATATCCAAGGAAGACGCTTTCTCTTCCAATTCTGAAAACTCGCGAG
GAACATCTCTCTGAATTTTGACGAGAACGAACCCATTCCGTCGAAGCATTTCAGCCTCTTCTTTCTTTCGTACGTCCGTC
ACAAAAATATTTCCCTTCCTGTTGAGACTTATTTGCTCCTCCACCTGGTCAACAAACAAAAGAGAGTTTTTAGATGTCGC
GTACTCGCCAAGAAGAAGGAGAAGTTTTCTATCTTTTTCTATGGGCATGTCAAAGGTTTCCAAAACTTTATTGTGAGCTT
TGTACAGCAACTGACTAAAACTTAGAATTTTCCCTCCGTGCTTTTTTTGAAGGAAAAAGCACGCCGTGTCCTTCCCGCTT
CTCATGAAAGGACCGAATGCCACCCTCATTGTAATATGGAAAAAGCTGTTTGGTTAAGTTTGTCAGCCATTGTGGGGGCT
TCTGCCGTTCTTTCGGTAAAATGGTATGTTGACACAAAAAACCCGTATTGGCTCTTTGCTGCTCTCGCTCTCGAATTTTC
TCTCGTTTTTTGCCATGTGCACCTGCTGAGTTTGGGAGAGGTGGAAATTCTCTTTGTGATAGCGCGAATGCTCGCTGTTC
TTCTTGTTGTTTTCGCTGCTGTTTTATGGCTCTCTCGTTCAACAAAAAGTTGGAAGTTGTGGTTGGGAGTTTCCCTCGCT
CTTGTTTCAATGACCCTTTTGGGTATCGCATAATATTTTTGAGTTCAAAAATATTATTTATTCGCTGGCCTTGACACGCT
TCACAGCCTTGACGATATTGTCATAGTTGACAGTGTAGCTCGAACCTCCCCTCACCACAACATGAGGCTCGTCCCTCTTG
ACTCGCTCTCCCTCATAGGAGAAAGGAATCTTCTTGCGGCCCTCGGTGTCTTCCACAATCTCGTACCTGTACTTGACGAC
TTCGCTGTCCTTGTACTTTTTGAGAAGGCGGGTGAAGGTCTTCCTTCCAGCCTGGAGAGGAGCAGTGCTGTGAACGATGA
GGTCGTTTCCCTTCAGAAGAGAAACATCCACAGCAGGTTCGATGGATTCAGGAACAATCCGGAAAGAGCGATGACCCTGT
TTGAGAACAGGGGGTTTCTTCTCTCGTCGCTGGGTCTTTTTAGGGGTCGCTTCCACAGTCTCTTCCTCCTCGGCAATCTC
TTCCTTTGCGGGAGCAGCCTTGGGTTTCCGAGGTTTCTTCTCAGCAGGAGGAGCAGCGGTCTTTTTGGGAGCTGCCTCTT
TTTTGGGAGCAGAAGCCTTCTTGGTGGCAGTCTTCTTTTTGGGAGCGGCTTTTTTCTTGGGCTTCTCAACAACAAGTTCC
TCTTCGGACTCTGAGTTGTCGGTAGTCGACTCGTCTTCTGAGAGTTTCGGTTCGGTGGCGCGCTTGGATTTCGACATCTT
AGTGCTCACAAGTCTTTTTCCTTAAGTTCGTATTTTCGGCGCCGAGTATGCGCAAGCGTTCAAAAAGTTTTTATTCTTTT
CAAATGCTGAATTTCTACACTCTGATATTTTCGGACAAGCGTGGTTCTCTGGTTGGAAGTGGAATATACAAAACCCCTGA
CCGCAGAACTTGCAATCTCCCAAAAAAGCCGTTCTTTTTCTACAGGCTTCGCTCCCACAAATTTTCCTCATTACAAACAG
ATTAAAGCAGCAAAATTCGCGGGTAAATGGCGGCTTGGAAGACACTTGACCATCAAAGTTTGATTGAATTTGTTGGACAG
CTCATCAAAGACGCTGCTATCACCGAACAACAAATCTCTCGCGCCCTTCCGTCGGATCGCCCTTCGACCTGTCGCTTTAA
AGCGACCAGAGGAAACGGTGAAATTTGTGGCCAACAATCCGTTGTTCCGTATGGGTTCTGCACCAAGCACAAGTCTTCCG
TCCAAGGAAAACAAGCAAAGGAACTTTTTGAAAAACAACAAGAAGAAAAAGCAAAACAGCCAGAGAAAGAGCCCGCTCCT
CCTGCGCCTGCACAAGAAAAGAAACAGGCAACGACAATCAGGAAGAACGCTTATGGGAACTGGCAGCACTCAGAGACTGG
CATCATTTTCAGGAACCACGACAAAAAGGCTTCCGGTATCCAGAACTCAAAAGGAGACATTTCTCCTTTGACGAAGGAGA
CCATACAGTATTGCGAAAAGAGGGGATGGAGTTACGTCGCGCCAAAGGTCGAAGAAGAAACGGAAGAGGAAGAAACGGAA
GACGACGAAGAATCGGCCGAAGAGGGAGAAGAGTCTGAAGAAGACGAGACGGAAGAGGAAAATGACGAAGAGGAAGAAGG
AGAACAAGACGAGGAAGAGGATACTCCAGAAGAGGACGATTAAAATATTTTGGATAAAATATTTACTGGTGCGATACAAA
GCTCTTTCCACAGGCGGCACAATCCACAAAAGCGGTCGCCATCTCATCACCACTTCTCGTTTGCACAACGAGAGAAGATG
TGTTCTTTGACTTGCAAAACATACAAGCGACAAAGCCGTTCTGCACAACCAAAGGTTTGATGATGAGCTCGATGGCATTT
TCTTCTCTTTCTCTTTCTTCAGAAAAAGTTGAGGAAGAAAATCCAAAGTTTCTTTGGTTGAGGTCTTTGACCGCTCCGGG
TTTCGCGAGAACGTTCAAACACGCCCCGAGAACGTCGTAGTAGTACCACGTCGCAAACTCTAGAAATTTGGCGTTTCTCG
GATCGACCTTGATGCCAAGTTCTTTGCAAATTTTCGTGGTGTTTGTGTTCATCTTCTTCTCGATGGCTTCTGCTTTCTCG
GGAAACACTTCCCTGAGCTTTTCGAGAGTCATCTTTCTTATGGACATCTTGTGAAAAACAAGAAGAGAAAAGTTCTTGTA
ATACTTCGATAAAAAAATATGAAGAAACAGCTCGTTCTTCCGAAAGGACTTGTTGATGTTTTTGGAGGGAGCAATGAAGA
AGCATTGAGGGCTTTTCTTTTGTCTTTGCTCACGAAGAAGAAGGAAGATTTTTCCCTCCCAAACTCTTCTCTCGTTTGGG
ATGAAGCGGAAGAATCTGGACTTTTCCATGGAGGAGATGTGAGGACAATTTCAAAGATATGGTTTGAAATGTGCCTTTTG
AGCTATTCTGAAAAACCTCTCCCTCAAAGTCTAAGATTGGGAGACGTGCCCCTGCGCTTTCTTTGCGTGTTCACAGCAAG
CAACAATTCCAGGTGCTTTGCTTTCAAAACAGAGAAGGGGAAGCTTTCAAAAACACAAAGCAGAGAAGAGGCGCTTGTTT
TCGTATTTTCTCCCTTTCTTCCACAAAATCTGTATCAAGAAATTTCTCTTGGTTTTGGGACCATGTGCGACACAAGCATA
GAACCAAAGTCTGGGGTGCACTCTGGAATCCTCTCAATGTCTTCAGAATTCACAAAAAACATCATCTCGTGTATATCCAA
TCAAGGGACCACTCCAAAAATTTGGCTTGTTGGGATGTCGATGGGAGGAGCGATGGCAAAGGCGAGTTTGTGGCACATTC
GAGCCACGGGATGGAGAGGGGAGGCAGAGATCGTGACTTTTGGTTGTCCGAGGATAGGAAATGCGAAATTTTGTGGATGG
TTAAAGAACAACGCGTCCGCTGATTCCTTCTCTGCCGTGCTTGTGACAAACGACGAAAGGAAAGTGGTCGCTGACCCAGT
GTCCTTGTTTCCTCCGAAAGACAAGGGGTATGAGGATTGTCCCTTCTCGTGGTTTTTGCACAACAAAGTGCTTTTTTGGA
GCGACTGGCTGCGCTCCACTCAGTCCGACCTTGACATCACAATTTCTGGTTGCCTTGCAGGTCTTTTGAATTTTGAAGAT
GTCAAAAAATACGGAATGTCCAACATGGGCATCGGAGAGAGAAACAGAGAATGGGAGATTGCACATAACCCCCAAGCGTA
TTTTGACAACCTATAAAAATATTTTGGATAAAATATTTTATTCAGACAGAGAATTTGAGTTGGAGCCAACGTATTCCGAG
AGGGTCATTTCTTTTCCCGAGTACGTTCAGAATATTTGGTCGGCGCTTTATGGTTTGTTATTATTCTCGAGAATAATAAT
TGGAGCGAAAAGTCGCTTTATGCAGAGGCTGTTGGGGAAGGACAAACCGGTTTGGAAAAACGAGACGGTTTGATAGGCTG
AACTCCGCCAGCACATTTCGGGTCTCCCTTGTGTCTTACGATGACAAAAATAACAACACCTATGATGATGAGGGCGATGA
CTGCGAGGACAATAAGAATAATCATACCCTTTCCGGATTTTTTTCCGACTTTTTTGCCAGACGAAGCACTTTGTTCGATG
CTCCCTGTCGCCGTGGCGGAACCCACAAAATTCGCTTGCATCGCGCAAGAGCCGTTGACGTTTCCTTGTTGTGAGATGTC
GATGCCTCCAGAAATGTCAGAGTTCGTGGCAAGAATCGTCAACCTGTCAATGTCGTTGAGACTGCTGATTTTACAGTGGT
CTTCGATGGACTGGTTGATGTCTTGGCGGATGTCCTGATACGAAGTGTTTGTCGCGATGTCGTTGTTCAAAAAACCGCTC
AACCAAGATCCGGCGTTCTTTGCTTGGGCGGAGTTCTTTGCGAAGAACACAGAGTCGATGGCTGCGTTTTGCGTCGTTGA
GAAAAGACAATCCCCCGTCGCGGAGCAGGTTTGTGTAAAGTTGATGCCTCCTCTGATTGTCGAATCTTGAATGTCTATCG
TCGTTCCACTGGCGATGTTCTGGCATTGGATGTCACAAGTGCCTGTGAAATTTTGAGCGACACTGGAAGATGCGAGTTGT
TCTGCTGTATTTTGCGAGAGAGAAAATCCTGAACCCATCGCGCGTACTTACCAAAGATTTTTCTTTGAGCCAAGAAAAAT
CAGAATTCGAGGGTCATGGACCATTGACCCGCATTTTTGAGTTGTTTACCTTCTGCGTCTACGAGACGAACACAGACGTG
TGGCGCCACTCCGTGCCTCTTATCAAAAAAATCTTTTGGAGAAATTTCTCCCATCCAATGGCTCTCTTTTTTCGAGAGTT
TGACCTCCATGAGTGAAGAACGCCACGGACGCAACACACCTTCCAAAGTCACAACGATGAAAATTTTCTTTTTGCCTTTA
AATCCAGAGAAGCTTGCTGTTGCGAGCATTTCCGTCACATTCGTGCGAGGAAGGAGAACGTCCGCGTGGGTGTTGTTGTT

CTGTTCCACAAAAATTTTATCCATCATCATAGAAAATATTTTAGTCTTGAAAATATTTATTGTTCGTATCTTTGTCTCAT
TTTTCTGATGAAACCCTCTGGAAGTTTCTTCTTTGTCGAGGGGTCAATGAATTTTCCCGTTTTGATGAAGGATTTTATCT
CCCTTTCGACGTCAAAACAATGGAACACCCCTTCGGAATAGCACATGACAATATTCCCCAAAGGAATTCCGAGAGCGTCC
GGATTAGCGCATTTTTCTTGGAGTTTTGGAGGATATGGAAGAGAAGCTCCAGAAGAGAGTTTGTATTGCTTCTTTCCGTC
TCCAGAGAGTTCCTGGACCACCAGAGCCAAAAATTCGCCTTCCAATTTTTTGATGTGTTTGAGGAATTCGCTTTTGTGCT
CTTTGTCCAGAAGGAATTCTGGGGAAAGTTCTTCTTCTGGAACGAGGCCGATGGCACCCATCTCGTACAAACCACTTTTC
AGCTTTTCTTCGAGATACGGAGTCTTTGAAGACAATGTCTTGGATAAAAGACCCAGGACCAGCGCAATGCGGCGGACATA
ATATTTCGTCTCGACACCTTTTGTCTCGAACAAGGCTTTCTCCAAACCACGAATGTCGCGCTTTGGCAATATCTTTTTGC
TCGTCGAGAGAACGGCCTCGCGAACCTGTTCTGACGGTTTTTCCGACAGGAGAATGTACTGGGAGAAAGCATCCACCTTT
GGAAGGGTATCAAACTTCTCTGGTTTCGGAAGCTGCGAAAGCAAAGATTCGGACCAAGCCTTTCGAGTCCTGACAACTTC
CGCAAGAGCATCTGAGCGTTTCCTCAAAAAAGCGCGGAATTTGTTCGCATCAGGACCAAGAAGATGTTCCGGAAACAGTT
CGAGCGCTTTGACACTTGCGATGTCTTCTCCTGGAGTGAACGCTCTGCGGAACACGAGACTCTTTTTTGACACTGAATTC
TTTGCATCGAGAAGAAGCTGAAGTTCAGCCAGTTTTTGGATATATTCTCCCGTTGTTTTTGAACTGACGTCGAGCTGCTT
TTCCAACGACCTGGAGTCTGCTTCGCTCAGATAGTCTCCAAGAATTTCTTTCGCAAACTTTCTGGTCTTTTCATCCCTTG
ACTTTGCCTTTTTCCAAGCAGCAAAAGCAAAGTCTGTCTTTTTCGTGGGGGACTTTCTGGATGCAGAACCTCTTTCCTTC
ACCTCTGCGCCGATGAGAATCGTTCCTCTCTTTTTCGGAGCGATGCCCATGGTCGACGCAAGACGAGTCCTCAGAAGAAC
AGCTTCGTCTCTCGCCCTTTTTTCTTGCTCATCAAGGTCTTCCAAAAACTTTTGTCTTTGCCTTCTGGGGAGAATTTCTG
CTTTTTCTCTCTCTCGTTCGATGCGTTCCATCTCAGAGACAAGGAATTTTCCTGCCTCTGTTCTCGGAGCGAGAGAATCT
CTCGCTCCTTCTGCGGCTTCGAAAATTTCTCTCTCCAACTCGCTTATTTTTTCTCTCTCTTCCCGAGAGAGTTGAAGTCT
TTCCTTTGGATTCGGAAAACGCAATCTTCCTCCTTCCGGAAGTTTTTCTCGAGTCAACAGTTTCTTTTGTTCTTTGACTT
CATCAAGAATTTTTTGAATATCTTCTGACCTCTTTTGCAACACAGCGAAATCTCTGTAGACGAGAGGGATGTCGTAGACC
CTCCTCTTCTTTGACTTTTTTTCGTCCTTCAAAACTCGAGAAATTGTGGGGATGGAAGACCTTGAAATCGGCTTGCTTAT
GCCTTTCTCTGTGGCGTATCTTTTTTGCCACCTCTTCACAAGTTCCACGAACCTGTCGTAAGGCTTCTCTTCGTACTTGT
AGACATCCACATCCTCTTCCAAATGGGTGCCGACAAGTCTGACGGTTCCTATCCTCTTTGCTCCATAGACGGTCCTTTCT
GCTTTTCCGACCATTTCCCGAACTGCACCTCGTCTCTTTGCTGTGGTTTTTGACTTTTTCGTGGATACTCTCTCGGTCAA
AACCTTTCCAACTTTCTTTTGTGCTGGAGGGAGTCCCAAAAGTTTCCGCTCAGCAGGAGTGTATCCGAGCCTGCCGCTCG
CACCTCCTTTTTGTTCTTCCCCACCGGGTTCTGAACCCACTCCCACAGGAACGACGATGACGTTATATCCGAGTTTCTGA
AGTCTCGCGGCTTGTCTTTCCGCTTCTTCCTCGGCGAGAACGAGGTCTTCGTCTTTTTTAGTTCCAGGAATTATGGGCAG
TTCTTCCTCTTTTTCTGCCTCCTGTTCTTCTTCGAGCTCAACTTCGAGCTCCCTCTCAAATCTTTTCATCTCCTCTTCTT
CTTGTTCTGCGAGACCTTCGTCGTCCTCCGTTTCGACGTCCTGTTCAAACTCTTCTTCCGACTCCTCTTGTTCGGAGAAT
TCTCGTTCAGATTCGTTATATTCGTCCGAGTCCATTAATATTTTCTCAGCTTTCAGAAAAATTCTTTCGAAGCATAATGG
GAGAAAGACTTGTAGCAACGATTAGCGCCATCGGTGAGTTTGTCGGAGCTCTCGCTGAGCTTTTCCCGAAAGACAAACCT
CTCGTTGCTTATGATAAGAGGCTCCAAAAAACTGGAGTCAGAAATAAGGTTGCGATGAAGAAGCATTTCCAAGAGTTTGA
AAATTTCTTCCAAGAGAATGGAGAGAACGTGGCGACAGGGAATGTCGATGGCATACCAGAAGACACAAAGATTATGTACG
CGTCAGAGACGTACATCGCCATCGGAAAGATTCTTCGAGACATGGAAAAGGACGACACTGTTGTTGTGTTTGACCATCTG
AGAGCCATTAGAGCGCTCGTTGCTCCGGAAAAGCCCAAGGAGGGGACGGAACACCTTCTCGGTTCCGTCATCTCTGAGGC
GAAACAGATAGGAAAGAACCTCTTGGACAGCGGTAAGATTGACAGGTCAAAACTTTCCGGAAAGCCAGACGCCAAAGCCA
TTGCGGAAATTGCACCTGTCATGCTCAAGGAGTTCCTCGAGAGTGGAGCCATCGAGAGAATGGTCGAGAAGTTTGACGGA
AAGGAACTCGATATGGGCTCTGTTCTTGGGATGATAAAGGGAATGGCTGGAGAAATGCAGTAATAAAATATAACGAAACT
TATATTTTAGGAAGGGAATGGCGGAGCTCAGAAACAGAAGAAAACCACAGGAACAAAAAAAGGAGAAAGTCGATGCTCTC
TGTTTGTCTGGTGGAGGGATAAACGGCATCGCGACTCTCGGGGCGCTCGAATATTTCTCGAGAGTTTTTGACCTTGATTG
TGCCACAAAATTTTGTGGCACATCCATTGGAGCGATAATCTGTCTTTTGCTTCTTTGTGGCTACAAACCAAGAGAGATTT
TGGATAGAGTGCTCTGCGAAGGAGACATCTGTTCTCCACTTTCGAAGCTCGCGTTTTTCACAAACTATGGCTTGAACGAC
TCCACTCCTTTGGAAAAAAAATTGGAAGAGCTCATCGAGCAAAAGTTTGGGTTTGTCCCGTCTCTCCTGGAACTTTTCAC
TATCACCCAAAAAGAATTTGTCTGCGCTTGCGCAAACCTCTCTGCGATGAGGATAGAGTATTTTTCCAAAACGACAATGC
CGAGCATGTCGTGCGTGGAGGCGGTTCTTTTGTCTTGTAACGCTCCAGGTGTTTTTAAAAAGAGAGAGTATCTCGGAAGC
ACCTATACAGACGGAGGAATTTTTGATAACTATCCATTGTCTACGTTCGACGATGGAAAAACAAAAATCCTCGGTATCAA
CATCGGAAGCTCCTGTTTCTCTGGGGACGTTCCAAACTTTTTTGACTACATGCACCGCCTGGCGTCGTTTGCTGCTGTTC
AAAACTCTGAGAAAACTTTCAGCCCCCTTGTGCTGAACATCAAACTCAAGGTCGAAGAGTTTGCTTTCAACCTCTTCATT
CCAAACGAAAGAAAGATTGAGCTGTTTGGAATAGGTCATGTGAAAGCGTCGAAAGCGTACAGAGAGATACTCGAGCTGAG
AGAAACTCTAGAGATGCATTGAGGAAAGAAAAATATTTTGCAAATAAAATATTTACCCGAAACGTATCGTCTTATAGGAT
TTCCTTTGACCGAATAATGTCGGAGGAAACTCAAACTCCAGAAAAGAAATACTCTGTCATCGTCAACCTTTTGCCGAGGA
CGACTCCTTTGGTTTCAAAGAAACCCGACTTTGGAGACCTCGGAGACCTTCATCTCGACATGATCGAAGTGAAGGAGAAA
CTGCGCCCAGGGCTTCCTCTCGTCCCTCCGAAAAGAAAGGAAGAACCAAAGAAAGAGGAAGAAGAAAAGAAAGAGGAAAT
TCTTGCTCCACCAGAACCAGAAAAACAGACGCAAGAAGAGCCGCCTGTCGAGCAACCTGGACTCGTTTTTGAGGAAGAGG
ATGACGACCCTTCTCCGGCAAGCGATTCGGAAGAAGAGCCGGATGCGCCGCAGACAAACGCGCAGAGACCTCCCCCAGTT
GTCGTACCTCCAGTTGTCCTTCCACAAGAAAACCAGGACGATGACGACTCAGAAACATCCTCTGAAGATGAACCAGAACA
ACAGGACCCAGAGGAGATTCTGACAGAGGAGCAAAAGGAAGAGAAAGAAAGGGACGAACTTCTCTGGAAGTTTAAAATTC
TCAAGAAGAAACATCCACAACATTTGGATACAATCCCTGACTTTGACGAGCACAGCGACATCGTCACAATGAGAAGAAAG
TACCAAATGACAGTCAAAGAACTTCATATGGAAAGCAAGCTCTTTGAGTACCGCAAATTTGTGTTTTTCGGCGGGTGTTT
GTTCGAAGGTCTGTGCACAAATCTCCTTGGCGTGGACGTGAAAGGATTCTCAGAAGTCCAAGATATTCACGGTTACGACG
CTCTGCTTGTCGAACTTGGTGAAAAATCCTACACAAACTTCACTTCTTACTTGCCTGTGGAGCTCAGGTTGCTCATCGCC
TTTGCAATGAATGTCGCGATCTTTTACATTTTCAAGACGATGTCCATTCGGGTGACGAGCGAGGACGTGTTCAACATGAT
ATCGCAGGTTGGAGGACAAAAGGTAGCAGATTTCTTGCGTCAGGCAACAGGGAAAGAGCAAAAGAAAGAGACAGAAAGTG
CACCGACAACGAAAAGAAGGATGAGAGGGCCGACCATCAAAGCAGAGGATGTGAGAAAGATGTCTATAGGTAAAGAGGAC
TAAAAATATAAAAATATATTTTTCTAAAATTTTTGACACAGTGCCTTGTCTTTTGTCAAAGGGTTGCCAGAATAGAGGAA
CGCTTTGCATGGAGAGTCTTTGTACGTCGGACAACCTTTGACAACTTTTTTTCCTTCGCTCTGCGCAAAAGCAGCGAACC
TTTCGGCAAGGTCTCTCAGGCGCTCTGGGGACACTCCATGGGTCAAGCCGTAGTGTATCTCTGCATCGGAAAGTTCCGGA
AATTTTATCTTGCAATAGACAAAGAGGTTCGCCCACGTTTCGCAATAGCCTGTGATGAGGCCTGGAACTTTGGAAGCTCT
GGATTCGACGCTTTGTGGACCAAACCAAGGACAATTTTCTTCTGTTTTTGCGAATGCATAGCCTATCAACCCGAAAGTTT
CTGCAAACTTTTTCACAAACCCTTCCTGACTTTTTATATATTTTTTACTAGAAACAACACCATGAGGTTCAAAGAAAGAA

ATTTTCTTTGTCTTTCTGTCTATGACCATGGTGTTTGCGTGTTCTCCGCCTCCGACGATGATAGGAACAACGACGTGGCG
CGTTTTACTCGGCAAAGTTCTCACCTTCTTCATCTCAAGAACGAGCTCTTTCGGATAACAAAAACTATCGCGTTTGACTT
CGACATACCTCCCAAAATATTTTCCATGTTCTGACCATGTGTGGATGCGAGGAACCCATAACAACCCCTCTCTTTCGAGA
GCAAACAAAAGGGAGAAGGTTTTGAGGTAGAGATAGCGAACGCCTTCCTTTTTCGTCGCAAAGGATTCTGACAGAAGCGG
AAGACCGACTCCCACAGTGGATGTAAATTCTCTGAGAGCAGAGAGCTTCGTCTTTGAAGTTTTTACGGTTTGTCCGAGCC
GAGCAGGAAGAGGTAAAGTTTCCAAAGTTTTGATGTGCTCTGGAAGCAGAACGTTTCCTGTGAACGGGTCTTTGTTGTAA
CCTGTTGCCAGCACGCGTTCCAAGTAGTTTTTGTCTGGGCGAGTGTAGCACGTTCTTCCTATTTTAAGAAAAGAGTCTGA
ACTGAGGTCACCAATGTCGTCTCCGTCGACGACAGAAAATCTGTTGACGCAACCAGACTGGACGAATCTCTTTTTCCTGA
CCCTGTCGATGTCAAGAAGGTTGCCTTTCTCTTTGAAAAGTTTGAGGCACTTCTCGCCTTTGACGAACCTTTCCATCTTA
CTTTTTTCTTTTGAACCAAAAGAAAATATTTACTGGGGCTTTCCGATGAATTGGTCAATCCAAGCAGCCATCGACTTGAC
TTCATCTCCGCCGCTCTTTCTTGCGACTTCGACTCCGTTTTTGAAGATGATGAAAGTGGGAAGGGAAGAAATTTTCAGAG
CGTCCTTGACTCTTGCGGATGCGGGGTCAGCAACATGCACCTTGGCAAACGACACGTCCTTTCGCGCGCACGCCGCCGTC
AGTTCATCAAACTTTGGCAACATGGCACGACAGGGTCCGCACCAACTCGCCCAAGCATCAACGACGTAAAATGTGTTTGA
GCGGATAGCTCTAGCAAAGTTGCCGTCGGTGAGTTCTTGAGGAACTCTCTTTTGCGTTTGTTGCTGTTGCGGTGCTTGCC
TCATCTTACAAGTCAAACGCTCCCTCTTGGAAATAAAAAAAGTTTTTATTGTAAAAAAGAATCCCAGAGAAAGTTTTGTT
CCTTCTTTTGTTCTACGAGTTGGAGCAACATGCCTGGCTCTTTTGTGTTGCAGAACTCGAGGATGTGTTTTTCCTTTCTT
GCTCCCAGTTCGATGAAAAAGTTTGCGAGCCCTTTTTGTCTATGTTTCAAAGCCATCGAAAGAGCAAACTCCCAATCGTC
GAAACTGTACCTTTCACAAACAAAAGAGACAAGCGTCGAATCTCCAGAAATTATGGCTTTTTCCAGCATGTTTTTTGCCT
CAGAATCTCCAAGAGGAAATAAAGAAAAAGAGAGCTCTTGCTTTCCCGTGTTTTCTGCTATGTATGCAGCTTCTGGTGTT
CTTGTCCCACAAAAAGTAAGGATTTTGGATGGAATATTCCATCCCTTGTCGATGGTCCAACAAAGACATTTTTCTGCGAA
AGATGTCATGCTCTTTTTTAACTTCGAATAAACTTTACCTTTCCCTTTTTTTCTTCTGGAGGCTGGTTTTTGGCTGTCTT
TTTCTTTTTCTTTTCGTCGGGAAACCTGTCCTCGATGAGCTCTCTGTTTTCCTCGACAATCTGAATGAACGAGTCAAACG
CTTTTTCCATGCTTTCCTTGTGCTTGCCAGAGACTATTGTTTTCGAGCTTCCAAACACCATAAACGTGGTGTGTTTCTTT
GGCTTTCTCCTTTTGATGTATTTATTCTCTCTTTCGTATTTTATGCTTCTTGACCAGCCGTCGCTGTACTTTTCAAAAAC
CATCACCTCATACGCAAAGTCATCGGGGACGGAACTTCTCATTCTGATGTTCACTTGAGTGTTTGTGGTAGGTTCAAACC
TTGAAATTTTGACTCTGTCCTCGTAAGGCTCGGAATTCATGACTGTGTTCAGGACCTCTCTGTCGATGGTGAAGCCAAAC
GTAAAGTCCACATTCGTCATCACCGTCTCGAGAAAAAAGTTTGGGTGCTGTTCGCCTTCGTTCAAAACAAAAGAGTCGGG
TATGTCGGAAATGTATTTCCAAAGGATGTCCACAGTTTTCTTGACTTGTTCTTCCGTCCTGCAACCAGGAATTTTAAAAT
TCCCTCGGAATATCATAATGTGGAGGTTCTTTCCATCCACCGAGAGAATGCAGGTGACCTGATTGAGAAAATACTTTTTT
GTTGTTTGGTTCTTTGCTGCCTCTGGATTAGTGACGATGCCGCGAAAATTGTTGCCGTGTCTGAGGCTCACTATAGAACC
TTCTTCCGCAGACACAGACTTGATGTCTGGAATTCCAGACTTTCTTTTCACCTTATATTCCGCCTCTGTCACAGGAAGTC
CCGCAAAAATGTTCTTGAGGTCGAATGTAACGTTTGTGTACGCCATGACCGTCTTGGTCGAAACCTTGCAATCTGAGAAC
TTCATCTCTTTGCTTCATGAAATAAAAAAACGGCTTTTTATTTCCTTTTCATACGACTTCCACCCTTTTGTGGTGCTTCA
AAAAAAAGTGACAACCTAGAAAAAGACAACAGGCAGCAGCAGAGTTCAATAGAATCACTTCTACGACGTTCGTCTCGTCC
TTTGCTCTGTCTTGTCTTTCGCAAATTCCCATCGCGACGCAGAGACAGAACAAAAAGTATATCGCAGAACCAAACTTTTT
GTGGTTCAAACCAAAGAAGAGTTCCATCCATTCTCTTTGGAACCTCTTTGGTGTCAAAACAACAGAAAGTTTTACGGCTG
CATAAAAGACGCTTGTGAGAGCCAGAGAAAAGCCAAGAAAACTGTGGAATGTTGTGGGAAGAAATAACTCTTCTCTTTGC
TTTTGCTTTCTCGGCAACCAAACAAAAGGAGCAACGAACGTCGAAAAAGAGAGCACATGCAAGGAGACATGTAAGATTTT
TTTGTGTTCAACAAAAGGAACAAAGAGGACGCTGAGACTCAAAAACACAAACGCGAGAGACGCGAAAAGAGGGTGAAGCA
TGGTTGGATTGACCCATTTATCTGCTTTTTCCCACACTAACAAAAACCAGAGGCTTTTCAGAACAACAAATTTTCCGAGG
ATCACGGCTGTGAGAATATCTTCTCTATTCATTTTTTTTATATAAAATTCTCTAAGTAATATGTCACTAAAAAGGGGTTC
TGCCGATTTGGGACAAGACGTTGACGGCTTTTCTCTTGATGCCGGAGCAGCCCTAACTTCAGCATTTGGTACGAGTTGCT
TTCCCCTTCTGGTCGCTGTTGGCGCGATCACTCCTCTGCTCGTTTGGCTTGTACTCTATTTTATTCAGCCAAGGTTCATC
CAGACCAGGAAGGACACGGGTTACGCGCAAGATAATTGGAAGCTCCTCAAATGGACCGCAGTTTTCACGATCGGCATCTG
GCTGATTTTCTGGGCTTTCACCTTCTGTAAAGACTACAAACAACAGACCATCTGTCTGGGTTAGAGAAATTTTTAAGAAT
AAAAATTTCATAGACACGCTCTGCAAACGGAGGCGTACATGTTTCCGACAACTTCCTGTTTTTGGTCGTCGGACAACCTT
TTTGTGAAGGGAGCAGGAACCATAAGTTTTTTCTTTGCGCAAAAGTCACAAGAAGCAAGCAACTTCGATACGGAATCGGC
TTGCGGGATCAAGCTGAGAATTTTGCCCATCTTTTTCTGCTCAAAGTCTCCATCCAATCCAGATACGATGACCCTCTTCC
CGGAACGGACGATGACCAACACTCTCTCTGCAAAGTCTTTCTCTTTGAAAAACTGTGCTTCGTCGATGCCTATAACGTCA
AAATCTGCTATTTCAATGTCCTTGACGAAAAGAACTTTTTGAGACTCGAATGCGCGACTGGCTCCGGAAAGTTCAGAGTT
ATGGGTGGAAAATGCCACAGAACCTTCTGGACCTTGTCTCGTGTCTTTCGACGAGTTGACGTAAAGAACCTTGAAACCGA
TACTGGAGAAGACTCCAAGTCTTCTCCGAAGCTCTGTTGTTTTTCCTGACCTCATCGGTCCACAGATTATTTCGAGACCG
GAATTGGAATACATGGTTTCAAGCTTTCTCTGCTTTGAATAAATTATGGCTGAAAAAAATTTTGTGATTGTCGACAATGC
ATCTTTTCGTGGTTCTCAGCAAGCCACTGCTGTGCTGATGAAGGACATGGTCAAGGGAGTTTTTTTGCCGTTGACGACAG
ACGAGCCTTGTTATTGGTGTAGAGAACCATTTCACACGATGTGTTACGCTGTGCCTCTAAAATACCACAAACACAGGGAA
ACGGGAATTCACGCGGAACGGATGAAGGAACATTTTAGGCTTTACAACCTTCCTTCTGACCACGGAAACGACCACTTTGA
AACGGAAGGGATTTTTTGCGGAAGGGGGTGTGTCAAGGCGTTCATAGAAGACGAGCTTCCAAAGAAGCCCCATTTTTACA
GGAAGTCTCTGGGACTTTTGACCATAATGTGCATGAAAATAGACGGAAGTCTTTCAACAATTATCTCATCCCCTCATTGG
AGACTTTTGAAAAAATGGGGTGGAATTCTGACGATAGAAGAGTTTCGCGCAAATTCATGCCTCAAAAGATACGAAATTTC
CCCAAACACCAAAAGACCGTATATGTATTGTACTGGAAGGTATGTCGAAGAGAGAGACATCTAAAGAAAAATATCCAAAG
ATATTTTTTGTAATGTTTACTTTTGGCTTTGTTCGAACAGCGACCCACGGAGGAATTTTCAGAGACTCTTACAGAGTTTG
GACAGAGAGAAAGGAGTCGCTGCAAAAGATTGTCGAAGGAAAGGAAGGAGACTGTTCCATCCCAAAAATGACGGCTTGGT
TTTCTGCGTTCTATCCTGACTATTCTCATTGTTTCGAAGAATTCCTTCAAAGGGAAGATGCGATCATTTCTCGTCTTTCT
CAAGTTGAGGGCAAAGAGTTTGAAAAACTTGTGGCTCAAGGAAGAACGAATGCAGAGAGGATGGCGGAATTTCTAAGCCA
ACACAACCCTTGGTTTTTGAAAGGACCGGGTGCGTTGATGTTGCAAAGATTTTTTGACATGTTTGTGATGCAAAAGCTCG
GATACCAAGGTTGTCAGGACGAACAAACAAGGCTTTCATTTCAGATAGCGGAAGAATTCGCGAAAGGATTGGTGGAACAA
TTGGGCCTTGTCTAGTGAACAAGAGAATTTGTGTCTTTGTATGGAGCGTGCTCGAAAAGTTCCACGTCGGCTCCTGGAGG
ACCTCCCCACTGAATTTTTCCAGACCACACATCGTTGACCCAGTCAGCTGCCACTCTCATTTTTTCTCTTTTGGTTTGGT
TCGTCTCGTCGAAAAAGATGTATCTGTACTCGAAATCTTCTTCTTTCCCGAGACATCTTTCATGAAGTTTTTTCTTTCCT
TCCAAAGAAATATTCTCTTTGCTTGCGAGACTTGTTATCGTTGTAAATTTTTCTCCTGTCTTTTTGGAGCGCACAGCCGC
GTCCTCTTCGGAAAAGCCAAAGATCGGATGTGACAAACTCAAAGAGAATTTTCCTGGCGGAAACACCACAAATTTTTCTA

AAACTCTCTGAGATGGAAGGAGTAAATCCGCGAAAATTCTCTCTTCAGGCACTCCAGGAACAACTTCCACCCTTTTGCCT
TCCATACGGAACTCGAACCCTTTCTCTGTTGCGCTCCATTCGCTCTCTGGAATCCAAGAACCCATTCTCGGTGGAGGGGA
AACGAGAAACTTGCCTTTCTCTGAAATTCTCCATGACCTCGTATCGAACGGAAACACTCCAAAAAAGTTTTTGACCCAGC
TGTCCTTGCATGGAACGATAAAGAGGTCGATGTCTAAAAGTCCCTCTGACGAAACGACAAATCTCGTCCAATGTGCTTGG
TTGGACAACCTGTTTTCTGCGTCGTAGGCTCCCATATTTCCGTTGATAAAATAGTATCTCTTGCACTCTTTGAGTTTTTG
TGATGGAACCCATACGTAAGGCATCCCTTTCACCCAGTATCCGACAGAGTCAAAGTCGAGAGACATTACACAACATTTTT
ATTGTCTCAATAAAAATCAGCGGCATCCCAAACAACCGTCCCTTTCGACGATACAAATTCTCTCTGAAAAACTTTTGTCA
CACTCTTTGCACTCCCAAAGAGCAAACATTCTCGAGGTTTTCTTCACTTGCATGGGGGTCTGCTCGTTCCTTTTTGACCA
AAGCTTTGCCTTTCCGCTCGAAGCGAGAGAACGACGGAAACACTCTTTACAATCTTGGGTGTTGCAAAGTCTCTTGCACA
TTTTTCAAAAAATATATTTCGTCAAGAATATATTTTCTTTTTTTGAAATCAAGAGAAGAACAAAGGAATGGCTTTTGCTT
TCCAACACTTGTTATGAACACCACAGACAAAAAGTTGGCATGTCTCTCTTGCGGAGTGTCGACCGACCGTCCGAAAGAGT
TTCTGAAACATTGCAAACTTCACGCTCATACACAAAACAGGTATTTTTGTGGATATTGCGAGTCATTTGCTGACTCTTCT
CAAGAATTTTTTGAACATCCCACAAAGAAGCCATGCGTTCTTCACAGCTTTTTTGAGCTCTTCACGAAAGAGGCGTGAGA
ATATACTTTTGTCGACAAAAGTATTGTAATGGACGCTTCGAACTGGAAATTTGAGAGAGAGGTTTTTCCTCAAGAGGAGC
AGCGCGCGCCGAGAGTCACAGAGACAGGAGAGTGCAGAGACGAAAATTCGATGGTCCCTCTTTTGAGGCTGAAAACTCCC
CCTCAAAACCAAACACTTCAAGAGTATTTTGGTCTTGTGCAAAAGGACCTTCCTTGGTACAGCGATGTCGACGCGTTCGG
CACTCCGAAAATCAATGGATGCAACTTTTTGGCTCTGGGTACACAAGTGCCTGAACTTGCACTTTTCCCCAGAAGTCCTT
ACACGGACTATGGAAATCCAAGAGGAACAACAGGACAGGAACTTGTTGGAGTAGCGCAGAGGTCGGGAGCGCAATATCTC
AGCTCTTCGACAAGAACTGGGAAGCAGATATTTCGCTCAAAGCACAGAGGTATTTGTCCTCTTGGGTGGGTTTCCGGACC
GAATGGGTTGTGTCTCCCAGAAAATACAGCTCAAAGAGATGGAATGTTCTACAGCGTTCCGAACGTTTCCGCTCTCTACA
ACTTTTCGAATTGCGGCATACAACAGTGTATGGGAGCCACACAATAAAATATTTTTTGAAATATTTTATATGCCATGTCG
AACAAGACCGAGCCTTTCGAGTTCGGACTCGACGATGTCGCACAGAGTGTCTTTTTTGACAGACGTTCCCTTCTTTTTGC
GTTTGTTTGGGTTGTCCCTTATCAGATGGGCTGCCGAGAGCATGTCTTCGTCTTTGTATCCTTTTGCTTTCATCGCCCGA
TAGTCTATGGACCCTGAAGCGAGAGCCTCTTCTATCTCTTCTGGCTCGAGAGGTTCGAGCTCTTCTTCTGGGAGTTCTCC
CCTTTCTCTCGAAATTTTGGATATCTCCATGAGAATTTGGAACAACAAAGGAATTTTGACAGAGCTTCTGCAAACGACGC
CGAGGCCCTTTTTGCCGCTGAGATTTGTCTTTACGCGGAAGACGCCTGGGTCCTTTGCGAGATAGAGTCCAAGAAGAGAC
TCCGACTCTGTGGCTTCTTTCCGCCTCTTCACCACCTGCTTTTTGATGTCGGCGACAATTTTCTCTTCGAGCTCTCCTGT
CGCCGTCGTCCAGACAAACTTCTCTTCGTCAAACATTCGAGACTTTCCGTTGGGGGCGTATCCTTTCCTTGCGATGTCGT
ACGCAGCGGTGCTGACGAGGTCCTGCGAAAGGAGATGGACGACCGTTTCTTTTCCATCGATGTCCACTCTGTGGATAAAG
TTTTTGTATTTTCCGAGAATGTACTCGAGTTGTTCCAGATGTTTGTCAGAGAGAGGTTCGCCTCTTTGGACGAGAGAGTG
CGCAAGTTCAAAGAGGACGATTCTGACCTTGTAGTCCAAGGATTCAAACTGCTTTTTGAATTTGGCGAGACTGACCTCGC
ACAAACCCCTGACTTTTTCTTTGCTCGCTTTGAAAATTTGAACATCAAACACAGACTCTGTGTCCTCCTTCTTTGTCACA
AGAGGAGACAAAACGTAGAAGATGTCTGCGAAATTCCTCTGCTCTTCTCCAAAAGATTTTACGAGGTAAAAGACATCGTT
CTCTTCTGCGAGATAACAGGGGAACCCGAAACTGTCGAGTATGGTTTCCCTGTCGTCGATGAGATCGCTCAGAACAGCGA
GAAGAAGCCTTTCCTTTCCAAAGGTTTTGCTCAACTTTTCGAAACTTGCGTACGGATTCCTCCCAAAAAAGTCAAGAAGT
TTTCTCTTGATGTCCTCTCTTTCTTTCCCAGAGTAGTACATGTCGTACGTGTCGTCGCGATATTCTTGTTGGGGGTATCC
GTACCTTTCTTCTGGACCCGAACGAGGATTCTCTGGTTTGGCAGAGGAACAACCAAAGTCGCAAACGTCAAAGTCGCAAA
CTTCCGAACCGTCCTCGTCTGCGGGATCCACGTTTCTGGCGTAGTTTAGAGGACAGTCGATGGCGTTTTGCTTCATCAGA
CGAAGAACTTTCGCGGATTTCAAAGCCTTCTTTTCTGCCTTTTTATATGTCAAAATATCTGGTGTCACATCAGGAAGAAA
TTTTCCTCTCTTTGCATTGACGGCGACGTGGCGGTAGACTTCGACGTTTCTTTGGGATTTTGGAAGGTCGGCATGAGAAC
CGAGACGGATGGCTCTTGCGATCGCCTGTTCTATTTGGGGAGATTGCCAATGAGGTGTGAGAACATGGACCTTTCTGACG
TTTTTGAGAGTGACTCCTTGAGAGAGGATTTTGGACGCGATGAGAACTTGTATTTTCTGTCCGTTTTTGTTTTCTGGTGA
AGTGTAGGCTCCAAGAACACTTTGGACTGTGTTTTTGTCCATTCCTCCCTGAAGGACAGCAAAGCGTCTCAAACTTTTTT
CTGGGTTCACTGCACCATTGTTTGCTTGTTTGAGACCAAAGAGTTTCAAGATGATGGCGAGAAGGAGAGCTCCACCAGAA
CGGACGGATGAACTGTAAACAAAGATGAGCTGGTTGGGGTTCTCCAGAATAGAGTCCACGATCTGTTTGAATTTTGAGCT
GTACTCTCCAAGGTTTTCTTTTATCTTTTTTGAGATGGCAGGGGATATGCCATAGTTTCCCTTTGCTCCTTTCTTCGTCG
CAAACCTTTCGAAACCTCTTATGCCGTAGAGGTCAACAGGAGCAACTCCCTCTTGTTCCGGCCATACGAAAAGAGAAGCA
TCAAGGCCGAATCTGTGGAAGCCACCGCCTTTCTTTGACACTTTTCCTTTGACTTCGAGAGTTTCTGTCTCTGCTCTTTC
GACCGCCTTTTGTTGGTATTCGCTCATTGCGGACGGGTAGAGACGAATGTGTTCCGTCCAGGGTCTCGAACTTTCCTTTA
GCGAAGACTTTGTTGCTTTTTTCCAGACAGAGAAATTTTCTGGTTCTACCCAAGGCACAGTGGAACCGATGTCCACTCTC
TTTGTGTCTGCGGCTGCGGCTCTCAAATAAGAGACCCTCCCGCGAAAGGCGTCCTCGAGTTTTTTCTTTCCTTCTGGCAA
AGATAAAAGGCGCTCGAACGCCCTTTCTTCAGGAAGCTGCTTGTCGGAAGGGAGGATGAGGTTCATCACGTATGCGATGT
CCCAGACTTCGTTCAAAATAGGCGTTCCAGTGAGAAGGACAATTTTTGAATCTTTTATTTTATGCAAGAAGGTGTGCATG
AATTTGTACACGTTGGATTTTTGCTCTTTCCCTTGTTTTTGTGGAGCGACTTCGTCTTCTTCTGTTTCTTGTCCAAGATT
TTGCACTTCGTCGATGATGATAAGCCTCCCAGAGTATTTTCTGGCAACAAGTTCCCAGTCGATGGCTCCGTCCCTCACAA
AACTTTTTACGACAAGATTATAAAAAGTTGTGGGTGTCCAAAATTCGAACTTTGCGTCTGTCATCCTTCGAAGGGCGATA
CGATACGCTTTTTCTGTTGGGTCCCCAGGAGGTTTGGGAAAGGATTTTGAAGCGCACATCGTCAAATCTTTACGAAAATT
TTCCTGAAGAATGCTGCTTGCAAACACCATGGGTTTTTCCATATCCTTTGACTGGGAAAAAGCCTCTGAGATGGCGATGG
CAGTGCATGTGTTGTGACAAACAATTCCTTCCGCGAGGTAGTTGTGGTGCTTTTGAACCTCAAAGTCGTACACGTATCCG
TCGTGCTCTTCCTCTTCGAAAGAAGAGATGGGGACAAACACAAGCTCTTGCTCGAGAACTCGCGAGAGTTCTTCAGAGAA
CAATCTCACGTCCGTTTTGCTCGCGCTCTCGAGTTGGGGATTCTCTCCCTGCCTCGCACGAACAAGAGAAAGAAGCACTT
GTCTCGCATCGGTCATGCTCTCGATGTTTCTCTTGTCTGGCAAAAGACAGGAAGATTGCACTCCGAGTTTTTGTGAGATT
TTTTGAAGAAATTCTGGGTAAGGGAAAGGTTCGGATTCTGAGGTGCTCTTTGTTGTCGGTTTTTGGATGGACTTTGCCGT
CAGAAGAGGAAAATGGATCTTCTCTTCGAGACGGAGAGCGTATCTTCTGTGGATTTTTCCAACATTTCCATGGACGGACA
TCTGAACTCCAAACCTCAAAAGCAAGGAGGAAAAATCCAGACAACAGGATCTGTTTTGGAATCTGACGAGGATGCTTCCG
TCTCCACCCACCCGAGAACTTTCAGAAAGGTAGGATTCGAGGAACCCGATAAACTCCTTTTGTTCGCATTCAGCGAACCA
CGGAGACAGGCCGCTGCTGAATGTGAAGTCAAACTCGTACGTCGCAAGAAAACGTTCCAAGCTTCCAGACCGGAAGGCAC
AACAAAAAATGTCCTCCTCTGAGCCATCTTTTCTCATTGTCGTCTTTTGTGGGCGAATTTCTCTGTCCGTGGCGATGTTG
TTCTCCATCAAAAAGTTGTTAATAAACTTCTCAAAGAGCATATATCCTCCCATGCTGGAAAATGTCCACAAAAACTTGCC
GTTATCCTTTTGCAGAAGGGGGTTCTGAGAAACAGGAGAACTTCCGAGGCCGAAATTTCTGGGGTCTTGGTTCCTCAAAA
AGTTTCCATAAGTTGTGAACCATACAGCAAACTCGAGAGGCACTTTTTGGGTTTGCTGTTCGCATCTTAGAGCCTTTGGA

ACAGCCACAAAATTACACTTTGAAAAGTCGTTGGTCCAGCCACGCTCCGTAAGAACGCGGTGCGCCTTTGTCATTCGAAG
TTTCGTGCCTCCGGGGATTGTGATGACGTTGAGCCTTTCGCGAACTCGTTGACGGTAGAGCTTTGAAATCTGTGAGCGGC
ACATTTTCTTTGTTTGTTCATCAAAGCTGTGGGTGAAAAGTTTGGAAGAAGGGACCTTCCACTCTCCTCCTTCGGCATCA
ACTTTTGGAGGGCCGACACCATAACTTTTCCAAATTCCTTCAATGTCTCTGTATTTGCCATTGACAGAGACGAGAGTGGA
ACCGTGGACACATTTCCCAGAACCGAGACCGTGATACACGAGTGCCTCGCTGTACTCGGTGTGAGGGGACACGATCCTCG
CCATCGTCTTTTGGTGGTTGTAGAAATCTTCTTCTTTGTCTTCTTTCTTTTCGAGCCTGAGCTCAGCGAATTCTCTCTTT
TTCGCCAACTTCTCTTGCAATAGAGGGTCGTTGATGTCCGGATAAAACGGAATAAAACTCTCGAGAGGGATTTCCATGTC
CTTTGTTACATTGGGGAAATATTCCCTTTGAAACATCGTCGAGCAACTTTTGTATTTTTTCTTTGCTCAAGAAAAAATAT
TATCTCCTCCATGAAATCTTCTTGTTCTCCTCGTTGGCGAGAAACTTCATGTACTGTGTTATCGATCTATTGAAAGAACA
TGGGCGACGTTTCAAAACTCTGGTTTTTTCCATCCACAAAAGTCCCTTTGTCTCTTTGTTTGGGCGAAATTTGGCAATGT
CCACCATGTCGATACGCAACAAGATGATGTACCCTCCGATTTGGTTCTGCCTCCCACAGCAAGAGCGAAGAACGAGAGCT
TCTCTTTGTTTGTGAAGAGCGTCCTTGACAATGTCTGTCAAAAGACCAGAAGATTCTTCGGTGACTTCGCGGAGAAGAGC
GTCGACAAGAGTTTCCGTTCTCTTTCTTCCTCCACCAAAGTCTGAAATCCTTCCATCTTCCTGTTGCGCCAAAAGCCAAT
GTTGTCTCTTTCCGACAGTGACGTATGGGACAACTCCAACTCTGATGACTCCTTCATCATCAGAGAGAAACTTGTCGAGG
GTGGATAGTGAAACTTTGGACATTTTGTCTGTGAAAAACAAAAATATTTTTAAACGACTAAAAGTTCAATAAAATGTCTC
GCAAATTTTATCAAGAACTTTACATGGAGAGCATTTGGAGTGGACAGAGAGAAGTCTCTGAGTGTATGAGGAAGTTCACG
GACCAACACAAGAAAGATCCAAACTTTGTGTCAGAAGATTTTTCATGTCTTTCGAAGGTGTCGTCTCATTGGGCAAAGAT
AAGCGGTTTCTGCTCTTTTGGAGGATGTGAAAGAATTGCGTTTACAGAGGGATGGGAAGGCCAACATCGACACTTTGTGA
AAGTTCTGGGACATCTCAACAAGGAAAAGGATGGATTTTTCAAAGGAGAGGTTTTCGACTCTGACAAAGTTTTCGGAAAG
TGAAGAAGCGCCGGAGTCTCTTGAAATTTTCTTTTATGAGCGAAAAGAAAACAGAGCGTAATGTCCGACTTTGCAGAGTT
TGTCGATAAAGTTCTCATTCTTGCAGGCATCGGTCCAAAGAACAGGAAGAAGATGACATCGGAAAAGAATCTCGCAAAAT
TTCGTGTTGCTTTCACCCACAAAACTGTGCAAACCAACGACAAGAACAACTATGAGCTCTACGAACTCGTCGGAGACAGT
CTTGTGAACGCTGCCATCCTCGGATTCATTCGAACGAAAAGGCCAGAGATCACAGACGTCGAGACTCTGACGAGAATAAA
ACACTATATTCAGTCGAGGTCGTTCCTTTCCCTTCTTGCTTTCCAAAACGGATACTTTGAGCACGTCACCGTCGGCGAAG
AGTTTGCAAGGACCATTCTGGAAGGAGCAGAAAGGAGAGGAAGACGTCACGACAGAGAACAGAGAGTGATGAGTGATGAC
ACGAGGCACAAAATTTCAACTCTTGGCGAAGAGGCTCTCCAGAACGAGACGTTTTCCAAGTTGATGACAGACCTCTACGA
AGCGATGTGCGGTGTCATCTCATCTCTCACGGAAGAGATGACCAAAGTTCCGGGGATGGGGTACATCCCCGTCTATCAAC
TCACGAGCAACTTTTTGGAAAGGTCGGACATTGAACTGACGTACGAGAACATCGTCGATCCTGTCACCCGTCTGAAGGAA
ACGTATCAAAAGGTGAGATACACCACAGATTCTGGAGAAAAGAAAATCTGGAATCTTTCTGAACTCGAAGAGAAGGAGAA
ACTCCCAGACGGAAATTTCAAAGTCACGATCTACGGATGGTTCGGAAAGACAAAGAATCCAGCCATTGAGAAGAAGCAAG
TGCTCGCCTTCGGCATTGCAAAGGACGTGAAAAAAGCGCGTTTGCAAGCGGCAGAAAGAGGTCTTGCCGCTCTTCCAAAG
TATGGCCTGTACGAACAAAAGAAGCTCATGCGGAAGTAGAGCGTTCAATATTTTTTCATCGATGAAAAAATATATACATG
AGGCGAGGGTTTTTACTGACCGTACGCGACGTTCAGACCGAAATACCTCTCACCACAACAAGCGTTCTGAACGAGAGGGG
CGGGCGCACACGGAAAAGACGGAATAGCTGGCGCGGCTAAAGTGCATCCACTAAACGCGGGACCGCATCCGGAAGAAACC
GCAGCGCAACCTCCCTGGGTAGCAACGGGCATCGCCGCAGCGACGGGCACCATCGGAGCTGACACTCCAGAACACGAACC
GCAAGCGGGCGAAGAAAAAGCAGTCGAAGAAATACGAGCACCACACGACATTACTTCTATTCGACAAAAGAAAAAAAATT
ATTGAGAAAAATTTTTTATTGTGACCAAAAGAAAAAACATGAACTCGAAAATTCCGTGCGCTGAAAGAAAACAAGGCCTC
TGCGGTTTGGGAGAGTGCGTTTACTGTTTTCCAAAATCTTTTGCTTCTCACCCTTTGTCGAAGCATTGGTCAGAGAACAA
CAAAGAAACCCCAAATCTTGTGGTCTTGAACTCCAGAAAGGTGGTTCTTTTCGTTTGCGACTGCGGGCACGAATACGAAA
GGAAACCGTTTGATGTCTCCAAGAGGGGTCTTCTTTGTCTCTTTTGCAAAGGCGAGAAACTTTGTGGCTCTCCCTCTTGT
GAAGCCTGTAAACAGATTTCTTTTGCGTCTCACGAAAAAGCGCAGTGTTGGTCCGAGAAGAACGGATTCTCCCCCGAAAC
GGTCACTCAAAAACATTCGAAAAAATGCTGGTTCGAATGCCCCAAATGTTCCCACTCTTTCCAACAAGAGCCGAGAGTCG
TCTGTCTTCCAGACAAGTCCAAAGGGAAACGTTGGTGCCCTTATTGCGCCAATCAGAAAAAATGCGAAGACGAGGGTTGT
GAGCACTGTCTAAAAAAGTCTTTGTGTTTTCTTCCTCCGTCCCTTTCGTGGAGTAAACGGAACTCTGTCGTCCAAAGGCA
AGTCTCTGCGGGAAGTAAAAAGAAACATTGGTTTGAGTGCAAAATTTGTTCGCACTCCTTCCAAATGGCCCCTGGTTGCA
TCACGCACCAAAAACAAGGGTGTCCTTATTGTTGCAACCAAAAAAGATGCGAAGATGATACTTGCGGTCATTGTTTCAAA
AATTCATTTGCTTCCCATAACCTTTCGAAACTCTGGAGCAACAAAAATATAAAGACCCCAAGACAGGTGTCCCTTTGGGG
AGGGAAAAGTTACCTTTTTGAATGCGATGTGTGTTTGCACGAGTTTTCAATGGAGCTCCGTAACATCAACGCAGGGCAAA
GATGTCCTTACTGCGCTTGCCAGAAGCTCTGCGATTCCAAAGATTGTAAAATGTGTTTTGAAGGCTCGTTCGCTGCCTTT
CCACAATCTTCCTTTTGGTCATCGAAGAACGAAAGAAGTCCAAGACAAGTCAGAAAGGGTTCCGGAAAGAAATACAAGTT
CCAATGTGAGAAGAAACATCTTTTCGAGACACCCCCTCGTAACGTCAGGGCAGGCTCTTGGTGTCCCCTCTGCAAATCCA
AAACGGAGGCCAAGGTCTTCTCCTGGCTTCTCTCTATCACCCCTCTCGCTGTTCATCAAGCGAGATTTTCATGGTGTATC
AACCCAGAAACAGGAAAGGTTCTTCCTTTCGACATCTGCGTTGCGGACAAGTTCATTGTCGAGATCGACGGAAGACAGCA
TTTCGAGCAAGTCTCAAACTGGACACCTCCAGAAGAGACACAGGAAAGGGATAAACTCAAAGAAGAACTTGCAAAGAACC
ACGGATTCCAAATTCTTCGCCTTTCGCAACGAGACGTTTGGGAAGATTCTCTGGATTGGAAGAACCAGATAAAAGAGTTT
ATCAAAGAAATATTTTAGACACAGACAAAATATTTCGAGAGATGGAGAAATTTTATAGGATGAGAGAGCTTGTTGCTCTG
TGTTGTTCCAACGGAGAAAACTTCCCAAACCCCAAAGATTACGCTTGTTTGTACTATGACGAAAAAACGGCAAACTTTGG
GACGATGTTACCAGACGGGAGCTTTCATGGACCTTTTACCAAGTTTTACGAAAACTTTACCGAAGTCATCACATACGACA
GAGGAGTAAAGCATGGGCCTTGGTCAAAATCCTCAAAGAGGGCGCGGTGTGATGGAAACTTTGTGGATGGGAAGCCAGAA
GGAGAATTTATCATATCTCAGAGAGTGGAAATCCCGTCGAACGCTTGGCCAAAGAAATTTCGAACTTCCACATCTTCTGT
GTTTTACGAGAAAGGAAAGGGAGTAAAGCACGTCCACAACTCTGGGGATTTTTGCCCCTTTGAGTGTAGTTTTTTGGGGA
TGACCCTTCGAGAATCTCATACAGTAGACTGGACACTTTCTCCAGAAATTTGGTTCAAAGTTACCACAAACACTATAACG
CTCGAAGGAAGATTGCAGGATATGGAGAAAGGCTGCCACGCATGGAACTCTCTATGGTGCTGGTCAGAGTTGCTGCCAGG
AGAAGCCAGAAGACTCAGTCCGTCCAAGGAAGAAGGAATTTGTATTGTCCTGCCTTGAGATATTAAAGAAAAAAAGAATG
CGTCAAACCATCGCCAAGATATCAGACTTTGCGAGATTTTCTCGCGGAACGCTCCATACGCATCTCAAGCGGATGTGTTT
TTTTGAATGAAAGGTAAAAAACAGTAAATATTTTATTTCTGAAATAAAATATTCTAACGAAGTCTCAGGACAAGGTGGAG
AGTGGATTCTTTTTGGACATTGTAATCGGCCAAAGTTCTCCCATCTTCTAGCTGCTTCCCAGCGAAGATAAGTCGTTGTT
GGTCGGGCGGAATACCTTCCTTGTCTTGGATCTTTTGCTTCACAGCTTCGATGGTGTCTGAGGTTTCCACGTCTAACGTG
ATCGTCTTGCCCGTCAAAGTTTTTATGAACACTTGCATTTTATGGTTACTTACCGCCTGTTTAAAATATTTTTATTCGAA
TAAAATATTGTAGTTCTGTGGAGAAATTCTCTCGGAAAAAGAGACGAGTTTGTTGTTCTTACAAAAGATGTAGAGTCGAG
TGTTGTACCCTTGTTCACGACAAGCAGCCAACTTTTTCAAAGTCTTTTCCGCTTCGTCTTTCTTCTTCCCTCCACAACCA

CCAAAGGTCCAACTTGACTTTACCTCGACGAGAAGATTTTTTGAAGGGATGAAAATATCTGGATGATACATGTGGCTCTT
TCCTTGAAAATTATAGAGAATTTTAATGTCTTGTTCTGAAGGGGAGAGAATACCTTGTTCGTCTATTCCCTCATCAAGAA
GGAGTTGAAGAGCGAGGGGTTCAAAGCCTTGGCAGATGAAGGGTGTTCCAGAAGGCATTAGAAACTCTTTTTTGCGAAAG
GAAGATTTGAGAATTTTGCGAGCGACTTCGGGGTTTTGAGAAGGATGGTCGTAGCCGGTTTTGGCTTTGTACGTTTTTTG
AGACTTCTCCCAAGTCTCTTTGAGTTTCATGGGGTTGGACACACCATATTTTTTCATACATGTCTCCTTGCATTTTTTGT
TCGCGTTTTCAAGTTTTTCTGGCTTTCCCTTGTATCTTTCATCCACAGATTTTTTGTGGTTTTTGATGACTTCCTCATTT
TTCATGGGGTTGGTCGAACCGTGGCGCTCCAAATTAGTGATTTCCCTTTTCTGCTTAGTTTTTTCTTTATCTTCGATGCT
TTTTTTGGAAATGGCTTCTTTGAATTTATTTTTGACGGTTTCGCATTTCATGGGGTGGCGAACCCCATATTTTTGTATGT
TTCTTTGTCTGGTTTTCTCTTTAATCTCATCACTTTCCATGGTGTTGTGTACGCCATGATTTTCAAAGAGAGTCTTTTTG
CATTTTTTCTGGATTTCCGGATTTCCCAAAGGACTGTCTGACCCCCATTTTTCTTTCCATCCTTTGATTATTTTTTCCCT
TATTTCTGGGGATTGCAAGGGAGTTGTACTACCATATTTTTCCAAGCAAGTTTTTTTGACTCCTTCGGCTTTACATGAAG
GGCATCCATACCATTTCTCACGTCTCACAGGGTATATCGATGCAACGACGGATTTCTTCCCACAAGAACAAATAAACTCC
AGTTTTTGCTGTGCTTTTTTATATTCTGTCGAAAGGCGACATCCTTTGCGCAAAAAGAGGTTACTTGCTTCTTCATGGGT
CATTTTACCACTCTTTTTTCTTATGCATTCCTGGCATCCGGTGAAAGGCTTTGTGGTGAGTCTAGAGTAAGACGTGGTTC
CCTCTTCTCCACAAAAACACTGAAAAGTCATATTGCAGTTCGAACCTCCGTATTTGCTGAGAAGTTTGCACCCTTTCTTT
TCGAAAATAAGAGCAACTTCTTCTTGGGTTTTTCTGGGAGGCATTTTATTTCTTTGGGTAAAACAAAGAAATATTTGTTC
GATAGCGCTTGTTTATTCGCAGGGTCCGCATTGAAAAGGCCCCAATTCTTCAGGCATACCTTGGAAGTTTCCGGACTGAT
AGGCAGCGTGACCCACCATTTCGAGGGGCATTTGCTTTTTGCTCATACCGTACTTGTACATAAAAGAATTGCCTGGAACG
AATCCAGCGTGCGAATATGCCTCGGGATCAGTGAGCGTGTCAAGCCCCTCCCTCTTCGCGCCTTTCCCATCACACTCCAC
TCTGCAACCTCCGAAAACAAAGAACACAATCGCAACAACCGCCACGATTAGGACGATGGTAAGAAGCCGACAGTCAGTCA
TTACACCTCGAAAAAATATTTTCAACACAAAGAAAAATATTTTCAAAGGAAAGATATTTTTCTTGCAAGCATAAATGGCG
TCAGAAATAAAAAGAGCAGTAAAAGAAGCGTTCCCTTTCTCAGAAGAGACAAAGGAAGCTTCTCTTGAAGCTCTCGACAA
ACAAATTCTCAGCCTCCAGAACAATATCCAAAAATATGGTACATTGTCCACCAGAAACAGAATAATCTTTGCTCCTCTCC
ACTCTAAAAATCATGTGTGCCGATACCGCGAACTCGAAGCTTGGAAGCTCGCAGTCGAGGAGAGAAGAATAATATTTCTT
AGTTAAGAAATATTGTGGAATGAGGGACCAGAAAACTTCCAACCTTTCGCTCTGGATGTTTGAGACTGTTGCTTTCGATG
TTTGGCGAAACTATAAAAGTCTTCGTTCTCTCTCTGCTCGCCATTTATTTTGACATTTCTGTTCCCAGGCCAAAAGGTCC
ACAGATTCATAGAAAGACATCATGAATCTCGAGCGAGACAAAATATCGTAAACAAGACAAAAATTTCCTTCTCCGATACA
ACCGCAGTCTCTGACTGGAGAGATTCCTTCAGGGAGTGGACAAGGCGCGTCCCCTCTCCTTCCGCTGAACATCAATCCGT
CGCGAACAAAAGGTCTCTGGGACATTTTTTATTTTTGTTGAATAAAAAATATTTATGATTGACTAGAGCGTGTAAAGTTC
GTTCTGGACGCTTTGGACTGGGTCGCTAAACATCGCATTGTCCGTCGCTGCTGTCTCTGAAAAAACCTTGTCTTGTGTGG
CGAGTTGTTCTGGAGCTGGAGCTTTGTTCATGAGACGAACAGCGAGCATTCCCAAAGCGACCATCCCCAAAAGAGCCGCA
ACTCCAACGACTGCGCCAATCACAGCTCCGACAACAATTCCCGCAGTCTGTCCCGGAGTGAGACCTCCACCTCCTCCGTC
TGTCGAACAGTAGTTGTTTGAAAAGACAGTGGAAAGGATGGCGCTCTCGCTGTCGTTGGACGGAAAAACATCGGACAGTG
CATTGAAAGAAACCACAAACACTCTGTTGCTGATTGTCTCACGAACGTCAGAGTCGTTGAAATACACGGCAAACGCAAAG
ACCACGTTCTTTTCTGTCTCTCGAAAATTTCTTCCATAGACAAACAAAGAAACGTCCAAAGGCACCAGTTCTCGAAAACT
TTCCAAGATCTTTGAGAAGAGAAGGGTTTCGTTGCCTGTGGTTTGAAAAGTTCCGCTCACTGTATATCTTCCAGAGAAAT
CCTGGAGACAGGTCCCATAAACTAGAGGAGTTTGCGAAGGGGAAGAGCTCGTGGATGGAGTTGGAGACGACGAAGGAGTT
TGTGTGGGAGAAGGAGAAAGTGATGGACTCGGAGTGGTCGTTGGAGAAACTGACCTCGTAGGAGATGCCAAGGGACTTCT
CGAAACGCTCGGAGACAAAGACGGAGAAACGGAAGAAGAGGGACTCGCTGTTGGGGTCGAAGAAGCGCTTCTTGTGGGAG
AAGGAGAAACAGGGGAAGGCGTTCCGCTAGGAGTGGACGAGGGTGTTCTCGAAGGTGTCGGGCTTGGGCTTTTCGTCACT
TGCGATGGAGAGGGAGTTCTTGATGGACTTTTTGACTTTGATGGTGTGGGAGAAGGACTCTTTGTCACCTGTGATGGAGA
GGGAGATTTTGAAGGGGAAGGCGTTCTCGAAGGGGACCTTGTCGGCGTTGGAGAAACAGGAGAGGGCGTCGACGAAGACG
TCGGGGGAGGACTTTGTGAACGAGAAATTGTGGGAGATGCAGACACCATCGAAGGAGAGGGAGTTCTTGAAGGAGAAAGA
GAGGGAGAATTAGAAGGTGTTGGAGATGCGCTTTTCGATGGTTGAAACGAAGGTGAACTTGAGCTTGTTGGGATTGGGGA
GTTTGTGGGGGAAGGAGACGGCGTTCTCGAAGGAGAAGGAGAAGGCGAGGGGAAGCAATACGCGGAAAACAAAACTTCCA
CAGCCACCGTACCGTTTCCGCTGTTGCAGATTCCTGAAACCACCTGGTTGTTTATACCCAAAATTTCCGCTCTTTTAGAA
CTATCGTATGCTGGGTCCACGACAACATATGAAAAAGACACAGACAGACTGTCTGCTGAAAAACTAAAGTTGTTGCTCCA
GCTGTACGACGGACCGAATGTCTGCGTATAGAGTCCCGAAAGTTGAGAGGACACAGAGGAAGAAGTGACTGGATTCCTTT
GCAATGAACCAGAACAACTCGAGTTTGTCGAAAGAAGACAAGAATCCGAAATGACCTGTCCCGCAAGGGAGAGCAAAAGC
AGAGTCGTCAAGATTTTTTGCATCGTGTTGTTTTTCTTTTCTCTGTACAAAAGAAAATTCGTTTGAACTAAAATATTCAC
CAGATTTTCACGACGCGCCTGTCCTCTTCTCGCCTATTTTTGACGTTTCCTTCTTTGTCGCAAAAGAGGAAGATTTCTTT
TACGCTCGGGAGCAGAGTGTGTCCGGCGACAAAGGAAAGACCATCGCTAACGTGAAGAGGACCGAACGTTTTGTTGTCTT
TTCCTCGACATCTTACCAGATTTTCACAGTTCTCTGGGCTTCTGTCTACCACAAACCACTCGCAAAAGTGCTCGAAATCT
GTGTCTTCGAAACAGACAGAAACCCTTTCTCCTTCTTCTTTTACGATCACTTTGTGGTTTGGGGAGCTCCCAGAATATTC
CATCGACGACGTCTCGACAGCAGCAGACACTGTCTACTTTCGCCTTCATGGTTCTGAAGGAATCTGCATTGGTACATATT
CCGACGAGTTTCTGGAGAAACTGTCCAGAAAATGCAAAGAGCTGACAGAGAACGGAAAAAGGTGATCGTTGCGTTCAACA
ACATAGTTTCTCAATGGTTCTACAAGAGAAACTTTCATTCGTTCGAAGAGTACACGTCCGCCATTTCTGACATCACACCG
AGTGCAGTCTACAACGGACGGAACCTGCTGGGTATGCTCTCTCAATAAATTTTATCAAAGAAAATTTATACAGACGTCGC
GGTCATGGGCCTTCCAAAGCTTGAAATATATCCGCCAGCTTGAGTGGCAAGTTCGTGGCGAGAGGTGAAAATGTACACAA
GAACTGCGGCGATGACAAGGAAAACTCCCACACCAGAGATGATGGCGGAAATCATGGCGTATTTATGGGCAGATTTCGCG
AAGCTGTCTGTGTTTGCACAGCTGCTCTTTTTCACATCGTTTGCCGCCATCGCTCCAAACACGAGGTTCAAAAAGACAAG
GACGACTGTGGCGACCAAAAGAATCACAAGAAAGGCAGACACCATGTTACAAATGTTTTTTATACAAAAAATTTATTCAA
GTTCATCTAGTTTTTTCGAGAGCCTCTGAACTTCAGCGAGCAAGAGAGAGATGAGAGAAAGATGCCTTATTCCCACAGCC
TCTCCTTTTGCGTTTTTGACCACAAGTTCTGGGAAAACTTCCTCCAATTCAGATGGAACGACACCATAGTCTTTTATCCC
TCCAAGTTCGTACGTTTTCACAGAGAGATATTGGAGTTTCTCCGTATCGACGTCAAGCTCCCTCGCGTTCTCCATTACAA
TTCCTTTGAACAACAAAAGAATTCACTCTCCTATCCATCGAAATGTGAAGTTGTACCTTGCTTTTTCGAGATTCTTTCTC
TTTGGAAGACAGTGCTGCCAAACTTGCTGCATCCCTGGCAGCATCACAAGGACGTCTCCACTTCCCAGCGATATTGTTTT
TTTGTGTTTGTGGTCCTTTATCAGCCTCAAACAAAAGTCCCTCCTCGCCCCCAACGAAACAGACACAATGGGATAGCCTA
CTGCGAGGTCCCTTTCTTTGTCCGAATGCCAACCGATGTAGTGGTCTCCGTTCACGTATTTGTTGACCAACACATAGTTT
GGAGGAGGGCTGTCTTTCTCCATCACCCCTTTATCGACGAGGTGAGCGTGAAGTCTCAGCGCCAACTTCTCGATAAAGGG
AGGCCACACTTTTGCAGGGATTTGCACGCCTGTGAAGCCATACGAAAGATTTTCCGTTCTTCCAAAGCCCACCTGAAGCC

TTGGAATCGGGATGACTTTGCCATGCATTTTAATGGCTGTGTCCTCTGGCGGAAGATATTCCACCTCTTCGAGCTGCCTT
TGCAACTTTTTCCTTGAGCCAAAGAGAAATCTCTCAGAATAAAAAATGGAGGTTCCATTGCGAATTTATATTTTTATAA
ATTCTGTCATATCTTCGAATTTGCAAACAAAACATTTTGTCAAAGGGGCAGTTCCCCCAGGAAATACCTCATTTAACAAC
AGTTCCAAATCTCTGAGCTGCTGTTGCGTCGGCGCTCCGTCTCGAACACGATACCCAAGGGTGATGTGAAAATTGTACGG
CTTGTTTTCGACACCTGACGCTCTCGATATTTTATCTCTGAGCCCGTCATCGCTCCACGACACTTCTAGACCGAGCGTTC
CGTTTGTCCAATAGATCGCAACAAGAGAACATCTTGTTTCTTCAGAAGCGTCGCATACAGAGGAAATATTTCTTAGGACG
CTGGAATTGTCCCAAAGAAAACAGTCAAATTTTTCCTTTGTTTGCGCTTTCTTTGGGACGACAAGGTCAAAAACTGTCAT
GTGATAACTTTCGAAAGGAAGAGGAGAGAAAATCTCAGAAAGACCAGAAGTTCTCAGATGGTCCTCCACCTTTTCGCAGA
GAACGCTCTTCACAAAACGCAACGTCGTCACTCCAAAGAAGGGCAGGTACTTTCCAGAGTTTGAGAGCTTCTTCATCTTT
TCTTTTTCAACAAAAGAAAATTACGAATTTTCGAAAAGCATCGAGACGCCGAGTTTTCTCTTTTCCGGATCGGGAAGCAA
CCACCTCGGAGCGTCTCCTTCTTTCGTCACAACAGCGTTTGCGCTGAACCCTCCATCGAGAGAAACAGCATGCAAAACAT
TCCCTCCAAAGAAAAGTTCGATGGCCTTGGCAACTTGCACTCTGTCCGTTCCGAGCGCATCGTATCCTCTCCCTTCAAAG
AAAAAGAAGCCAAAACTTCCATCCTTCAAAAGAACCATGATGTTGTGAATTTGGAAATTTGCACTGTGTCTTTGGCCGTA
CGGGAACTGCGTTTCTCCTTGTTCAGAGAACCACATTTTTGTGTTTTTTGCTGCGTCAAACACTTTGTACTTTCTTCCGT
CCTGAAGGGAAAACTCAGAGTTTAGCATCGTGTTTTCGTCAAACACCCTCTTCCCTCCCCAGATCAGGATGGGTCCAGAA
CAGAAGCATTGGTCGTAGATGTCGTTTCTGCTCTCTGCGATGACGGTCTGAGAGAACTCGAGAGTTTCTCTTCCCTCTGT
CAGCACACGAAAGGGGACAGATCTCAGCTTGTGTTTCTTTTCAAACTCTGGAAGTCTTTCCATCCCCAGTTTCCCGCCCT
TGACCGTCACAGCGACAAACCAATCCCTGTAAGGAGGAGGGACAGGCAGAGTCGTTCCTGATTTGCTCGTCCCATCAAAG
TAGTAGCCGATGGGAGAATACTCTTTTCCCTTCAAACCTGGAGTCAACGAGTTTGCGATGTTTTGTTGAACAACGAAATA
ACCTCCATTGACGCAAAGGGCGGCACTCCCATCCAAGACATTTTTTGTGATTCTTTGTTGGAGTCTTTTCCCTGTGGACA
GAGAACACCTTGCCCTTCTCGAAAGACCGACAAAGGAGCATCTAACCGTGCGCATCGGGCGTCCCCAAAGTTTTTCAGAG
GGACTTTTAAAGTTCAAAAGTCTTTGGTACTGTTTGTACACGAAAATGTCTGATATTTCTGTTTGAATATTTGGAAAAAG
AGGCCTTGAAAAAATAGAACACACTTCGGAACCGTACGTCTCGAATTCTTTCCTTGCAAAGGGAACGACCTGCGCCAACG
AAGGAGACAGCGATGTGATTTTCGGCAAAAGGAACTCTTGGTCGTTGTTCAGCGACTCCACAAGCTTGATGGCGCACTCT
TTCTTTCTGTTTTTATCCACGATATAACCCTCTCCAAAAAGTTCAGTGCTCTTTCCCTCCACACATTCCTTTGAATATCT
TAGAATCTCTGAGCGGAATTTTGTCTCTGTGTTTGAGGATTGCACTCTCGCGTTGGGGACGTGAGGAACGACGATGGTGG
AGGGGCTGTCCAAAATAGCAGAGAGTTGTGCTTCTGAAGGTCTCTCGAGTTTTTTTGTGAAAACATGGAATGTTCTTCCT
CTGAAATTTACGGATGGAACGAGGGAACTCACAGCTTCCGTGGAAGTTTTTAGAGAAGAGTTTCCGACATCTCTCTCGTT
GAACCAAATTCTCTGGGCTTCTTCTGGGGTTGAAGCTCTTCTTACAGCCTCTGCTTTCTTTGCGAACGCTCTCTTGATGG
AGTTCTCGTTGATGCCAAAGGCAAGAAGGGTCGCCTTCGTGGCATCGTAGAGCCCTCCGTCGAGAACAACTCCCGGATAC
ATTCCCAAAGAAATTTTTTCACAGTCAAACAAAATGTTCAAAAGAAAAAAGATGGCTTCGAAATTTTTCAAACCCTGTTC
GTCCAAGAGGCTCTCTTTGCACCTGTTTGCCATTTCATAAATTTTGGCTTCGTTCGAGACAGCAAAGTCTCTCCTTTTGA
GCTCTGGGTATAGGGCACCAGAACTTTTCGAGAACCTGACCACTGTGAGTCCATAGTCGATGACTTTCGGTATTTTGCTG
TTGTTCTGGATGACAAAGAAATTTCCTGTGGGAAGTTCCTCCACAATGAACTCTGCCTCTGCCCACGGTTTCCCATCATA
AAGTTCAGCTCCTTTCTCATCTGTCGTTGGAGAGCCTACGCTCTTCACAAGCACGTTGTCCGGATGAAGGTCTGCATGGT
AAAATCCGATGAGAGACTTTGCTGTCTCAAGGTCTCGAAACAAAAGAGCGAAAAATGCAAGATAGTCGCCCACAGAAAAC
CCAGAAATTCCTCCTTTTGTTCTGACGAGTTTGCGAAAGCTCTCGCCCGACGATTCCATCAACACAGTGGCCGTGTACTT
TCGCTGTTCGCTCTCACAGAATTCCGTCCCAAACACTTTTGAAAAACAAGGGAGAACTCCAAGGTCGAAAAGATTCGACA
AAAAGCTCGAAACTATGGCTTCATAGAGAGGGTCTGTCAAAACAAGAGTCTTTTGCTGCATGTTGCAAATAAGGGGCTGC
GCTCTTGCTTTGTGAGTTCTTTTGGCAAACGCAGGAACCCAATAAACTCTCCATCCGCCTTCGTGGTTGAAACTTATCTG
CTTCAATCTAAATTTTTTGCCTGAAGTCGTTGGGAAACCGGCGATAGAAAATCCTCCGGCTCCCTGTGTCTCGAAAGACA
CCTCGTCTTTTTTGCAAAATCCCCTCGACCGAACAACAAAAGGTTGGACGAGATTTGCAAAAAGCCAGTCTCTTTTAGTT
TCGACGTTCAAAATAACGGGAAAGAGGCGAAACAGTGCTCCCAACGCTTTTTTCGAGTCGTTCAAGCTGAGGTCAAGGTC
CTCAAAGCACGTGTCGAGCTCAAGGTCGCATTTATCCTGCTCCATTACAAGGAAAGAAAAGATGTGTATTTTCTTTCAAA
GACAAAATTTGGCTTTTATGGGTGTTCCATGCCGTGGAAGTGTTTGGCACTCAATGTCAAGTTTTTCACAAAGTTGCGGA
TAAAGAGAAAAAATGGAGCTCTGGTTATCAATGCCCACAATCTGTAAAAATGCCATAGTTTCCGTGCTGCTTTCAATATA
GAGGTCCAAGTTCAAATATTTTGTGGGAGCGATGTCCTTATATTCGAGTCCTAAAATCTTGCAAGATTTTCTATCGAATA
TAAGGTTCTCCATTATTTTGAACCTCTCTTTTTCTTTTGCTTTTTCGGTTGGCCCATCGTACGAACGGCATACTTTTGTT
CTGCTTCCCCATGAGGAACATAGACTTTCACACCTTGAACGTAAAAGTAGAGACCTCCGTTTGGCCCTTCAAATAGGGGT
CCAGAACATCTTTTTGTGCCGCATTTCGCCCACTCGTCGCAATCCCAGTTCCATTTTTCTCCTCTCGCCAAAGCGGACAG
CCTTTTCCCGTAACAAGTCTGAAGTTGTCTCTTGCTTTTGAAGGGCATCTTACAAAATATTTTATAAAATATTTTATGAC
CAGTTCCCTCCAGACATTGGTCTCAGTTTTTCCTTCCCCCGAAGGTGAGGTTCACTTCTTCTTTCCACAAACTGCGGAAT
TGCAGATTGGGAAGTGTACGTATTTCCCTTGTTCGGTTCAAGAGTTTGTTTGAGAGAAATTTGTTCGTTCCTCAGCGTTT
CTTTGACAGGATTTTCTTTCCTTGAGTCGGCGGATGTGGACACTCTATTCGCGAGCCTGGCAGGCCCCTGCCTAGAATGT
TCTGGATTTCCAAATTCCATCGCTTTCCTTCCCGTGCTGGTGTTTGTGTGGATGACTCTCTGTTTGAGTTCTATATTTCT
CTCTTGAACGTTTTCGAGGGGCTGCGACCTTCCAGAGCCGACAGAGATGGTGTGTCTTTTTTGTTGGAGGAAAACGTCAG
GACTTTCTGCGTTGACGTTAACACCAAATTCCTTTCTCGCATCCGCAAAGACTGAAGGGTTTCGTTGAGAGAGTTGAAGT
TCCTGCTCCGGTGCGTTTGTGTGCTTGACGGGGTTCCAAGAACCGGCAGACGCGAAAACTGAAGGGTTATGAAGCTCAAG
TTCGACATCAGGCTGCTGTGCGCTGAATGTTGTGAACCATTCCTTTCTCGCATCTGCGAAAACAGAGGGGTTTCTTTTGA
CGAGTTCAATGTCGGATTGTCCCATCGAACTGTTTTCTCTCAAACAAAAAGTTTTTCCAGCACCTGCAAAAACAGATGGG
TTGTGCCTCTCGAGTTCTGGCTCTTGGCTTTCTTGTTGTAACGAGAGGGGGATTGAGCTGTTCATTCGAGGCTGAACAGC
TCCCTTATTTCTGAGTGTGACCTCCCATCCAATCTTTCGAATGTTTGGGTCTGAAGCGCATTCGTTCGAAAAGAACACAG
GAGTGTTTTTTGGAGCTTCAGCAGAAATTGTGGGACGTTTCTGGCTCAAGTCAACAATGACATCGGGCTGAGGTTCGTCC
ACCCTATGGAAAAATGTCGGTCTTATGCTGTCTGCGCATCTCTGCTGTTTTATGTACCGAAGTCCCTCCACAGAGATCGG
ATTCATTGTTTGGAAGGGAATGGAAGGGTTGTTCCGATATTGCGTGGTCGGTCGAGGCAACCTCGAAAGGGGAACAAGGT
CATCTCTGTCGATGAGAGGAGGACGGAAATTTTTATTGACCTTGAAAGTGTAGCTTCTTTGACCGTACTGAGTTTTGTTC
CTGTCTCTTGCGACTGCGAGAATCGTTCCGTCGATGAGATAGGGATTCTCTGCATTGTCCAGAATGATGTCTCCTGGATT
CACAGACCACGCTTTGCCAGCTTCAACAAACCTCGGAGGGTCTTTTTTGATACAAGGCTGGATCGGCCCTTCGATGGGGT
TCAGAGGTTGCTTCTGTCTGAATAGCATTACTGATGTCTTCTTTTCACGAAAAGAAGAGAGAAGATAAAAATTTTATCCA
AATATAAAATTTACCCAAAAAGCCTCCACAAAAGTTTGTGGTTCTCGCTCTTTTCACAGAACTCCTCCGCGTCCACCCCA
CAACGATACGTCAATCTCGAAAACCTTTGGTTCGCTTCCACACATTTCCTTGTCTGTTTTTCTTCGCATGAAGCAAAGGC

TCTGCTGTTTTCATACGTGGCGCACTTGGCCTTGCATTCTGAAAAGATTTCTTTCTCGACCTGCAATATGCACAGGTAGT
TTGGTGTTTCCCCGCACTTTTGTCGGAGTTCAGACCTCGTGCACTGTTCTCTGCACAAACTCTGTAATTTTGTTTGGCGA
AACATTTTATGTTTTATTCTGAAAACATAAAGGGTCGCTCGTTTTGACTTGCGAATGATGAGCAAACGTCCATCAAAGAT
GGAGTGTCTAACCAGGAATCGCGGAACGACAGCACGTGGAACTTACACTCGACGGCATGATATGGATCGTCGTTCCGTCA
GAATCGTCTCTTATCAGAGAGTATGTTGTACCCTGTCCGACGTTGAGTTGGCCGAGGTAACGAACCTGAACTCCGTCTTT
GTTAGTTCCGCGAACCACCAACTTTGCATACGGAGAATAAAAAATGGGAGGAAGCTTCACAAAAGTTCGCAGCTTTCCGA
GACTCTTCCAGTCTCCTGAAAGGTCCTCCACGAGAATCTCAAAGTTGCAAGGTTCGATGGAAGGAATCCACTTGAAAAAA
ATCATACGAGTCGCATCCAAGTCGAGAGCGTTGGCCGGAATGCCCACTCTGTTGCTGTTAAAATAGATCATGCGATCAGC
CATCGTTACCCCAAGACTTTTGGAGAAAAAAACTTTTTGAAATGTTCAGAAAGACAGAGATTTGGCGCTCCATCGCTCTA
CAGAATTTTTCGAAAAGCTTCGTACACGCTGCATGCTCGAGGTCTCGCCTTTGGGTCGACAGAAAGAGCGTCCAAAAAAA
TCTGCGGAAGTTTATACTTTCCTTTTGCAAGAGAAATTCTTCTTGAAAGATTTTGGAGCACCAAAGGACGAAAGTCCCTT
ATCGGAGGGGCAAATCCCTCATTCTGTTCTATGGTCAAAACAGCCAGAATACTGTAAGCTGCACACCAAACATCGACAGC
GAACGCTTCCTCTTTTGTCTTTTGTTTCGCGTACATTGTGAATTCTGGAGGGTTGACCGTGGACTGTGACCAGTAGCAAG
GTTTCGAGAATACACAGCTAAAACCGTAGTCGACAAGCTTGAGTTCCTTTCCTGTGAACATGATGTTGTTTGGGTTGATG
TCGAGGTGGGCAAGATTCGCAGTATGCAGCTGACAGAGACCATGAAAAAGAACGTATGAAAGTTTCTCGAATTGTTCTTG
AGTGGGTGTCCACCCAGTTTCTTCTCCGCAAAAGATGAGTTTTGTGAGAGAGGTCCCTTCGACAAACTCAAGAAGAGAGT
AAACATACACTTGATGCTCTGTTGAAGTCACGCCGTATCCATAAGATGCCATCAAAAGGTTTGTGGGGGTTTCTGCTGCA
TTTATCTCAATGAAAGCGTCTTCGTCAGAAAGCCCGTACTCTCCCTCCCATTTCTGGAAAAAGGTCTCTCCAAAGTAGTC
TCTGTACCTTTTCGCTGCTGCCTCCATTCCCCTGGTGGAAACGTCCGCCAATTCGTTCCAGTCAAACTCCACATCAAATA
GCTTCAGAGCGTATTTTTTCCCTGTGTTTTTCTTTTCTACGAGATAAACTGCGCCATAAGTTCCCTCTCCAAGTTTTTTT
CTCACGACGTACCCAGAAAAGTCGTTGTAAGAAATGTTTCTCTTGAGAGCGCGAGAAGAAACGAACGCAAAAGTTTTTGC
ATAATCGGCATTGGAAATGGGAGCGACTCTCTGACCCAATTTTCTTGCTTCCCAGACCAACCACTGGGCATTGACATTTT
CCCCTCCTTCGAGGGGGTGTTCCCGTCGAATGAGTCCTTTCGTCTCTTCGACAAGCATATTACATCCAGAGAAACTTTTG
GGATGAGAAAAGTTTCATGCGTAAAAGGCAAGTATGTCCTTTGACGGGTAACCGATGGTTGTTTTCTCTTTGAAAAAACA
GAAATCCAGCTCTTTCCTTTGGAGAGAATTTTCTGGCACCGCCTCGAGCCAGTACGTCCTCTTTGGGTTCTTTTGAAATG
ATGGTTTTTCACAGGACGAAAAACACCAACAAATTTTTATGGAATCTTTGTTCCTCAAAAATATATAAAAAGTTTCCTCC
ATGTTTTGACAAATTCTTGTCTACTTTTCTTTGACAAATTTTTTTGTTCTTGAATTGTTTGTAATGTCAGGATTCGGTCC
CATCCCCGCCACCAACGTTACTCGCAAGTTTCTTCCGCGTCTCGCCACTGCGGCCAACTCCATCACCTCTACCGCAACCA
CCTTTGCTCTGACCACGCCTCAGACGGAGGCGAGCTACTGGTCGCGCATCGGAAAGACCACGACCGTCCATATCCACATG
GTGAACGCGACCCTTCCCGGAGCCGCCGCGTCCACTCGAATTTACGGAGATTTCCCTCCGCTCGACATCACTCCGAGCTC
CGCTCTCGCTGCTCAGCACGGAGTGATCGTCCCCGTTCAGTATTTCGTGGCGCCGACTCTTCCCGTCGGTTCTGACGCTG
CCGCCAGAATCCAGACTGGTTACATCGAACTCGGTTCTTTGCTGAGTGGAGCGTTCACCCCTCTTGCCGCCAACTTGATC
GGAACCGTTGGGTTTGAATTCTCTATCGATGCCACCTACGTGGCTCAGTAAAAATATTATGTTCAAATAATATTTATCAG
AGGCTAAAGAAACCATGGAGAGAGTTTGGCTCGATAGAAAAGCCTACGAAAAGGCCAAGGAAAAACATATAGAAAGACGG
AACAAACTCAGAGGACACAAGGGATGTGTTGTCCAGGAACTTCTTCTCGAGCCAAGAGAGCTCGACTTTCTCGTCTTCGA
GGTTTGTGACAAGCCTGCAAATACGGGATGTTGCTCTCTTTCTTAGCATCGGAAAAAGAAAGAGAGGGACAGAAACAGGC
CATGTGACAGCGATGGAAAATCCCTTTGCCATGCTTGTCGAAAGCCATTCGTTCCTCTTTTTCTGTTCCACAGAAAGGAA
CAAAGAGACAGGCAAAAACGCGAAAGCTCCAGCAACGAAACAAGACAAAGCCATTTATTTCCCAAACAAGTTCTGAGAAA
TCTGGTTCAAAAGATAGGGTTGTTCCGTCTTCATCATCCATATCGTGTATGGGCTTTCTTCGACCATAACAAACGTCCTT
TCGCTCCATTCGTTCAGGCCGAAATTGCAGCTCCATCCAGAGTGAGATTGTGATATCTCTCTGCATGTCGTTACGACATG
CGTCGTTTCTTTTGCTCTTGTCTGATATTCTATAGAACTTGAAAGTTCTGACTGGAAGAAAGGGACCTTTGTTTCTGATA
TTATTTTGCCAAAGTGCGAGCGAAACACGTCAACGTTCCCGACGACGCCAGAGAGTTTCACAGAGAAATTTCGACAACCG
ACGATGGAGAGAGTCAAGAGTTTCTGCTCGCAATGTACCACAAAATTCTCAGAATCAGAGATTAAAACTTTCTTGAACCC
TTCAAACTTTGGAAGGTGAAAATTCTGGTTGTTTATACCAGAGAAATATATTATCGGATCTGGAGGTTCGATGGAAATCT
TTGAAATGATGTCTTGAACGTCAATGTCCTCTTTCTTCATTACTTGTTCCTTGAAAAAGGCGCTTCAAACATATCGAACT
TAAATTCCGTTCTCAACGTCTATAAAACAATAAGATGCAGCACCATCCCTCGCACTACAACTTTCGCAGCCACAACGATG
TGCTCAAGGAGTTTTATCAGAAGCTCAAAAAGTCCCATTGGGTTCCTGAAGAGCTCGACCTTAGCAAGGAGATGGCGGAC
TGGACAAGACTGTCAAAAGGAGAGAAAAAGTTTGTCAAGCTCATCCTCGCGTTCTTTGCCCAGTTTGATGGCATCGTAGT
TGAGAATCTTGTCCAGAACTTCCAACAGGAGGTGTCTTCGTTCGCAAAGGAAATAGAGTGGTTCTATATCCTCCAAGCGG
CTAACGAACTTATCCACAACGAAACGTACATGAACCTCATCGATAACCTCTTGCCCTCACCACAAAAAAGAGAGAAAGCT
CTCGACGCCATAAGCAACTACGCTTCCATTCGTCTCATCGCCGACTGGGTGGAAAGGTGGATGGACAGAAACGTCCCCCT
TTCTGAAAGGCTCATCGCTTTCGCTGCTTTGGAAGGAGTTATTTTCACTGGAGCTTTTGCAGCCATCCACTACTTTACAA
TTTCAAACAGACTGAAGGGTCTCAGAAAAGCGAACGAGTGGATTTCGAAAGACGAGGCTCTTCATCAAAAATTCGCAGAG
ACGGTATATCGTTACATCGTGGACGTGCTCGGCGCTCTTCCGAGAGTTTCCCAAGAAAGAGTCGAAGAAATTCTTGGCTC
GGCGATCGACGCTGTCTCTCCTTTCATCCATGACTGTCTTCGGTTTAAGAACGTCAACATCGACGCAGAGAGGATGATCG
GATACATGAAGAACGCGGCAGATGGTCTTGCGTTCTCCATCGGAAACGAAAAATTCTTCAACTCTGACTCCTCTCTCGAC
TACATGCTCCTCATTGGGATGGACAACAAAACAAACTTTTTCGAAGAGGTGGTCTCGGACTACGCAAAGGGCCTCGAAGG
AGGAGGCGAATTCCGCAAAGACGTAAAGTTTTAAAAATATTTTGTATAAAATATTTTATTTGAGATGGACGCGGATATTT
CCTCTTTTTCCTTCTTTCGTTCGGAGACCCCTCCCGCGAAGGAGCAAATTGTTCCCTTCCTTTGATTGTTCTTTGCTTGC
AAAGAACTTGAGCCTTTCTCCCCCAAAAAATTCAAAGTCGAACGCGTTTGTTTTGTTTGGGATGATGTCCACAGAGATTT
CAAGGTCGAGCTCGCCGAGCCTTTTGTAGAGCGGATGCTCCAAGATGTAAATTTTGCAAACGGACAAACAAGAGGAGAGA
CCATAAGTTTGTCCGTCTCGAAGTTGTGGTCCAAGTTCCAAGCAAAGTCTCTTTTCCAACTTTGTCTTTCCTTTGCCGAG
ACAAAACTCGCAAGTCCTTTCCTTCTGGTGTTTGAATCCATTTCCAAAGCACGAAGAGCAATCCTCTCCAGAAAATCTGG
ATACACCAAAGCATTCCTGGCAAAGGGTGCTTGGGGACCTTTTTCCCATCCCCAAACAAACGAGGCAAGGACCGAAATCC
TCGAGCAAAAATTCCCTCTCCTCGCAACTCCAAACATCCGCAAGTTCAAGCTCCAACCGAAAAGCATGGACCATCAAAAG
GTTTGAAAACATGGTCGCGAAATTTGCGTGAAGGTCAAATCCTTTCGATGGGACGTAAGGAGCTTTGGATGAAAGAATTT
CATACGCTCTTTGAACAAGAAGAAATTCGTGCGTGGTGTCCCTGTCAATATTTCTGTCCGGATGAAGGGAAAGCGCTGCC
TTGTGATATGCCTTTTTTATTTGTGGTTGGTCTGCGTCCTCGGAAACTCCAAGCACTTCCCAAGGAGATTTTTCATCGGT
CCACGCGTCACTCATTTCTTTTTGAGTCTCTTCAAAGTCAAGAGAAAATATTTGGATAGAAAAAAATATTTTACCAAGCG
ACCCAACTGCTGTACGTGCCGTTCATCAAATTCTGGCAGTTTCCTCCAGACGGAAGAGTCGTATGAGGATTTAAGCAGGC
AGTGGGACAACTCATCTTTTGTGTTGCGTTGAAAAACATGGGCTCTCCTGGCCTTCTCCATCTGTAAGCACCCGACCCTC

CAATAACAAAGGAAAGGTCTGGAGTCGACTGGTTGATATAAATTCCATCAGAACCCGTTGCGTAACTGTGAGTCATTACA
AAATATCTTTTCTTGAAAAGATATCGAAAGAAAGAAAAAAATAGAAGCAAAGAGAAAATGTCGTTCCGCGTCTCTAAAGC
ACTTGTGTCGAGAGAAAAAAGCAAGCTCGCGAGGAAACTTTGTTCTCTGACACCCATTGCAACAAAGTACAATCCCGCCC
CTCAAGAGATCCGTCTTTTCAAAGCAGAGGGAGAGGAATACGTTCTCCCTCTCGCGACTTGGAAAAAACTCGAACTCCCA
AAGCCAGAAAAACGCGGTCAAGACATCAGCGGAGAGTTTATCGGAGAGCTTCTCGAAGAGACAAAACCCGCTCCCGGAAG
GCACACGAAAAGGGACCAGCAGACGATATTCGAGAGAGCGAAAGAGCTCATCGACAGAGAAAGCGTCTGTCTTTTGAGTT
TGAGCACAGGGATCGGGAAAACTTGCCTCGGAATTTGCCTTGCCTCTCACTACGGAGGAAAAACTCTTGTGCTTTGCAAG
TCAAACAAAGTCAAGAGACAATGGGTGGAATCCGTCCAGAAATTCACAAACCACAGCGTAGAGATTGTCAAGGGAGAGAC
GCTCCCAGATGCAGATTTTTACATTATGGGACCTATGAAGTGCAGAAATTTTAAAGGGGACCTCTCCTGTTTTCGAACTG
TGATAGTCGATGAGTGCCACGAAATCTGCACGAGTGTCTTTAGCGAGGCGCTCTTTAAAGTTCATCCCTCCATCCTCATA
GGTCTCTCTGCCACTCCCGACAGAATGGATGGATTGGGAGAGATGCTACCACCTTTTTTCGGAGAAAATCCCATCGTCCG
CAAAGAGAAAAAAGAGTTCAAGGTTTACAAGATCCTGACCGGTTTCGTTCCAAACAGAAAGTACGACAAAAGAGGAACTC
TCATTTGGACAGAAATTCTTCGAAGTCTCGCAGAGAACAAAGAAAGACAAAAGTTGATAACAAAAGTTCTTCAAAGGCCC
TCAGAGGGAAAGACAATCGTTCTTGGGAAGAGGAAGGCAGAACTTCGCGCTCTCTCCTCTCTTCTCGGAGAAGCAAAAGT
CTCAAACACTCTGTACATCGAAGGAGCAAAAACCTATGACGACGACGCTTCAGTAATTCTAACGACTGTCGGAAAGGGCG
GCGTCGGAATGGACGACTCAAAGATAAAAACTGTCGCAATAATCAGCGATGCGATGGACGTGAGACAGTACGAGGGAAGA
GCAAGAGGCGCAAATTCCATAGTGTACGATTTTGTGGATAAACACAGCACTCTAGAAAAACATTGGAGAGAAAGGGAATC
TTGGTATTTGGAGAGAGGGGCGACCATCGAGGTCGTGAACCTTCGCTCTTTCGTATAATTTTTGTTCAAAAAATTATTTC
AACGTACGTCTTTGGACCGACCACTTTCTCCACACAAAAAATTTGTGGAGAGAGTATGACCGTATACCTTTCCCCTTTTT
TGGCGTTCCAAGTCACAGAAGAGTGCGATCTTTCAGATAGTGGGACAGGTCCGAAATCTCCCATCGCGACTGCGTCGACG
TTCACCTGCATGGCATAATTCTCTGGTGTCCAAGTGTCTGGATTTTTCCAAGACACAGTGACTTTGTCGTTCTCCAAAGA
CGCGACTCTGACGTCCTTTACACCTCCGATGACCGCCCTTTTTTCATCCACAATTTTAACTTTCATGTCCTAAATAATTT
TCACTGATATCCGAAAGAGATGTCGTTCGGAGAGAAAGCAGGCACAGAAACGCCAGGAATTTGACCTGTGGTGGTAACAG
TCTTCGTTTCTCCATAAGAAAAGTAGCTCACAGAAAAGGTTATGGTGTTCCATTTGCGAAGAACCTTTGAAGCGTCCACC
GTTCCGTTTGTTTGAATCACCTGCTGACCGTAGCCATAATTCCAGCTACACGAAGTTCCGCCACTGGAGTTTGCCTGGCA
CACGTACGGAGCCACAGCCGTCGCCGAGCCACCCGTCAATGGCAAAGGAACAGTGGCTCCGAGTTGTCCGTTTTGCCCAA
GAGCTTGCGTGGTGGCAGTGAGTTTAAGGTCGGAAACCTCACTTCCCGTGTAGCTTGCAATGACAGAGAATTGTCCTCCA
GGACTCGAATGATATTGGAGAGTCACTTCAGGCGGACCAGTGACAACTCCAGTTTTTGTCGTCGTTCCGGAAATTCCGAT
CTGGTTCCTCGCTTGGAGGTACAGAGTGTAGGTGCCTGGAATGACAGGAGTCGGAAGGGCGATGAAATCTTGCTGGAGGG
CAGGGCCATCTCCGTTCCCTGTTATCCCTCCATTCGGGTCTTTCAAAATGTAGACATAGCTGCAAATGTAACCTGTACCG
CATCCCACTGTCGGTGTGGGCCAGGAGAACTTCCACTGTCTCGCGTCTTGGTCGTATCCAAAGTCGACTTCTGGCGGAGG
AGGGGAACCTGATTTTTTATGACAACACTTTCCCCAACAGAGCAGCCACACAAAGAAGAGAATTGCGACGAGCAAAATAA
TCAGAGCACAACCAGCCATCGTTAACAAAAGATATTTTGAGAAAAATATCTTATCTCACCAAAAACCACAACACAAGAGC
GAACAGGACAATGACCACGAGACCGATTCCTCCAAATATCGCTATTTTTTCTATCCACCCTCCGCCAACATTTGGAGGAG
AAGGAGGAGTCGGCGGCGGAGGGGTTGGAGGAGGTGGCGTCGGAGGCACTCCGCAACTCACTTCGTTTGGGTTTTCTGCC
GTTCCTCCAGAAAAACAAATGTCACATCCTTGCTGGATTGTGTCTCCAGAAGTTAGCATGGCGTTCACGCCGTCGACAGT
GACATTGTCGAAATAACACTGGCTAAATTTTGAGTCTTTTCCCCTACTGTTTCCGCAGAGTTGGTTCAGAGCTATCTTTC
CGCCAGAACTATTTTTGACATCGATGGAAATGTTGTCCAGAATGCAGACGGTGTCTGTGCATCTCTCTTCTGTGTCCGAA
CCTGGTTTCGCGAAAGGGATGGCGTTTGGAAGATTACATATCGGATCGCACGGCTTTGGAACCTTGTCAGAAAAAGGATA
CTGTGTTTCTGGCATGTAACATCCACAAATGTCGGCGTAAATCTGCCCTTGTGTCATATCCTCTCTCGACAGCGCTTGGC
AAAATCCCGAAAGAAAAGAGGAACAAGAACCTGGAAAAAGTTTGCACGTGTTTGAAAGATAATTATTTAGCGACTGTATT
GGAGGAGGTATCGTTTCGCCTGTGATAAGTCTGTAACCTCCAAAAGCAGAGGGAAGAGTTTTCTGGAGGAAATCCGCAGC
AAGTTCTTTCTTTGAGTTGTTGACATACCTGGCGCAGCTCGAGGTGCTGTAATCTCTCGGATTGGAACCGTCTTGCCAAC
CTGTCGCCGAACTGCAATGCGAAACCATCGCATCATGACAAAGCTTTGTGTCGGGGCACCACCCCGGAGCGCATTTGTTG
AGAGAGGTTTCGTTGTTGAACAGTCCAGTACAACAGGATGTGACCCTTGCTGTATTTTCTGCATTGTTCATGGACATGCT
GGGCTTTCGAAGCATACACTTGTACTTGTACGCGCAAGAAAGTCCCGAACAACCGCAGTTGTCTGTTATTGTCGAATCCC
ACTGCTTCGATGTATCGCATCCGGCGCAATCAGGGCCGTCGTCGTAATTGCATCCTGTGTTGCAAGGCTGGAGATACCTC
GCGTCGAAAGTGCAACCTGGCAAAAACGGGAGATTTTCAGAGGTCATTTACTTTTCTTTTCCAAAAAAGAAAAGTTTCTC
TGGGACCGAAACAATATGGCTTTTGTAAGATGTCCATAATCAGTTGCCCTGAACTCCCTTGTCCGGGAGTCGGAGAACAA
GGAGAAGTAGAGTATGGAGAAACCACTTGTGGAGTTTTTGGCAACAACGTTTGCAGTTTCGACGGAGAGTACGACAAGTG
CTATTCTCCGCAAGATTGTCAAACTGGAACTGCTCTCAAATGCAGCGAAGCAAAGTGTTCGAAATTTCCTCTTTGTTCTA
CAGGAAACGTGGACCCGAATTGCTCTCCTTTCTCTGGCAGACAAGTTTGTCGGATCGCACAACCGGCAATGACGAAACAG
ACAACCGCTTCTTGTTGTCTCAACAAGTATACGACTCAAGAAGCAGTGGGCGTATGCGCAAAAGGGTGGTGCGGAGACGC
AACTACTGGAACGCCTGCGTGTTTCAACGCTATGCTGACGCTCTGCTCAGATTCCTCAGATCCGAATATTTTGGATATGT
GGGCAAATAAGCCAAGTGATCCGAAAAAGGACCCAAAGTTGACAGGAGTTTGTCGAGAGTTTCTCCTTCAAAACCCCTAC
ATGAACGAAAATCCAGAACGAAGGAGAGAGTTCTTCCAAACTGTCGTCTCAAAACTCGGAAAGACACCATACCGAAAGGG
TGACGGTTTCAATGCTGTGATGGCGGACGTTTGTGGCAAAGTTCCGGGGTCTTGCGACTCTTTCCTCTCTCAAATGTGCG
GGGCGAATTCTGTGGGTCTCGGCCCCATCACAAGACAACAAACAGCCAATGACAAAGAGCTTCGAAAGCTTTGCTCTTGT
TTTTTGAGCGACCTGCAATATCCGTTTGCAGGTAAGTTTGGAGTGGAATGCGACATCCTCTGTAACGACACATCGGTGAA
TCCCATCCCAAAAGCAGAGCCAAAGGGAAACACATGGGTAAAAAAGAATTGTCAACAGAACGTTTGCATTTTGGACGATG
TGGTCATCGACATTCTAAATTCCGAGGTGGGAGGGATAAATTTGTCCCAAGTTTGTCCTTCTTCTGGACCAGGAGTGACG
CAATGTTACCTCGACGGAGTGACTGTCGATGTTGTGAACAGCAAGGTCACAGGAGGTATAAATTTGGACCAATGGTGTTC
AGGCTGTTCCAACGAAGACGGAGCTGGCATCTCTTGCGGAAAGGGAGGAGGCAGCAACAACGGAGGCGGCGGAAGCGGGA
GCAGTTTTTTGAAAGACATCCTTTTGTTTGGAGGGATTGGTCTGATACTTTTGCTCGTTCTCGGCGTTGTCGTTTGGTTT
TTGTTCAAGTAATGGCGGAAGTGTGGATGAACAAATGGTCTCTCGTCGTAGAGACAGAAAATTCAAAGTTGTGCGTGACA
AGAAGCAGAGGAAAGGAAAAAACCGCTTTTGTGAAGGAAGGGAAAATGCGCTCCCCCGACGAGCGCTCTAGGAGACACAT
CGCCCTCTGGAGACATTTGATGTTCAAAGAATAAAATATTTTTTACAAAATATTTACTGATGGCATCCCCAAGTATCTTG
AATCGCTTGCATCTGCTGTTCGGGCGTTCTGCCGAACGCGGCCTGTTGTTTCAAAAACGCATCGTAAGAACCTTCCGTTG
AGCCAAGAGCGGAAAGAAGTTCGTACCTCTGGGGAGTTTCTTGTTGTTGAGAAGCTTCCATAGCTTCTCCGAAACTCCCC
CAAGTGTCTTGAATCGCTTGCATCTGCTGTTCGGGCGTTCGGCCGAACGCGGCCTGTTGGGCGTCAAAAGCAGAAACAGA
GCCTTCCGTAGAGCCAAGAACGGAAACAACTTCGTGAGTCGGTTGAGGAGGCTGTTCAATCCAATTTGTGGCCCTCATTG

AAGCAAGCATCGCCTGGTCTCGAATTTGTTGCATTTGGGATGCCGCTGTTTTCGCCGTTGGGTTGACGACAATGCCTCTT
GGAATGGACATTGTCGTTCTCGGGGAGAAACCGGTCGTCTTTTTGACTCCGCAGCCCCACACTTCTGTCGGATCTTCGAA
ACCGCAGAAGTTGATACCTCTCTGAATTCTGAAATAGCCGCTGTCTGCCCAGTACGGACCCCACGAGTTTCTGACGAGCC
AATATTTGACTCCTTCGGCAGAAGTTCCCCAACCGACAATGTCAACAGCGTGACCTCCCATCGGCTGACCGAGAGGAGAA
GTGCTGCTAAACACCTTCTTTGCCGCATCAGGAGCAGAAAAGAATTGTTCAAAGCCAGAGTATACCATGTAGCCGATGGT
GAGCGGACCGTACAAGAAAATGTCCTTCATCATCTGAACCTCGGCAGATTCAGAGCTGGAAGAGTCGATGACAGGATAGA
CGATGTCGGGCCTGAACTTTTTACATCCCGGCTTTTCCTTGCAAGAATACTCCTCGATGTTTCCGTTGCACACCATCGAA
ACGCCTCCGACGTCTCTCATGTACTGGTAGGCCTCTCCAATCATTCCTCCCTGACAGACTTCGTTGGAACAAGTTCCTTC
AGAGCCTCTCAAGAAAGAGCTGAGAGTCGGATTCATCTGTTTGCTGTAGTTGCAGAAATCACAAGAGGCGAAAATCCAGG
GAGCGATGTTGTTGTGTCTGTCGATCTTTGTGCCGTTGGAGAGGGTGATACAGACTCTTTGTTGAAGTTCTTTGTCTTGT
GTGGAGATACGGAATCTGTCAGAGATGGCAGTGGCAGTCGAAAACGCCCAGCAACTTCCGCACTGTGCCTGGTCAAGTGG
CCCAGAAATAAGACCCGGCCATTTCTTCCTCGAATCAAATTCCGTAGGAAGAGATGTGACAGTCTCTGACGGCTTTGGGT
CGGCCGCAGCCTTCACCGACTCTTGAACTGCGACAGGGGCGCTGCTCAACAAAACTGTATTGAAAGGCCTCGGCTTTCCC
ATTGTTTCAGACGGAACAGATGAAGTCGCCGAAAGGGCGCATGGCTGTCCTGAAAGAGACAAATTCTCTTCTTCGAACCC
CTCCTTTTCACTTGGAGACCACAAAACAACGACCAAAAAGATGATGATGGCAATAACCAAGATTGGCAAGAAAGCGCCCC
ACATTACAAAAGCAAAAATATTTTTGCAGAAAAGAGAGAAAGAATTGTTTGAGAGGAAAGGGAAAGACAAGGTAATATCA
TGGCCGCCATGTTATACGACAAGCAATGGCCGAGGCAAATGACCACATACGGTCGAAGCCGAGGACCTTACATCAAAAGA
AAAACTTACAGCGCGAACGCGAGGTACACACGTCCGATTATGAGCTATGGAATTTTGCTCTATACGTTTCGTGGTCCAGA
ACCAGTCTTTCTTCTGTGTCAAAGGAGGCACACCATCGAGTTTGTTGAATTCATTCTCTCAAAAGTTCCGAAAGAAAGAT
TGCTTAGTGCTTGTTCTCGCCTCACCGAACAAGAAAGAAAAAGATTGAGAGAATGGTCGTTCGACGACCTTTGGGACGAC
TTTCTTCCTCAAAAGAATTGCAGGCTCTACATTGAGGATAAAAAGGATATGAAGATGAGGTTCGAACGAAACAGAGAGGA
AATTATTTCTGGCATCGCGGCGACAACATCAACGATCAAGGAGCCTCAATGGGGCTTCCCAAAGGGAAAGAAAAACGTTA
AAGAGTCAAACACGATCTGCGCTCTTCGCGAGTTTGTTGAAGAGACTGGAATGGAAAGGCACAAAATACAGATTGTCGAC
GACTCAAATCCTTTCATTGAAAGATTTGTGGGGTCCAACGGCAAAATTTACGGAAGTCAGTATTTTCTCGCATATGCCGA
AGAGCAGTTGAAAATCCAAAAGAAAAATTACGACGGAGTCTTGACAATCTCTGAAGAGATTGCTGACCTTCGATGGACTT
CTTTCGAAGAAGCAAAAAATTATCTCTCGAAAGAAAAAATCGCTCTTCTGACAAACGCTCTCCTTTGCATTCGCTCCTTC
AATGGAGCAGCATAAAACATATCTCACGCAAATATGTTTTCTTGTAATGAGTTTTGTCTTGTTTGCCGTGTTGGCTGTGG
GGTTTATCATAATCCTTATTTTTGTGCTCAACAGAAAGCACACTCCCACAGACCCCACAATAGTGGCACTCAAGGAGAGG
TTGGCTGTCCTTGATAAAAAATACCTCTCCTTGGACATTCGAGAGGTTGATAGGGGTGCGTACACTGAGAACAAAAAGGC
CATTTTTCTTTGTCTCAAAGACCCAGAAACCAAGCAGTATTACGACATGAACACTCTTGTCTATGTTGTGCTTCACGAAC
TTGCGCACATGTCGAGCGTAACATACGGGCACAACCAGGAATTTCATCAGAACTTTGCTCGTCTTTTGAGACAGGCTGCC
CAAAAGCAGGTTTTCGATCCGAGCATCCCCATTCCGACAAAATATTGCGGCATCGACTCTGAACATCCCGGCCATTAGAA
ATCGGCCTCTCGAACGATAAAAGAAGATATTTGAAAGACCTCAAATATGAACTCTCTCGAAAATCTTTGTTTTGAAAAAG
TCAAGAAACTCTTTGTTTCGAAAAAGATAAAAGTGGAACAATTCAAGGTTCTGCCAGAGAATATTTTGGAGAGAGTTTGC
AGTTTCGTGCCTACCCCGCAACAGGTGCGCGCTCTCGGTCTTTGCGTTCGTTGGATGAAAGGAGTTCTCAGAGAGAAGGG
ATTTTATGTTGACGGAAAACTCGAAGGACAGCTTATCAAATGGGACAACCAAGGGCGAGTTCTCGAGAGAGCAGAGTACA
AATCCGGGAGAAAGAACGGAGAGACAGTGCTCTACAAGCCTTCCAAAAATTCTGTATTTGTCATTGAAAATTATAGACAT
GGCGAACTTCACGGAGAAAAGCTTGTGTATTTTTCCAGTTCGGAACAAGACGAAGAGCAAAAGCTCAAAAAACGGAAAAC
CTACCACAAAGGTCTCAAGGAGGGAAAGTTTTTCTGGTACGGCCTCGACGGGTCCATTTTAAAGGAGCAACTTTGGGTTC
GTGGCATTCTCGTGAACAATGCAAGATAATTTTTAATGATAAAAATTATTGGAAACAACACCCAGAATCTACAGAAAACT
CCAACTTTCCCCCGTCCCTCACTTTTTCATAGCCCCTCCAGTTTTCGATGAAAACTTGTGGCTTTTTGAACCATTTGGAA
GAGACGATTCTCGGGAGGACAGTTTCGAGCTCCTGTTTTGTTATGTCTGGAGTGAGAACTAGAGGGTAGCTTGTGTCTGG
GAGAGGCACCCACACAATTTTGCATTTGGATATGTTGTATCCAAACTGCTGAACGCATAGATTCTTCCTGAGCTCGTCTA
CGTTTCCCTCTGGGAGGTCGAGCCATTTTTCATATACCTTTGAATTTCCAAACCACTTCGTCATTCGTTACACTTCTCTG
TTTTTTCCCTTAGAATGTCCACAGCTTCGTTGATATTGTCCGTCGCGGCCTGATAGTGATGGAACGCATAATATCCAAAG
CTAAAGTGCCAAACTGCCATCGCGATGAAGAGCATCAAAACAAACACAACGACACAAGGTATGGCTTGAAGCACCAAAAT
CCAGATCGCAGAGATGATGAGGAAACTGAGAAGAAAGCACCTTCTCCATTTGACAGTTCTTTCTTCTGTTTTTGAAGCGA
TAAGAATTTTATCCAAAAGTTGGGACACACTGTCTTGTTCGGAAGGAGTGCTCCCGAAATAAGCCATTCCTTTGCCATAG
TCTGCGCATTCGTCTTTGGAGCAATTCGGGTTTGGGCAGCGGATGTCTGTCATCTCTGCCTTGACCACAAAAACAAGAGC
GACAAGCAAAAGAATGTACCCGATGATGTTGAACATAGAGGACGACGCGAACCCTTTCATTGTATTACATTTCACTTTTT
TTCTCAAAGTTTACAGCAACCTCGCAACGCAAGAAAACGTTGCCAGCGCTTGAGCCACAGACAATCTGTCTGGAAACTGT
TCTACGGATTTCTTCTTTGGGTTGTGCAAGAGAACGAAATCCGCGTACATGGAGGGACTCGAATAGTATGTGGCAGTGCT
TGTTCTGTTTGTGGCGTGCAGTTGGTCCTGTTTGTTCCTTGAAAACTCGAGGCTTTGGGAAGGAATGTCATAGTTTTTTT
GCAATGTCCTTGTTTGCAACGAGTCCCAGTTTAGCATGCTGATGTGGCTCTTGGAGACGTCACTCAGAGAGGAAGGGACA
AACAAAAAAACGTACCTTTGCCCATCAGCCAAAAGACTCGAAATTTTCGCTGCGTAGACAGAATAGTTTGCGCCAGACAC
TTGCTTCTGTTCGACAAGATAAAACTGCAAGGACTGAAAGTATCCGTTCAACGTATCCATCTTTATGAGTCGAGAGCGGT
CGCTTCAAATATTTCTTTTCAAAATATTTTTTCCTTCAAAACATAGAGTTTATCTTGTCGAGCATAAACTCTTTGCTCA
ACTCTTCCGGCAACTTGAGGATGTCGCTCGAAATTATGCTCCTCCAGTTTTCAAGCACTTCCAACAAACGAGGTCTCTCT
TCGTTGCACATTCTTTTGTAGATGGCGTATCTGTTCTTCTTGAGCTCTTTCTCCACATCCACTTCTCTCCCGCAAATGTC
CTCGTCGATGGAGTAGAGAGCCGTCTTTCTGGGTACATAAAGAAAATTTCTCTGTGCAAGGTCAGACACACCACAAACTT
TACGAAACATCACGGCGATACAAAAGTCAAGACACACGTCTCTGTTTTCGCAACCGATAAAGTCGAAATGCTCCAACGAC
TTCACTTTCGACCAGTCCACGACTTCTGTCTCTGGAGTCACATTCCCTTTTTTGAGCATTGTGGGAAGAATTTCTTCGTT
CGTCTCATCTGGGAACACGAGGAAGCAACTGATTCTGTTCCTGTCTTGCATTTTCGCGCGAAGTCCCATGACAGCATCAG
GGAAAAAATCTGGTCTCATGGCAAGTCTCTTTGGGGAAAGACTTGGGAGACCAAACGCCTTCTTGATGTTTGCGAGACGG
AGAACGTTCTCCACGCTATTTTTCCCCTTAAATGGTCCCTTTACAAAAACTCTTTTTCCCGTGGCCTTCTCCACGGCGAA
ATAAGTGTCTGTTTTGCCATAGCCGCAAACAAGCTGAGCTTTGACCACAAAGTCAAAGAGGTCGGATTCCTTGGCGACTC
GCCTCCTTCGAACTCCCTTCATCCCAGAAATCCTAAAGACGTTGACGTCCTCGTAAAACGCTCTGAGTTTGGGATGACCT
ATTTTTGCTTCGCGAACCACAAACGCGCCATCAAAAACAAAGTCTTCGGAATCTGCGCCTCCCTCTCTTCCTTGTTTTGT
GTGCTTGTCGATGACATAAGAGTCCATTTCCATTGCAGGTTGTTCCTTGTCGTTTCTGGCATATACCTCTACCGTGTCTG
GAACGTTATCTGCAAGTTCGCGTTCCTGCCATCCGATGACGCATGCACACACCAAACACCACCAGCAAAGGAAAGACTCT
CTGAGACTCGAAAGTTCCTTGCACCACTCAAGAGCCACCTTGTGCACAGAAAGAAGGAATTTTTCCTCCCTCTTTGTTTC

AAAGTATTTCTCAAGAATCCAAAATATCAGAAACTCTGCCTTTGAACTGCGGTAATACTTTCCTTTCACCTTTTTCTTCT
CAAAGATTTTCGTTGCCCAATGAAAGGCAGAATATTTTGAGTTTTCGAGAGCAGAGACAAAGTTGGAACACAACCTGTTC
GTTTCATCGTCTTCCTTTTCGAGGTGAATGCGAAATTCCGTTCGCGGTTCTTCTTCTCCAAAGGCAGAGATTGTCTCATA
AAACTCTGGATAGAATTCCTTTGACACCTGAAGAGATTCTTCACTCCTTCCATGTCCGAACGCTGACCTTGTGTGACTTA
GAATGCGAACGTGTCTCGAGCGGCACATCTGCGTAACAATCCAAACAAGTGCTTCCTTTTCTTGTTCGCGAAGAGCGTTC
CACGACTTTGTTCCTGGCTCTTTCTTCTTCCTTTTTTCTCTTGCGGAGAAAAGGAAGTCAAACTTCTCTGCGAGGAAAAG
CCAGAGACCGAGGTTGCACACTGAAATCTCTTCCAGAAAAATCACCATCAAACGATGAAGGAGGTTCGCTCGAATTCCCT
CTCCAGCCTGTCTGTCTGTTGCGTCGGCGAAAAGGTCGAGTTCAACCATGCACCAAAGCGCCTTTTTGAGTTTGTTTCTT
CGAGTGTATTTTTGAGTTCCAGACTTTGCAATGTCCACATCCAGACGATGGAGAGTTTTGGCGTTCCGATATGTCGTTTG
CATTTTCACAAAATAACCTTGTGAACGAACCAACTTTGTTTCAATGTTTGGGTATAAAAAAAGATGGAAAAATATAACAA
AATTCTCTCAGAGCTAAAATTTCTCTCCAAGCTGAGAAAGGGAGAGAAAATCATGGTCAAAACCATGGCAGTGCAACAGG
GAGATTTTTTCTCTGGACTTTATCGGACATTCGTCGGAGAATCTCGAGAAGCCACGCTGGACTTTGTCTGTGATGTCTGG
GGGCGAACAGTGGATCTTGTCCAAGACGAAGAAACAACGGAAGAGTGCAGAAAGTCGCTTTGTGAGAATTTAAACGAGTC
GAGGAAGGGCGTCATCTCACTTCTTGGCACGTATGAACAAGACAGGTACTTTTGCAGTAGATTGGAAAGTCTCGTCGCGA
CGACAGAAAACCAGGTGTGCCAAAAAGTTCCAACTTTCTCTCTCTGCATAACAAAGGAAAACGCGGGACTCGCGAGGACA
ATTCAAGCGCAAGAAGCATCTCTTCGCAAAGTATAAAATATTTTGAATAAAATATTTTATTGGAACCTCTTCAGGAGAAA
AAAAGCTACGCCAAACGCAAGAAGCAAAAGAGCAAATAATAACCAAGAAGGGACTTTTTCTTCCTCTTTGCGAAAAAATT
CGAAATGTTCTCTTGGAGGCTCTTCTGCACCAGCCAATTCCATCGGATTGTTCATCCTCGCAGCAACAAAAGTTTTTATG
TCTGGCAAGGAACCTTGCGGTACAAGGGGGCGAAGCCAGCTCTTTTGTTCCTTTGTCATTCCATCGACGACGCTCATCGG
AAATCCAGAGTCGTGAAGGGGAAACGTGTCTCTCAAGGCCTTGTGAAAAGGAGAGTTAAGATATTTTTCTTGGAGTTCTT
CTGGGAGGTCTAAGCCAGGAATGTCTGGAATGCACGCCATGTTGTTTGTCTCGAGATGAGAGTAAGCGCAGTTTCTTGCC
ATCAAAAAGTAGCCGCTGTCTCCCCATTCCGGTCCCCAAGAATTTCGAACTATCCAGTACGGAACACCCTTTGGTGAGTT
TCCCCAACCGACAAGAACAACGGCGTGACCTCCCATAATTTCTTGAGTCTCTGGATTTTGGATGTGCTTATAGACCCCTT
CCTTCCAAGAGTCTGGAAACTGCTTTGGGAACATAAAGTCCTTGTACACCACGTAACCGCAAGAAATTGGGCCTCTCATG
TAAATGTCCGCCATCAAGTCTCTGTCTGTTGTGTCTTTTCTCAACTTCCTTTGGACAAAATAGGGAATCTTTACGCGAAA
TATTTTCATGGGCACTCCGGAAGGGCAATGGTCAAGTTTCTCTCCGAGAAGGTCTATGCAAAAGGGAAGTCGCTCTTTGT
TTGTGTAGTTTGCGAAGGTGTACGGAAAGCAGTCGAGAGTTGTTGTTCCGTACACGTACAAATATTCGAAACTTTCTGGG
AGAGAGTTGCCATGACAAGCTCCGTCCTTGCTAAACTTTTCGTTCAGTTGTCTTATTTTCTGCTGTGACTGGAGGTCTGC
TACAGTCGGATGGTAGTCACAAACTATCGGCTTTGAAGGGGAAGGAACAAACTTTATTTTTCCTTCAGAAAAAATGGCAA
ACCTGTCGGCCATCGAAGATGTGGAACTAAAAGACCAGCAAGCTCCGCAGTTTCCCTGGTTCATCACCGGAGACAAAAGA
CCGTTCCATTTTTTCCTCCCATCGAAAAATTCTGGAAGTTCCTTCTCCAACACATCCACAGACGAAATGCTCGGATTCGT
TGAACGATAATATTCTTCCACAGATTCTGAATCTCCTCTTAGAATGGTGAAAGAGGGTCTTGGCGTCTCAGAAGCGAAAC
GGGAGAGTTCCATTAAAAATGTCTTTGGTGCCTTCCATAAAATATTTTGTTTGAATAAAATATTTTATCTGAACAACTTT
TTCTTGAGTTGCGTCTTGGTCATGCTCTTCTTGAAATCCTCAAACGACTCAAAGTCGCATGGTATGTCGTAGTAAACGTA
TCTTTCGCTGTCGCCCATTCCCCACCATGTGAGGTCATAGAGCGAAACGCACTCTATGACAGTGCAAAAGGAACCTGTTT
TTCCATCGCACGTCGAACATCCGTCAACATTTCCGTCTCGAAAGATGAACCATCTTCCGCCTTCTATCAGTTGGCAAACA
ATGTCATCCGAACAGGTCCTGTAGATCGCGTGCGGTTTGTTCAAGTATAACTTCGTAGTCCAGCAATCGTTGCGAACACC
GAGGTTGAACTCGCAGCTCTTCCACTCTGGGACAAGACCCAGAAGGCCCATCGCGGGAGTAGTCCTGTGATTCACATCAC
AAATTCCGCTGCTTTCCCAATGCTTTGTACAGAGCAGAATTTCTTTCCACTCTTTGGGAAAGTGCTCGAGTTTTTCCATC
TCATGTATTTTGAAGATAAATACGTTTCTTTCCCTTCGATAAGATGTCTGTATATATTCACCTCGACAGCAGCTACAGAG
ACCTCGCCCAATACCCTAAGCCGAGCGACTGGTCTTTCACCAGCGCGACTTCGGCTTGGACAAACACCAGAACAGTTCAA
TGCGTAAGACCCACAAACTCGAGAACGGCATGCAATCTCGTATACAACGTCAAACTGGAGAAACTCATCATTCCTGTGGA
TGCGGCAACTTTCCCCAACTTCTTGGCAGACTATCCCTATCTTTACGTCTCTGTTTACACGGGAAACAATCCGGACGTGA
ACTCCATCAACACGATGGGGCAAGTTTCTGGGGGAGCCAAGACCAGCAAAGTGCAGTTTGTAGTTTATTGGGAAAAAACA
CAGGGAACTTTCTGGTACCACTACTCGAGTCCGATGGTTCAAAGCATCCGTTGGAATTCCAACGAGCCCCTCACCTTCAA
ACTTTTGGACCCAGCGGGAGAGGTTCTTGCGTTCCCTGATGAAGTCGCCATCGACCCCGCCGCACAAATTTCGTGTTTGC
TCTCGACGATGCCATATGTGAGAGACGGAAGTTATGACAACCACCTTGTCACTCTGTACACAGACAGTCCATACTAAATA
TTTTTGAATAAAATATTTTATGAGAGAAACCTTTGCTTGTGCCTGTTGAGGAGCTTGTCAAGAGACCGGAGAAACTCTCT
TTTGTCGAAAATTTCATCAAAGGAATGGGTTCTGTTGTCAGCTCCCACAGAAATGTCTTCTCCGTCCAACCAAAAGACGA
GACCAGAGACGTTTGCGTCTCCATTTGCTTCTCCATCAACAAACTCTCTCAAAATTTCGTACTCTTCCCTTCCAACAACC
CAGGAAATTCTACAAGAGCTTTCTCCCTTCGAAACAACGAACGTCGAGGTTCCGTCTTCTGAAAAAAGTGAGGTGCAACG
CATTACAATCTCTTGAGTTCTCTCAATCTTGTCCGAATGAATCTGTCGAGGTCATCGTACCGAATGTAGTGCGGTACGCG
GACAAGGTCGATGCCCATTCTTTTTGTTACTTTATCTTTGAAGAAATCTCTCCTTGCTTGGTCCACAAACTGTTGTGGAC
CTTTTTTGTGAAAGAATTTGCTATACTTTGCGTGTTGAACGCCGTCATACTCAAGGGCAAGTTTCAGATCAGAGTTGTAG
CAGTCCAGTTCGAGGTTCCTTCCTGTTTTCGGGTTTTTGAGAAAATCAGGTCTTATGGATGGGAACTTTTGTCCATAGAT
TTCCTCAAGAATTTGACGGCATCGAAGTTCGTGCTTTCCGTCCTTTTGTTTCACAATCCTCCTTTTTTTCTTTTGTGCTG
GGAAGAAGAAAGGAAAGCCTCCTTTCTTCCCCTTTGTCGTCTTCTGGTATTTGAAGTAACAGAATACAGCCACTCCGAGA
ACGAAAGCAAACTTTAGAAATTCGAAACTACAGAGCATCGCAAGAACGCCCATCTCTTACAACTTTGCGAATGTCGCGGT
CTATATGTAAATATTTCGAGAGAAATATTTTTTATGAGAGACTGAAAAAAGCAGAACGTGTCAAAATCGCTCCCTTCTTT
ATTTTGCGCAAAAGCACTCCGCTGTGTTTTTCTGAGAGAAGCTCGATGGCTGCTTGATGGAGTTTTTTACTTCTCCATTG
CTCCATCAACTTTTCTTTCGCCGCGCGAGACTCTTTCGAAAGAAGAAGGGAAATGGTCGGACCGATTTCTCCCTTTCGCG
TCTCAACGAGGACGTCAGACGCCTCATCATAGAATTTTTGTTTCGCCTCGAGAGGTAGAACGATGAGCACAGCAAGAATC
GTTCTGAACGCGAACGAAAGGTCTCCTGGAGGAGTTCCGAGTTTCTTTCTTATTTTTTCAACTGCGACAGAGAACATTTT
GTCAGCAGTTCCGTATTTTTTGAGTCCCTTCCTTGCGAGTTCTGCTCCCTCTTGCGAGACTCTGCCTATAATTTCATCAT
AAGAAAGTTTTTCGAGGTGGGACACTTGCGCTGTCACCGTCCGTGCAATGATGTCCTTTGCCTCTTGTGAAAAGTTTTCC
TGGATGGTTTTGGCCCATCTTTCAACAGACGAACGCACAGGTTTCGAGATGTTGCGAAAAGATGGCTTATAACATCCATA
AATGTCTTGAAGAAGAAGAGCGTCTTCTCCCTTTACTTGTTTAGTGTCCTTTCGCAAAAGCTCGGCGATGTATGCAGACT
CTACCAAAAAACAATGGACCCTTTCGTTCATATATTTTGAGAGAAATACGTCCTTCTTGTTGTCCATATTTTGGAACCTT
GCGAAATGTTGTGAAAAATCTTGTTCCAGGCGAAGAGGGTACCACCAGGAAGATGTCGTTTCGTCGTAAAGAAGACCCTG
CCTTCGAAGGAGTTTGAGATTTTTTGTGACGACTTCTCCAGAAACGTATACTTTGCCGCTCTTCCTATCCACAATTTTTT
CAAAGTTGTGAGAGGACTCCTTTCTTTGGAAGCTCATGTTACTTCTTTGGGAACTTTTTCTTTGTCAAAGAAAGAAAAAG

TAATGGGCATTGTGATATCTGTAATAATTTCGTTCGCGGCTCTTGCGATATCTGGAACTCTTTCTGCCGTCGCTGCGTCG
AAATCGGACGAACCAGCAAAGAAATGGGCCATTTTCTCCACAATTACAAGTTACGTCTCATTGGTCATTGCTCTGTTGAT
AGCGATATTCCTCCTCTAGTAGAGGAGGGTCTACAAAATATTTTAGAGAAAATATTTTATTGTTGCTGGACAAAGTGGGG
AGCGATAAGCTTCTGAAGAACGCAGTAGTTGAGCCTGTCGTCGGTCACAACAGCGCCTGTGCTCTTGATGTGAACTTTTC
CCTTTGCGACGTCCTTGCGATATTGGTCGTATCGCAAAAGTTCAGAAAGGGACTCGTCGGGAACGATGTTCTTCTTTGAA
CTTTGATCTTGGAGATTTCTTTCGCCGTTGGGGTTGAGATGTTCCCACCTCTTTCGACTCTCGAGTTTTTCTGGGTTTGT
TTCGTCGTCCTTGATGCGGATGTAGGTGCAAACAGCAGAGGTGACATCCGCCATAGAGAGAAGAGTTCCCCTCTCGACTT
GGAGAAAGTCGGCGAGTTCATCGCTAATCGGCTTGGGCTTTTTGATGCCAGAGTTTCCCTTCACCACTCGCTTCTTTGCC
TTTCCGTTGCGGATGCGAGGAAGTTGCTTGTTCACATCCACAAGTTGACGTTTCACCTTGCTGAGGAAACGGGCACCCTT
CACCTTTTCGGTGGTTTCCTTGACTCTCTTAATTTCGGCATCCAAAGCCTCGATGATAGCCAAAAGTTCTTCAGAGACAG
AATCTGAGGTCGGAGCTGGCTTCCTCACACGAGGAGGTTTCACTTCGACAATCTCCTCTGATTCCTGGCTCTCAATATTC
TTGGTGGGTGACTTGGGCATTTTATACCTTGGGTTTGTTTTCTTTAACCGTGGTTGTTGGTGACCCAAAAATTCCAAGAC
ACAAACTCCCAATCGAAAACGGGACAACTTCCAAAAGGTGGAATAAAAGCGTCAACAACAACAAAGAAAAAAAGATGTCG
AGAAACACCAAAAACGTCAAGAAAATTGCTCTCTCCGTCAGCCCTCTTGAGTTCGATGTCAACAACTTGGTCGTAAAGAA
GGTCGCCTCCAGGAAAGGAGCTGTTGCGTTTGAGATGGTGGATCTTGCGTACAAGTACCCAGGAGGAAAAGAGGAGAAAC
TTATGGTCAAGTTTGCCCGTTGTTTCACTTTCGGTCTCAAGAATTGTTACAAGTTCCAAGCAGCCAAAACGGACGAGAAC
ATCAAGCCTGGAGAGTTTGAGTTCTCAATCGTTCCTTATTCGAAGGAGTGGGCAACCAACCCCACTCCTATGGACATCGC
TCTCGAGAAGATGGAAAGGGAAATCATCGATAAGGTGCGAGCAGTCTGCGCAAAGGACGATGGAATTTCTGAGAAACTTC
AGAAGAAATTCAAGAAGAAGAATCCTGACTACAAGGATGCGGAAGATTGCGGTGTTTCTCCCATCTTTGGTTGGCAAAAG
GAGAAGAGGGAAGAGGGAGACAAGCGAAAGGACATGCCCATTGACCCCACCAAGCAGAAAATCTTCAACGTCAAGACGTT
GTCGTCTCGCGGAGGGGGCGGAAAGCCTGTCACCATGTACAGCGTCTTCCGCGCACCGAAGAAGGCAGATGCTCTCGACT
ACAGGACTCTGTTGAAAAAGACTGGAGACACCGCTGCTTGTGTTATATTTGAAGGTTTCTTTGTGAAGGGAACAGAGAAG
CTTTGTTTGCAACCCAAACTCTACGACGCTGTTTTCACAGAGAAGGCACGAGAGTACGTTCCTGCTGCGGACGACGATGA
GTTTGACGAGGAAGAAGAGAAGGCGGTCGAAGAGAAGGAAGAGTTTGATGAGTCTGACGAAGAAGAGACTCTGGACGACT
AAGAGATAATTGTGAATTCTTTTTTTGAACAAAAGGAATTATAAAAAATGTCGTTGTGCATTGGCTCTCACATCTCTTTC
TCATCAAAGGACGGAATTTGCAAGAGCGTCGAATCTGATTTGTCCCTTGATTGCTATCAACTCTACATCTCAAATCCAAA
GGCTTTCTCAAGCACAGAATACAAAGAATCTGACCTATTGTCTTTTCGAAAGTCTGGAAAAAAGCTGTTTGTGCACGCAA
GTCTCATTTATAATCTTTGTGGCTCAAAGGAAGGGGAAGATTGTCCAAAGTACAAAAGGAACCTCGAATTCACGAGAAGG
GGACTTGCTCATGACCTTGATGTTTGTGCTTTGATGGGAGCACGAGGTCTCGTCGTCCATGTCGGCGCCGCAAGGGACAG
AGAATGGGGATGCCAAAAGGTGGCAGAAACTGTCAACTTTGTTTTGAAAGAAAGGTCAAAACTCACAGACAGATATTCCG
AAATTTTGGGAAAAGACATTAGAAAGGAAAGGACGCTCCTTCTCGAAAACTGCGCAGGAGAGGGAACAAAACTTGGCAGA
GACATACCAGAACTTTTTAAAATATGGTCTCTCATCGAGAGGAAAGAACAGGTTGGATTCTGTGTTGACACATGCCACGG
TTTCGCCTCTGGTTCTATGGATTTTGGGAAAACAAAGGGTATCGACTCTTTCTGGAGGGAATGGGAAGAACATTTTCCCA
AAGAGTCGCTTCGCCTTTTTCACCTCAACGATTCAAAGGGAATTCTAGGGTGCAAGGTGGACAGACACGAAACTTTGTTG
TGTGGAAGGATATGGGAAAACAAACCAGAAGTTCTCGCTCATTTTTTGGAAGGAGCGAAAGGGAGAGAAATTCCCCTCGT
CCTCGAAAGGAAGGACAACACAGTGGAGAAGGAACTTGAGATTTGCAGGGCCTTGGTAGAATAAAATATTTTTTACAAAA
TATTTAGTCAGAGTCGGAAAACTCTTCGTCGAAATCATAGAACACGAGAGCATCGTCCACCCATTCGCACGCGTCGCCTT
CATCGTCGAAAGATTCGAGCAAAAGTCGCCAAACTTGGAGAATCGTCTCGTCCACCTTCTTGTTTTCTTCTCCGTGTCGA
CAGCCCATCAGTTGCTCCATGAGAACCTGTTTTTCTAGAGGACGTTCCAACAAAAGGCTGCCCAACACATCGTTTGCAAG
CTTTTTCTTTCTTCTTTGTTCTTCCAGAACTCTCTCGTTCTTTGGGAGTTCGAACGGAGTGCTTTTTTGACGTCGCGATG
AGATTTTTTTGAGAGCGTTGATGCGTGCTGGAGAAGACCACTTTTCTCCAAACTTGATGTGAGGAAGAACAGAGTTTAGA
AACAACAACCTTGTTTGGTACTTTTTGTTCCTCAAGAAAATTTGAGCACATGCAAAGCCCAAAGCTGGGCTGCTTCTGTC
GACAGAAGCAAGGTCTCTCATTTTGAGGTACCCAAGAATCTGCAAGAGGTTATCGATGGGAAGTTTTTCCAACATTTTAG
AGTGAAAGAAAAAACAAATGTTCTTCGGAAGAAGAGATTTCTATATTTTTTTAGAGTAAAAAATATGGATGAAATGAGCC
TCTTAGATTGGAGAAATGGCTTCGTAGTGCGTGTTGGAAAATTCCCTTCTAAGACGGTTTTGGAAGAGGCGCACGTGGTC
GTTCCTGAACTGCAACTGGTCACTCAAAAAGGCGTTGTTCATCTGCACCGTAGAGTCGACGAGGGACTGATTTCTCAGTT
CTTCGTCTCTGAAAGGTTGAAGATTGCTTTGGGTGTAGTATTGCCCGTACACAGGAAATGCGCTTTCGTCGAACGTGGGA
GGGATGCTGCAATTCGGTCCCTGTTCGTCAGCCAAAAGAGTGTAGTTGTTGTATGTCGGGGAAGGATTTTCCCATTGCTC
TCCGAGAACAGGCGGGATCGTACTCACGATTTGGGAGCCCACCAAGTTTGTTTGCGGGATGGTGAACCCTTCTTTTTTCG
GGTCCTTTCGAGAGTATTTGAAAAGGATGAGGACTCCGAGACTGATGAGAAGAAAAACGTACCAGTATTGATACCCCAGA
AAATACGCAATGACAGAGATGATGATGACGAGGCGCGTTATCGCGTTGAGCTTCGAGTCAAGGCTCATCGATGCCTTTGG
GATTATCGACAGATTGCAAAAGAGGTCGCAGAGGTCCTCTGCCCAAAACTTTTCCTCGGTGCAACAACTCATTACACAAA
AGAAGTTTATAAATATTTTTGAACAAGAAAATATTTTTATGGTTGGAGTGAACCATCTCTGTCAAACAGACAGTACAGAG
AAGTCATTCCAAATTTTCTCGAGAGGGGAGAGACTCCCCCTTTCGGTTCGATGAGTTCAATATTGGACAAATCCACGAAC
AAGAGAGCGCCTTCTTGTGAGAGGCAAAAAACTCTGTTTTCGCTCTGAGAGAGAACAGTAAAGTTTTGTCCTTTGCAGTG
TATCTCGTCCCCAGAGTTCACAAAGCTTCCAATAAAAAATCCATCCTCTGGACGATGTCCAACATCTCGCCCTTCCGACA
GAGGACTCTCTGTATTTGGGACGCGTTCTCTGTGAACAATTTTCTTTCTTTGGAATAGCCAAGAAAGCATAATCCTGGTT
CTTTTTGGAGAAAAATGCAGCTTGTTCCGTCGACGTTGAGGAGAATGTCTCCGACCTTCAAAAGTTCCAGAACTTTTCCA
AATTCTCTCTCCTCTCTTTCAAAGAAAAGAAGAACGTCCCAAAATTTGTCTGTTTCTTGAGGCTTGTGTTCTCTCGCAAA
AGAGAGGGAGGCGTTTATCTTTTCGAACCTTTCCATGCTTACAGATTATTTATGTTCAGGGAGCATTTCAAAGTCATAAG
TGGTCGTCCTCGGGTATGTTGGATGGTCGAAAGCGCAAGCTATGCTGTGCGTACAAACTTCGCAACGGAAGAAAGAAAAC
CAAATGTTCAAAAGGGATGATATTATTCCCATCTTTTTTATAAAATAATCTTTCAAGTCAAAGATTAAGCGCAACGCTTT
CTGCAAATTTAATGGACTACATCGAAGACCTTCCCGTTGACCAAGAAGAAGCGACTCCAGTCGAGATGGCGACCGCTCAA
AAATATCTGAATCCCACACGAAGCAAGAAACAAAAGTCAAAGGTCACCACAAAAACAACAAGTTGGAAGGATATCATCAA
ATGGGCTGTCGCAGTCACGCTCGTGTTTTTGCTCGTGTCGAATCCTTGGTTCGACAAGGTTCTGGGCTTTGTCCCCACAG
AATCACCTATCATCCTTTTCGCTGTCAAAGCTGGAATCTTTTTCCTCCTTTCGTTCCTCGTTCTGTGGAAATTCTCGTAA
TATTTTTAACTTTTAAAAATATTTCTCCTTCTGTTCTCCCAGCTGTGAAGCAATCCGCAGTTCCAAAGAGAACAAACATC
TTTTTCCTTTGGAACTGCGAGTTCGAAAATCTTGCACATCTTTCCCTGTTTTTCTTTTTTCGTTTGAAAAGAAAAAATTT
GTGTCTCCTTGGAGTAATGAACTGCAAGATTCTTATTGGTGTCGCGTGCCTCTTGCTTCTTTTGGGAGTTCTCTGGTTCT
TGAGAAAGAAGTCCAGCCCTTCAGGTGAGACAAAAGAAAATTTCACATGGGCCAACAACATCAGAGAAAATCCAGGTCCT
TACACCCCCACCTACGACCTTGTACAACAGGATGCTCCCGACATTGACGCGTTCGCAAACTTTGTTGAACCTGTGGGAAA

CGCTGTGGGACAAAATCAGGGTCAAGGGATGATTCCCACTCCTTCGGTAGTTCAATATGATTACAAAGACCTTCTCCCAG
ATTTGAACACCAACGTCGCGATGTACGACAAGGACATTTCTGATCCCGAAGTGTTCATGTGGCGTCCTTCCGTTCGTGTG
GACATGAAGAACAGGCAACAAGCTGGAGCTGACCCTTTCAGGGGAGACCTTCCCATCCAAAAAAATAACTGCTACGGAAG
CGGGGGCTGGTTTCAAAGTCGCTACGGTCCCGGAGACAACAATCTTAACGGTATCTTCAATGAGCTCTACCAAGAGAAAT
ACAGGGCTCTCACTGGCCAGAAATCTTACAATATTGCGATCGCAAACGAAGAAACGGTTTGTGATAGTTATCCGCAGGAG
GACCAAATGTTCGTTGCTTCCGATGTCAACTAAAGGTTGTTTTTGTCCACAAAAATATATTCTCAAATATATTTTTATTC
TTCTCTGTCTTCCAAAATTTCTCCCTTCGAACCAAGCACCAAAAGTCTCGTCTTGTATCCTGCCCTCCTGCAAGTCTCCA
GTTTTTTCAAAGTTTTCTCTTCTTCGTCAGGTTTTACCCCTCTCAACGTCCATGTCGACTTGACTTCGATGATAAGATTT
CGAGAAGCAACAAAGATGTCCGGAAAGTAAAAGCTGTTCTTTCCTTTGTGGGAATACGGAAAGCTCATGTCTTTTTCATA
GCAGCCGAAAATGTCTTCCTCTTGAACCCCCTCCGAGAGCAGAATGTCGATGGCAAAGTTCTCATATCCTTGAATCTTTA
TTGTCCTTCCGGAGGGGAAGGAATAGTCCCTGAAACTGTATGACGACTTCATCGCCTTCTCTCTGACTTTTGGGTGTTGA
GAGGAATATTCGACACCCCAGTTTTTTAAGCACGTTTCTCTGGATTTTTGTCGGAACTCTTCCACTTGAAGGGCGTGCTC
AACCCCATATTTTTCCTTCATTGTTGAGTGCATTTTCTCCTTGTGTTCTTGGTTACCGAGAGGTCCACTTCTGCCGTACT
TTTCAAAGAGAGCATCTTTTCCTTTCTTCATGTTCTCTGGGAGACTGAGGCACGTGTTCACCCCATATTTTTCCATGCAT
GTTGCTTTCGTCTTCTCCACAATTTCAGGATTCTGGGCAGAGTGTTCCACCCCATATTTTTCCATCATCGTCTTTTTCTT
CTTTTCCTTCACTGCTTCCGACTTTAGTGGGTTGGTCACCCCGTAGTTTTTCAAAAGCGTTTCTTCCATCTTTTTCTTGA
CTTCTTCGTTCCTCATCGGAACTTCACAACCAAACTTTTCCAAGTTTTTCTTCTTTGTCCTTTCCGGTTCACATTTCCGA
CATCCTCCCCAGTTATCTTTCTGCGCTGTTTTGAGAGCGACAAAGTTCCCCACTGTTCCACAGTGGCAAGTGTACTCGAC
TTTTGTCGAATTATTCACATACTCCCCCGTCATGATGCATCCTTTTGACTCGAAGTATTTTCTGGCCTGTTCCTCTCTAA
TACCTAGACCTCGAATTTCTTTCCCTTCGTCTTTGTTTTCTTCCTTGGTCAAACGTGTCTTTTCATTCCTTGCTTTCGCT
GCGCATTCTTTGCAACCTGCCCACGCCGCTCCTCCCTTATTCCCTTTCGCGATGCGGAGCTTTACAAAGTTCCCTTCGCT
TCCGCAATGACAGGTGAACTCTGTCTCGACTCCATCATTCACATACTCTCCGGTCATGACGCACCCTTTTGATGTGAAAA
ATTCCATTGCCGCTTCGAGGGAACTCTTTGCTTCTGTTTCTCTCTTCTTTTCCATAGTCTCCAAAGAGGAGAGTGGGCTC
TTTCCTCCATATTTTTCTTCGGGAGTTTTCAGTCTCTTTTCCTGGTTCTTCTCGAGTTGAGAGACATCCTCGACACCGTG
AACAGACAAGCATCTCTCTTTCTTGGCTTTTAGAGAACAATCCTTGCATCCCGACCATGTCTCGAGATTCACAGAGTTCA
TCAAAGCAAAAAGTCCCTCCTTCCCGCAGTGGCATGTATATTCGACTTTTGTCTTTGCATTCACGTACTCTCCCGTCATC
GTGCATCCCTTTTCGGCAAAACGTCGTCGAGCCTCTTCTGTTCTTTTCGCAAGAGACATCTTTCTCAGAATATTTTTATC
AAAGAAAAAATATTCCATTCATCCCACAAGGCAAGCAAACTCTTCCGTTCCTTCTGGGAAAATCCCCTTTTTCGCATCCA
TGAACGTTCTCCAACATAGAATACTTCTCAAAAAATCGTCCTTTCCTAAAAGTCCAAGCATCACAGAGAGCTTCTTGGAT
GTCTCTAGATTTTCCTCTTTGTTCATCATTAAGCTTTCAGCGCAAATCCAAGCTGCGCACATCGTGTCTTTTCGTCGATA
GCGAGGGCGAACGGCGTGGTGATGAACGAAACCGCAGACGAATTCAGGAAGGGACGCTTTCCTCTCCTTCAAGGATTTTC
TTCTCGCGTTTCTTTCACCATCGCCAAAAGAGAACGCCCTCCATCCGCACTCGGGAATAATTTTTCTGGCTGATGGACCA
AACTCTCCAATGTTTTTGCAGAACAAGAAAAACTCCCTTTTGTCTTTTTTTAAAAGTCTCTCAATATGGACCCGAAGAGG
AAGAAGGAATGTTTTGTTCGCGCACTCGACGACAATTTCTTTTGTCATCTCTGAAGAAAACTTTGGGAGACAAGCAAAGG
CGTTTCTCTCTTGAATGAGGCTCTTCAGAGCCTTTTCTTCGCATCTTTTCTTATTGTGGAGAAAGATGCCATAGAATGTC
TTGCAACAAAATGCGTAGTTAAACTCTTCCACAAACCAGAAAATGTGCTCTTGCGTCTCTTGGGGAAGCTCAAGAAACTC
CATCGAAAAAAACGAAAAACATCTCCACGAATTTCCTTTTATACAAACAATGGAACAAACAATCCAAAGAGCAATCTTTT
CTATCGACTCGAAGCGCAACGCTTCAGAGTATGGCGTTCCCACTCTCGATGCCGACACATGCAGAGAGCTCATCGACTCT
GTTCTTTCATCCATTCCAGAGGACAAACAGGTAGATTCTGTTCTTCATTTGGTTTCTGCAAAGGAAGCTCTTGGAGATGG
TTCTTTGGACTTTGACTCTGCGAGATACTATCTGAAGAGAGCTGTTCGGGAGCTCTGTTGGTGAATATATTTTTTGACAA
AATATATTTTTCTCGTTCTCGCAAAGAAAGAAAGCGAACGCTTCATGTCCCATCCTAGCAAACAGTAAAGGAGCGAAATG
TCGTCTTTTACAGAGACTCTGTCCCCATCTTTATCAATAAAAAATATGGAAACTCCATAGACCCCAACAGTCAACTCAAC
ATCTTTCTCAAACTCGTAAAAATCTGGGTTTTCGTGGTGGATGCCAACCATGTACGTATTCTCTTCAAATTTGAGCTTCG
CCCCCAAAACTCCAAACACTTTCAACCACTCGATGCTCAGGTTCTTCCTCATTAAAATTTGGAATGAAATCTTTCCTCCT
TGTGTTGAAGAGACATTTTTTGACAAATAAAAATATAGAATGTATGTGGCACGCGGAACACCAAAGATTGTCTCCATAGA
CCTCGACTGTAGTGACGACCCTTTCTATCGCTACAAGATGAGACAGTTGAAGATAACAGATTCTGGAAAGTCGAGGACCT
TTTTTGACAACATAAAAGACGTCTCCAAGGACCTTAACGTCCCTGCAAATCTTTTGCCAAAGTTTCTGGCTCTTGAGCTC
GGTACAAGTTGCGGCTTTGACAAAGGTAGAAATTTATGGTACATTACAGGGTCTCACAGCACAGAAACCATTCACGGAAA
AGTTGGAAGTTTTATCCAGAGTTTTGTTCTTTGTAAAACTTGTGGGCAACCTGAGCTTTTCTATGAAACAAAAAAAGGAA
AAGTGATGATGCGTTGTAGGGGATGCGGAGATTTTTCCTCTCAAGAAGAGCATGAAAAATTTTGCAAGTTTATGGTCAAA
GAGCTCCAATCGAACACTTGAAAAAATAATTCATCCATATTCTTTTGCTCAAAAGAATATGGATGAATTACCCTTGGAAA
TTTCATGTCTCGTGTTTTCCTTCCTCAGCATCAGAGAAAAAACGGCACTTTCTCTCACTTGCCAAACGCTGAATTCAGCG
CTCCACAATCCATGCTCGTGGGAAAACGTTGCTGTTCCGGACAGGTTGCGTCTCTTGAAAGGAAACACCATAGAGAGACT
CAAAAAGTACAAAAGCGCGAAAGAGAAACACGCAGAGCTCTCTTTCATCCCTTTTTTGCAATCTCCTTTGGACTTGGGAG
ATGACGAGGTGGAAATGCTCTGCAATCTCGCAGAGCTAAAGAAACAAAAGTCCCCTTCCGCATTCGCGAGGATTTGCACA
TCAAAGTTTGGAGAGATTCCAGAGTTTCTTTTGTGAGAAGATATATTTTTTGGTGTCAAAAAATATTTAGTTGTTATACC
TCGGGAAGAACATCGAGGAGAGAGACGACGAAGGGCAAGCGTAGCTCTTGACCGGTTGAGGGGGAAGAGAAAGGCCGTTG
GACTGGCAAGCCATCTGGTCGTGAGTGTCCGAGTTCATTCCCGTTTGGAACCACCAATTCGTGGGTTGTTCGCCGCACTG
GGTAGGGAGGGGTTGCTGACGACCGACGCCCATCGTATCATACATGTACCTCTGCGACATGTCAACGGCGATCACAGGCC
TGTCGACGGCGTTCTCGCGAGCGATGAAAGCCTGCAAGTTGTAATTGGGGTCCACAGCGTTACAAGAACCAGCTCCGATC
TTCTTCAAACTGTAGGGGTCCGCCATCCTTCCATACTGGTCTCGGTTGAACGCGAGAGGGTTAATGTTACAGAGTGAGCC
ACTGGCAAGGTTGAATCTTTCCGACCAAGCTCTCTCTGCCGACTGGGGGTCAATCTGCGTTGTATAAAGCGCCTTCTGAC
TGAGCACTGACGACATCTTACTATCCCCAAAATACAAAGAAAAAACTTGCAAAAAATATTCAAAGGATATTTTTTATTCG
AGACGAGCCGACCATTTTGACCCTGCCTCTGAGACGCAATTTCCGACCACTTTGTCGTTACATTCTGGTGCTCCCTCAAA
AGTGCCATTCGTTTCATAGCCTGGGTTTGACCTTTTCTTTCTCCAACGGTCCACGATAAACCCTGCTGTTTCCTTTTTTC
CGTCCTTGACGTTCTGGACGACGAGGAGTCTCTCTCTGTCGAAAAAGTAAGGGAGCGTTGTGTCTGGATCTGCTGTGCTT
CTCATCGTTATTGTGGTGTCTTGAGCCTCGTAATTTTTCACCCACACTCCCAGGGCTTTTTCTGTGAGGAAGACCACAGT
GTTTTGCAAGATTCTGAAATCAGAGACGGTTTTGTACTGTTCCGTTTGTATCTTTGCAAAGGGAAGATTTTTAATGGCGG
AAGAAACGGCAAACAACAGTTTTTCTACAGAAGTTTTTGTGGGGACTCGAAGCTTTCGGTCAGAGGTTATAAGGGATGAC
GGGTCCGTTTTGGCAGCGTCCACATCAAACTCCCCATCGACAACAAAGTCATCTCTTCTCAAAAAGCGCCCCAGTTGAGC
CGACAAAATTATACCATATTCCACGAGCCTGGAAGCCGCCGCTCTCTTTGTCCTGAAAATTTGAAGGGACGACTCGCCAC

GAGAGAGCGTGATCGTGGTCGCTTCTGGCACCCCTTCTATTTTTGGAGAAGAGACGAGAGGCACAAAACAAAGCTCGAGA
CCAGTGCCTTCCGTGTCGAGATGGACGCCTGTCGTTCTTTTTTCTCTATCGAGCTCTTGGGAGAGAACGGGAGCGCCAAG
AGCCTTTGAAAGTTCTCTCGCTTTCGAAACTCTCGTCAGAGAGGAGAACCTTTCCTTTTCTGGGATATCCAAAGGACAAA
TGGGAGAGATGACCAAAGACGTTCCTCCTTCAAAAAAGAGCGTTCGCGTTTTACCGAAAGAGTCGATGTATTGCGAAGTC
GCAAGTTTCAGAATGGAGTGCTTTTTCGGGTCCTTTGGTTCGACGGGGCTAGTTTCCAGACCTTTCCTGTCTACGGACAC
AGACCAAACTCTGTTTGTGGAGTAAAGGCTGCTAAAAAGTTTGCTGATAAATTCTGGGTCCCTGAAGGTGAAATCTTTGT
GGTTGATGGAAATTTTTGACTTTGGTTCTCCAGGCTTCTTTTTCTTCCCGAGTCCGTCCGTTTGTTCTTTCGGGACGGTG
AAGAGGACCTCGTACTGCGAAGGGTAGGGCATGTCCCTCCAAGGAACGCGCAAGAGAACGACCGTCTTTTTCTTTGGGTC
AAAATATGGAGCGATCCACGACTTTGCGTGTCTCGGAATTTCGAAAGACGCCAACCTGTCCTTCCCCAAAGAGAAAACAA
CGATGTTGATGTCATAAAATTTTTCAAGAATTCTCACAAACAAAAGAGAGTCAAAGAATGTGGTCTTCTTGATGAGCTCT
CGGATCTCTTCTGTGCGTCGGAAATCATAGAGTTCCTGCCTTCCAACGCTGAAGGTGTCCATTTCAGAAATTTCCTGTCT
TCTAACCTTGATGTACTGAGCTCTTTCGGACTCTTGCTCCAACGCATAAAATTCCTTGTTTCTTGCCGTTTCGATACAAT
AGAGCATACTGTCCGGAGCAACGTCGACAGAGTGTCTCAAAATGGTGACAGGAGACTCTGAATCCATCACAACCTGGAAA
AATCTCTCCAAAGAAGAAGGGATGAAGCCGCAAGTGTTTTCTGGCAACTCCTTTTTATCAGCGCCGAAAACTCTCTCTAC
GCAGCCCTCTGGATGTCTCCTCGGTTTTTCTGACGGCTCCGTGTAACAACAAGGGACAAATTCGTATTTTTCAGAGAAAG
GTCTGACGTTAAATCTCAGGGCAGGGTATTTGTGGTCTTTGTGCTTTTTTGACTTGCAGCCAAAAAACATGCCGCTCCCC
TTTGGATACTCTAGGACTCTGTTTTTTTGTCCTGAGCGTTTGAGCTCTCTCGCTTCTTTTTCTGAAATGACAGAAACAGC
GGCAGTGCATTGTTTCGAATATTTTTTCGAGAATAAATCTGGATATGTTCTCCTCAAATTCACAATGTTTCTAAGCTCTT
CGTCTTCTGCGCCTTTCCTTATTTTTTCCTTGAAACGCTGGGTCGTTGGAATGAACTTGTCATAGAGCGACGTGATGCGG
GGAAGTTCTTTCTCGTAAAACCCAAGGACAGAAAGGAAAAGGTCGTTGAAAGCCTGGATTTCACCCTCTGATTTCGCCCT
CGCAATGCGAATTTTTGTGTCTGTGGATTTGCTCGATGCAGGGAGGAACGCAATGGAAATATCGGAAGGGTCCCGTTTTT
GCATTGGGTCAAAGTAGACATAGAATCTGCTTTTTGAAAGTGTTGTTGAAACTGTCTCATTGGAGAAGAAGAATTTCGAA
GTGAGAGGATTTCTCAAGAGAACATCCACAAACTCTGCCCTTTTGTACGCAAGGTCGTACACGTAATATGAGGCTTTGAC
AGCGGAAGTTTTTGTGTTTTCGAAAACGATGGGTTCGCCTTGTATCGCGTCAGAGACTCTCTTCTGGACGTCAGAATATG
TCATCCCTTCGCGGATGGTCACATCCACAAGAACATGTTGCTTTCCTTGCTCTGAAGAATCCGGCGTCCATATCACCTCA
TCATAGAATTTGTCCAACCTTGCGAGCTGTGAGGGGTTGTTCTCTGAAAAGTCAGTCGAAGCGAACGACAAAACTTTCAT
CAAGATGACCTCTCCTGTTCCGAAATCTTCGAGACCTCCCCTTCCAGTAAGCCACGATTGTGCTGGGGGAATTCTCGCGT
AAGATTTATAGAGATTTCTCCCAGAAGTTTGGATAGAAATAAAAGGCACATCTTTCGAACACTGCATCCTGTCAAAAAGT
TGAAGTAGAGAATCTCCCGTGTTGACTGTGGACAATCGCGCCTCTCTTTCGACGCGATAATCTCTCGCCTGTTTTTGGGG
GTTTTCTGTCAGCTGTGATGCGGAATTTTCATAGTCATCGACAAGTTTCTGTAGGCGCTCCCTTTCTGCTTCCACGTACT
CCTTGTACTCTTTGATGCGAGATTTTGGGTCAAAGTATCCAACGTCCAAAAATTTTATTCCTTTCGTTGAAGTTGCGTTG
TTCGCGACAAGCCAAACAAGAGCTACTCCAGTTTTTTTCTGTCTGAGAGACCAACGTCTCTTTGTCTGTGTTTTGTAGAC
TTTTTCGAGGTCTGTCGCTGCGACTCCCTTGAGCACACTTCGAATGTCCAGGATTTTTGACGGGTCTTCCCGAATTATTT
GCCTGAGTTTCAAAACGTCATCGGTGGGAAAGATGAAATATTCTGGTATCGCTGCCCTCTTTGAGAGGGCGTACTTTGTC
TTGACGATGTCGAGCGTGTCATAGTCCCGAAATCCAAAGGACGCCATTATTACAACTAAGTCACAAAGAAAATATTTCTT
GAGGTAATGCAGGCATGGATATGGTTCGTCGTGCTATTTTTAATCGTTGGAATTGTGGCTGCCCTTGGTGTCGGCTGGTT
TGTGCTTTGGAAAAAGGATGAACCTCAAAAACGGACCGCAGAAAGCGTAAGATTCCCTTCTTCCGATGAAGTGGTCGACG
TCGCTGATGGGTTCGGAGTTGTGTTTTCAAACAAAACACGTCTCCCCCTCAACATCAAAGCGATGGGAGGAAAACAGTTT
GACCTTCCTCCCCTTCAAGAGACGATGGTGTTCCTCCCTGATTTTGAGGACGTATTGGTATTTTTTGACCAGATACCAAT
ATTTGTTGTTGACAAAAAACTTCTATCGAACGGAAAGGTTTACTTGGGAGCGACAACGACTCTCAACATCATCTACGAAG
ACATGAACGGCTTTTTGCCTGTGATGGACATTCCTGTCCTGAGAATTCATAATCTTTCTCTATTCCCTCTGACTTTTGGA
AATGTCCAAGTGCCTCCAAACACCACCGTTTCGTACAAGGGAACAGAGGGGAACGGTATAGCGTTTGGGACGGTCCTCCA
GGACTATGCAGGAATTTTCAGGTCTGTTGTGGTTCAACAAAAAATATCGGACGTCTATTTCGGCATCGTCTCTAACAAAC
TCCCCGCTCTTTTCTCCACACAGGCCCACTACTAAGTTTTTTATTATCTATAAAAAACAAGGAAATTCGAGAGAATACTT
TTCTTCTCCCTCTTCGGTACAAACGACCATCTTTGTGTTGTACCCAAGTCTCGCAGACGCCCACAGCTTTTCGTACATTT
CTGTTTTGTTCTGGCGAAACCAAGCATCCGTTGAGACAGAAATGCAGACTCTTTCAGCCTCGTCGAGAACGTCTGGAAAC
CATTCCCTTTCTTTTCCCGCAAAAGAGTACCGAATGCCTTTCTTCGCGACCTCGGCTTGGGGTTTTGGTAATTTGCAGCC
AAACCAAAGCCTCGAAACAGCTCCAACACAAGAGCATCCCGCGTCGTCTTTTTCGGAACAGTTTTCACACTTTCTGCCAG
ACAAAAACTCGAGTATTTTGCTCTCAGCTGCGTGGCCGCAGTCGCAGATGTATTTGAGCATCTCCAACTCCCTCGACACG
AAAACGCAACCCCCCTCTTCGACAAGCTCTTTGAGCTCAGAGTCCGAGAATTCAGGAACAGAGCCGCAGTTTTCGCAAAG
AGAGGGACATTCCATTTTTTGGAGAGCCCCTTTGCCGCACACACAACAAAAACATTCCACTTCATCAGAAAGGGTATCGC
AATACTCTGAACGCGCAACAAAGCCAAATCTTTGGAAGAAGCTCGCACACCTCAACATTTCGCAGATAAAAATTTGTTGA
AAATAACTCGACGACAGTTATTTTTTATACTCTGAAAGGAACAAAAAAAGTCCTATGTTCTGTCGTGAAAAAACGGTTGG
AAAATACATCTCTTTGCAAGTTTCTTTTAGCCTCGTCTCAATGGCTGAAATTTTTTCATATTCATCTTCAAAGGAACGAG
AGAGCATCTGAAAAACTTCGTGATGATGCTCTTCGTGAACGTCGTCTCCGTAAGAAAGACTTTCGGCGAGTTTTGACATT
TTCACTTCTTATTCAAGAGGAAAATATTTGTTGTAACGTAATGAGACAGGTCAAAAGACAGGTGAGGCAGGAACCGCAAG
CTCCTCGAACGACCGGAGAAAATAAAGAGTTTTGGGGTCCATGTTTGTGGAGGACAATTCACAGTTTTGCTGCAACATAC
ACTCCTCAGCAAGCGCAAGCTTTTTTGAATTTTATCACGGGTCTTCAGAGCCTTATCCCCTGCGTCTCTTGCAGAGCAAA
TTTCAAGAAAAATCTGACAGAGCTTCCGCCTCTTCGGGGATACTTGGACAGCAGGGAGAAAGCGTTTTATTGGACGTACC
TTCTTCATGACAAGGTAAACAAGGAACTCGGGAAAAAAAGCCCTCCCTTTCAAAAAGTGAGAGAAGTTTACTTTGAGCGT
CTCCTCGGAAAAGGAAGTTGTTCGACTTGCAGAGGATAAAGTTTTTTTATTTTTCCAATAAAAAACAACAATAAGACGTT
CGTTGTCTCGGTCTTAAGGTCAAAGGAAATGGACGCTTTCAAAGTCGCCCACATCGCAACAGAGATCGCCGTCATCGGCG
CTGTGTACGTCATTTTGAAAAACAAGGTAAATGCCGAAAAAGCGGAAAGAGAGCTTCTTGCGCAGAAGGTGCAAAAGCTC
GAAGAGATGTCAAAACAGCAGATGGAAGCTCTGAAAATTCTTTACGCCAGAATAGAAGAGAATGAGAAACAACAGGGAGT
TTCTGGCTCGTTCAAGGAAAAAAAGAAGCGTTTCGAACCTTTGAAAAAAGCATACTCTGAGGAAGAACCAATGAAATACA
CGAAAGGTGCAGGTGTTTTCGCCACAGAAGCAGACCATCAAGAGAAAGAAGAAACTCCTTCTGGCTACCAACCTTTGGAA
GAAGACTACGAAACTCCTGAACACAGAATAGGGTCTTCAAATATGGAAGATACAGACTAAAATGGACGAGACTTGGTTGA
GAGAAAATTCTGGAATTGTTCTGGTTCTGTATAACCAGCTTATCAACGAACTCGAAATGATGGGACTTTCAAAGCTTTTG
AACAAACCCGACCTCGGAGACTTTGCAAATTTTGTATACCGTAACTCCGCCTGATTTTCTTTTGCATAAAAGAAAAATAT
TTTCCCCGGATGGTAATGACCGACACTGGCAAGTGCACCGACAAGGCTATCGCTTCTTGCAAAAAGCAGGGGAAACTCTG
CAATGTTGACACTGCGAGATGTGTCGTGGCTGACCCAAAGAGAAAAGTAAAGGGAGCCGTCTACGACAAGAAGAGGTCTC

TCTACTTTCCGCAAGGACTTTTGTTTGAGGATGACGATGGTTCGCAAGAGCTCGTCTCTCCCAAGAAAAAGAAACCCTCG
CCGAAAAAGGCGACAAAGAAAAAGACCTCCCCCAAGAAGCCCGTCGCGAAGAAGAGGAAAGAACCCTCTTCTGAAGAGGA
GGAATCAGAGGAAGAAGCTCCCAAGAAGAAGACAGTGAAAAAGAAGCCTTCACCAAAGAAGAAAACCCCGGCAAAAAAGA
AGCCCTCTTGCTTTGAGGAAGAAGGAGAATGCCCCGATGAAAAACCCATTTGCAGTGTCTCTGATAAGGGAAGGGGTACT
TGCATCAAGGGTTCACAGAAAACTCTGGCTTCAAAGTCCATGATTAAACATAGCGGTCTCACCATCGTCGGTTCTTCCGA
TGTGCTCAAGAAGTTGAGGAAGGATTATGAACACCTCGAGGGAGATATCAAGAAGTGGAAGGAAGAGTCTGAAGAAGAGG
AATCTGAAGAAGAGGAATCTGAAGAAGAGGAATCTGAAGAAGAGGCGCCCAAGAAAGTGGTAAAGAAAAAGACTCCAGCC
AAAAAGGTTTCTCCCAAAAAGAAACCTTCACCCAAGAAGAAGTCGCCTGTCAAAAAGCCGTCTCCCAAGAAGAAGTCAAA
GAACGAGGAGCTTCTCTGCTTTGAAGAGGATGCAGAGCCCTGTGATGAAGGAGAGGCTTGTTCCGCCACAGGAAAATGCG
TACCAAAAACGGCAGCTTTCTTGAGAAAGAAGAAGAAGATTCCCCTTCACGGCAAGGAGGTGTACGGCACAGAGCTTCAG
TTCAAGAATTTGTCAAAGAAATACAAGGACGTCGCAAAGGCGAGGAAGTACGAGGTGGGAGAAGAAATTCATATCGAGGC
TCCGAAGCTGGCTTCACCAGCCATCAAAAAGACTGCCAAAAAGATTGAAGAGGTCGTCGAAAAGGCCGACCTTTCCGAGG
AGGTGAAACAAGTCATCGCCGATGGCATCGAAGCTGCTCTTGCGGAAGGTCACAAGGAAGAGAAGAGAGAAGAAAAGGGA
AAGGAAAAAATGGAAGAGGAAGAACCCAAGAAGAGGGTAGGAGGAGGAAGGAAAAAGAAAAAGGTCGTCCTTCCCGAATC
CGAGTCTCGACAGGTCGAAAAATCTGCCGAAGAAATCCAACAAGAGTTTGAGAGATGTCTCGGCATCCAGGTGAGCGGTT
TGTAAAAAAGATATTTTGAGATAAAATATCACAAGTTAAATTCTTTCGTTGGCAGAGACTAATGGCTCTGCATCTCGGAA
GTAAAGATTTTACGGTTAGGGGCTCCCTTTTGAACGTCAGCACGAGGACAGGAAACGTCCTTGTGTTTTTCAAAAAGAAC
GCGTGCCCGCATTGCGCGGCGTTTGAAAATACGTTCTTGGATTGTGCTCGGTCAGAACCAGGAATTGAATGGGCGATCGT
CGACCTTGACAGGCACCCTGAAGTTATTCAAAAATCAAGACAAACCAGAAATCCCATCAAGGGCGTGCCGTTTGTGGTCA
TGTTCGTCAACGGCGTGATGAAAGCGATCCATACAAACAGAGACAGGAGTCGGCAGGCGGTGCTTTCGTTTGTGAACGAA
ATCATGGGCATCTCTGGCATAAATTTCCAACAGGTTCCTCAGATGCAGATGCCGCAAACACCTCAAAACTCTTTCATGCA
ACACGCTCAACAACAAAGGCAGAATCAGTCCACGAGTTACAGAAACGTCACTCCACCACAAAGAGATGAAGAGACCACAA
GGTTTTCTTCAGAATTTGTTCCGTACAACATTCCTTGGGCTTCGACTGAAAAGACGAATTAACAGAAAAAGAAAACACTC
TAGAATTTCTTTTTGCATTCCAAAACATAATGTCTCAACTCTCCGAATGGTTCGGCAGCAACGAAGTCGCGCAGTTTCTA
ACGAAACACGACAAAAACATTGAGGTCGAGCTTTCGTTTGGACGTTGGGAAAGAGCAGGAAAACACAAGAGCTTTATTTC
AGGCGTCACAAAAGCTGAATTTTACAATCTCTTTGATTCTTTGGATAGAATCTCCAAAAAGTCTCCAGAACTTTTTCAAA
GACAAGACGTTCACACTTTGGAAGAAACAGGGAGAAGGGTTCCTGGACAGAGAGGAAATGTCAGGCGCATCAAGGACATC
GGAACAGGTCAAGTTTCTTTCCTCTCAAAGGACAGAAACAAGGTCTGGGACGAAAGGAAATGGGGCATGCGACTTGCGAT
GTCTGTTGAGGAGGAACTTTTCGACTCTCCTGGCTTCCAACCCACAGGTTTCCGCGAGAAGAAAAGGTCGAGATTCATCT
TTGTGGGGAACAAATCCGGTTTCAAGGGTCTCACAGTCGACATGACAAAAGTTGTGAAAAGCAGCTTCAGAGAAGGCGTC
GTGTACGAGGTCGAGATTGAGAAGGACAGTTCCGTCTCAAAAACCCCGGACGCCATGGTCAACTCTGTGAAAAGAGTTCT
CGAAATGATGCAGTCCCAAGCCTCTTGCCAAACGAACGAAATTATCTCTATGGAAGAAAAGGAACAGGCTCTTTCCCTTT
TTAACCAACTGGTCGGTTCGAAGAGACACGGAGACACTTCGTTCCTCAACAAACCCAGACAGATAAAGTCGAAGACCTT
CTTGAGCCCAGGATTCTTGCCATCACGAACAAACTCGACGGTGAAAGGCGTCTGTTGTGGTTTGGCCCTCTCGGCACCTA
CATCGTCAACCCTCCATACGACATCCAAAAAATAGCTGGCGTTTTCTTCCAGTACAGGGACACAGTCTTGGACGTTGAAC
TTTATGGAGAAGAAAAAGGAAAGTGTCTGTGCTATGCGTTTGATTGCCTTTGGTTCTCTGGTCAAAACCAGGCGTCGAAC
AACTTTGACACGCGCTTCCGTCATGTCCTCGAGGTGGAAGAGAATGTGGGACAATCTGCCCATATTATCTCCAAAAAGTA
TTTTGAGGTTGGTCTCCCACTCGGCTCTTTTGAAGGGATGGCAGCAGCCACAAAAACAACAAGAGGAACATATCTCGCAA
AGGCCAAAACAGAAAAGCTTGGAGGCGATCTTTTGAGAGAGGCTGTCGAAAAGGCTCTAGAGTATGCAGAGAAAAAGGGA
TTCAGAACGGACGGACTTATCCTCCAACCGAGAGACCAGGTCTACAAAAACAGTGACACCCTCAAGTGGAAACCAGAGAA
TCTGATGACCGTAGACTTTCGCCTCAAAACCAAAGGAAAGGACGAATTCTTTTTGATGATGGGAGTGCAAGGAGGAGAAG
CAAAGTTTGAGGGCACCGCTGCAACAAAGAGAATCCCCGGAACCGTAAAGCTCCCAAAGTCGTTCTTGGAGGAAAATTCG
GGTCACGACTTTGAAGGGGAAATTCTCGAGTTTGGCTTTGACAAGGAGAAGAGCGTTTTTATCCCTCATCGCGTCAGAAC
GGACAAGGATATGCCAAATTTCTCCACCACAGTCGTGTCTGTCTGGAACGACATCTTTCGCGGAGTGACTTTGGAGACTT
TGAAAGGAGAGGACCTCGTTCTACCGAGAAGGTACAACAACATCGTCAAACAGTGTCTTTTGGAAATACCGAAAGGGAAA
AATCCCAAACTTGTGGACATTGGTCCAGGAAGGGGAGGAGACATTCAAAAATGGCTCAAGAGAGGCATCGGTTTCGTGAA
AGGTATTGAGCCGAACAAGGAAAACATCGTCGAGTTTGAAAAGAGAGCAAAGGTCTCGAGGTTCTCGTCTTTCAAACTTT
TCAATGGCAAGGCACAAAACACCCAGCAAGTCATCAAATCCTTTGGAGAAGAAAAAGTGGACGCGTCCACTGCTTTCTTC
TGTCTTGGATATTTTGCAGAGACAGAGAGGGATTTGGAAGGTCTTTGTGAGACGCTTTCTCTTTTGCTCGAGAAGGAAGC
TGTCGCGATGTTTTCGTTCATGGACGGCGAGGAGATAAAGAGCGTCCTCGGAAAGAAGGGCAAGTTTGAAAACTCTGCTT
TTTCTATCGAGAAAGGAGAATGGTCCAAGAAAAAGTTTGGAAACAGCGTCGTCGTCCACATCAAGGACGAGACGAGCATG
GTGAAAAACCAAACTGAATGGCTTGTCGACTTGGGAATGCTGAGCGCAGCCATGAAAAAAAAGGGCTTTGAGCTTGTGTT
TTCGAGGTTGATGGATGAAGGAATCTCGTTCCTTTCGACGCAGGGGTTCGAATTTGTGTCTCTCCACAGGATCGCCGTTT
TCAAGAAGTTGTAAATTTTCTTTGTCTCAAAAGAAAAAAAACTTGTAATGGAAGACCTCACCTGCGTCAAACCCTACACG
AGCAAAGACAAGTGGATTGTTTCTGCCATCGCTGGAGTTTTGTTTCTCATCGTCGCTTCCCCTTTCCTCTTCAAGATTAT
GAATGGCCTCACAGGGATCTTTGGAATCAGCATCGCCGACCCAGACACTGGAGCACCCAACCTTTTGGGTCTTGTGTTGC
ACGGAGTTGTGTTTATGCTCATCGTGAGACTTTTGATGAACTAAAATATTTTATTCAAACAAAATATTTTTTGAGAAGAC
AAGTTGTCGAAAGAAAGAGATGGAGATTGTCAGTTGCCTTTGCGTTGGGGACCCGCACTTCAAAACGAACAACGTTCAAG
AGGTGGAAAAGCTCACAGCGAAAATTCTCGAGATCGTCGAAAAAAGAAAGCCCACATTTGTGGTGGTTTTAGGAGACATT
CTCGACACACACGAAACGTACCATGAAACGCCGCTCAACAAGGCCATTTTCTTTTTGAGCAAACTTAGCGTTCTTTGTCC
CACATTTCTCCTTATAGGTAACCACGACTACTGCAACAACTCCCAATTCCAAACGACGAGACATGCGTTCAACGCGTGCA
AAAGGTGGAAGAACATGTATGTTGTGGACAAGGCAATGTCCAAAGAATTCAAAGGACACACCTTTTGGTTTTGTCCTTAC
GTTCCACCAGGAAGATTTGAAGAATGCCTTAACACCACAGGGACAGGATGGAAAAAGGCGTCTTGCATATTCGCTCACCA
AGAGTTTGCTGGATGCAAGATGGGGCCTATCGTGTCAACAGAGGGAGACGTTTGGAAACCAGAACACCCTCTCGTGATAT
CTGGGCACATTCACGACTCGCAGATTGTTGGGAAGAACGTCTACTACACAGGGAGCGCGCTTCAGCACAGTTTTGGAGAT
TCGGAAAAGAAGGGAATCTGGCTCGTTGAATTCTCTGGTCTTTTCGAGGCTGGATGGAGATACAAAAAATACGAACTCGG
AGGAAAGAGAAAGAAAACCATCTATGTCGACGCAAAGGAAGCACTTGAGAAAGTTCCCAACATGGCAGATTCTGAAGATT
CCGACGTTCGCGTTGTGTTCCGTGGCTCGGCTGCGGACTTTGCTGCGCTCAGAAAGTCAAAGAATTTCGCAGAGATTTCA
AAGTCTGCAACAAAGGTCGCGTTCACCAGAGAAGAGCCAGAATATCAAGAGCCAGAGACTGTGGCAAAAAAGACGTATGA
CGAAATCATGGCAGAGTTGTTGGAAGGAGAGGACGAAAGCGTTCGAAGTCTTTGGAAACAAATGAGAGATTAGAATATTT
TTTCTTCCGAAAAATATTGAAACTGTTCCAGAACAAAAGGTAACATTCTTTTTCACAGGCAAAAGATGAGACGAAACATT

TGGCAAGCCCTTGAAAATTACGACACTCCTTGGTTTGAAGGGGCTGTGCGTTCCAAACAATGCATCCCAAACACTGAAAA
AGATGGTCTGGACGTTCTAAGCCATGCCATAGAACTCAGATTTTCCGATGCTGTCTTGGCGATGTGCGCTTGCGGCTTCC
AAGTCTTTCAGCACCATGTCGACAGAGCATGCAGCGCGTTTGAAAAGGACGAGTACGTTTTCTTTCCAGAGCCGAGGAAG
CTTCTCCAGGAACACGTCCCTCTCGTCAAAAAGATGGACAAGAAGACAAGGACAAGTTTGTGGCTGGGAGACGACTCGAA
ACTTCCATCAGATTTTCCAGCAAAGGAAAAGGCGGAATGGGTCCGCTACAGCGCAGAGACACTCAGATTCCTTCTTGCGA
CCCTTCGCCTCTCTGAGGTGGATTCTGGTTGGAGAAGGCTTCCTGCTGGCTTGAGGAACAAGAGAATTCTCGAGTTCAAC
GCGCAGTTTGCTCCAAACACAAGGTACTTTGGTCTTGGAAAAAAGGACTTTCTGTTCTATGCCGTTTCCAAAGGGTATGT
TGCGAGCTCGAGAATGTCAGCTCTGGAATATCCACCAAAGACAAACGCTCTCGTCTTTGCCATCAGAGAGTATGCCAAGA
ACGTGGCAGGAGCCATCAAACTCTCTGGTCAGCAGAACGAAAGGTTCATGCTCCACGGAAGGACTCCCGAAAACATTCCC
GGACTCGAGGAAAAAGACAACAAGCTCCTCCCTCAGATGCAGAGAGTTTTGGACTCTCTTCAAATTGTGGACACTTTGGC
CAAGATTATTGGACAAGAGCAGGTGAACGGAAGTTGGGACGGAACCACTCCAGTTCTCACCGCTGTGGAAACAAGGTCGT
CGATACTCTTGGAAAAACTTATCAAGAGAGGAGCGTATCTCAACACAGTGCCAGAGGGAGCCAGGAAACTTCCTCTCACT
GCTTGCAGAGATGCAGACTTGTCTGGAATTCTTGTCTTGAACGGAGCGAATCCTGACCTTCCAGATGCGGACGGAGAGAC
GTGGCTTTCTTGGAGCGTTCGTTGTGTCGCTGAAGCAAAGGACCAAAGAACTTCGAACAGGGCGATGGAGCTGTTGCGAA
CGTGTGTCGACTCGAGACCGAACCCGGACGTTCCTGCGAGGGAACATCCAATCATCACGGCGATGAAAGCAGACAACGTC
CAGACACTCGAACTCGTTTTGAGTTCGGGCATCAACCCAGACGAGGGAGTTTTCTGGGCAAAGGACAACGGGAGAAAGGC
GTCTTTGGATAGACTTGTCGAGTACAAGAAGTTGTATGACCTCAGCCTCGACGACCTCGTACAAAAGGTGGAATCTCTCC
AAGCTTCTCTCAAGATCGCAGAGAAAGAGGTTCAAACCATCTCCGCGTCGATGTCTGGTCTTTCTGACGAGGAGTCGAAA
GAAATCCTTCCATCTCTCATCGATGCAAAGAGAAAGAGGACGGGAGTGCGAAAAGAGCTCGACTCTGTCACCAACATTTT
GAGGCTGTTCCTGCTTCGGAGTCAGCTGGATTTCTCAGACTCTGACCTCTTGAGACCGAGGGGTGTGGCCACATGCCTCT
CTATCATGGGTGAAAACAGAACAAAAGTGGACTATCAAAAAATCAGAGAGAGAAGGGCCTTGATGAGAAGGGAAAGATAC
GCAGAGACAAAACCTTTCGATGAGACACTTCTCCCTGATGGAACAGACGCCGGAAACTTTTCGGATGACCAGCTTGTTTG
GCTTTGGAAAGATGGGAAAGCGACAATCCTTCCAACTTGGGACATTCCATCCATGAAAAAAGATGGTTGGACAAACTCTG
AATTTGAAAGGATTCGAAGCACCCGCATCGCGACGAGAACTCCAGGGTTGGAAGAGGAACTCGAGTTCCTCTTCTCGAGG
CAGCTGATGGGAACAAGAACTCTCATCTCGTTCAAGGAACAACAAAAGGAAGATGAGCAAGAACCGGAAGAACAAGAACA
AGAAGAAGAAAGCGAGGAACCGAGTCGAGAGGAAGACGATGTGCTGGACGCGGCGATGAAGGCGTTTCAAAAACTTAGGC
TCTGAATAAAAAATATGTTTGTGATATTTTTTCTTACAAATTTTTCTAGTTGTAATGGCTCTCAGAACTCCCCGAGGAAT
TCCCAGTATTTCTTTCGACACTCAGCCAATCACCGGTCTCGACCTTTCCGAGACGGGAGCATCTTGGCTCAACGGAATTG
TCTTCTTCCTGGTCATCTTCCTCGTAACTTTGCTCGTTCTCAGAATTTCGGAACCGAGCGGCGTAAAAACTGGAACTGTG
CTCGACCATGGGAAACTCTTTAGAACCGCTCTCCTGTGGAGCTTGGGTATTTTTATTGCAATTTGGTTGCTAATGGTACT
AATGTCCTACCGCCAGTAGCGCGGTACACAACACAAAGGCGAGGATTTCCGCACCCGCGCACAAACAATAAATTTTGGAA
AAAATTTATATCGCGCCTTCTCAAGAACAAAAATGTTGCGGATTGGGAGTTTCCTCCCAAAGATTTTTAGTGATGGGAGT
TTCGTAGTCCTTGATATGCTCAAGAAGCAGAGATTTCCAGTCTATCTTATCGTCCCATACATCTTCCTGCAAAATACGAA
CTACGGAATATCCATTTTTATTGGCACATTCCTCTTTGAATCTATCGGATTTTTGAATGAGTTCTGGTGACTTCCAATTT
AAAACTTGTTCGTAGTGCTGAGGACCGTCGAGTTCAATGATTGTCTTCGAGACGCAGAAGTCGAAGGGGAGAAATTTGTT
TGTTTCTGGGTTCTTACACCATGAAACTTTAAGTTGATGGATTGGGTCTTTGAAATGCTCTTCGAAAAAGGAGAGGAGCT
TCGCTTCAGTCTTGTTTTTGCAGAGAGGACACCATTTTCCGGAAGAGGTGTTGGAGAGAGCAGAAAAGAATTTGTGTTTC
TTCTCACATTTGAACCAGAACTTTTTAGCTGAGCTTATAAACACTTCTCTCGGACTCTGCTCGTTCTTTTCAAAGTCCCA
ATATTCCGCCTTGTCTGAAGAAGCAAAACTCTTCTCAAAACACGTTCTACATTCATCGGAAAGACAGAGTTGTTTATTCG
AACAAAAGGGACAAAACTGTCCATTCGAGACATCGCTCAACCTCGTTTCAAAACTATGTTTGCATTTCCCGCATTCGAAC
CAATGCTTTTTTCCTGAGCTTATAAATATCTCCCTCGGGCTCTGCTTATTTTTTTCAACGCTCCAAAACTTTGCTTTTTC
ATGACTTGCAAAACTCTTTTCAAAGCATGTTTTGCAATCGGGGAAAGAACAGAGTTTTTTTGGTGGGTTAGAGCAAAAGG
GGCAAAAACACCCATTAAAAACGTCATTCAACCTCGTTTCGAAACTGTGTTTGCACCTTCCACAGTCAAACCAATACTTT
TTTCCCGAGCTTATAAACACTTCTCTCGGACTCTGCTTATTTTTTTCTATATCCCAAAACTTTGCCTTGTCTGAAGAAGC
AAAACTCTTTTCAAAGCACAAACTGCATTCTTCGAAAGCGCACAGTCCGTTGTTCGAACAAAAGGGACAAAAATGCCCAC
TCGACACATTGCTCAGTTTTGCTTCAAAACTATGTTTGCATATTCCGCATTCGAACCAAAACTTTTTATTGCTGTTTCTC
GCTATCAACAGAGGGCTTCCATGCTCTTCTTTCAAATATTTTGAGTTTTCGTGGCTTGCGAAACTTCGTTTAAAACAAGG
CTTGCACTTCTCCTCTCCGCAGAGCCTCCCCTTCTTTCTGTTTTCACAGTTCATCTTTTTTCCCTTCGCCCAAGTTTCAC
CTTTTCACTTTCCATAAAATATTTTATGGAAAGCGAATGACTTTCAGCGTTATTTATTTTTTTGATACGAACAAAAGAAA
TGGGAGTTCCGGGTCTCTTTTATTGGATTCAAGGGAAATATCCTCAGCACACTCGCGTCGTCCAAAAGAAGGAGCCAACA
ACAGCTGACAATTTGTATATCGATGCAGTCTGTTTTGTGCACAGCAGCGCTCAGGACGTCTATGGGTATAACGGGTCAAT
CTCTTTCTCTGAGACCTACAAGTCTTTCACAGAGAAACAGAAGGAGATGGAGGTCTTTCGTCTCACGAGGAACGCGATTT
TTGCCCTCACAAAGTACGTCAAGCCAACAAAGAGGTTCTACGTTCTGTTCGATGGCGTTGCGCCTCTCGCGAAGCAGGGA
CAGCAAAGGCACAGGCGCTTCCTCGGCAGTCCGACAGAGGGGTTCGATCCGAATTCCATTACTTGTGGGACTGAATTCCT
CGATAGGCTGCAAAACTATGTCCGAAGGGGTCTCGAAAACTTTTGTTCAAAGGAGAGTTTCCAGCTCATCTTCTCAGACA
CTTCCATCGCGGGAGAGGGAGAGCACAAGGCCCTCTCTTTCGCAAGGGCAAATGGGCCAGAAGAGAGCCATTGTTTTTAC
AGTCCGGATGGAGACCTTGTCATTCTGGCGATGACGCTCGAATTTCCAAAGACTTGGCTGCTTCGAAAGGACCACATCCA
GCAATGGAAACATCTCTTGACAGACATCTCTGGAATTTCTCAAGAGTTTGAGCAGGAAATCCCTATAAAAGATTTTGTCT
TTCTCTCTTGCCTTTTGGGGAACGACTTCTTGCCGAGGCTCGGAATGTTTGAAGGAAAGTTCTCTGACACTGCCGAATTC
CTCCTTGCAAAGTACAAGGAAAAAGGACTTCACATTTTCAGCAGAAAGACAGGCAAGTTCCTGGCTAAAAACACCTTCAA
AATTCTCAAGTTTTTGGGAACTTTTGAGAAGGAGCTGTTCGATGAGAGGTGCTATTTCGAGCTGACGAAACAAAAGGAAG
AGAGGGTCGACAAGACGCTTTTGTCCGCATACGATGCAAAGGGAGGAGTGGACGTCGAAAAGTACAAGGAAGTGTTTGCC
CTCAAAAAACAAAGGTCGAGAGAACAGACGAGAGAGTATTTTCGAGGCTGTTTGTGGGTGACTCTCTACTACACGCAGGG
CTGTCCAGACAGCGGATGGAGCTACAAGTATTTCTACCCTCCGTTCCTTTCCGACGCCGAACAAGAGGATTTGGTCCACG
AACCGTTCCAACAAACTGTTGTTCCGAACCAGCTTTTGCAGCTCCTTTGTGTTCTCCCTCCACACAGTTCTCACCTCTTG
CCGAGGAAGTGGAAGAAATACGCTTGCGTCTATGAGAAGAAGGAATTGGCAGAGTTTTATCCAAAGGAAGTTGAGGTGGA
TCTTGAGGGGAAATTCAGGGACTACGAATCTGTTCCGCTCATTCCGCATGTGGACATTTCGAGAGTTCAGAGGGAAGTTG
AGAAGATTCGTTGAGAGAGAAGAAAAATATATATTTTGTTTGAGAAAATATATGAAGTGCGAGAATTTGCTTTGCTCCAA
AAAGTAGTTTTGCGCAGACGAAGACGGGGAGAAGACATAAAAACGGTAGTTTTGCGCAGTAGTTTTGCGCAGACGAAGAC
GGGGAGAAGACATAAAAACGGTAGTTTTGCGCAAAATGGACGACTAGGGGGTACCCCCTAGTCGTCCAAAACATACTAGC
ATATAAAAAAATTTTATATGGCCGTATATAAAATCATTGACATTTTTTTTGCACCGAAAATGATGCAAAAAAATACTGAA

TAAAGAACAGTCGAGCATAAGTAAAATGTTCACATGTGAACCTTGTTGTTTTATGACGAAGGACATCTCGAACTACAAAA
AACACCTGTCGACGAAGAAGCACGAAAGACTTTGCTCACAAGACTCTGCCGACCACAAAGAGAAATATTTTTGTGAAACG
TGCAGTTATTTCACAGACAGAAAGTCGTGCATCGACCGTCATTTGAGCTCTGAAAAACATAAAAAGAAAGTCTCCCTTGC
TCTTGGACCCAACGGAAAAAAAGCTTGCCTCTCCGAGGACAAACTCAAAAAGTTGCTCTCGAGAATTCTCGAGAGCAAAG
AAGAAGATGAACTCCGTAAGATAAAAGACGAAGATGGAAGGAAAATTCGCGTAAAATATGTCGTGAAGGAGAGAAGTTCT
CTTGTGAAGGATAGAATTTCCTATTTTGAATGGATAAAAGGAGTTTTGGAATTTTTTGAGGTAAAAGCGAGAGAAACAGA
GGGAGGATTTTTTGTGACATGGAGAACGGCCACATATATGGTCTCAAGAAAAGACTTTTTTGTTCTTTCTTTTGAACTCG
CTGTGGGGGGAATGCAGAAGTCCCAAAGAGCCGCATCGACGGAATACGAAAACATCGCGAAAAGAGGAGGATACAATATC
CCAGCAACTGTGGGAAGAATTGTTGGACTGATAAACCAAGAAAAGATGAAAATGGTTGTCTATGCGAGCCCAAAAAGATG
TCAATACCTCATTATCCACGGCAAAGAAACTCTCGAAATCACGGATATCATCAGGGACAAAAAACTCAAATTCGACAATC
TTTCGGAGGGATTTTGTCGTTGGTGGGCGAGCGGCGAGCATTTTAAAAATGCTTTCACTGATAATTTGGTTGTGGTGCCG
AATGGAGACGTATTCCCGGATTGCGAAACAAAAAGAGTCAAAGAGAAAATATTCGAAGGAGAAAATTTTATTGGGGAGGA
TTTCGCAGGCTACAGTTCCAAGTGCGTTATCGACGACGAAAAGTACGTTGCCTTTTTGGATAATTTTTCCATCAAATTTT
CACAAAGGGAACTGCATCCGGACATCAAAGCGGTCAAGGCTCTCAGAGGAACTTTATTCTCTTTTTGGGAAGAAACACAA
CAAAAAAAGCTCCCTTTGGAAGCGTACGGGGCGGTACAGTCAAAAAAAGAGCAGATTGAGAAAAACATCATCTATTCGCA
TTTCGGCATCGTGAAGAGGATAAAAATCGCGTCTTTGATCGTGTCCAAATTCAAAGATGTATAATTGAGTGTTTTCCAAA
TTGCGAAGATTCCAATAAATGAGCCGAAACAACCCTGTGTTCTCCACAAATGTGTGGTGCGCTTGTGGTGAAATTATGGA
CGAGAAAGAAATTCTTCCCCATCTTTGTGCTCATCGTCGGAACGCAGAGTATCTTTTTTCAGATGATGGGGGTAAATTCA
CGTGCATGGCATGCAAGAAGGAAACGGACAATGTTCTGGGTATTATCAACCACCTACAAAAAGAAATGCACAAAGGAGTT
GTTTATTGGAGCAAACAAGACAGAAACTCTCAAAGAGCTCTGGAACTCTTTCAGGAGAAATTCTCCAAAAAAGTTGTCGA
AGAGGTCACGGATGAAGAGGTTTGGCTCTACGAATTTTGGAACGTAGAGGACTATCTGCCCAAAGGAGAAAATTCCGAAG
TTTTCTGTAATAAAAATTAGCCGGAAAAAATAAAATATACCTGAATATATTTTATGGACAAACAAAGCACAAATTTTGAC
GTTCCACTCCTTCCGCATGTGAACATTTCGAGAGTTCAAAAGATTCGTTGAGAGAGAAGAAAAATATCACCCTTTCATTC
TTTGGATTTGAACCAAGAACGAATTTTTCCCTCTCCGTCATTGAAAAATCATGAAACTGCAAGAATGAATTTCTTTTCTC
TGAACAAAAGGAATATAAGATGAGCCAATGTGCGACTACTCGAAGGATGGAGATAGTTTGCAGTTGTGGGAAAGTTTGTA
GACAAAAAGATGTTCTTGAACACCTTGGAGACCACCAAGAAAAAATGGAGCTGTGTTCGGAAGTGGAGGATGGAAAAGAG
TTTTATTGTGGGCCATGCAACCTCATGACAAAGAACCTTCTCACTTTCAAAAAGCACCTCACAAAAAAGACTCACAAAGC
CGCGGTTTATTACGCCTGCGTTTTTCTTTTCACGAAAAACGCCGAGATAGCGAAGAGAGAACTTTTTTCGAATCACGACT
GGAAAACTGCCTCAAATTTCATCCAAACGGAATATCTTCGACAGGTGGGTACCGTCTACGACGTGATGCGGCCCGCGAAT
ATCCTAAAGAACGAATGATTATTTCTTTTCTTCCAAAAGAAATCAAGCGAACCCGACTCCTTTCGAACCTTTGAACACAC
ATACAGCATGCAAGAGAACAAGAACATCATCATCCATCTCCGAGGTGCGATCGGTTCTGGAAAAACAACCTATGCCAACA
AGCTCAAAAAGTTTGTTGAGGAAAACAATGGACGGTGTTTTATCGCTGGAACGAACGAACTCTGTGGACAGGGTAAATCT
CCTCAACAAGCCTCTTCCATCGTCGAACAAGAGATTCGCGATTGGCTCAAAGGCGTTGTTCCAGAACAACTCAATGTTCT
TGTTGTGGACACTTGTGGTGAAAAGAAACACCAAACGGTCTTTGGCATCGACGTGTCCTCTTGGAAGTCTGTGGATGTTT
TTCCCAACAAAACACAGAATCTCAAGGGCTATCTCTCTTGGTCTCTTCGCAACGTTCTCGAAAGACCAAAGTTTAAGAAG
GGATGCGGTTATTGGCTCAACCCGAGAGACAATGGGATAGATGGTCCAGAATTTTGCTATAAACTTCACAAAGAGAAAGC
GCAAGCGCTCTTTGGCAAAAGAGGATATCCGAATCTCTTTCCCGGAAAAGGCGAATTTCCGAGGACGACGCGGGCTGCTT
TGAACCATCTGAAAGCATCTGCCGATTTTTATGAGATGGAACTGAAGAGACTCGGGGAACCAGATTTTTCGTGGGTGTTG
TGAACAATACAATATTTTTGAAGACAAAAATATTATTCGGAGGTTTCCCAAAGGTTCTTGGTGATGGGAGTCTCGTAATC
TCTGATGTGTTTGAGAAGTAGAGATTTCCAATCTATCTTGTCGTCCCATACATCTTCTTGCAAAATTCGGAGAACGGAAT
AGCCATTATTTATGGCTTGTTCTTCCTTGAATCTGTCAGATTTTTGAATGAACTCCGGAGATTGCCAATTAGAAACTTGT
TCGTAATGTTGCCTTCCATCAAGCTCTATAATGGTCTTTGAGACGCAAAAGTCGAAAGGGAGAAATTTGCCTGTTTCTGG
GTTCTTGCACCATGAAACTCTGAATTGGTGAACGGGGTCTCGGAAATGCCATTGGAGAAAGGAGAGGAGCTTCGCTTCAG
TCTTGTTTTTGCAAAGGGGACACCATTGACCGTTCGAGATGTGGTGGAGAGCAGAAGAGAATTTGTGTTTCTTTTCACAC
TCGAACCAGAATTTTTTATGTGAGCTCAGAAAAACTTCTCTTGGATTCTGCTTATTCTTTTCGACATTCCAAAATTCTAC
TTTGTCTGAAGAAGCGAAACTCTTTTCAAAGCATATTCCGCATTCAGAAGAAGAGCAGAGATTTTTTGGTGGGCTAGAGC
AAAAGGGGCAAAAGCTCCCACTCGAAACTCTATCCGACCTTATTTCGAAACTGTGTTTGCATTTTCCACACTCGAACCAG
AATTTTTTATGTGAGCTCAGAAAAACTTCTCTCGGATTTTGTTTATTTTTTTCAAAGTTCCAAAATTCCGCCTTGTCTGA
AGAAGCGAAACTCTTTTCAAAGCAAATTTTGCATTCATTGGAAGCGCAAAGTTTATTGTTCGAACAAAAGGGACAAAAAT
GCCCGTTGGAGATATGACCCGAAGTTGCCGCAAAACTGTGTTTACATAGCCCACATTCGAACCAAAACTTTTTATTTGAA
GTTAAAAACACTTCTCGTGGACTCTGTTTGTTCTTTTCAATGCTCCAAAATTCTGCCTTATCGGAAGAAGCGAAGCTCTT
TTCAAAGCATATCTCACAATCATCCAAAGAGCAGAGTTGTTTGTTCGAGCAAAAGGGGCAAAAACTCCCATTCGAGACGT
TGTTCAAAATAGCTTCAAAACTATGTTTGCATGTTCCGCATTCGAACCAAAACTTTTTATTGCTGTTTCTCGCGAGAAAC
AGAGGACTTCCGTGCTCTTCTTTCAACCACTTTGACTTTTCACAAGAAGCGAAACTTCTTTTAAAACAAGGCTTACACTT
CTCCCTTCCGCAAAGTCTTCCTCCCCTTCGAGTTTCACAGTTCATCTTATTTTCCTTTCGTCTAAAGTTTCACCTTTTCA
CTTTCCACAAAATATTTTTGACAAAAATATTTTATCTCCTCCTCATCACAAGTACAAGCAGCAAAATAATCAATCCAATT
CCAACCCCGGCCACAAGCAACAAACAATTCTCTTTTTTCATTCCACACCTCGCAACAGCCTCCTGTTTTTCTTCTGGAGA
GCGAATTTTCTTCATCACGTTTTTTATGGCGCTTTTTCCTGGGAAGCTCGCCGTCCATTCATTGTGCCATAAATGCACGA
CATAAGGAATTTTTTCCTTGCATGGCGGATTCAACAGATCGCAGAACCTGGAAACGCCTTTGAAGCCCAGTTGCTCCTTA
TTGGTGAAACCGTTTGTCACTGAAAAAAAGGCGCATGGGTCCGCAACTACCACATTTTCAAAGGCCCTTGGATATTTTTC
GAAAAAGAGTGTCATCGCCAACGGACCTGTGTTCATCACTGGGTCCGATTTTGGGTCGTAATTGTCCGCGATAAAGTCTA
TTAGTTTCAGCCAAAACTCTTCCTTGGGCGGAGAAATCATGAAAGCGTTGCAAAGAACGACCTCTCTTCCATACAAAGTT
CGAGAATGTTCCAAAGGTTCCCTTCCTAAAACTATTTGTCCTTTTTCGAGCCAATTATCTATTCTCCGGAGAGGCAATGT
ATCGATATCGGCATAGACTCCACCAATTCCGTACAATATCACATACCGCGCAAAATCCACTCGTTCGATATTTTTTGACA
TCGCATCGTACAAAGCAAGATGCTGAGGAACATATTTTTTCACCGCTTCTCTGAGGTCATCGTCCAGATAAAATTTATAT
TCGAAGTCCGGGAACATGTCCCTCCATGTTTTGACGAGGTCCTTGCAAGACTCTGGGAGCTCTTTGCTCTTCCATGTCTG
ATGCAGAATTTTCGGCGTCGAAAACTTGACCTTTTTCATCTCTTACTTCTTCTTCACGCCCAACATATCTGCATTCTCAA
ATATTTTCAACAAAATATTTTATTCTATGACCGGACGAGCAAAGAGCTTCTTTATCTGTTTGACTGTCTCGGAATGGGCG
AGATGAGCTCTTCCCAAGTTCCGCAAAAGTTTATCGTCTTGCACGAGGACACTGGATTTTCTCTTTGGAAGTCTCTGTCT
CACTACAGGAAGGCCAAACATCTTCCTTCTTACGTTCAAGGTCTTGATGTGTTTTTCTCTGTTGTAAGAAACCACAAGTT
GAACGAGGATCGACTTGTCCGTATTCAGCGCTCTTTTGCAAATGTCCATCGCTCTCTCCAAAAGATTCGGCCTTTGAACT

GGTTCTTTTCTCTTGACAAATGCGTCCATACTCTGTTGGTCTTTTTGCGGAATGTGGTCATTCATGTTTTGCTTCCGAAA
GAGATTCCTTAAAAGGTCCACAACGTCCTTGTCTCCGTCTGAAACCTCTCCTTCCAATCTCTTTATCTTCTCGCAAAGTT
TTTCGTATGGAATGGTCTGTTTCTCCCAGACATTCAAAAGTTCATCGATGGGCTTCATCAACTTTTTGTCTAGGTACAAC
CCTGTGTCGATGCGAAGACCGAGTCTCTTTTTATTGTCCAGGTACCAGGTGAAATCCTCAATCTGCTCTGTCTGAAGAAT
TTTGTCTCCCTTCTTCGCTCCAGGAACTTCCAAAAACACAAACTCGAGGCGAGTGTTTGGGATGACCTCTTCGCCTCTTG
TCTGCATCTTCTTTGCGAGAGCAACGTTGGACAAACCCTTTCCCTCGTTCTTGTACTCGTCAAGAGCTCTGATAGACTTT
GTGATAACGTATTTTTGGTCTGGAAGAGCTCTTGCCATCATCAGGTAGATTCTATCCAAAACATTGTGGAGGATCTCTTG
CTGTGGTCTCTTTTCGATGAGAGACGTGACTGACTCTCCGAAAAGGTCCCGCGCCAACAGACAATTATCTCTCCTCGTGA
GGATGATGCCCTTTTTGTCTATGGCGATGACCTCTCCTTTTTCGTTCACGATCTTTGTGTAGTATCTTTTCTTCGAGAGC
AAGAAAAAGTCCTTACAGAGTTTCTCAAACTGGAGGTTCATCGGAGCCACAAGATGCTTTGAAACCTCTTTCCCGACCAT
GTTTCCAAACTCGATGAGCTCTCTCGGAGTCTTTCCTGGGTAGTGAATCATACAGGAGTCTGTGTCGCCATATACTAGGG
TTCCATCGGGCCATCTTTCCAATATCATCTGGACGACTTTTTGAATGTCCTGCCTTCCGGCTGTCGTCACAGCCGCCGCC
GCTTCTACCAAAGGAGCAAGTCCCTGTGTCGCTCCAAGAGCTCCGTATCCAGAGTTTGCTGCGATCTTGAGAGCGAGCTG
TGTCGAGTTGAACACTTTGACCTTTGCAAGAAGTTCTTTCTTTTTCTCTGGGTCGGTTTCAGCGTCTGCGAGTTTTTGCA
TATCTCCCATCATCTTTTTGACAACCTTTCTCTGTGTCAACACGCCACGAAGCATGCGAGGCAAAACTCCCTCTTCGACG
CTTCCGTCGTCCAAAATTCTTGACTTTTTGAATCTCTGGACAAAGTGTCCACAAACAACTTCTTCATCCTTCTTTTTCTT
CTTTCCTGCCTTGCTGTCTAGAGGGCAACCGCAAAACTCGTGCTGGTCCCATTCGATGACATTGCAATCAGAGTCTGGCA
CCGATTTGTCGTCAGTGCAAAAACTTGTGTAGTCAATGTTGTTTGAGATAATCTCTGAAGGATAAAGGGACTCGAAATCC
CACACGAGGATATCCTCCCAAAGTCCGATCTTTGCGTCAAACACTGTGGCTCCCTTGAACTTGACGTTGGATTCTTCCTT
TTCGTATCCACGAAACTCGACGATGACATCGTCTTTCTGAAGTTCTCGATAGAGGCGAGAAAAGACCTTGACCTGTTGCC
CTCTCGTTTGAAGGTACTCTCTCGGAACACAGAAGACGCTCGCAAGCATGTCCATGTTTAGAACAACGTCTCTTTTCTCC
AGAAGTCGAAGAGGCAGAACAGTGTCCTGAACGCAGTAGTCTGCGAGTTTTGTCACCCCCTCCCTCACAAGAAGGTCGAG
CGCTTTGAAGCTTTTGCAAGTTTTTATCTTTTGACGAAAGTCAATGAGGTACTTTGTGCTGTGTCTTCTGTTGATGGAGG
TGTAGAGCCTCCTCCTTGTCTCTCGAAAGCCCATCTTTCCGCGCTTTTCCAGTGCAAAGGAAATCTCCATGACTTTGAAA
AGCTCCTTTGCGCTGACATCTTTCTTCTTCTCTCCAAGGAACTTTTCAGCCACAAAGTCGAGGGTGTTTTTGTCCAGTTT
CTCGTTCCTCTCAAAGTCGATGTACATGTCGATCTGGGAGATTCCAGGAATTTTGAGAAATTTGAACTCTTGCGTTCCAT
AGGCAGAACTCTTCCATGACATCTTGTCTTCAGAGGCAGGAACAGCGAAAATTCTCGTGACGGAGTTGCAGAGTCTCTGG
AAGAGACCGCACTTTTTGATGCGAACCATGAGATAGTTCCAATCAAACTTTAGGCTGTTGTACCCCACGATGATGTCGGG
GTCAATCTCTGCGATCCTTTCAATCATCGCAGCGAGAAGACGCTTCTCTGTTGGGTAATTCTTGACCACCACCCCAGGAA
TTTTGAGAGAATTTCCCAGACTGAGAACTTCTTTGAACGTTGCGCCGTCGAGACCGCTCACAGTCCATGCGACGTGCGTA
GTAACATTCTCCTTGATGTTCGGATCGGGGAACGCAGAGTGGTTGTGAGAGTACGCCTCAATATCGAAGCTCATCTCTAC
GGTGGGAGCAGAGCCCTCATTCTCGCCAGTAGAGATATGCTTCCAAAAACAAGAGAATTCAACGTCTGCCTTTGAAAACT
TTTCATAGTTCTTTCCAGCCTTTTCTTTGACAGAAATCCATCCTGTGGAGTCGATGGAACGAAGGGTGAACAGTTTTGTG
TACGTGTCGATGTTTTGTTCGTGTGTTCGAAGCTCTTTCGCATCGAAAGAGCCAACGCCGTCTATGCGATGCTGGCGAGT
TTCCATCGCTCTCGCGCAGGCTCTCATCGCAGAAAGAGTTCGGAATTCCAGCTTCAAAACATCCACAGGAATTTTTCCCT
TCAAGAACGACTTTTTCTTCCTCTCGAAAGAGACGGGCGCTCCGCTTTTCGCGCAAACTCTCTCCTGAAGATATTCAAAC
AGGAGACGTTGTTCTCTTTTTCCCCAAGAAATTCCCTTCTTTTTGGGAAGTTGCATGTAGATGTATGGACAAAATTCTTC
GATGCGGGTGCAAACGGTCCTTCCATCCTTCATCCTTCCATAGCAGTAAAAGTTGAGACCTTCCTTTGCGTCGTCCATGT
CCCATTGCCCCACCTTGTAGTTGCAAATTCTATCCATGTCTTTCTTTTGTCAAAAGAAAGAGGAACTCTGAATTCGAATT
TCCATCTGTAATGAACCTTGAGAGCGTCTATCCAAGAATCCGAGATCCGAGAAAGAGCGCTTTCCTTGCTGTCGAGAAGG
CGTCTCCGAGAGACCTCGCCATAGTTTGTTATGTTTGGGCACCTGCCCCACAACTTTTTGAGTTTTGTGTTGCAGAGTAC
GAGAAAGAATGTCAAAAACAATGGGAGAGTTTTAGCAAAATTTCTGGAAGCGTCTCTGAACTTTTTTCAGACACAGTGCA
GGGTCTGTCTTCAAAACACAAAAAGTTGGAGAGAGTTTTGGGTCTTTTGAACTGCATCGGCGTTCTTTTGATAGCCTCGA
GTTACCCAGAAATTTCAACAGAAACTGCGAGCCTCAAAGACAAGGACAAACTCTTTTGGTCCATAAAGCTCCCAGCAAAT
TACGGGGATTATATTTTTGCAATCTCTGACAAGAAGAGTCTTCTCAACTTTTGTTCGGAAAAAAAGGCAAAGTTCTGGTT
CTCTTTCCTCTATAAAACGGCGACGTCAGAAAGAGTTCGTTGGCTTTGCCAACTCGATGGAGAGTTTCTGTATTTTTCCT
GCTTGCTTTCAAGCATTCAAGAAATAAACGGAGAGTTTTTTGGGCGAACGCCTCTGATGTCTACTGTCGCGAGGGACGAC
ACAGAAAGCGCCAGCCTCCTTTTGGACTTTCATGCTTGCAAGAACCTCTCTCATCCAAGATGCGAAACGAACGCCCTTGG
AATGACGAGCACATTGAGCACATTCGAGTTTTTGATGGAGAAGCAAGTTTGCCCAGACATTGTGGATAGGTCGGGCTTTA
CGTTTCTCTCTCGTCTTTGTGGGTCAAAGGGAGTGGATAGAAAATTTTTGAAACATGTCGTGGACGCGTCCAATCCAAAC
GTTCCGCAAAAAACTCTGCCATTGACCTGGGCTCTTTTGCAAGGGGACGAGGAACTGCTCTCTTGGTTGTTTGAGAGAGG
AGCCGACCCCTTGTTTGCAGAGAGAACGTCAGGTGTTTCGGAAACAGAGAGTGAAGTGTGGAGAGAAGCCAAAAGAATTT
GGGAAATGGACATCCCAAGCCTCAAAAAAGAAAAGTCAAAAATAGCAAAGGGACGGACCAGAGTTTTGTGTGAGACTTTA
CAAGTCAAGAGCGAGAAATGTCTTTGGTGGCTGTCCAAACACTTCAAATCCGTCTCTGTTCGGGAAGCCGCCAAAAAGAG
GAACTCGGAAAGGAAGGAACTGTCCCTCTTCGATGGAGGGGGTCTGAACTTTGGAGAGTTTTCAGACTTGCAAATTCTCT
GGTTCCAAGAGGGAGACAAACGCCCCGCTCTTCTTTCTTCTCAAGTCCAAGGATTCGTTGAGAAAGAATCAGAGAGACCG
AATCCAAGATACGACAAGATAGACTTTTTTGCTTCGCGCTCTGTCGTCACAACGATTCCCCTTTCATCAGCGATAAAGCT
GCTCGAATAAAATATTTTTATAAAAATATTTATAAATGGTCTGTCCTTGCTCTCGAAAGAAAAAAAGTCAAAAGGCGAAG
CAGAGGTGTCAAGCAAAAACAGCGAAAGGGAAGAGATGCAAGAACAGCGCTCTTCCTGGGAAGAAATTTTGTTGGACACA
CAGAGACTTTTAAACGAGCCATCCATCGTCGATCGTTTTGTCAGTGTCAAAGAACGCCCACTCCTGCTGGATTCCGTCGT
TTGAGCGATATCCAAGCTCCTTTAGTCCATCCAAAAATTGGTCTTTGATGGATTGCGCTTCCTCCTTCCAACTGTCAAAA
ACTCGGATCGTGAAAAGACTTCCCACCTTTTGCGAGAGGATGTTGGTGCTTACGCCAAACTTGAACTCGCACTCGGATTT
TATCTTTGCAGACACCAAACAAGCGTCTTCGAAAAGCTTCTCGTTTTCGAGGCGACATTCTCTCGGAGGGTACTTGAGGT
GAAACTCGAAATACGTCCCCAAGATGCATTTTTTGGCATCTTCTTTCGTGGCAGGCACTCCTTCGTTGCATTGCATCGCT
TCAACCTTTACACGAAGCACAGAAAACCCACTCTCTGAGAGGTCTTTGGCGATGGAGTTTGCCTTTTGTATCGCGACATC
AGAGGTTCCAGAGCACCACTTGCTCATCATCGCCTGAGATTGCGTATTGTCGCTTCCCCTTCCGCTGCATGCGAAGATTG
CCTTGTAGTTTTTCTGTTTGCAGTAGAAGAGAAGGTCGACTACATCCAGGTTGTCTACGGTCACGTGAATCTCAAAAAGT
CCCTTGATGTTGCAGCTCATTTTCTTTCAAAGGCAAAAGAAAAGCTCAAAAATTCCGATTATTAATGGAAGAGTTGGGCG
CTCTTGTGTTTGTAGAAGAAGAGCAATCTCTTGTTCCTATCGAGGAGCTCCATTTTTTCGATGAGAGGTTTGTCAAGAAA
CTCAAGGACGAAGACTTTGACTCCAATGGCAACATCAAAAACAAAGGGTGTAGCTTCGTGGCGTTCTACGCTCCTTGGTG
CGGTCACTGCAAACAGCTTGCACCAGAGTGGTCAAAATTCGCAGAGACGGCGGCGTTTGTGGACGTGTACTCTGTCAACT

CTGATGAAGAGAAACCTCTCATCGAAAGGTTGAACAGAAAAGTGCCAGATTTTGTTGGTGGTTATCCCACAATAATCGCC
TACAAACAAGGAGAGGCGGTCGAAACGTACGAAGGAGAGAGGAAGGCTGGAGCGTTTCTCAAGGAGGCGATGATGCTGTG
CAATAGATAATTTTTTCTTTTCAGAGAAAAAGAAAACAAGAGAAACAACATGAACAACAACCTTTGCCACGCTTTGAAAG
AACAGGTCAGAAATCTTCTGGAGGACCTTTGCGAAGTGTTCAAAAGCGAACCAGAGAAGAAGGCGGACGTCCGTTTCGTT
CAGATGTTCTTCTCTGTTGTCCCAGAAGAAAAGCTGATGGAACATTTTGTCAAATTTGTCCTTCCTCACGCCGATAAAAT
TCACGCAAAGAACGAGAGCTTCTTCATCAAAAACACCGACCTTTGGAGAGGGCTCCCGGAAGACAAGGTGAAAATGGTCT
CTGAAATGTGGCTGACTGGAAGGTTGGACGCAGACGATAAGCAGATGATGTGGGATTATTTCGAGTGTTTTGTTGAACTT
GCGAAAAGTTGGAGAAAACGCAAATAAGCCAACCTTCCAGATGCTGTAACAGAATGTCTGGAATGGTTCTCTTTTATAGC
AAGCGCTCTCCACACTCGAGAAACGCCATTGAAATCATCAGGTCGAATTCCATACCGGTGTCCATGGTTTGTGTGGATAG
CCAGTCCATCCGTTCCATCATAAAAAATTCTGGAAACTACAAGATCCGAGGTGTGCCCACTCTCTTTGTCATGAACGGGT
CAAAACTTTCCATCTTCGAAGGAGAGAAAGTTTATAATTGGCTCCTTACGCTGGTGAAGGGTGAAGAAGAGCCAGAACAG
AATCCGGACCTCCTTTTTGAAGAAACGCCGATAGGTGATGACGAAGGAGAAGAGGAAGAACCTCCTGTTGTTCTCGGACC
AAAAACTTTGAAGAACCCCACAGGCGAATCCATCAAAGACAGAGCGGCGAGACTTCAAAGGGAAGCGCAGAACGCACTTG
GAATGAAGTACGAATAATTTTTCTTTTCTCAACAAAAGAAAATATTTAGTTGGCAACGTTTGTGAAACGGAAGGTGAACT
TCCTCTGTTTATGTTCGGGCCTTAGCTTGGGAGTTTCATAGGGTTTGATACCGAATCTGTCCTCGTGCAGAAGAACGTCT
TTATAAGTTTGTTGCCACAAATTCTTCTTTTGAATGTCTGAGGTGAAGTCCAAAAATTCAGAGACGATGTTGTGCACAAT
TCTCTGGTTCATTTGAGGCAAAGGTTCCACCCTTTTTTGGAATTCGTTCACAGTGATGGTGTGAATGTCGTGAAGCGAGA
CCACAACTTTCTTTGCGTTGTAGTGTTTCGCGATCTCTTGCGTGATAAGGTTCTGTAAGAATCGCATGTTTTCGATAGAA
AAGAGCTGTCTGTCGACAAACATCGGATAGATAGTGTAGGGTGTTGACTGCATCTTACAACTATGTCTGACAAACCTTTG
CTTGGTGAAAGTTCGAAAAGTCTTTGGTCCGCGTTCTCATCTAAGAAGCAACAAGTGAAATCTTCCGTTTTCGAGCATGT
CATGTCCCACAAAACTGTACTGTTCGAACACACGACCACGCGCGGCGTCTTTTTTCTGGACACGAAAGGGATGAAGATAT
ACACTCCAAACCCAAAGAACCCAGAACAAAAACTCGTCGTTTGCACAAAGGTCTCTGTTGACACAGCAAACGACAACAAG
GTGTGGTTCAAAGGAACGCACATCAACGGAGAAGAGTTCTTTCTCAAGGCGACGATGAAGGATGGAAAGGTCTTGTTTCG
AGGAAATAACGAAAAATGCACGATAAAATATATTTGCTGAATATATTTGAAAGGAGATGGAAGCCGTCCCGTTGTTGTTT
GAGGAAGACGGGTTCGCACCAAAGCCGAACAAGTCGCTCATCGTCAAAATTTTGAACGCTCTAAGAATTTCAACCCTGGA
AGAAGACCTGATAAAAAGTTCGAGAGTTTTGAGGTATGTCCTGATGCATGAACGGGTCATTTTACAGAGCGAGGAAAGAG
CGTTTGCGTTTTACCTCGACGTTGGAAGGAGGAAGCTCGTCTCTCCAAAAATGACCCTTTGTGAAAAGGTCGAACTCGAC
ACTGCAAACGACAGAAAAGTTTGGTTCAAAGGAACGGATGTGAAGGGCCATGAATTCTACCTGAAGTTAACGATGAACGA
TGGGAAACTGCTCTCTGGAGGGAAAAGGGAAAGGTACACGATAGTTGTCCAATAAAATATTTTCTTGAAAATATTTTTAT
GTTGCGTACGCAACAAGTCCGTAGATGACACTTGTGAAGCACCAGTAAGAGCTGAATTCTCTCCCTCGAGTTTTCCACCA
ACTGTAGAACGCAGTGAGCGCACCAGTTGCGATGAGAGGAATTCCCTTTTTCTTTTGCCAAAGTATAGGAGCAAAAAGTC
CAAAGAGATAGAGAGGAGCGAGCCAGCCGGGCATCACACCGTTCCCGGAACGGTCTTCCCAAACGAGATGCCCTTCCTCT
CCGACGACAGTTCGCATCTTTCCAGAAGTTTGTGCGAGACCTATCATGAACAGCACAGCGTACATCGTCGTCATGTTTTT
GAGGAAAGGACTTCCTGTGGCCTTCCAACCTCCATAAGACTGGACGAGTGGCTGCAAAAGCAAGAGAGGCAATATCGTCT
TTGTCAGCGCTTTGTTTGAATGTTCAGAGCCGAGGTTCCACCACATTCCAGCTTCGACAAGTTGTATGGTCGAAAAACAC
AGAAGAAAGGAAGCGTTCCATCTGTCGTAGATGCCGTCTCTTACCCACAAAAGAACAACAAAGACAATGGAGAATGCCCA
CGCGGAGAGAGAAGCTTCGAGAGAGAAGCACATCGTTACAATAAAATATATAAAAGTAAGATGAACACGGGCACTGCTGC
GTTGTTGGTGTTGGCGTTGTTTGTTGTCCTTTTGTGGTTGATGTTGAAAACAAGACCCAGCAAAGAGGCGAGATTTAAAG
GTTCGAGTTTTACTGGACCGAGCACACTAGAAATTCCAAACTCTGCTGTAACGACATCCACAGAAAAAGAGTGTCTTCTC
GTTTGCAAAGCTGTGCCCTTGGCAAACGCGTACACGTTCGACCCGACCACAAAGGTGTGTCAGCCGTTCATTGTAAGAAA
AAACTTTACAGTACGAGAGGACGACAGGAAGAACTCGATGGTTTTTGACTGTGTGTGCTAACATAAAATATTTTATATAA
AATATTTTATTCGGGGACGGGGTCGTATCCCATCTTTTTCCTCTCCTCGTTGTAGAGAGCAAGCCACTTGTCAGAGTAGT
CAGAGTTCTTCCCATCGAGCTCGGCGATCTCGTCATCGAGTTTTTGCGCAGACTTCTTCATCACTTCGATCCGTTCAAGA
TATTCTGTGATGTACTGCTCCACCTGTCTCCGAGCCACCCGCTTCTTTGTGTAATATTCGAGGGTGGTTGGGTCGTCATC
GAGAGCTCCTTCATGAAGAGCGGTTTTCTTTCTCTCTTCGAGTTCACGAGCAAACTTTTTATCCTTCTCAATGTTTTGCT
GGATGACTCTATCCTGAAGTTTCTTTTGATACTCGTCTCGAGGGTTTTCGATGGCCACATCCTCCACCTCTGCCGCGAGT
TCGGTGTTGAGAGTGAAGGGCAAAAGAGTTCCGACCTGTGCGGCGAAAATGGGGGCTGTCGAATCGTGTTCCTTGAAAAT
CTTCATCGCGGAGCGCTCCGCGAGTTCGGCATCGGGCCATACACCGCGAACGCGAACGCAACCGTGGATTCCGTTCACCG
GTTTTTCGAGAGGGTAGAAACTCAGGTTGACAACAGCCTGTCCAGGGATGGGAGGGTCGCTCTTCAACCCACGGGAAACA
GAGGGAAAGGATTTGTAAAGAGCCTCTGTGGCGGCAGCAGTTTCTTCGTCCGTGAGCGGAGGCGCCTTGGGGTCAACGAA
AGCTCGGATCATTGTTTGTCTTACCGTTCTGGAGGGAGAGCGTTTATCTCGAAATTCCAAAGAGAAATTTGGAATTGTTT
CACAGAGTGTACCGGAAGTCGTTCCACCCGAGGCGTTTGCACACCTCGAGAAACTTTTCATCGCAGTCTCGTGTTTTCAT
AGTGTTTTTGAGGAAGCAAAAGTCGTCTGGGTTTGGGACGTACTTGAGATGCTGAAGCAACTTGTACAGCACATACCACG
CATTGAGAGATTTTCTTCTCTCTTTTTCTTTTATTTGCAGATAAACTTTTTCATACACGTCATAACAGTTTGAAAGTTCC
TCTTGATATGCGCTGATGTCTGGGGGAAGCGTCCCATTGAGTTCCCAAAGCAAAAGAGAAAGTTCTTCGTAATAGTCTGT
GAGATTGTTGTCCACAAGAACCATATAGATATGTTTCTTTGTTACGTTCTCGAGGGGTATGTCGTATTTTTTGAGTTCCG
ACTCTATCTTCGCCTTCACCTCAAACGGTATTCTCGTGTTTTTCTTTGCCTGGAACTTCTCAATGACTTCAAGGAACTGG
AGTTTGGTGACATATCCTGGCTTACTGGAACCACAGTTTCTCTGAGAGTCTTTGTATGGAGAAGCCTCTTCCACAACATG
GAAAATTTCGCCGCACACGCCGCATATGAGCTTTTCGTTCTTTTCAAAACTCTGGGAGAGCTCTGCGTTACATGTCCGGC
AGCGGGACACGCGAAGAGCTCTGTCGTCGACCTCGATGTAGTTCCTCGCGATCTTTAAAAATTCGTTCAACAACTTCACA
TCAGCGTCCTGTTCCTTTGGCCTGCTACCAAAGAAGCTAATTTTTCGAACTTTTCCCAAATTCTCTTGATACCTTTTGAG
AAGAGGAGCAGAAAGAGCAGAATATTCTTCCATCTGCAAGAGAGTGTCGTCTCCAGAGATTTCTTTCCGGAGAGTGTACG
CCCTCTCCTTTGCTCTCGAAACGTCCTCCCTGTTTGGACTGTTGAAATAGATGAATTCGACCCTGTCGAGCTCTTTTTCT
TTTTTTGAGATGGTGTCGAGTCTCTGAGCAAATTCTTGTTTTATTTTTTCATCAAGTCCAGGAACGTCAAGCTGGGTCAT
TACAGGTCTGTGCCTTTCGCTGGTTAAGCCATAAGAGAATGAGCTTTCGTACGAAAGCTCATGTTTTCCTCGTGAAAGAT
TTTTTTTGAATTTTTTTTGTATGTTTGAGAGTA
